# Sex-specific blood-derived RNA biomarkers for childhood tuberculosis

**DOI:** 10.1038/s41598-024-66946-6

**Published:** 2024-07-23

**Authors:** Preethi Krishnan, Carly A. Bobak, Jane E. Hill

**Affiliations:** 1 Department of Chemical and Biological Engineering, University of British Columbia, Vancouver, V6T 1Z3 Canada; 2 Department of Biomedical Data Science, Dartmouth College, Hanover, NH 03755 USA

**Keywords:** Transcriptome, Sexual dimorphism, Biomarker, Childhood tuberculosis, Diagnosis, Blood-based, Infectious-disease diagnostics, Diagnostic markers

## Abstract

Confirmatory diagnosis of childhood tuberculosis (TB) remains a challenge mainly due to its dependence on sputum samples and the paucibacillary nature of the disease. Thus, only ~ 30% of suspected cases in children are diagnosed and the need for minimally invasive, non-sputum-based biomarkers remains unmet. Understanding host molecular changes by measuring blood-based transcriptomic markers has shown promise as a diagnostic tool for TB. However, the implication of sex contributing to disease heterogeneity and therefore diagnosis remains to be understood. Using publicly available gene expression data (GSE39939, GSE39940; n = 370), we report a sex-specific RNA biomarker signature that could improve the diagnosis of TB disease in children. We found four gene biomarker signatures for male (SLAMF8, GBP2, WARS, and FCGR1C) and female pediatric patients (GBP6, CELSR3, ALDH1A1, and GBP4) from Kenya, South Africa, and Malawi. Both signatures achieved a sensitivity of 85% and a specificity of 70%, which approaches the WHO-recommended target product profile for a triage test. Our gene signatures outperform most other gene signatures reported previously for childhood TB diagnosis.

## Introduction

In 2022, an estimated 10.6 million people were diagnosed with tuberculosis (TB) worldwide, and 1.6 million died of TB, making it the second leading cause of death by a single infectious agent (*Mycobacterium tuberculosis*) after the recent COVID-19^1^. The incidence of TB in children (age: 0–14 years) is estimated to be roughly 12% (1.3 million in 2022) of the total TB burden. Among adults, TB was diagnosed more significantly in males (55%, 5.8 million in 2022) than in females (33%, 3.5 million in 2022)^1^. Further, sex-specific incidence rates in children vary according to age. For female children, the incidence rate is higher between 10 and 14 years, whereas it is higher at a considerably younger age (less than 12 months) for male children. Sexual dimorphism in TB has also been reported to vary across different countries^2^, but the reasons for this dimorphic pattern remain unknown.

According to the World Health Organization’s estimate, only 30% of childhood TB cases are diagnosed^3^. The high burden and poor outcomes of childhood TB are partially attributed to the challenges involved in obtaining a confirmatory diagnosis. Sputum smear microscopy or sputum culture is the gold standard for the diagnosis of pulmonary TB. However, diagnoses made through sputum smear microscopy are frequently negative due to the paucibacillary nature of childhood TB^4^. Moreover, clinical overlap of childhood TB with other common childhood diseases, such as pneumonia and other lower respiratory infections, may result in false negative diagnoses of TB. These diagnostic challenges are amplified when there is an active synergistic interaction of the TB bacterium (*Mycobacterium tuberculosis*) with other disease-causing infectious agents, such as in the case of co-infection with the human immunodeficiency virus (HIV). Overlapping clinical manifestations, such as cough, fever, weight loss, and lymphadenopathy, often lead to missed or late diagnosis or even misdiagnosis of either TB or HIV^5^. In this regard, the Xpert MTB/RIF assay has been endorsed as the initial diagnostic test for children suspected of having drug-resistant TB. However, it still depends on the sputum samples. These challenges underscore the urgent need for developing rapid, accurate, and non-sputum-based triage and confirmatory tests for TB that are minimally invasive. In recognition of this, the World Health Organization (WHO) created guidelines for developing non-sputum based TB tests for children, according to which the target product profile for a non-sputum-based triage test should have at least 90% sensitivity and 70% specificity, while the diagnostic test should have at least 66% sensitivity and 98% specificity in children with culture-positive TB^6^.

One minimally invasive approach to diagnose childhood TB is by utilizing blood-derived gene expression signatures as potential diagnostic markers for tuberculosis^7–20^. This approach is beneficial for diagnosing childhood TB, as it does not depend on sputum samples to detect *Mycobacterium tuberculosis*. Previously, Anderson et al. identified a 51-transcript signature that classified children with active, culture-confirmed TB from other similarly presenting diseases, with a sensitivity of 82.9% and specificity of 83.6%^10^. Tornheim et al. identified a 71 gene signature from Indian population by for childhood TB diagnosis. However, the 71 genes identified from the childhood population showed less than 50% overlap with genes identified from adult TB datasets, suggesting that a separate biomarker panel may be necessary for childhood TB diagnosis^9^. In addition, Gjoen et al. identified two sets of transcripts (one with seven transcripts and another with 10 transcripts) as potential diagnostic biomarkers for childhood TB^13^.

These study results are promising. However, the implementation of a gene signature with several tens of genes as a diagnostic gene signature is not practically feasible using current technologies. For example, the most widely used diagnostic platform for TB, the PCR-based Cepheid GeneXpert system, are limited in terms of the number of genes (~ 10 genes) that can be detected^21^. Therefore, an ideal gene signature should be composed of ≤ 10 genes. In this regard, the three-gene signature (GBP5, DUSP3, and KLF2) has shown promise as a non-sputum-based biomarker for both adults and children^7,22,23^. Using a specific cut-off for children, the three gene signature showed a sensitivity of 82.1% and specificity of 76.4% which approaches the WHO-recommended target product profile for a non-sputum-based triage test. However, we do not yet know if the same gene signature can be applied to both male and female children or if a sex-specific biomarker panel is more effective.

In adults, sex bias is observed in the incidence rate of TB^24^, bacterial load^25^, inflammatory response, mortality^26^, and response to treatment^27^. Studies have also highlighted a sex-specific difference in the gene expression pattern of infected individuals^28,29^. Although the exact reason for this dimorphism is unclear, Krug et al.^29^ demonstrated using mouse models that PARP1 contributes to sexually divergent TB immune responses and disease susceptibility partly due to the differences in the immune response. This study shows that basic molecular mechanisms could be different between sexes. Whether this sex-specific difference could also be reflected in the diagnosis, remains unanswered. A better understanding of the impact of sex-specific differences on disease diagnosis is important for establishing universal gene signatures for childhood TB diagnosis.

Using publicly available childhood TB datasets from Kenya, South Africa, and Malawi, we aimed to perform a sex-stratified analysis to identify transcripts that differentiate TB disease from other similarly presenting conditions and understand if sex-specific biomarkers are necessary for diagnosis.

## Results

### Overview of pediatric datasets selected for initial analysis

**Figure 1:**
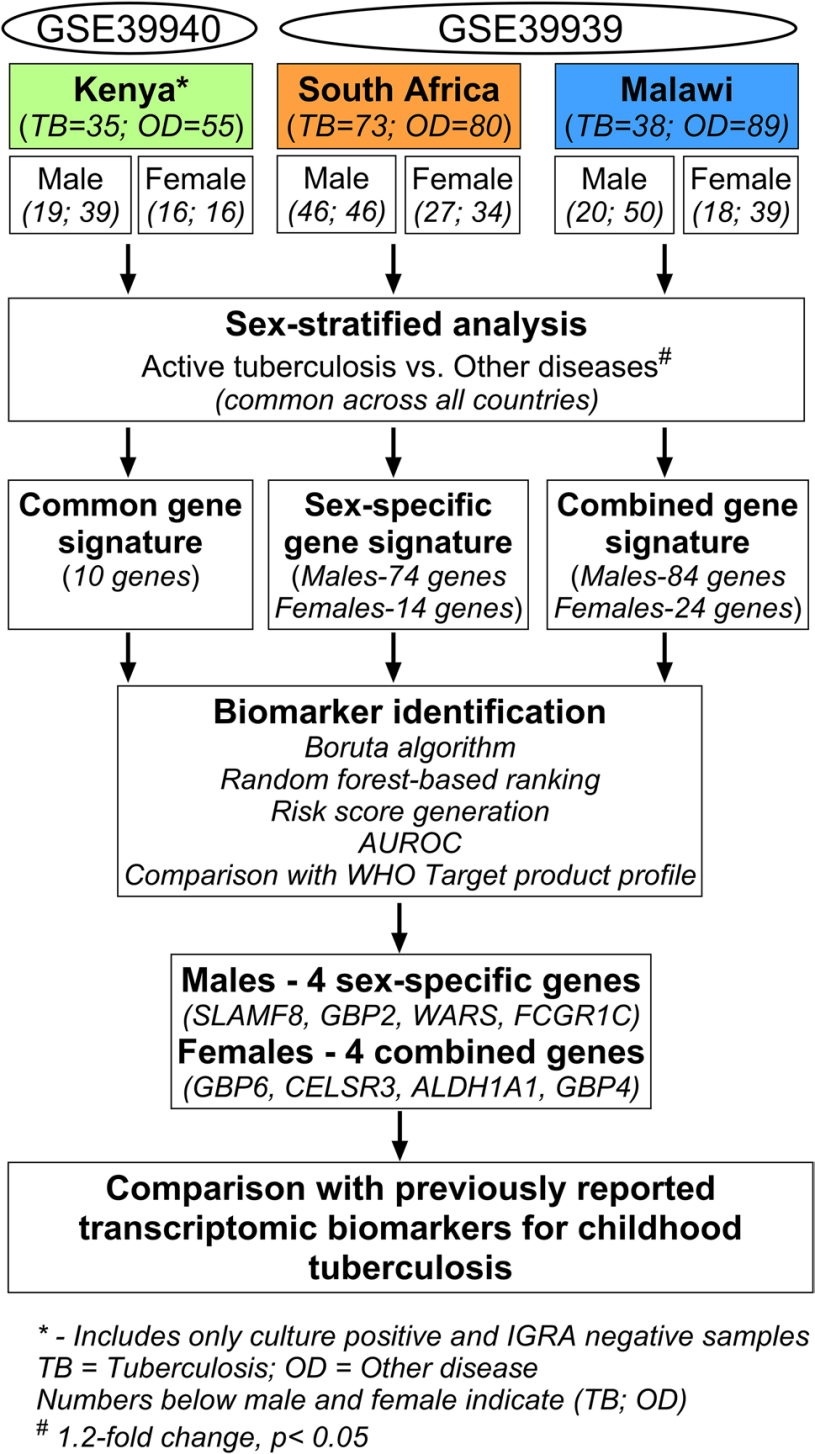
Overall workflow for identifying tuberculosis biomarkers in children aged ≤ 15 years.

The overall study design is illustrated in Fig. 1. Based on our inclusion criteria (see Methods Section for details), we identified GSE39939 and GSE39940^10^ from the National Institute of Health’s (NIH) Gene Expression Omnibus (GEO). These datasets included samples from Kenya, South Africa, and Malawi. Table S1 summarizes the clinical data of the study subjects aged ≤ 15 years. Briefly, the total number of culture-positive samples was 146 and included 51 subjects with HIV coinfection. Further, 44 samples from Kenya that were culture-negative were also included in the study. Patients presenting with various other diseases (OD) but not TB were used as the control group. In the control group, 224 patients presented similar symptoms to those of TB, and this group included 64 samples that tested negative for the Interferon-Gamma Release assay (IGRA), a blood test used for TB diagnosis, mainly for TB infection. Because our study objective was to identify markers that classify active TB from other diseases, we did not include samples that tested positive for IGRA. The control group (i.e., OD group) also included 92 subjects with HIV coinfection. The OD group was further subdivided into lower respiratory tract infection, malnutrition, malaria, pneumonia, and lymphadenitis.

### Blood-derived transcriptome profiles from children with TB disease exhibit a sexually dimorphic pattern

The overall objective of the study was to catalog the gene expression differences between sexes and understand the impact of sexual dimorphic patterns on identifying diagnostic biomarkers. To test if there are differences in the transcriptome profiles in each country^30^, we first compared TB group with the OD group and identified differentially expressed genes from each country. Next, for each country, we performed a sex-stratified analysis, where we split the samples based on sex and compared the TB group with the OD group to identify differentially expressed genes for each sex. For each sex, we compared the differentially expressed transcripts between the three countries. Next, we identified the number of common and unique differentially expressed genes among male and female children across all three countries.

**Figure 2:**
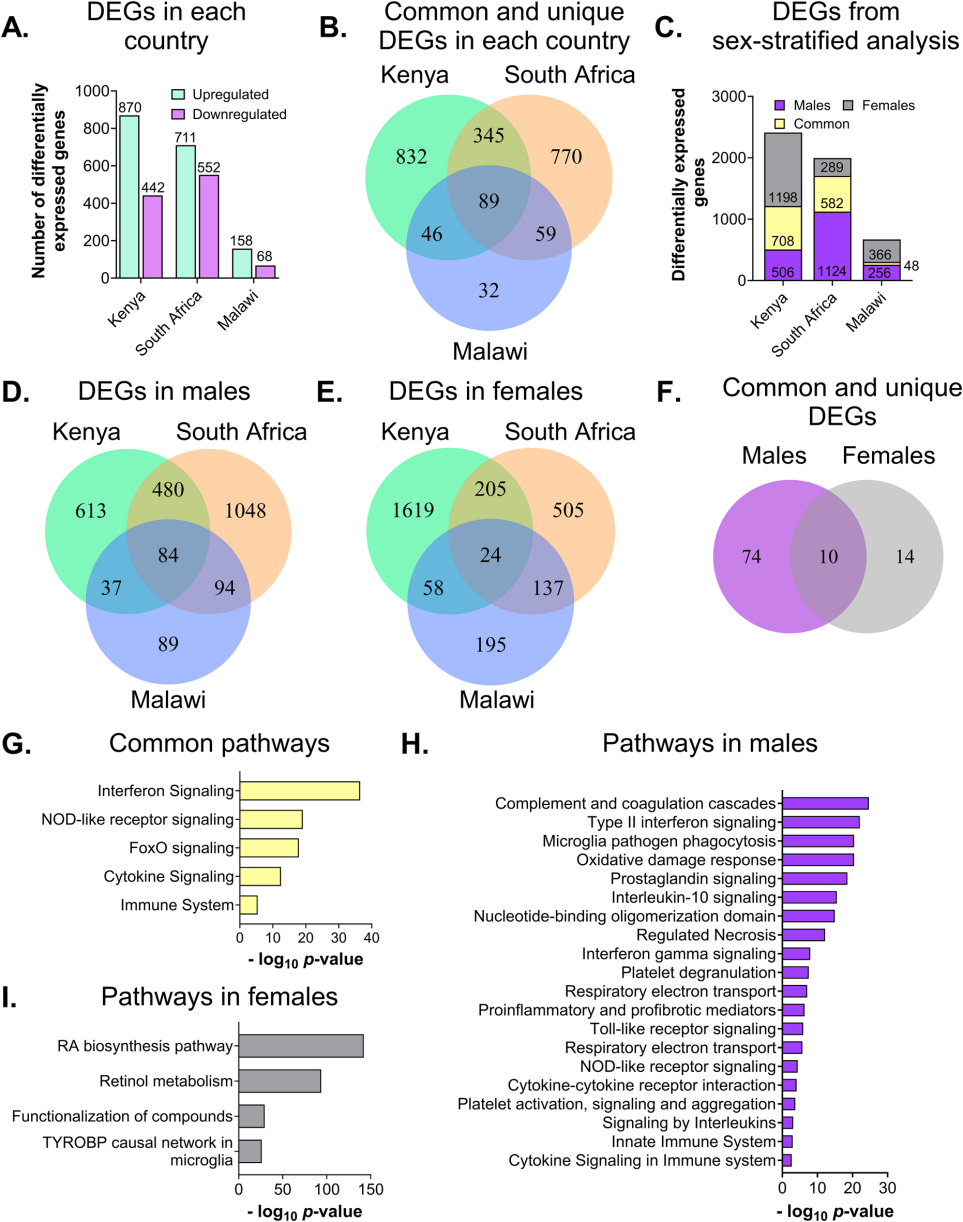
Evidence of sexual dimorphism in blood-derived transcriptome profiles of children diagnosed with TB. (**A**) Number of differentially expressed genes identified in pediatric patients from Kenya, South Africa, and Malawi in response to tuberculosis vs. other diseases. The number of up- and down-regulated genes are indicated above each bar (cyan: upregulation; purple: downregulation) for each country. (**B**) Number of common and unique differentially expressed genes identified in subjects from each country. The number at the center of the Venn diagram (89) indicates the total number of differentially expressed genes that are common to all three countries. (**C**) Number of differentially expressed genes (TB vs. other diseases)—genes that are common (yellow) and unique to male (purple) and female (grey) subjects in each country. The number of differentially expressed genes for each group are specified within the graphs. (**D**) Number of common and unique differentially expressed genes identified in male blood samples from each country. The number at the center of the Venn diagram (84) indicates the common differentially expressed genes in male samples across the three countries. (**E**) Number of common and unique differentially expressed genes identified from female samples in each country. The number at the center of the Venn diagram (24) indicates the common differentially expressed genes in females across the three countries. (**F**) Comparison of differentially expressed genes that are common to male and female samples. The number at the center of the Venn diagram indicates the number of differentially expressed genes common to male and female samples across all three countries. (**G**) Top five pathways identified from the ten common genes between male and female samples. (**H**) Top 20 pathways identified from the 74 common male genes. (**I**) Top five pathways identified from the 14 common female genes.

The transcriptomic analysis of the 34,694 unique transcripts identified 1312, 1263, and 226 differentially expressed protein-coding genes in samples collected from children in Kenya, South Africa, and Malawi, respectively (Fig. 2A, Supplementary Tables S2a–S2c). Less than 10% (89 genes) of the differentially expressed genes were common to all countries (Fig. 2B). Among these, 79 upregulated and 6 downregulated genes were expressed in the same direction in pediatric patients from all three countries.

We then stratified our analyses based on the sex of the child, obtaining 58 (19 TB; 39 OD), 92 (46 TB; 46 OD), and 70 (20 TB; 50 OD) male samples from Kenya, South Africa, and Malawi, respectively. For females, we had 32 (16 TB; 16 OD), 61 (27 TB; 34 OD), and 57 (18 TB; 39 OD) samples from Kenya, South Africa, and Malawi, respectively. Differential expression analysis with respect to TB and other similarly presenting diseases revealed clear differences in the gene expression profiles of male and female pediatric patients from all three countries (Fig. 2C). Notably, 84 transcripts were common among the male pediatric patients from all three countries (Fig. 2D, Supplementary Tables S3a–S3c). On the other hand, 24 transcripts were common to the female patients in all three countries (Fig. 2E, Supplementary Tables S3d–S3f). Among these transcripts, ten transcripts were common to both sexes in all three countries, whereas the remaining transcripts in each sex group (74 transcripts for males and 14 for females) were unique to male and female patients, respectively (Fig. 2F). To understand the biological relevance of these unique (sex-specific) and common transcripts, we performed a functional enrichment analysis and identified the significant pathways associated with transcripts in common, as well as male- and female-specific transcripts. Inflammatory pathways were enriched for the 10 common transcripts (Fig. 2G). Immune-related pathways as well as stress pathways, including interferon signaling, were also among the most enriched pathways in male patients (Fig. 2H). The retinoic acid biosynthesis pathway was the most enriched in female patients (Fig. 2I).

### Sex-specific gene signature adequately satisfies the WHO target product profile

For all analyses in this section, we considered male and female pediatric samples separately. Differential expression analysis yielded both common genes and genes that were unique to males and females. Therefore, to identify gene signatures that can potentially serve as biomarkers, we tested common genes, sex-specific genes, and a combination of common and sex-specific genes (henceforth referred to as a combined gene list).

**Figure 3:**
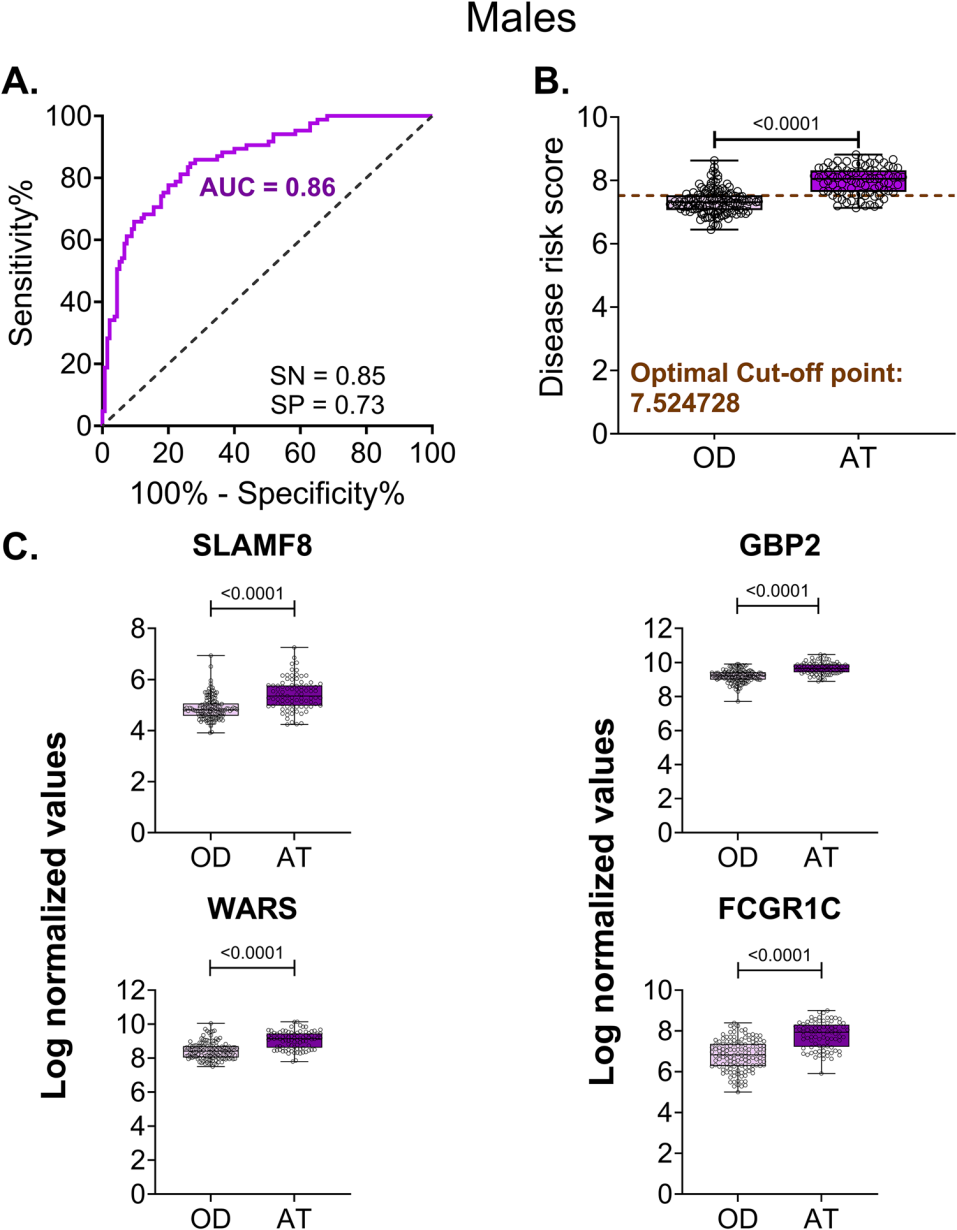
ROC and stratification graphs for male-specific genes in childhood tuberculosis. (**A**) AUROC curve for the risk score using the four male-specific genes. (**B**) Box plot showing the disease risk score cut-off between active TB and OD groups. The dotted line indicates the risk score cut-off estimated using the Youden index. SN = sensitivity, SP = specificity, AT = active tuberculosis, OD = other disease. (**C**) Box plots of the four male-specific genes selected for risk score construction. Log normalized values obtained from microarray are represented as expression values for each gene. *p*-values are indicated above the graphs (Unpaired t-test).

For samples derived from males, following feature selection using samples from all three countries (Tables S4a–S4c), ranking (Tables S5a–S5c), and evaluation of different combinations of genes (Figures S1a–S1c), we selected the gene set with the top four gene candidates (SLAMF8, GBP2, WARS, and FCGR1C) that yielded an area under the receiver operating characteristics curve (AUROC) of 0.86 from the list of male-specific genes. At an optimal cut-off, this gene signature yielded a sensitivity of 0.85 and specificity of 0.73 (Fig. 3A). When the specificity was fixed at the WHO recommended specificity of 0.7 for a triage test, this gene signature yielded a sensitivity of 0.86 which is close to the WHO-recommended sensitivity of 0.9 for a triage test. Further, the gene signature clearly stratified the active TB group from the OD group (Fig. 3B). All four genes were upregulated in active TB samples (Fig. 3C). The optimal cut-off estimated for male risk score separated the active TB cases from each of the different categories of similarly presenting diseases, such as pneumonia, malnutrition, lymphadenitis, and lower and upper respiratory tract infection, in each country (Figures S2a–S2c). The associated AUROC values ranged from 0.7 to 0.97 for all three countries (Figures S2d–S2f). To test if the gene signature can also be applied to patients with HIV, we split the male samples based on HIV status and evaluated the performance of the gene signature based on AUROC, sensitivity and specificity. The sensitivity (0.82) and specificity (0.8) values in HIV negative samples approached the threshold for non-sputum based triage test (Figure S3a). We observed a significant separation between OD and active TB groups (Figure S3b). However, while HIV positive male samples showed a specificity of 0.9, the samples had a sensitivity of ~ 0.7 and did not satisfy the sensitivity threshold for a triage test (Figure S3c). We also observed a significant separation between OD and active TB groups (Figure S3d). However, the AUROC was > 0.85 in both HIV positive and negative samples.

**Figure 4:**
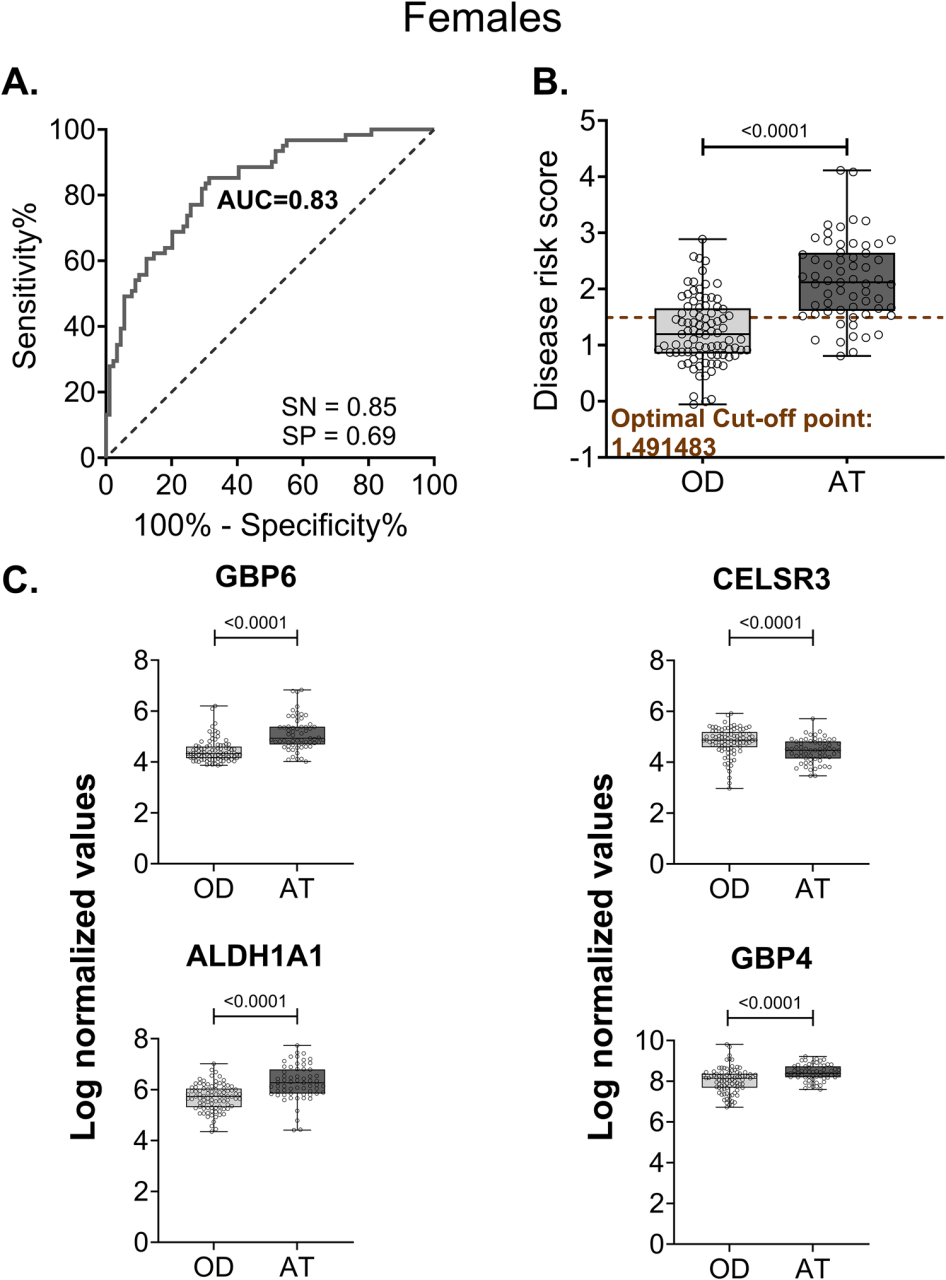
ROC and stratification graphs for combined (common + specific) genes in females. (**A**) AUROC curve for the risk score using the four combined female genes. (**B**) Box plot showing the disease risk score cut-off between active TB and OD groups. The dotted line indicates the risk score cut-off estimated using the Youden index. SN = sensitivity, SP = specificity, AT = active tuberculosis, OD = other disease. (**C**) Box plots of the four combined female genes selected for risk score construction. Log normalized values obtained from microarray are represented as expression values for each gene. *p*-values are indicated above the graphs (Unpaired t-test).

For the samples from females, following feature selection using samples from all three countries (Tables S4d–S4f), ranking (Tables S5d–S5f), and evaluation of different combinations of genes (Figures S1d–S1f), the gene set with the top-ranked gene (GBP6) from the common and combined gene list identified active TB cases with a sensitivity of 0.87 and specificity of 0.74. Likewise, the gene set with the top four ranked genes (GBP6, CELSR3, ALDH1A1, and GBP4) from the combined gene list yielded a sensitivity of 0.85 and specificity of 0.69 (Fig. 4A) at an optimal cut-off value, satisfying the WHO recommended target product profile for a non-sputum based triage test. When the specificity was fixed at WHO recommended value of 0.7, the female gene signature presented a sensitivity of 0.84, which is close to the WHO recommended value of 0.9. Further, the signature clearly stratified active TB cases from the OD group (Fig. 4B). For females, three of the genes, except for CELSR3, were upregulated in active TB samples (Fig. 4C). The optimal cut-off estimated for female risk score separated active TB cases from each of the categories of similarly presenting diseases, such as pneumonia, malnutrition, and lower and upper respiratory tract infection, in each country (Figures S4a–S4c). The associated AUROC values ranged from 0.7 to 0.9 for all three countries (Figures S4d–S4f). Similar to male samples, we also split the female samples based on HIV status. The sensitivity (0.89) and specificity (0.72) values in HIV negative samples approached the threshold for non-sputum based triage test (Figure S5a). We observed a significant separation between OD and active TB groups (Figure S5b). However, while HIV positive female samples showed a specificity of 0.71, the samples had a sensitivity of ~ 0.7 and did not satisfy the sensitivity threshold for a triage test (Figure S5c). We also observed a significant separation between OD and active TB groups (Figure S5d). However, the AUROC was ≥ 0.8 in both HIV positive and negative samples.

When we evaluated the performance of the female risk score cut-off in male samples, although the AUROC was 0.83 (Figure S6a), the sensitivity (0.8) did not meet the WHO-recommended threshold for a triage test. However, we observed a significant separation between the OD and active TB groups (Figure S6b). Similarly, we also tested the performance of male risk score cut-off in female samples. In this case, the AUROC was 0.77 (Figure S6c) and the sensitivity (0.75) failed to meet the WHO threshold for a triage test. Nevertheless, we observed a significant separation between the OD and active TB groups (Figure S6d).

We also noted that the male and female risk scores identified from culture-positive samples did not perform well in culture-negative samples in both male (Figures S7a–S7b) and female (Figures S7c–S7d) groups.

Finally, we tested the identified signature in adult population using datasets deposited in GEO. Due to the limited datasets available with information on sex, we could only test these genes in four datasets (GSE28623, GSE73408, GSE83456, GSE144127). From each of these datasets, we extracted the four genes for males and four genes for females, constructed risk scores, and calculated the AUROC, sensitivity, and specificity values. In males, all four datasets satisfied the specificity value (0.7) recommended by WHO for non-sputum based triage test. However, three of the four datasets did not cross the WHO recommended sensitivity value of 0.9. In females, however, two datasets fulfilled the sensitivity value, and two datasets satisfied the specificity value recommended by WHO (Table S7).

### Gene signature identified by sex-stratification analyses performs better than most of the previously reported transcriptomic biomarkers for childhood TB

We compared our four male (sensitivity 0.85, specificity 0.73) and female (sensitivity 0.85, specificity 0.69) gene signatures with seven gene signatures previously reported for pediatric population as well as one meta-analysis study, which included the relevant age groups (Table S6). Considering that the performance of the reported gene signatures was not evaluated in male and female samples separately in the previous studies, in our analysis, we used the gene signatures reported in each published study and evaluated their performance in male and female samples obtained from GSE39939 and GSE39940, the datasets used for our analysis. Each study generated a specificity of ≥ 0.7 in male and female children, but no study surpassed or approached the sensitivity threshold of 0.9 recommended by WHO for a non-sputum-based triage test. The sensitivity values ranged from 0.01 to 0.73 in males and 0.18 to 0.67 in females. The study by Anderson et al.^10^ that identified 49 transcripts, whose datasets our analysis was based on, was the singular study that had a sensitivity of 0.89 in males. However, in females, the sensitivity was 0.77. In contrast, the findings from our study utilizing the same dataset presents a higher sensitivity in females based on as low as four genes.

## Discussion

This study was conducted to understand the effect of sex-specific transcriptomic differences on childhood TB diagnosis across South Africa, Malawi and Kenya. Sex-based differences in the incidence rate of TB have been reported to be age-related. Whereas a higher incidence rate has been reported for boys less than 1 year and boys/men older than 15 years, the reported incidence rate is higher for females aged 10–14 years^2^. Whether these differences impact the molecular profiles of males and females, and in turn the diagnosis of TB disease, remains less understood. We observed that sex-specific gene expression differences might impact the classification power of transcriptomic markers. For example, when we compared the transcriptomic signatures from sex-specific, common, and combined gene sets, male-specific genes (SLAMF8, GBP2, WARS, and FCGR1C) yielded higher sensitivity (0.85) and specificity (0.73) values, closer to the target product profile of the triage test (0.9 sensitivity and 0.7 specificity) for childhood TB disease. Similarly, in female children, the genes from the combined gene set (GBP6, CELSR3, ALDH1A1, and GBP4) yielded the best sensitivity (0.85) and specificity (0.69) values, closer to the target product profile.

Sex-specific differences have been widely acknowledged to impact disease incidence rates, mortality, treatment responses, and overall disease manifestation in subjects with different acute and chronic conditions^31–38^, including TB, for which a higher incidence rate has been reported in boys/males aged less than 1 and more than 15 years^2,24^. The observed bias has been attributed to various factors, including behavioral and occupational causes and access to health care^39^. Experiments conducted on model organisms suggested that innate physiological differences may influence the susceptibility of an individual to TB^39^. Additionally, while it is true that sex hormones play a major role in imparting sex-specific molecular differences, it is also possible that the inherent biological sex, including the differences in male and female chromosomes, may influence gene expression and these differences cannot be negated. However, considering these physiological differences between males and females, no conclusive data indicates whether a separate gene signature is required for males and females for improved diagnosis. To address this, we first performed a sex-stratified analysis to identify differentially expressed genes between subjects afflicted with TB and other similarly presenting diseases. We observed that only approximately 30% of differentially expressed genes are common between males and females across the three countries included in this study. We further observed that only 10 genes were common to males and females across all three countries; 74 genes were seen as a unique gene set for males while 14 genes were identified as a unique gene set for females across all three countries. Using these gene sets, we determined that the best sensitivity and specificity values can be obtained when a separate gene signature is used for diagnosing TB in male and female children. Our next question was if these male and female gene signatures can be used interchangeably between sexes or if each performs better for the respective sex. For verification, we tested the male gene signature in female population and vice versa. Clearly, the overall performance of the risk scores were moderate in both the sexes and did not approach the WHO-recommended target product profile, suggesting that the potential diagnostic signature might be sex-specific. Additionally, when we tested the identified signature in adult datasets, the results were inconsistent in both males and females, suggesting that the signature may be more effective for childhood TB diagnosis.

To date, a few transcriptomic signatures have been proposed for childhood TB^9,10,13,40–42^, including the three-gene signature developed by Sweeney et al.^7^. However, none of the earlier studies evaluated if the same gene signature could act as an effective biomarker for both the sexes. To verify this, we obtained gene signatures from each study and conducted separate analysis for males and females using the datasets included in our study. While all the gene signatures yielded a specificity of at least 0.7, the sensitivity was ≤ 0.7. The 49 gene signature proposed by Anderson et al. showed a sensitivity of 0.89 in males. However, the sensitivity decreased to 0.77 in females. In comparison, the separate male and female gene signatures proposed in this study generated a sensitivity of 0.85 in both sexes. These observations suggest that the same gene signature may not yield the best results for males and females, and a separate sex-specific diagnostic gene signature might result in improved diagnosis outcome.

Of the four genes identified for males (SLAMF8, GBP2, WARS, and FCGR1C), three were previously reported as differentially expressed genes associated with TB disease in pediatric samples obtained from the Indian population^9^. On the other hand, all four genes identified for females (GBP6, CELSR3, ALDH1A1, and GBP4) were expressed in the same direction in the Indian population, and GBP6 was included as a part of their diagnostic gene set, which included 71 genes. Similarly, Anderson et al. have reported ALDH1A1 and GBP6 as parts of the 51 transcript signatures identified for distinguishing active TB from other similarly presenting diseases^10^. However, because a sex-stratified analysis was not conducted, we could not perform a direct sex-specific comparison and validate our findings in an external population. Nevertheless, we verified if these genes have been reported as potential biomarkers in other published datasets (adult population inclusive). We found studies that have reported a few of the identified genes, but the samples were not stratified based on sex. For example, GBP2 was found to have significant potential in the treatment monitoring of TB^43,44^. It was also found to be a part of the biomarker panel proposed for triage and confirmatory tests for controls vs. active TB cases in the adult population^45^. FCGR1C was proposed as part of a three-signature panel with a sensitivity of 0.75 and specificity of 0.81 to distinguish active TB from the OD group^8^.

Amongst the gene signature proposed for females, GBP6 was included as a part of a biomarker panel that can stratify TB from other diseases in the adult population^17,46^. Similarly, GBP4 was included as a part of a biomarker panel for HIV+/TB^47^. It was also reported as one of the 16 gene panel that predicted TB progression in South African adolescents aged between 12 and 18 years^16^. Finally, ALDH1A1 was shown to classify TB from other diseases^11^. However, the biomarker potential of SLAMF8, WARS, and CELSR3 has not been reported for TB. Altogether, the biomarker potential of ~ 50% of our list of identified genes is supported by the above-mentioned studies. In addition, we are adding a new set of genes that require further validation in a sex-specific model.

We perceive these sex-specific signatures to follow the diagnostic implementation pathway that mirrors the three gene signature diagnostic. The three gene signature was first identified from meta analysis and then was validated by multiple studies. The validation was done using the PCR based Cepheid GeneXpert system, which is a commonly used platform for infectious disease diagnosis, including tuberculosis. Since this system exists in many TB infected areas, such as India, South Africa, we believe findings from this study can be translated to a resource challenged setting. However, further evaluation and validation of the sex-specific genes are warranted in clinical samples, a key limitation of this study. The current risk score cut-off applies only to microarray platforms. Therefore, a different cut-off score specific to the platform will be generated for validation. This is expected, given the exploratory nature of the current study, until we translate the findings to a commercial platform. Moreover, male- and female-specific gene signatures identified in our study are applicable to three African countries, viz., Kenya, South Africa, and Malawi. It is possible, if not likely, that not all genes will be differentially expressed and show diagnostic promise in other populations. Therefore, subsequent validation studies will include samples collected from other geographical locations, in addition to those from the African continent. Similarly, we see HIV as the confounder that could impact the results of these tests and therefore for further validation, we would also include samples that are HIV positive.

In conclusion, we have demonstrated the importance and impact of including sex as a variable for identifying diagnostic biomarkers for childhood TB. Importantly, we identified a minimal set of genes (four genes for each sex) that adequately satisfies the target product profile recommended by WHO for a non-sputum-based triage test and the sex-specific markers were not effective when interchanged between males and females. The inclusion of clinical factors or other existing molecular tests, such as the Xpert Ultra assay, along with these molecular biomarkers may further increase the sensitivity and specificity of these signatures. While blood-based assays are comparatively less invasive than induced sputum or lavage samples, moving towards finger prick samples, which are even more less invasive will be more translational and beneficial to communities where childhood TB is prevalent.

## Methods

### Datasets for the study

We consulted publicly available transcriptomic datasets that included blood samples from pediatric patients with culture confirmed active TB and other similarly presenting disease groups. The following are the inclusion criteria used in this study: children aged ≤ 15 years, (i) whose blood samples were collected using PAXgene RNA tubes, (ii) for whom treatment was not initiated at the time of sample collection, and (iii) datasets that had HIV+ and HIV− samples. Based on our inclusion criteria, we identified GSE39939 and GSE39940^10^ from NIH Gene Expression Omnibus (GEO) as suitable datasets for this study. Patient identifiable data was not available on the datasets downloaded for this study.

### Microarray data analysis and identification of differentially expressed genes

The overall workflow of the study is depicted in Fig. 1. To verify if geographical differences also affect childhood TB diagnosis, GSE39939 and GSE39940 samples were first split based on geographical location. The datasets, collected as a part of a single study^10^, contained samples from Kenya, South Africa, and Malawi. For the analysis, the quantile normalized file was downloaded as deposited, the values were scaled, and a log2 transformation was performed. Both GSE39939 and GSE39940 were generatedusing Illumina HumanHT-12 V4.0 expression bead chip; therefore, these datasets shared probe IDs. Probe IDs were annotated to gene symbols. The median expression value was calculated for genes that had multiple probe IDs^48^. For each country, cases with other (non-TB) diagnosed diseases were assigned as the control/reference group and the gene expression values were compared with those of the active TB group. R studio v4.2.1 (2022.7.1.554) was used for all analyses. Further, differential expression analysis was performed using the *limma* package in R^49^. Transcripts that showed at least a 1.2-fold change (non-log space) difference, along with the statistical significance of *p* < 0.05, were classified as differentially expressed genes. The non-coding genes (any transcript with RefSeq accession type starting with XM/XR) were filtered out and only differentially expressed protein-coding genes were retained for subsequent analysis.

Pathway analysis using differentially expressed genes was performed using the Metascape tool^50^. Reactome, KEGG, Wikipathways, Panther, and Hallmark databases were considered for pathway enrichment and the following criteria were applied for analysis: minimum enrichment of 1.3, *p*-value cut-off of 0.05, and minimal overlap of three genes in each cluster.

### Identification of sex-specific transcriptomic signature score

The differentially expressed genes were classified into (i) common gene signature—genes that were common between sexes in all three countries, (ii) sex-specific gene signature—genes resulting from sex-stratified analyses (male/female specific signature) across all countries, and (iii) combined gene signature—the combination of common and sex-specific gene signature for male and female children (male/female signature), regardless of geography.

Feature selection was performed using the Boruta algorithm (Boruta package)^51^, where the algorithm was repeated 100 times and features classified as “confirmed” all 100 times (Figure S8) were selected. The Boruta selected features were then ranked using the Gini score obtained from the Random Forest algorithm (caret package)^52^. This ranking split the genes into several gene sets, as observed from “elbows” or “kinks” in the importance plot. For each gene set, the risk score was calculated using the normalized, log2-transformed values of the selected genes. The average expression of the downregulated genes was subtracted from the average expression of the upregulated genes, as reported in Ref.^7^, and the final value was defined as the risk score. The diagnostic performance of each gene set was assessed based on a receiver operating characteristics (ROC) curve, sensitivity, and specificity calculated using easyROC, a web tool for ROC curve analysis^53^.

Youden index^54^ was used to calculate the optimal cut-off for the risk score obtained. The risk score was evaluated using the sensitivity and specificity values obtained at the cut-off that maximized the Youden index, and the associated sensitivity and specificity values were compared with the WHO-recommended target product profile for a non-sputum-based triage test (90% sensitivity and 70% specificity). Risk scores with maximum sensitivity and specificity and those achieving values closer to the target product profile threshold of a triage test was considered final. The optimal cut-offs for each gene set were calculated using samples from all three countries. As the reference group included several health conditions (Table S1), such as pneumonia, lower respiratory tract infection, malnutrition, and lymphadenitis, the Youden-index-estimated cut-offs was tested to see if it separated TB from each of these conditions. A specific health condition was considered only if a minimum of five samples were available. However, to construct AUROC curves for TB vs. each non-TB condition, health conditions that had at least 10 samples, wherever possible were considered. Furthermore, the risk scores identified for male and female pediatric samples were tested in culture-negative samples.

All figures were prepared using GraphPad Prism version 9 for Windows, GraphPad Software, San Diego, California, USA (www.graphpad.com).

### Evaluation of previously published pediatric transcriptomic signatures

Eight gene signatures published in seven reports^7,9,10,13,40–42^ were compared with our gene signatures. For this, the list of genes reported in each study as biomarkers was downloaded and a risk score was generated using the formula provided in Ref^7^. Then, the sensitivity and specificity for this list of genes was calculated using the male and female samples included in pediatric datasets GSE39939 and GSE39940 (the datasets used in the current study). Risk scores for all published gene signatures were calculated separately for male and female pediatric samples, as explained above (subtracting the average expression values of downregulated genes from the average expression values of upregulated ones). To draw parallel comparisons while calculating the risk score, we divided the genes into upregulated and downregulated groups, as done in the parent studies, irrespective of the direction in which they were expressed in the datasets used for our analysis. AUROC, sensitivity, and specificity values were calculated and compared with the target product profile and the values obtained from our analysis of sex-specific gene signatures.

### Supplementary Information

Supplementary Information.

## Data Availability

The datasets analysed during the current study are available in the GEO repository under the accession numbers GSE39939 and GSE39940.
