# Supplementary material for: Sex-specific blood-derived RNA biomarkers for childhood tuberculosis
Source: Sci Rep. 2024 Jul 23;14:16859. doi: 10.1038/s41598-024-66946-6 (PMC11263679; doi:10.1038/s41598-024-66946-6)
Supplement: Supplementary file 1 — Supplementary Information. [file 41598_2024_66946_MOESM1_ESM.pdf]

**SUPPLEMENTARY INFORMATION**

# Sex-specific blood-derived RNA biomarkers for childhood tuberculosis

Preethi Krishnan[1], Carly A. Bobak[2], and Jane E. Hill[1, *]

**Supplementary Figures**

**Supplementary Figure S1.** Random forest-based ranking of common genes (S1A - males, S1D - females), sex-specific genes (S1B - males, S1E - females), and combined genes (S1C - males, S1F - females) using the genes selected by the Boruta algorithm. For each category, ranked genes were grouped based on their separation (kinks), represented as gene sets. For each gene set, a risk score was calculated, which was then evaluated using ROC, sensitivity, and specificity. Risk score that matched closely to the target product profile for a triage test was selected (highlighted in purple for males and in grey for females). Numbers corresponding to the risk scores indicate the number of genes included in each risk score. For example, “Riskscore_8” indicates that the top eight genes were used for risk score construction.

**Supplementary Figure S2. Evaluation of male risk score for different countries.** Stratification of TB and different disease conditions in Kenya (A), South Africa (B), and Malawi (C) based on the estimated risk score cut-off for the male gene signature. The dotted line indicates the Youden-index-based cut-off value for the male gene signature risk score. AUROC curves comparing male TB samples and different health conditions for the three countries separately (D-F). SN = Sensitivity, SP = Specificity. p-values are indicated about the bars for each significant comparison (One-way ANOVA).

**Supplementary Figure S3. Evaluation of male risk score in HIV positive and negative samples.**

(A) ROC of the male risk score in HIV-negative male samples. (B) Stratification of HIV negative male samples using the male risk score. (C) ROC of the male risk score in HIV positive male samples. (D) Stratification of HIV positive male samples using the male risk score. The optimal cut-offs are indicated using dotted lines. SN = sensitivity, SP = specificity. p-values are indicated about the bars for each significant comparison (Unpaired t-test).

**Supplementary Figure S4. Evaluation of female risk score for different countries.** Stratification of TB and different disease conditions in Kenya (A), South Africa (B), and Malawi (C) based on the estimated risk score cut-off for the female gene signature. The dotted line indicates the Youden-index-based cut-off for the female gene signature risk score. AUROC curves comparing female TB samples and different health conditions for the three countries separately (D-F). SN = Sensitivity, SP = Specificity. p-values are indicated about the bars for each significant comparison (One-way ANOVA for more than two groups, unpaired t-test for less than three groups).

**Supplementary Figure S5. Evaluation of female risk score in HIV positive and negative samples.**

(A) ROC of the female risk score in HIV-negative female samples. (B) Stratification of HIV negative female samples using the female risk score. (C) ROC of the female risk score in HIV positive female samples. (D) Stratification of HIV positive female samples using the female risk score. The optimal cut-offs are indicated using dotted lines. SN = sensitivity, SP = specificity. p-values are indicated about the bars for each significant comparison (Unpaired t-test)

**Supplementary Figure S6. Evaluation of male and female risk scores in opposite sexes.** (A) ROC of combined female genes in male samples. (B) Stratification of male samples using the combined female genes. (C) ROC of male-specific genes in female samples. (D)

Stratification of female samples using the male-specific genes. SN = sensitivity, SP = specificity. p-values are indicated about the bars for each significant comparison (Unpaired t-test).

**Supplementary Figure S7. ROC and stratification in culture-negative samples using male and female risk scores.** (A) ROC of the male risk score in culture-negative male samples. (B) Stratification of male samples using the male risk score. (C) ROC of the female risk score in culture-negative female samples. (D) Stratification of female samples using the female risk score. The optimal cut-offs are indicated using dotted lines. SN = sensitivity, SP = specificity.

**Supplementary Figure S8.** Workflow for identifying and constructing risk scores for the common, sex-specific, and combined genes of male and female groups.

**Supplementary Tables**

**Supplementary Table S1.** Sample distribution of samples used for this study.

**Supplementary Table S2.** Differentially expressed genes identified between TB and other diseases groups in three African countries: Kenya (a), South Africa (b), and Malawi (c).

**Supplementary Table S3.** Sex-specific differential expression of genes identified between TB and other diseases groups in Kenya (a, d), South Africa (b, e), and Malawi (c, f).

**Supplementary Table S4.** Results of Boruta algorithm for common (a, d), sex-specific (b, e), and combined (c, f) genes. Rows highlighted in yellow indicate the genes selected as confirmed features all 100 times.

**Supplementary Table S5.** Results of random forest-based ranking of common (a, d), sex-specific (b, e), and combined (c, f) genes.

**Supplementary Table S6.** Evaluation of other published pediatric gene signatures in male and female pediatric samples.

**Supplementary Table S7.** Validation of the sex-based gene signature in adult datasets.

# Figure S1

## Males

**A.**

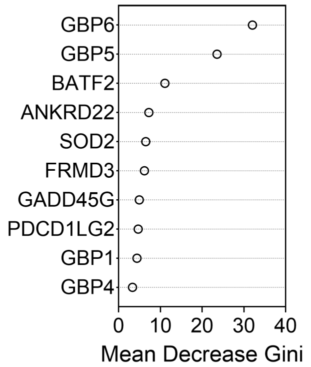


| Common - male | ROC | Sensitivity | Specificity |
|---|---|---|---|
| Riskscore_1 | 0.888 | 0.820 | 0.870 |
| Riskscore_2 | 0.903 | 0.790 | 0.890 |
| Riskscore_3 | 0.897 | 0.790 | 0.890 |
| Riskscore_6 | 0.892 | 0.720 | 0.920 |
| Riskscore_9 | 0.891 | 0.750 | 0.870 |
| Riskscore_10 | 0.886 | 0.780 | 0.840 |

**B.**

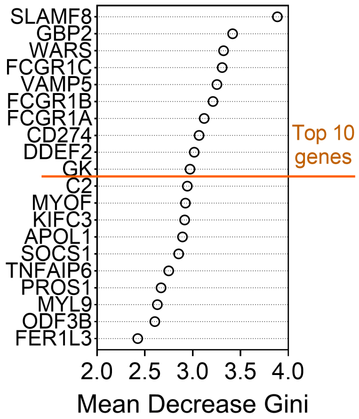


| Male-specific | ROC | Sensitivity | Specificity |
|---|---|---|---|
| Riskscore_1 | 0.782 | 0.840 | 0.680 |
| Riskscore_2 | 0.830 | 0.730 | 0.790 |
| Riskscore_4 | 0.864 | 0.850 | 0.730 |
| Riskscore_6 | 0.860 | 0.850 | 0.730 |
| Riskscore_9 | 0.870 | 0.820 | 0.730 |
| Riskscore_10 | 0.864 | 0.820 | 0.720 |

**C.**

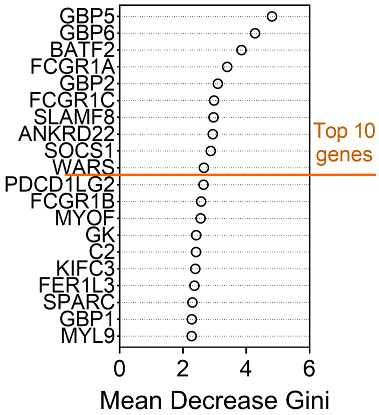


| Male-combined | ROC | Sensitivity | Specificity |
|---|---|---|---|
| Riskscore_1 | 0.889 | 0.770 | 0.890 |
| Riskscore_2 | 0.903 | 0.790 | 0.890 |
| Riskscore_3 | 0.897 | 0.790 | 0.890 |
| Riskscore_4 | 0.900 | 0.750 | 0.900 |
| Riskscore_5 | 0.898 | 0.750 | 0.910 |
| Riskscore_9 | 0.889 | 0.750 | 0.870 |
| Riskscore_10 | 0.887 | 0.740 | 0.870 |

## Females

**D.**

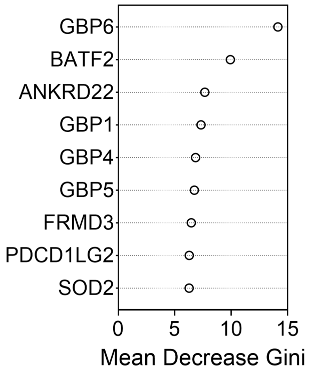


| Common - female | ROC | Sensitivity | Specificity |
|---|---|---|---|
| Riskscore_1 | 0.831 | 0.870 | 0.740 |
| Riskscore_2 | 0.829 | 0.710 | 0.880 |
| Riskscore_4 | 0.807 | 0.660 | 0.880 |
| Riskscore_6 | 0.808 | 0.750 | 0.760 |
| Riskscore_9 | 0.809 | 0.720 | 0.810 |

**E.**

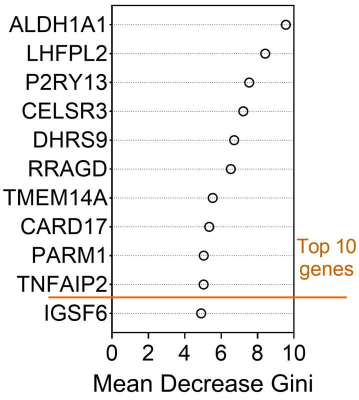


| Female-specific | ROC | Sensitivity | Specificity |
|---|---|---|---|
| Riskscore_1 | 0.770 | 0.670 | 0.740 |
| Riskscore_2 | 0.796 | 0.690 | 0.810 |
| Riskscore_4 | 0.806 | 0.770 | 0.760 |
| Riskscore_6 | 0.810 | 0.750 | 0.780 |
| Riskscore_8 | 0.798 | 0.740 | 0.730 |

**F.**

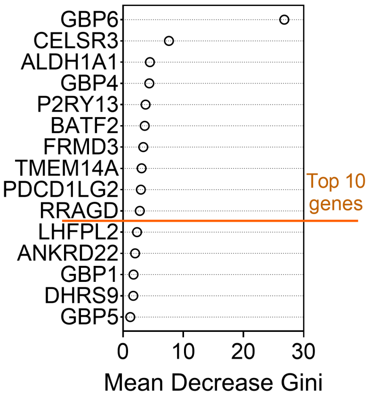


| Female-combined | ROC | Sensitivity | Specificity |
|---|---|---|---|
| Riskscore_1 | 0.831 | 0.870 | 0.740 |
| Riskscore_2 | 0.839 | 0.800 | 0.760 |
| Riskscore_4 | 0.835 | 0.850 | 0.690 |
| Riskscore_6 | 0.833 | 0.790 | 0.740 |
| Riskscore_8 | 0.825 | 0.850 | 0.670 |
| Riskscore_10 | 0.820 | 0.870 | 0.640 |

**Figure S2** Males

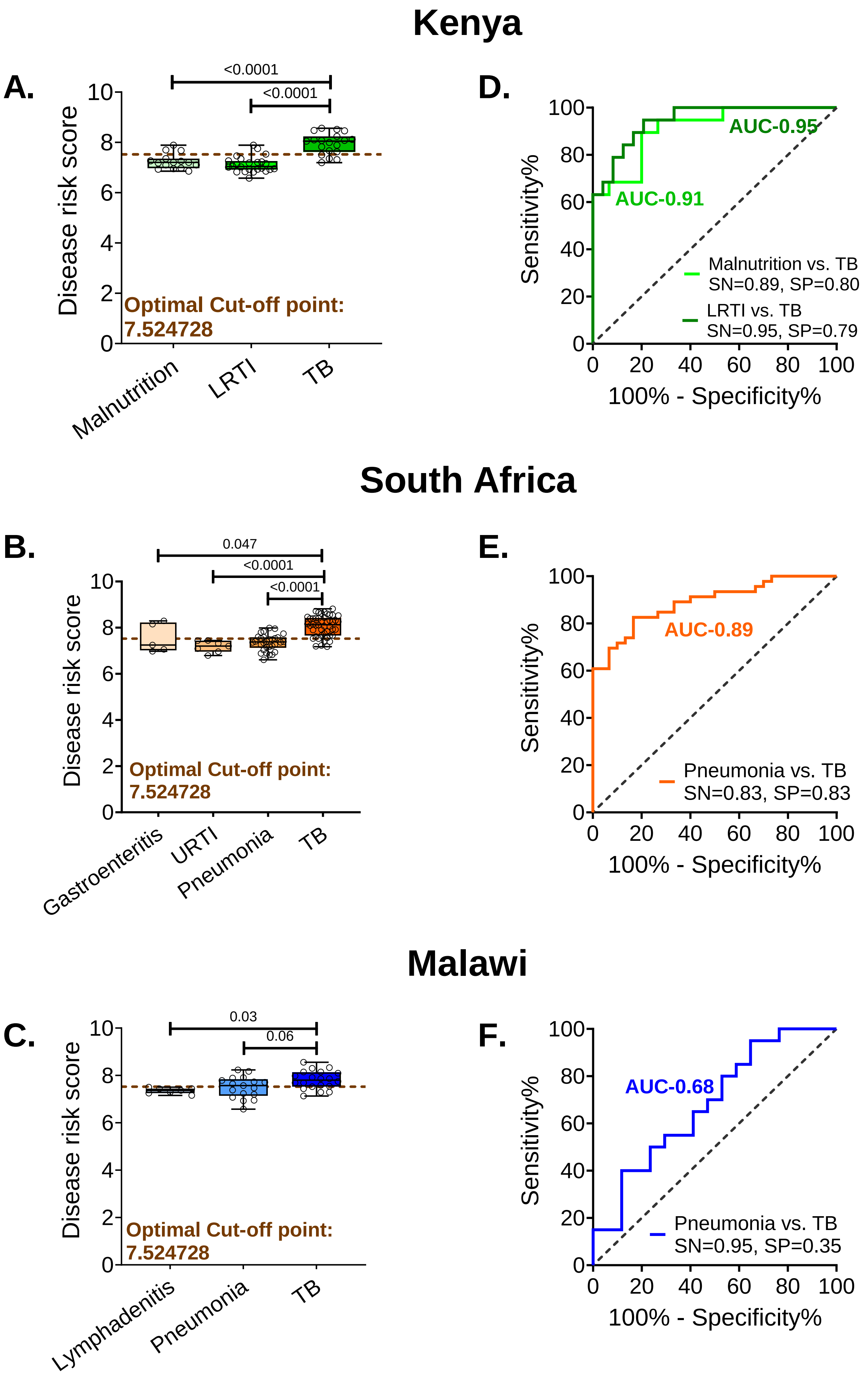

**Figure S3** Male HIV negative samples

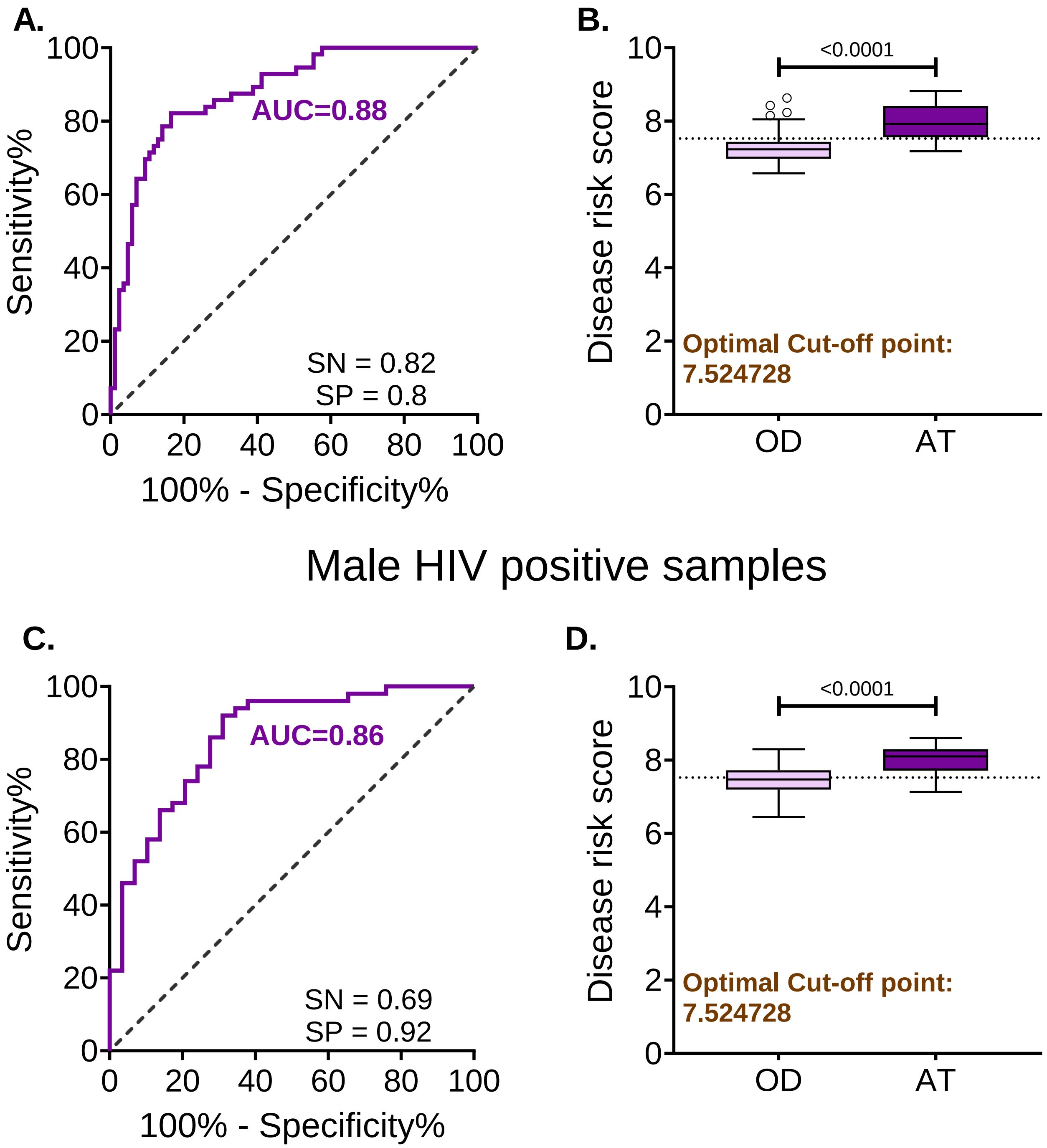

**Figure S4** Females

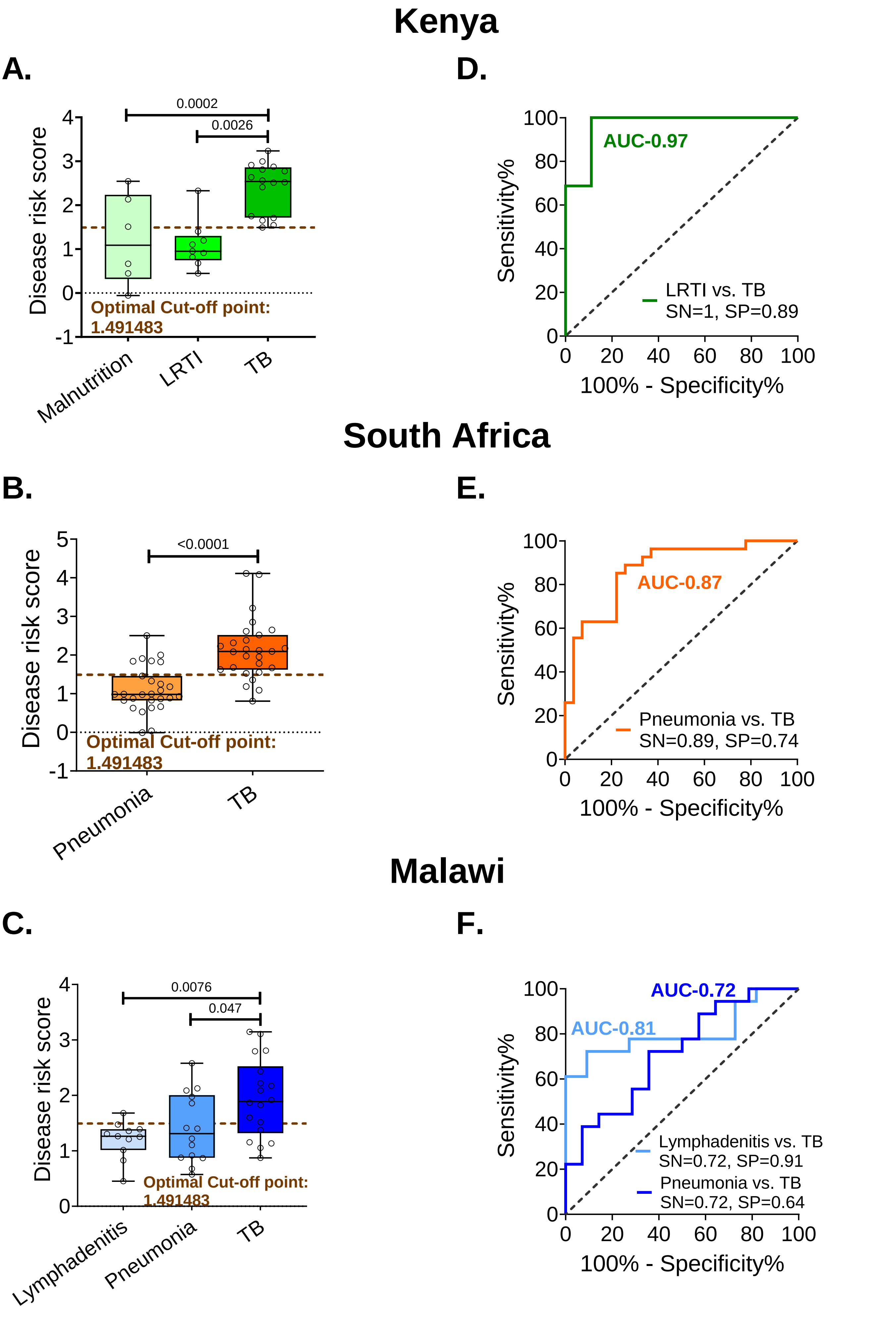

**Figure S5** Female HIV negative samples

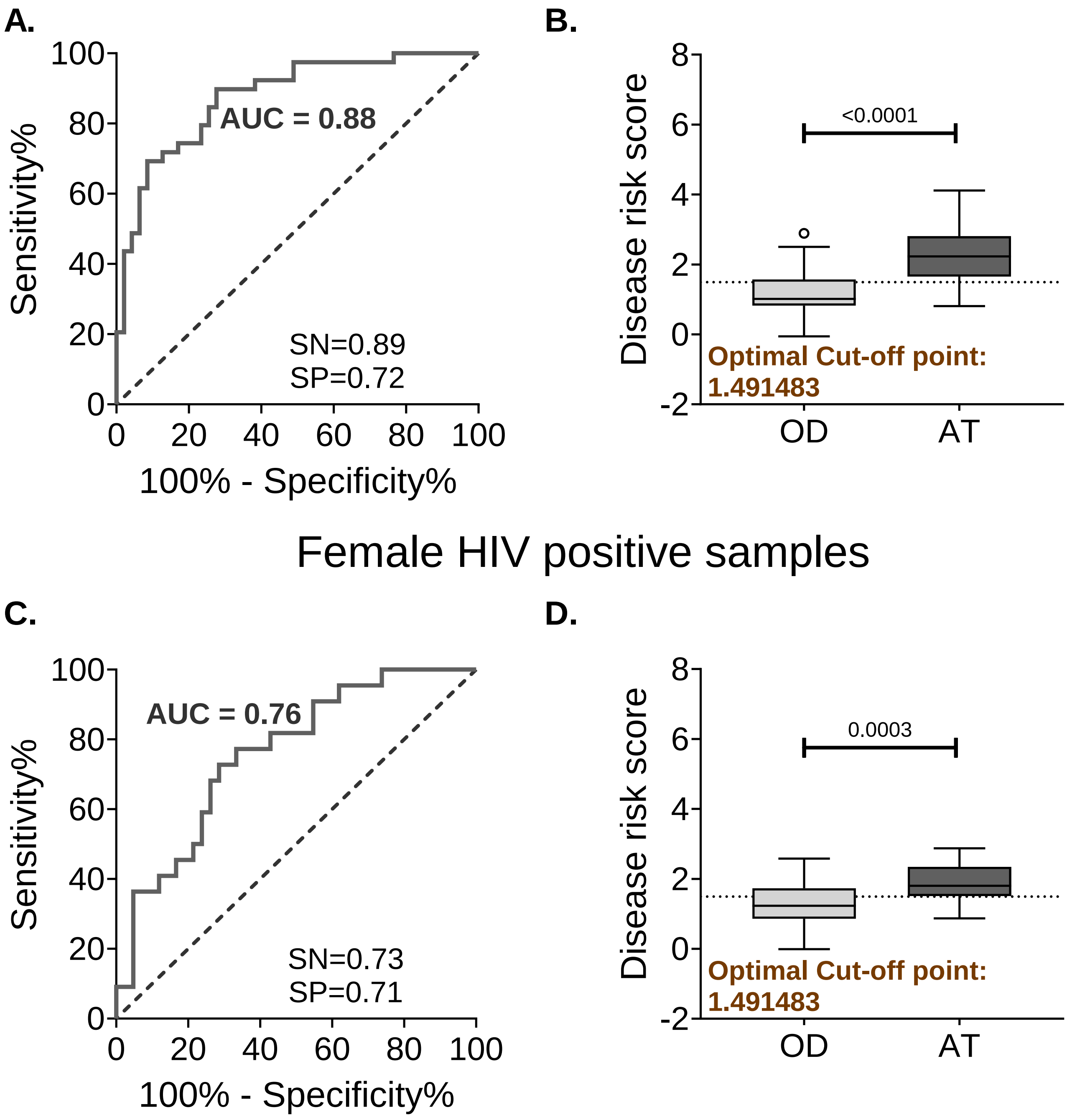

**Figure S6** Testing female gene signature in male samples

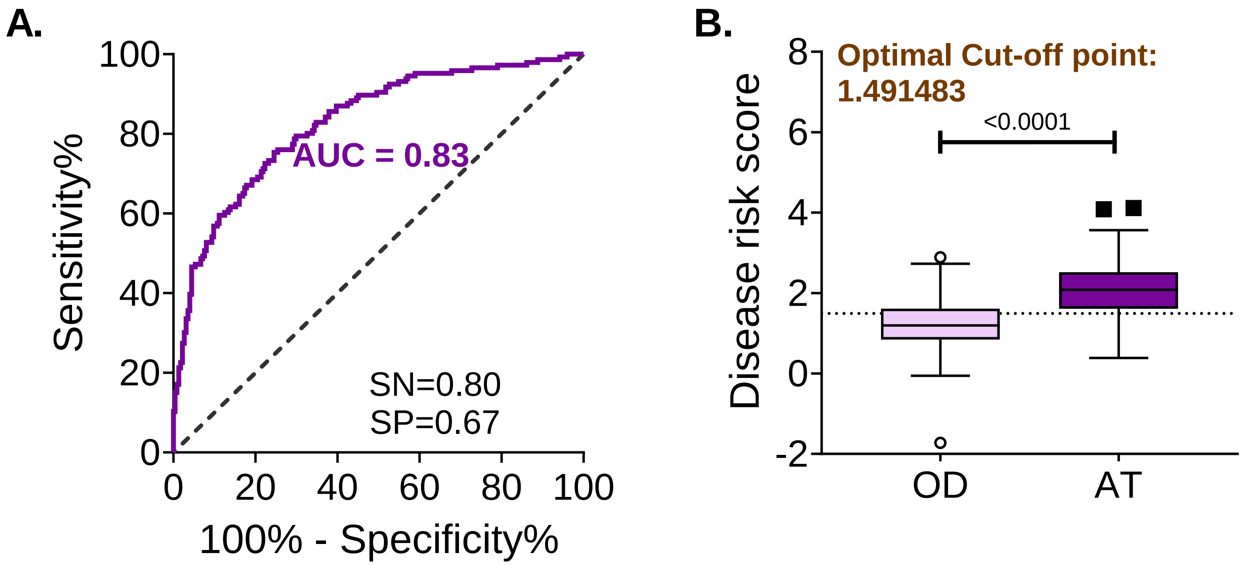


Testing male gene signature in female samples

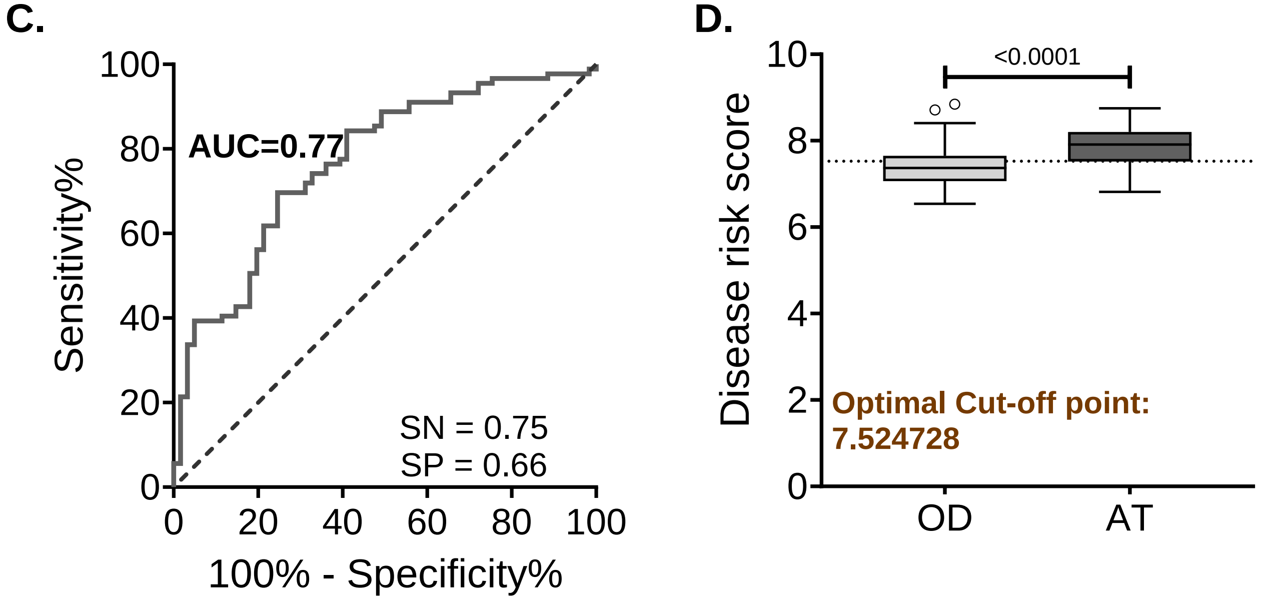

**Figure S7** Testing in culture negative male samples

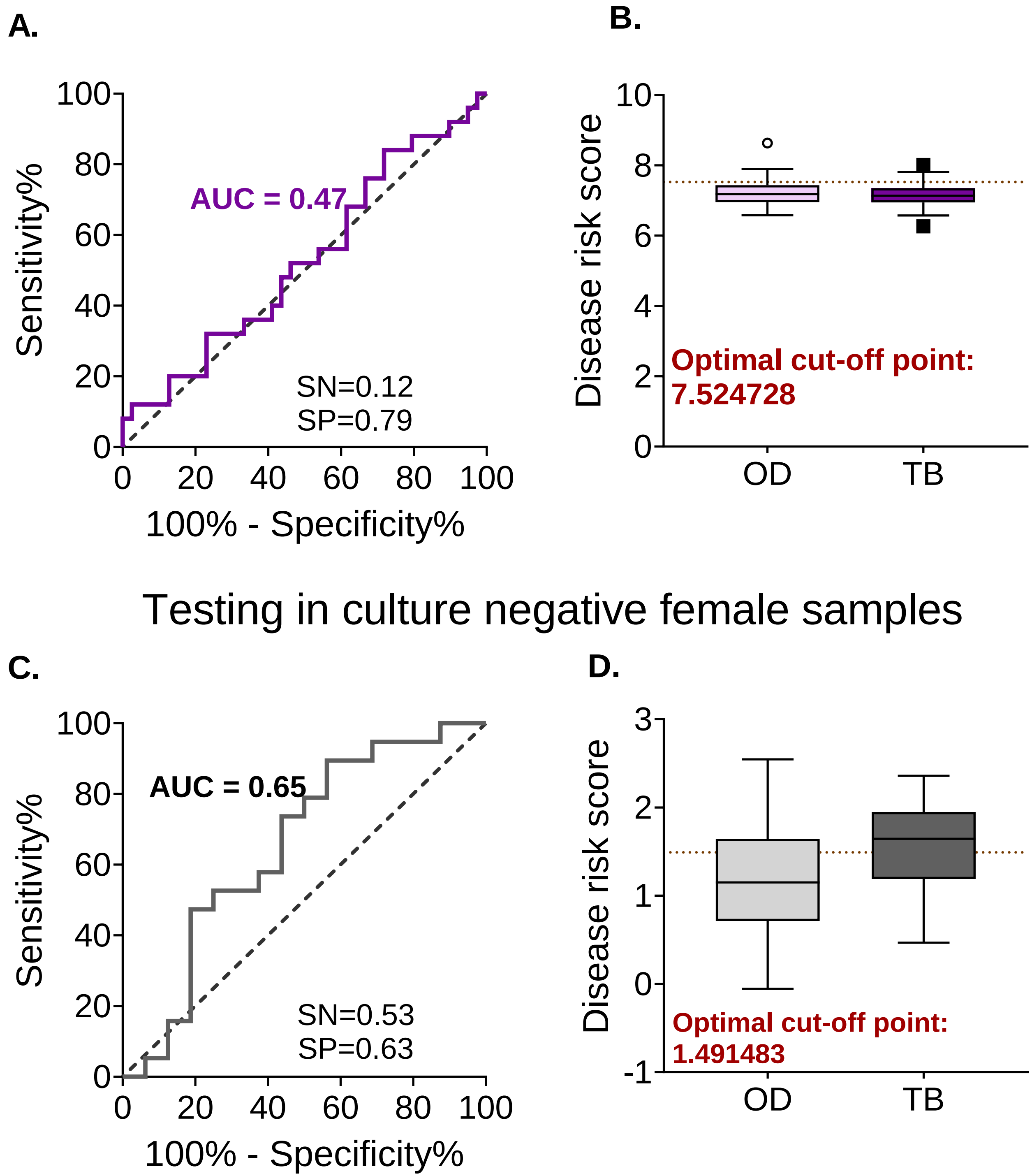

**S8**

| Common | Sex-specific | Combined |
| --- | --- | --- |
| [Males (10), Females (9)] | [Males (74), Females (14)] | [Males (84), Females (24)] |

↓

**Feature selection**
(Boruta algorithm, 100 times repeat, *p* value < 0.01)

↓

**Select confirmed features with 100% frequency**
[Common - Males (10), Female (9);
Sex-specific - Males (41), Females (11);
Combined - Males (45), Females (15)]

↓

**Random forest ranking based on Gini score**

↓

**Feature selection based on elbow separation**

↓

**Risk score construction**
(avg of upregulated genes) - (avg of downregulated genes)

↓

**Cut-off estimation**
(Youden index)

↓

**Evaluation**
(ROC, sensitivity, specificity)

**Table S1. Sample distribution**

| Characteristics | Kenya | | South Africa | | Malawi | | Total | |
|---|---|---|---|---|---|---|---|---|
| | Males | Females | Males | Females | Males | Females | Males | Females |
| **Active tuberculosis (Culture confirmed)** | 19 | 16 | 46 | 27 | 20 | 18 | 85 | 61 |
| **Active tuberculosis (Culture negative)** | 25 | 19 | | | | | | |
| **Other diseases** | 39 | 16 | 46 | 34 | 50 | 39 | 135 | 89 |
| *Lower respiratory tract infection (LRTI)* | 24 | 9 | | | | | 24 | 9 |
| *Malnutrition* | 15 | 6 | | | | | 15 | 6 |
| *Gastroenteritis* | | | 5 | 2 | | | 5 | 2 |
| *Pneumonia* | | | 30 | 27 | 17 | 14 | 47 | 41 |
| *Upper respiratory tract infection (URTI)* | | | 7 | 3 | | | 7 | 3 |
| *Abscess* | | | | | 3 | | 3 | |
| *Lymphadenitis* | | | | | 7 | 12 | 7 | 12 |
| *Others* | | 1 | 4 | 2 | 23 | 13 | 27 | 16 |
| **HIV positive** | | | | | | | | |
| *Active tuberculosis* | 6 | 4 | 14 | 9 | 9 | 9 | 29 | 22 |
| *Other diseases* | 19 | 7 | 14 | 16 | 17 | 19 | 50 | 42 |
| **HIV negative** | | | | | | | | |
| *Active tuberculosis* | 13 | 12 | 32 | 18 | 11 | 9 | 56 | 39 |
| *Other diseases* | 20 | 9 | 32 | 18 | 33 | 20 | 85 | 47 |

**Supplementary Table S2a. Differentially expressed genes_Kenya**

| Gene | log FoldChange | Direction of expression | P.Value | adj.P.Val |
|---|---|---|---|---|
| LAG3 | -0.733 | Downregulated | 4.56E-05 | 1.88E-03 |
| GZMK | -0.645 | Downregulated | 1.26E-05 | 7.61E-04 |
| CD8A | -0.618 | Downregulated | 7.07E-06 | 5.12E-04 |
| MCOLN2 | -0.608 | Downregulated | 5.69E-07 | 8.92E-05 |
| LRRN3 | -0.584 | Downregulated | 2.06E-03 | 2.61E-02 |
| TARP | -0.562 | Downregulated | 2.38E-05 | 1.19E-03 |
| GNLY | -0.538 | Downregulated | 3.41E-03 | 3.68E-02 |
| CD3D | -0.537 | Downregulated | 3.30E-06 | 3.05E-04 |
| CYORF15A | -0.533 | Downregulated | 2.65E-02 | 1.49E-01 |
| ZNF683 | -0.530 | Downregulated | 6.72E-04 | 1.20E-02 |
| PYHIN1 | -0.525 | Downregulated | 1.94E-05 | 1.04E-03 |
| CD2 | -0.522 | Downregulated | 1.84E-07 | 3.84E-05 |
| IFNG | -0.519 | Downregulated | 1.02E-03 | 1.60E-02 |
| PVRIG | -0.517 | Downregulated | 1.92E-06 | 2.09E-04 |
| CD160 | -0.516 | Downregulated | 5.17E-04 | 1.02E-02 |
| RASGRP1 | -0.513 | Downregulated | 3.19E-06 | 2.98E-04 |
| CTSW | -0.502 | Downregulated | 5.02E-05 | 2.00E-03 |
| GZMM | -0.499 | Downregulated | 4.10E-06 | 3.53E-04 |
| SKAP1 | -0.497 | Downregulated | 3.78E-07 | 6.65E-05 |
| EOMES | -0.497 | Downregulated | 1.40E-04 | 4.10E-03 |
| KLRD1 | -0.497 | Downregulated | 2.86E-04 | 6.69E-03 |
| JARID1D | -0.495 | Downregulated | 4.98E-02 | 2.22E-01 |
| SH2D1A | -0.490 | Downregulated | 6.11E-06 | 4.70E-04 |
| CD27 | -0.490 | Downregulated | 1.68E-04 | 4.68E-03 |
| TOX | -0.490 | Downregulated | 8.10E-06 | 5.66E-04 |
| CD3G | -0.487 | Downregulated | 4.99E-05 | 2.00E-03 |
| RPL14 | -0.486 | Downregulated | 7.12E-03 | 6.09E-02 |
| C6ORF190 | -0.482 | Downregulated | 6.10E-05 | 2.29E-03 |
| PTPRCAP | -0.479 | Downregulated | 7.71E-07 | 1.09E-04 |
| ETS1 | -0.477 | Downregulated | 4.17E-06 | 3.57E-04 |
| PRKCH | -0.472 | Downregulated | 1.59E-06 | 1.83E-04 |
| GZMH | -0.471 | Downregulated | 2.96E-03 | 3.32E-02 |
| CHST12 | -0.467 | Downregulated | 8.69E-05 | 2.93E-03 |
| PDCD1 | -0.466 | Downregulated | 3.63E-05 | 1.61E-03 |
| PBK | -0.458 | Downregulated | 9.38E-04 | 1.51E-02 |
| EVL | -0.458 | Downregulated | 3.83E-06 | 3.40E-04 |
| CLIC3 | -0.457 | Downregulated | 1.08E-03 | 1.65E-02 |
| MAP4K1 | -0.457 | Downregulated | 3.37E-06 | 3.10E-04 |
| KIAA0101 | -0.450 | Downregulated | 5.73E-03 | 5.24E-02 |
| TGFBR3 | -0.449 | Downregulated | 6.38E-04 | 1.16E-02 |
| KLHL3 | -0.449 | Downregulated | 1.68E-04 | 4.68E-03 |
| SAMD3 | -0.448 | Downregulated | 2.34E-05 | 1.17E-03 |
| LIME1 | -0.447 | Downregulated | 4.48E-06 | 3.77E-04 |
| CLIP3 | -0.444 | Downregulated | 2.39E-05 | 1.20E-03 |
| CD247 | -0.442 | Downregulated | 4.41E-05 | 1.86E-03 |
| RPS23 | -0.441 | Downregulated | 1.48E-02 | 1.01E-01 |
| GPR56 | -0.439 | Downregulated | 4.55E-03 | 4.47E-02 |

| | | | | |
|---|---|---|---|---|
| FGFBP2 | -0.436 | Downregulated | 7.66E-03 | 6.40E-02 |
| TC2N | -0.436 | Downregulated | 1.22E-04 | 3.71E-03 |
| CD6 | -0.436 | Downregulated | 8.73E-05 | 2.94E-03 |
| CCDC102A | -0.436 | Downregulated | 1.13E-04 | 3.52E-03 |
| MS4A1 | -0.435 | Downregulated | 1.61E-03 | 2.18E-02 |
| ZNF827 | -0.432 | Downregulated | 9.24E-06 | 6.12E-04 |
| CD3E | -0.429 | Downregulated | 8.85E-06 | 6.00E-04 |
| OCIAD2 | -0.427 | Downregulated | 1.71E-05 | 9.55E-04 |
| FANCI | -0.426 | Downregulated | 2.85E-04 | 6.67E-03 |
| TIMD4 | -0.425 | Downregulated | 9.79E-03 | 7.58E-02 |
| SLAMF6 | -0.424 | Downregulated | 3.12E-06 | 2.94E-04 |
| CXCR3 | -0.423 | Downregulated | 4.57E-07 | 7.70E-05 |
| HRK | -0.422 | Downregulated | 1.04E-02 | 7.94E-02 |
| ZNF831 | -0.422 | Downregulated | 7.52E-06 | 5.33E-04 |
| IL32 | -0.420 | Downregulated | 4.78E-05 | 1.95E-03 |
| NCALD | -0.419 | Downregulated | 1.59E-04 | 4.48E-03 |
| ITK | -0.418 | Downregulated | 1.86E-05 | 1.01E-03 |
| KIAA1324L | -0.416 | Downregulated | 1.83E-05 | 1.00E-03 |
| SIRPG | -0.415 | Downregulated | 9.77E-04 | 1.55E-02 |
| ENOSF1 | -0.415 | Downregulated | 1.80E-05 | 9.87E-04 |
| PRKY | -0.411 | Downregulated | 1.30E-02 | 9.23E-02 |
| TYMS | -0.410 | Downregulated | 1.04E-03 | 1.61E-02 |
| CLECL1 | -0.408 | Downregulated | 1.64E-02 | 1.08E-01 |
| CEP78 | -0.408 | Downregulated | 1.76E-04 | 4.84E-03 |
| C16ORF30 | -0.407 | Downregulated | 2.49E-03 | 2.96E-02 |
| CCL4L1 | -0.406 | Downregulated | 8.74E-03 | 6.99E-02 |
| GPR68 | -0.404 | Downregulated | 1.58E-03 | 2.15E-02 |
| S1PR5 | -0.403 | Downregulated | 8.49E-03 | 6.85E-02 |
| LDHB | -0.403 | Downregulated | 6.78E-05 | 2.46E-03 |
| CCND2 | -0.399 | Downregulated | 6.41E-07 | 9.72E-05 |
| PLEKHF1 | -0.399 | Downregulated | 9.75E-04 | 1.55E-02 |
| CD96 | -0.398 | Downregulated | 7.98E-05 | 2.76E-03 |
| TOP2A | -0.397 | Downregulated | 1.03E-02 | 7.86E-02 |
| TMEM14A | -0.397 | Downregulated | 2.13E-05 | 1.11E-03 |
| NELL2 | -0.396 | Downregulated | 3.46E-03 | 3.71E-02 |
| CXCR7 | -0.396 | Downregulated | 3.74E-05 | 1.65E-03 |
| GZMA | -0.396 | Downregulated | 2.95E-03 | 3.31E-02 |
| CCDC99 | -0.396 | Downregulated | 4.41E-04 | 9.09E-03 |
| BUB1 | -0.395 | Downregulated | 1.08E-02 | 8.18E-02 |
| STK39 | -0.395 | Downregulated | 1.96E-06 | 2.13E-04 |
| C12ORF23 | -0.393 | Downregulated | 1.39E-04 | 4.08E-03 |
| NOC3L | -0.392 | Downregulated | 8.13E-04 | 1.37E-02 |
| OSBPL10 | -0.391 | Downregulated | 1.53E-02 | 1.03E-01 |
| USP18 | -0.390 | Downregulated | 3.75E-02 | 1.86E-01 |
| LAX1 | -0.389 | Downregulated | 9.58E-05 | 3.10E-03 |
| PRF1 | -0.389 | Downregulated | 6.25E-03 | 5.54E-02 |
| PPP3CC | -0.388 | Downregulated | 6.59E-07 | 9.81E-05 |
| LIMA1 | -0.388 | Downregulated | 6.61E-05 | 2.42E-03 |
| IL2RB | -0.385 | Downregulated | 1.12E-03 | 1.69E-02 |
| SPOCK2 | -0.385 | Downregulated | 7.15E-04 | 1.25E-02 |
| ADA | -0.384 | Downregulated | 9.45E-05 | 3.07E-03 |

| | | | | |
|---|---|---|---|---|
| TRIB2 | -0.384 | Downregulated | 3.60E-04 | 7.81E-03 |
| CBLB | -0.383 | Downregulated | 3.73E-06 | 3.35E-04 |
| IL28RA | -0.383 | Downregulated | 4.88E-04 | 9.77E-03 |
| PRSS23 | -0.383 | Downregulated | 2.60E-03 | 3.06E-02 |
| DTX3 | -0.383 | Downregulated | 1.25E-05 | 7.61E-04 |
| PRKCQ | -0.381 | Downregulated | 5.23E-06 | 4.18E-04 |
| FAIM3 | -0.381 | Downregulated | 9.28E-04 | 1.50E-02 |
| ITGB7 | -0.376 | Downregulated | 3.15E-05 | 1.47E-03 |
| CD7 | -0.375 | Downregulated | 3.04E-04 | 6.97E-03 |
| CDT1 | -0.375 | Downregulated | 5.79E-03 | 5.28E-02 |
| GPR18 | -0.374 | Downregulated | 1.57E-04 | 4.46E-03 |
| CCDC34 | -0.372 | Downregulated | 8.99E-04 | 1.46E-02 |
| ITM2A | -0.371 | Downregulated | 2.19E-05 | 1.13E-03 |
| LY9 | -0.371 | Downregulated | 2.56E-05 | 1.27E-03 |
| RPS28 | -0.371 | Downregulated | 6.31E-04 | 1.15E-02 |
| SP4 | -0.370 | Downregulated | 2.96E-06 | 2.82E-04 |
| DENND2D | -0.370 | Downregulated | 3.80E-06 | 3.39E-04 |
| CYORF15B | -0.370 | Downregulated | 9.81E-03 | 7.59E-02 |
| CD5 | -0.369 | Downregulated | 2.22E-03 | 2.75E-02 |
| CRYZ | -0.369 | Downregulated | 1.49E-04 | 4.29E-03 |
| GPR114 | -0.368 | Downregulated | 1.77E-03 | 2.33E-02 |
| NMUR1 | -0.366 | Downregulated | 3.82E-03 | 3.98E-02 |
| RRAS2 | -0.366 | Downregulated | 4.69E-05 | 1.93E-03 |
| ZNF573 | -0.366 | Downregulated | 1.21E-02 | 8.80E-02 |
| CLEC2D | -0.363 | Downregulated | 7.51E-07 | 1.08E-04 |
| KLRA1 | -0.363 | Downregulated | 6.73E-04 | 1.20E-02 |
| CCL5 | -0.362 | Downregulated | 1.57E-03 | 2.14E-02 |
| CDC45L | -0.362 | Downregulated | 1.67E-02 | 1.10E-01 |
| MSC | -0.362 | Downregulated | 4.26E-04 | 8.87E-03 |
| RPL23 | -0.359 | Downregulated | 4.26E-02 | 2.01E-01 |
| C20ORF100 | -0.359 | Downregulated | 3.06E-05 | 1.44E-03 |
| EBI2 | -0.358 | Downregulated | 4.01E-03 | 4.12E-02 |
| FAM113B | -0.358 | Downregulated | 9.28E-05 | 3.03E-03 |
| RNASEH2B | -0.358 | Downregulated | 2.29E-03 | 2.79E-02 |
| GFI1 | -0.357 | Downregulated | 4.28E-04 | 8.88E-03 |
| NCAPG | -0.356 | Downregulated | 2.36E-02 | 1.38E-01 |
| FAM102A | -0.356 | Downregulated | 3.17E-03 | 3.50E-02 |
| ITPR3 | -0.355 | Downregulated | 7.08E-06 | 5.12E-04 |
| BIN1 | -0.355 | Downregulated | 2.91E-05 | 1.39E-03 |
| KIFC1 | -0.354 | Downregulated | 9.66E-03 | 7.51E-02 |
| TMEM204 | -0.354 | Downregulated | 3.17E-03 | 3.50E-02 |
| CRIP1 | -0.354 | Downregulated | 2.25E-04 | 5.72E-03 |
| PARP15 | -0.353 | Downregulated | 1.28E-04 | 3.84E-03 |
| MEX3C | -0.352 | Downregulated | 3.12E-04 | 7.09E-03 |
| ASPM | -0.352 | Downregulated | 9.36E-03 | 7.36E-02 |
| RPS4Y2 | -0.352 | Downregulated | 3.82E-02 | 1.88E-01 |
| FCRL3 | -0.351 | Downregulated | 1.12E-03 | 1.69E-02 |
| FLJ33590 | -0.351 | Downregulated | 2.46E-04 | 6.08E-03 |
| SLC25A23 | -0.351 | Downregulated | 9.49E-05 | 3.08E-03 |
| C10ORF33 | -0.349 | Downregulated | 1.93E-03 | 2.48E-02 |
| NMT2 | -0.348 | Downregulated | 2.64E-04 | 6.35E-03 |

| | | | | |
|---|---|---|---|---|
| FAM83D | -0.348 | Downregulated | 8.96E-05 | 2.98E-03 |
| MCM4 | -0.348 | Downregulated | 3.05E-03 | 3.41E-02 |
| ICOS | -0.347 | Downregulated | 3.90E-03 | 4.05E-02 |
| PACSIN1 | -0.346 | Downregulated | 2.54E-04 | 6.20E-03 |
| QPRT | -0.346 | Downregulated | 8.40E-04 | 1.40E-02 |
| ESYT1 | -0.345 | Downregulated | 8.76E-05 | 2.94E-03 |
| CDKN1C | -0.345 | Downregulated | 1.86E-02 | 1.18E-01 |
| WDR54 | -0.345 | Downregulated | 1.31E-05 | 7.83E-04 |
| KLF12 | -0.345 | Downregulated | 1.35E-04 | 3.98E-03 |
| HNRNPH1 | -0.344 | Downregulated | 2.04E-05 | 1.08E-03 |
| MCM2 | -0.344 | Downregulated | 4.23E-03 | 4.26E-02 |
| RFC4 | -0.344 | Downregulated | 1.19E-04 | 3.65E-03 |
| PMS1 | -0.344 | Downregulated | 2.39E-03 | 2.88E-02 |
| SNRNP70 | -0.343 | Downregulated | 9.13E-03 | 7.21E-02 |
| PCNT | -0.343 | Downregulated | 7.47E-06 | 5.32E-04 |
| UAP1 | -0.341 | Downregulated | 2.77E-03 | 3.19E-02 |
| CTLA4 | -0.341 | Downregulated | 1.86E-04 | 5.07E-03 |
| TSEN54 | -0.340 | Downregulated | 6.23E-04 | 1.14E-02 |
| GLOD4 | -0.339 | Downregulated | 7.85E-08 | 2.05E-05 |
| SLC39A10 | -0.339 | Downregulated | 4.45E-04 | 9.16E-03 |
| JAKMIP2 | -0.339 | Downregulated | 2.86E-04 | 6.69E-03 |
| GZMB | -0.338 | Downregulated | 2.96E-02 | 1.60E-01 |
| FAM84B | -0.338 | Downregulated | 2.17E-03 | 2.70E-02 |
| MRPL40 | -0.337 | Downregulated | 1.81E-02 | 1.16E-01 |
| CELSR3 | -0.337 | Downregulated | 1.53E-03 | 2.11E-02 |
| NKG7 | -0.337 | Downregulated | 5.47E-03 | 5.08E-02 |
| MRPL1 | -0.336 | Downregulated | 4.44E-03 | 4.42E-02 |
| S1PR1 | -0.336 | Downregulated | 6.91E-04 | 1.23E-02 |
| FASLG | -0.335 | Downregulated | 1.61E-02 | 1.07E-01 |
| TNFRSF13B | -0.334 | Downregulated | 3.12E-02 | 1.65E-01 |
| STMN3 | -0.334 | Downregulated | 1.55E-03 | 2.12E-02 |
| MCM10 | -0.334 | Downregulated | 1.67E-03 | 2.24E-02 |
| NOP58 | -0.333 | Downregulated | 4.81E-05 | 1.96E-03 |
| ALS2CR4 | -0.333 | Downregulated | 1.86E-04 | 5.07E-03 |
| CCNB2 | -0.332 | Downregulated | 3.04E-02 | 1.62E-01 |
| GMNN | -0.332 | Downregulated | 1.14E-02 | 8.46E-02 |
| STIL | -0.332 | Downregulated | 1.50E-03 | 2.08E-02 |
| C11ORF80 | -0.332 | Downregulated | 5.46E-03 | 5.07E-02 |
| PTPN4 | -0.332 | Downregulated | 3.32E-04 | 7.43E-03 |
| PTGDR | -0.332 | Downregulated | 3.71E-03 | 3.89E-02 |
| CD8B | -0.329 | Downregulated | 1.04E-06 | 1.35E-04 |
| STAT4 | -0.329 | Downregulated | 9.82E-05 | 3.16E-03 |
| HMMR | -0.328 | Downregulated | 1.71E-02 | 1.11E-01 |
| MXD4 | -0.326 | Downregulated | 4.33E-05 | 1.84E-03 |
| MCM6 | -0.325 | Downregulated | 8.70E-05 | 2.93E-03 |
| KIFAP3 | -0.325 | Downregulated | 1.05E-03 | 1.62E-02 |
| KLRC1 | -0.324 | Downregulated | 2.25E-03 | 2.76E-02 |
| SLAMF1 | -0.324 | Downregulated | 1.52E-03 | 2.10E-02 |
| ZNF256 | -0.324 | Downregulated | 6.87E-04 | 1.22E-02 |
| MDC1 | -0.323 | Downregulated | 7.93E-07 | 1.11E-04 |
| RFTN1 | -0.323 | Downregulated | 2.77E-05 | 1.35E-03 |

| | | | | |
|---|---|---|---|---|
| G3BP1 | -0.323 | Downregulated | 4.27E-04 | 8.87E-03 |
| PFAS | -0.322 | Downregulated | 2.14E-04 | 5.56E-03 |
| CACNA2D3 | -0.322 | Downregulated | 1.07E-02 | 8.08E-02 |
| CLDND2 | -0.322 | Downregulated | 4.23E-03 | 4.26E-02 |
| TIPIN | -0.322 | Downregulated | 4.03E-04 | 8.50E-03 |
| PEBP1 | -0.321 | Downregulated | 9.33E-06 | 6.17E-04 |
| CDCA5 | -0.321 | Downregulated | 1.75E-02 | 1.13E-01 |
| CHI3L2 | -0.321 | Downregulated | 1.45E-02 | 9.98E-02 |
| GPATCH4 | -0.321 | Downregulated | 2.24E-03 | 2.75E-02 |
| PPP1R16B | -0.320 | Downregulated | 8.49E-05 | 2.89E-03 |
| TARBP1 | -0.319 | Downregulated | 7.90E-04 | 1.34E-02 |
| RRM1 | -0.319 | Downregulated | 6.02E-05 | 2.27E-03 |
| CDCA4 | -0.318 | Downregulated | 5.13E-05 | 2.03E-03 |
| SLC27A2 | -0.318 | Downregulated | 1.06E-03 | 1.63E-02 |
| PLCG1 | -0.318 | Downregulated | 2.66E-05 | 1.31E-03 |
| KIF20B | -0.317 | Downregulated | 1.92E-03 | 2.47E-02 |
| LYAR | -0.317 | Downregulated | 5.04E-06 | 4.07E-04 |
| BRIX1 | -0.317 | Downregulated | 5.59E-05 | 2.15E-03 |
| PLEKHA1 | -0.317 | Downregulated | 2.13E-04 | 5.54E-03 |
| EPHA4 | -0.317 | Downregulated | 7.93E-04 | 1.35E-02 |
| DLGAP5 | -0.316 | Downregulated | 2.39E-02 | 1.39E-01 |
| TTK | -0.316 | Downregulated | 5.95E-03 | 5.37E-02 |
| CDKN3 | -0.316 | Downregulated | 4.11E-02 | 1.97E-01 |
| TECR | -0.315 | Downregulated | 5.90E-06 | 4.62E-04 |
| C6ORF173 | -0.315 | Downregulated | 1.15E-02 | 8.49E-02 |
| MLLT11 | -0.314 | Downregulated | 2.90E-05 | 1.39E-03 |
| Septin 11 | -0.313 | Downregulated | 7.01E-04 | 1.24E-02 |
| DCLRE1A | -0.313 | Downregulated | 8.01E-04 | 1.35E-02 |
| HSPE1 | -0.313 | Downregulated | 2.71E-03 | 3.14E-02 |
| KIF11 | -0.313 | Downregulated | 4.57E-03 | 4.49E-02 |
| ATP6V0E2 | -0.313 | Downregulated | 8.17E-04 | 1.37E-02 |
| ZFP82 | -0.312 | Downregulated | 1.13E-03 | 1.70E-02 |
| UPF3A | -0.312 | Downregulated | 6.81E-07 | 1.01E-04 |
| RPL10A | -0.312 | Downregulated | 1.87E-04 | 5.08E-03 |
| CCNF | -0.311 | Downregulated | 3.40E-04 | 7.55E-03 |
| CLYBL | -0.311 | Downregulated | 1.56E-03 | 2.13E-02 |
| ATIC | -0.311 | Downregulated | 1.16E-05 | 7.12E-04 |
| FAM167A | -0.310 | Downregulated | 3.87E-03 | 4.02E-02 |
| BIVM | -0.310 | Downregulated | 2.11E-04 | 5.52E-03 |
| FAM179B | -0.310 | Downregulated | 1.22E-02 | 8.87E-02 |
| AGMAT | -0.310 | Downregulated | 4.49E-03 | 4.44E-02 |
| MGC3020 | -0.310 | Downregulated | 4.19E-04 | 8.77E-03 |
| ID3 | -0.309 | Downregulated | 1.94E-02 | 1.21E-01 |
| PARM1 | -0.309 | Downregulated | 2.93E-02 | 1.59E-01 |
| RPN2 | -0.309 | Downregulated | 4.95E-03 | 4.74E-02 |
| ARMC5 | -0.309 | Downregulated | 1.60E-05 | 9.04E-04 |
| SIT1 | -0.308 | Downregulated | 1.04E-04 | 3.30E-03 |
| DTL | -0.308 | Downregulated | 2.63E-03 | 3.09E-02 |
| SMC6 | -0.308 | Downregulated | 7.78E-03 | 6.46E-02 |
| ECHDC2 | -0.307 | Downregulated | 3.82E-05 | 1.67E-03 |
| MRPL48 | -0.306 | Downregulated | 1.37E-03 | 1.94E-02 |

| | | | | |
|---|---|---|---|---|
| SCML4 | -0.306 | Downregulated | 3.16E-04 | 7.16E-03 |
| TBC1D4 | -0.305 | Downregulated | 5.01E-03 | 4.78E-02 |
| MYO18B | -0.305 | Downregulated | 3.75E-02 | 1.86E-01 |
| RFX7 | -0.305 | Downregulated | 4.48E-05 | 1.87E-03 |
| TNFRSF25 | -0.305 | Downregulated | 2.63E-03 | 3.08E-02 |
| PARP1 | -0.304 | Downregulated | 2.17E-04 | 5.61E-03 |
| AKR1B1 | -0.304 | Downregulated | 4.87E-05 | 1.98E-03 |
| SLC38A1 | -0.303 | Downregulated | 1.11E-06 | 1.41E-04 |
| C14ORF145 | -0.303 | Downregulated | 1.32E-03 | 1.90E-02 |
| CARD11 | -0.303 | Downregulated | 2.28E-05 | 1.16E-03 |
| CD70 | -0.303 | Downregulated | 1.64E-03 | 2.21E-02 |
| NCAPG2 | -0.303 | Downregulated | 3.27E-03 | 3.58E-02 |
| NAT6 | -0.302 | Downregulated | 2.27E-04 | 5.75E-03 |
| STAMBPL1 | -0.302 | Downregulated | 3.77E-04 | 8.11E-03 |
| DNAJC9 | -0.302 | Downregulated | 7.46E-05 | 2.63E-03 |
| KIF4A | -0.301 | Downregulated | 1.47E-02 | 1.01E-01 |
| PDXP | -0.301 | Downregulated | 6.30E-05 | 2.35E-03 |
| FAM179A | -0.301 | Downregulated | 1.11E-02 | 8.29E-02 |
| CEP290 | -0.300 | Downregulated | 9.95E-04 | 1.57E-02 |
| GRAMD3 | -0.300 | Downregulated | 4.55E-05 | 1.88E-03 |
| GINS2 | -0.300 | Downregulated | 2.54E-02 | 1.45E-01 |
| TSEN15 | -0.300 | Downregulated | 1.07E-04 | 3.37E-03 |
| LRFN3 | -0.299 | Downregulated | 1.67E-04 | 4.65E-03 |
| AURKB | -0.299 | Downregulated | 3.56E-02 | 1.80E-01 |
| IRF4 | -0.299 | Downregulated | 1.41E-03 | 1.99E-02 |
| PASK | -0.299 | Downregulated | 4.59E-03 | 4.50E-02 |
| CDC7 | -0.299 | Downregulated | 4.46E-04 | 9.16E-03 |
| INPP4B | -0.298 | Downregulated | 3.18E-05 | 1.47E-03 |
| RBBP7 | -0.298 | Downregulated | 1.41E-05 | 8.26E-04 |
| MBNL2 | -0.297 | Downregulated | 4.96E-03 | 4.75E-02 |
| BCAR3 | -0.297 | Downregulated | 1.66E-02 | 1.09E-01 |
| CD248 | -0.297 | Downregulated | 1.15E-02 | 8.52E-02 |
| RPL5 | -0.297 | Downregulated | 2.11E-03 | 2.65E-02 |
| WEE1 | -0.296 | Downregulated | 3.13E-04 | 7.10E-03 |
| IBTK | -0.296 | Downregulated | 2.49E-03 | 2.97E-02 |
| THOC4 | -0.296 | Downregulated | 9.30E-04 | 1.50E-02 |
| CD19 | -0.295 | Downregulated | 4.11E-02 | 1.97E-01 |
| MAK16 | -0.295 | Downregulated | 1.47E-03 | 2.04E-02 |
| SLFN13 | -0.294 | Downregulated | 5.63E-04 | 1.07E-02 |
| SLC16A10 | -0.294 | Downregulated | 3.59E-02 | 1.81E-01 |
| CYFIP2 | -0.294 | Downregulated | 3.85E-06 | 3.41E-04 |
| XPO4 | -0.294 | Downregulated | 1.49E-05 | 8.52E-04 |
| HDDC2 | -0.294 | Downregulated | 3.10E-04 | 7.05E-03 |
| CHAC2 | -0.294 | Downregulated | 3.06E-03 | 3.41E-02 |
| ZNF529 | -0.293 | Downregulated | 7.09E-06 | 5.12E-04 |
| UPLP | -0.293 | Downregulated | 1.91E-04 | 5.15E-03 |
| KIF22 | -0.293 | Downregulated | 2.32E-07 | 4.58E-05 |
| CTPS | -0.293 | Downregulated | 1.41E-03 | 1.99E-02 |
| LPIN1 | -0.293 | Downregulated | 8.13E-05 | 2.80E-03 |
| AURKA | -0.292 | Downregulated | 1.67E-02 | 1.10E-01 |
| KIAA1430 | -0.292 | Downregulated | 3.92E-04 | 8.33E-03 |

| | | | | |
|---|---|---|---|---|
| DNMT1 | -0.292 | Downregulated | 1.85E-04 | 5.05E-03 |
| P2RY11 | -0.291 | Downregulated | 8.54E-04 | 1.41E-02 |
| MEF2D | -0.291 | Downregulated | 1.18E-06 | 1.46E-04 |
| RPUSD2 | -0.291 | Downregulated | 1.68E-04 | 4.68E-03 |
| PHGDH | -0.291 | Downregulated | 8.15E-03 | 6.66E-02 |
| C8ORF13 | -0.290 | Downregulated | 1.37E-02 | 9.55E-02 |
| C1ORF21 | -0.290 | Downregulated | 1.25E-02 | 8.99E-02 |
| FBXO31 | -0.290 | Downregulated | 2.85E-05 | 1.38E-03 |
| MIF | -0.290 | Downregulated | 6.67E-05 | 2.43E-03 |
| BYSL | -0.290 | Downregulated | 1.77E-03 | 2.32E-02 |
| KDELC2 | -0.289 | Downregulated | 2.16E-03 | 2.69E-02 |
| HRASLS2 | -0.289 | Downregulated | 1.53E-02 | 1.03E-01 |
| MEI1 | -0.289 | Downregulated | 4.97E-04 | 9.87E-03 |
| PAICS | -0.289 | Downregulated | 9.42E-04 | 1.51E-02 |
| HSD17B8 | -0.288 | Downregulated | 2.17E-04 | 5.61E-03 |
| RAB40B | -0.288 | Downregulated | 3.33E-05 | 1.51E-03 |
| CD79B | -0.288 | Downregulated | 2.32E-02 | 1.36E-01 |
| NLRC3 | -0.288 | Downregulated | 9.53E-05 | 3.09E-03 |
| SMARCAD1 | -0.288 | Downregulated | 1.42E-03 | 1.99E-02 |
| CHAF1B | -0.288 | Downregulated | 7.22E-03 | 6.15E-02 |
| SIDT1 | -0.287 | Downregulated | 9.25E-04 | 1.50E-02 |
| ZFP42 | -0.287 | Downregulated | 9.49E-04 | 1.52E-02 |
| SPIB | -0.287 | Downregulated | 3.45E-02 | 1.76E-01 |
| SEL1L3 | -0.287 | Downregulated | 7.39E-04 | 1.28E-02 |
| URG4 | -0.286 | Downregulated | 2.47E-07 | 4.76E-05 |
| TBC1D19 | -0.285 | Downregulated | 4.48E-05 | 1.87E-03 |
| WDR34 | -0.285 | Downregulated | 9.55E-04 | 1.53E-02 |
| SNRPF | -0.285 | Downregulated | 4.39E-05 | 1.85E-03 |
| ENPP5 | -0.285 | Downregulated | 4.63E-03 | 4.52E-02 |
| POLE2 | -0.285 | Downregulated | 3.85E-03 | 4.00E-02 |
| NUSAP1 | -0.285 | Downregulated | 3.55E-03 | 3.78E-02 |
| CENPA | -0.285 | Downregulated | 1.15E-02 | 8.48E-02 |
| CXCR6 | -0.285 | Downregulated | 3.32E-02 | 1.72E-01 |
| DENND5B | -0.285 | Downregulated | 3.74E-02 | 1.86E-01 |
| ARL4C | -0.284 | Downregulated | 5.87E-05 | 2.23E-03 |
| LPXN | -0.284 | Downregulated | 2.57E-06 | 2.58E-04 |
| NUDCD2 | -0.284 | Downregulated | 6.45E-04 | 1.17E-02 |
| ELOVL6 | -0.284 | Downregulated | 1.51E-03 | 2.09E-02 |
| TUBB | -0.284 | Downregulated | 5.78E-05 | 2.21E-03 |
| SAE1 | -0.283 | Downregulated | 8.35E-06 | 5.76E-04 |
| GIMAP1 | -0.283 | Downregulated | 3.72E-03 | 3.89E-02 |
| NDC80 | -0.283 | Downregulated | 4.89E-03 | 4.70E-02 |
| CCNA2 | -0.283 | Downregulated | 2.43E-02 | 1.41E-01 |
| MYBL1 | -0.283 | Downregulated | 2.27E-02 | 1.34E-01 |
| WBSCR22 | -0.283 | Downregulated | 6.56E-05 | 2.41E-03 |
| NDUFB9 | -0.282 | Downregulated | 1.53E-04 | 4.36E-03 |
| RANBP1 | -0.282 | Downregulated | 5.96E-05 | 2.25E-03 |
| ABCE1 | -0.282 | Downregulated | 5.63E-04 | 1.07E-02 |
| TMEM118 | -0.281 | Downregulated | 3.57E-03 | 3.80E-02 |
| VIL2 | -0.281 | Downregulated | 1.58E-04 | 4.47E-03 |
| EBP | -0.281 | Downregulated | 9.09E-05 | 3.01E-03 |

| | | | | |
|---|---|---|---|---|
| MPI | -0.281 | Downregulated | 2.91E-05 | 1.39E-03 |
| LCK | -0.281 | Downregulated | 6.55E-06 | 4.88E-04 |
| PCID2 | -0.281 | Downregulated | 1.01E-05 | 6.43E-04 |
| FAM98A | -0.281 | Downregulated | 1.57E-03 | 2.15E-02 |
| MGC26718 | -0.281 | Downregulated | 1.51E-03 | 2.08E-02 |
| MRPL38 | -0.280 | Downregulated | 1.28E-05 | 7.68E-04 |
| NOP56 | -0.280 | Downregulated | 1.48E-05 | 8.48E-04 |
| C6ORF129 | -0.280 | Downregulated | 2.48E-03 | 2.95E-02 |
| FAM162A | -0.280 | Downregulated | 1.07E-03 | 1.65E-02 |
| CCDC14 | -0.280 | Downregulated | 5.59E-04 | 1.06E-02 |
| RAD51AP1 | -0.279 | Downregulated | 1.01E-02 | 7.76E-02 |
| NUCKS1 | -0.279 | Downregulated | 2.48E-05 | 1.24E-03 |
| PRC1 | -0.279 | Downregulated | 6.05E-03 | 5.42E-02 |
| SLC4A7 | -0.279 | Downregulated | 2.44E-04 | 6.06E-03 |
| MND1 | -0.278 | Downregulated | 2.63E-03 | 3.09E-02 |
| SNX25 | -0.278 | Downregulated | 9.17E-04 | 1.49E-02 |
| C12ORF57 | -0.278 | Downregulated | 6.54E-03 | 5.73E-02 |
| DBP | -0.278 | Downregulated | 3.79E-05 | 1.66E-03 |
| DYRK2 | -0.278 | Downregulated | 1.78E-04 | 4.87E-03 |
| FABP5 | -0.278 | Downregulated | 4.42E-03 | 4.40E-02 |
| ZZZ3 | -0.278 | Downregulated | 3.40E-04 | 7.55E-03 |
| SPTAN1 | -0.277 | Downregulated | 1.12E-04 | 3.50E-03 |
| LANCL1 | -0.276 | Downregulated | 5.74E-05 | 2.20E-03 |
| ITGB3BP | -0.276 | Downregulated | 1.08E-02 | 8.17E-02 |
| TMEM156 | -0.275 | Downregulated | 2.43E-03 | 2.90E-02 |
| COL4A4 | -0.275 | Downregulated | 4.90E-03 | 4.71E-02 |
| CD52 | -0.275 | Downregulated | 2.40E-02 | 1.40E-01 |
| SLC41A1 | -0.275 | Downregulated | 1.40E-03 | 1.97E-02 |
| POLR2J2 | -0.275 | Downregulated | 5.69E-04 | 1.08E-02 |
| C10ORF88 | -0.275 | Downregulated | 1.12E-02 | 8.37E-02 |
| UFSP2 | -0.275 | Downregulated | 2.04E-03 | 2.58E-02 |
| NAE1 | -0.274 | Downregulated | 3.56E-03 | 3.79E-02 |
| NEK1 | -0.274 | Downregulated | 5.23E-03 | 4.93E-02 |
| MINPP1 | -0.274 | Downregulated | 2.40E-03 | 2.88E-02 |
| DLAT | -0.274 | Downregulated | 3.92E-03 | 4.06E-02 |
| CPOX | -0.274 | Downregulated | 9.15E-04 | 1.49E-02 |
| Septin 1 | -0.274 | Downregulated | 9.96E-05 | 3.19E-03 |
| CYP2J2 | -0.273 | Downregulated | 6.85E-04 | 1.22E-02 |
| AGK | -0.273 | Downregulated | 9.21E-05 | 3.02E-03 |
| FLJ20628 | -0.273 | Downregulated | 1.51E-04 | 4.33E-03 |
| FAM159A | -0.272 | Downregulated | 8.93E-03 | 7.11E-02 |
| CDCA7 | -0.272 | Downregulated | 3.23E-05 | 1.48E-03 |
| NELF | -0.272 | Downregulated | 2.06E-04 | 5.43E-03 |
| ARPC5L | -0.272 | Downregulated | 6.33E-06 | 4.82E-04 |
| ABHD14A | -0.272 | Downregulated | 6.56E-04 | 1.18E-02 |
| SPRY2 | -0.271 | Downregulated | 1.17E-03 | 1.75E-02 |
| FYN | -0.271 | Downregulated | 7.21E-05 | 2.58E-03 |
| SLC25A42 | -0.271 | Downregulated | 7.27E-06 | 5.21E-04 |
| RRN3 | -0.271 | Downregulated | 2.69E-05 | 1.32E-03 |
| SETBP1 | -0.271 | Downregulated | 4.98E-04 | 9.88E-03 |
| YES1 | -0.271 | Downregulated | 2.20E-04 | 5.66E-03 |

| | | | | |
|---|---|---|---|---|
| MNAT1 | -0.271 | Downregulated | 2.31E-03 | 2.81E-02 |
| MRPS6 | -0.270 | Downregulated | 2.79E-05 | 1.36E-03 |
| NARG1L | -0.270 | Downregulated | 2.84E-04 | 6.67E-03 |
| P2RY10 | -0.270 | Downregulated | 1.22E-03 | 1.80E-02 |
| INTS2 | -0.269 | Downregulated | 2.23E-03 | 2.75E-02 |
| TUBB4Q | -0.269 | Downregulated | 3.18E-02 | 1.67E-01 |
| ZNHIT6 | -0.269 | Downregulated | 8.13E-05 | 2.80E-03 |
| ZBED2 | -0.269 | Downregulated | 2.74E-02 | 1.52E-01 |
| CENPE | -0.269 | Downregulated | 4.88E-03 | 4.70E-02 |
| RAB33A | -0.269 | Downregulated | 8.82E-06 | 5.99E-04 |
| C1ORF19 | -0.269 | Downregulated | 7.69E-05 | 2.69E-03 |
| CHCHD6 | -0.269 | Downregulated | 9.13E-04 | 1.49E-02 |
| GPAM | -0.268 | Downregulated | 2.75E-04 | 6.52E-03 |
| ZNF181 | -0.268 | Downregulated | 2.81E-02 | 1.54E-01 |
| MIB2 | -0.268 | Downregulated | 9.34E-04 | 1.50E-02 |
| ARHGEF3 | -0.268 | Downregulated | 6.33E-04 | 1.15E-02 |
| CEP70 | -0.268 | Downregulated | 2.14E-03 | 2.67E-02 |
| C13ORF7 | -0.268 | Downregulated | 4.08E-03 | 4.16E-02 |
| ABHD15 | -0.267 | Downregulated | 1.37E-03 | 1.95E-02 |
| NUP54 | -0.267 | Downregulated | 7.63E-04 | 1.31E-02 |
| GTSF1L | -0.267 | Downregulated | 2.10E-04 | 5.50E-03 |
| IKZF3 | -0.267 | Downregulated | 4.12E-02 | 1.97E-01 |
| NOLC1 | -0.267 | Downregulated | 5.65E-03 | 5.18E-02 |
| ZNF121 | -0.267 | Downregulated | 2.28E-03 | 2.78E-02 |
| MGC72104 | -0.267 | Downregulated | 4.07E-04 | 8.57E-03 |
| CENPK | -0.267 | Downregulated | 3.10E-02 | 1.64E-01 |
| SLC25A4 | -0.267 | Downregulated | 1.32E-02 | 9.33E-02 |
| TMEM181 | -0.267 | Downregulated | 6.39E-04 | 1.16E-02 |
| CCDC58 | -0.267 | Downregulated | 2.80E-02 | 1.54E-01 |
| ZFY | -0.266 | Downregulated | 8.79E-03 | 7.02E-02 |
| Septin 7 | -0.266 | Downregulated | 3.64E-02 | 1.82E-01 |
| SPNS3 | -0.266 | Downregulated | 4.34E-03 | 4.34E-02 |
| BOLA3 | -0.266 | Downregulated | 2.14E-04 | 5.56E-03 |
| CDCA2 | -0.266 | Downregulated | 1.51E-02 | 1.03E-01 |
| MTX3 | -0.266 | Downregulated | 1.04E-03 | 1.62E-02 |
| SF3A3 | -0.265 | Downregulated | 7.12E-05 | 2.56E-03 |
| LAMA5 | -0.265 | Downregulated | 6.24E-03 | 5.54E-02 |
| CCDC107 | -0.265 | Downregulated | 4.79E-04 | 9.62E-03 |
| C1GALT1C1 | 0.265 | Upregulated | 1.19E-03 | 1.77E-02 |
| GLUL | 0.265 | Upregulated | 5.02E-03 | 4.78E-02 |
| FOSL2 | 0.265 | Upregulated | 1.32E-04 | 3.92E-03 |
| MBOAT2 | 0.266 | Upregulated | 6.40E-04 | 1.16E-02 |
| TNFAIP8L2 | 0.266 | Upregulated | 3.24E-06 | 3.02E-04 |
| CCNJL | 0.266 | Upregulated | 5.78E-03 | 5.27E-02 |
| KIAA0513 | 0.266 | Upregulated | 1.33E-03 | 1.91E-02 |
| ITGA5 | 0.266 | Upregulated | 1.68E-03 | 2.25E-02 |
| DIRC2 | 0.266 | Upregulated | 6.40E-03 | 5.65E-02 |
| LONRF3 | 0.266 | Upregulated | 3.63E-04 | 7.86E-03 |
| HIST1H3H | 0.266 | Upregulated | 2.99E-02 | 1.60E-01 |
| GAB2 | 0.266 | Upregulated | 1.63E-06 | 1.87E-04 |
| TBC1D14 | 0.267 | Upregulated | 2.85E-04 | 6.68E-03 |

| | | | | |
|---|---|---|---|---|
| ZNF213 | 0.267 | Upregulated | 1.14E-04 | 3.54E-03 |
| MX2 | 0.267 | Upregulated | 2.49E-02 | 1.43E-01 |
| TBXAS1 | 0.267 | Upregulated | 4.37E-03 | 4.36E-02 |
| FKBP5 | 0.267 | Upregulated | 9.34E-03 | 7.34E-02 |
| RGS18 | 0.267 | Upregulated | 6.50E-03 | 5.70E-02 |
| ATP6V0D1 | 0.267 | Upregulated | 1.33E-03 | 1.90E-02 |
| TMEM8 | 0.267 | Upregulated | 4.18E-04 | 8.75E-03 |
| ARHGAP27 | 0.268 | Upregulated | 5.71E-03 | 5.23E-02 |
| IL10RB | 0.268 | Upregulated | 1.81E-04 | 4.95E-03 |
| IFIH1 | 0.268 | Upregulated | 6.69E-03 | 5.83E-02 |
| C16ORF57 | 0.268 | Upregulated | 1.32E-03 | 1.90E-02 |
| HIP1 | 0.268 | Upregulated | 8.74E-03 | 6.99E-02 |
| DTX3L | 0.268 | Upregulated | 5.21E-03 | 4.92E-02 |
| MARCKS | 0.269 | Upregulated | 1.41E-03 | 1.99E-02 |
| CD55 | 0.269 | Upregulated | 1.26E-03 | 1.83E-02 |
| CXCL16 | 0.269 | Upregulated | 2.14E-04 | 5.56E-03 |
| FKBP15 | 0.269 | Upregulated | 9.14E-05 | 3.02E-03 |
| HERC3 | 0.270 | Upregulated | 1.46E-04 | 4.22E-03 |
| RCVRN | 0.270 | Upregulated | 7.38E-04 | 1.28E-02 |
| ERI1 | 0.270 | Upregulated | 1.55E-02 | 1.04E-01 |
| MT1P2 | 0.270 | Upregulated | 1.18E-04 | 3.63E-03 |
| KLHL21 | 0.271 | Upregulated | 7.04E-04 | 1.24E-02 |
| HINT3 | 0.271 | Upregulated | 2.76E-06 | 2.68E-04 |
| C5ORF41 | 0.271 | Upregulated | 9.09E-06 | 6.09E-04 |
| OSGIN2 | 0.272 | Upregulated | 4.66E-06 | 3.85E-04 |
| SRGN | 0.272 | Upregulated | 6.82E-06 | 5.01E-04 |
| FAM20A | 0.272 | Upregulated | 2.77E-02 | 1.53E-01 |
| RAB43 | 0.272 | Upregulated | 2.94E-05 | 1.40E-03 |
| CMIP | 0.273 | Upregulated | 1.78E-04 | 4.87E-03 |
| AVIL | 0.273 | Upregulated | 8.81E-05 | 2.95E-03 |
| NFE2L2 | 0.274 | Upregulated | 1.26E-05 | 7.61E-04 |
| ADAP2 | 0.274 | Upregulated | 1.25E-03 | 1.83E-02 |
| PIK3AP1 | 0.274 | Upregulated | 2.23E-02 | 1.32E-01 |
| MTMR3 | 0.274 | Upregulated | 8.14E-04 | 1.37E-02 |
| P2RY12 | 0.274 | Upregulated | 2.04E-04 | 5.40E-03 |
| SRPK1 | 0.274 | Upregulated | 6.00E-04 | 1.11E-02 |
| MAP1LC3B2 | 0.275 | Upregulated | 1.75E-03 | 2.31E-02 |
| ARHGEF10L | 0.275 | Upregulated | 1.11E-07 | 2.66E-05 |
| WWC3 | 0.276 | Upregulated | 3.29E-03 | 3.60E-02 |
| NLRP12 | 0.276 | Upregulated | 1.84E-02 | 1.16E-01 |
| HEMK1 | 0.276 | Upregulated | 2.02E-05 | 1.07E-03 |
| CSAD | 0.277 | Upregulated | 1.08E-05 | 6.74E-04 |
| NUAK2 | 0.277 | Upregulated | 1.75E-05 | 9.61E-04 |
| CCDC17 | 0.277 | Upregulated | 1.62E-04 | 4.56E-03 |
| GLRX5 | 0.277 | Upregulated | 3.44E-02 | 1.76E-01 |
| ERO1L | 0.279 | Upregulated | 9.17E-05 | 3.02E-03 |
| NUP214 | 0.279 | Upregulated | 8.61E-04 | 1.42E-02 |
| THOC5 | 0.279 | Upregulated | 6.26E-05 | 2.34E-03 |
| CLEC2B | 0.279 | Upregulated | 1.77E-03 | 2.32E-02 |
| FIG4 | 0.280 | Upregulated | 7.00E-07 | 1.02E-04 |
| CD33 | 0.280 | Upregulated | 1.56E-03 | 2.13E-02 |

| | | | | |
|---|---|---|---|---|
| ALDH2 | 0.280 | Upregulated | 8.36E-03 | 6.78E-02 |
| C10ORF119 | 0.281 | Upregulated | 3.91E-04 | 8.31E-03 |
| CBARA1 | 0.281 | Upregulated | 5.47E-04 | 1.05E-02 |
| PSG3 | 0.281 | Upregulated | 7.98E-05 | 2.76E-03 |
| FFAR3 | 0.282 | Upregulated | 2.22E-03 | 2.74E-02 |
| MICB | 0.282 | Upregulated | 1.90E-05 | 1.03E-03 |
| MYO1F | 0.282 | Upregulated | 5.05E-03 | 4.80E-02 |
| KLF4 | 0.283 | Upregulated | 5.77E-04 | 1.09E-02 |
| LOXL3 | 0.283 | Upregulated | 2.86E-04 | 6.69E-03 |
| NLRP6 | 0.283 | Upregulated | 4.07E-04 | 8.57E-03 |
| RHOG | 0.283 | Upregulated | 2.39E-04 | 5.98E-03 |
| PREX1 | 0.283 | Upregulated | 2.71E-03 | 3.15E-02 |
| ZNFX1 | 0.283 | Upregulated | 6.82E-05 | 2.47E-03 |
| ZNF350 | 0.284 | Upregulated | 2.70E-04 | 6.43E-03 |
| TMEM165 | 0.284 | Upregulated | 3.71E-03 | 3.89E-02 |
| NTN3 | 0.284 | Upregulated | 1.76E-05 | 9.68E-04 |
| ZFAND3 | 0.284 | Upregulated | 3.96E-05 | 1.71E-03 |
| TSPO | 0.284 | Upregulated | 5.66E-03 | 5.19E-02 |
| LILRA2 | 0.284 | Upregulated | 8.67E-03 | 6.96E-02 |
| CD300C | 0.285 | Upregulated | 7.41E-03 | 6.26E-02 |
| KIAA0040 | 0.285 | Upregulated | 1.06E-04 | 3.33E-03 |
| ARAP3 | 0.285 | Upregulated | 4.59E-03 | 4.50E-02 |
| ANPEP | 0.286 | Upregulated | 4.51E-02 | 2.09E-01 |
| SLC12A9 | 0.286 | Upregulated | 1.20E-04 | 3.67E-03 |
| NLRP3 | 0.286 | Upregulated | 1.06E-03 | 1.64E-02 |
| C20ORF24 | 0.286 | Upregulated | 2.78E-06 | 2.68E-04 |
| TNFRSF10C | 0.286 | Upregulated | 1.21E-03 | 1.78E-02 |
| TRIM5 | 0.286 | Upregulated | 1.10E-03 | 1.67E-02 |
| FADS1 | 0.286 | Upregulated | 1.35E-02 | 9.45E-02 |
| IRF2 | 0.286 | Upregulated | 1.73E-05 | 9.58E-04 |
| ADCY4 | 0.287 | Upregulated | 2.63E-04 | 6.33E-03 |
| DNAJC25-GNG10 | 0.287 | Upregulated | 1.11E-05 | 6.88E-04 |
| AKIRIN2 | 0.287 | Upregulated | 4.32E-06 | 3.68E-04 |
| HAL | 0.287 | Upregulated | 2.23E-03 | 2.75E-02 |
| IFNGR2 | 0.287 | Upregulated | 2.07E-04 | 5.44E-03 |
| CD40 | 0.287 | Upregulated | 2.29E-05 | 1.16E-03 |
| STAT2 | 0.288 | Upregulated | 1.21E-03 | 1.79E-02 |
| ACTA2 | 0.288 | Upregulated | 1.28E-02 | 9.16E-02 |
| TUFT1 | 0.288 | Upregulated | 2.40E-03 | 2.88E-02 |
| Septin 4 | 0.289 | Upregulated | 9.19E-09 | 4.55E-06 |
| SLC15A3 | 0.289 | Upregulated | 1.62E-04 | 4.56E-03 |
| TMEM154 | 0.289 | Upregulated | 1.22E-04 | 3.71E-03 |
| TICAM2 | 0.290 | Upregulated | 1.83E-06 | 2.01E-04 |
| THBS1 | 0.290 | Upregulated | 3.73E-02 | 1.85E-01 |
| ABLIM3 | 0.290 | Upregulated | 3.56E-03 | 3.79E-02 |
| PAPSS2 | 0.290 | Upregulated | 4.73E-04 | 9.56E-03 |
| IMPA2 | 0.291 | Upregulated | 1.14E-02 | 8.44E-02 |
| KLHL2 | 0.291 | Upregulated | 2.59E-02 | 1.47E-01 |
| CNIH4 | 0.291 | Upregulated | 4.49E-03 | 4.45E-02 |
| RIPK2 | 0.291 | Upregulated | 6.48E-08 | 1.78E-05 |
| CHST15 | 0.291 | Upregulated | 1.19E-03 | 1.77E-02 |

| | | | | |
|---|---|---|---|---|
| PHF21A | 0.291 | Upregulated | 1.47E-05 | 8.43E-04 |
| SLITRK4 | 0.291 | Upregulated | 2.17E-04 | 5.61E-03 |
| KIAA1009 | 0.291 | Upregulated | 1.85E-06 | 2.02E-04 |
| FLJ20489 | 0.292 | Upregulated | 3.88E-02 | 1.90E-01 |
| SPOCD1 | 0.292 | Upregulated | 1.56E-02 | 1.04E-01 |
| HIST1H4D | 0.292 | Upregulated | 3.59E-03 | 3.80E-02 |
| CHMP2A | 0.292 | Upregulated | 2.26E-05 | 1.15E-03 |
| CETP | 0.292 | Upregulated | 1.55E-03 | 2.12E-02 |
| DTNBP1 | 0.293 | Upregulated | 6.45E-06 | 4.83E-04 |
| GNAI3 | 0.293 | Upregulated | 3.38E-04 | 7.52E-03 |
| UBR2 | 0.293 | Upregulated | 1.33E-07 | 2.99E-05 |
| RBMS1 | 0.293 | Upregulated | 8.29E-04 | 1.38E-02 |
| STARD10 | 0.293 | Upregulated | 1.42E-04 | 4.16E-03 |
| ACRBP | 0.293 | Upregulated | 2.96E-02 | 1.60E-01 |
| MUTYH | 0.294 | Upregulated | 1.43E-06 | 1.71E-04 |
| SLC15A4 | 0.294 | Upregulated | 1.02E-04 | 3.24E-03 |
| CFLAR | 0.294 | Upregulated | 6.70E-04 | 1.20E-02 |
| FLOT2 | 0.294 | Upregulated | 5.84E-04 | 1.09E-02 |
| PHCA | 0.294 | Upregulated | 2.61E-04 | 6.31E-03 |
| ZC3H12A | 0.295 | Upregulated | 2.23E-03 | 2.75E-02 |
| KIAA0232 | 0.295 | Upregulated | 1.63E-03 | 2.20E-02 |
| OSTALPHA | 0.295 | Upregulated | 2.02E-02 | 1.25E-01 |
| TESK2 | 0.295 | Upregulated | 3.17E-07 | 5.79E-05 |
| TMLHE | 0.295 | Upregulated | 2.09E-08 | 8.72E-06 |
| CALCOCO2 | 0.296 | Upregulated | 6.91E-06 | 5.03E-04 |
| VASP | 0.296 | Upregulated | 8.44E-05 | 2.88E-03 |
| MOBK1B | 0.296 | Upregulated | 5.86E-06 | 4.60E-04 |
| CHMP5 | 0.296 | Upregulated | 4.93E-03 | 4.73E-02 |
| S100A11 | 0.296 | Upregulated | 5.21E-03 | 4.92E-02 |
| CENTD2 | 0.297 | Upregulated | 6.25E-08 | 1.75E-05 |
| ASAP2 | 0.298 | Upregulated | 2.61E-04 | 6.30E-03 |
| HIST1H2AC | 0.298 | Upregulated | 1.67E-03 | 2.24E-02 |
| ATF5 | 0.298 | Upregulated | 1.96E-02 | 1.22E-01 |
| NFKB2 | 0.298 | Upregulated | 7.19E-05 | 2.58E-03 |
| AOAH | 0.298 | Upregulated | 4.73E-04 | 9.56E-03 |
| GYPC | 0.298 | Upregulated | 3.75E-02 | 1.86E-01 |
| TMCC3 | 0.299 | Upregulated | 4.78E-04 | 9.62E-03 |
| FAM89A | 0.299 | Upregulated | 3.50E-03 | 3.74E-02 |
| C3AR1 | 0.299 | Upregulated | 1.53E-02 | 1.03E-01 |
| CTTN | 0.299 | Upregulated | 1.64E-02 | 1.08E-01 |
| GPR27 | 0.299 | Upregulated | 6.80E-06 | 5.01E-04 |
| GPER | 0.299 | Upregulated | 4.92E-02 | 2.21E-01 |
| FYB | 0.299 | Upregulated | 5.76E-05 | 2.20E-03 |
| VSIG4 | 0.299 | Upregulated | 3.61E-03 | 3.82E-02 |
| TSPAN9 | 0.299 | Upregulated | 3.87E-02 | 1.90E-01 |
| SOS2 | 0.299 | Upregulated | 4.26E-04 | 8.87E-03 |
| ESAM | 0.300 | Upregulated | 2.97E-02 | 1.60E-01 |
| LTA4H | 0.300 | Upregulated | 4.94E-04 | 9.81E-03 |
| KIAA0556 | 0.300 | Upregulated | 3.00E-07 | 5.54E-05 |
| CSDA | 0.300 | Upregulated | 2.45E-02 | 1.41E-01 |
| OGFR | 0.300 | Upregulated | 1.37E-06 | 1.66E-04 |

| | | | | |
|---|---|---|---|---|
| LGALS3BP | 0.300 | Upregulated | 4.61E-02 | 2.12E-01 |
| TCIRG1 | 0.300 | Upregulated | 3.14E-05 | 1.46E-03 |
| MTF1 | 0.301 | Upregulated | 1.12E-03 | 1.69E-02 |
| IFITM2 | 0.301 | Upregulated | 1.57E-05 | 8.93E-04 |
| C5 | 0.301 | Upregulated | 4.13E-05 | 1.77E-03 |
| TMEM127 | 0.301 | Upregulated | 5.80E-04 | 1.09E-02 |
| ZBTB16 | 0.301 | Upregulated | 8.08E-04 | 1.36E-02 |
| ELMO2 | 0.301 | Upregulated | 1.07E-03 | 1.65E-02 |
| WSB1 | 0.302 | Upregulated | 2.34E-03 | 2.83E-02 |
| PPP4R1 | 0.302 | Upregulated | 1.75E-06 | 1.95E-04 |
| SERPINB1 | 0.302 | Upregulated | 2.40E-03 | 2.88E-02 |
| PYCARD | 0.302 | Upregulated | 4.85E-05 | 1.97E-03 |
| RHBDF2 | 0.303 | Upregulated | 7.11E-08 | 1.91E-05 |
| CHSY1 | 0.303 | Upregulated | 9.79E-05 | 3.15E-03 |
| SKAP2 | 0.303 | Upregulated | 1.84E-04 | 5.02E-03 |
| PDK3 | 0.303 | Upregulated | 6.56E-06 | 4.89E-04 |
| TRIM27 | 0.303 | Upregulated | 1.45E-06 | 1.73E-04 |
| CRK | 0.303 | Upregulated | 3.83E-07 | 6.68E-05 |
| ALOX5AP | 0.303 | Upregulated | 4.24E-04 | 8.85E-03 |
| GBGT1 | 0.304 | Upregulated | 2.00E-03 | 2.54E-02 |
| NBN | 0.304 | Upregulated | 1.70E-03 | 2.26E-02 |
| GNS | 0.304 | Upregulated | 2.50E-04 | 6.15E-03 |
| SLK | 0.304 | Upregulated | 1.18E-04 | 3.63E-03 |
| C4ORF40 | 0.305 | Upregulated | 4.97E-06 | 4.03E-04 |
| TMEM120A | 0.305 | Upregulated | 2.91E-03 | 3.28E-02 |
| ACSBG1 | 0.305 | Upregulated | 1.68E-03 | 2.24E-02 |
| CPVL | 0.306 | Upregulated | 4.21E-03 | 4.24E-02 |
| TOM1 | 0.306 | Upregulated | 3.43E-05 | 1.54E-03 |
| CMTM5 | 0.306 | Upregulated | 2.55E-02 | 1.45E-01 |
| PARP10 | 0.306 | Upregulated | 2.08E-04 | 5.45E-03 |
| MT2A | 0.307 | Upregulated | 2.39E-02 | 1.39E-01 |
| ZYX | 0.307 | Upregulated | 7.62E-04 | 1.31E-02 |
| CD14 | 0.308 | Upregulated | 5.99E-03 | 5.39E-02 |
| C14ORF4 | 0.308 | Upregulated | 1.35E-03 | 1.93E-02 |
| CD58 | 0.308 | Upregulated | 3.34E-04 | 7.47E-03 |
| PFKFB2 | 0.308 | Upregulated | 7.39E-03 | 6.25E-02 |
| C7ORF34 | 0.308 | Upregulated | 4.71E-06 | 3.88E-04 |
| RAB11FIP1 | 0.308 | Upregulated | 3.07E-05 | 1.44E-03 |
| OPLAH | 0.309 | Upregulated | 2.29E-02 | 1.35E-01 |
| GSDMD | 0.309 | Upregulated | 8.29E-08 | 2.11E-05 |
| CYB5R4 | 0.310 | Upregulated | 1.16E-05 | 7.12E-04 |
| PTPRE | 0.310 | Upregulated | 2.27E-05 | 1.16E-03 |
| HECW2 | 0.311 | Upregulated | 5.80E-04 | 1.09E-02 |
| ITPK1 | 0.311 | Upregulated | 3.48E-06 | 3.16E-04 |
| IFNGR1 | 0.311 | Upregulated | 1.47E-04 | 4.23E-03 |
| ODF3B | 0.312 | Upregulated | 9.70E-10 | 9.38E-07 |
| RGL2 | 0.312 | Upregulated | 2.57E-07 | 4.89E-05 |
| LONRF1 | 0.312 | Upregulated | 1.98E-04 | 5.28E-03 |
| ABHD2 | 0.313 | Upregulated | 3.95E-05 | 1.71E-03 |
| IFIT5 | 0.313 | Upregulated | 9.69E-04 | 1.54E-02 |
| APOL2 | 0.313 | Upregulated | 4.80E-09 | 2.92E-06 |

| | | | | |
|---|---|---|---|---|
| LRRC6 | 0.313 | Upregulated | 1.63E-02 | 1.08E-01 |
| FAM176B | 0.313 | Upregulated | 5.62E-06 | 4.42E-04 |
| B9D2 | 0.313 | Upregulated | 5.45E-06 | 4.31E-04 |
| AXUD1 | 0.314 | Upregulated | 5.18E-04 | 1.02E-02 |
| SLC25A44 | 0.314 | Upregulated | 2.80E-05 | 1.36E-03 |
| HIST1H2BK | 0.314 | Upregulated | 4.60E-04 | 9.39E-03 |
| RBCK1 | 0.314 | Upregulated | 1.04E-06 | 1.35E-04 |
| MS4A6A | 0.315 | Upregulated | 2.21E-04 | 5.68E-03 |
| INSL3 | 0.316 | Upregulated | 9.28E-03 | 7.31E-02 |
| RAB32 | 0.316 | Upregulated | 1.61E-03 | 2.18E-02 |
| GRAMD1A | 0.316 | Upregulated | 9.85E-05 | 3.16E-03 |
| HLA-C | 0.316 | Upregulated | 1.63E-02 | 1.08E-01 |
| SP100 | 0.316 | Upregulated | 8.88E-04 | 1.45E-02 |
| SLC19A1 | 0.316 | Upregulated | 3.24E-04 | 7.30E-03 |
| GLIPR2 | 0.316 | Upregulated | 4.04E-06 | 3.49E-04 |
| CKAP4 | 0.316 | Upregulated | 1.03E-02 | 7.88E-02 |
| C3ORF34 | 0.317 | Upregulated | 9.98E-06 | 6.39E-04 |
| GHRL | 0.317 | Upregulated | 6.54E-04 | 1.18E-02 |
| ATP6V1B2 | 0.318 | Upregulated | 4.03E-06 | 3.49E-04 |
| ENTPD1 | 0.319 | Upregulated | 4.23E-05 | 1.80E-03 |
| APH1B | 0.319 | Upregulated | 7.78E-05 | 2.71E-03 |
| IRAK2 | 0.319 | Upregulated | 1.88E-04 | 5.10E-03 |
| ACSL4 | 0.319 | Upregulated | 8.26E-05 | 2.83E-03 |
| CTRL | 0.320 | Upregulated | 5.15E-05 | 2.04E-03 |
| SMAP2 | 0.320 | Upregulated | 2.65E-05 | 1.30E-03 |
| DENND1A | 0.320 | Upregulated | 2.85E-05 | 1.38E-03 |
| C11ORF82 | 0.320 | Upregulated | 1.11E-03 | 1.68E-02 |
| SYTL4 | 0.320 | Upregulated | 4.31E-03 | 4.32E-02 |
| KCNE3 | 0.321 | Upregulated | 1.09E-04 | 3.42E-03 |
| SLC16A5 | 0.321 | Upregulated | 1.21E-04 | 3.69E-03 |
| LTB4R | 0.321 | Upregulated | 6.23E-03 | 5.53E-02 |
| SRBD1 | 0.321 | Upregulated | 8.74E-10 | 8.92E-07 |
| PPP1R15A | 0.321 | Upregulated | 3.35E-05 | 1.51E-03 |
| FHL1 | 0.321 | Upregulated | 4.75E-03 | 4.61E-02 |
| NFKBIA | 0.321 | Upregulated | 3.58E-04 | 7.79E-03 |
| ZFP36 | 0.321 | Upregulated | 1.53E-05 | 8.72E-04 |
| NACC2 | 0.321 | Upregulated | 1.53E-04 | 4.37E-03 |
| F13A1 | 0.321 | Upregulated | 3.27E-02 | 1.70E-01 |
| PACSIN2 | 0.321 | Upregulated | 6.92E-05 | 2.50E-03 |
| MVP | 0.322 | Upregulated | 5.50E-06 | 4.35E-04 |
| USP10 | 0.322 | Upregulated | 3.51E-03 | 3.74E-02 |
| PLAGL1 | 0.323 | Upregulated | 8.00E-07 | 1.12E-04 |
| CTSL1 | 0.323 | Upregulated | 6.28E-03 | 5.56E-02 |
| HIST2H2AB | 0.323 | Upregulated | 1.45E-03 | 2.02E-02 |
| GNG8 | 0.323 | Upregulated | 3.09E-04 | 7.05E-03 |
| MTX1 | 0.324 | Upregulated | 5.03E-07 | 8.16E-05 |
| GLRX | 0.324 | Upregulated | 2.00E-05 | 1.06E-03 |
| SLC46A2 | 0.324 | Upregulated | 3.09E-03 | 3.44E-02 |
| TMEM49 | 0.325 | Upregulated | 1.00E-05 | 6.40E-04 |
| KIAA0367 | 0.325 | Upregulated | 3.55E-02 | 1.80E-01 |
| CMTM6 | 0.325 | Upregulated | 7.61E-07 | 1.09E-04 |

| | | | | |
|---|---|---|---|---|
| IL8 | 0.325 | Upregulated | 1.96E-03 | 2.51E-02 |
| CCRL2 | 0.326 | Upregulated | 6.73E-04 | 1.20E-02 |
| GAA | 0.326 | Upregulated | 3.58E-04 | 7.79E-03 |
| GPR42 | 0.326 | Upregulated | 5.34E-04 | 1.04E-02 |
| IFI35 | 0.326 | Upregulated | 6.00E-03 | 5.39E-02 |
| C4ORF18 | 0.326 | Upregulated | 4.55E-03 | 4.47E-02 |
| ST8SIA4 | 0.326 | Upregulated | 1.85E-07 | 3.84E-05 |
| TMEM180 | 0.327 | Upregulated | 4.25E-04 | 8.86E-03 |
| ATG16L2 | 0.327 | Upregulated | 1.57E-04 | 4.46E-03 |
| SECTM1 | 0.327 | Upregulated | 3.00E-07 | 5.54E-05 |
| CASP4 | 0.328 | Upregulated | 6.21E-09 | 3.47E-06 |
| EMILIN2 | 0.328 | Upregulated | 1.63E-03 | 2.20E-02 |
| RSPH3 | 0.329 | Upregulated | 4.41E-06 | 3.73E-04 |
| HIST1H2BF | 0.329 | Upregulated | 2.24E-03 | 2.75E-02 |
| SELP | 0.329 | Upregulated | 8.24E-03 | 6.71E-02 |
| RILPL2 | 0.329 | Upregulated | 7.51E-06 | 5.33E-04 |
| RAB3D | 0.329 | Upregulated | 2.89E-05 | 1.39E-03 |
| CLEC7A | 0.329 | Upregulated | 3.27E-03 | 3.58E-02 |
| C17ORF62 | 0.329 | Upregulated | 2.76E-07 | 5.22E-05 |
| PPP1R3B | 0.330 | Upregulated | 9.04E-06 | 6.08E-04 |
| IGF2R | 0.330 | Upregulated | 6.88E-04 | 1.22E-02 |
| JMJD1C | 0.330 | Upregulated | 1.06E-05 | 6.69E-04 |
| PSCD4 | 0.330 | Upregulated | 9.83E-06 | 6.34E-04 |
| MRVI1 | 0.331 | Upregulated | 1.36E-05 | 8.05E-04 |
| IL4R | 0.331 | Upregulated | 1.67E-03 | 2.24E-02 |
| MLKL | 0.331 | Upregulated | 1.86E-05 | 1.01E-03 |
| HTATIP2 | 0.331 | Upregulated | 5.14E-07 | 8.29E-05 |
| TMC4 | 0.331 | Upregulated | 3.80E-05 | 1.67E-03 |
| NSUN7 | 0.331 | Upregulated | 1.60E-02 | 1.06E-01 |
| SH3GLB1 | 0.331 | Upregulated | 1.78E-04 | 4.89E-03 |
| DHRS13 | 0.331 | Upregulated | 3.58E-04 | 7.79E-03 |
| BAZ2B | 0.331 | Upregulated | 1.23E-05 | 7.47E-04 |
| TMEM164 | 0.332 | Upregulated | 1.25E-07 | 2.90E-05 |
| RHOT1 | 0.332 | Upregulated | 1.60E-07 | 3.43E-05 |
| RIT1 | 0.332 | Upregulated | 5.61E-06 | 4.42E-04 |
| SLC25A37 | 0.332 | Upregulated | 9.68E-03 | 7.53E-02 |
| MR1 | 0.333 | Upregulated | 2.75E-06 | 2.68E-04 |
| C4ORF3 | 0.333 | Upregulated | 1.01E-04 | 3.21E-03 |
| RELB | 0.333 | Upregulated | 2.06E-06 | 2.20E-04 |
| GBA | 0.333 | Upregulated | 2.31E-04 | 5.83E-03 |
| WDFY1 | 0.334 | Upregulated | 6.35E-06 | 4.82E-04 |
| LPGAT1 | 0.335 | Upregulated | 3.38E-06 | 3.11E-04 |
| DDIT3 | 0.335 | Upregulated | 6.29E-05 | 2.34E-03 |
| NAIP | 0.336 | Upregulated | 5.13E-04 | 1.01E-02 |
| CEACAM4 | 0.336 | Upregulated | 2.40E-04 | 5.99E-03 |
| CADM4 | 0.336 | Upregulated | 1.59E-04 | 4.49E-03 |
| UBTD1 | 0.337 | Upregulated | 2.31E-03 | 2.81E-02 |
| LILRB3 | 0.337 | Upregulated | 2.60E-06 | 2.60E-04 |
| GUCY1A3 | 0.338 | Upregulated | 2.71E-04 | 6.47E-03 |
| MXD3 | 0.338 | Upregulated | 7.01E-04 | 1.24E-02 |
| NFE2 | 0.339 | Upregulated | 1.92E-03 | 2.47E-02 |

| | | | | |
|---|---|---|---|---|
| PAK1 | 0.339 | Upregulated | 1.82E-06 | 2.00E-04 |
| ADAM8 | 0.339 | Upregulated | 1.27E-05 | 7.63E-04 |
| C6ORF150 | 0.339 | Upregulated | 1.27E-03 | 1.85E-02 |
| C10ORF54 | 0.339 | Upregulated | 4.37E-04 | 9.05E-03 |
| STAT1 | 0.340 | Upregulated | 7.27E-05 | 2.59E-03 |
| SLCO3A1 | 0.340 | Upregulated | 3.37E-06 | 3.10E-04 |
| ITPRIPL2 | 0.341 | Upregulated | 5.55E-04 | 1.06E-02 |
| ETV6 | 0.341 | Upregulated | 4.14E-08 | 1.33E-05 |
| SLC9A8 | 0.341 | Upregulated | 2.31E-05 | 1.17E-03 |
| NDUFAF3 | 0.342 | Upregulated | 7.90E-03 | 6.51E-02 |
| FAM8A1 | 0.342 | Upregulated | 7.68E-06 | 5.40E-04 |
| SAMD14 | 0.342 | Upregulated | 1.48E-02 | 1.01E-01 |
| RENBP | 0.343 | Upregulated | 2.82E-06 | 2.71E-04 |
| HCG9 | 0.343 | Upregulated | 1.43E-03 | 2.00E-02 |
| SAMHD1 | 0.343 | Upregulated | 1.92E-04 | 5.18E-03 |
| TCN1 | 0.343 | Upregulated | 3.33E-02 | 1.72E-01 |
| PDLIM7 | 0.344 | Upregulated | 3.80E-05 | 1.67E-03 |
| MOV10 | 0.344 | Upregulated | 2.02E-04 | 5.36E-03 |
| CYBB | 0.345 | Upregulated | 2.66E-06 | 2.63E-04 |
| SELPLG | 0.345 | Upregulated | 6.55E-04 | 1.18E-02 |
| TMEM144 | 0.345 | Upregulated | 4.04E-03 | 4.14E-02 |
| GRINA | 0.345 | Upregulated | 2.68E-03 | 3.12E-02 |
| LYRM1 | 0.346 | Upregulated | 3.29E-08 | 1.15E-05 |
| PIK3CB | 0.346 | Upregulated | 7.00E-04 | 1.24E-02 |
| NETO2 | 0.346 | Upregulated | 4.94E-05 | 1.99E-03 |
| PLEKHO2 | 0.346 | Upregulated | 1.08E-05 | 6.78E-04 |
| RNF130 | 0.347 | Upregulated | 2.17E-04 | 5.61E-03 |
| NDST1 | 0.347 | Upregulated | 2.27E-05 | 1.15E-03 |
| VWF | 0.347 | Upregulated | 2.63E-02 | 1.48E-01 |
| GUK1 | 0.347 | Upregulated | 1.53E-02 | 1.03E-01 |
| CLEC1A | 0.347 | Upregulated | 2.58E-05 | 1.27E-03 |
| C21ORF7 | 0.348 | Upregulated | 2.60E-03 | 3.06E-02 |
| SLC43A2 | 0.348 | Upregulated | 3.80E-04 | 8.14E-03 |
| LITAF | 0.348 | Upregulated | 3.13E-06 | 2.95E-04 |
| CXCL1 | 0.348 | Upregulated | 4.58E-05 | 1.89E-03 |
| MAP1A | 0.349 | Upregulated | 6.03E-03 | 5.41E-02 |
| ZNF230 | 0.349 | Upregulated | 1.19E-06 | 1.47E-04 |
| NUMB | 0.349 | Upregulated | 9.19E-06 | 6.12E-04 |
| TFPI | 0.349 | Upregulated | 2.20E-03 | 2.73E-02 |
| STAT3 | 0.349 | Upregulated | 4.95E-06 | 4.03E-04 |
| ECHDC3 | 0.350 | Upregulated | 1.25E-02 | 8.98E-02 |
| SIGLEC9 | 0.350 | Upregulated | 2.57E-03 | 3.04E-02 |
| ZBP1 | 0.351 | Upregulated | 3.21E-03 | 3.54E-02 |
| DDEF2 | 0.351 | Upregulated | 8.81E-05 | 2.95E-03 |
| SULT1B1 | 0.352 | Upregulated | 2.32E-03 | 2.82E-02 |
| SDPR | 0.352 | Upregulated | 4.89E-03 | 4.71E-02 |
| EDG4 | 0.352 | Upregulated | 5.22E-06 | 4.18E-04 |
| EPHB1 | 0.352 | Upregulated | 8.03E-05 | 2.77E-03 |
| CPEB4 | 0.353 | Upregulated | 1.70E-03 | 2.26E-02 |
| SLC2A3 | 0.353 | Upregulated | 5.71E-04 | 1.08E-02 |
| HSD3B7 | 0.353 | Upregulated | 1.02E-03 | 1.59E-02 |

| | | | | |
|---|---|---|---|---|
| SLC45A4 | 0.353 | Upregulated | 6.33E-06 | 4.82E-04 |
| C9ORF72 | 0.355 | Upregulated | 4.16E-06 | 3.57E-04 |
| NINJ1 | 0.355 | Upregulated | 1.74E-04 | 4.80E-03 |
| TLR1 | 0.356 | Upregulated | 2.08E-02 | 1.27E-01 |
| C19ORF38 | 0.356 | Upregulated | 7.81E-06 | 5.47E-04 |
| TMEM55A | 0.356 | Upregulated | 4.47E-05 | 1.87E-03 |
| PGD | 0.356 | Upregulated | 3.35E-03 | 3.63E-02 |
| HAUS4 | 0.356 | Upregulated | 9.21E-05 | 3.02E-03 |
| FLJ42957 | 0.356 | Upregulated | 1.36E-04 | 4.00E-03 |
| CYTH4 | 0.357 | Upregulated | 1.07E-05 | 6.72E-04 |
| LPPR2 | 0.359 | Upregulated | 2.75E-04 | 6.52E-03 |
| VAMP3 | 0.359 | Upregulated | 3.83E-07 | 6.68E-05 |
| PYGL | 0.360 | Upregulated | 4.73E-03 | 4.59E-02 |
| SDCBP | 0.360 | Upregulated | 2.99E-04 | 6.90E-03 |
| CD163 | 0.360 | Upregulated | 1.07E-02 | 8.09E-02 |
| CTBS | 0.361 | Upregulated | 8.48E-06 | 5.82E-04 |
| HCK | 0.361 | Upregulated | 1.07E-03 | 1.65E-02 |
| ATF6 | 0.361 | Upregulated | 1.00E-07 | 2.49E-05 |
| C9ORF164 | 0.362 | Upregulated | 5.96E-06 | 4.65E-04 |
| CLEC4A | 0.362 | Upregulated | 5.91E-05 | 2.24E-03 |
| AIF1 | 0.362 | Upregulated | 1.24E-04 | 3.75E-03 |
| CDS2 | 0.362 | Upregulated | 1.70E-06 | 1.92E-04 |
| FAM160B1 | 0.363 | Upregulated | 1.57E-04 | 4.46E-03 |
| NPC2 | 0.363 | Upregulated | 6.24E-08 | 1.75E-05 |
| GALM | 0.364 | Upregulated | 4.82E-04 | 9.66E-03 |
| TNFSF14 | 0.364 | Upregulated | 1.99E-04 | 5.30E-03 |
| ACTN1 | 0.364 | Upregulated | 1.50E-04 | 4.30E-03 |
| LPAR2 | 0.364 | Upregulated | 4.05E-06 | 3.49E-04 |
| TDRD9 | 0.365 | Upregulated | 2.76E-02 | 1.53E-01 |
| GYG1 | 0.366 | Upregulated | 4.12E-03 | 4.19E-02 |
| PTPRJ | 0.366 | Upregulated | 6.06E-06 | 4.70E-04 |
| OBFC2A | 0.367 | Upregulated | 1.57E-05 | 8.94E-04 |
| AKAP13 | 0.367 | Upregulated | 9.14E-07 | 1.24E-04 |
| CAPNS2 | 0.367 | Upregulated | 1.39E-05 | 8.15E-04 |
| EMR1 | 0.368 | Upregulated | 1.22E-02 | 8.87E-02 |
| PISD | 0.368 | Upregulated | 9.01E-06 | 6.07E-04 |
| NRGN | 0.368 | Upregulated | 1.66E-02 | 1.09E-01 |
| PROK1 | 0.368 | Upregulated | 6.55E-07 | 9.80E-05 |
| TRIM25 | 0.369 | Upregulated | 6.65E-05 | 2.43E-03 |
| DDX58 | 0.370 | Upregulated | 6.84E-05 | 2.47E-03 |
| PGCP | 0.371 | Upregulated | 3.79E-04 | 8.13E-03 |
| RGL4 | 0.371 | Upregulated | 5.02E-03 | 4.78E-02 |
| WDFY3 | 0.371 | Upregulated | 9.47E-06 | 6.19E-04 |
| LBA1 | 0.371 | Upregulated | 5.68E-07 | 8.92E-05 |
| IFITM1 | 0.372 | Upregulated | 1.52E-05 | 8.69E-04 |
| DENND5A | 0.373 | Upregulated | 8.87E-08 | 2.23E-05 |
| HIST1H2AE | 0.374 | Upregulated | 9.21E-05 | 3.02E-03 |
| CYP4F3 | 0.374 | Upregulated | 2.24E-02 | 1.33E-01 |
| FZD2 | 0.376 | Upregulated | 4.68E-04 | 9.50E-03 |
| CDC42EP2 | 0.377 | Upregulated | 8.50E-05 | 2.89E-03 |
| TYROBP | 0.377 | Upregulated | 2.87E-06 | 2.75E-04 |

| | | | | |
|---|---|---|---|---|
| GM2A | 0.377 | Upregulated | 1.95E-05 | 1.04E-03 |
| ARHGAP26 | 0.377 | Upregulated | 3.65E-05 | 1.62E-03 |
| CTDSPL | 0.377 | Upregulated | 9.14E-03 | 7.22E-02 |
| C1QA | 0.377 | Upregulated | 8.81E-06 | 5.99E-04 |
| PSMB9 | 0.378 | Upregulated | 2.99E-08 | 1.09E-05 |
| FAR2 | 0.378 | Upregulated | 2.88E-05 | 1.39E-03 |
| DHRS12 | 0.378 | Upregulated | 1.54E-07 | 3.36E-05 |
| NT5M | 0.378 | Upregulated | 1.00E-03 | 1.58E-02 |
| RNASEL | 0.378 | Upregulated | 5.81E-08 | 1.67E-05 |
| PARP14 | 0.378 | Upregulated | 6.76E-06 | 5.00E-04 |
| CDA | 0.378 | Upregulated | 5.54E-03 | 5.11E-02 |
| TSEN34 | 0.379 | Upregulated | 1.80E-06 | 1.99E-04 |
| KIFC3 | 0.379 | Upregulated | 9.82E-04 | 1.55E-02 |
| DDAH2 | 0.380 | Upregulated | 4.39E-05 | 1.85E-03 |
| TRPM6 | 0.380 | Upregulated | 2.59E-03 | 3.05E-02 |
| HIST1H2BD | 0.380 | Upregulated | 2.41E-03 | 2.88E-02 |
| CEBPB | 0.380 | Upregulated | 4.09E-05 | 1.76E-03 |
| GNB4 | 0.381 | Upregulated | 1.10E-05 | 6.83E-04 |
| ITGAX | 0.381 | Upregulated | 7.01E-05 | 2.52E-03 |
| CKLF | 0.382 | Upregulated | 1.13E-05 | 6.95E-04 |
| IL1F9 | 0.382 | Upregulated | 3.51E-05 | 1.57E-03 |
| TRIB1 | 0.382 | Upregulated | 4.55E-05 | 1.88E-03 |
| TM6SF1 | 0.382 | Upregulated | 1.22E-04 | 3.71E-03 |
| MPZL1 | 0.383 | Upregulated | 8.47E-04 | 1.40E-02 |
| TREML1 | 0.383 | Upregulated | 1.16E-02 | 8.57E-02 |
| KLHDC8B | 0.384 | Upregulated | 7.85E-04 | 1.34E-02 |
| SESTD1 | 0.384 | Upregulated | 1.08E-09 | 9.88E-07 |
| IL17RA | 0.384 | Upregulated | 2.93E-04 | 6.81E-03 |
| SPARC | 0.384 | Upregulated | 3.22E-03 | 3.54E-02 |
| CLEC12A | 0.385 | Upregulated | 6.53E-03 | 5.72E-02 |
| ITPRIP | 0.385 | Upregulated | 4.40E-07 | 7.49E-05 |
| MANSC1 | 0.386 | Upregulated | 4.44E-03 | 4.42E-02 |
| CTRC | 0.387 | Upregulated | 1.34E-06 | 1.64E-04 |
| BEND7 | 0.387 | Upregulated | 8.10E-06 | 5.66E-04 |
| PARP9 | 0.387 | Upregulated | 2.69E-05 | 1.32E-03 |
| MMP25 | 0.387 | Upregulated | 5.27E-04 | 1.03E-02 |
| C5ORF32 | 0.388 | Upregulated | 6.57E-03 | 5.75E-02 |
| SMCHD1 | 0.388 | Upregulated | 3.36E-08 | 1.16E-05 |
| JAM3 | 0.389 | Upregulated | 3.40E-03 | 3.67E-02 |
| C15ORF39 | 0.389 | Upregulated | 7.82E-07 | 1.10E-04 |
| PF4V1 | 0.389 | Upregulated | 3.05E-02 | 1.63E-01 |
| PPBP | 0.389 | Upregulated | 4.86E-03 | 4.68E-02 |
| AIG1 | 0.390 | Upregulated | 2.66E-06 | 2.63E-04 |
| C14ORF147 | 0.390 | Upregulated | 9.68E-06 | 6.27E-04 |
| IER3 | 0.390 | Upregulated | 1.01E-03 | 1.59E-02 |
| S1PR3 | 0.391 | Upregulated | 1.68E-04 | 4.68E-03 |
| FBXL13 | 0.391 | Upregulated | 1.29E-04 | 3.86E-03 |
| SIRPD | 0.391 | Upregulated | 4.60E-05 | 1.89E-03 |
| C5AR1 | 0.391 | Upregulated | 7.24E-04 | 1.26E-02 |
| PFKFB3 | 0.392 | Upregulated | 5.33E-04 | 1.04E-02 |
| NRBF2 | 0.392 | Upregulated | 1.18E-06 | 1.46E-04 |

| | | | | |
|---|---|---|---|---|
| SSFA2 | 0.392 | Upregulated | 1.89E-06 | 2.06E-04 |
| UBE2L6 | 0.393 | Upregulated | 4.88E-06 | 4.00E-04 |
| REPS2 | 0.393 | Upregulated | 2.88E-04 | 6.70E-03 |
| ZNF467 | 0.393 | Upregulated | 1.44E-03 | 2.01E-02 |
| SHKBP1 | 0.393 | Upregulated | 1.23E-05 | 7.47E-04 |
| DYNLT1 | 0.393 | Upregulated | 7.16E-07 | 1.04E-04 |
| KCNMB1 | 0.394 | Upregulated | 2.00E-05 | 1.06E-03 |
| MPL | 0.394 | Upregulated | 1.16E-02 | 8.55E-02 |
| SELL | 0.394 | Upregulated | 2.66E-06 | 2.63E-04 |
| JAK2 | 0.396 | Upregulated | 1.17E-06 | 1.46E-04 |
| CFH | 0.397 | Upregulated | 2.64E-04 | 6.35E-03 |
| NFXL1 | 0.397 | Upregulated | 2.63E-03 | 3.08E-02 |
| OSCAR | 0.397 | Upregulated | 9.35E-04 | 1.50E-02 |
| C7ORF53 | 0.398 | Upregulated | 1.09E-03 | 1.67E-02 |
| BAZ1A | 0.399 | Upregulated | 1.12E-08 | 5.35E-06 |
| HLX | 0.399 | Upregulated | 4.27E-05 | 1.82E-03 |
| TAP1 | 0.399 | Upregulated | 1.96E-04 | 5.24E-03 |
| SPI1 | 0.400 | Upregulated | 1.54E-04 | 4.38E-03 |
| RNF144B | 0.401 | Upregulated | 1.30E-04 | 3.89E-03 |
| APOB48R | 0.401 | Upregulated | 1.97E-03 | 2.51E-02 |
| SAMD4A | 0.401 | Upregulated | 2.23E-05 | 1.15E-03 |
| TRAFD1 | 0.401 | Upregulated | 1.85E-07 | 3.84E-05 |
| SAT1 | 0.402 | Upregulated | 5.37E-08 | 1.59E-05 |
| SNX20 | 0.403 | Upregulated | 2.17E-07 | 4.43E-05 |
| ITGA2B | 0.403 | Upregulated | 2.71E-02 | 1.51E-01 |
| PROS1 | 0.405 | Upregulated | 9.65E-03 | 7.51E-02 |
| CLEC1B | 0.406 | Upregulated | 1.57E-02 | 1.05E-01 |
| CXCL9 | 0.406 | Upregulated | 3.73E-05 | 1.65E-03 |
| IRF7 | 0.407 | Upregulated | 7.94E-03 | 6.54E-02 |
| TAP2 | 0.407 | Upregulated | 2.90E-09 | 1.97E-06 |
| RERE | 0.407 | Upregulated | 1.21E-07 | 2.86E-05 |
| OBFC1 | 0.407 | Upregulated | 5.71E-09 | 3.30E-06 |
| KIAA0247 | 0.407 | Upregulated | 1.68E-07 | 3.59E-05 |
| DOK3 | 0.407 | Upregulated | 1.98E-04 | 5.29E-03 |
| RRAGD | 0.407 | Upregulated | 1.15E-04 | 3.55E-03 |
| TREML4 | 0.407 | Upregulated | 3.88E-03 | 4.03E-02 |
| HIST1H3D | 0.409 | Upregulated | 1.03E-04 | 3.26E-03 |
| UBXN2B | 0.409 | Upregulated | 1.01E-06 | 1.33E-04 |
| LAT2 | 0.409 | Upregulated | 6.51E-07 | 9.80E-05 |
| FLJ22662 | 0.409 | Upregulated | 1.37E-03 | 1.95E-02 |
| TAPBP | 0.409 | Upregulated | 3.23E-09 | 2.07E-06 |
| LYN | 0.409 | Upregulated | 8.66E-07 | 1.19E-04 |
| KCNE1 | 0.410 | Upregulated | 6.58E-03 | 5.75E-02 |
| GLDN | 0.410 | Upregulated | 2.27E-03 | 2.78E-02 |
| LACTB | 0.411 | Upregulated | 3.97E-06 | 3.48E-04 |
| HIST1H1T | 0.411 | Upregulated | 8.29E-05 | 2.84E-03 |
| RAB3IL1 | 0.411 | Upregulated | 3.51E-02 | 1.78E-01 |
| B4GALT5 | 0.411 | Upregulated | 2.79E-04 | 6.57E-03 |
| AGPAT9 | 0.411 | Upregulated | 2.16E-03 | 2.69E-02 |
| SLC22A4 | 0.412 | Upregulated | 1.02E-03 | 1.59E-02 |
| CSTA | 0.412 | Upregulated | 1.97E-04 | 5.28E-03 |

| | | | | |
|---|---|---|---|---|
| AMICA1 | 0.414 | Upregulated | 6.06E-06 | 4.70E-04 |
| PPP1R3D | 0.415 | Upregulated | 2.39E-06 | 2.43E-04 |
| IFI30 | 0.416 | Upregulated | 6.38E-06 | 4.83E-04 |
| HRH2 | 0.416 | Upregulated | 4.91E-04 | 9.80E-03 |
| IL6R | 0.416 | Upregulated | 6.21E-05 | 2.33E-03 |
| TRIM21 | 0.416 | Upregulated | 4.98E-09 | 2.98E-06 |
| XPO6 | 0.417 | Upregulated | 6.43E-06 | 4.83E-04 |
| ASPHD2 | 0.417 | Upregulated | 1.24E-09 | 1.08E-06 |
| FES | 0.417 | Upregulated | 5.14E-05 | 2.04E-03 |
| CASP1 | 0.418 | Upregulated | 2.54E-07 | 4.86E-05 |
| IFNAR1 | 0.420 | Upregulated | 8.37E-08 | 2.12E-05 |
| ITGAM | 0.420 | Upregulated | 4.06E-04 | 8.56E-03 |
| SH3BGRL2 | 0.420 | Upregulated | 4.13E-03 | 4.20E-02 |
| GCA | 0.420 | Upregulated | 4.83E-05 | 1.96E-03 |
| EIF4E3 | 0.420 | Upregulated | 2.00E-07 | 4.13E-05 |
| BRI3 | 0.420 | Upregulated | 5.27E-06 | 4.20E-04 |
| PELI1 | 0.420 | Upregulated | 2.61E-08 | 9.74E-06 |
| CATSPER1 | 0.421 | Upregulated | 3.85E-05 | 1.68E-03 |
| FAS | 0.421 | Upregulated | 4.35E-07 | 7.43E-05 |
| FLJ14166 | 0.421 | Upregulated | 2.97E-05 | 1.41E-03 |
| CTSA | 0.421 | Upregulated | 4.05E-05 | 1.74E-03 |
| C17ORF60 | 0.422 | Upregulated | 1.22E-04 | 3.71E-03 |
| TSC22D3 | 0.423 | Upregulated | 2.72E-04 | 6.49E-03 |
| SVIL | 0.423 | Upregulated | 5.46E-04 | 1.05E-02 |
| EIF2C4 | 0.423 | Upregulated | 1.73E-05 | 9.58E-04 |
| KDM6B | 0.424 | Upregulated | 4.26E-07 | 7.32E-05 |
| SEMA4A | 0.424 | Upregulated | 3.32E-04 | 7.43E-03 |
| RP2 | 0.424 | Upregulated | 1.91E-05 | 1.03E-03 |
| NFIL3 | 0.424 | Upregulated | 7.82E-05 | 2.72E-03 |
| PFKFB4 | 0.425 | Upregulated | 5.24E-05 | 2.06E-03 |
| CTSS | 0.425 | Upregulated | 3.02E-08 | 1.09E-05 |
| NCF2 | 0.425 | Upregulated | 5.08E-05 | 2.02E-03 |
| IL15 | 0.426 | Upregulated | 1.78E-08 | 7.82E-06 |
| EPB41L3 | 0.427 | Upregulated | 3.28E-06 | 3.04E-04 |
| FBXL5 | 0.427 | Upregulated | 2.74E-06 | 2.68E-04 |
| SNX10 | 0.428 | Upregulated | 1.04E-04 | 3.29E-03 |
| FLOT1 | 0.429 | Upregulated | 1.26E-05 | 7.61E-04 |
| C9ORF66 | 0.429 | Upregulated | 7.51E-08 | 1.98E-05 |
| RAB31 | 0.429 | Upregulated | 5.35E-06 | 4.25E-04 |
| FLJ10357 | 0.430 | Upregulated | 1.42E-05 | 8.29E-04 |
| SQRDL | 0.431 | Upregulated | 4.99E-07 | 8.12E-05 |
| PRRG4 | 0.433 | Upregulated | 9.79E-10 | 9.38E-07 |
| PELI2 | 0.433 | Upregulated | 4.78E-08 | 1.47E-05 |
| GLT1D1 | 0.433 | Upregulated | 5.02E-05 | 2.00E-03 |
| ZCCHC6 | 0.433 | Upregulated | 3.44E-07 | 6.24E-05 |
| ECE1 | 0.433 | Upregulated | 9.70E-05 | 3.14E-03 |
| CMTM2 | 0.434 | Upregulated | 1.37E-02 | 9.56E-02 |
| FRMD3 | 0.434 | Upregulated | 3.12E-05 | 1.46E-03 |
| CMBL | 0.434 | Upregulated | 1.21E-02 | 8.80E-02 |
| LATS2 | 0.435 | Upregulated | 1.66E-06 | 1.87E-04 |
| MNDA | 0.435 | Upregulated | 1.66E-05 | 9.31E-04 |

| | | | | |
|---|---|---|---|---|
| ECGF1 | 0.438 | Upregulated | 4.53E-06 | 3.80E-04 |
| DRAM1 | 0.438 | Upregulated | 1.06E-05 | 6.69E-04 |
| CD300LF | 0.439 | Upregulated | 3.52E-06 | 3.19E-04 |
| TSHZ3 | 0.440 | Upregulated | 2.15E-04 | 5.58E-03 |
| FAM26F | 0.441 | Upregulated | 1.60E-04 | 4.50E-03 |
| HPSE | 0.441 | Upregulated | 2.27E-04 | 5.75E-03 |
| HIST1H2BG | 0.441 | Upregulated | 7.42E-05 | 2.62E-03 |
| KIF1B | 0.441 | Upregulated | 1.26E-04 | 3.79E-03 |
| TNFRSF1A | 0.442 | Upregulated | 3.92E-06 | 3.46E-04 |
| VNN1 | 0.442 | Upregulated | 2.49E-02 | 1.43E-01 |
| ST3GAL6 | 0.444 | Upregulated | 9.93E-06 | 6.37E-04 |
| KIAA1598 | 0.444 | Upregulated | 9.17E-05 | 3.02E-03 |
| FNDC3B | 0.444 | Upregulated | 2.78E-07 | 5.22E-05 |
| EVI2A | 0.444 | Upregulated | 3.23E-04 | 7.28E-03 |
| ASGR2 | 0.445 | Upregulated | 6.61E-04 | 1.19E-02 |
| NUDT16 | 0.446 | Upregulated | 8.04E-08 | 2.08E-05 |
| MSL1 | 0.446 | Upregulated | 5.55E-07 | 8.84E-05 |
| NADK | 0.446 | Upregulated | 5.36E-07 | 8.57E-05 |
| C19ORF35 | 0.446 | Upregulated | 2.90E-04 | 6.74E-03 |
| TRIM22 | 0.447 | Upregulated | 6.28E-06 | 4.80E-04 |
| IL1R2 | 0.447 | Upregulated | 2.74E-04 | 6.50E-03 |
| TYMP | 0.447 | Upregulated | 7.50E-07 | 1.08E-04 |
| SLC2A14 | 0.447 | Upregulated | 8.86E-04 | 1.45E-02 |
| HK3 | 0.448 | Upregulated | 1.00E-03 | 1.58E-02 |
| PHTF1 | 0.448 | Upregulated | 1.05E-06 | 1.36E-04 |
| RNF13 | 0.449 | Upregulated | 2.42E-09 | 1.75E-06 |
| CDK5RAP2 | 0.450 | Upregulated | 1.28E-04 | 3.84E-03 |
| SIGLEC5 | 0.450 | Upregulated | 3.62E-04 | 7.85E-03 |
| DGAT2 | 0.450 | Upregulated | 1.75E-05 | 9.65E-04 |
| METTL7B | 0.451 | Upregulated | 9.01E-03 | 7.15E-02 |
| SV2A | 0.451 | Upregulated | 5.91E-06 | 4.62E-04 |
| BMX | 0.453 | Upregulated | 2.65E-04 | 6.36E-03 |
| IGF2BP3 | 0.453 | Upregulated | 6.39E-04 | 1.16E-02 |
| VNN3 | 0.453 | Upregulated | 3.57E-06 | 3.23E-04 |
| REM2 | 0.454 | Upregulated | 4.94E-06 | 4.03E-04 |
| PTAFR | 0.454 | Upregulated | 1.36E-04 | 4.01E-03 |
| TBC1D8 | 0.454 | Upregulated | 1.71E-05 | 9.55E-04 |
| RNF19B | 0.457 | Upregulated | 3.73E-07 | 6.60E-05 |
| ERLIN1 | 0.458 | Upregulated | 9.70E-07 | 1.29E-04 |
| DENND3 | 0.460 | Upregulated | 1.22E-06 | 1.50E-04 |
| GPR141 | 0.460 | Upregulated | 3.81E-04 | 8.15E-03 |
| IRAK3 | 0.460 | Upregulated | 6.17E-04 | 1.13E-02 |
| SAMD9L | 0.461 | Upregulated | 6.54E-05 | 2.41E-03 |
| NTNG2 | 0.462 | Upregulated | 1.18E-04 | 3.64E-03 |
| CPD | 0.462 | Upregulated | 4.80E-06 | 3.93E-04 |
| ACSL1 | 0.462 | Upregulated | 2.00E-03 | 2.54E-02 |
| PILRA | 0.463 | Upregulated | 4.54E-05 | 1.88E-03 |
| TMEM149 | 0.464 | Upregulated | 1.29E-09 | 1.09E-06 |
| AIM2 | 0.464 | Upregulated | 8.45E-07 | 1.17E-04 |
| C11ORF75 | 0.464 | Upregulated | 6.45E-08 | 1.78E-05 |
| TECPR2 | 0.465 | Upregulated | 3.31E-04 | 7.43E-03 |

| | | | | |
|---|---|---|---|---|
| PANX2 | 0.466 | Upregulated | 4.46E-04 | 9.16E-03 |
| MEFV | 0.467 | Upregulated | 1.08E-06 | 1.38E-04 |
| C1ORF38 | 0.468 | Upregulated | 2.23E-08 | 8.97E-06 |
| PLXNC1 | 0.469 | Upregulated | 1.44E-04 | 4.18E-03 |
| CEBPD | 0.469 | Upregulated | 1.67E-05 | 9.37E-04 |
| SLC16A3 | 0.470 | Upregulated | 2.24E-05 | 1.15E-03 |
| ST6GALNAC2 | 0.470 | Upregulated | 1.19E-05 | 7.30E-04 |
| AGTRAP | 0.471 | Upregulated | 7.18E-06 | 5.16E-04 |
| MEGF9 | 0.471 | Upregulated | 2.24E-05 | 1.15E-03 |
| HIST2H2BE | 0.472 | Upregulated | 6.61E-05 | 2.42E-03 |
| SLC22A15 | 0.472 | Upregulated | 5.50E-05 | 2.14E-03 |
| LRRC25 | 0.473 | Upregulated | 6.12E-06 | 4.70E-04 |
| GADD45B | 0.473 | Upregulated | 6.55E-09 | 3.52E-06 |
| PLXDC2 | 0.474 | Upregulated | 8.29E-06 | 5.73E-04 |
| TLE3 | 0.474 | Upregulated | 1.07E-05 | 6.74E-04 |
| ABCA1 | 0.475 | Upregulated | 2.32E-04 | 5.84E-03 |
| MSRB2 | 0.475 | Upregulated | 1.05E-07 | 2.56E-05 |
| GADD45G | 0.475 | Upregulated | 1.02E-07 | 2.50E-05 |
| TREML2 | 0.475 | Upregulated | 1.87E-08 | 8.10E-06 |
| PSG9 | 0.476 | Upregulated | 8.59E-05 | 2.91E-03 |
| DMXL2 | 0.477 | Upregulated | 2.48E-08 | 9.47E-06 |
| ETS2 | 0.477 | Upregulated | 4.67E-05 | 1.92E-03 |
| BST1 | 0.478 | Upregulated | 1.20E-04 | 3.67E-03 |
| ZAK | 0.478 | Upregulated | 2.94E-05 | 1.40E-03 |
| DDX60L | 0.479 | Upregulated | 1.30E-05 | 7.78E-04 |
| CECR6 | 0.479 | Upregulated | 2.67E-05 | 1.31E-03 |
| IRF1 | 0.481 | Upregulated | 5.05E-11 | 8.76E-08 |
| ERV3 | 0.481 | Upregulated | 6.43E-06 | 4.83E-04 |
| GCH1 | 0.483 | Upregulated | 4.52E-06 | 3.79E-04 |
| RNF149 | 0.484 | Upregulated | 1.55E-07 | 3.36E-05 |
| C1ORF138 | 0.484 | Upregulated | 2.40E-07 | 4.69E-05 |
| TNFRSF10B | 0.485 | Upregulated | 9.14E-05 | 3.02E-03 |
| CYP1B1 | 0.486 | Upregulated | 1.42E-02 | 9.80E-02 |
| LTBR | 0.487 | Upregulated | 2.01E-06 | 2.15E-04 |
| BCL3 | 0.488 | Upregulated | 4.33E-06 | 3.68E-04 |
| ZNF200 | 0.491 | Upregulated | 1.12E-10 | 1.76E-07 |
| SORL1 | 0.491 | Upregulated | 5.06E-05 | 2.01E-03 |
| FRAT2 | 0.494 | Upregulated | 6.14E-07 | 9.38E-05 |
| TMEM88 | 0.495 | Upregulated | 2.55E-05 | 1.27E-03 |
| HIST1H2BE | 0.495 | Upregulated | 1.38E-05 | 8.13E-04 |
| PLAUR | 0.496 | Upregulated | 5.30E-06 | 4.22E-04 |
| BRSK1 | 0.496 | Upregulated | 1.99E-06 | 2.13E-04 |
| HIST2H2AC | 0.497 | Upregulated | 8.88E-05 | 2.96E-03 |
| APOL1 | 0.499 | Upregulated | 1.20E-12 | 3.79E-09 |
| GNAQ | 0.501 | Upregulated | 1.02E-05 | 6.48E-04 |
| CLEC4E | 0.502 | Upregulated | 8.89E-06 | 6.01E-04 |
| ARL11 | 0.502 | Upregulated | 4.19E-06 | 3.58E-04 |
| SIRPA | 0.503 | Upregulated | 5.36E-05 | 2.10E-03 |
| SOCS3 | 0.504 | Upregulated | 6.67E-07 | 9.89E-05 |
| SIRPB1 | 0.505 | Upregulated | 3.91E-05 | 1.70E-03 |
| CRISPLD2 | 0.507 | Upregulated | 6.85E-04 | 1.22E-02 |

| | | | | |
|---|---|---|---|---|
| FCAR | 0.508 | Upregulated | 9.10E-05 | 3.01E-03 |
| SLC31A2 | 0.509 | Upregulated | 6.72E-08 | 1.82E-05 |
| EPSTI1 | 0.509 | Upregulated | 7.34E-05 | 2.61E-03 |
| TREM1 | 0.510 | Upregulated | 3.22E-04 | 7.27E-03 |
| GPR160 | 0.511 | Upregulated | 7.82E-05 | 2.72E-03 |
| CD300A | 0.511 | Upregulated | 2.33E-09 | 1.72E-06 |
| HIST1H4H | 0.511 | Upregulated | 1.25E-04 | 3.78E-03 |
| TNFAIP6 | 0.511 | Upregulated | 5.32E-10 | 6.36E-07 |
| CLEC4D | 0.513 | Upregulated | 4.86E-04 | 9.74E-03 |
| TCN2 | 0.513 | Upregulated | 3.81E-06 | 3.39E-04 |
| TIMP2 | 0.514 | Upregulated | 6.14E-06 | 4.70E-04 |
| FGL2 | 0.515 | Upregulated | 5.52E-10 | 6.39E-07 |
| NCF1 | 0.515 | Upregulated | 1.31E-07 | 2.96E-05 |
| FLVCR2 | 0.515 | Upregulated | 2.07E-08 | 8.72E-06 |
| CD97 | 0.516 | Upregulated | 5.13E-08 | 1.53E-05 |
| GBP4 | 0.517 | Upregulated | 4.60E-06 | 3.83E-04 |
| NCF4 | 0.518 | Upregulated | 5.11E-06 | 4.11E-04 |
| MAML3 | 0.518 | Upregulated | 4.68E-07 | 7.80E-05 |
| BASP1 | 0.521 | Upregulated | 2.18E-05 | 1.13E-03 |
| HIST2H2AA3 | 0.522 | Upregulated | 4.48E-05 | 1.87E-03 |
| POLB | 0.523 | Upregulated | 1.75E-16 | 6.07E-12 |
| SLC26A8 | 0.524 | Upregulated | 1.01E-05 | 6.44E-04 |
| ZDHHC18 | 0.525 | Upregulated | 8.27E-08 | 2.11E-05 |
| MBOAT7 | 0.525 | Upregulated | 6.12E-05 | 2.29E-03 |
| VNN2 | 0.525 | Upregulated | 2.60E-04 | 6.30E-03 |
| MAPK14 | 0.527 | Upregulated | 2.30E-05 | 1.16E-03 |
| SERPINA1 | 0.528 | Upregulated | 4.40E-06 | 3.73E-04 |
| C1RL | 0.529 | Upregulated | 5.73E-07 | 8.92E-05 |
| TLR5 | 0.530 | Upregulated | 2.88E-04 | 6.70E-03 |
| LMNB1 | 0.530 | Upregulated | 8.86E-07 | 1.21E-04 |
| GBP2 | 0.530 | Upregulated | 1.85E-12 | 4.95E-09 |
| CLEC5A | 0.531 | Upregulated | 7.58E-04 | 1.31E-02 |
| MCTP2 | 0.533 | Upregulated | 9.66E-06 | 6.27E-04 |
| DKFZP761E198 | 0.533 | Upregulated | 3.76E-08 | 1.25E-05 |
| RALB | 0.534 | Upregulated | 2.46E-08 | 9.47E-06 |
| FCER1G | 0.537 | Upregulated | 1.36E-06 | 1.65E-04 |
| TNFSF10 | 0.537 | Upregulated | 5.10E-09 | 3.00E-06 |
| MMRN1 | 0.538 | Upregulated | 4.16E-04 | 8.74E-03 |
| ETV7 | 0.538 | Upregulated | 5.84E-07 | 8.99E-05 |
| TMEM119 | 0.539 | Upregulated | 7.64E-03 | 6.39E-02 |
| MYL9 | 0.540 | Upregulated | 1.17E-03 | 1.74E-02 |
| HIST2H2AA4 | 0.541 | Upregulated | 1.45E-05 | 8.39E-04 |
| SLAMF8 | 0.541 | Upregulated | 1.26E-07 | 2.90E-05 |
| SLC6A6 | 0.541 | Upregulated | 2.35E-07 | 4.60E-05 |
| CCR1 | 0.542 | Upregulated | 2.25E-05 | 1.15E-03 |
| TLR6 | 0.543 | Upregulated | 1.93E-09 | 1.52E-06 |
| GNG10 | 0.544 | Upregulated | 1.51E-06 | 1.78E-04 |
| P2RY14 | 0.545 | Upregulated | 1.49E-08 | 6.78E-06 |
| PLSCR1 | 0.547 | Upregulated | 8.55E-05 | 2.90E-03 |
| SEPX1 | 0.547 | Upregulated | 4.74E-07 | 7.80E-05 |
| SCO2 | 0.548 | Upregulated | 4.74E-08 | 1.47E-05 |

| | | | | |
|---|---|---|---|---|
| DOCK5 | 0.548 | Upregulated | 1.72E-06 | 1.92E-04 |
| JUNB | 0.548 | Upregulated | 1.98E-08 | 8.47E-06 |
| FRAT1 | 0.551 | Upregulated | 4.43E-08 | 1.39E-05 |
| FCGR3B | 0.551 | Upregulated | 1.30E-05 | 7.79E-04 |
| LILRA5 | 0.553 | Upregulated | 5.42E-04 | 1.05E-02 |
| PGS1 | 0.554 | Upregulated | 1.54E-06 | 1.80E-04 |
| GPR84 | 0.556 | Upregulated | 2.65E-03 | 3.10E-02 |
| EMR2 | 0.556 | Upregulated | 6.02E-05 | 2.27E-03 |
| SBNO2 | 0.557 | Upregulated | 3.41E-08 | 1.16E-05 |
| DUSP1 | 0.558 | Upregulated | 1.12E-06 | 1.41E-04 |
| C1ORF24 | 0.558 | Upregulated | 1.52E-05 | 8.69E-04 |
| VAMP5 | 0.558 | Upregulated | 2.30E-09 | 1.72E-06 |
| C19ORF59 | 0.560 | Upregulated | 2.01E-02 | 1.24E-01 |
| BCL6 | 0.560 | Upregulated | 3.08E-05 | 1.44E-03 |
| DUSP3 | 0.561 | Upregulated | 1.48E-09 | 1.22E-06 |
| IL1RAP | 0.561 | Upregulated | 9.55E-07 | 1.28E-04 |
| RSAD2 | 0.562 | Upregulated | 1.63E-02 | 1.08E-01 |
| FLJ20273 | 0.562 | Upregulated | 2.89E-05 | 1.39E-03 |
| FBXO6 | 0.564 | Upregulated | 2.78E-06 | 2.68E-04 |
| EGLN1 | 0.564 | Upregulated | 2.78E-06 | 2.68E-04 |
| PTGS2 | 0.566 | Upregulated | 1.44E-05 | 8.37E-04 |
| FOLR3 | 0.567 | Upregulated | 1.21E-02 | 8.82E-02 |
| TIMM10 | 0.568 | Upregulated | 2.41E-06 | 2.44E-04 |
| CXCL10 | 0.569 | Upregulated | 1.17E-03 | 1.75E-02 |
| ROPN1L | 0.570 | Upregulated | 1.58E-05 | 8.95E-04 |
| IL13RA1 | 0.571 | Upregulated | 5.80E-08 | 1.67E-05 |
| IFITM3 | 0.571 | Upregulated | 8.41E-06 | 5.79E-04 |
| MXD1 | 0.571 | Upregulated | 6.67E-08 | 1.82E-05 |
| ALDH1A1 | 0.571 | Upregulated | 9.82E-07 | 1.30E-04 |
| MYBPC3 | 0.575 | Upregulated | 2.45E-07 | 4.76E-05 |
| RTP4 | 0.578 | Upregulated | 2.20E-06 | 2.29E-04 |
| RAB24 | 0.578 | Upregulated | 1.42E-11 | 2.89E-08 |
| NLRC4 | 0.580 | Upregulated | 6.94E-06 | 5.05E-04 |
| DSC2 | 0.583 | Upregulated | 1.94E-05 | 1.04E-03 |
| ADCY3 | 0.584 | Upregulated | 8.70E-06 | 5.93E-04 |
| HIST1H2BC | 0.584 | Upregulated | 1.57E-06 | 1.83E-04 |
| FAM129A | 0.584 | Upregulated | 9.12E-06 | 6.09E-04 |
| CEACAM3 | 0.584 | Upregulated | 1.61E-04 | 4.53E-03 |
| LIN7A | 0.586 | Upregulated | 2.10E-05 | 1.10E-03 |
| STEAP4 | 0.587 | Upregulated | 8.90E-05 | 2.96E-03 |
| LRRK2 | 0.589 | Upregulated | 5.16E-07 | 8.29E-05 |
| CR1 | 0.590 | Upregulated | 8.54E-05 | 2.90E-03 |
| RBM47 | 0.591 | Upregulated | 2.25E-08 | 8.97E-06 |
| TLR4 | 0.591 | Upregulated | 7.70E-07 | 1.09E-04 |
| RAB20 | 0.594 | Upregulated | 3.02E-08 | 1.09E-05 |
| SLC6A12 | 0.596 | Upregulated | 2.35E-08 | 9.27E-06 |
| FOS | 0.597 | Upregulated | 9.22E-07 | 1.24E-04 |
| TNFAIP2 | 0.601 | Upregulated | 2.40E-08 | 9.36E-06 |
| HCG27 | 0.603 | Upregulated | 4.45E-10 | 5.72E-07 |
| ST3GAL4 | 0.604 | Upregulated | 1.59E-07 | 3.42E-05 |
| TIFA | 0.606 | Upregulated | 1.54E-08 | 6.92E-06 |

| | | | | |
|---|---|---|---|---|
| PROK2 | 0.606 | Upregulated | 6.63E-05 | 2.42E-03 |
| STK3 | 0.606 | Upregulated | 2.13E-09 | 1.64E-06 |
| LHFPL2 | 0.607 | Upregulated | 6.02E-08 | 1.71E-05 |
| IFIT3 | 0.609 | Upregulated | 1.74E-03 | 2.30E-02 |
| HSPA6 | 0.610 | Upregulated | 6.70E-09 | 3.52E-06 |
| LY96 | 0.610 | Upregulated | 2.51E-04 | 6.16E-03 |
| RNF24 | 0.611 | Upregulated | 7.35E-07 | 1.06E-04 |
| SOCS1 | 0.613 | Upregulated | 9.30E-07 | 1.25E-04 |
| TPST1 | 0.613 | Upregulated | 2.62E-04 | 6.32E-03 |
| ASPRV1 | 0.614 | Upregulated | 5.68E-07 | 8.92E-05 |
| CSF2RB | 0.614 | Upregulated | 6.08E-09 | 3.46E-06 |
| TNFSF13B | 0.617 | Upregulated | 1.13E-08 | 5.35E-06 |
| ALPK1 | 0.619 | Upregulated | 4.05E-08 | 1.31E-05 |
| TMEM140 | 0.622 | Upregulated | 2.27E-07 | 4.54E-05 |
| TGFA | 0.628 | Upregulated | 2.19E-06 | 2.28E-04 |
| NOD2 | 0.630 | Upregulated | 1.45E-08 | 6.70E-06 |
| RFX2 | 0.632 | Upregulated | 1.47E-06 | 1.75E-04 |
| DYSF | 0.632 | Upregulated | 3.72E-05 | 1.64E-03 |
| STX11 | 0.632 | Upregulated | 2.76E-09 | 1.92E-06 |
| CSF3R | 0.634 | Upregulated | 1.52E-06 | 1.79E-04 |
| AQP9 | 0.634 | Upregulated | 1.42E-06 | 1.71E-04 |
| STX3 | 0.635 | Upregulated | 1.42E-07 | 3.16E-05 |
| FCGR2A | 0.635 | Upregulated | 1.22E-07 | 2.87E-05 |
| FPR1 | 0.636 | Upregulated | 4.97E-06 | 4.03E-04 |
| IL27 | 0.638 | Upregulated | 5.71E-08 | 1.67E-05 |
| SERPING1 | 0.639 | Upregulated | 1.96E-10 | 2.84E-07 |
| IL18RAP | 0.639 | Upregulated | 2.15E-06 | 2.25E-04 |
| IL1RN | 0.641 | Upregulated | 6.08E-05 | 2.29E-03 |
| TLR8 | 0.642 | Upregulated | 2.08E-06 | 2.20E-04 |
| IGSF6 | 0.642 | Upregulated | 4.42E-08 | 1.39E-05 |
| GRAMD1B | 0.642 | Upregulated | 2.32E-10 | 3.22E-07 |
| LAP3 | 0.643 | Upregulated | 1.52E-07 | 3.36E-05 |
| SORT1 | 0.646 | Upregulated | 3.57E-07 | 6.42E-05 |
| ZMYND15 | 0.653 | Upregulated | 4.92E-07 | 8.06E-05 |
| APOL6 | 0.653 | Upregulated | 1.26E-11 | 2.74E-08 |
| IFIT2 | 0.658 | Upregulated | 1.95E-05 | 1.04E-03 |
| OSM | 0.661 | Upregulated | 6.95E-07 | 1.02E-04 |
| C2 | 0.665 | Upregulated | 1.24E-09 | 1.08E-06 |
| BEST1 | 0.666 | Upregulated | 2.59E-09 | 1.84E-06 |
| WARS | 0.671 | Upregulated | 6.90E-09 | 3.57E-06 |
| ALPL | 0.675 | Upregulated | 7.45E-04 | 1.29E-02 |
| F2RL1 | 0.676 | Upregulated | 3.11E-08 | 1.11E-05 |
| ZDHHC19 | 0.676 | Upregulated | 1.34E-02 | 9.44E-02 |
| ANXA3 | 0.681 | Upregulated | 9.15E-04 | 1.49E-02 |
| PDCD1LG2 | 0.681 | Upregulated | 8.61E-09 | 4.33E-06 |
| TLR2 | 0.684 | Upregulated | 2.25E-06 | 2.31E-04 |
| LRG1 | 0.686 | Upregulated | 4.51E-05 | 1.88E-03 |
| ZNF438 | 0.688 | Upregulated | 3.68E-08 | 1.24E-05 |
| DHRS9 | 0.695 | Upregulated | 1.76E-09 | 1.42E-06 |
| IL8RB | 0.708 | Upregulated | 3.07E-06 | 2.91E-04 |
| INDO | 0.708 | Upregulated | 3.38E-04 | 7.52E-03 |

| | | | | |
|---|---|---|---|---|
| C4BPA | 0.709 | Upregulated | 8.05E-03 | 6.59E-02 |
| IL18R1 | 0.711 | Upregulated | 3.25E-05 | 1.49E-03 |
| PSTPIP2 | 0.712 | Upregulated | 6.48E-09 | 3.52E-06 |
| P2RY13 | 0.720 | Upregulated | 3.87E-08 | 1.28E-05 |
| LIMK2 | 0.724 | Upregulated | 3.18E-09 | 2.07E-06 |
| SOD2 | 0.729 | Upregulated | 4.01E-08 | 1.31E-05 |
| C16ORF7 | 0.734 | Upregulated | 3.47E-11 | 6.34E-08 |
| KREMEN1 | 0.737 | Upregulated | 2.54E-06 | 2.55E-04 |
| GPR97 | 0.742 | Upregulated | 3.86E-07 | 6.69E-05 |
| NAMPT | 0.744 | Upregulated | 3.63E-06 | 3.28E-04 |
| CXCR1 | 0.746 | Upregulated | 8.91E-07 | 1.21E-04 |
| ADM | 0.750 | Upregulated | 2.14E-07 | 4.40E-05 |
| SIPA1L2 | 0.759 | Upregulated | 1.59E-06 | 1.83E-04 |
| FPR2 | 0.759 | Upregulated | 4.71E-07 | 7.80E-05 |
| KCNJ2 | 0.779 | Upregulated | 3.19E-09 | 2.07E-06 |
| FFAR2 | 0.780 | Upregulated | 2.53E-08 | 9.52E-06 |
| C1QC | 0.782 | Upregulated | 8.16E-05 | 2.80E-03 |
| LPCAT2 | 0.786 | Upregulated | 2.29E-07 | 4.54E-05 |
| GPR109B | 0.803 | Upregulated | 5.94E-10 | 6.44E-07 |
| SMARCD3 | 0.817 | Upregulated | 4.31E-08 | 1.37E-05 |
| KCNJ15 | 0.846 | Upregulated | 6.67E-09 | 3.52E-06 |
| FER1L3 | 0.846 | Upregulated | 1.46E-10 | 2.21E-07 |
| C1QB | 0.854 | Upregulated | 5.23E-05 | 2.06E-03 |
| IDO1 | 0.859 | Upregulated | 1.16E-05 | 7.12E-04 |
| GBP6 | 0.864 | Upregulated | 6.52E-15 | 7.54E-11 |
| IL1B | 0.886 | Upregulated | 6.56E-10 | 6.90E-07 |
| GBP1 | 0.887 | Upregulated | 5.76E-10 | 6.44E-07 |
| CEACAM1 | 0.918 | Upregulated | 2.24E-08 | 8.97E-06 |
| MYOF | 0.924 | Upregulated | 2.85E-11 | 5.50E-08 |
| GPR109A | 0.930 | Upregulated | 5.18E-10 | 6.36E-07 |
| CACNA1E | 0.940 | Upregulated | 8.45E-09 | 4.31E-06 |
| CARD17 | 1.020 | Upregulated | 9.08E-12 | 2.10E-08 |
| GBP5 | 1.033 | Upregulated | 3.25E-13 | 1.25E-09 |
| GK | 1.053 | Upregulated | 3.12E-13 | 1.25E-09 |
| CD274 | 1.056 | Upregulated | 7.33E-13 | 2.54E-09 |
| FCGR1B | 1.171 | Upregulated | 1.66E-12 | 4.81E-09 |
| FCGR1C | 1.204 | Upregulated | 1.67E-13 | 8.30E-10 |
| CASP5 | 1.224 | Upregulated | 3.15E-10 | 4.20E-07 |
| BATF2 | 1.303 | Upregulated | 4.23E-15 | 7.33E-11 |
| ANKRD22 | 1.351 | Upregulated | 2.72E-12 | 6.75E-09 |
| FCGR1A | 1.374 | Upregulated | 1.92E-14 | 1.33E-10 |

**Supplementary Table S2b. Differentially expressed genes_South Africa**

| Gene | logFC | Direction of expression | P.Value | adj.P.Val |
|---|---|---|---|---|
| CDKN1C | -0.762 | Downregulated | 4.63E-11 | 6.78E-09 |
| TNFRSF13B | -0.735 | Downregulated | 4.49E-13 | 1.90E-10 |
| C20ORF103 | -0.730 | Downregulated | 7.75E-11 | 9.63E-09 |
| CDC20 | -0.675 | Downregulated | 1.60E-08 | 6.64E-07 |
| GLDC | -0.667 | Downregulated | 2.60E-07 | 6.50E-06 |
| CCNB2 | -0.633 | Downregulated | 1.52E-08 | 6.39E-07 |
| TNFRSF17 | -0.621 | Downregulated | 2.73E-05 | 3.03E-04 |
| AURKB | -0.580 | Downregulated | 2.46E-09 | 1.52E-07 |
| KIAA0101 | -0.572 | Downregulated | 2.38E-06 | 3.96E-05 |
| TXNDC5 | -0.558 | Downregulated | 7.56E-06 | 1.05E-04 |
| EIF2B5 | -0.541 | Downregulated | 7.24E-12 | 1.59E-09 |
| VPREB3 | -0.536 | Downregulated | 2.34E-05 | 2.67E-04 |
| MGC29506 | -0.533 | Downregulated | 2.26E-06 | 3.79E-05 |
| TOP2A | -0.532 | Downregulated | 1.09E-06 | 2.07E-05 |
| ITM2C | -0.527 | Downregulated | 5.37E-08 | 1.81E-06 |
| CDC45L | -0.514 | Downregulated | 2.33E-06 | 3.89E-05 |
| CD79A | -0.510 | Downregulated | 5.62E-09 | 2.92E-07 |
| SCGB3A1 | -0.505 | Downregulated | 4.41E-04 | 3.12E-03 |
| HRK | -0.494 | Downregulated | 1.37E-07 | 3.89E-06 |
| IFI27 | -0.492 | Downregulated | 4.30E-02 | 1.29E-01 |
| ZNF573 | -0.492 | Downregulated | 1.90E-08 | 7.52E-07 |
| CRIP2 | -0.489 | Downregulated | 9.53E-09 | 4.40E-07 |
| ZBED2 | -0.480 | Downregulated | 2.11E-10 | 2.10E-08 |
| NCAPG | -0.479 | Downregulated | 7.20E-06 | 1.01E-04 |
| BUB1 | -0.478 | Downregulated | 2.04E-05 | 2.38E-04 |
| OSBPL10 | -0.475 | Downregulated | 2.60E-07 | 6.50E-06 |
| KIFC1 | -0.462 | Downregulated | 5.31E-08 | 1.80E-06 |
| MCM4 | -0.459 | Downregulated | 1.37E-08 | 5.95E-07 |
| CXCR3 | -0.458 | Downregulated | 1.17E-09 | 8.52E-08 |
| AURKA | -0.457 | Downregulated | 8.54E-09 | 4.06E-07 |
| CENPA | -0.453 | Downregulated | 1.35E-08 | 5.86E-07 |
| C19ORF48 | -0.453 | Downregulated | 9.51E-09 | 4.40E-07 |
| PACAP | -0.450 | Downregulated | 2.17E-08 | 8.40E-07 |
| MCM2 | -0.449 | Downregulated | 1.51E-07 | 4.26E-06 |
| PNOC | -0.449 | Downregulated | 3.44E-08 | 1.24E-06 |
| CHI3L2 | -0.448 | Downregulated | 7.93E-07 | 1.60E-05 |
| CHEK1 | -0.445 | Downregulated | 2.64E-09 | 1.61E-07 |
| C16ORF59 | -0.445 | Downregulated | 1.22E-09 | 8.77E-08 |
| LIMS2 | -0.444 | Downregulated | 1.58E-08 | 6.58E-07 |
| PDCD2L | -0.442 | Downregulated | 6.92E-12 | 1.55E-09 |
| CD52 | -0.440 | Downregulated | 4.59E-05 | 4.67E-04 |
| GPRC5D | -0.439 | Downregulated | 6.21E-07 | 1.31E-05 |
| CD19 | -0.436 | Downregulated | 7.53E-07 | 1.54E-05 |
| IGJ | -0.435 | Downregulated | 2.74E-03 | 1.43E-02 |
| FCRLA | -0.435 | Downregulated | 4.73E-06 | 7.07E-05 |
| TYMS | -0.434 | Downregulated | 1.42E-05 | 1.76E-04 |
| IL28RA | -0.433 | Downregulated | 1.37E-09 | 9.45E-08 |
| CDT1 | -0.433 | Downregulated | 3.24E-05 | 3.48E-04 |

| | | | | |
|---|---|---|---|---|
| CDCA5 | -0.433 | Downregulated | 5.66E-06 | 8.20E-05 |
| EBI2 | -0.432 | Downregulated | 4.10E-07 | 9.35E-06 |
| VIL2 | -0.425 | Downregulated | 1.38E-13 | 7.16E-11 |
| ANKRD55 | -0.425 | Downregulated | 1.61E-06 | 2.86E-05 |
| RFC3 | -0.424 | Downregulated | 1.35E-10 | 1.48E-08 |
| C6ORF125 | -0.424 | Downregulated | 1.90E-06 | 3.29E-05 |
| STOML2 | -0.423 | Downregulated | 1.55E-11 | 2.87E-09 |
| BCAR3 | -0.422 | Downregulated | 1.06E-06 | 2.03E-05 |
| CCNA2 | -0.418 | Downregulated | 4.81E-06 | 7.17E-05 |
| SDF2L1 | -0.418 | Downregulated | 4.33E-09 | 2.38E-07 |
| POU2AF1 | -0.417 | Downregulated | 5.09E-07 | 1.12E-05 |
| PARK7 | -0.417 | Downregulated | 8.40E-09 | 4.02E-07 |
| CEP55 | -0.416 | Downregulated | 8.65E-07 | 1.72E-05 |
| MCM7 | -0.416 | Downregulated | 2.97E-09 | 1.76E-07 |
| DLGAP5 | -0.415 | Downregulated | 1.08E-05 | 1.40E-04 |
| RRM2 | -0.415 | Downregulated | 9.09E-06 | 1.22E-04 |
| EVL | -0.413 | Downregulated | 1.74E-09 | 1.13E-07 |
| ABCB9 | -0.413 | Downregulated | 7.20E-05 | 6.79E-04 |
| GINS2 | -0.413 | Downregulated | 3.25E-06 | 5.16E-05 |
| CDKAL1 | -0.413 | Downregulated | 2.76E-15 | 2.84E-12 |
| TNFRSF13C | -0.411 | Downregulated | 4.92E-07 | 1.09E-05 |
| C16ORF33 | -0.411 | Downregulated | 5.91E-11 | 8.04E-09 |
| CKAP2L | -0.410 | Downregulated | 2.91E-06 | 4.72E-05 |
| CACNA2D3 | -0.410 | Downregulated | 1.13E-04 | 9.96E-04 |
| CCR9 | -0.409 | Downregulated | 2.86E-07 | 6.99E-06 |
| C11ORF80 | -0.409 | Downregulated | 1.76E-09 | 1.14E-07 |
| GSG2 | -0.408 | Downregulated | 6.08E-09 | 3.12E-07 |
| TK1 | -0.408 | Downregulated | 2.57E-06 | 4.24E-05 |
| ALDH18A1 | -0.407 | Downregulated | 2.34E-11 | 4.04E-09 |
| CDCA2 | -0.407 | Downregulated | 6.50E-09 | 3.28E-07 |
| EBF1 | -0.406 | Downregulated | 1.91E-05 | 2.24E-04 |
| TRIP13 | -0.406 | Downregulated | 9.71E-07 | 1.89E-05 |
| C1ORF77 | -0.406 | Downregulated | 8.31E-12 | 1.78E-09 |
| PBK | -0.404 | Downregulated | 3.29E-06 | 5.23E-05 |
| TIMD4 | -0.404 | Downregulated | 2.68E-04 | 2.05E-03 |
| QPRT | -0.403 | Downregulated | 1.04E-09 | 7.71E-08 |
| AGMAT | -0.402 | Downregulated | 1.98E-08 | 7.78E-07 |
| UFSP2 | -0.401 | Downregulated | 3.84E-12 | 9.73E-10 |
| GPR18 | -0.401 | Downregulated | 1.70E-07 | 4.68E-06 |
| PRC1 | -0.400 | Downregulated | 5.44E-08 | 1.83E-06 |
| C20ORF100 | -0.400 | Downregulated | 6.90E-09 | 3.43E-07 |
| CDKN3 | -0.398 | Downregulated | 1.02E-04 | 9.12E-04 |
| DENND5B | -0.395 | Downregulated | 1.41E-06 | 2.56E-05 |
| MELK | -0.393 | Downregulated | 6.63E-07 | 1.38E-05 |
| HSPC111 | -0.393 | Downregulated | 1.88E-09 | 1.21E-07 |
| HJURP | -0.393 | Downregulated | 3.38E-08 | 1.23E-06 |
| ID3 | -0.392 | Downregulated | 5.28E-06 | 7.75E-05 |
| STRBP | -0.391 | Downregulated | 5.48E-07 | 1.18E-05 |
| PHGDH | -0.391 | Downregulated | 5.26E-06 | 7.72E-05 |
| FAM3C | -0.391 | Downregulated | 4.40E-08 | 1.53E-06 |
| C22ORF28 | -0.390 | Downregulated | 1.95E-12 | 5.69E-10 |

| | | | | |
|---|---|---|---|---|
| ICOS | -0.390 | Downregulated | 6.66E-06 | 9.43E-05 |
| CDCA3 | -0.389 | Downregulated | 4.70E-07 | 1.05E-05 |
| RFC4 | -0.387 | Downregulated | 3.37E-09 | 1.96E-07 |
| PAICS | -0.387 | Downregulated | 7.20E-10 | 5.86E-08 |
| VPREB1 | -0.387 | Downregulated | 1.90E-03 | 1.06E-02 |
| PACSIN1 | -0.386 | Downregulated | 7.49E-06 | 1.04E-04 |
| FLJ11795 | -0.385 | Downregulated | 5.71E-06 | 8.26E-05 |
| WDR92 | -0.385 | Downregulated | 2.60E-12 | 7.17E-10 |
| NLRP2 | -0.385 | Downregulated | 8.36E-07 | 1.68E-05 |
| CKS1B | -0.385 | Downregulated | 7.79E-07 | 1.58E-05 |
| KIAA0125 | -0.384 | Downregulated | 3.27E-07 | 7.81E-06 |
| NTHL1 | -0.384 | Downregulated | 9.33E-12 | 1.90E-09 |
| SLC25A4 | -0.384 | Downregulated | 5.87E-08 | 1.94E-06 |
| LAG3 | -0.383 | Downregulated | 1.08E-03 | 6.58E-03 |
| GZMK | -0.382 | Downregulated | 3.84E-04 | 2.78E-03 |
| BHLHB3 | -0.382 | Downregulated | 6.17E-10 | 5.13E-08 |
| CRIP1 | -0.382 | Downregulated | 1.18E-11 | 2.29E-09 |
| EBI3 | -0.382 | Downregulated | 4.88E-06 | 7.25E-05 |
| HES4 | -0.382 | Downregulated | 2.11E-03 | 1.15E-02 |
| USP18 | -0.381 | Downregulated | 2.07E-02 | 7.33E-02 |
| ZNF260 | -0.379 | Downregulated | 2.69E-10 | 2.56E-08 |
| ASPM | -0.378 | Downregulated | 1.06E-05 | 1.38E-04 |
| MAP4K1 | -0.378 | Downregulated | 3.91E-09 | 2.19E-07 |
| CD27 | -0.377 | Downregulated | 9.43E-06 | 1.25E-04 |
| CHST12 | -0.377 | Downregulated | 8.04E-07 | 1.62E-05 |
| FAM83D | -0.375 | Downregulated | 8.48E-09 | 4.04E-07 |
| HIBCH | -0.374 | Downregulated | 1.72E-12 | 5.15E-10 |
| SLC16A10 | -0.373 | Downregulated | 2.48E-04 | 1.93E-03 |
| ADA | -0.373 | Downregulated | 3.89E-09 | 2.18E-07 |
| CCDC134 | -0.373 | Downregulated | 3.83E-16 | 6.99E-13 |
| HDAC1 | -0.372 | Downregulated | 1.48E-14 | 1.28E-11 |
| CD38 | -0.372 | Downregulated | 1.69E-04 | 1.40E-03 |
| CLDND1 | -0.370 | Downregulated | 8.77E-10 | 6.79E-08 |
| SIRPG | -0.369 | Downregulated | 1.42E-05 | 1.76E-04 |
| NT5DC2 | -0.368 | Downregulated | 1.15E-05 | 1.48E-04 |
| LRRN3 | -0.367 | Downregulated | 2.79E-03 | 1.46E-02 |
| C9ORF37 | -0.367 | Downregulated | 9.63E-08 | 2.91E-06 |
| KIF4A | -0.367 | Downregulated | 6.26E-07 | 1.32E-05 |
| C6ORF129 | -0.366 | Downregulated | 3.62E-06 | 5.66E-05 |
| CCDC99 | -0.366 | Downregulated | 2.07E-07 | 5.48E-06 |
| ZNF593 | -0.366 | Downregulated | 9.72E-11 | 1.15E-08 |
| E2F5 | -0.365 | Downregulated | 1.61E-06 | 2.87E-05 |
| TPX2 | -0.365 | Downregulated | 7.03E-07 | 1.45E-05 |
| TMEM14A | -0.365 | Downregulated | 6.96E-07 | 1.44E-05 |
| C16ORF74 | -0.364 | Downregulated | 4.67E-09 | 2.52E-07 |
| C19ORF10 | -0.364 | Downregulated | 1.65E-08 | 6.82E-07 |
| CCNF | -0.363 | Downregulated | 1.12E-07 | 3.30E-06 |
| CDC25C | -0.363 | Downregulated | 2.10E-08 | 8.19E-07 |
| HERC2 | -0.363 | Downregulated | 4.82E-09 | 2.57E-07 |
| THOC3 | -0.360 | Downregulated | 4.86E-07 | 1.08E-05 |
| FAIM3 | -0.360 | Downregulated | 6.34E-07 | 1.33E-05 |

| | | | | |
|---|---|---|---|---|
| CENPM | -0.360 | Downregulated | 9.19E-07 | 1.80E-05 |
| PPP3CC | -0.358 | Downregulated | 1.36E-08 | 5.89E-07 |
| POLA1 | -0.357 | Downregulated | 7.04E-11 | 9.08E-09 |
| DNAJC9 | -0.357 | Downregulated | 8.36E-09 | 4.01E-07 |
| SPIB | -0.357 | Downregulated | 3.92E-05 | 4.09E-04 |
| CHAF1B | -0.356 | Downregulated | 6.60E-08 | 2.12E-06 |
| KIF20A | -0.355 | Downregulated | 2.57E-06 | 4.24E-05 |
| BIRC5 | -0.355 | Downregulated | 8.14E-08 | 2.53E-06 |
| PDCD1 | -0.354 | Downregulated | 5.52E-05 | 5.44E-04 |
| TUBB3 | -0.353 | Downregulated | 1.44E-08 | 6.19E-07 |
| PDK4 | -0.352 | Downregulated | 8.21E-04 | 5.25E-03 |
| CDCA4 | -0.352 | Downregulated | 1.29E-09 | 9.01E-08 |
| STAP1 | -0.351 | Downregulated | 1.28E-04 | 1.11E-03 |
| PMM1 | -0.351 | Downregulated | 1.50E-05 | 1.84E-04 |
| TEX10 | -0.350 | Downregulated | 4.68E-14 | 3.18E-11 |
| REXO4 | -0.350 | Downregulated | 1.09E-11 | 2.15E-09 |
| MCM10 | -0.350 | Downregulated | 1.70E-06 | 2.99E-05 |
| TIMELESS | -0.350 | Downregulated | 2.03E-08 | 7.93E-07 |
| APOBEC3B | -0.350 | Downregulated | 6.71E-04 | 4.44E-03 |
| BCAS4 | -0.349 | Downregulated | 1.74E-07 | 4.76E-06 |
| CLECL1 | -0.349 | Downregulated | 3.04E-03 | 1.56E-02 |
| PTTG1 | -0.349 | Downregulated | 9.91E-07 | 1.92E-05 |
| CTPS | -0.349 | Downregulated | 8.53E-09 | 4.06E-07 |
| TUBB | -0.349 | Downregulated | 1.62E-11 | 3.00E-09 |
| PRKAR1B | -0.348 | Downregulated | 5.31E-09 | 2.78E-07 |
| C10ORF6 | -0.348 | Downregulated | 2.51E-14 | 1.90E-11 |
| CD72 | -0.348 | Downregulated | 4.95E-06 | 7.33E-05 |
| NUBP2 | -0.348 | Downregulated | 2.11E-11 | 3.71E-09 |
| GNG3 | -0.348 | Downregulated | 5.72E-11 | 7.87E-09 |
| SEL1L3 | -0.348 | Downregulated | 3.02E-07 | 7.31E-06 |
| KIF20B | -0.348 | Downregulated | 5.75E-07 | 1.23E-05 |
| C18ORF56 | -0.347 | Downregulated | 1.98E-07 | 5.28E-06 |
| PTDSS1 | -0.346 | Downregulated | 3.17E-12 | 8.41E-10 |
| PARM1 | -0.346 | Downregulated | 2.01E-06 | 3.43E-05 |
| MRPL12 | -0.345 | Downregulated | 1.42E-10 | 1.55E-08 |
| HESX1 | -0.345 | Downregulated | 8.76E-03 | 3.69E-02 |
| HMMR | -0.345 | Downregulated | 1.45E-04 | 1.23E-03 |
| GALR2 | -0.345 | Downregulated | 2.07E-07 | 5.48E-06 |
| CHCHD6 | -0.345 | Downregulated | 3.54E-09 | 2.04E-07 |
| C1ORF135 | -0.344 | Downregulated | 1.11E-09 | 8.18E-08 |
| MYOM2 | -0.344 | Downregulated | 4.73E-02 | 1.39E-01 |
| CD320 | -0.344 | Downregulated | 2.13E-07 | 5.59E-06 |
| MRPS26 | -0.343 | Downregulated | 1.66E-11 | 3.06E-09 |
| SLAMF1 | -0.343 | Downregulated | 4.05E-07 | 9.28E-06 |
| ADAM23 | -0.343 | Downregulated | 8.93E-06 | 1.20E-04 |
| MSC | -0.343 | Downregulated | 5.12E-04 | 3.55E-03 |
| SCG5 | -0.342 | Downregulated | 9.49E-13 | 3.33E-10 |
| MEI1 | -0.342 | Downregulated | 3.89E-11 | 5.87E-09 |
| IMPDH2 | -0.341 | Downregulated | 1.95E-08 | 7.67E-07 |
| CTLA4 | -0.340 | Downregulated | 1.37E-06 | 2.50E-05 |
| WDR54 | -0.340 | Downregulated | 2.13E-08 | 8.26E-07 |

| | | | | |
|---|---|---|---|---|
| SIGIRR | -0.340 | Downregulated | 2.13E-10 | 2.12E-08 |
| CELSR3 | -0.338 | Downregulated | 3.03E-06 | 4.87E-05 |
| MTSS1 | -0.338 | Downregulated | 1.73E-08 | 7.03E-07 |
| UCK2 | -0.338 | Downregulated | 3.32E-09 | 1.94E-07 |
| OLIG1 | -0.338 | Downregulated | 3.31E-04 | 2.45E-03 |
| CXORF64 | -0.337 | Downregulated | 3.64E-10 | 3.27E-08 |
| CCNE1 | -0.337 | Downregulated | 5.10E-09 | 2.70E-07 |
| MCM6 | -0.337 | Downregulated | 7.12E-07 | 1.46E-05 |
| AHI1 | -0.336 | Downregulated | 8.85E-06 | 1.19E-04 |
| ARMET | -0.336 | Downregulated | 6.96E-07 | 1.44E-05 |
| TECR | -0.336 | Downregulated | 8.18E-11 | 9.99E-09 |
| POLR2D | -0.335 | Downregulated | 6.07E-17 | 1.87E-13 |
| GRWD1 | -0.334 | Downregulated | 6.56E-12 | 1.48E-09 |
| ALS2CR4 | -0.333 | Downregulated | 5.34E-08 | 1.80E-06 |
| EPPB9 | -0.332 | Downregulated | 1.71E-07 | 4.69E-06 |
| ELP4 | -0.332 | Downregulated | 3.80E-10 | 3.40E-08 |
| GTSF1L | -0.332 | Downregulated | 1.24E-09 | 8.85E-08 |
| COBLL1 | -0.331 | Downregulated | 1.62E-04 | 1.35E-03 |
| NDUFAF1 | -0.331 | Downregulated | 1.42E-07 | 4.03E-06 |
| MRPL2 | -0.331 | Downregulated | 6.96E-12 | 1.55E-09 |
| LAS1L | -0.331 | Downregulated | 2.07E-12 | 5.93E-10 |
| ZFP82 | -0.330 | Downregulated | 4.60E-08 | 1.59E-06 |
| LSM2 | -0.330 | Downregulated | 1.82E-14 | 1.54E-11 |
| HNRNPH1 | -0.329 | Downregulated | 1.76E-07 | 4.78E-06 |
| SNRPF | -0.328 | Downregulated | 7.00E-08 | 2.23E-06 |
| TCL1A | -0.327 | Downregulated | 2.03E-03 | 1.12E-02 |
| ZNF296 | -0.327 | Downregulated | 1.62E-10 | 1.71E-08 |
| BOLA3 | -0.326 | Downregulated | 1.47E-07 | 4.15E-06 |
| SLC38A1 | -0.326 | Downregulated | 1.81E-10 | 1.87E-08 |
| PWP1 | -0.326 | Downregulated | 7.35E-11 | 9.36E-09 |
| PPAN | -0.326 | Downregulated | 5.78E-09 | 3.00E-07 |
| EZH2 | -0.325 | Downregulated | 2.86E-07 | 6.99E-06 |
| APOD | -0.325 | Downregulated | 2.82E-05 | 3.11E-04 |
| MPP6 | -0.324 | Downregulated | 3.20E-06 | 5.08E-05 |
| BYSL | -0.324 | Downregulated | 1.04E-08 | 4.78E-07 |
| RAN | -0.324 | Downregulated | 1.40E-09 | 9.60E-08 |
| PARP1 | -0.324 | Downregulated | 2.27E-08 | 8.73E-07 |
| STK39 | -0.323 | Downregulated | 4.79E-06 | 7.15E-05 |
| LIMA1 | -0.323 | Downregulated | 6.34E-06 | 9.06E-05 |
| TMEM110 | -0.323 | Downregulated | 3.26E-10 | 2.96E-08 |
| COX11 | -0.323 | Downregulated | 1.35E-07 | 3.84E-06 |
| NCBP2 | -0.322 | Downregulated | 3.01E-12 | 8.15E-10 |
| ALG8 | -0.322 | Downregulated | 3.17E-12 | 8.41E-10 |
| TACO1 | -0.322 | Downregulated | 1.42E-06 | 2.58E-05 |
| CD79B | -0.322 | Downregulated | 9.19E-05 | 8.33E-04 |
| STT3B | -0.321 | Downregulated | 1.18E-08 | 5.26E-07 |
| TMEM118 | -0.321 | Downregulated | 2.78E-07 | 6.84E-06 |
| TULP4 | -0.320 | Downregulated | 6.60E-11 | 8.65E-09 |
| MOBKL2B | -0.320 | Downregulated | 1.10E-04 | 9.73E-04 |
| RAD51C | -0.320 | Downregulated | 3.30E-08 | 1.20E-06 |
| HRASLS2 | -0.319 | Downregulated | 1.78E-04 | 1.45E-03 |

| | | | | |
|---|---|---|---|---|
| TMEM156 | -0.318 | Downregulated | 1.21E-07 | 3.52E-06 |
| PSMB5 | -0.317 | Downregulated | 4.79E-12 | 1.15E-09 |
| GOT1 | -0.317 | Downregulated | 3.02E-10 | 2.79E-08 |
| CPNE5 | -0.316 | Downregulated | 6.74E-06 | 9.52E-05 |
| PVRIG | -0.316 | Downregulated | 4.85E-05 | 4.89E-04 |
| MMACHC | -0.316 | Downregulated | 1.28E-09 | 9.01E-08 |
| PASK | -0.316 | Downregulated | 4.33E-04 | 3.07E-03 |
| NPM3 | -0.315 | Downregulated | 1.01E-07 | 3.04E-06 |
| UCKL1 | -0.315 | Downregulated | 3.86E-14 | 2.68E-11 |
| CUTA | -0.315 | Downregulated | 2.77E-11 | 4.58E-09 |
| CXORF12 | -0.314 | Downregulated | 5.58E-07 | 1.20E-05 |
| STT3A | -0.314 | Downregulated | 8.87E-10 | 6.85E-08 |
| CKB | -0.314 | Downregulated | 1.94E-06 | 3.34E-05 |
| CDCA7L | -0.312 | Downregulated | 3.84E-11 | 5.85E-09 |
| POLR2I | -0.312 | Downregulated | 2.07E-13 | 1.03E-10 |
| C20ORF196 | -0.312 | Downregulated | 1.35E-13 | 7.16E-11 |
| SIRT4 | -0.312 | Downregulated | 1.82E-07 | 4.91E-06 |
| KRR1 | -0.312 | Downregulated | 3.75E-11 | 5.76E-09 |
| ATXN7L1 | -0.311 | Downregulated | 2.52E-07 | 6.36E-06 |
| GNB1L | -0.311 | Downregulated | 2.79E-15 | 2.84E-12 |
| THOC6 | -0.311 | Downregulated | 4.34E-09 | 2.38E-07 |
| COX5A | -0.311 | Downregulated | 1.22E-12 | 4.19E-10 |
| NUSAP1 | -0.310 | Downregulated | 1.26E-05 | 1.60E-04 |
| ZNF121 | -0.310 | Downregulated | 1.85E-06 | 3.21E-05 |
| PPIL1 | -0.310 | Downregulated | 6.12E-08 | 2.01E-06 |
| SFTPD | -0.310 | Downregulated | 3.52E-07 | 8.26E-06 |
| MRPS9 | -0.310 | Downregulated | 1.94E-10 | 1.96E-08 |
| AXIN2 | -0.309 | Downregulated | 1.21E-03 | 7.24E-03 |
| C21ORF33 | -0.309 | Downregulated | 5.24E-14 | 3.43E-11 |
| SRPRB | -0.309 | Downregulated | 4.22E-08 | 1.47E-06 |
| BLK | -0.308 | Downregulated | 1.72E-04 | 1.41E-03 |
| ZMYND19 | -0.308 | Downregulated | 1.04E-08 | 4.78E-07 |
| SAMM50 | -0.308 | Downregulated | 1.46E-11 | 2.76E-09 |
| HSZFP36 | -0.307 | Downregulated | 2.85E-06 | 4.63E-05 |
| PYHIN1 | -0.307 | Downregulated | 2.79E-04 | 2.13E-03 |
| PRMT7 | -0.307 | Downregulated | 4.00E-09 | 2.23E-07 |
| PLK4 | -0.306 | Downregulated | 6.04E-06 | 8.67E-05 |
| GINS3 | -0.306 | Downregulated | 2.02E-06 | 3.46E-05 |
| ZNF263 | -0.306 | Downregulated | 7.96E-08 | 2.49E-06 |
| TRIM47 | -0.306 | Downregulated | 4.72E-08 | 1.62E-06 |
| ZNF215 | -0.305 | Downregulated | 5.72E-07 | 1.23E-05 |
| LMNB2 | -0.304 | Downregulated | 2.33E-08 | 8.94E-07 |
| LIME1 | -0.304 | Downregulated | 4.14E-06 | 6.33E-05 |
| CLDND2 | -0.304 | Downregulated | 7.72E-04 | 4.98E-03 |
| NDUFS3 | -0.304 | Downregulated | 1.06E-09 | 7.83E-08 |
| RIOK2 | -0.303 | Downregulated | 3.94E-08 | 1.39E-06 |
| FLNB | -0.303 | Downregulated | 1.64E-06 | 2.91E-05 |
| CLEC4F | -0.303 | Downregulated | 1.70E-03 | 9.64E-03 |
| TRIB2 | -0.303 | Downregulated | 3.18E-05 | 3.43E-04 |
| EBP | -0.303 | Downregulated | 6.21E-09 | 3.16E-07 |
| ATAD2 | -0.303 | Downregulated | 2.45E-06 | 4.06E-05 |

| | | | | |
|---|---|---|---|---|
| C7ORF54 | -0.302 | Downregulated | 1.02E-12 | 3.54E-10 |
| EIF2A | -0.302 | Downregulated | 2.59E-06 | 4.26E-05 |
| DNAJB11 | -0.302 | Downregulated | 8.82E-09 | 4.16E-07 |
| UBE2T | -0.302 | Downregulated | 1.10E-04 | 9.69E-04 |
| C18ORF45 | -0.302 | Downregulated | 3.79E-05 | 3.98E-04 |
| WDR34 | -0.302 | Downregulated | 7.72E-08 | 2.42E-06 |
| GPR55 | -0.302 | Downregulated | 1.67E-08 | 6.89E-07 |
| ANKRD57 | -0.302 | Downregulated | 1.39E-05 | 1.73E-04 |
| RAB30 | -0.301 | Downregulated | 5.88E-06 | 8.47E-05 |
| GPT2 | -0.301 | Downregulated | 2.73E-09 | 1.65E-07 |
| KIFAP3 | -0.301 | Downregulated | 3.43E-06 | 5.42E-05 |
| C3ORF75 | -0.301 | Downregulated | 3.80E-09 | 2.15E-07 |
| CHAD | -0.301 | Downregulated | 4.67E-10 | 4.01E-08 |
| FAM167A | -0.301 | Downregulated | 2.11E-04 | 1.68E-03 |
| PTPRK | -0.301 | Downregulated | 1.08E-04 | 9.52E-04 |
| GPX7 | -0.301 | Downregulated | 3.77E-06 | 5.85E-05 |
| HNRNPA1 | -0.300 | Downregulated | 3.17E-11 | 5.07E-09 |
| GINS4 | -0.300 | Downregulated | 1.21E-09 | 8.75E-08 |
| PPA1 | -0.300 | Downregulated | 3.49E-06 | 5.48E-05 |
| C1ORF112 | -0.300 | Downregulated | 2.82E-08 | 1.05E-06 |
| HDDC2 | -0.300 | Downregulated | 6.17E-07 | 1.31E-05 |
| FAHD2B | -0.300 | Downregulated | 1.51E-09 | 1.01E-07 |
| IPO7 | -0.300 | Downregulated | 2.79E-07 | 6.86E-06 |
| RPLP0 | -0.299 | Downregulated | 7.03E-05 | 6.66E-04 |
| SCML4 | -0.299 | Downregulated | 8.03E-07 | 1.62E-05 |
| CLIP3 | -0.299 | Downregulated | 3.92E-05 | 4.09E-04 |
| TUBG1 | -0.298 | Downregulated | 3.19E-10 | 2.92E-08 |
| EXOSC7 | -0.298 | Downregulated | 6.30E-08 | 2.05E-06 |
| RAPGEF5 | -0.298 | Downregulated | 3.70E-06 | 5.76E-05 |
| BPNT1 | -0.298 | Downregulated | 1.54E-07 | 4.31E-06 |
| WDR57 | -0.298 | Downregulated | 3.04E-12 | 8.18E-10 |
| HPGD | -0.298 | Downregulated | 1.44E-03 | 8.40E-03 |
| GNLY | -0.298 | Downregulated | 1.09E-02 | 4.39E-02 |
| CSE1L | -0.298 | Downregulated | 2.94E-09 | 1.75E-07 |
| DONSON | -0.297 | Downregulated | 1.92E-07 | 5.12E-06 |
| FLJ46020 | -0.297 | Downregulated | 1.83E-08 | 7.34E-07 |
| CDCA8 | -0.297 | Downregulated | 7.28E-08 | 2.30E-06 |
| FCRL3 | -0.297 | Downregulated | 4.03E-05 | 4.17E-04 |
| DPAGT1 | -0.297 | Downregulated | 1.30E-12 | 4.41E-10 |
| RRM1 | -0.296 | Downregulated | 2.55E-05 | 2.87E-04 |
| FUBP1 | -0.296 | Downregulated | 9.82E-10 | 7.41E-08 |
| ATRIP | -0.296 | Downregulated | 1.46E-13 | 7.45E-11 |
| C8ORF13 | -0.296 | Downregulated | 4.29E-04 | 3.05E-03 |
| EZR | -0.296 | Downregulated | 1.62E-10 | 1.71E-08 |
| RAB33A | -0.296 | Downregulated | 4.74E-10 | 4.05E-08 |
| KIAA1324L | -0.296 | Downregulated | 7.87E-05 | 7.32E-04 |
| C11ORF48 | -0.296 | Downregulated | 2.40E-11 | 4.11E-09 |
| BCL2L12 | -0.296 | Downregulated | 1.19E-11 | 2.29E-09 |
| RFTN1 | -0.295 | Downregulated | 3.30E-08 | 1.20E-06 |
| NUP37 | -0.295 | Downregulated | 4.21E-09 | 2.32E-07 |
| WFS1 | -0.295 | Downregulated | 1.28E-06 | 2.37E-05 |

| | | | | |
|---|---|---|---|---|
| SELS | -0.295 | Downregulated | 2.37E-08 | 9.08E-07 |
| CD8A | -0.294 | Downregulated | 2.81E-03 | 1.46E-02 |
| FKBP11 | -0.294 | Downregulated | 9.63E-05 | 8.67E-04 |
| HIST1H4C | -0.294 | Downregulated | 3.14E-04 | 2.34E-03 |
| TBC1D7 | -0.293 | Downregulated | 5.85E-13 | 2.33E-10 |
| MCEE | -0.293 | Downregulated | 2.24E-05 | 2.57E-04 |
| RUVBL1 | -0.293 | Downregulated | 1.02E-11 | 2.03E-09 |
| EWSR1 | -0.293 | Downregulated | 2.02E-09 | 1.29E-07 |
| UHRF1 | -0.293 | Downregulated | 2.17E-07 | 5.68E-06 |
| ZBTB32 | -0.293 | Downregulated | 2.61E-07 | 6.53E-06 |
| C20ORF52 | -0.293 | Downregulated | 6.97E-08 | 2.22E-06 |
| GLCCI1 | -0.293 | Downregulated | 2.18E-07 | 5.69E-06 |
| LSM4 | -0.293 | Downregulated | 1.44E-08 | 6.18E-07 |
| MPV17 | -0.293 | Downregulated | 8.46E-13 | 3.14E-10 |
| NXT1 | -0.292 | Downregulated | 1.35E-10 | 1.48E-08 |
| FAM159A | -0.292 | Downregulated | 5.65E-06 | 8.18E-05 |
| PPIH | -0.291 | Downregulated | 1.61E-09 | 1.07E-07 |
| FARS2 | -0.291 | Downregulated | 3.59E-11 | 5.56E-09 |
| ATIC | -0.291 | Downregulated | 4.60E-08 | 1.59E-06 |
| PAFAH1B3 | -0.290 | Downregulated | 9.26E-08 | 2.82E-06 |
| N6AMT2 | -0.290 | Downregulated | 1.89E-10 | 1.93E-08 |
| SHMT2 | -0.290 | Downregulated | 5.25E-08 | 1.78E-06 |
| PDXP | -0.290 | Downregulated | 2.52E-08 | 9.50E-07 |
| KBTBD8 | -0.290 | Downregulated | 5.35E-06 | 7.82E-05 |
| BTLA | -0.290 | Downregulated | 3.05E-04 | 2.29E-03 |
| NHP2 | -0.290 | Downregulated | 1.77E-10 | 1.84E-08 |
| KRTCAP2 | -0.290 | Downregulated | 1.65E-12 | 5.06E-10 |
| HLA-DOB | -0.290 | Downregulated | 5.58E-04 | 3.81E-03 |
| CCR6 | -0.290 | Downregulated | 2.24E-04 | 1.76E-03 |
| POLR1C | -0.290 | Downregulated | 4.01E-12 | 1.00E-09 |
| GTPBP4 | -0.289 | Downregulated | 3.41E-07 | 8.06E-06 |
| FAM44B | -0.289 | Downregulated | 1.16E-06 | 2.18E-05 |
| SEH1L | -0.289 | Downregulated | 9.43E-12 | 1.90E-09 |
| SLAMF6 | -0.289 | Downregulated | 8.55E-07 | 1.71E-05 |
| ICT1 | -0.289 | Downregulated | 1.53E-07 | 4.29E-06 |
| BIK | -0.288 | Downregulated | 7.21E-05 | 6.80E-04 |
| RBM4B | -0.288 | Downregulated | 8.21E-13 | 3.10E-10 |
| MIB2 | -0.287 | Downregulated | 1.69E-08 | 6.91E-07 |
| TIMM44 | -0.287 | Downregulated | 4.50E-09 | 2.44E-07 |
| RNMTL1 | -0.287 | Downregulated | 2.99E-10 | 2.77E-08 |
| GLO1 | -0.287 | Downregulated | 1.26E-05 | 1.60E-04 |
| CEP78 | -0.286 | Downregulated | 2.03E-04 | 1.62E-03 |
| EPHA4 | -0.286 | Downregulated | 2.33E-05 | 2.66E-04 |
| ETFB | -0.286 | Downregulated | 1.89E-11 | 3.40E-09 |
| LEF1 | -0.286 | Downregulated | 7.12E-03 | 3.12E-02 |
| NIT2 | -0.286 | Downregulated | 3.90E-09 | 2.18E-07 |
| SERP2 | -0.286 | Downregulated | 1.57E-07 | 4.36E-06 |
| UBA5 | -0.285 | Downregulated | 6.13E-07 | 1.30E-05 |
| MED8 | -0.285 | Downregulated | 2.96E-12 | 8.10E-10 |
| LAX1 | -0.285 | Downregulated | 5.15E-06 | 7.58E-05 |
| ZFP90 | -0.285 | Downregulated | 5.97E-08 | 1.97E-06 |

| | | | | |
|---|---|---|---|---|
| KIAA0391 | -0.284 | Downregulated | 6.79E-13 | 2.64E-10 |
| C17ORF53 | -0.284 | Downregulated | 1.11E-05 | 1.43E-04 |
| FAM102A | -0.284 | Downregulated | 2.42E-04 | 1.89E-03 |
| BZW2 | -0.284 | Downregulated | 5.43E-07 | 1.17E-05 |
| HIRIP3 | -0.284 | Downregulated | 1.29E-09 | 9.01E-08 |
| SPRED1 | -0.284 | Downregulated | 4.69E-04 | 3.29E-03 |
| IDH3A | -0.284 | Downregulated | 6.38E-12 | 1.45E-09 |
| SEC22A | -0.284 | Downregulated | 1.43E-08 | 6.14E-07 |
| MCPH1 | -0.284 | Downregulated | 4.61E-18 | 5.34E-14 |
| WDR51A | -0.284 | Downregulated | 1.76E-06 | 3.09E-05 |
| BCS1L | -0.284 | Downregulated | 1.82E-08 | 7.30E-07 |
| AES | -0.283 | Downregulated | 1.44E-06 | 2.62E-05 |
| ZW10 | -0.283 | Downregulated | 7.89E-11 | 9.74E-09 |
| TTC27 | -0.283 | Downregulated | 1.97E-06 | 3.39E-05 |
| MRPL52 | -0.283 | Downregulated | 4.89E-09 | 2.60E-07 |
| MTA3 | -0.283 | Downregulated | 2.15E-09 | 1.36E-07 |
| SLC25A29 | -0.283 | Downregulated | 1.72E-05 | 2.06E-04 |
| FMO4 | -0.283 | Downregulated | 1.51E-08 | 6.37E-07 |
| PDCD5 | -0.283 | Downregulated | 3.82E-09 | 2.16E-07 |
| KIF11 | -0.282 | Downregulated | 2.65E-04 | 2.03E-03 |
| MCOLN2 | -0.282 | Downregulated | 1.96E-03 | 1.08E-02 |
| PEBP1 | -0.282 | Downregulated | 5.04E-08 | 1.72E-06 |
| DCTPP1 | -0.282 | Downregulated | 8.40E-10 | 6.60E-08 |
| TMED3 | -0.282 | Downregulated | 8.73E-09 | 4.13E-07 |
| PGAM5 | -0.282 | Downregulated | 2.76E-15 | 2.84E-12 |
| SAAL1 | -0.282 | Downregulated | 1.10E-06 | 2.10E-05 |
| NSA2 | -0.282 | Downregulated | 4.35E-06 | 6.61E-05 |
| SIVA | -0.281 | Downregulated | 5.43E-12 | 1.27E-09 |
| CDK2AP2 | -0.281 | Downregulated | 1.40E-08 | 6.02E-07 |
| PDE7B | -0.281 | Downregulated | 1.93E-04 | 1.56E-03 |
| CHAF1A | -0.281 | Downregulated | 7.79E-08 | 2.44E-06 |
| MYL6B | -0.280 | Downregulated | 2.64E-07 | 6.57E-06 |
| CENPP | -0.280 | Downregulated | 7.54E-07 | 1.54E-05 |
| POLE2 | -0.280 | Downregulated | 3.10E-06 | 4.96E-05 |
| HERC6 | -0.280 | Downregulated | 1.13E-02 | 4.51E-02 |
| PSMD10 | -0.280 | Downregulated | 2.67E-08 | 1.00E-06 |
| C1ORF149 | -0.280 | Downregulated | 2.23E-08 | 8.61E-07 |
| RRP15 | -0.280 | Downregulated | 1.03E-06 | 1.98E-05 |
| NOP58 | -0.280 | Downregulated | 3.92E-05 | 4.09E-04 |
| HSP90B1 | -0.280 | Downregulated | 1.42E-04 | 1.21E-03 |
| MOXD1 | -0.280 | Downregulated | 5.48E-07 | 1.18E-05 |
| MYH10 | -0.280 | Downregulated | 2.53E-06 | 4.18E-05 |
| UCHL5 | -0.279 | Downregulated | 2.08E-06 | 3.53E-05 |
| FIBP | -0.279 | Downregulated | 8.07E-14 | 4.91E-11 |
| ILF2 | -0.279 | Downregulated | 4.79E-09 | 2.56E-07 |
| MAGED1 | -0.279 | Downregulated | 7.84E-09 | 3.78E-07 |
| NDUFA12 | -0.279 | Downregulated | 3.98E-09 | 2.22E-07 |
| SLC35F2 | -0.278 | Downregulated | 1.09E-06 | 2.07E-05 |
| OIP5 | -0.278 | Downregulated | 3.65E-06 | 5.70E-05 |
| ATPAF2 | -0.278 | Downregulated | 4.80E-11 | 6.94E-09 |
| TMEM168 | -0.278 | Downregulated | 3.72E-07 | 8.60E-06 |

| | | | | |
|---|---|---|---|---|
| ZBTB46 | -0.278 | Downregulated | 2.12E-04 | 1.68E-03 |
| VRK1 | -0.278 | Downregulated | 1.18E-04 | 1.03E-03 |
| OSBPL9 | -0.278 | Downregulated | 6.59E-08 | 2.12E-06 |
| CENPE | -0.278 | Downregulated | 9.62E-06 | 1.27E-04 |
| IMP4 | -0.278 | Downregulated | 3.67E-09 | 2.10E-07 |
| KIF3C | -0.277 | Downregulated | 1.27E-06 | 2.35E-05 |
| SCAMP5 | -0.277 | Downregulated | 2.73E-06 | 4.46E-05 |
| TEX264 | -0.277 | Downregulated | 3.24E-12 | 8.43E-10 |
| POLR2F | -0.277 | Downregulated | 1.56E-12 | 4.89E-10 |
| CXORF56 | -0.277 | Downregulated | 7.49E-11 | 9.38E-09 |
| KIF2C | -0.277 | Downregulated | 2.15E-06 | 3.64E-05 |
| FEN1 | -0.277 | Downregulated | 1.64E-07 | 4.54E-06 |
| RPUSD2 | -0.277 | Downregulated | 7.84E-09 | 3.78E-07 |
| NSMCE2 | -0.276 | Downregulated | 3.99E-05 | 4.14E-04 |
| CSTB | -0.276 | Downregulated | 1.36E-12 | 4.55E-10 |
| FLJ33590 | -0.276 | Downregulated | 5.69E-04 | 3.87E-03 |
| NDUFB7 | -0.276 | Downregulated | 5.12E-06 | 7.54E-05 |
| NCR3 | -0.276 | Downregulated | 3.30E-05 | 3.53E-04 |
| STAMBPL1 | -0.276 | Downregulated | 1.40E-04 | 1.19E-03 |
| DDX10 | -0.275 | Downregulated | 3.86E-08 | 1.37E-06 |
| ARPC5L | -0.275 | Downregulated | 6.64E-10 | 5.48E-08 |
| ZDHHC16 | -0.275 | Downregulated | 2.32E-13 | 1.14E-10 |
| LAGE3 | -0.275 | Downregulated | 4.65E-07 | 1.04E-05 |
| FAM195B | -0.275 | Downregulated | 2.65E-11 | 4.44E-09 |
| DDX50 | -0.275 | Downregulated | 9.78E-07 | 1.90E-05 |
| KLHL3 | -0.275 | Downregulated | 5.38E-04 | 3.69E-03 |
| MRPS24 | -0.274 | Downregulated | 3.45E-09 | 2.00E-07 |
| FLJ23834 | -0.274 | Downregulated | 1.68E-04 | 1.39E-03 |
| CCDC34 | -0.274 | Downregulated | 2.09E-04 | 1.66E-03 |
| ENDOG | -0.274 | Downregulated | 6.41E-08 | 2.08E-06 |
| NOLC1 | -0.274 | Downregulated | 2.95E-08 | 1.09E-06 |
| COX10 | -0.274 | Downregulated | 1.26E-09 | 8.92E-08 |
| GSPT2 | -0.274 | Downregulated | 2.38E-07 | 6.08E-06 |
| FAM58A | -0.274 | Downregulated | 5.91E-09 | 3.04E-07 |
| SUV39H1 | -0.274 | Downregulated | 8.59E-08 | 2.64E-06 |
| TMEM48 | -0.274 | Downregulated | 1.75E-08 | 7.07E-07 |
| API5 | -0.273 | Downregulated | 2.13E-08 | 8.26E-07 |
| WDR70 | -0.273 | Downregulated | 8.15E-08 | 2.53E-06 |
| TYSND1 | -0.273 | Downregulated | 3.62E-07 | 8.42E-06 |
| CKAP5 | -0.272 | Downregulated | 1.88E-09 | 1.21E-07 |
| GATM | -0.272 | Downregulated | 4.74E-05 | 4.80E-04 |
| ILDR1 | -0.272 | Downregulated | 2.80E-07 | 6.87E-06 |
| SLC25A15 | -0.272 | Downregulated | 6.99E-10 | 5.72E-08 |
| SAE1 | -0.271 | Downregulated | 7.38E-09 | 3.63E-07 |
| THEM4 | -0.271 | Downregulated | 1.09E-04 | 9.61E-04 |
| CD83 | -0.271 | Downregulated | 3.84E-06 | 5.93E-05 |
| C1QBP | -0.271 | Downregulated | 3.47E-07 | 8.17E-06 |
| NDUFS8 | -0.271 | Downregulated | 2.19E-09 | 1.38E-07 |
| DIS3L | -0.271 | Downregulated | 9.62E-08 | 2.91E-06 |
| SAMD3 | -0.271 | Downregulated | 7.35E-04 | 4.79E-03 |
| NCAPG2 | -0.270 | Downregulated | 1.66E-05 | 1.99E-04 |

| | | | | |
|---|---|---|---|---|
| DNAJC19 | -0.270 | Downregulated | 3.86E-07 | 8.88E-06 |
| DCPS | -0.270 | Downregulated | 3.56E-08 | 1.27E-06 |
| RBM14 | -0.269 | Downregulated | 5.37E-07 | 1.17E-05 |
| MIPEP | -0.269 | Downregulated | 1.55E-07 | 4.34E-06 |
| CPA5 | -0.269 | Downregulated | 1.31E-04 | 1.13E-03 |
| SIVA1 | -0.269 | Downregulated | 5.79E-10 | 4.86E-08 |
| BCCIP | -0.269 | Downregulated | 4.98E-11 | 7.12E-09 |
| C14ORF124 | -0.269 | Downregulated | 9.53E-09 | 4.40E-07 |
| PPP1R14A | -0.269 | Downregulated | 6.41E-06 | 9.14E-05 |
| TRAM2 | -0.269 | Downregulated | 6.32E-05 | 6.09E-04 |
| C19ORF44 | -0.269 | Downregulated | 1.23E-09 | 8.85E-08 |
| LARS2 | -0.269 | Downregulated | 9.91E-08 | 2.98E-06 |
| DNAJC3 | -0.269 | Downregulated | 5.72E-05 | 5.61E-04 |
| FLJ35801 | -0.269 | Downregulated | 1.59E-06 | 2.84E-05 |
| CD3D | -0.269 | Downregulated | 1.79E-03 | 1.01E-02 |
| LYAR | -0.268 | Downregulated | 7.75E-08 | 2.43E-06 |
| AHCY | -0.268 | Downregulated | 1.24E-07 | 3.59E-06 |
| WEE1 | -0.268 | Downregulated | 1.40E-05 | 1.74E-04 |
| C16ORF30 | -0.268 | Downregulated | 2.43E-03 | 1.30E-02 |
| DDX47 | -0.268 | Downregulated | 6.81E-09 | 3.40E-07 |
| C6ORF190 | -0.268 | Downregulated | 4.57E-03 | 2.17E-02 |
| CDCA7 | -0.268 | Downregulated | 1.84E-07 | 4.94E-06 |
| PRMT1 | -0.267 | Downregulated | 1.10E-08 | 4.99E-07 |
| ABLIM1 | -0.267 | Downregulated | 6.96E-04 | 4.58E-03 |
| SNRNP40 | -0.267 | Downregulated | 2.47E-11 | 4.18E-09 |
| RNASEH2A | -0.267 | Downregulated | 1.19E-06 | 2.23E-05 |
| WBSCR22 | -0.267 | Downregulated | 8.63E-09 | 4.09E-07 |
| FOXRED1 | -0.267 | Downregulated | 5.26E-09 | 2.76E-07 |
| C11ORF10 | -0.267 | Downregulated | 8.54E-10 | 6.67E-08 |
| TMEM160 | -0.267 | Downregulated | 2.52E-10 | 2.46E-08 |
| TOMM22 | -0.266 | Downregulated | 8.69E-10 | 6.78E-08 |
| RPA3 | -0.266 | Downregulated | 2.69E-04 | 2.06E-03 |
| CPSF6 | -0.266 | Downregulated | 2.41E-07 | 6.14E-06 |
| TWISTNB | -0.266 | Downregulated | 7.47E-06 | 1.04E-04 |
| SDAD1 | -0.266 | Downregulated | 1.93E-09 | 1.24E-07 |
| GEMIN8 | -0.266 | Downregulated | 1.41E-09 | 9.65E-08 |
| DPPA4 | -0.266 | Downregulated | 4.43E-04 | 3.13E-03 |
| PDIA5 | -0.266 | Downregulated | 1.45E-05 | 1.79E-04 |
| TUBB2B | -0.266 | Downregulated | 5.72E-06 | 8.27E-05 |
| KMO | -0.266 | Downregulated | 1.24E-04 | 1.08E-03 |
| AEBP1 | -0.266 | Downregulated | 4.50E-05 | 4.59E-04 |
| DENND2D | -0.265 | Downregulated | 6.64E-06 | 9.41E-05 |
| DGUOK | -0.265 | Downregulated | 3.54E-15 | 3.51E-12 |
| SLC15A2 | -0.265 | Downregulated | 1.58E-07 | 4.39E-06 |
| MRPL38 | -0.265 | Downregulated | 2.93E-08 | 1.09E-06 |
| PDK1 | 0.265 | Upregulated | 3.04E-07 | 7.34E-06 |
| RELB | 0.265 | Upregulated | 3.02E-05 | 3.28E-04 |
| CFB | 0.266 | Upregulated | 8.45E-09 | 4.03E-07 |
| ATF4 | 0.266 | Upregulated | 1.55E-05 | 1.89E-04 |
| LY6G6F | 0.267 | Upregulated | 1.73E-03 | 9.79E-03 |
| DDEF2 | 0.267 | Upregulated | 3.44E-06 | 5.43E-05 |

| | | | | |
|---|---|---|---|---|
| TAF4B | 0.268 | Upregulated | 3.88E-05 | 4.05E-04 |
| CPVL | 0.268 | Upregulated | 3.63E-03 | 1.80E-02 |
| GABARAP | 0.268 | Upregulated | 1.81E-07 | 4.87E-06 |
| ARAP2 | 0.268 | Upregulated | 1.90E-07 | 5.08E-06 |
| MBOAT2 | 0.268 | Upregulated | 3.48E-07 | 8.20E-06 |
| CR1 | 0.269 | Upregulated | 5.11E-03 | 2.38E-02 |
| TMEM154 | 0.269 | Upregulated | 3.17E-06 | 5.06E-05 |
| PRDM8 | 0.269 | Upregulated | 1.77E-11 | 3.21E-09 |
| CROP | 0.269 | Upregulated | 8.06E-04 | 5.16E-03 |
| PPP1R15B | 0.270 | Upregulated | 1.86E-09 | 1.20E-07 |
| SMNDC1 | 0.270 | Upregulated | 4.03E-05 | 4.18E-04 |
| ATP11B | 0.270 | Upregulated | 9.24E-06 | 1.24E-04 |
| SPTA1 | 0.270 | Upregulated | 5.85E-04 | 3.96E-03 |
| LYZ | 0.270 | Upregulated | 9.93E-05 | 8.90E-04 |
| SLC6A6 | 0.271 | Upregulated | 4.69E-04 | 3.29E-03 |
| GDPD5 | 0.271 | Upregulated | 4.56E-05 | 4.64E-04 |
| GATA1 | 0.271 | Upregulated | 2.93E-03 | 1.51E-02 |
| ITGAM | 0.271 | Upregulated | 1.33E-03 | 7.87E-03 |
| MBD4 | 0.271 | Upregulated | 6.31E-04 | 4.22E-03 |
| RAP1B | 0.271 | Upregulated | 3.61E-06 | 5.64E-05 |
| PABPC1 | 0.272 | Upregulated | 2.32E-06 | 3.88E-05 |
| EXOC8 | 0.272 | Upregulated | 6.17E-06 | 8.84E-05 |
| TUBB1 | 0.272 | Upregulated | 4.09E-03 | 1.99E-02 |
| C4ORF3 | 0.273 | Upregulated | 3.29E-05 | 3.52E-04 |
| HSN2 | 0.273 | Upregulated | 2.45E-13 | 1.17E-10 |
| PLAUR | 0.273 | Upregulated | 9.85E-04 | 6.10E-03 |
| SQRDL | 0.273 | Upregulated | 4.28E-05 | 4.39E-04 |
| RHBDL2 | 0.274 | Upregulated | 5.56E-05 | 5.47E-04 |
| FGFR1OP2 | 0.274 | Upregulated | 9.88E-07 | 1.91E-05 |
| TBC1D10B | 0.274 | Upregulated | 1.48E-03 | 8.57E-03 |
| IL1F9 | 0.275 | Upregulated | 3.46E-04 | 2.54E-03 |
| SUCNR1 | 0.275 | Upregulated | 9.36E-06 | 1.25E-04 |
| WDR26 | 0.275 | Upregulated | 1.31E-07 | 3.78E-06 |
| RHCE | 0.275 | Upregulated | 9.95E-05 | 8.91E-04 |
| NAMPT | 0.276 | Upregulated | 1.53E-02 | 5.75E-02 |
| ROCK1 | 0.276 | Upregulated | 1.89E-05 | 2.22E-04 |
| PLIN5 | 0.277 | Upregulated | 9.44E-05 | 8.52E-04 |
| GADD45B | 0.277 | Upregulated | 6.82E-06 | 9.61E-05 |
| CD300A | 0.277 | Upregulated | 2.02E-05 | 2.35E-04 |
| RILP | 0.277 | Upregulated | 4.30E-05 | 4.41E-04 |
| DDX17 | 0.277 | Upregulated | 5.81E-05 | 5.68E-04 |
| CTR9 | 0.277 | Upregulated | 1.16E-06 | 2.18E-05 |
| CD36 | 0.278 | Upregulated | 7.20E-04 | 4.71E-03 |
| ZC3H11A | 0.278 | Upregulated | 6.69E-09 | 3.36E-07 |
| ZNF600 | 0.278 | Upregulated | 2.82E-04 | 2.14E-03 |
| NFIA | 0.278 | Upregulated | 1.32E-05 | 1.66E-04 |
| PTPRC | 0.278 | Upregulated | 2.97E-04 | 2.25E-03 |
| CXORF39 | 0.278 | Upregulated | 5.56E-06 | 8.08E-05 |
| OLR1 | 0.278 | Upregulated | 1.52E-02 | 5.73E-02 |
| PNPLA8 | 0.279 | Upregulated | 1.37E-05 | 1.71E-04 |
| LCP2 | 0.279 | Upregulated | 4.88E-10 | 4.15E-08 |

| | | | | |
|---|---|---|---|---|
| ZBTB2 | 0.279 | Upregulated | 2.26E-06 | 3.79E-05 |
| PARP9 | 0.279 | Upregulated | 1.73E-04 | 1.42E-03 |
| FLJ27255 | 0.279 | Upregulated | 4.23E-07 | 9.62E-06 |
| RNPC3 | 0.279 | Upregulated | 9.82E-10 | 7.41E-08 |
| CENTD2 | 0.279 | Upregulated | 1.89E-10 | 1.93E-08 |
| TMEM106A | 0.280 | Upregulated | 9.05E-03 | 3.78E-02 |
| HMGB1 | 0.280 | Upregulated | 1.26E-05 | 1.60E-04 |
| ATF1 | 0.280 | Upregulated | 7.49E-06 | 1.04E-04 |
| SRRD | 0.281 | Upregulated | 2.95E-03 | 1.52E-02 |
| RTN3 | 0.281 | Upregulated | 1.67E-05 | 2.01E-04 |
| DNAJC25-GNG10 | 0.281 | Upregulated | 2.41E-08 | 9.14E-07 |
| OAS3 | 0.281 | Upregulated | 8.62E-03 | 3.64E-02 |
| CSNK1A1 | 0.281 | Upregulated | 2.14E-09 | 1.35E-07 |
| SNAP23 | 0.282 | Upregulated | 4.93E-06 | 7.31E-05 |
| JAK2 | 0.282 | Upregulated | 8.76E-05 | 7.99E-04 |
| NADK | 0.282 | Upregulated | 1.16E-04 | 1.01E-03 |
| PRO1853 | 0.282 | Upregulated | 1.09E-03 | 6.65E-03 |
| TRAFD1 | 0.283 | Upregulated | 3.86E-06 | 5.95E-05 |
| PMAIP1 | 0.283 | Upregulated | 2.57E-07 | 6.44E-06 |
| SV2A | 0.283 | Upregulated | 1.39E-04 | 1.18E-03 |
| TTC32 | 0.283 | Upregulated | 2.22E-14 | 1.78E-11 |
| PPM1B | 0.283 | Upregulated | 4.47E-07 | 1.01E-05 |
| GRN | 0.283 | Upregulated | 2.24E-04 | 1.76E-03 |
| GAA | 0.283 | Upregulated | 6.88E-05 | 6.53E-04 |
| RPS15A | 0.283 | Upregulated | 1.71E-02 | 6.30E-02 |
| LPCAT2 | 0.284 | Upregulated | 7.80E-03 | 3.36E-02 |
| MYL4 | 0.284 | Upregulated | 2.51E-02 | 8.52E-02 |
| FAM116B | 0.284 | Upregulated | 2.04E-04 | 1.63E-03 |
| BAGE2 | 0.284 | Upregulated | 2.62E-09 | 1.60E-07 |
| KLF5 | 0.284 | Upregulated | 3.45E-10 | 3.11E-08 |
| CALCOCO2 | 0.285 | Upregulated | 2.62E-10 | 2.51E-08 |
| SERINC3 | 0.285 | Upregulated | 4.36E-07 | 9.83E-06 |
| MAGT1 | 0.285 | Upregulated | 2.38E-06 | 3.96E-05 |
| UBA6 | 0.286 | Upregulated | 4.66E-05 | 4.73E-04 |
| HLA-C | 0.286 | Upregulated | 1.05E-02 | 4.27E-02 |
| STX11 | 0.286 | Upregulated | 5.25E-04 | 3.62E-03 |
| RRM2B | 0.286 | Upregulated | 1.40E-05 | 1.74E-04 |
| DMXL2 | 0.286 | Upregulated | 1.26E-05 | 1.60E-04 |
| CEP27 | 0.286 | Upregulated | 6.25E-04 | 4.18E-03 |
| TMEM176A | 0.287 | Upregulated | 4.13E-02 | 1.25E-01 |
| SPIN1 | 0.287 | Upregulated | 7.39E-06 | 1.03E-04 |
| OR2W3 | 0.287 | Upregulated | 9.31E-03 | 3.87E-02 |
| SLC38A5 | 0.287 | Upregulated | 2.27E-04 | 1.78E-03 |
| VSIG4 | 0.287 | Upregulated | 3.12E-04 | 2.33E-03 |
| HS2ST1 | 0.287 | Upregulated | 2.73E-07 | 6.75E-06 |
| EVI2A | 0.288 | Upregulated | 3.81E-03 | 1.87E-02 |
| DMXL1 | 0.288 | Upregulated | 8.18E-06 | 1.12E-04 |
| ASAP2 | 0.288 | Upregulated | 3.85E-07 | 8.86E-06 |
| PANX2 | 0.289 | Upregulated | 4.24E-03 | 2.04E-02 |
| FPR1 | 0.289 | Upregulated | 1.72E-03 | 9.74E-03 |
| FHDC1 | 0.289 | Upregulated | 1.82E-05 | 2.16E-04 |

| | | | | |
|---|---|---|---|---|
| UHMK1 | 0.290 | Upregulated | 1.17E-06 | 2.19E-05 |
| CARS2 | 0.290 | Upregulated | 4.93E-10 | 4.18E-08 |
| SLC26A8 | 0.291 | Upregulated | 5.92E-04 | 4.00E-03 |
| SUMO4 | 0.291 | Upregulated | 1.57E-09 | 1.05E-07 |
| DDX60L | 0.291 | Upregulated | 2.29E-03 | 1.24E-02 |
| SFRS12 | 0.291 | Upregulated | 2.53E-07 | 6.36E-06 |
| ANKRD33 | 0.291 | Upregulated | 4.12E-04 | 2.95E-03 |
| WDR51B | 0.291 | Upregulated | 7.47E-07 | 1.53E-05 |
| SCO2 | 0.292 | Upregulated | 6.38E-04 | 4.26E-03 |
| HLA-DRB3 | 0.292 | Upregulated | 3.54E-04 | 2.58E-03 |
| TGM2 | 0.292 | Upregulated | 2.52E-03 | 1.34E-02 |
| GP9 | 0.293 | Upregulated | 7.83E-03 | 3.37E-02 |
| MAOA | 0.293 | Upregulated | 3.82E-03 | 1.88E-02 |
| CCNDBP1 | 0.294 | Upregulated | 2.60E-05 | 2.90E-04 |
| CARS | 0.294 | Upregulated | 2.30E-14 | 1.78E-11 |
| UBE2O | 0.294 | Upregulated | 2.48E-03 | 1.32E-02 |
| OSBPL11 | 0.294 | Upregulated | 2.67E-05 | 2.97E-04 |
| TRIM38 | 0.295 | Upregulated | 8.80E-09 | 4.15E-07 |
| CTSO | 0.295 | Upregulated | 4.35E-07 | 9.83E-06 |
| TMSL3 | 0.295 | Upregulated | 8.07E-08 | 2.51E-06 |
| CMTM5 | 0.295 | Upregulated | 2.31E-03 | 1.25E-02 |
| CCNT2 | 0.295 | Upregulated | 7.38E-06 | 1.02E-04 |
| ISCA1 | 0.295 | Upregulated | 1.70E-05 | 2.04E-04 |
| TRIM10 | 0.295 | Upregulated | 3.37E-04 | 2.49E-03 |
| GCLC | 0.296 | Upregulated | 5.42E-08 | 1.82E-06 |
| DYNC1LI1 | 0.296 | Upregulated | 1.74E-08 | 7.06E-07 |
| POLB | 0.296 | Upregulated | 1.40E-09 | 9.64E-08 |
| IL17RA | 0.296 | Upregulated | 1.32E-04 | 1.14E-03 |
| UBE2B | 0.297 | Upregulated | 2.14E-07 | 5.62E-06 |
| CHD1 | 0.297 | Upregulated | 9.93E-06 | 1.31E-04 |
| MAP1S | 0.297 | Upregulated | 4.39E-04 | 3.11E-03 |
| GBP2 | 0.298 | Upregulated | 2.81E-08 | 1.05E-06 |
| ZAK | 0.298 | Upregulated | 7.48E-05 | 7.01E-04 |
| RANGRF | 0.298 | Upregulated | 1.76E-05 | 2.09E-04 |
| RBAK | 0.299 | Upregulated | 5.39E-03 | 2.48E-02 |
| TRIML2 | 0.299 | Upregulated | 7.39E-10 | 5.99E-08 |
| FBXO7 | 0.299 | Upregulated | 1.91E-02 | 6.87E-02 |
| FNDC3A | 0.300 | Upregulated | 9.48E-05 | 8.55E-04 |
| AQP12A | 0.300 | Upregulated | 3.17E-05 | 3.42E-04 |
| HSPA6 | 0.300 | Upregulated | 1.22E-04 | 1.06E-03 |
| C1ORF24 | 0.300 | Upregulated | 6.56E-04 | 4.36E-03 |
| UBE4A | 0.301 | Upregulated | 1.20E-06 | 2.24E-05 |
| GNB4 | 0.301 | Upregulated | 1.22E-06 | 2.27E-05 |
| TLR4 | 0.302 | Upregulated | 7.70E-04 | 4.97E-03 |
| OR52K2 | 0.302 | Upregulated | 1.75E-07 | 4.76E-06 |
| SHKBP1 | 0.302 | Upregulated | 1.41E-04 | 1.20E-03 |
| CPD | 0.302 | Upregulated | 7.15E-05 | 6.75E-04 |
| MMRN1 | 0.303 | Upregulated | 1.44E-03 | 8.36E-03 |
| PTGR2 | 0.303 | Upregulated | 1.60E-05 | 1.94E-04 |
| SP3 | 0.303 | Upregulated | 5.57E-08 | 1.86E-06 |
| ESAM | 0.303 | Upregulated | 2.37E-03 | 1.27E-02 |

| | | | | |
|---|---|---|---|---|
| RFWD2 | 0.303 | Upregulated | 1.03E-07 | 3.07E-06 |
| HSPA4 | 0.304 | Upregulated | 2.40E-06 | 3.99E-05 |
| SEC14L1 | 0.304 | Upregulated | 9.76E-05 | 8.78E-04 |
| PTAFR | 0.304 | Upregulated | 6.43E-04 | 4.28E-03 |
| TRIP12 | 0.304 | Upregulated | 2.65E-07 | 6.58E-06 |
| SPATA2L | 0.304 | Upregulated | 1.18E-11 | 2.29E-09 |
| NR1D1 | 0.304 | Upregulated | 9.00E-05 | 8.18E-04 |
| RPL7 | 0.305 | Upregulated | 1.84E-03 | 1.03E-02 |
| ADCY3 | 0.305 | Upregulated | 1.52E-03 | 8.78E-03 |
| ASCC2 | 0.305 | Upregulated | 8.64E-04 | 5.47E-03 |
| TFE3 | 0.305 | Upregulated | 2.10E-07 | 5.54E-06 |
| BEND7 | 0.305 | Upregulated | 3.31E-07 | 7.86E-06 |
| COL10A1 | 0.305 | Upregulated | 1.88E-05 | 2.22E-04 |
| GYPC | 0.305 | Upregulated | 1.87E-03 | 1.04E-02 |
| SEC24A | 0.306 | Upregulated | 1.09E-04 | 9.66E-04 |
| RNF13 | 0.306 | Upregulated | 1.55E-08 | 6.48E-07 |
| THEX1 | 0.306 | Upregulated | 1.84E-06 | 3.21E-05 |
| DUSP1 | 0.307 | Upregulated | 1.05E-03 | 6.46E-03 |
| GABARAPL2 | 0.307 | Upregulated | 3.56E-08 | 1.27E-06 |
| SPARC | 0.307 | Upregulated | 1.39E-03 | 8.12E-03 |
| PXN | 0.307 | Upregulated | 8.88E-07 | 1.76E-05 |
| IL8 | 0.307 | Upregulated | 4.33E-04 | 3.07E-03 |
| USP8 | 0.307 | Upregulated | 2.43E-07 | 6.17E-06 |
| ITPRIP | 0.308 | Upregulated | 3.53E-07 | 8.27E-06 |
| TYMP | 0.308 | Upregulated | 2.23E-04 | 1.75E-03 |
| ARHGAP18 | 0.308 | Upregulated | 9.05E-09 | 4.24E-07 |
| DHRS12 | 0.310 | Upregulated | 2.84E-06 | 4.61E-05 |
| SPHAR | 0.311 | Upregulated | 1.09E-06 | 2.09E-05 |
| HSD17B11 | 0.311 | Upregulated | 3.66E-06 | 5.71E-05 |
| ESPN | 0.312 | Upregulated | 1.51E-02 | 5.70E-02 |
| CXCL5 | 0.312 | Upregulated | 8.08E-04 | 5.18E-03 |
| ABP1 | 0.312 | Upregulated | 2.76E-02 | 9.19E-02 |
| ATF5 | 0.312 | Upregulated | 6.64E-05 | 6.35E-04 |
| HSPB1 | 0.313 | Upregulated | 2.83E-04 | 2.15E-03 |
| TRIM56 | 0.313 | Upregulated | 3.28E-09 | 1.92E-07 |
| DDX6 | 0.313 | Upregulated | 1.41E-05 | 1.76E-04 |
| GPX8 | 0.314 | Upregulated | 1.64E-05 | 1.98E-04 |
| PPP2R5B | 0.314 | Upregulated | 8.40E-06 | 1.14E-04 |
| C1ORF138 | 0.314 | Upregulated | 8.21E-05 | 7.57E-04 |
| AFTPH | 0.314 | Upregulated | 2.40E-08 | 9.13E-07 |
| PTPN2 | 0.314 | Upregulated | 1.58E-08 | 6.59E-07 |
| RIOK3 | 0.315 | Upregulated | 5.47E-04 | 3.75E-03 |
| CSF3R | 0.315 | Upregulated | 7.33E-04 | 4.78E-03 |
| KIAA1539 | 0.315 | Upregulated | 3.07E-07 | 7.40E-06 |
| SF3B1 | 0.315 | Upregulated | 6.34E-07 | 1.33E-05 |
| CARD16 | 0.316 | Upregulated | 1.33E-07 | 3.80E-06 |
| MAP1LC3B2 | 0.316 | Upregulated | 1.12E-05 | 1.44E-04 |
| IL4R | 0.317 | Upregulated | 8.45E-06 | 1.15E-04 |
| DICER1 | 0.317 | Upregulated | 1.01E-09 | 7.58E-08 |
| RAX2 | 0.317 | Upregulated | 2.70E-05 | 3.00E-04 |
| HIST1H2AD | 0.317 | Upregulated | 1.89E-07 | 5.08E-06 |

| | | | | |
|---|---|---|---|---|
| JAM3 | 0.317 | Upregulated | 7.02E-04 | 4.61E-03 |
| ERGIC1 | 0.317 | Upregulated | 2.18E-05 | 2.51E-04 |
| YPEL3 | 0.317 | Upregulated | 7.30E-06 | 1.02E-04 |
| GCA | 0.318 | Upregulated | 1.33E-04 | 1.14E-03 |
| TOPORS | 0.318 | Upregulated | 2.04E-12 | 5.89E-10 |
| NUBPL | 0.318 | Upregulated | 2.15E-04 | 1.70E-03 |
| RBM7 | 0.318 | Upregulated | 1.89E-05 | 2.22E-04 |
| DPM2 | 0.318 | Upregulated | 9.51E-04 | 5.93E-03 |
| SLPI | 0.318 | Upregulated | 1.46E-02 | 5.55E-02 |
| SLC5A8 | 0.318 | Upregulated | 8.12E-04 | 5.20E-03 |
| TREM1 | 0.318 | Upregulated | 5.34E-04 | 3.67E-03 |
| BAGE3 | 0.320 | Upregulated | 1.77E-05 | 2.10E-04 |
| AZU1 | 0.320 | Upregulated | 9.60E-03 | 3.96E-02 |
| MTMR6 | 0.321 | Upregulated | 1.68E-05 | 2.02E-04 |
| TNFAIP6 | 0.321 | Upregulated | 3.15E-07 | 7.55E-06 |
| FZD2 | 0.321 | Upregulated | 6.90E-05 | 6.55E-04 |
| LYPLA1 | 0.321 | Upregulated | 6.32E-09 | 3.21E-07 |
| OR51S1 | 0.321 | Upregulated | 6.52E-08 | 2.10E-06 |
| ALS2CR14 | 0.321 | Upregulated | 7.92E-05 | 7.35E-04 |
| FTH1 | 0.322 | Upregulated | 1.92E-05 | 2.25E-04 |
| EVI5 | 0.322 | Upregulated | 8.17E-05 | 7.54E-04 |
| FCGR2A | 0.322 | Upregulated | 5.61E-04 | 3.83E-03 |
| TIPRL | 0.322 | Upregulated | 9.16E-07 | 1.80E-05 |
| PPP1R12A | 0.322 | Upregulated | 3.04E-05 | 3.30E-04 |
| SORL1 | 0.323 | Upregulated | 1.65E-04 | 1.36E-03 |
| RNF10 | 0.323 | Upregulated | 5.50E-04 | 3.77E-03 |
| KBTBD7 | 0.324 | Upregulated | 1.99E-05 | 2.32E-04 |
| PKN2 | 0.324 | Upregulated | 1.26E-08 | 5.55E-07 |
| BLZF1 | 0.324 | Upregulated | 3.43E-06 | 5.42E-05 |
| REM2 | 0.324 | Upregulated | 8.31E-05 | 7.65E-04 |
| BPI | 0.324 | Upregulated | 1.45E-02 | 5.52E-02 |
| RNF130 | 0.324 | Upregulated | 1.67E-06 | 2.96E-05 |
| DCTN4 | 0.325 | Upregulated | 4.09E-09 | 2.27E-07 |
| FLJ34047 | 0.325 | Upregulated | 2.14E-04 | 1.70E-03 |
| PPM2C | 0.325 | Upregulated | 5.25E-07 | 1.14E-05 |
| MCTP1 | 0.326 | Upregulated | 8.37E-09 | 4.01E-07 |
| TMEM49 | 0.327 | Upregulated | 6.99E-08 | 2.23E-06 |
| C17ORF91 | 0.327 | Upregulated | 4.28E-07 | 9.71E-06 |
| NDUFAF3 | 0.327 | Upregulated | 4.73E-04 | 3.32E-03 |
| CAT | 0.327 | Upregulated | 1.18E-06 | 2.21E-05 |
| RAXL1 | 0.327 | Upregulated | 1.99E-06 | 3.41E-05 |
| MICAL2 | 0.328 | Upregulated | 1.24E-04 | 1.07E-03 |
| FKBP8 | 0.328 | Upregulated | 7.36E-04 | 4.79E-03 |
| TGFA | 0.328 | Upregulated | 4.12E-04 | 2.95E-03 |
| CRISPLD2 | 0.329 | Upregulated | 2.30E-03 | 1.24E-02 |
| SLC1A5 | 0.329 | Upregulated | 5.15E-06 | 7.57E-05 |
| CBX3 | 0.329 | Upregulated | 2.10E-07 | 5.54E-06 |
| C4ORF32 | 0.329 | Upregulated | 1.51E-08 | 6.37E-07 |
| GUK1 | 0.329 | Upregulated | 3.19E-04 | 2.38E-03 |
| UBE1C | 0.330 | Upregulated | 7.70E-10 | 6.19E-08 |
| ALDH5A1 | 0.330 | Upregulated | 4.49E-06 | 6.79E-05 |

| | | | | |
|---|---|---|---|---|
| GNA13 | 0.331 | Upregulated | 2.70E-07 | 6.69E-06 |
| SLC25A39 | 0.331 | Upregulated | 8.46E-04 | 5.38E-03 |
| JMJD1A | 0.331 | Upregulated | 5.90E-07 | 1.26E-05 |
| ZDHHC18 | 0.331 | Upregulated | 1.45E-05 | 1.79E-04 |
| MOBKL1B | 0.332 | Upregulated | 1.28E-06 | 2.37E-05 |
| RBM47 | 0.332 | Upregulated | 4.76E-05 | 4.81E-04 |
| GTF2H1 | 0.332 | Upregulated | 5.02E-07 | 1.10E-05 |
| MEGF9 | 0.332 | Upregulated | 3.04E-05 | 3.30E-04 |
| MEFV | 0.333 | Upregulated | 3.37E-05 | 3.59E-04 |
| MPO | 0.333 | Upregulated | 5.10E-03 | 2.37E-02 |
| DGAT2 | 0.335 | Upregulated | 5.17E-05 | 5.15E-04 |
| CREBBP | 0.335 | Upregulated | 4.36E-09 | 2.39E-07 |
| CSDA | 0.335 | Upregulated | 1.66E-05 | 2.00E-04 |
| NMI | 0.335 | Upregulated | 2.68E-09 | 1.63E-07 |
| GIMAP1 | 0.335 | Upregulated | 6.96E-05 | 6.59E-04 |
| CECR6 | 0.335 | Upregulated | 3.55E-05 | 3.76E-04 |
| ZNF860 | 0.335 | Upregulated | 4.18E-06 | 6.38E-05 |
| GLRX5 | 0.336 | Upregulated | 1.70E-04 | 1.40E-03 |
| LTBR | 0.336 | Upregulated | 3.81E-05 | 3.99E-04 |
| FAM175A | 0.336 | Upregulated | 5.27E-05 | 5.22E-04 |
| EIF2AK4 | 0.337 | Upregulated | 5.33E-06 | 7.80E-05 |
| RPS28 | 0.337 | Upregulated | 1.91E-04 | 1.54E-03 |
| CA2 | 0.337 | Upregulated | 9.76E-04 | 6.06E-03 |
| RAB6B | 0.337 | Upregulated | 4.43E-08 | 1.54E-06 |
| MED21 | 0.338 | Upregulated | 4.02E-10 | 3.54E-08 |
| C1ORF38 | 0.338 | Upregulated | 3.63E-07 | 8.44E-06 |
| PNPLA2 | 0.338 | Upregulated | 8.65E-05 | 7.91E-04 |
| GTF2H2 | 0.338 | Upregulated | 3.30E-05 | 3.53E-04 |
| FLJ20489 | 0.339 | Upregulated | 6.29E-04 | 4.21E-03 |
| GPRIN3 | 0.339 | Upregulated | 1.44E-04 | 1.22E-03 |
| HIAT1 | 0.340 | Upregulated | 1.12E-10 | 1.31E-08 |
| HNRPC | 0.340 | Upregulated | 1.68E-06 | 2.97E-05 |
| CPA3 | 0.341 | Upregulated | 1.83E-04 | 1.48E-03 |
| FLVCR2 | 0.341 | Upregulated | 2.17E-07 | 5.68E-06 |
| RGMA | 0.341 | Upregulated | 1.18E-10 | 1.34E-08 |
| SLC22A4 | 0.342 | Upregulated | 6.36E-04 | 4.24E-03 |
| GPR97 | 0.342 | Upregulated | 3.22E-03 | 1.63E-02 |
| CXORF21 | 0.342 | Upregulated | 8.26E-12 | 1.78E-09 |
| ADM | 0.342 | Upregulated | 9.64E-04 | 5.99E-03 |
| C13ORF7 | 0.344 | Upregulated | 2.12E-08 | 8.23E-07 |
| HLX | 0.344 | Upregulated | 5.13E-06 | 7.55E-05 |
| RNF19A | 0.344 | Upregulated | 1.37E-05 | 1.72E-04 |
| BEST1 | 0.344 | Upregulated | 1.15E-04 | 1.01E-03 |
| C14ORF11 | 0.344 | Upregulated | 6.04E-07 | 1.28E-05 |
| UBXN6 | 0.344 | Upregulated | 2.01E-03 | 1.11E-02 |
| ECHDC1 | 0.344 | Upregulated | 1.20E-08 | 5.34E-07 |
| HNRPLL | 0.346 | Upregulated | 1.11E-07 | 3.28E-06 |
| EIF1AD | 0.346 | Upregulated | 6.06E-07 | 1.29E-05 |
| MFSD11 | 0.346 | Upregulated | 1.13E-07 | 3.33E-06 |
| KLHDC8B | 0.346 | Upregulated | 9.11E-05 | 8.27E-04 |
| RAB6A | 0.347 | Upregulated | 1.36E-08 | 5.91E-07 |

| | | | | |
|---|---|---|---|---|
| STEAP4 | 0.347 | Upregulated | 2.13E-04 | 1.69E-03 |
| CYB561 | 0.347 | Upregulated | 3.17E-17 | 1.37E-13 |
| CNIH4 | 0.347 | Upregulated | 9.93E-06 | 1.31E-04 |
| SAMD14 | 0.348 | Upregulated | 1.67E-03 | 9.51E-03 |
| LMO2 | 0.348 | Upregulated | 4.93E-06 | 7.31E-05 |
| IRF2 | 0.348 | Upregulated | 1.24E-08 | 5.47E-07 |
| LSM6 | 0.348 | Upregulated | 1.34E-08 | 5.83E-07 |
| FAM73A | 0.348 | Upregulated | 1.35E-07 | 3.84E-06 |
| GHRL | 0.348 | Upregulated | 1.31E-04 | 1.13E-03 |
| WDR23 | 0.349 | Upregulated | 1.55E-08 | 6.49E-07 |
| SEC24D | 0.349 | Upregulated | 1.40E-06 | 2.56E-05 |
| CFP | 0.349 | Upregulated | 2.72E-06 | 4.45E-05 |
| USP25 | 0.349 | Upregulated | 8.50E-07 | 1.70E-05 |
| CAPZA1 | 0.350 | Upregulated | 6.59E-09 | 3.31E-07 |
| WNK1 | 0.350 | Upregulated | 6.03E-08 | 1.98E-06 |
| HOXC10 | 0.351 | Upregulated | 6.67E-06 | 9.44E-05 |
| PRRG4 | 0.351 | Upregulated | 1.13E-10 | 1.31E-08 |
| RTN4 | 0.351 | Upregulated | 1.22E-10 | 1.37E-08 |
| FLI1 | 0.351 | Upregulated | 5.72E-07 | 1.23E-05 |
| DKFZP761E198 | 0.352 | Upregulated | 1.16E-05 | 1.49E-04 |
| SOCS3 | 0.352 | Upregulated | 3.57E-06 | 5.59E-05 |
| PHLDB1 | 0.353 | Upregulated | 4.99E-05 | 5.01E-04 |
| ARHGAP25 | 0.353 | Upregulated | 6.49E-11 | 8.60E-09 |
| CREG1 | 0.354 | Upregulated | 2.91E-05 | 3.19E-04 |
| NBEAL2 | 0.354 | Upregulated | 1.45E-08 | 6.20E-07 |
| FURIN | 0.354 | Upregulated | 2.99E-06 | 4.81E-05 |
| TMEM86B | 0.355 | Upregulated | 1.60E-04 | 1.33E-03 |
| CTBS | 0.355 | Upregulated | 2.11E-07 | 5.55E-06 |
| BMP8B | 0.356 | Upregulated | 3.77E-04 | 2.74E-03 |
| ZNF223 | 0.357 | Upregulated | 1.76E-04 | 1.44E-03 |
| CCDC125 | 0.357 | Upregulated | 4.98E-06 | 7.36E-05 |
| TTC25 | 0.357 | Upregulated | 5.67E-04 | 3.87E-03 |
| APOL6 | 0.357 | Upregulated | 2.78E-07 | 6.85E-06 |
| IGSF6 | 0.357 | Upregulated | 3.01E-05 | 3.28E-04 |
| IDI1 | 0.358 | Upregulated | 1.10E-05 | 1.43E-04 |
| TAOK1 | 0.359 | Upregulated | 8.81E-07 | 1.75E-05 |
| LRG1 | 0.360 | Upregulated | 2.47E-03 | 1.32E-02 |
| SLC40A1 | 0.360 | Upregulated | 3.04E-06 | 4.88E-05 |
| DSC2 | 0.360 | Upregulated | 2.76E-04 | 2.11E-03 |
| MS4A3 | 0.361 | Upregulated | 4.98E-04 | 3.47E-03 |
| NAP1L1 | 0.361 | Upregulated | 6.42E-06 | 9.15E-05 |
| SLK | 0.361 | Upregulated | 4.80E-08 | 1.64E-06 |
| FCHO2 | 0.361 | Upregulated | 1.57E-04 | 1.31E-03 |
| MBNL2 | 0.361 | Upregulated | 6.26E-06 | 8.95E-05 |
| SESTD1 | 0.362 | Upregulated | 4.42E-11 | 6.53E-09 |
| ZRANB1 | 0.362 | Upregulated | 1.29E-08 | 5.65E-07 |
| DYSF | 0.362 | Upregulated | 1.51E-03 | 8.72E-03 |
| LCN2 | 0.364 | Upregulated | 1.15E-02 | 4.58E-02 |
| PRKAR1A | 0.364 | Upregulated | 2.54E-02 | 8.62E-02 |
| IGFBP1 | 0.365 | Upregulated | 1.03E-09 | 7.69E-08 |
| PLA2G4A | 0.365 | Upregulated | 8.93E-12 | 1.84E-09 |

| | | | | |
|---|---|---|---|---|
| PHF20L1 | 0.366 | Upregulated | 3.27E-10 | 2.96E-08 |
| RTF1 | 0.366 | Upregulated | 5.38E-06 | 7.86E-05 |
| FCGR3A | 0.366 | Upregulated | 2.92E-07 | 7.11E-06 |
| MPP1 | 0.366 | Upregulated | 5.55E-04 | 3.80E-03 |
| ASPRV1 | 0.367 | Upregulated | 3.27E-04 | 2.42E-03 |
| C16ORF69 | 0.367 | Upregulated | 2.01E-07 | 5.34E-06 |
| ECE1 | 0.367 | Upregulated | 1.09E-07 | 3.23E-06 |
| CPEB4 | 0.368 | Upregulated | 1.41E-06 | 2.56E-05 |
| TMEM119 | 0.368 | Upregulated | 4.18E-02 | 1.26E-01 |
| IL13RA1 | 0.368 | Upregulated | 3.51E-06 | 5.51E-05 |
| BCL2L1 | 0.368 | Upregulated | 2.36E-03 | 1.27E-02 |
| PLXNC1 | 0.369 | Upregulated | 2.01E-05 | 2.35E-04 |
| MTF2 | 0.369 | Upregulated | 7.42E-11 | 9.36E-09 |
| DPYD | 0.370 | Upregulated | 7.58E-08 | 2.38E-06 |
| SFMBT2 | 0.370 | Upregulated | 2.16E-05 | 2.50E-04 |
| USP49 | 0.370 | Upregulated | 1.62E-05 | 1.96E-04 |
| RNF149 | 0.370 | Upregulated | 2.77E-07 | 6.84E-06 |
| IFP38 | 0.371 | Upregulated | 6.14E-08 | 2.01E-06 |
| SACM1L | 0.371 | Upregulated | 6.00E-07 | 1.28E-05 |
| GADD45G | 0.372 | Upregulated | 3.73E-06 | 5.79E-05 |
| ETV7 | 0.372 | Upregulated | 1.06E-05 | 1.38E-04 |
| DNTTIP2 | 0.373 | Upregulated | 2.60E-07 | 6.50E-06 |
| IL18R1 | 0.373 | Upregulated | 6.26E-03 | 2.81E-02 |
| HCG27 | 0.374 | Upregulated | 1.71E-06 | 3.01E-05 |
| IL1B | 0.374 | Upregulated | 5.79E-04 | 3.93E-03 |
| WARS | 0.375 | Upregulated | 1.54E-05 | 1.88E-04 |
| RPS4Y2 | 0.375 | Upregulated | 9.40E-03 | 3.90E-02 |
| ZNF641 | 0.376 | Upregulated | 2.87E-06 | 4.66E-05 |
| SMARCD3 | 0.376 | Upregulated | 1.72E-04 | 1.41E-03 |
| SBNO2 | 0.376 | Upregulated | 3.09E-07 | 7.44E-06 |
| ITGAX | 0.376 | Upregulated | 3.87E-07 | 8.91E-06 |
| HIST1H1C | 0.377 | Upregulated | 8.13E-05 | 7.52E-04 |
| NRGN | 0.379 | Upregulated | 1.35E-03 | 7.96E-03 |
| RAB20 | 0.379 | Upregulated | 5.06E-06 | 7.46E-05 |
| NPTN | 0.379 | Upregulated | 2.23E-07 | 5.79E-06 |
| RAB24 | 0.379 | Upregulated | 1.75E-07 | 4.76E-06 |
| MYBL1 | 0.379 | Upregulated | 3.52E-05 | 3.73E-04 |
| HIST1H3D | 0.379 | Upregulated | 4.55E-06 | 6.86E-05 |
| ALPK1 | 0.379 | Upregulated | 9.01E-06 | 1.21E-04 |
| FRMD3 | 0.380 | Upregulated | 5.19E-07 | 1.13E-05 |
| PLXDC2 | 0.380 | Upregulated | 5.11E-07 | 1.12E-05 |
| C4ORF16 | 0.381 | Upregulated | 1.95E-12 | 5.69E-10 |
| SAMSN1 | 0.381 | Upregulated | 2.91E-06 | 4.72E-05 |
| VCAN | 0.381 | Upregulated | 5.08E-05 | 5.09E-04 |
| CXCR1 | 0.381 | Upregulated | 6.79E-04 | 4.49E-03 |
| NXT2 | 0.382 | Upregulated | 4.92E-07 | 1.09E-05 |
| CDC34 | 0.382 | Upregulated | 7.66E-05 | 7.15E-04 |
| PTPLAD2 | 0.383 | Upregulated | 3.17E-05 | 3.42E-04 |
| RNASEH2B | 0.383 | Upregulated | 2.47E-05 | 2.79E-04 |
| CXCL10 | 0.383 | Upregulated | 4.46E-03 | 2.13E-02 |
| CPPED1 | 0.383 | Upregulated | 2.25E-07 | 5.81E-06 |

| | | | | |
|---|---|---|---|---|
| ARID4B | 0.383 | Upregulated | 9.89E-10 | 7.45E-08 |
| CLTC | 0.383 | Upregulated | 2.89E-08 | 1.07E-06 |
| NLK | 0.384 | Upregulated | 4.65E-06 | 6.98E-05 |
| NAT13 | 0.385 | Upregulated | 2.29E-10 | 2.25E-08 |
| RPL9 | 0.386 | Upregulated | 5.89E-04 | 3.98E-03 |
| SAP30 | 0.387 | Upregulated | 4.73E-05 | 4.78E-04 |
| LAP3 | 0.388 | Upregulated | 5.56E-06 | 8.08E-05 |
| SDPR | 0.388 | Upregulated | 2.32E-05 | 2.64E-04 |
| PLAGL1 | 0.390 | Upregulated | 4.83E-11 | 6.95E-09 |
| SIPA1L2 | 0.391 | Upregulated | 5.77E-04 | 3.92E-03 |
| SLC25A37 | 0.392 | Upregulated | 1.48E-04 | 1.24E-03 |
| CAMP | 0.392 | Upregulated | 4.78E-03 | 2.25E-02 |
| GNG11 | 0.392 | Upregulated | 1.47E-05 | 1.80E-04 |
| APOL1 | 0.392 | Upregulated | 3.20E-11 | 5.10E-09 |
| RSAD2 | 0.393 | Upregulated | 2.24E-02 | 7.82E-02 |
| GBP4 | 0.393 | Upregulated | 1.55E-06 | 2.78E-05 |
| FAM83F | 0.393 | Upregulated | 5.22E-05 | 5.19E-04 |
| PAK2 | 0.393 | Upregulated | 1.85E-03 | 1.03E-02 |
| RAB11FIP1 | 0.394 | Upregulated | 8.61E-09 | 4.09E-07 |
| IFIT3 | 0.394 | Upregulated | 9.64E-03 | 3.97E-02 |
| ELANE | 0.394 | Upregulated | 1.70E-02 | 6.27E-02 |
| C6ORF204 | 0.394 | Upregulated | 1.14E-07 | 3.35E-06 |
| ANKRD9 | 0.394 | Upregulated | 2.34E-05 | 2.67E-04 |
| OSBPL8 | 0.395 | Upregulated | 2.38E-11 | 4.09E-09 |
| OSM | 0.395 | Upregulated | 3.27E-05 | 3.50E-04 |
| FPR2 | 0.395 | Upregulated | 2.35E-04 | 1.83E-03 |
| SPATA13 | 0.395 | Upregulated | 7.39E-05 | 6.95E-04 |
| TFPI | 0.395 | Upregulated | 3.65E-06 | 5.70E-05 |
| HAGH | 0.396 | Upregulated | 2.66E-04 | 2.05E-03 |
| ZBTB44 | 0.396 | Upregulated | 3.79E-09 | 2.15E-07 |
| CCR1 | 0.396 | Upregulated | 2.54E-05 | 2.86E-04 |
| LY96 | 0.397 | Upregulated | 1.00E-03 | 6.17E-03 |
| FGL2 | 0.398 | Upregulated | 1.63E-08 | 6.75E-07 |
| B3GNT5 | 0.399 | Upregulated | 8.47E-08 | 2.62E-06 |
| KEL | 0.399 | Upregulated | 1.48E-05 | 1.81E-04 |
| TCN1 | 0.400 | Upregulated | 1.12E-03 | 6.76E-03 |
| PIP5K2A | 0.401 | Upregulated | 5.29E-04 | 3.64E-03 |
| FFAR2 | 0.402 | Upregulated | 4.23E-04 | 3.02E-03 |
| PBX1 | 0.402 | Upregulated | 9.37E-06 | 1.25E-04 |
| VAMP5 | 0.403 | Upregulated | 1.42E-07 | 4.04E-06 |
| GPR146 | 0.403 | Upregulated | 3.80E-05 | 3.99E-04 |
| ZBTB11 | 0.403 | Upregulated | 8.58E-08 | 2.64E-06 |
| FAM104A | 0.404 | Upregulated | 1.76E-06 | 3.09E-05 |
| TPST1 | 0.405 | Upregulated | 3.21E-03 | 1.63E-02 |
| TSC22D1 | 0.405 | Upregulated | 1.32E-07 | 3.78E-06 |
| PINK1 | 0.405 | Upregulated | 7.37E-08 | 2.33E-06 |
| CTDSPL | 0.405 | Upregulated | 9.32E-05 | 8.43E-04 |
| LTF | 0.406 | Upregulated | 2.36E-03 | 1.27E-02 |
| MBOAT7 | 0.406 | Upregulated | 2.98E-05 | 3.25E-04 |
| SLC6A12 | 0.407 | Upregulated | 7.23E-06 | 1.01E-04 |
| LACTB | 0.408 | Upregulated | 1.04E-07 | 3.11E-06 |

| | | | | |
|---|---|---|---|---|
| GP6 | 0.408 | Upregulated | 1.18E-07 | 3.46E-06 |
| CLEC7A | 0.411 | Upregulated | 1.38E-05 | 1.72E-04 |
| FAM117A | 0.411 | Upregulated | 9.59E-09 | 4.41E-07 |
| EAF1 | 0.412 | Upregulated | 9.42E-10 | 7.17E-08 |
| LIMK2 | 0.412 | Upregulated | 3.53E-05 | 3.74E-04 |
| SMOX | 0.413 | Upregulated | 8.47E-03 | 3.59E-02 |
| SBDS | 0.414 | Upregulated | 1.02E-09 | 7.61E-08 |
| NOL10 | 0.414 | Upregulated | 3.57E-06 | 5.59E-05 |
| IL1RAP | 0.414 | Upregulated | 4.52E-08 | 1.57E-06 |
| HBQ1 | 0.415 | Upregulated | 1.62E-04 | 1.34E-03 |
| ELOVL5 | 0.416 | Upregulated | 6.96E-09 | 3.45E-07 |
| TREML1 | 0.418 | Upregulated | 2.93E-04 | 2.22E-03 |
| PHOSPHO1 | 0.419 | Upregulated | 3.26E-04 | 2.42E-03 |
| DHRS9 | 0.419 | Upregulated | 1.93E-07 | 5.16E-06 |
| SLC14A1 | 0.419 | Upregulated | 4.75E-07 | 1.06E-05 |
| HDC | 0.421 | Upregulated | 7.43E-04 | 4.83E-03 |
| FAM21A | 0.421 | Upregulated | 4.32E-09 | 2.38E-07 |
| CSF2RB | 0.422 | Upregulated | 2.03E-07 | 5.39E-06 |
| CMBL | 0.422 | Upregulated | 4.55E-04 | 3.21E-03 |
| MCTS1 | 0.422 | Upregulated | 1.12E-05 | 1.45E-04 |
| DUSP3 | 0.422 | Upregulated | 1.11E-10 | 1.30E-08 |
| POTEE | 0.423 | Upregulated | 1.52E-08 | 6.39E-07 |
| UBE2H | 0.424 | Upregulated | 3.72E-10 | 3.33E-08 |
| YPEL4 | 0.424 | Upregulated | 1.37E-07 | 3.89E-06 |
| C2 | 0.425 | Upregulated | 9.90E-08 | 2.98E-06 |
| KCTD12 | 0.427 | Upregulated | 3.12E-08 | 1.15E-06 |
| VTI1B | 0.427 | Upregulated | 9.32E-11 | 1.11E-08 |
| GRAMD1B | 0.428 | Upregulated | 2.06E-06 | 3.51E-05 |
| CD226 | 0.429 | Upregulated | 2.80E-06 | 4.56E-05 |
| SOD2 | 0.430 | Upregulated | 3.11E-05 | 3.36E-04 |
| BMP2K | 0.432 | Upregulated | 3.26E-12 | 8.43E-10 |
| NFIX | 0.434 | Upregulated | 1.18E-04 | 1.03E-03 |
| F13A1 | 0.434 | Upregulated | 6.85E-05 | 6.51E-04 |
| DEFA1 | 0.435 | Upregulated | 4.46E-03 | 2.13E-02 |
| PF4V1 | 0.438 | Upregulated | 2.03E-04 | 1.62E-03 |
| GRINA | 0.439 | Upregulated | 7.87E-07 | 1.60E-05 |
| PCAF | 0.440 | Upregulated | 2.67E-11 | 4.46E-09 |
| ZCCHC6 | 0.440 | Upregulated | 5.61E-14 | 3.54E-11 |
| SERPING1 | 0.440 | Upregulated | 8.49E-08 | 2.62E-06 |
| CD97 | 0.440 | Upregulated | 1.78E-08 | 7.17E-07 |
| TUBA4A | 0.441 | Upregulated | 9.30E-09 | 4.32E-07 |
| FAS | 0.442 | Upregulated | 6.48E-10 | 5.38E-08 |
| TANK | 0.443 | Upregulated | 2.43E-13 | 1.17E-10 |
| CIR1 | 0.444 | Upregulated | 7.33E-16 | 1.02E-12 |
| SSTR2 | 0.445 | Upregulated | 8.87E-07 | 1.76E-05 |
| STRADB | 0.446 | Upregulated | 1.45E-03 | 8.43E-03 |
| TMEM56 | 0.446 | Upregulated | 1.17E-04 | 1.02E-03 |
| USP6 | 0.447 | Upregulated | 2.58E-08 | 9.68E-07 |
| AIM2 | 0.447 | Upregulated | 6.82E-11 | 8.90E-09 |
| SLC2A14 | 0.448 | Upregulated | 6.01E-05 | 5.84E-04 |
| C16ORF7 | 0.449 | Upregulated | 9.10E-09 | 4.25E-07 |

| | | | | |
|---|---|---|---|---|
| SIGLEC14 | 0.451 | Upregulated | 4.82E-02 | 1.41E-01 |
| C4ORF18 | 0.452 | Upregulated | 1.63E-05 | 1.97E-04 |
| TLR2 | 0.453 | Upregulated | 5.69E-05 | 5.57E-04 |
| TIMM10 | 0.454 | Upregulated | 1.93E-04 | 1.56E-03 |
| DAPP1 | 0.457 | Upregulated | 1.25E-13 | 6.77E-11 |
| WDR40A | 0.457 | Upregulated | 1.51E-04 | 1.27E-03 |
| ITGB3 | 0.459 | Upregulated | 6.88E-05 | 6.53E-04 |
| HIST2H2AB | 0.459 | Upregulated | 1.54E-06 | 2.77E-05 |
| CACNA1E | 0.459 | Upregulated | 4.58E-04 | 3.23E-03 |
| GPR109B | 0.460 | Upregulated | 5.76E-08 | 1.91E-06 |
| NOD2 | 0.461 | Upregulated | 4.75E-08 | 1.63E-06 |
| RAD21 | 0.461 | Upregulated | 4.08E-12 | 1.01E-09 |
| ANXA4 | 0.461 | Upregulated | 9.68E-07 | 1.88E-05 |
| FER1L3 | 0.462 | Upregulated | 3.48E-06 | 5.48E-05 |
| C16ORF35 | 0.462 | Upregulated | 7.24E-05 | 6.82E-04 |
| SPOPL | 0.463 | Upregulated | 1.24E-08 | 5.45E-07 |
| SH3BGRL2 | 0.464 | Upregulated | 1.30E-05 | 1.64E-04 |
| DEFA4 | 0.465 | Upregulated | 9.54E-03 | 3.94E-02 |
| KLF1 | 0.465 | Upregulated | 1.32E-05 | 1.66E-04 |
| EMB | 0.466 | Upregulated | 2.90E-11 | 4.75E-09 |
| IDO1 | 0.468 | Upregulated | 1.44E-03 | 8.39E-03 |
| ADIPOR1 | 0.469 | Upregulated | 1.49E-05 | 1.83E-04 |
| MXI1 | 0.469 | Upregulated | 2.82E-05 | 3.11E-04 |
| FBXO38 | 0.470 | Upregulated | 1.00E-09 | 7.51E-08 |
| PDCD1LG2 | 0.470 | Upregulated | 2.57E-07 | 6.45E-06 |
| MGC13057 | 0.470 | Upregulated | 4.85E-05 | 4.89E-04 |
| NCOA1 | 0.471 | Upregulated | 1.64E-09 | 1.08E-07 |
| FAM8A1 | 0.471 | Upregulated | 7.81E-11 | 9.68E-09 |
| VWCE | 0.471 | Upregulated | 2.17E-03 | 1.18E-02 |
| ACTR3 | 0.471 | Upregulated | 1.47E-07 | 4.15E-06 |
| SORT1 | 0.474 | Upregulated | 8.52E-07 | 1.70E-05 |
| KRT1 | 0.475 | Upregulated | 2.76E-02 | 9.20E-02 |
| KIFC3 | 0.476 | Upregulated | 2.85E-10 | 2.66E-08 |
| CD46 | 0.477 | Upregulated | 4.99E-11 | 7.12E-09 |
| PRDM1 | 0.477 | Upregulated | 5.97E-11 | 8.06E-09 |
| LRRK2 | 0.480 | Upregulated | 1.49E-09 | 1.00E-07 |
| RRAGD | 0.483 | Upregulated | 7.06E-08 | 2.24E-06 |
| GSTA5 | 0.484 | Upregulated | 6.86E-05 | 6.52E-04 |
| FCGR3B | 0.484 | Upregulated | 2.06E-05 | 2.40E-04 |
| HPSE | 0.484 | Upregulated | 1.57E-07 | 4.36E-06 |
| SNCA | 0.486 | Upregulated | 3.85E-03 | 1.89E-02 |
| RHAG | 0.486 | Upregulated | 9.29E-06 | 1.24E-04 |
| RAP1BL | 0.486 | Upregulated | 1.03E-06 | 1.97E-05 |
| CARD17 | 0.486 | Upregulated | 4.59E-07 | 1.03E-05 |
| NCF2 | 0.487 | Upregulated | 8.03E-10 | 6.40E-08 |
| SLC6A8 | 0.489 | Upregulated | 6.10E-05 | 5.91E-04 |
| MYOF | 0.489 | Upregulated | 2.05E-06 | 3.49E-05 |
| HSPA1A | 0.489 | Upregulated | 1.73E-05 | 2.07E-04 |
| RHD | 0.490 | Upregulated | 2.23E-04 | 1.76E-03 |
| TNFAIP2 | 0.490 | Upregulated | 7.43E-09 | 3.65E-07 |
| KLHL2 | 0.491 | Upregulated | 3.66E-07 | 8.49E-06 |

| | | | | |
|---|---|---|---|---|
| TXNL1 | 0.491 | Upregulated | 3.61E-07 | 8.41E-06 |
| XK | 0.493 | Upregulated | 7.39E-05 | 6.95E-04 |
| METTL7A | 0.493 | Upregulated | 1.14E-08 | 5.15E-07 |
| GNG10 | 0.493 | Upregulated | 2.52E-08 | 9.49E-07 |
| FECH | 0.495 | Upregulated | 3.88E-05 | 4.06E-04 |
| FAM129A | 0.496 | Upregulated | 1.71E-07 | 4.69E-06 |
| MICALCL | 0.496 | Upregulated | 6.79E-07 | 1.41E-05 |
| BIN2 | 0.503 | Upregulated | 7.40E-09 | 3.64E-07 |
| SPRYD3 | 0.505 | Upregulated | 7.04E-07 | 1.45E-05 |
| CCDC52 | 0.505 | Upregulated | 4.70E-08 | 1.62E-06 |
| C14ORF45 | 0.506 | Upregulated | 1.18E-07 | 3.46E-06 |
| GPR65 | 0.506 | Upregulated | 1.01E-13 | 5.69E-11 |
| RNF182 | 0.507 | Upregulated | 7.02E-04 | 4.61E-03 |
| DEFA3 | 0.507 | Upregulated | 4.57E-03 | 2.17E-02 |
| RAB3IL1 | 0.508 | Upregulated | 1.59E-04 | 1.33E-03 |
| TSPAN5 | 0.508 | Upregulated | 1.17E-04 | 1.02E-03 |
| HIF1A | 0.509 | Upregulated | 3.54E-08 | 1.27E-06 |
| INDO | 0.510 | Upregulated | 7.75E-04 | 5.00E-03 |
| IL27 | 0.511 | Upregulated | 1.75E-06 | 3.07E-05 |
| ANP32A | 0.511 | Upregulated | 1.15E-10 | 1.32E-08 |
| KCNJ2 | 0.511 | Upregulated | 1.56E-07 | 4.35E-06 |
| PPIB | 0.514 | Upregulated | 3.21E-08 | 1.17E-06 |
| TUBB2A | 0.515 | Upregulated | 1.31E-03 | 7.73E-03 |
| PPBP | 0.519 | Upregulated | 2.05E-06 | 3.50E-05 |
| FYB | 0.519 | Upregulated | 4.87E-15 | 4.56E-12 |
| C15ORF29 | 0.519 | Upregulated | 3.56E-13 | 1.60E-10 |
| IFIT1L | 0.521 | Upregulated | 8.08E-03 | 3.46E-02 |
| MYADM | 0.521 | Upregulated | 6.92E-07 | 1.43E-05 |
| ITPRIPL2 | 0.522 | Upregulated | 5.86E-09 | 3.03E-07 |
| RBM38 | 0.527 | Upregulated | 8.85E-07 | 1.76E-05 |
| PPP2CB | 0.528 | Upregulated | 6.71E-10 | 5.52E-08 |
| SLAMF8 | 0.528 | Upregulated | 8.46E-10 | 6.62E-08 |
| CEACAM6 | 0.529 | Upregulated | 5.33E-04 | 3.67E-03 |
| HDAC4 | 0.529 | Upregulated | 3.04E-08 | 1.12E-06 |
| C1QC | 0.529 | Upregulated | 3.39E-04 | 2.50E-03 |
| BAGE5 | 0.530 | Upregulated | 8.46E-12 | 1.80E-09 |
| TTRAP | 0.533 | Upregulated | 7.66E-10 | 6.17E-08 |
| VPS26 | 0.535 | Upregulated | 8.72E-10 | 6.79E-08 |
| WSB1 | 0.535 | Upregulated | 2.14E-11 | 3.75E-09 |
| GIMAP2 | 0.536 | Upregulated | 1.21E-08 | 5.36E-07 |
| MYL9 | 0.542 | Upregulated | 2.29E-05 | 2.62E-04 |
| HMGB1L1 | 0.544 | Upregulated | 1.60E-10 | 1.70E-08 |
| PTGES3 | 0.544 | Upregulated | 1.42E-07 | 4.04E-06 |
| FAM44A | 0.545 | Upregulated | 9.00E-13 | 3.26E-10 |
| SELP | 0.546 | Upregulated | 1.39E-08 | 5.99E-07 |
| SFRS11 | 0.546 | Upregulated | 9.49E-10 | 7.20E-08 |
| TMEM158 | 0.547 | Upregulated | 2.23E-05 | 2.57E-04 |
| S100A12 | 0.550 | Upregulated | 1.10E-03 | 6.70E-03 |
| PROS1 | 0.550 | Upregulated | 2.23E-07 | 5.79E-06 |
| ADI1 | 0.551 | Upregulated | 1.24E-09 | 8.85E-08 |
| C1QB | 0.552 | Upregulated | 5.58E-04 | 3.81E-03 |

| | | | | |
|---|---|---|---|---|
| HIST1H2BC | 0.553 | Upregulated | 2.78E-09 | 1.67E-07 |
| PTGS2 | 0.556 | Upregulated | 2.08E-08 | 8.12E-07 |
| ILK | 0.556 | Upregulated | 2.64E-10 | 2.52E-08 |
| EPB49 | 0.557 | Upregulated | 7.00E-05 | 6.63E-04 |
| GK | 0.558 | Upregulated | 1.63E-07 | 4.51E-06 |
| CYP4F3 | 0.558 | Upregulated | 5.61E-06 | 8.14E-05 |
| GMPR | 0.562 | Upregulated | 1.34E-04 | 1.15E-03 |
| KIAA1033 | 0.564 | Upregulated | 2.40E-08 | 9.13E-07 |
| HECA | 0.566 | Upregulated | 4.72E-11 | 6.87E-09 |
| HIST1H2BE | 0.566 | Upregulated | 9.19E-10 | 7.06E-08 |
| AQP10 | 0.569 | Upregulated | 8.06E-06 | 1.11E-04 |
| RPL23 | 0.570 | Upregulated | 2.73E-05 | 3.03E-04 |
| DEFA1B | 0.571 | Upregulated | 2.62E-03 | 1.38E-02 |
| SOCS1 | 0.572 | Upregulated | 2.02E-09 | 1.29E-07 |
| PLEK2 | 0.574 | Upregulated | 2.54E-06 | 4.20E-05 |
| TRIM58 | 0.575 | Upregulated | 1.32E-04 | 1.13E-03 |
| FAM49B | 0.576 | Upregulated | 1.42E-09 | 9.68E-08 |
| PDZK1IP1 | 0.577 | Upregulated | 1.69E-04 | 1.40E-03 |
| TMCC2 | 0.579 | Upregulated | 1.20E-05 | 1.54E-04 |
| BNIP3L | 0.585 | Upregulated | 1.89E-08 | 7.50E-07 |
| FLJ20309 | 0.587 | Upregulated | 8.00E-08 | 2.50E-06 |
| SELPLG | 0.589 | Upregulated | 2.07E-11 | 3.66E-09 |
| SNX10 | 0.590 | Upregulated | 7.59E-10 | 6.12E-08 |
| ANXA3 | 0.592 | Upregulated | 9.25E-05 | 8.38E-04 |
| IGF2BP2 | 0.593 | Upregulated | 2.09E-06 | 3.55E-05 |
| ERGIC2 | 0.595 | Upregulated | 8.05E-09 | 3.86E-07 |
| ALDH1A1 | 0.595 | Upregulated | 1.53E-08 | 6.42E-07 |
| CHPT1 | 0.599 | Upregulated | 1.96E-08 | 7.71E-07 |
| GPR175 | 0.601 | Upregulated | 2.48E-05 | 2.80E-04 |
| PSG9 | 0.601 | Upregulated | 2.26E-07 | 5.83E-06 |
| ZFP36L1 | 0.601 | Upregulated | 1.27E-09 | 8.94E-08 |
| TNS1 | 0.602 | Upregulated | 7.65E-05 | 7.14E-04 |
| GYPB | 0.602 | Upregulated | 1.19E-03 | 7.16E-03 |
| FCGR2C | 0.604 | Upregulated | 1.86E-09 | 1.20E-07 |
| KCNJ15 | 0.604 | Upregulated | 1.38E-08 | 5.97E-07 |
| CLK1 | 0.606 | Upregulated | 1.59E-09 | 1.06E-07 |
| TMOD1 | 0.609 | Upregulated | 6.91E-05 | 6.56E-04 |
| ITGA2B | 0.612 | Upregulated | 2.83E-06 | 4.60E-05 |
| GBP1 | 0.613 | Upregulated | 3.48E-09 | 2.01E-07 |
| SESN3 | 0.615 | Upregulated | 1.25E-05 | 1.59E-04 |
| CEACAM1 | 0.616 | Upregulated | 4.16E-07 | 9.49E-06 |
| CEACAM8 | 0.621 | Upregulated | 1.51E-04 | 1.27E-03 |
| SIAH2 | 0.622 | Upregulated | 4.50E-09 | 2.44E-07 |
| ACSL1 | 0.622 | Upregulated | 1.02E-07 | 3.07E-06 |
| P2RY13 | 0.624 | Upregulated | 1.81E-10 | 1.87E-08 |
| RNF213 | 0.626 | Upregulated | 7.71E-09 | 3.75E-07 |
| PSTPIP2 | 0.626 | Upregulated | 2.94E-10 | 2.74E-08 |
| AHSP | 0.628 | Upregulated | 2.36E-04 | 1.84E-03 |
| SPAST | 0.632 | Upregulated | 2.70E-10 | 2.56E-08 |
| CAST | 0.635 | Upregulated | 9.11E-17 | 2.43E-13 |
| WSB2 | 0.637 | Upregulated | 5.59E-11 | 7.79E-09 |

| | | | | |
|---|---|---|---|---|
| CASP5 | 0.639 | Upregulated | 5.51E-07 | 1.19E-05 |
| Septin 7 | 0.646 | Upregulated | 2.52E-09 | 1.55E-07 |
| KREMEN1 | 0.653 | Upregulated | 7.23E-09 | 3.57E-07 |
| GBP3 | 0.655 | Upregulated | 6.14E-12 | 1.41E-09 |
| C1GALT1 | 0.677 | Upregulated | 1.09E-16 | 2.51E-13 |
| DPYSL5 | 0.677 | Upregulated | 3.37E-05 | 3.59E-04 |
| HBD | 0.678 | Upregulated | 1.09E-05 | 1.41E-04 |
| GPR109A | 0.679 | Upregulated | 6.32E-11 | 8.43E-09 |
| C18ORF32 | 0.691 | Upregulated | 2.10E-14 | 1.74E-11 |
| C20ORF108 | 0.693 | Upregulated | 2.93E-06 | 4.73E-05 |
| CA1 | 0.696 | Upregulated | 1.29E-03 | 7.66E-03 |
| LHFPL2 | 0.699 | Upregulated | 6.48E-16 | 9.37E-13 |
| HLA-G | 0.699 | Upregulated | 4.71E-15 | 4.54E-12 |
| F2RL1 | 0.702 | Upregulated | 1.38E-13 | 7.16E-11 |
| CD164 | 0.708 | Upregulated | 5.03E-11 | 7.15E-09 |
| RAB33B | 0.735 | Upregulated | 3.12E-09 | 1.84E-07 |
| FLJ20273 | 0.740 | Upregulated | 3.14E-11 | 5.04E-09 |
| PIK3AP1 | 0.748 | Upregulated | 1.14E-10 | 1.31E-08 |
| EPB42 | 0.749 | Upregulated | 3.05E-05 | 3.30E-04 |
| APOBEC3A | 0.760 | Upregulated | 1.56E-12 | 4.89E-10 |
| LGALS3 | 0.769 | Upregulated | 3.28E-12 | 8.43E-10 |
| RAP1GAP | 0.778 | Upregulated | 1.81E-03 | 1.02E-02 |
| PTMA | 0.795 | Upregulated | 1.20E-08 | 5.33E-07 |
| BLVRB | 0.796 | Upregulated | 3.53E-11 | 5.49E-09 |
| CD274 | 0.800 | Upregulated | 2.79E-10 | 2.62E-08 |
| GBP6 | 0.801 | Upregulated | 1.19E-17 | 6.87E-14 |
| SLC4A1 | 0.805 | Upregulated | 8.17E-07 | 1.65E-05 |
| P2RY14 | 0.811 | Upregulated | 2.28E-14 | 1.78E-11 |
| SELENBP1 | 0.828 | Upregulated | 3.45E-05 | 3.66E-04 |
| MBNL3 | 0.844 | Upregulated | 2.00E-11 | 3.57E-09 |
| OSBP2 | 0.883 | Upregulated | 3.33E-07 | 7.90E-06 |
| GBP5 | 0.904 | Upregulated | 6.32E-17 | 1.87E-13 |
| HBE1 | 0.953 | Upregulated | 1.11E-10 | 1.30E-08 |
| TLR1 | 0.955 | Upregulated | 5.02E-13 | 2.07E-10 |
| FCGR1B | 0.958 | Upregulated | 9.36E-13 | 3.31E-10 |
| SERPINA13 | 0.963 | Upregulated | 1.11E-06 | 2.11E-05 |
| BATF2 | 0.964 | Upregulated | 6.37E-14 | 3.95E-11 |
| ANKRD22 | 1.009 | Upregulated | 9.40E-12 | 1.90E-09 |
| ALAS2 | 1.009 | Upregulated | 5.89E-08 | 1.95E-06 |
| FAM26F | 1.016 | Upregulated | 1.20E-19 | 4.18E-15 |
| FCGR1A | 1.036 | Upregulated | 1.66E-13 | 8.35E-11 |
| FCGR1C | 1.156 | Upregulated | 2.10E-17 | 1.04E-13 |

**Supplementary Table S2c. Differentially expressed genes_Malawi**

| Gene | logFC | Direction of expression | P.Value | adj.P.Val |
|---|---|---|---|---|
| LRRN3 | -0.506 | Downregulated | 1.31E-03 | 1.24E-01 |
| RPL14 | -0.454 | Downregulated | 7.86E-03 | 2.36E-01 |
| PTMA | -0.450 | Downregulated | 2.24E-02 | 3.27E-01 |
| HRK | -0.425 | Downregulated | 7.50E-04 | 9.89E-02 |
| HSPA1A | -0.414 | Downregulated | 1.65E-02 | 2.94E-01 |
| OLIG1 | -0.387 | Downregulated | 2.26E-03 | 1.54E-01 |
| METTL7A | -0.375 | Downregulated | 3.02E-03 | 1.72E-01 |
| CLK1 | -0.372 | Downregulated | 5.15E-03 | 2.04E-01 |
| ACTR3 | -0.368 | Downregulated | 3.95E-03 | 1.89E-01 |
| MBNL2 | -0.367 | Downregulated | 1.38E-03 | 1.25E-01 |
| PTGES3 | -0.340 | Downregulated | 1.06E-02 | 2.55E-01 |
| RPS28 | -0.338 | Downregulated | 3.45E-03 | 1.77E-01 |
| HNRPC | -0.337 | Downregulated | 2.36E-03 | 1.56E-01 |
| PPIB | -0.332 | Downregulated | 2.12E-02 | 3.20E-01 |
| CLECL1 | -0.328 | Downregulated | 3.66E-02 | 3.85E-01 |
| CACNA2D3 | -0.325 | Downregulated | 2.76E-02 | 3.50E-01 |
| Septin 7 | -0.325 | Downregulated | 2.06E-02 | 3.16E-01 |
| ADI1 | -0.324 | Downregulated | 1.36E-02 | 2.73E-01 |
| TXNL1 | -0.320 | Downregulated | 2.08E-02 | 3.17E-01 |
| PTPLAD2 | -0.317 | Downregulated | 9.24E-03 | 2.46E-01 |
| C13ORF27 | -0.317 | Downregulated | 5.76E-04 | 9.02E-02 |
| CPOX | -0.316 | Downregulated | 6.29E-05 | 3.31E-02 |
| FAM129C | -0.314 | Downregulated | 1.07E-02 | 2.55E-01 |
| C12ORF23 | -0.313 | Downregulated | 9.41E-03 | 2.47E-01 |
| RPN2 | -0.310 | Downregulated | 1.46E-02 | 2.80E-01 |
| SACM1L | -0.310 | Downregulated | 4.36E-03 | 1.95E-01 |
| HSPH1 | -0.308 | Downregulated | 1.80E-03 | 1.41E-01 |
| SFRS11 | -0.307 | Downregulated | 9.44E-03 | 2.47E-01 |
| KBTBD7 | -0.307 | Downregulated | 7.93E-03 | 2.37E-01 |
| FAM98A | -0.305 | Downregulated | 3.18E-03 | 1.75E-01 |
| RAP1BL | -0.302 | Downregulated | 1.77E-02 | 3.01E-01 |
| FAM179B | -0.301 | Downregulated | 1.08E-02 | 2.56E-01 |
| IFP38 | -0.299 | Downregulated | 5.71E-03 | 2.11E-01 |
| RASGRP1 | -0.297 | Downregulated | 3.90E-03 | 1.88E-01 |
| MEX3C | -0.296 | Downregulated | 1.10E-03 | 1.18E-01 |
| HECA | -0.295 | Downregulated | 1.19E-02 | 2.60E-01 |
| C8ORF13 | -0.295 | Downregulated | 4.37E-03 | 1.95E-01 |
| ZFP36L1 | -0.292 | Downregulated | 4.01E-02 | 3.95E-01 |
| SPIB | -0.292 | Downregulated | 1.83E-02 | 3.04E-01 |
| FCRL3 | -0.292 | Downregulated | 4.72E-03 | 1.98E-01 |
| EIF3A | -0.292 | Downregulated | 1.14E-03 | 1.19E-01 |
| OSBPL10 | -0.290 | Downregulated | 3.46E-02 | 3.77E-01 |
| C13ORF18 | -0.290 | Downregulated | 4.33E-04 | 7.91E-02 |
| HMGB1L1 | -0.289 | Downregulated | 1.42E-02 | 2.77E-01 |
| ZNF281 | -0.287 | Downregulated | 5.89E-03 | 2.13E-01 |
| SPAST | -0.286 | Downregulated | 1.99E-02 | 3.12E-01 |
| Septin 11 | -0.286 | Downregulated | 6.92E-04 | 9.64E-02 |

| | | | | |
|---|---|---|---|---|
| SLC39A10 | -0.285 | Downregulated | 2.14E-03 | 1.51E-01 |
| VPS26 | -0.284 | Downregulated | 1.32E-02 | 2.71E-01 |
| BUB3 | -0.282 | Downregulated | 1.21E-03 | 1.21E-01 |
| HSPA4 | -0.282 | Downregulated | 4.85E-03 | 2.01E-01 |
| EFCBP1 | -0.282 | Downregulated | 2.09E-02 | 3.17E-01 |
| PMS1 | -0.282 | Downregulated | 9.46E-03 | 2.47E-01 |
| TMEM106A | -0.282 | Downregulated | 1.91E-02 | 3.08E-01 |
| S1PR1 | -0.281 | Downregulated | 8.23E-03 | 2.40E-01 |
| NT5E | -0.280 | Downregulated | 4.06E-02 | 3.96E-01 |
| SPIN4 | -0.278 | Downregulated | 7.92E-04 | 1.01E-01 |
| G3BP1 | -0.277 | Downregulated | 5.81E-03 | 2.13E-01 |
| NUBPL | -0.275 | Downregulated | 1.14E-02 | 2.59E-01 |
| FLI1 | -0.274 | Downregulated | 4.10E-03 | 1.90E-01 |
| FCHO2 | -0.273 | Downregulated | 4.57E-02 | 4.11E-01 |
| FAM167A | -0.271 | Downregulated | 5.33E-03 | 2.06E-01 |
| CD24 | -0.270 | Downregulated | 8.46E-03 | 2.42E-01 |
| SBDS | -0.270 | Downregulated | 1.11E-02 | 2.58E-01 |
| CCDC125 | -0.267 | Downregulated | 8.43E-03 | 2.41E-01 |
| SFRS12 | -0.266 | Downregulated | 9.94E-04 | 1.12E-01 |
| GIMAP1 | -0.265 | Downregulated | 4.76E-02 | 4.17E-01 |
| SSTR2 | -0.265 | Downregulated | 2.02E-02 | 3.14E-01 |
| SLC25A39 | 0.266 | Upregulated | 2.23E-02 | 3.26E-01 |
| SPARC | 0.266 | Upregulated | 9.61E-03 | 2.47E-01 |
| PSME2 | 0.266 | Upregulated | 4.03E-05 | 2.53E-02 |
| DDEF2 | 0.266 | Upregulated | 9.71E-05 | 3.96E-02 |
| NGFRAP1 | 0.269 | Upregulated | 4.55E-03 | 1.97E-01 |
| TMSB15A | 0.270 | Upregulated | 8.61E-05 | 3.93E-02 |
| GALM | 0.270 | Upregulated | 1.28E-02 | 2.67E-01 |
| GYPC | 0.270 | Upregulated | 3.96E-02 | 3.94E-01 |
| SELP | 0.270 | Upregulated | 1.21E-02 | 2.61E-01 |
| IL15 | 0.271 | Upregulated | 2.76E-05 | 2.32E-02 |
| PARP14 | 0.271 | Upregulated | 4.64E-03 | 1.97E-01 |
| TNFAIP2 | 0.274 | Upregulated | 8.89E-03 | 2.45E-01 |
| DHRS9 | 0.274 | Upregulated | 2.49E-03 | 1.61E-01 |
| ECHDC3 | 0.274 | Upregulated | 3.79E-02 | 3.89E-01 |
| MAX | 0.275 | Upregulated | 8.99E-05 | 3.95E-02 |
| IGSF6 | 0.276 | Upregulated | 7.89E-03 | 2.37E-01 |
| GRAMD1B | 0.276 | Upregulated | 3.04E-03 | 1.72E-01 |
| PLAUR | 0.278 | Upregulated | 4.03E-03 | 1.90E-01 |
| IL12RB1 | 0.279 | Upregulated | 3.65E-03 | 1.81E-01 |
| SOCS1 | 0.279 | Upregulated | 2.62E-03 | 1.63E-01 |
| TSPY3 | 0.279 | Upregulated | 1.37E-05 | 1.59E-02 |
| VSIG4 | 0.282 | Upregulated | 2.82E-03 | 1.67E-01 |
| CREG1 | 0.282 | Upregulated | 2.33E-02 | 3.32E-01 |
| ARHGEF10L | 0.282 | Upregulated | 1.18E-06 | 2.80E-03 |
| LY6G6F | 0.282 | Upregulated | 8.90E-03 | 2.45E-01 |
| TGM2 | 0.284 | Upregulated | 4.75E-02 | 4.17E-01 |
| JAM3 | 0.286 | Upregulated | 5.86E-03 | 2.13E-01 |
| FLJ20309 | 0.286 | Upregulated | 3.57E-02 | 3.81E-01 |
| GM2A | 0.287 | Upregulated | 1.47E-03 | 1.28E-01 |
| CD151 | 0.287 | Upregulated | 3.67E-04 | 7.34E-02 |

| | | | | |
|---|---|---|---|---|
| CXCL9 | 0.289 | Upregulated | 4.62E-03 | 1.97E-01 |
| ITGB5 | 0.289 | Upregulated | 1.91E-03 | 1.46E-01 |
| ABLIM3 | 0.290 | Upregulated | 5.08E-04 | 8.47E-02 |
| FAM21A | 0.290 | Upregulated | 4.05E-02 | 3.96E-01 |
| ETV7 | 0.292 | Upregulated | 8.13E-04 | 1.03E-01 |
| GK | 0.294 | Upregulated | 8.43E-03 | 2.41E-01 |
| GNG11 | 0.295 | Upregulated | 1.61E-02 | 2.91E-01 |
| HIST1H2BG | 0.295 | Upregulated | 6.52E-03 | 2.19E-01 |
| C10ORF82 | 0.295 | Upregulated | 1.10E-05 | 1.32E-02 |
| HIST1H3H | 0.296 | Upregulated | 2.29E-02 | 3.30E-01 |
| FGL2 | 0.296 | Upregulated | 5.28E-04 | 8.63E-02 |
| MT1A | 0.296 | Upregulated | 1.03E-02 | 2.52E-01 |
| HOXC10 | 0.296 | Upregulated | 1.12E-02 | 2.58E-01 |
| OASL | 0.297 | Upregulated | 4.55E-02 | 4.11E-01 |
| HSD3B7 | 0.298 | Upregulated | 5.20E-03 | 2.04E-01 |
| EPB41L3 | 0.299 | Upregulated | 4.26E-03 | 1.93E-01 |
| RGS10 | 0.303 | Upregulated | 2.09E-03 | 1.50E-01 |
| CTDSPL | 0.303 | Upregulated | 1.15E-02 | 2.59E-01 |
| NT5M | 0.305 | Upregulated | 2.82E-03 | 1.67E-01 |
| CTSA | 0.306 | Upregulated | 2.92E-04 | 6.66E-02 |
| C18ORF10 | 0.307 | Upregulated | 3.55E-02 | 3.80E-01 |
| KLHDC8B | 0.307 | Upregulated | 7.88E-03 | 2.37E-01 |
| BEND7 | 0.308 | Upregulated | 3.62E-04 | 7.34E-02 |
| SORT1 | 0.308 | Upregulated | 1.85E-03 | 1.43E-01 |
| ALOX12 | 0.310 | Upregulated | 7.88E-03 | 2.37E-01 |
| SCO2 | 0.313 | Upregulated | 9.99E-04 | 1.12E-01 |
| ECGF1 | 0.313 | Upregulated | 8.51E-05 | 3.93E-02 |
| SAMD14 | 0.313 | Upregulated | 9.56E-03 | 2.47E-01 |
| FAH | 0.314 | Upregulated | 4.66E-04 | 8.06E-02 |
| LHFPL2 | 0.315 | Upregulated | 4.83E-04 | 8.13E-02 |
| ITGB3 | 0.318 | Upregulated | 1.29E-02 | 2.68E-01 |
| HIST1H4H | 0.318 | Upregulated | 2.76E-02 | 3.50E-01 |
| SAMD4A | 0.319 | Upregulated | 3.96E-03 | 1.89E-01 |
| CCDC52 | 0.323 | Upregulated | 1.49E-02 | 2.81E-01 |
| GNA12 | 0.324 | Upregulated | 1.30E-02 | 2.68E-01 |
| F2RL1 | 0.326 | Upregulated | 9.70E-03 | 2.48E-01 |
| OBFC1 | 0.326 | Upregulated | 6.09E-08 | 5.28E-04 |
| IFITM3 | 0.326 | Upregulated | 5.10E-03 | 2.04E-01 |
| VAMP5 | 0.327 | Upregulated | 3.59E-05 | 2.46E-02 |
| DPM2 | 0.327 | Upregulated | 1.33E-02 | 2.71E-01 |
| FBXO6 | 0.327 | Upregulated | 1.17E-03 | 1.19E-01 |
| SDPR | 0.329 | Upregulated | 9.26E-03 | 2.46E-01 |
| FRMD3 | 0.330 | Upregulated | 6.76E-04 | 9.54E-02 |
| APOL1 | 0.333 | Upregulated | 1.19E-04 | 4.42E-02 |
| CD226 | 0.333 | Upregulated | 8.38E-03 | 2.41E-01 |
| STAT1 | 0.334 | Upregulated | 1.81E-04 | 4.98E-02 |
| PF4V1 | 0.336 | Upregulated | 3.53E-02 | 3.79E-01 |
| GADD45G | 0.336 | Upregulated | 2.07E-04 | 5.46E-02 |
| GP6 | 0.336 | Upregulated | 1.76E-04 | 4.93E-02 |
| FLJ20489 | 0.337 | Upregulated | 9.36E-03 | 2.47E-01 |
| ACRBP | 0.338 | Upregulated | 5.78E-03 | 2.12E-01 |

| | | | | |
|---|---|---|---|---|
| TYMP | 0.340 | Upregulated | 1.12E-04 | 4.30E-02 |
| GBP2 | 0.340 | Upregulated | 4.21E-07 | 1.82E-03 |
| GCH1 | 0.343 | Upregulated | 9.96E-04 | 1.12E-01 |
| CASP5 | 0.344 | Upregulated | 4.24E-02 | 4.02E-01 |
| TIMM10 | 0.344 | Upregulated | 1.16E-02 | 2.59E-01 |
| IFIT2 | 0.346 | Upregulated | 1.91E-02 | 3.08E-01 |
| MXI1 | 0.350 | Upregulated | 3.50E-02 | 3.78E-01 |
| HIST1H2BJ | 0.350 | Upregulated | 2.25E-03 | 1.54E-01 |
| C2ORF88 | 0.357 | Upregulated | 1.02E-03 | 1.12E-01 |
| SH3BGRL2 | 0.362 | Upregulated | 5.39E-03 | 2.07E-01 |
| ALDH1A1 | 0.364 | Upregulated | 6.32E-04 | 9.41E-02 |
| UBE2L6 | 0.364 | Upregulated | 3.46E-04 | 7.31E-02 |
| CLDN5 | 0.368 | Upregulated | 8.31E-03 | 2.41E-01 |
| RTP4 | 0.370 | Upregulated | 4.41E-03 | 1.95E-01 |
| ESAM | 0.370 | Upregulated | 5.04E-03 | 2.04E-01 |
| PVALB | 0.370 | Upregulated | 8.37E-03 | 2.41E-01 |
| IDO1 | 0.374 | Upregulated | 3.35E-02 | 3.75E-01 |
| TFPI | 0.378 | Upregulated | 8.80E-04 | 1.06E-01 |
| KEL | 0.378 | Upregulated | 1.80E-02 | 3.03E-01 |
| KLF1 | 0.378 | Upregulated | 3.66E-02 | 3.85E-01 |
| EPSTI1 | 0.379 | Upregulated | 3.25E-03 | 1.76E-01 |
| NRGN | 0.380 | Upregulated | 7.48E-03 | 2.31E-01 |
| KIFC3 | 0.380 | Upregulated | 6.63E-05 | 3.33E-02 |
| SERPING1 | 0.382 | Upregulated | 6.15E-04 | 9.27E-02 |
| ITLN1 | 0.383 | Upregulated | 3.83E-02 | 3.91E-01 |
| TSPAN9 | 0.383 | Upregulated | 3.61E-03 | 1.81E-01 |
| PLEK2 | 0.390 | Upregulated | 2.48E-02 | 3.39E-01 |
| NDUFAF3 | 0.391 | Upregulated | 9.84E-05 | 3.97E-02 |
| FBXO7 | 0.396 | Upregulated | 3.10E-02 | 3.65E-01 |
| XK | 0.397 | Upregulated | 4.24E-02 | 4.02E-01 |
| SLC6A12 | 0.399 | Upregulated | 2.35E-05 | 2.26E-02 |
| MT2A | 0.400 | Upregulated | 2.23E-03 | 1.54E-01 |
| PPBP | 0.404 | Upregulated | 2.47E-03 | 1.60E-01 |
| GMPR | 0.412 | Upregulated | 4.03E-02 | 3.95E-01 |
| MGC13057 | 0.412 | Upregulated | 1.64E-02 | 2.93E-01 |
| FCGR1C | 0.414 | Upregulated | 2.14E-03 | 1.51E-01 |
| APOL6 | 0.416 | Upregulated | 5.03E-06 | 7.93E-03 |
| CD274 | 0.422 | Upregulated | 1.36E-03 | 1.25E-01 |
| VWF | 0.423 | Upregulated | 1.96E-03 | 1.46E-01 |
| CMTM5 | 0.423 | Upregulated | 2.80E-04 | 6.47E-02 |
| LAP3 | 0.424 | Upregulated | 1.33E-04 | 4.42E-02 |
| MMRN1 | 0.426 | Upregulated | 1.32E-03 | 1.24E-01 |
| DEFA1 | 0.428 | Upregulated | 3.18E-02 | 3.69E-01 |
| PROS1 | 0.430 | Upregulated | 9.97E-04 | 1.12E-01 |
| SLC6A8 | 0.431 | Upregulated | 1.91E-02 | 3.08E-01 |
| CLEC1B | 0.433 | Upregulated | 6.49E-03 | 2.19E-01 |
| RAB3IL1 | 0.448 | Upregulated | 1.14E-02 | 2.59E-01 |
| CXCL10 | 0.462 | Upregulated | 3.21E-03 | 1.76E-01 |
| GBP4 | 0.463 | Upregulated | 8.89E-05 | 3.95E-02 |
| OSBP2 | 0.463 | Upregulated | 4.20E-02 | 4.01E-01 |
| MYOF | 0.469 | Upregulated | 1.82E-04 | 4.98E-02 |

| | | | | |
|---|---|---|---|---|
| SMARCD3 | 0.470 | Upregulated | 3.46E-05 | 2.46E-02 |
| GBP6 | 0.475 | Upregulated | 4.99E-08 | 5.28E-04 |
| TREML1 | 0.476 | Upregulated | 1.29E-03 | 1.24E-01 |
| MYL9 | 0.480 | Upregulated | 7.23E-04 | 9.72E-02 |
| SOD2 | 0.480 | Upregulated | 3.99E-05 | 2.53E-02 |
| C1QA | 0.485 | Upregulated | 5.88E-07 | 2.04E-03 |
| GP9 | 0.489 | Upregulated | 1.11E-03 | 1.19E-01 |
| FCGR1A | 0.491 | Upregulated | 6.60E-04 | 9.49E-02 |
| CMBL | 0.494 | Upregulated | 1.05E-03 | 1.14E-01 |
| LGALS3BP | 0.495 | Upregulated | 4.68E-04 | 8.06E-02 |
| FCGR1B | 0.495 | Upregulated | 4.01E-04 | 7.63E-02 |
| AQP10 | 0.502 | Upregulated | 3.60E-03 | 1.81E-01 |
| IFIT3 | 0.503 | Upregulated | 1.47E-02 | 2.81E-01 |
| WARS | 0.510 | Upregulated | 3.05E-08 | 5.28E-04 |
| C2 | 0.521 | Upregulated | 1.93E-08 | 5.28E-04 |
| PDCD1LG2 | 0.530 | Upregulated | 5.16E-07 | 1.99E-03 |
| FER1L3 | 0.530 | Upregulated | 8.71E-06 | 1.12E-02 |
| TMEM158 | 0.570 | Upregulated | 6.98E-04 | 9.64E-02 |
| GBP1 | 0.591 | Upregulated | 2.63E-05 | 2.32E-02 |
| CA1 | 0.597 | Upregulated | 3.70E-02 | 3.87E-01 |
| ITGA2B | 0.663 | Upregulated | 1.54E-04 | 4.60E-02 |
| ANKRD22 | 0.670 | Upregulated | 5.82E-06 | 8.18E-03 |
| C1QC | 0.704 | Upregulated | 2.01E-05 | 2.05E-02 |
| GBP5 | 0.769 | Upregulated | 1.71E-07 | 9.90E-04 |
| BATF2 | 0.787 | Upregulated | 3.35E-07 | 1.66E-03 |
| C1QB | 0.951 | Upregulated | 3.84E-06 | 7.01E-03 |

Supplementary Table S3a. Differentially expressed genes_Kenya_Male

| Gene | log FoldChange | Direction of expression | P.Value | adj.P.Val |
|---|---|---|---|---|
| LAG3 | -0.866 | Downregulated | 1.48E-04 | 1.34E-02 |
| ZNF683 | -0.736 | Downregulated | 3.04E-04 | 1.95E-02 |
| CD8A | -0.681 | Downregulated | 7.70E-05 | 9.70E-03 |
| USP18 | -0.646 | Downregulated | 1.15E-02 | 1.24E-01 |
| MCOLN2 | -0.635 | Downregulated | 4.48E-05 | 7.76E-03 |
| GZMK | -0.633 | Downregulated | 4.87E-04 | 2.48E-02 |
| TARP | -0.624 | Downregulated | 3.42E-04 | 2.05E-02 |
| GNLY | -0.603 | Downregulated | 1.35E-02 | 1.34E-01 |
| CD160 | -0.602 | Downregulated | 2.43E-03 | 5.44E-02 |
| PYHIN1 | -0.593 | Downregulated | 1.57E-04 | 1.37E-02 |
| CD2 | -0.592 | Downregulated | 3.37E-06 | 2.60E-03 |
| CD3G | -0.588 | Downregulated | 1.73E-04 | 1.47E-02 |
| CCL2 | -0.588 | Downregulated | 2.53E-02 | 1.87E-01 |
| CLIC3 | -0.586 | Downregulated | 1.12E-03 | 3.75E-02 |
| CTSW | -0.586 | Downregulated | 1.35E-04 | 1.27E-02 |
| GZMH | -0.578 | Downregulated | 4.71E-03 | 7.71E-02 |
| KLRD1 | -0.573 | Downregulated | 2.24E-03 | 5.23E-02 |
| FGFBP2 | -0.572 | Downregulated | 8.77E-03 | 1.07E-01 |
| NMUR1 | -0.568 | Downregulated | 5.81E-04 | 2.75E-02 |
| LRRN3 | -0.567 | Downregulated | 2.16E-02 | 1.72E-01 |
| CCDC102A | -0.563 | Downregulated | 1.48E-05 | 5.31E-03 |
| EVL | -0.559 | Downregulated | 2.88E-05 | 6.74E-03 |
| TOX | -0.555 | Downregulated | 5.40E-05 | 8.39E-03 |
| SAMD3 | -0.551 | Downregulated | 2.14E-05 | 5.83E-03 |
| PVRIG | -0.547 | Downregulated | 1.37E-05 | 5.16E-03 |
| PRKCH | -0.544 | Downregulated | 1.54E-05 | 5.34E-03 |
| PDCD1 | -0.538 | Downregulated | 2.35E-04 | 1.72E-02 |
| EOMES | -0.535 | Downregulated | 1.97E-03 | 4.94E-02 |
| CD247 | -0.533 | Downregulated | 8.40E-05 | 1.02E-02 |
| SKAP1 | -0.533 | Downregulated | 1.45E-05 | 5.29E-03 |
| GZMM | -0.530 | Downregulated | 9.72E-05 | 1.08E-02 |
| IL32 | -0.526 | Downregulated | 3.91E-05 | 7.36E-03 |
| QPRT | -0.526 | Downregulated | 3.13E-05 | 6.98E-03 |
| GPR56 | -0.524 | Downregulated | 1.20E-02 | 1.27E-01 |
| CLIP3 | -0.523 | Downregulated | 7.10E-05 | 9.40E-03 |
| ZNF827 | -0.515 | Downregulated | 1.40E-05 | 5.18E-03 |
| CD27 | -0.515 | Downregulated | 6.94E-04 | 2.97E-02 |
| PTPRCAP | -0.510 | Downregulated | 1.12E-05 | 4.71E-03 |
| CDKN1C | -0.510 | Downregulated | 7.00E-03 | 9.59E-02 |
| MYO18B | -0.503 | Downregulated | 1.22E-02 | 1.29E-01 |
| TGFBR3 | -0.500 | Downregulated | 3.73E-03 | 6.81E-02 |
| CD3E | -0.497 | Downregulated | 6.66E-05 | 9.21E-03 |
| MAP4K1 | -0.496 | Downregulated | 1.14E-05 | 4.71E-03 |
| LIME1 | -0.494 | Downregulated | 1.91E-05 | 5.72E-03 |
| FANCI | -0.493 | Downregulated | 6.10E-04 | 2.79E-02 |
| NCALD | -0.493 | Downregulated | 2.22E-04 | 1.68E-02 |
| PLEKHF1 | -0.492 | Downregulated | 2.04E-03 | 5.03E-02 |

| | | | | |
|---|---|---|---|---|
| PBK | -0.491 | Downregulated | 7.51E-03 | 9.90E-02 |
| CD3D | -0.491 | Downregulated | 1.01E-03 | 3.55E-02 |
| CD6 | -0.486 | Downregulated | 5.51E-04 | 2.64E-02 |
| SIRPG | -0.486 | Downregulated | 2.79E-03 | 5.84E-02 |
| CDT1 | -0.484 | Downregulated | 3.96E-03 | 7.04E-02 |
| ZNF831 | -0.483 | Downregulated | 3.62E-05 | 7.09E-03 |
| PRF1 | -0.475 | Downregulated | 1.16E-02 | 1.25E-01 |
| KLHL3 | -0.475 | Downregulated | 7.48E-04 | 3.09E-02 |
| CHST12 | -0.474 | Downregulated | 2.40E-03 | 5.41E-02 |
| S1PR5 | -0.470 | Downregulated | 2.22E-02 | 1.75E-01 |
| ADA | -0.469 | Downregulated | 9.64E-05 | 1.08E-02 |
| ETS1 | -0.469 | Downregulated | 4.69E-04 | 2.45E-02 |
| THOC4 | -0.467 | Downregulated | 4.83E-06 | 3.35E-03 |
| CCL4L1 | -0.467 | Downregulated | 2.95E-02 | 2.03E-01 |
| SH2D1A | -0.465 | Downregulated | 5.14E-04 | 2.56E-02 |
| BUB1 | -0.464 | Downregulated | 2.10E-02 | 1.70E-01 |
| IFNG | -0.464 | Downregulated | 2.32E-02 | 1.79E-01 |
| HRK | -0.461 | Downregulated | 2.01E-02 | 1.66E-01 |
| CCL5 | -0.459 | Downregulated | 3.14E-03 | 6.23E-02 |
| SLAMF6 | -0.458 | Downregulated | 4.69E-05 | 8.02E-03 |
| CCDC99 | -0.458 | Downregulated | 1.08E-03 | 3.70E-02 |
| RASGRP1 | -0.455 | Downregulated | 7.78E-04 | 3.13E-02 |
| PRKCQ | -0.453 | Downregulated | 5.85E-05 | 8.61E-03 |
| ITM2A | -0.453 | Downregulated | 1.41E-04 | 1.30E-02 |
| LAX1 | -0.450 | Downregulated | 2.21E-04 | 1.68E-02 |
| G3BP1 | -0.450 | Downregulated | 1.30E-04 | 1.26E-02 |
| CD5 | -0.449 | Downregulated | 3.93E-03 | 7.01E-02 |
| ENOSF1 | -0.448 | Downregulated | 1.21E-04 | 1.20E-02 |
| ZNF573 | -0.447 | Downregulated | 1.70E-02 | 1.53E-01 |
| CDC45L | -0.445 | Downregulated | 1.56E-02 | 1.46E-01 |
| IL2RB | -0.444 | Downregulated | 4.19E-03 | 7.26E-02 |
| JAKMIP2 | -0.442 | Downregulated | 7.42E-05 | 9.64E-03 |
| IL28RA | -0.442 | Downregulated | 4.58E-04 | 2.41E-02 |
| CBLB | -0.441 | Downregulated | 1.61E-05 | 5.52E-03 |
| FASLG | -0.440 | Downregulated | 1.30E-02 | 1.32E-01 |
| DTX3 | -0.439 | Downregulated | 6.88E-06 | 3.73E-03 |
| AURKB | -0.439 | Downregulated | 8.80E-03 | 1.08E-01 |
| BYSL | -0.439 | Downregulated | 6.45E-05 | 9.17E-03 |
| ITK | -0.437 | Downregulated | 3.92E-04 | 2.20E-02 |
| TMEM14A | -0.435 | Downregulated | 3.01E-05 | 6.86E-03 |
| TSEN54 | -0.433 | Downregulated | 4.23E-04 | 2.30E-02 |
| PPP3CC | -0.433 | Downregulated | 5.23E-06 | 3.36E-03 |
| LIMA1 | -0.432 | Downregulated | 1.17E-04 | 1.20E-02 |
| CXCR3 | -0.430 | Downregulated | 1.93E-06 | 1.92E-03 |
| CD7 | -0.430 | Downregulated | 6.37E-04 | 2.85E-02 |
| FAM83D | -0.429 | Downregulated | 2.40E-04 | 1.73E-02 |
| CEP78 | -0.429 | Downregulated | 2.11E-03 | 5.10E-02 |
| DENND2D | -0.427 | Downregulated | 3.28E-05 | 7.03E-03 |
| CD96 | -0.427 | Downregulated | 8.81E-04 | 3.33E-02 |
| CCDC34 | -0.426 | Downregulated | 1.94E-03 | 4.91E-02 |
| NOLC1 | -0.425 | Downregulated | 4.60E-04 | 2.42E-02 |

| | | | | |
|---|---|---|---|---|
| KIFC1 | -0.425 | Downregulated | 1.29E-02 | 1.32E-01 |
| CCND2 | -0.424 | Downregulated | 1.77E-05 | 5.62E-03 |
| CXCR7 | -0.422 | Downregulated | 3.82E-04 | 2.16E-02 |
| KIAA1324L | -0.421 | Downregulated | 3.24E-04 | 2.00E-02 |
| SPATA20 | -0.418 | Downregulated | 4.32E-04 | 2.33E-02 |
| HERC6 | -0.417 | Downregulated | 2.21E-02 | 1.74E-01 |
| GPRIN3 | -0.416 | Downregulated | 7.84E-04 | 3.16E-02 |
| ZFP82 | -0.416 | Downregulated | 1.56E-04 | 1.37E-02 |
| FAM159A | -0.415 | Downregulated | 4.93E-04 | 2.49E-02 |
| RRAS2 | -0.414 | Downregulated | 1.26E-04 | 1.23E-02 |
| CLDND2 | -0.412 | Downregulated | 5.12E-03 | 8.09E-02 |
| FAM102A | -0.411 | Downregulated | 5.03E-03 | 7.99E-02 |
| CRIP1 | -0.411 | Downregulated | 1.84E-04 | 1.50E-02 |
| NKG7 | -0.408 | Downregulated | 8.72E-03 | 1.07E-01 |
| ITGB7 | -0.405 | Downregulated | 3.69E-04 | 2.13E-02 |
| TIPIN | -0.404 | Downregulated | 6.11E-04 | 2.79E-02 |
| PACSIN1 | -0.403 | Downregulated | 8.16E-04 | 3.22E-02 |
| STK39 | -0.403 | Downregulated | 7.48E-05 | 9.66E-03 |
| CDCA4 | -0.403 | Downregulated | 4.38E-05 | 7.76E-03 |
| PRSS23 | -0.402 | Downregulated | 1.46E-02 | 1.40E-01 |
| BIN1 | -0.402 | Downregulated | 5.68E-05 | 8.56E-03 |
| TYMS | -0.402 | Downregulated | 9.23E-03 | 1.11E-01 |
| PTGDR | -0.401 | Downregulated | 7.50E-03 | 9.90E-02 |
| CCDC107 | -0.398 | Downregulated | 1.67E-05 | 5.53E-03 |
| TRIB2 | -0.398 | Downregulated | 2.95E-03 | 6.01E-02 |
| ALS2CR4 | -0.398 | Downregulated | 4.75E-04 | 2.46E-02 |
| GFI1 | -0.398 | Downregulated | 2.82E-03 | 5.88E-02 |
| SPOCK2 | -0.396 | Downregulated | 7.82E-03 | 1.01E-01 |
| CLEC2D | -0.395 | Downregulated | 5.30E-05 | 8.37E-03 |
| FAM113B | -0.394 | Downregulated | 1.02E-03 | 3.59E-02 |
| FCRL3 | -0.393 | Downregulated | 1.40E-03 | 4.18E-02 |
| CHI3L2 | -0.393 | Downregulated | 2.51E-02 | 1.87E-01 |
| MCM2 | -0.393 | Downregulated | 6.70E-03 | 9.40E-02 |
| GPR68 | -0.393 | Downregulated | 8.66E-03 | 1.07E-01 |
| MCM10 | -0.392 | Downregulated | 4.30E-03 | 7.35E-02 |
| LY9 | -0.390 | Downregulated | 6.25E-04 | 2.82E-02 |
| RPN2 | -0.390 | Downregulated | 5.17E-03 | 8.13E-02 |
| ARMC5 | -0.390 | Downregulated | 6.22E-06 | 3.49E-03 |
| CTLA4 | -0.390 | Downregulated | 1.12E-03 | 3.75E-02 |
| OCIAD2 | -0.390 | Downregulated | 1.85E-03 | 4.80E-02 |
| CXCR6 | -0.385 | Downregulated | 1.38E-02 | 1.36E-01 |
| GPR114 | -0.382 | Downregulated | 8.78E-03 | 1.07E-01 |
| KIF4A | -0.381 | Downregulated | 1.39E-02 | 1.36E-01 |
| CRYZ | -0.380 | Downregulated | 3.04E-03 | 6.12E-02 |
| MIB2 | -0.379 | Downregulated | 9.58E-05 | 1.08E-02 |
| LDHB | -0.376 | Downregulated | 4.81E-03 | 7.78E-02 |
| FAM84B | -0.376 | Downregulated | 2.95E-03 | 6.01E-02 |
| MCM4 | -0.376 | Downregulated | 7.39E-03 | 9.83E-02 |
| RNASEH2A | -0.375 | Downregulated | 4.99E-04 | 2.52E-02 |
| WDR54 | -0.374 | Downregulated | 2.74E-05 | 6.66E-03 |
| CD8B | -0.374 | Downregulated | 2.38E-06 | 2.23E-03 |

| | | | | |
|---|---|---|---|---|
| PLCG1 | -0.374 | Downregulated | 6.48E-05 | 9.17E-03 |
| STIL | -0.373 | Downregulated | 7.34E-03 | 9.81E-02 |
| CYP2J2 | -0.373 | Downregulated | 9.26E-04 | 3.42E-02 |
| AGMAT | -0.373 | Downregulated | 3.78E-03 | 6.85E-02 |
| TRAM2 | -0.373 | Downregulated | 1.17E-03 | 3.84E-02 |
| TECR | -0.372 | Downregulated | 9.90E-06 | 4.40E-03 |
| TMEM204 | -0.372 | Downregulated | 1.48E-02 | 1.41E-01 |
| KDELC2 | -0.372 | Downregulated | 1.08E-03 | 3.71E-02 |
| KLRA1 | -0.372 | Downregulated | 5.21E-03 | 8.16E-02 |
| FAIM3 | -0.371 | Downregulated | 6.81E-03 | 9.50E-02 |
| TC2N | -0.369 | Downregulated | 1.00E-02 | 1.16E-01 |
| ZBTB46 | -0.368 | Downregulated | 3.74E-03 | 6.82E-02 |
| CCNB2 | -0.368 | Downregulated | 4.73E-02 | 2.60E-01 |
| RFC4 | -0.368 | Downregulated | 1.11E-03 | 3.75E-02 |
| CHAF1B | -0.366 | Downregulated | 3.63E-03 | 6.74E-02 |
| P2RY11 | -0.364 | Downregulated | 1.22E-03 | 3.91E-02 |
| CDCA5 | -0.364 | Downregulated | 3.33E-02 | 2.18E-01 |
| FAM179A | -0.363 | Downregulated | 2.28E-02 | 1.78E-01 |
| SCML4 | -0.363 | Downregulated | 4.95E-04 | 2.50E-02 |
| CX3CR1 | -0.363 | Downregulated | 1.22E-02 | 1.29E-01 |
| LYAR | -0.363 | Downregulated | 2.74E-05 | 6.66E-03 |
| FLJ33590 | -0.362 | Downregulated | 2.59E-03 | 5.60E-02 |
| IKZF3 | -0.361 | Downregulated | 3.83E-02 | 2.34E-01 |
| RNASEH2B | -0.360 | Downregulated | 2.61E-02 | 1.91E-01 |
| GMNN | -0.360 | Downregulated | 3.21E-02 | 2.13E-01 |
| IDH2 | -0.359 | Downregulated | 8.76E-05 | 1.04E-02 |
| ICOS | -0.359 | Downregulated | 2.48E-02 | 1.85E-01 |
| ADCY9 | -0.359 | Downregulated | 2.24E-03 | 5.23E-02 |
| PPAN | -0.358 | Downregulated | 4.69E-04 | 2.45E-02 |
| SIDT1 | -0.358 | Downregulated | 3.67E-04 | 2.13E-02 |
| B3GNT6 | -0.357 | Downregulated | 1.84E-04 | 1.50E-02 |
| CDCA3 | -0.357 | Downregulated | 1.02E-02 | 1.17E-01 |
| KLF12 | -0.357 | Downregulated | 8.66E-04 | 3.31E-02 |
| MEX3C | -0.356 | Downregulated | 5.31E-03 | 8.26E-02 |
| MXD4 | -0.356 | Downregulated | 2.05E-04 | 1.61E-02 |
| MS4A1 | -0.356 | Downregulated | 4.91E-02 | 2.65E-01 |
| SLAMF1 | -0.356 | Downregulated | 1.18E-03 | 3.87E-02 |
| SLC41A1 | -0.356 | Downregulated | 1.75E-04 | 1.47E-02 |
| NMT2 | -0.355 | Downregulated | 1.28E-03 | 3.99E-02 |
| TMEM118 | -0.355 | Downregulated | 1.53E-03 | 4.34E-02 |
| MSC | -0.354 | Downregulated | 9.26E-03 | 1.11E-01 |
| AURKA | -0.354 | Downregulated | 2.03E-02 | 1.67E-01 |
| WDR34 | -0.354 | Downregulated | 4.73E-04 | 2.46E-02 |
| FANCG | -0.353 | Downregulated | 3.21E-04 | 2.00E-02 |
| ESYT1 | -0.353 | Downregulated | 1.53E-03 | 4.34E-02 |
| KIFAP3 | -0.353 | Downregulated | 4.74E-03 | 7.72E-02 |
| TTC38 | -0.353 | Downregulated | 1.49E-02 | 1.42E-01 |
| SIT1 | -0.352 | Downregulated | 2.43E-04 | 1.74E-02 |
| ATXN7L1 | -0.352 | Downregulated | 1.48E-05 | 5.31E-03 |
| S1PR1 | -0.351 | Downregulated | 7.68E-03 | 1.00E-01 |
| GZMA | -0.351 | Downregulated | 4.57E-02 | 2.56E-01 |

| | | | | |
|---|---|---|---|---|
| PFAS | -0.350 | Downregulated | 7.29E-04 | 3.06E-02 |
| PHGDH | -0.350 | Downregulated | 1.21E-02 | 1.28E-01 |
| UAP1 | -0.350 | Downregulated | 2.77E-02 | 1.97E-01 |
| PPP1R16B | -0.350 | Downregulated | 3.57E-04 | 2.11E-02 |
| ITPR3 | -0.350 | Downregulated | 1.92E-04 | 1.53E-02 |
| SLC39A10 | -0.349 | Downregulated | 2.25E-03 | 5.24E-02 |
| ENPP5 | -0.349 | Downregulated | 5.68E-03 | 8.59E-02 |
| SDF2L1 | -0.348 | Downregulated | 6.97E-03 | 9.59E-02 |
| CCNF | -0.348 | Downregulated | 1.70E-03 | 4.58E-02 |
| GPR18 | -0.347 | Downregulated | 4.46E-03 | 7.49E-02 |
| CENPA | -0.347 | Downregulated | 1.64E-02 | 1.50E-01 |
| LMNB2 | -0.346 | Downregulated | 3.03E-04 | 1.95E-02 |
| NOC3L | -0.346 | Downregulated | 1.94E-02 | 1.64E-01 |
| PMS1 | -0.346 | Downregulated | 2.20E-02 | 1.74E-01 |
| EBI2 | -0.345 | Downregulated | 1.88E-02 | 1.61E-01 |
| SLC27A2 | -0.344 | Downregulated | 7.53E-03 | 9.91E-02 |
| MLLT11 | -0.343 | Downregulated | 6.67E-04 | 2.92E-02 |
| PARP1 | -0.343 | Downregulated | 4.85E-04 | 2.48E-02 |
| PARP15 | -0.343 | Downregulated | 1.81E-03 | 4.74E-02 |
| ATP6V0E2 | -0.342 | Downregulated | 3.93E-03 | 7.01E-02 |
| TDP1 | -0.341 | Downregulated | 3.19E-04 | 1.99E-02 |
| EPHA4 | -0.341 | Downregulated | 3.39E-03 | 6.50E-02 |
| RPS28 | -0.341 | Downregulated | 1.75E-02 | 1.55E-01 |
| GLOD4 | -0.341 | Downregulated | 6.23E-06 | 3.49E-03 |
| SLC25A23 | -0.340 | Downregulated | 2.83E-03 | 5.90E-02 |
| CUTA | -0.339 | Downregulated | 2.89E-05 | 6.74E-03 |
| RFTN1 | -0.339 | Downregulated | 1.30E-04 | 1.26E-02 |
| TUBB | -0.338 | Downregulated | 8.84E-05 | 1.04E-02 |
| DNMT1 | -0.338 | Downregulated | 6.74E-04 | 2.93E-02 |
| TBRG4 | -0.338 | Downregulated | 5.30E-05 | 8.37E-03 |
| CBX5 | -0.337 | Downregulated | 2.01E-03 | 5.02E-02 |
| TNFRSF25 | -0.336 | Downregulated | 5.92E-03 | 8.77E-02 |
| MRPL38 | -0.336 | Downregulated | 2.51E-05 | 6.49E-03 |
| FYN | -0.336 | Downregulated | 1.67E-04 | 1.44E-02 |
| WBSCR22 | -0.336 | Downregulated | 1.25E-05 | 4.83E-03 |
| ZFP42 | -0.336 | Downregulated | 7.68E-04 | 3.12E-02 |
| ARPC5L | -0.335 | Downregulated | 4.58E-06 | 3.29E-03 |
| ITM2C | -0.335 | Downregulated | 4.52E-02 | 2.54E-01 |
| FBXO31 | -0.335 | Downregulated | 3.36E-05 | 7.03E-03 |
| KLRC1 | -0.333 | Downregulated | 1.67E-02 | 1.52E-01 |
| IFFO2 | -0.332 | Downregulated | 2.45E-05 | 6.43E-03 |
| GPATCH4 | -0.332 | Downregulated | 7.87E-03 | 1.01E-01 |
| URG4 | -0.332 | Downregulated | 3.37E-06 | 2.60E-03 |
| MIF | -0.331 | Downregulated | 1.37E-04 | 1.28E-02 |
| NTHL1 | -0.331 | Downregulated | 6.14E-04 | 2.80E-02 |
| NEK1 | -0.331 | Downregulated | 3.71E-03 | 6.81E-02 |
| PDXP | -0.330 | Downregulated | 3.77E-04 | 2.15E-02 |
| TAF1B | -0.330 | Downregulated | 1.45E-04 | 1.33E-02 |
| MTP18 | -0.330 | Downregulated | 2.79E-04 | 1.87E-02 |
| DCLRE1A | -0.330 | Downregulated | 4.71E-03 | 7.71E-02 |
| RANBP1 | -0.329 | Downregulated | 1.20E-04 | 1.20E-02 |

| | | | | |
|---|---|---|---|---|
| CKAP2L | -0.329 | Downregulated | 4.80E-02 | 2.62E-01 |
| MEI1 | -0.329 | Downregulated | 6.81E-04 | 2.95E-02 |
| CCNA2 | -0.328 | Downregulated | 3.81E-02 | 2.33E-01 |
| MMACHC | -0.327 | Downregulated | 1.11E-03 | 3.75E-02 |
| CROT | -0.327 | Downregulated | 1.61E-03 | 4.43E-02 |
| SNRPF | -0.327 | Downregulated | 2.28E-04 | 1.70E-02 |
| GINS4 | -0.327 | Downregulated | 6.73E-05 | 9.23E-03 |
| MCM6 | -0.327 | Downregulated | 1.28E-03 | 3.99E-02 |
| TMEM156 | -0.327 | Downregulated | 3.30E-03 | 6.42E-02 |
| GINS3 | -0.327 | Downregulated | 6.08E-03 | 8.86E-02 |
| NCAPD2 | -0.327 | Downregulated | 3.83E-03 | 6.89E-02 |
| NDUFB9 | -0.326 | Downregulated | 9.38E-04 | 3.44E-02 |
| LPXN | -0.325 | Downregulated | 2.80E-06 | 2.33E-03 |
| ABHD15 | -0.325 | Downregulated | 8.82E-04 | 3.33E-02 |
| CD320 | -0.325 | Downregulated | 1.03E-02 | 1.18E-01 |
| MAK16 | -0.324 | Downregulated | 2.27E-03 | 5.26E-02 |
| YARS | -0.324 | Downregulated | 9.72E-06 | 4.38E-03 |
| SLAIN1 | -0.324 | Downregulated | 7.70E-05 | 9.70E-03 |
| ABLIM1 | -0.324 | Downregulated | 1.75E-02 | 1.55E-01 |
| NOL9 | -0.324 | Downregulated | 7.37E-04 | 3.07E-02 |
| RPUSD2 | -0.323 | Downregulated | 1.75E-04 | 1.47E-02 |
| CENPP | -0.323 | Downregulated | 1.59E-03 | 4.40E-02 |
| GOLGA8A | -0.323 | Downregulated | 4.19E-03 | 7.26E-02 |
| BIVM | -0.323 | Downregulated | 2.58E-03 | 5.60E-02 |
| SFMBT2 | -0.323 | Downregulated | 2.40E-04 | 1.73E-02 |
| CEP70 | -0.322 | Downregulated | 6.11E-03 | 8.90E-02 |
| MGC3020 | -0.322 | Downregulated | 3.38E-03 | 6.50E-02 |
| MNAT1 | -0.322 | Downregulated | 4.00E-03 | 7.08E-02 |
| ATIC | -0.322 | Downregulated | 2.20E-04 | 1.67E-02 |
| PLEKHA1 | -0.321 | Downregulated | 1.97E-03 | 4.94E-02 |
| FAM162A | -0.321 | Downregulated | 9.57E-03 | 1.12E-01 |
| SERPINA10 | -0.321 | Downregulated | 1.58E-03 | 4.40E-02 |
| STAT4 | -0.320 | Downregulated | 3.19E-03 | 6.29E-02 |
| GLB1L2 | -0.320 | Downregulated | 4.42E-04 | 2.35E-02 |
| SLC38A1 | -0.320 | Downregulated | 1.53E-05 | 5.34E-03 |
| GINS2 | -0.320 | Downregulated | 4.16E-02 | 2.45E-01 |
| ALDH18A1 | -0.319 | Downregulated | 6.69E-03 | 9.40E-02 |
| GEMIN4 | -0.319 | Downregulated | 9.52E-04 | 3.46E-02 |
| SUV39H1 | -0.319 | Downregulated | 9.41E-04 | 3.44E-02 |
| CLYBL | -0.319 | Downregulated | 5.13E-03 | 8.10E-02 |
| RRP7A | -0.319 | Downregulated | 1.58E-03 | 4.40E-02 |
| HRASLS2 | -0.318 | Downregulated | 4.73E-02 | 2.60E-01 |
| SLFN13 | -0.318 | Downregulated | 3.96E-03 | 7.04E-02 |
| BCAR3 | -0.318 | Downregulated | 4.19E-02 | 2.45E-01 |
| FABP5 | -0.317 | Downregulated | 2.51E-02 | 1.87E-01 |
| NOP56 | -0.317 | Downregulated | 1.32E-04 | 1.26E-02 |
| CD70 | -0.317 | Downregulated | 9.64E-03 | 1.13E-01 |
| AARS | -0.317 | Downregulated | 1.07E-03 | 3.68E-02 |
| LEO1 | -0.316 | Downregulated | 3.14E-05 | 6.98E-03 |
| CKAP5 | -0.316 | Downregulated | 1.86E-04 | 1.50E-02 |
| ZNF256 | -0.315 | Downregulated | 7.92E-03 | 1.02E-01 |

| | | | | |
|---|---|---|---|---|
| BRIX1 | -0.315 | Downregulated | 2.16E-03 | 5.15E-02 |
| RRM1 | -0.315 | Downregulated | 9.38E-04 | 3.44E-02 |
| NOP58 | -0.315 | Downregulated | 2.85E-03 | 5.93E-02 |
| AVEN | -0.315 | Downregulated | 4.71E-03 | 7.71E-02 |
| TNIP3 | -0.315 | Downregulated | 2.46E-03 | 5.47E-02 |
| GPR174 | -0.314 | Downregulated | 5.10E-05 | 8.35E-03 |
| TIMM8A | -0.314 | Downregulated | 9.95E-04 | 3.52E-02 |
| STMN3 | -0.314 | Downregulated | 2.58E-02 | 1.90E-01 |
| WDR67 | -0.314 | Downregulated | 8.16E-04 | 3.22E-02 |
| PRMT7 | -0.314 | Downregulated | 3.30E-04 | 2.01E-02 |
| RHBDD3 | -0.314 | Downregulated | 1.11E-03 | 3.75E-02 |
| FLJ20699 | -0.314 | Downregulated | 3.46E-02 | 2.22E-01 |
| NOP14 | -0.313 | Downregulated | 1.04E-07 | 2.77E-04 |
| SP4 | -0.313 | Downregulated | 2.03E-03 | 5.02E-02 |
| PPRC1 | -0.313 | Downregulated | 7.31E-05 | 9.57E-03 |
| GADD45GIP1 | -0.313 | Downregulated | 1.50E-03 | 4.31E-02 |
| CDCA2 | -0.312 | Downregulated | 2.76E-02 | 1.97E-01 |
| NCAPG2 | -0.312 | Downregulated | 2.66E-02 | 1.93E-01 |
| MRPL48 | -0.312 | Downregulated | 1.10E-02 | 1.22E-01 |
| CD81 | -0.312 | Downregulated | 6.42E-04 | 2.85E-02 |
| SLC25A4 | -0.312 | Downregulated | 1.61E-02 | 1.49E-01 |
| RAD51C | -0.312 | Downregulated | 1.89E-03 | 4.86E-02 |
| ALKBH8 | -0.312 | Downregulated | 4.36E-04 | 2.34E-02 |
| MRPL9 | -0.311 | Downregulated | 1.19E-04 | 1.20E-02 |
| NLRC3 | -0.311 | Downregulated | 5.19E-04 | 2.56E-02 |
| CARD11 | -0.311 | Downregulated | 5.05E-04 | 2.53E-02 |
| LCE3B | -0.310 | Downregulated | 1.89E-03 | 4.86E-02 |
| MPI | -0.310 | Downregulated | 2.07E-04 | 1.62E-02 |
| RBBP7 | -0.310 | Downregulated | 2.16E-04 | 1.65E-02 |
| BTG3 | -0.310 | Downregulated | 3.40E-03 | 6.50E-02 |
| FAM98A | -0.310 | Downregulated | 9.95E-03 | 1.15E-01 |
| GRWD1 | -0.310 | Downregulated | 8.76E-04 | 3.33E-02 |
| SPRY2 | -0.310 | Downregulated | 3.77E-03 | 6.84E-02 |
| VIL2 | -0.309 | Downregulated | 8.16E-04 | 3.22E-02 |
| FLJ46020 | -0.309 | Downregulated | 6.94E-05 | 9.30E-03 |
| TIMM44 | -0.309 | Downregulated | 7.66E-04 | 3.12E-02 |
| PDCD2L | -0.309 | Downregulated | 3.97E-03 | 7.04E-02 |
| KIF11 | -0.308 | Downregulated | 2.92E-02 | 2.02E-01 |
| SAE1 | -0.308 | Downregulated | 4.00E-05 | 7.43E-03 |
| PIK3IP1 | -0.307 | Downregulated | 3.53E-02 | 2.25E-01 |
| PTPN4 | -0.307 | Downregulated | 9.63E-03 | 1.13E-01 |
| A2LD1 | -0.307 | Downregulated | 3.52E-03 | 6.61E-02 |
| TRIM32 | -0.307 | Downregulated | 2.25E-04 | 1.69E-02 |
| AES | -0.307 | Downregulated | 5.86E-04 | 2.75E-02 |
| TRAF2 | -0.306 | Downregulated | 3.92E-04 | 2.20E-02 |
| LCK | -0.306 | Downregulated | 5.70E-05 | 8.56E-03 |
| TXK | -0.306 | Downregulated | 2.66E-02 | 1.93E-01 |
| YES1 | -0.306 | Downregulated | 1.37E-03 | 4.13E-02 |
| CTPS | -0.305 | Downregulated | 2.81E-03 | 5.87E-02 |
| HERC2 | -0.305 | Downregulated | 7.63E-03 | 9.98E-02 |
| HSPA8 | -0.305 | Downregulated | 1.66E-03 | 4.52E-02 |

| | | | | |
|---|---|---|---|---|
| SLC2A4RG | -0.305 | Downregulated | 2.13E-05 | 5.83E-03 |
| SMYD3 | -0.305 | Downregulated | 3.64E-05 | 7.09E-03 |
| CHAC2 | -0.304 | Downregulated | 2.74E-02 | 1.96E-01 |
| LPIN1 | -0.304 | Downregulated | 9.25E-04 | 3.42E-02 |
| SUMF2 | -0.304 | Downregulated | 3.32E-05 | 7.03E-03 |
| Septin 9 | -0.304 | Downregulated | 7.06E-05 | 9.38E-03 |
| GIMAP1 | -0.303 | Downregulated | 1.04E-02 | 1.18E-01 |
| ABHD14A | -0.303 | Downregulated | 8.67E-04 | 3.31E-02 |
| ZNHIT2 | -0.303 | Downregulated | 5.88E-05 | 8.61E-03 |
| POLD1 | -0.303 | Downregulated | 1.41E-03 | 4.18E-02 |
| FLJ20628 | -0.303 | Downregulated | 8.87E-04 | 3.34E-02 |
| SERPINF1 | -0.302 | Downregulated | 1.19E-03 | 3.88E-02 |
| FH | -0.302 | Downregulated | 1.41E-03 | 4.18E-02 |
| ECHDC2 | -0.302 | Downregulated | 6.52E-04 | 2.88E-02 |
| KIF22 | -0.302 | Downregulated | 8.48E-06 | 4.05E-03 |
| DNAJC9 | -0.302 | Downregulated | 9.47E-04 | 3.44E-02 |
| NSUN6 | -0.302 | Downregulated | 1.39E-04 | 1.29E-02 |
| LRFN3 | -0.301 | Downregulated | 2.51E-03 | 5.54E-02 |
| ALG8 | -0.301 | Downregulated | 5.30E-05 | 8.37E-03 |
| RPL10A | -0.301 | Downregulated | 5.36E-03 | 8.31E-02 |
| DBP | -0.300 | Downregulated | 4.15E-04 | 2.27E-02 |
| HNRNPH1 | -0.300 | Downregulated | 6.84E-04 | 2.96E-02 |
| LRDD | -0.300 | Downregulated | 2.98E-04 | 1.92E-02 |
| IGFBP4 | -0.299 | Downregulated | 1.46E-02 | 1.40E-01 |
| ELOVL6 | -0.299 | Downregulated | 5.24E-03 | 8.18E-02 |
| XBP1 | -0.299 | Downregulated | 1.40E-02 | 1.37E-01 |
| METTL6 | -0.298 | Downregulated | 3.70E-03 | 6.80E-02 |
| POLRMT | -0.298 | Downregulated | 9.66E-04 | 3.48E-02 |
| HSPH1 | -0.298 | Downregulated | 1.11E-02 | 1.22E-01 |
| SLC25A42 | -0.298 | Downregulated | 2.78E-05 | 6.66E-03 |
| UPF3A | -0.298 | Downregulated | 2.31E-04 | 1.71E-02 |
| FKBP11 | -0.298 | Downregulated | 2.71E-02 | 1.95E-01 |
| GJA9 | -0.298 | Downregulated | 1.97E-02 | 1.65E-01 |
| TARBP1 | -0.298 | Downregulated | 9.40E-03 | 1.11E-01 |
| TBC1D19 | -0.297 | Downregulated | 3.50E-04 | 2.08E-02 |
| DDX54 | -0.297 | Downregulated | 1.41E-03 | 4.18E-02 |
| ZNHIT6 | -0.297 | Downregulated | 1.37E-04 | 1.28E-02 |
| GPX7 | -0.296 | Downregulated | 5.02E-03 | 7.98E-02 |
| IBTK | -0.296 | Downregulated | 1.80E-02 | 1.58E-01 |
| ZMYND19 | -0.296 | Downregulated | 1.84E-03 | 4.79E-02 |
| MELK | -0.296 | Downregulated | 3.46E-02 | 2.22E-01 |
| XPO4 | -0.295 | Downregulated | 3.39E-04 | 2.04E-02 |
| NUSAP1 | -0.295 | Downregulated | 1.99E-02 | 1.66E-01 |
| AKR1B1 | -0.295 | Downregulated | 4.53E-04 | 2.39E-02 |
| EXOSC10 | -0.295 | Downregulated | 1.09E-03 | 3.72E-02 |
| GRAMD3 | -0.295 | Downregulated | 4.79E-04 | 2.47E-02 |
| NAT10 | -0.295 | Downregulated | 2.71E-03 | 5.74E-02 |
| RFC3 | -0.295 | Downregulated | 6.53E-03 | 9.29E-02 |
| MTHFD1L | -0.295 | Downregulated | 2.38E-03 | 5.39E-02 |
| MPP6 | -0.295 | Downregulated | 8.20E-03 | 1.04E-01 |
| APBA2 | -0.295 | Downregulated | 1.25E-02 | 1.30E-01 |

| | | | | |
|---|---|---|---|---|
| SF3A3 | -0.294 | Downregulated | 1.82E-04 | 1.50E-02 |
| PRC1 | -0.294 | Downregulated | 2.34E-02 | 1.80E-01 |
| ARL1 | -0.294 | Downregulated | 8.42E-03 | 1.05E-01 |
| IMP3 | -0.294 | Downregulated | 4.85E-05 | 8.14E-03 |
| MDC1 | -0.294 | Downregulated | 3.94E-04 | 2.20E-02 |
| NDC80 | -0.294 | Downregulated | 3.09E-02 | 2.09E-01 |
| UFSP2 | -0.294 | Downregulated | 8.93E-03 | 1.09E-01 |
| PAK1IP1 | -0.293 | Downregulated | 3.28E-04 | 2.01E-02 |
| POLE2 | -0.293 | Downregulated | 1.70E-02 | 1.53E-01 |
| RRP1B | -0.293 | Downregulated | 6.92E-04 | 2.97E-02 |
| CCDC28B | -0.293 | Downregulated | 4.84E-04 | 2.48E-02 |
| RNMTL1 | -0.293 | Downregulated | 4.89E-05 | 8.14E-03 |
| LSM2 | -0.292 | Downregulated | 5.24E-05 | 8.37E-03 |
| PTPRM | -0.292 | Downregulated | 2.81E-02 | 1.98E-01 |
| GIMAP5 | -0.292 | Downregulated | 1.96E-02 | 1.64E-01 |
| PEBP1 | -0.292 | Downregulated | 5.26E-04 | 2.57E-02 |
| NDUFV1 | -0.292 | Downregulated | 3.29E-05 | 7.03E-03 |
| PAICS | -0.291 | Downregulated | 4.47E-03 | 7.50E-02 |
| PTPLAD1 | -0.291 | Downregulated | 1.12E-04 | 1.17E-02 |
| FAM108A3 | -0.291 | Downregulated | 7.04E-04 | 3.00E-02 |
| AGK | -0.291 | Downregulated | 9.18E-04 | 3.40E-02 |
| TOMM40 | -0.290 | Downregulated | 2.96E-05 | 6.80E-03 |
| BOP1 | -0.289 | Downregulated | 2.73E-04 | 1.85E-02 |
| INPP5E | -0.289 | Downregulated | 1.04E-03 | 3.62E-02 |
| PCNT | -0.289 | Downregulated | 2.12E-03 | 5.12E-02 |
| INTS2 | -0.289 | Downregulated | 5.08E-03 | 8.04E-02 |
| MRPS9 | -0.289 | Downregulated | 5.16E-05 | 8.37E-03 |
| BZRAP1 | -0.289 | Downregulated | 7.41E-03 | 9.84E-02 |
| POMZP3 | -0.288 | Downregulated | 1.69E-02 | 1.52E-01 |
| SYTL2 | -0.288 | Downregulated | 5.92E-03 | 8.77E-02 |
| DONSON | -0.287 | Downregulated | 2.76E-03 | 5.82E-02 |
| MANEA | -0.287 | Downregulated | 6.81E-03 | 9.50E-02 |
| ACTR5 | -0.287 | Downregulated | 5.62E-06 | 3.36E-03 |
| APOA1BP | -0.287 | Downregulated | 9.18E-05 | 1.05E-02 |
| CENPL | -0.287 | Downregulated | 3.71E-04 | 2.13E-02 |
| NEK2 | -0.286 | Downregulated | 1.35E-02 | 1.34E-01 |
| RAB30 | -0.286 | Downregulated | 3.23E-02 | 2.14E-01 |
| NELF | -0.286 | Downregulated | 2.26E-03 | 5.25E-02 |
| PCID2 | -0.285 | Downregulated | 2.63E-04 | 1.82E-02 |
| FASN | -0.285 | Downregulated | 2.04E-02 | 1.67E-01 |
| GAR1 | -0.285 | Downregulated | 4.18E-03 | 7.25E-02 |
| WDR62 | -0.285 | Downregulated | 6.42E-03 | 9.20E-02 |
| HSPE1 | -0.284 | Downregulated | 4.38E-02 | 2.50E-01 |
| STT3A | -0.284 | Downregulated | 5.73E-03 | 8.62E-02 |
| RRS1 | -0.284 | Downregulated | 1.29E-03 | 3.99E-02 |
| DNAJA3 | -0.284 | Downregulated | 2.66E-05 | 6.66E-03 |
| PDIA4 | -0.284 | Downregulated | 2.41E-02 | 1.83E-01 |
| EIF2AK3 | -0.284 | Downregulated | 9.97E-03 | 1.15E-01 |
| WDR92 | -0.283 | Downregulated | 6.89E-04 | 2.97E-02 |
| NAT6 | -0.283 | Downregulated | 3.40E-03 | 6.50E-02 |
| NFATC3 | -0.283 | Downregulated | 2.08E-05 | 5.83E-03 |

| | | | | |
|---|---|---|---|---|
| CCL17 | -0.283 | Downregulated | 4.85E-02 | 2.63E-01 |
| SPTAN1 | -0.282 | Downregulated | 2.69E-03 | 5.73E-02 |
| TTC27 | -0.282 | Downregulated | 9.89E-04 | 3.52E-02 |
| KLHDC4 | -0.282 | Downregulated | 1.14E-03 | 3.79E-02 |
| SLC9A3R1 | -0.282 | Downregulated | 7.19E-04 | 3.03E-02 |
| ALS2CR8 | -0.281 | Downregulated | 1.87E-03 | 4.83E-02 |
| EZR | -0.281 | Downregulated | 1.53E-04 | 1.36E-02 |
| CHEK1 | -0.281 | Downregulated | 1.98E-02 | 1.65E-01 |
| PIGU | -0.281 | Downregulated | 3.55E-05 | 7.03E-03 |
| SLC35F2 | -0.281 | Downregulated | 4.77E-03 | 7.75E-02 |
| KIF20A | -0.281 | Downregulated | 3.33E-02 | 2.18E-01 |
| ELP4 | -0.281 | Downregulated | 1.72E-03 | 4.60E-02 |
| MCM3 | -0.281 | Downregulated | 4.73E-04 | 2.46E-02 |
| NAE1 | -0.281 | Downregulated | 2.93E-02 | 2.03E-01 |
| MKI67 | -0.281 | Downregulated | 9.80E-04 | 3.50E-02 |
| SFRS13A | -0.280 | Downregulated | 2.68E-03 | 5.71E-02 |
| SYT11 | -0.280 | Downregulated | 2.56E-03 | 5.58E-02 |
| MT1F | -0.280 | Downregulated | 2.65E-03 | 5.66E-02 |
| QDPR | -0.280 | Downregulated | 8.72E-05 | 1.04E-02 |
| RPL23A | -0.279 | Downregulated | 2.90E-03 | 5.98E-02 |
| NUDCD2 | -0.279 | Downregulated | 1.38E-02 | 1.36E-01 |
| GPR89C | -0.279 | Downregulated | 3.37E-04 | 2.04E-02 |
| FAM164A | -0.279 | Downregulated | 7.56E-03 | 9.92E-02 |
| HIRIP3 | -0.279 | Downregulated | 3.61E-04 | 2.12E-02 |
| UNC84B | -0.279 | Downregulated | 2.86E-04 | 1.89E-02 |
| SUPT3H | -0.278 | Downregulated | 2.51E-03 | 5.54E-02 |
| METTL2A | -0.278 | Downregulated | 3.57E-05 | 7.03E-03 |
| HARS | -0.278 | Downregulated | 1.98E-03 | 4.95E-02 |
| ARMET | -0.278 | Downregulated | 3.43E-02 | 2.21E-01 |
| GIMAP7 | -0.278 | Downregulated | 9.31E-03 | 1.11E-01 |
| MRPS6 | -0.278 | Downregulated | 4.43E-04 | 2.35E-02 |
| GK5 | -0.278 | Downregulated | 7.22E-03 | 9.75E-02 |
| TRMT112 | -0.277 | Downregulated | 7.60E-05 | 9.70E-03 |
| THOC1 | -0.277 | Downregulated | 5.03E-05 | 8.27E-03 |
| Septin 1 | -0.277 | Downregulated | 7.78E-04 | 3.13E-02 |
| EBP | -0.277 | Downregulated | 2.04E-03 | 5.03E-02 |
| THOC6 | -0.276 | Downregulated | 2.77E-03 | 5.82E-02 |
| FAM64A | -0.276 | Downregulated | 1.68E-03 | 4.55E-02 |
| ZCCHC3 | -0.276 | Downregulated | 6.42E-04 | 2.85E-02 |
| UHRF1 | -0.276 | Downregulated | 2.24E-03 | 5.24E-02 |
| ICA1 | -0.276 | Downregulated | 9.75E-03 | 1.14E-01 |
| LARS2 | -0.276 | Downregulated | 2.55E-03 | 5.58E-02 |
| SARM1 | -0.275 | Downregulated | 1.60E-03 | 4.42E-02 |
| GTPBP4 | -0.275 | Downregulated | 3.05E-04 | 1.95E-02 |
| TASP1 | -0.275 | Downregulated | 2.28E-03 | 5.28E-02 |
| THOC3 | -0.275 | Downregulated | 1.42E-02 | 1.38E-01 |
| PRKAR1B | -0.275 | Downregulated | 1.41E-03 | 4.18E-02 |
| HAUS5 | -0.274 | Downregulated | 6.40E-04 | 2.85E-02 |
| RAB40B | -0.274 | Downregulated | 8.95E-04 | 3.35E-02 |
| ILF3 | -0.274 | Downregulated | 4.84E-04 | 2.48E-02 |
| NPM3 | -0.274 | Downregulated | 6.87E-03 | 9.52E-02 |

| | | | | |
|---|---|---|---|---|
| CDRT4 | -0.274 | Downregulated | 6.22E-03 | 9.00E-02 |
| CELSR3 | -0.274 | Downregulated | 2.11E-02 | 1.70E-01 |
| TH1L | -0.274 | Downregulated | 8.57E-06 | 4.05E-03 |
| KCTD7 | -0.274 | Downregulated | 1.77E-02 | 1.56E-01 |
| ZXDB | -0.274 | Downregulated | 3.94E-04 | 2.20E-02 |
| GLCCI1 | -0.274 | Downregulated | 9.76E-03 | 1.14E-01 |
| LMF1 | -0.274 | Downregulated | 1.68E-03 | 4.55E-02 |
| FTO | -0.274 | Downregulated | 2.56E-03 | 5.59E-02 |
| ZBTB9 | -0.273 | Downregulated | 2.78E-04 | 1.87E-02 |
| FAM167A | -0.273 | Downregulated | 3.67E-02 | 2.30E-01 |
| PRMT1 | -0.273 | Downregulated | 2.73E-04 | 1.85E-02 |
| TSEN15 | -0.273 | Downregulated | 7.13E-03 | 9.68E-02 |
| DYRK2 | -0.273 | Downregulated | 2.91E-03 | 5.99E-02 |
| ILF2 | -0.273 | Downregulated | 1.09E-04 | 1.16E-02 |
| INPP4B | -0.273 | Downregulated | 5.41E-03 | 8.34E-02 |
| SMARCAD1 | -0.273 | Downregulated | 5.98E-03 | 8.81E-02 |
| PASK | -0.272 | Downregulated | 4.64E-02 | 2.58E-01 |
| GSDMB | -0.272 | Downregulated | 4.41E-04 | 2.35E-02 |
| GPAM | -0.272 | Downregulated | 3.06E-03 | 6.13E-02 |
| OIP5 | -0.272 | Downregulated | 2.68E-02 | 1.93E-01 |
| CDC7 | -0.271 | Downregulated | 1.23E-02 | 1.29E-01 |
| SPIN4 | -0.271 | Downregulated | 3.68E-02 | 2.30E-01 |
| MRPS30 | -0.271 | Downregulated | 2.01E-05 | 5.83E-03 |
| FAF1 | -0.271 | Downregulated | 3.45E-05 | 7.03E-03 |
| PLSCR3 | -0.271 | Downregulated | 1.20E-03 | 3.89E-02 |
| LONP1 | -0.271 | Downregulated | 2.00E-03 | 5.00E-02 |
| KIAA1430 | -0.270 | Downregulated | 4.43E-03 | 7.47E-02 |
| BOLA3 | -0.270 | Downregulated | 3.03E-03 | 6.10E-02 |
| AOF2 | -0.270 | Downregulated | 4.31E-05 | 7.66E-03 |
| MORC2 | -0.270 | Downregulated | 3.68E-03 | 6.79E-02 |
| PWP1 | -0.270 | Downregulated | 3.35E-04 | 2.04E-02 |
| PHB | -0.269 | Downregulated | 5.99E-04 | 2.77E-02 |
| EIF3C | -0.269 | Downregulated | 9.09E-05 | 1.05E-02 |
| FLJ35801 | -0.269 | Downregulated | 2.63E-03 | 5.65E-02 |
| DOLPP1 | -0.269 | Downregulated | 3.70E-04 | 2.13E-02 |
| TCTN3 | -0.269 | Downregulated | 3.76E-03 | 6.83E-02 |
| N6AMT2 | -0.269 | Downregulated | 1.32E-03 | 4.03E-02 |
| ANXA6 | -0.269 | Downregulated | 7.10E-04 | 3.02E-02 |
| IL27RA | -0.269 | Downregulated | 3.35E-04 | 2.04E-02 |
| GOT1 | -0.269 | Downregulated | 1.30E-03 | 4.00E-02 |
| ASF1B | -0.269 | Downregulated | 6.02E-03 | 8.84E-02 |
| HSP90B1 | -0.268 | Downregulated | 4.65E-02 | 2.58E-01 |
| SLC7A6 | -0.268 | Downregulated | 4.37E-03 | 7.41E-02 |
| TRNP1 | -0.268 | Downregulated | 2.48E-02 | 1.85E-01 |
| RLTPR | -0.268 | Downregulated | 1.24E-02 | 1.30E-01 |
| HNRPR | -0.268 | Downregulated | 3.41E-04 | 2.05E-02 |
| IARS | -0.268 | Downregulated | 8.14E-06 | 4.03E-03 |
| CYFIP2 | -0.268 | Downregulated | 5.94E-04 | 2.76E-02 |
| CPSF6 | -0.268 | Downregulated | 1.44E-03 | 4.23E-02 |
| TNIK | -0.267 | Downregulated | 3.41E-03 | 6.51E-02 |
| TMEM194A | -0.267 | Downregulated | 1.92E-03 | 4.89E-02 |

| | | | | |
|---|---|---|---|---|
| MGC26718 | -0.267 | Downregulated | 8.48E-03 | 1.06E-01 |
| CDC25A | -0.267 | Downregulated | 1.50E-02 | 1.42E-01 |
| MCM8 | -0.267 | Downregulated | 1.33E-03 | 4.04E-02 |
| CCNB1 | -0.267 | Downregulated | 2.24E-02 | 1.76E-01 |
| SHMT2 | -0.267 | Downregulated | 1.23E-03 | 3.92E-02 |
| TMEM194 | -0.267 | Downregulated | 3.43E-03 | 6.53E-02 |
| CHCHD6 | -0.267 | Downregulated | 5.17E-03 | 8.14E-02 |
| LCMT2 | -0.266 | Downregulated | 2.81E-04 | 1.87E-02 |
| E2F8 | -0.266 | Downregulated | 2.78E-03 | 5.84E-02 |
| SBF1 | -0.266 | Downregulated | 4.06E-05 | 7.49E-03 |
| CD1C | -0.266 | Downregulated | 1.33E-02 | 1.34E-01 |
| CENPJ | -0.266 | Downregulated | 1.19E-02 | 1.27E-01 |
| MYO1G | -0.266 | Downregulated | 5.87E-04 | 2.75E-02 |
| MYC | -0.265 | Downregulated | 4.04E-02 | 2.41E-01 |
| TTC16 | -0.265 | Downregulated | 2.78E-03 | 5.84E-02 |
| SLC15A4 | 0.265 | Upregulated | 7.94E-03 | 1.02E-01 |
| RILPL2 | 0.265 | Upregulated | 5.06E-03 | 8.02E-02 |
| CD300LD | 0.266 | Upregulated | 1.69E-03 | 4.57E-02 |
| NACC2 | 0.266 | Upregulated | 1.12E-02 | 1.23E-01 |
| SLC6A4 | 0.266 | Upregulated | 1.87E-02 | 1.60E-01 |
| NT5C3 | 0.266 | Upregulated | 7.23E-03 | 9.75E-02 |
| CDC42EP2 | 0.267 | Upregulated | 3.33E-02 | 2.18E-01 |
| SDCBP | 0.267 | Upregulated | 3.10E-02 | 2.09E-01 |
| NAIP | 0.267 | Upregulated | 2.64E-02 | 1.92E-01 |
| TCBA1 | 0.267 | Upregulated | 3.48E-04 | 2.07E-02 |
| ATP1B2 | 0.267 | Upregulated | 9.67E-03 | 1.13E-01 |
| TALDO1 | 0.267 | Upregulated | 1.48E-03 | 4.28E-02 |
| F11R | 0.267 | Upregulated | 3.43E-03 | 6.53E-02 |
| S100A8 | 0.268 | Upregulated | 4.59E-02 | 2.56E-01 |
| ARAP3 | 0.268 | Upregulated | 4.67E-02 | 2.58E-01 |
| NUAK2 | 0.268 | Upregulated | 1.24E-03 | 3.93E-02 |
| HRASLS | 0.269 | Upregulated | 6.16E-03 | 8.94E-02 |
| Septin 4 | 0.269 | Upregulated | 4.86E-05 | 8.14E-03 |
| SNX13 | 0.269 | Upregulated | 1.55E-03 | 4.38E-02 |
| SLC2A6 | 0.269 | Upregulated | 2.48E-03 | 5.51E-02 |
| EMILIN2 | 0.269 | Upregulated | 4.95E-02 | 2.66E-01 |
| SLC9A8 | 0.269 | Upregulated | 4.21E-03 | 7.27E-02 |
| HIST1H2AC | 0.270 | Upregulated | 1.69E-02 | 1.52E-01 |
| GUCY1A3 | 0.271 | Upregulated | 2.64E-02 | 1.92E-01 |
| ITPK1 | 0.271 | Upregulated | 1.47E-03 | 4.27E-02 |
| GLUL | 0.271 | Upregulated | 7.89E-03 | 1.01E-01 |
| CETP | 0.271 | Upregulated | 3.20E-02 | 2.13E-01 |
| FBXL13 | 0.273 | Upregulated | 3.35E-02 | 2.19E-01 |
| ATP6V1B2 | 0.273 | Upregulated | 2.15E-03 | 5.15E-02 |
| FAR1 | 0.273 | Upregulated | 1.49E-02 | 1.42E-01 |
| LRRFIP2 | 0.274 | Upregulated | 2.81E-04 | 1.87E-02 |
| IL6R | 0.274 | Upregulated | 2.38E-02 | 1.81E-01 |
| RHBDF2 | 0.274 | Upregulated | 1.33E-04 | 1.27E-02 |
| GLYATL2 | 0.275 | Upregulated | 5.77E-03 | 8.65E-02 |
| ZFYVE16 | 0.275 | Upregulated | 2.10E-04 | 1.63E-02 |
| TICAM2 | 0.275 | Upregulated | 1.65E-04 | 1.43E-02 |

| | | | | |
|---|---|---|---|---|
| STK38L | 0.276 | Upregulated | 1.95E-03 | 4.91E-02 |
| TESK2 | 0.277 | Upregulated | 2.47E-04 | 1.75E-02 |
| RHOT1 | 0.277 | Upregulated | 3.07E-04 | 1.96E-02 |
| CAPNS2 | 0.277 | Upregulated | 1.11E-02 | 1.23E-01 |
| ZNF213 | 0.278 | Upregulated | 3.15E-03 | 6.24E-02 |
| CASP4 | 0.278 | Upregulated | 2.84E-05 | 6.74E-03 |
| LILRB3 | 0.278 | Upregulated | 1.81E-03 | 4.74E-02 |
| PDK3 | 0.278 | Upregulated | 1.20E-03 | 3.89E-02 |
| ANXA1 | 0.279 | Upregulated | 1.18E-02 | 1.26E-01 |
| DNM3 | 0.279 | Upregulated | 2.30E-02 | 1.78E-01 |
| KCNMB1 | 0.279 | Upregulated | 1.25E-02 | 1.30E-01 |
| SRPK1 | 0.280 | Upregulated | 8.57E-03 | 1.06E-01 |
| OR6B2 | 0.281 | Upregulated | 1.42E-03 | 4.21E-02 |
| CEBPB | 0.281 | Upregulated | 1.41E-02 | 1.38E-01 |
| LBA1 | 0.281 | Upregulated | 3.71E-03 | 6.81E-02 |
| GMFG | 0.281 | Upregulated | 4.12E-03 | 7.19E-02 |
| NUCB1 | 0.282 | Upregulated | 2.83E-03 | 5.89E-02 |
| SLC40A1 | 0.282 | Upregulated | 8.85E-03 | 1.08E-01 |
| GSDMD | 0.282 | Upregulated | 7.74E-05 | 9.70E-03 |
| KIAA0232 | 0.282 | Upregulated | 1.11E-02 | 1.22E-01 |
| RIT1 | 0.282 | Upregulated | 2.32E-03 | 5.31E-02 |
| HSPC159 | 0.283 | Upregulated | 2.75E-02 | 1.96E-01 |
| P2RY5 | 0.283 | Upregulated | 1.76E-02 | 1.55E-01 |
| SLC16A5 | 0.284 | Upregulated | 8.25E-03 | 1.04E-01 |
| GBGT1 | 0.284 | Upregulated | 2.08E-02 | 1.69E-01 |
| STAT3 | 0.285 | Upregulated | 5.57E-03 | 8.50E-02 |
| ADAM8 | 0.286 | Upregulated | 3.39E-03 | 6.50E-02 |
| IFITM1 | 0.286 | Upregulated | 8.05E-03 | 1.03E-01 |
| KCNJ10 | 0.286 | Upregulated | 3.53E-02 | 2.25E-01 |
| IL1F9 | 0.286 | Upregulated | 1.39E-02 | 1.37E-01 |
| KIAA1009 | 0.287 | Upregulated | 3.99E-04 | 2.22E-02 |
| ECE1 | 0.287 | Upregulated | 4.17E-02 | 2.45E-01 |
| CCPG1 | 0.288 | Upregulated | 1.41E-02 | 1.38E-01 |
| SLC2A3 | 0.288 | Upregulated | 3.23E-02 | 2.14E-01 |
| PISD | 0.288 | Upregulated | 5.70E-03 | 8.61E-02 |
| S1PR3 | 0.289 | Upregulated | 2.68E-02 | 1.94E-01 |
| CD40 | 0.289 | Upregulated | 5.89E-04 | 2.75E-02 |
| PPP4R1 | 0.289 | Upregulated | 1.57E-04 | 1.37E-02 |
| TMEM154 | 0.289 | Upregulated | 2.97E-03 | 6.03E-02 |
| CSAD | 0.289 | Upregulated | 1.50E-04 | 1.35E-02 |
| CECR6 | 0.291 | Upregulated | 3.03E-02 | 2.06E-01 |
| TSC22D3 | 0.291 | Upregulated | 4.11E-02 | 2.43E-01 |
| UBE2L6 | 0.291 | Upregulated | 6.82E-03 | 9.50E-02 |
| SMPDL3A | 0.291 | Upregulated | 2.87E-02 | 2.01E-01 |
| TMLHE | 0.292 | Upregulated | 2.83E-06 | 2.33E-03 |
| HAUS4 | 0.292 | Upregulated | 1.08E-02 | 1.21E-01 |
| DHRS13 | 0.292 | Upregulated | 1.25E-02 | 1.30E-01 |
| CTRC | 0.292 | Upregulated | 2.08E-03 | 5.08E-02 |
| ARHGAP26 | 0.293 | Upregulated | 1.10E-02 | 1.22E-01 |
| CPEB4 | 0.293 | Upregulated | 2.90E-02 | 2.02E-01 |
| HSD3B7 | 0.294 | Upregulated | 2.62E-02 | 1.91E-01 |

| | | | | |
|---|---|---|---|---|
| PTPRJ | 0.294 | Upregulated | 3.05E-03 | 6.13E-02 |
| AKAP13 | 0.294 | Upregulated | 1.81E-03 | 4.74E-02 |
| TYROBP | 0.295 | Upregulated | 2.45E-03 | 5.47E-02 |
| PHCA | 0.295 | Upregulated | 3.11E-03 | 6.21E-02 |
| MLKL | 0.295 | Upregulated | 2.99E-03 | 6.05E-02 |
| FIG4 | 0.295 | Upregulated | 4.88E-05 | 8.14E-03 |
| MMD | 0.296 | Upregulated | 1.45E-02 | 1.40E-01 |
| ITPRIP | 0.296 | Upregulated | 1.88E-03 | 4.83E-02 |
| MUTYH | 0.296 | Upregulated | 1.78E-04 | 1.49E-02 |
| FYB | 0.296 | Upregulated | 1.83E-03 | 4.76E-02 |
| RENBP | 0.296 | Upregulated | 2.30E-03 | 5.29E-02 |
| SSFA2 | 0.296 | Upregulated | 1.71E-03 | 4.60E-02 |
| MAN2A2 | 0.297 | Upregulated | 5.34E-03 | 8.29E-02 |
| TNNC2 | 0.297 | Upregulated | 3.39E-02 | 2.20E-01 |
| CYTH4 | 0.297 | Upregulated | 7.06E-03 | 9.63E-02 |
| GDPD5 | 0.297 | Upregulated | 1.66E-02 | 1.51E-01 |
| LYN | 0.298 | Upregulated | 4.39E-03 | 7.43E-02 |
| SAMD4A | 0.298 | Upregulated | 1.02E-02 | 1.17E-01 |
| IFIT5 | 0.298 | Upregulated | 2.16E-02 | 1.72E-01 |
| ECGF1 | 0.298 | Upregulated | 6.00E-03 | 8.83E-02 |
| TMC4 | 0.298 | Upregulated | 2.63E-03 | 5.64E-02 |
| IDI1 | 0.299 | Upregulated | 4.31E-02 | 2.49E-01 |
| TMEM164 | 0.300 | Upregulated | 3.40E-04 | 2.04E-02 |
| CMTM6 | 0.300 | Upregulated | 5.31E-04 | 2.59E-02 |
| CALCOCO2 | 0.300 | Upregulated | 1.18E-04 | 1.20E-02 |
| USP15 | 0.301 | Upregulated | 3.02E-04 | 1.94E-02 |
| ERV3 | 0.301 | Upregulated | 2.18E-02 | 1.73E-01 |
| RNF130 | 0.301 | Upregulated | 5.47E-03 | 8.42E-02 |
| SLC19A1 | 0.301 | Upregulated | 9.25E-03 | 1.11E-01 |
| EDG4 | 0.301 | Upregulated | 2.22E-03 | 5.23E-02 |
| NFE2 | 0.302 | Upregulated | 1.82E-02 | 1.58E-01 |
| NETO2 | 0.302 | Upregulated | 2.14E-03 | 5.14E-02 |
| APH1B | 0.302 | Upregulated | 5.24E-03 | 8.19E-02 |
| PFKFB3 | 0.302 | Upregulated | 2.74E-02 | 1.96E-01 |
| ATP8B4 | 0.302 | Upregulated | 8.65E-03 | 1.07E-01 |
| REPS2 | 0.303 | Upregulated | 2.12E-02 | 1.71E-01 |
| MAX | 0.303 | Upregulated | 1.51E-03 | 4.32E-02 |
| ODF3B | 0.303 | Upregulated | 2.55E-06 | 2.33E-03 |
| CEBPD | 0.304 | Upregulated | 1.71E-02 | 1.53E-01 |
| TNFRSF1A | 0.304 | Upregulated | 1.51E-02 | 1.43E-01 |
| SNX20 | 0.304 | Upregulated | 1.56E-03 | 4.39E-02 |
| GAA | 0.305 | Upregulated | 1.07E-02 | 1.20E-01 |
| HTATIP2 | 0.305 | Upregulated | 2.80E-04 | 1.87E-02 |
| NFIB | 0.305 | Upregulated | 2.22E-02 | 1.75E-01 |
| TNFRSF10C | 0.306 | Upregulated | 3.94E-03 | 7.02E-02 |
| RIPK2 | 0.306 | Upregulated | 5.78E-05 | 8.61E-03 |
| ASAP2 | 0.307 | Upregulated | 3.26E-03 | 6.39E-02 |
| IL8 | 0.308 | Upregulated | 3.10E-02 | 2.09E-01 |
| SELPLG | 0.308 | Upregulated | 1.46E-02 | 1.40E-01 |
| SERPINB1 | 0.309 | Upregulated | 1.97E-02 | 1.65E-01 |
| SPI1 | 0.309 | Upregulated | 1.90E-02 | 1.61E-01 |

| | | | | |
|---|---|---|---|---|
| MNDA | 0.309 | Upregulated | 8.11E-03 | 1.03E-01 |
| MTX1 | 0.309 | Upregulated | 1.11E-04 | 1.17E-02 |
| RCVRN | 0.309 | Upregulated | 5.57E-03 | 8.50E-02 |
| MS4A6A | 0.310 | Upregulated | 1.70E-03 | 4.59E-02 |
| LPAR2 | 0.310 | Upregulated | 1.28E-03 | 3.99E-02 |
| RNF141 | 0.311 | Upregulated | 3.22E-04 | 2.00E-02 |
| STAT1 | 0.311 | Upregulated | 7.38E-03 | 9.83E-02 |
| WSB1 | 0.311 | Upregulated | 1.76E-02 | 1.56E-01 |
| ATG16L2 | 0.311 | Upregulated | 8.08E-03 | 1.03E-01 |
| LONRF1 | 0.311 | Upregulated | 2.94E-03 | 6.00E-02 |
| CKAP4 | 0.311 | Upregulated | 4.67E-02 | 2.58E-01 |
| KLHDC8B | 0.312 | Upregulated | 3.09E-02 | 2.09E-01 |
| NFIL3 | 0.312 | Upregulated | 1.08E-02 | 1.21E-01 |
| PLAGL1 | 0.313 | Upregulated | 9.23E-05 | 1.06E-02 |
| NUMB | 0.313 | Upregulated | 1.14E-03 | 3.80E-02 |
| RGL2 | 0.314 | Upregulated | 3.47E-05 | 7.03E-03 |
| IFI30 | 0.314 | Upregulated | 4.13E-03 | 7.19E-02 |
| REM2 | 0.314 | Upregulated | 5.16E-03 | 8.12E-02 |
| MR1 | 0.314 | Upregulated | 1.37E-04 | 1.28E-02 |
| RP2 | 0.314 | Upregulated | 4.27E-03 | 7.33E-02 |
| NDST1 | 0.314 | Upregulated | 3.61E-03 | 6.71E-02 |
| TSEN34 | 0.314 | Upregulated | 1.35E-03 | 4.10E-02 |
| HIST1H3D | 0.314 | Upregulated | 8.28E-03 | 1.04E-01 |
| TRIM27 | 0.314 | Upregulated | 1.15E-04 | 1.18E-02 |
| CAPZA2 | 0.315 | Upregulated | 2.39E-04 | 1.73E-02 |
| FFAR3 | 0.315 | Upregulated | 5.77E-03 | 8.65E-02 |
| LPPR2 | 0.316 | Upregulated | 1.65E-02 | 1.51E-01 |
| JMJD1C | 0.317 | Upregulated | 6.03E-04 | 2.78E-02 |
| PYGL | 0.318 | Upregulated | 4.62E-02 | 2.57E-01 |
| HRH2 | 0.318 | Upregulated | 3.39E-02 | 2.20E-01 |
| TFPI | 0.319 | Upregulated | 2.51E-02 | 1.87E-01 |
| MXD3 | 0.319 | Upregulated | 1.43E-02 | 1.39E-01 |
| PARP9 | 0.320 | Upregulated | 6.56E-03 | 9.30E-02 |
| SELL | 0.320 | Upregulated | 4.15E-03 | 7.22E-02 |
| MAZ | 0.320 | Upregulated | 5.66E-04 | 2.69E-02 |
| XPO6 | 0.320 | Upregulated | 8.41E-03 | 1.05E-01 |
| KLHL2 | 0.320 | Upregulated | 4.17E-02 | 2.45E-01 |
| SVIL | 0.321 | Upregulated | 4.59E-02 | 2.56E-01 |
| HLX | 0.321 | Upregulated | 4.59E-03 | 7.59E-02 |
| ST8SIA4 | 0.321 | Upregulated | 5.68E-05 | 8.56E-03 |
| DDIT3 | 0.321 | Upregulated | 1.82E-03 | 4.76E-02 |
| ATF6 | 0.322 | Upregulated | 2.94E-04 | 1.92E-02 |
| P2RY12 | 0.322 | Upregulated | 3.98E-04 | 2.21E-02 |
| DOK3 | 0.322 | Upregulated | 1.98E-02 | 1.65E-01 |
| CDS2 | 0.323 | Upregulated | 1.14E-03 | 3.79E-02 |
| MPZL1 | 0.324 | Upregulated | 2.28E-02 | 1.77E-01 |
| TRIM21 | 0.325 | Upregulated | 1.89E-04 | 1.52E-02 |
| ABHD2 | 0.325 | Upregulated | 7.52E-04 | 3.09E-02 |
| AIF1 | 0.325 | Upregulated | 4.79E-03 | 7.77E-02 |
| PARP14 | 0.325 | Upregulated | 2.91E-03 | 6.00E-02 |
| HPSE | 0.326 | Upregulated | 2.03E-02 | 1.67E-01 |

| | | | | |
|---|---|---|---|---|
| ARL11 | 0.327 | Upregulated | 1.22E-02 | 1.29E-01 |
| HIST1H2BG | 0.327 | Upregulated | 1.50E-02 | 1.42E-01 |
| TMEM49 | 0.327 | Upregulated | 3.79E-04 | 2.16E-02 |
| DENND5A | 0.328 | Upregulated | 1.45E-04 | 1.33E-02 |
| DDAH2 | 0.328 | Upregulated | 7.77E-03 | 1.01E-01 |
| PPP2R5B | 0.328 | Upregulated | 4.50E-02 | 2.54E-01 |
| CENTD2 | 0.328 | Upregulated | 1.42E-06 | 1.76E-03 |
| ACTN1 | 0.328 | Upregulated | 1.30E-02 | 1.32E-01 |
| BRI3 | 0.328 | Upregulated | 2.35E-03 | 5.34E-02 |
| DDX58 | 0.329 | Upregulated | 8.07E-03 | 1.03E-01 |
| VPS24 | 0.329 | Upregulated | 5.17E-04 | 2.56E-02 |
| CNIH4 | 0.329 | Upregulated | 1.28E-02 | 1.31E-01 |
| RHOU | 0.329 | Upregulated | 1.41E-02 | 1.38E-01 |
| IL17RA | 0.330 | Upregulated | 1.75E-02 | 1.55E-01 |
| RNF144B | 0.330 | Upregulated | 1.66E-02 | 1.51E-01 |
| KIF1B | 0.330 | Upregulated | 1.57E-02 | 1.47E-01 |
| NRBF2 | 0.330 | Upregulated | 8.71E-04 | 3.32E-02 |
| MSL1 | 0.331 | Upregulated | 9.74E-04 | 3.49E-02 |
| RILP | 0.332 | Upregulated | 2.45E-02 | 1.84E-01 |
| PDLIM7 | 0.332 | Upregulated | 3.49E-03 | 6.58E-02 |
| EIF4E3 | 0.332 | Upregulated | 5.42E-04 | 2.62E-02 |
| ITGAX | 0.332 | Upregulated | 6.88E-03 | 9.53E-02 |
| GNG8 | 0.332 | Upregulated | 2.95E-03 | 6.01E-02 |
| ROGDI | 0.332 | Upregulated | 2.02E-03 | 5.02E-02 |
| CD58 | 0.332 | Upregulated | 3.49E-03 | 6.59E-02 |
| EPB41L3 | 0.333 | Upregulated | 1.57E-03 | 4.40E-02 |
| SKAP2 | 0.333 | Upregulated | 3.14E-04 | 1.97E-02 |
| LTBR | 0.333 | Upregulated | 4.84E-03 | 7.81E-02 |
| LPGAT1 | 0.334 | Upregulated | 5.14E-04 | 2.56E-02 |
| PAK1 | 0.334 | Upregulated | 3.25E-04 | 2.00E-02 |
| DHRS12 | 0.334 | Upregulated | 2.84E-04 | 1.88E-02 |
| CYBB | 0.335 | Upregulated | 2.10E-04 | 1.63E-02 |
| CTTN | 0.335 | Upregulated | 3.29E-02 | 2.17E-01 |
| ASPRV1 | 0.335 | Upregulated | 2.75E-02 | 1.96E-01 |
| UBR2 | 0.335 | Upregulated | 8.67E-06 | 4.05E-03 |
| OSCAR | 0.335 | Upregulated | 2.50E-02 | 1.87E-01 |
| LAT2 | 0.335 | Upregulated | 8.71E-04 | 3.32E-02 |
| SLC25A37 | 0.335 | Upregulated | 3.34E-02 | 2.18E-01 |
| BRSK1 | 0.335 | Upregulated | 4.59E-03 | 7.59E-02 |
| TMEM144 | 0.336 | Upregulated | 3.34E-02 | 2.18E-01 |
| ZNF701 | 0.336 | Upregulated | 3.06E-05 | 6.93E-03 |
| PANX2 | 0.336 | Upregulated | 2.09E-02 | 1.69E-01 |
| ETV6 | 0.336 | Upregulated | 1.93E-06 | 1.92E-03 |
| ABLIM3 | 0.338 | Upregulated | 1.01E-02 | 1.16E-01 |
| RERE | 0.338 | Upregulated | 3.52E-05 | 7.03E-03 |
| TRIB1 | 0.338 | Upregulated | 3.93E-03 | 7.01E-02 |
| ENTPD1 | 0.338 | Upregulated | 6.93E-04 | 2.97E-02 |
| SLC45A4 | 0.339 | Upregulated | 3.81E-04 | 2.16E-02 |
| GPR141 | 0.339 | Upregulated | 3.31E-02 | 2.17E-01 |
| FLJ14166 | 0.339 | Upregulated | 8.13E-03 | 1.03E-01 |
| UBXN2B | 0.340 | Upregulated | 5.22E-04 | 2.57E-02 |

| | | | | |
|---|---|---|---|---|
| RAB11FIP1 | 0.340 | Upregulated | 7.64E-04 | 3.11E-02 |
| TNFSF14 | 0.341 | Upregulated | 6.87E-03 | 9.52E-02 |
| PILRA | 0.342 | Upregulated | 1.55E-02 | 1.46E-01 |
| VCPIP1 | 0.342 | Upregulated | 1.83E-05 | 5.68E-03 |
| LACTB | 0.342 | Upregulated | 1.74E-03 | 4.62E-02 |
| MGC33556 | 0.343 | Upregulated | 1.19E-03 | 3.88E-02 |
| NTNG2 | 0.343 | Upregulated | 2.26E-02 | 1.77E-01 |
| GRINA | 0.343 | Upregulated | 1.40E-02 | 1.37E-01 |
| SYTL4 | 0.343 | Upregulated | 1.93E-02 | 1.63E-01 |
| SRBD1 | 0.343 | Upregulated | 5.12E-07 | 9.86E-04 |
| PLXNC1 | 0.343 | Upregulated | 2.56E-02 | 1.89E-01 |
| CEACAM4 | 0.343 | Upregulated | 2.99E-03 | 6.04E-02 |
| LRRC6 | 0.344 | Upregulated | 3.69E-02 | 2.30E-01 |
| FAR2 | 0.344 | Upregulated | 2.79E-03 | 5.84E-02 |
| FBXL5 | 0.344 | Upregulated | 1.15E-03 | 3.81E-02 |
| TYMP | 0.345 | Upregulated | 1.34E-03 | 4.07E-02 |
| SELP | 0.345 | Upregulated | 3.31E-02 | 2.17E-01 |
| PFKFB4 | 0.345 | Upregulated | 6.42E-03 | 9.20E-02 |
| FLOT1 | 0.346 | Upregulated | 6.04E-03 | 8.85E-02 |
| KDM6B | 0.346 | Upregulated | 1.11E-03 | 3.75E-02 |
| FHL1 | 0.346 | Upregulated | 2.09E-02 | 1.69E-01 |
| SECTM1 | 0.346 | Upregulated | 6.17E-06 | 3.49E-03 |
| GCH1 | 0.347 | Upregulated | 1.23E-02 | 1.29E-01 |
| TAPBP | 0.347 | Upregulated | 2.91E-05 | 6.74E-03 |
| GNB4 | 0.347 | Upregulated | 1.11E-03 | 3.75E-02 |
| GPR42 | 0.348 | Upregulated | 3.45E-03 | 6.54E-02 |
| FLJ42957 | 0.349 | Upregulated | 2.07E-03 | 5.06E-02 |
| CYB5R4 | 0.349 | Upregulated | 1.20E-04 | 1.20E-02 |
| C5 | 0.349 | Upregulated | 2.96E-04 | 1.92E-02 |
| RNF19B | 0.349 | Upregulated | 2.89E-03 | 5.97E-02 |
| LATS2 | 0.350 | Upregulated | 1.59E-03 | 4.40E-02 |
| CABP5 | 0.350 | Upregulated | 3.73E-02 | 2.31E-01 |
| FES | 0.350 | Upregulated | 7.00E-03 | 9.59E-02 |
| MEGF9 | 0.350 | Upregulated | 6.90E-03 | 9.55E-02 |
| ETS2 | 0.351 | Upregulated | 1.70E-02 | 1.53E-01 |
| DYNLT1 | 0.352 | Upregulated | 3.09E-04 | 1.96E-02 |
| CTBS | 0.352 | Upregulated | 6.20E-04 | 2.81E-02 |
| C1QA | 0.352 | Upregulated | 1.81E-03 | 4.74E-02 |
| RNASEL | 0.353 | Upregulated | 2.47E-05 | 6.43E-03 |
| PGCP | 0.353 | Upregulated | 9.21E-03 | 1.11E-01 |
| SPOCD1 | 0.353 | Upregulated | 3.11E-02 | 2.10E-01 |
| PSMB9 | 0.353 | Upregulated | 6.64E-05 | 9.21E-03 |
| IL1R2 | 0.353 | Upregulated | 3.53E-02 | 2.25E-01 |
| SLC38A5 | 0.354 | Upregulated | 1.60E-02 | 1.48E-01 |
| HIST1H4H | 0.354 | Upregulated | 2.75E-02 | 1.96E-01 |
| SULT1B1 | 0.355 | Upregulated | 1.54E-02 | 1.45E-01 |
| SAMD9L | 0.355 | Upregulated | 2.20E-02 | 1.74E-01 |
| NAT8B | 0.356 | Upregulated | 2.27E-02 | 1.77E-01 |
| TRAFD1 | 0.356 | Upregulated | 5.96E-04 | 2.76E-02 |
| NDUFAF3 | 0.356 | Upregulated | 4.30E-02 | 2.48E-01 |
| C5AR1 | 0.358 | Upregulated | 1.45E-02 | 1.40E-01 |

| | | | | |
|---|---|---|---|---|
| GLRX | 0.358 | Upregulated | 5.42E-04 | 2.62E-02 |
| IFITM3 | 0.359 | Upregulated | 1.67E-02 | 1.52E-01 |
| ABCA1 | 0.359 | Upregulated | 2.13E-02 | 1.71E-01 |
| NSUN7 | 0.359 | Upregulated | 3.62E-02 | 2.28E-01 |
| TELO2 | 0.361 | Upregulated | 8.04E-06 | 4.03E-03 |
| ZNF185 | 0.362 | Upregulated | 1.48E-02 | 1.41E-01 |
| MCTP2 | 0.363 | Upregulated | 1.01E-02 | 1.17E-01 |
| DRAM1 | 0.363 | Upregulated | 2.64E-03 | 5.65E-02 |
| LYRM1 | 0.363 | Upregulated | 1.17E-05 | 4.74E-03 |
| KIAA0247 | 0.363 | Upregulated | 2.31E-04 | 1.71E-02 |
| ZCCHC6 | 0.364 | Upregulated | 2.49E-04 | 1.76E-02 |
| SAT1 | 0.365 | Upregulated | 1.01E-04 | 1.11E-02 |
| FLJ10357 | 0.366 | Upregulated | 3.15E-03 | 6.24E-02 |
| CCR1 | 0.366 | Upregulated | 1.76E-02 | 1.55E-01 |
| TRIM22 | 0.366 | Upregulated | 4.01E-03 | 7.08E-02 |
| ACRBP | 0.367 | Upregulated | 4.68E-02 | 2.59E-01 |
| BAZ1A | 0.368 | Upregulated | 3.50E-05 | 7.03E-03 |
| AMICA1 | 0.368 | Upregulated | 2.11E-03 | 5.10E-02 |
| HIST2H2AC | 0.368 | Upregulated | 1.25E-02 | 1.30E-01 |
| SLC2A14 | 0.369 | Upregulated | 1.77E-02 | 1.56E-01 |
| GM2A | 0.369 | Upregulated | 5.95E-04 | 2.76E-02 |
| AGPAT9 | 0.370 | Upregulated | 3.49E-02 | 2.23E-01 |
| ALOX12 | 0.370 | Upregulated | 3.91E-02 | 2.37E-01 |
| TMEM55A | 0.370 | Upregulated | 4.86E-04 | 2.48E-02 |
| VNN3 | 0.371 | Upregulated | 2.17E-03 | 5.16E-02 |
| FAM89A | 0.371 | Upregulated | 6.31E-03 | 9.08E-02 |
| DDX60L | 0.371 | Upregulated | 3.18E-03 | 6.28E-02 |
| CDK5RAP2 | 0.373 | Upregulated | 1.29E-02 | 1.32E-01 |
| IL15 | 0.374 | Upregulated | 1.29E-04 | 1.26E-02 |
| FNDC3B | 0.374 | Upregulated | 2.70E-04 | 1.84E-02 |
| SHKBP1 | 0.374 | Upregulated | 2.06E-03 | 5.05E-02 |
| TAP2 | 0.375 | Upregulated | 1.51E-05 | 5.34E-03 |
| BCL3 | 0.375 | Upregulated | 5.92E-03 | 8.77E-02 |
| TM6SF1 | 0.375 | Upregulated | 1.64E-03 | 4.49E-02 |
| SESTD1 | 0.375 | Upregulated | 6.98E-06 | 3.73E-03 |
| FLJ22662 | 0.375 | Upregulated | 1.88E-02 | 1.61E-01 |
| VAMP3 | 0.375 | Upregulated | 4.55E-05 | 7.82E-03 |
| TLR5 | 0.375 | Upregulated | 3.43E-02 | 2.21E-01 |
| ACSBG1 | 0.376 | Upregulated | 2.93E-03 | 6.00E-02 |
| CHMP5 | 0.376 | Upregulated | 7.09E-03 | 9.65E-02 |
| FAM8A1 | 0.376 | Upregulated | 5.85E-05 | 8.61E-03 |
| VSIG4 | 0.377 | Upregulated | 3.80E-03 | 6.86E-02 |
| SIGLEC5 | 0.377 | Upregulated | 1.53E-02 | 1.44E-01 |
| SERPINA1 | 0.377 | Upregulated | 7.54E-03 | 9.92E-02 |
| GNG11 | 0.378 | Upregulated | 1.86E-02 | 1.60E-01 |
| EIF2C4 | 0.378 | Upregulated | 1.03E-03 | 3.60E-02 |
| PELI1 | 0.379 | Upregulated | 1.00E-04 | 1.10E-02 |
| CSDA | 0.379 | Upregulated | 4.40E-02 | 2.51E-01 |
| ZNF230 | 0.379 | Upregulated | 5.39E-06 | 3.36E-03 |
| TSPAN9 | 0.379 | Upregulated | 4.96E-02 | 2.67E-01 |
| RGS18 | 0.379 | Upregulated | 2.37E-03 | 5.37E-02 |

| | | | | |
|---|---|---|---|---|
| NADK | 0.380 | Upregulated | 6.21E-04 | 2.81E-02 |
| IRAK3 | 0.380 | Upregulated | 2.24E-02 | 1.76E-01 |
| SLK | 0.381 | Upregulated | 1.38E-04 | 1.28E-02 |
| OBFC2A | 0.382 | Upregulated | 6.73E-04 | 2.93E-02 |
| TLE3 | 0.383 | Upregulated | 6.50E-03 | 9.27E-02 |
| PPP1R3D | 0.383 | Upregulated | 2.27E-04 | 1.70E-02 |
| SV2A | 0.384 | Upregulated | 2.58E-03 | 5.60E-02 |
| CLEC2B | 0.384 | Upregulated | 1.50E-03 | 4.31E-02 |
| HIST2H2BE | 0.384 | Upregulated | 5.48E-03 | 8.42E-02 |
| TIMP2 | 0.385 | Upregulated | 5.55E-03 | 8.50E-02 |
| HIST1H2BE | 0.386 | Upregulated | 3.28E-03 | 6.41E-02 |
| RRAGD | 0.386 | Upregulated | 1.61E-03 | 4.44E-02 |
| ASPHD2 | 0.386 | Upregulated | 3.20E-05 | 7.03E-03 |
| AIG1 | 0.387 | Upregulated | 8.82E-05 | 1.04E-02 |
| AGTRAP | 0.387 | Upregulated | 2.54E-03 | 5.57E-02 |
| JAK2 | 0.387 | Upregulated | 1.79E-04 | 1.49E-02 |
| PELI2 | 0.388 | Upregulated | 4.44E-05 | 7.76E-03 |
| VNN2 | 0.389 | Upregulated | 3.76E-02 | 2.32E-01 |
| IFNAR1 | 0.390 | Upregulated | 6.03E-05 | 8.75E-03 |
| IL1RN | 0.391 | Upregulated | 3.69E-02 | 2.30E-01 |
| SLC26A8 | 0.392 | Upregulated | 9.09E-03 | 1.10E-01 |
| SIRPD | 0.392 | Upregulated | 7.53E-04 | 3.09E-02 |
| BAZ2B | 0.393 | Upregulated | 5.01E-05 | 8.27E-03 |
| CTSA | 0.394 | Upregulated | 3.57E-03 | 6.68E-02 |
| DOCK5 | 0.394 | Upregulated | 4.07E-03 | 7.16E-02 |
| HIST1H2AE | 0.394 | Upregulated | 9.38E-04 | 3.44E-02 |
| SLC22A15 | 0.397 | Upregulated | 3.31E-03 | 6.43E-02 |
| SEMA4A | 0.397 | Upregulated | 9.58E-03 | 1.12E-01 |
| SLC22A4 | 0.397 | Upregulated | 3.89E-03 | 6.97E-02 |
| TMEM165 | 0.398 | Upregulated | 1.68E-03 | 4.55E-02 |
| CLEC4A | 0.398 | Upregulated | 1.58E-03 | 4.40E-02 |
| DMXL2 | 0.398 | Upregulated | 6.47E-05 | 9.17E-03 |
| SQRDL | 0.398 | Upregulated | 9.14E-05 | 1.05E-02 |
| LMNB1 | 0.399 | Upregulated | 2.71E-03 | 5.74E-02 |
| CLEC4E | 0.399 | Upregulated | 3.45E-03 | 6.54E-02 |
| FCAR | 0.400 | Upregulated | 1.40E-02 | 1.37E-01 |
| CTSS | 0.401 | Upregulated | 2.09E-05 | 5.83E-03 |
| CD300A | 0.402 | Upregulated | 1.12E-04 | 1.17E-02 |
| ITGAM | 0.403 | Upregulated | 7.94E-03 | 1.02E-01 |
| KIAA1598 | 0.404 | Upregulated | 2.93E-03 | 6.00E-02 |
| WDFY1 | 0.404 | Upregulated | 1.21E-04 | 1.20E-02 |
| IRF1 | 0.404 | Upregulated | 1.25E-05 | 4.83E-03 |
| NCF2 | 0.405 | Upregulated | 1.70E-03 | 4.58E-02 |
| MAPK14 | 0.406 | Upregulated | 8.68E-03 | 1.07E-01 |
| NUDT16 | 0.406 | Upregulated | 3.72E-05 | 7.17E-03 |
| GADD45B | 0.407 | Upregulated | 4.16E-05 | 7.52E-03 |
| GLT1D1 | 0.407 | Upregulated | 2.81E-03 | 5.87E-02 |
| BASP1 | 0.407 | Upregulated | 7.46E-03 | 9.88E-02 |
| MBOAT7 | 0.409 | Upregulated | 1.30E-02 | 1.32E-01 |
| SOCS3 | 0.410 | Upregulated | 6.34E-04 | 2.84E-02 |
| STEAP4 | 0.410 | Upregulated | 2.07E-02 | 1.68E-01 |

| | | | | |
|---|---|---|---|---|
| PROK2 | 0.412 | Upregulated | 2.01E-02 | 1.66E-01 |
| FCGR3B | 0.412 | Upregulated | 4.46E-03 | 7.49E-02 |
| HIST2H2AA4 | 0.413 | Upregulated | 4.93E-03 | 7.88E-02 |
| RGL4 | 0.414 | Upregulated | 1.62E-02 | 1.49E-01 |
| ZDHHC18 | 0.414 | Upregulated | 8.15E-04 | 3.22E-02 |
| HK3 | 0.414 | Upregulated | 1.58E-02 | 1.47E-01 |
| DENND3 | 0.414 | Upregulated | 4.18E-04 | 2.28E-02 |
| GYPC | 0.415 | Upregulated | 3.74E-02 | 2.31E-01 |
| SIRPA | 0.415 | Upregulated | 7.36E-03 | 9.81E-02 |
| PROK1 | 0.415 | Upregulated | 1.62E-05 | 5.52E-03 |
| FBXO6 | 0.415 | Upregulated | 7.06E-03 | 9.62E-02 |
| EGLN1 | 0.415 | Upregulated | 3.85E-03 | 6.92E-02 |
| TREML2 | 0.416 | Upregulated | 8.63E-05 | 1.03E-02 |
| FOS | 0.416 | Upregulated | 3.76E-03 | 6.83E-02 |
| CASP1 | 0.416 | Upregulated | 2.20E-05 | 5.88E-03 |
| DUSP1 | 0.418 | Upregulated | 2.38E-03 | 5.39E-02 |
| CATSPER1 | 0.418 | Upregulated | 1.27E-03 | 3.97E-02 |
| TREML1 | 0.418 | Upregulated | 4.75E-02 | 2.61E-01 |
| LRRC25 | 0.419 | Upregulated | 1.38E-03 | 4.15E-02 |
| SNX10 | 0.419 | Upregulated | 1.24E-03 | 3.92E-02 |
| CKLF | 0.420 | Upregulated | 2.47E-04 | 1.75E-02 |
| PLAUR | 0.420 | Upregulated | 3.11E-03 | 6.21E-02 |
| PLXDC2 | 0.420 | Upregulated | 4.24E-04 | 2.30E-02 |
| FCER1G | 0.420 | Upregulated | 2.21E-03 | 5.20E-02 |
| DGAT2 | 0.421 | Upregulated | 1.17E-03 | 3.84E-02 |
| TMEM88 | 0.421 | Upregulated | 3.03E-03 | 6.10E-02 |
| GNAQ | 0.421 | Upregulated | 1.43E-03 | 4.21E-02 |
| SLC16A3 | 0.423 | Upregulated | 2.27E-03 | 5.26E-02 |
| TNFAIP6 | 0.423 | Upregulated | 3.43E-05 | 7.03E-03 |
| IGF2BP3 | 0.424 | Upregulated | 1.69E-02 | 1.53E-01 |
| RAB31 | 0.425 | Upregulated | 1.54E-04 | 1.37E-02 |
| TBC1D8 | 0.425 | Upregulated | 1.27E-03 | 3.97E-02 |
| RNF13 | 0.426 | Upregulated | 1.18E-05 | 4.74E-03 |
| GUK1 | 0.426 | Upregulated | 3.37E-02 | 2.19E-01 |
| TIMM10 | 0.426 | Upregulated | 4.78E-03 | 7.75E-02 |
| LILRA5 | 0.426 | Upregulated | 3.19E-02 | 2.13E-01 |
| HIST2H2AA3 | 0.427 | Upregulated | 3.66E-03 | 6.77E-02 |
| MSRB2 | 0.428 | Upregulated | 1.73E-05 | 5.56E-03 |
| DDEF2 | 0.428 | Upregulated | 2.81E-04 | 1.87E-02 |
| MEFV | 0.428 | Upregulated | 2.02E-04 | 1.60E-02 |
| PRRG4 | 0.429 | Upregulated | 1.60E-06 | 1.92E-03 |
| C1RL | 0.430 | Upregulated | 6.84E-04 | 2.96E-02 |
| FRAT2 | 0.431 | Upregulated | 2.71E-04 | 1.84E-02 |
| BCL6 | 0.431 | Upregulated | 1.14E-02 | 1.24E-01 |
| SLC31A2 | 0.432 | Upregulated | 2.15E-04 | 1.65E-02 |
| NRGN | 0.433 | Upregulated | 4.22E-02 | 2.46E-01 |
| NCF1 | 0.434 | Upregulated | 3.45E-04 | 2.06E-02 |
| ST3GAL6 | 0.435 | Upregulated | 2.03E-04 | 1.60E-02 |
| EPSTI1 | 0.435 | Upregulated | 1.26E-02 | 1.31E-01 |
| CPD | 0.436 | Upregulated | 6.82E-04 | 2.96E-02 |
| PHTF1 | 0.439 | Upregulated | 7.91E-05 | 9.80E-03 |

| | | | | |
|---|---|---|---|---|
| TNFRSF10B | 0.440 | Upregulated | 5.66E-03 | 8.58E-02 |
| ERLIN1 | 0.440 | Upregulated | 2.90E-04 | 1.90E-02 |
| GCA | 0.441 | Upregulated | 3.89E-04 | 2.19E-02 |
| BST1 | 0.441 | Upregulated | 2.15E-03 | 5.15E-02 |
| TREM1 | 0.441 | Upregulated | 1.22E-02 | 1.29E-01 |
| RNF149 | 0.442 | Upregulated | 7.50E-05 | 9.66E-03 |
| AIM2 | 0.442 | Upregulated | 3.19E-04 | 1.99E-02 |
| TLR4 | 0.442 | Upregulated | 5.20E-04 | 2.56E-02 |
| FLJ20489 | 0.442 | Upregulated | 1.65E-02 | 1.51E-01 |
| ST6GALNAC2 | 0.444 | Upregulated | 7.41E-04 | 3.07E-02 |
| JAM3 | 0.446 | Upregulated | 8.91E-03 | 1.08E-01 |
| SMCHD1 | 0.447 | Upregulated | 4.09E-07 | 8.34E-04 |
| SBNO2 | 0.447 | Upregulated | 5.90E-04 | 2.75E-02 |
| ZNF200 | 0.448 | Upregulated | 3.69E-06 | 2.78E-03 |
| FAS | 0.448 | Upregulated | 4.90E-05 | 8.14E-03 |
| CLEC12A | 0.449 | Upregulated | 1.01E-02 | 1.16E-01 |
| SCO2 | 0.449 | Upregulated | 2.46E-04 | 1.75E-02 |
| CD97 | 0.449 | Upregulated | 4.21E-04 | 2.29E-02 |
| PFKFB2 | 0.449 | Upregulated | 4.66E-03 | 7.66E-02 |
| FGL2 | 0.450 | Upregulated | 3.91E-06 | 2.89E-03 |
| HLA-C | 0.450 | Upregulated | 3.68E-03 | 6.79E-02 |
| TSHZ3 | 0.451 | Upregulated | 1.54E-03 | 4.35E-02 |
| CD300LF | 0.451 | Upregulated | 9.50E-05 | 1.07E-02 |
| PTAFR | 0.451 | Upregulated | 4.42E-03 | 7.46E-02 |
| TMEM140 | 0.452 | Upregulated | 2.39E-03 | 5.41E-02 |
| BMX | 0.452 | Upregulated | 4.36E-03 | 7.40E-02 |
| FRMD3 | 0.453 | Upregulated | 7.42E-04 | 3.07E-02 |
| HSPA6 | 0.453 | Upregulated | 1.40E-04 | 1.30E-02 |
| TCN2 | 0.454 | Upregulated | 9.74E-04 | 3.49E-02 |
| TMEM149 | 0.454 | Upregulated | 6.97E-06 | 3.73E-03 |
| FAM129A | 0.455 | Upregulated | 7.30E-03 | 9.79E-02 |
| DUSP3 | 0.456 | Upregulated | 1.27E-05 | 4.84E-03 |
| ADCY3 | 0.458 | Upregulated | 6.64E-03 | 9.36E-02 |
| ZAK | 0.460 | Upregulated | 8.24E-04 | 3.24E-02 |
| APOL1 | 0.460 | Upregulated | 6.34E-07 | 1.05E-03 |
| JUNB | 0.461 | Upregulated | 1.22E-04 | 1.21E-02 |
| CTDSPL | 0.463 | Upregulated | 1.59E-02 | 1.48E-01 |
| CLEC4D | 0.465 | Upregulated | 1.27E-02 | 1.31E-01 |
| VWF | 0.466 | Upregulated | 2.60E-02 | 1.91E-01 |
| RNF24 | 0.467 | Upregulated | 2.61E-03 | 5.62E-02 |
| NCF4 | 0.468 | Upregulated | 1.01E-03 | 3.55E-02 |
| FLJ20273 | 0.470 | Upregulated | 2.13E-03 | 5.12E-02 |
| BEND7 | 0.471 | Upregulated | 5.30E-06 | 3.36E-03 |
| ETV7 | 0.471 | Upregulated | 8.97E-04 | 3.35E-02 |
| TNFSF10 | 0.473 | Upregulated | 2.06E-05 | 5.83E-03 |
| CFH | 0.474 | Upregulated | 4.06E-04 | 2.23E-02 |
| ST3GAL4 | 0.475 | Upregulated | 1.11E-03 | 3.75E-02 |
| FRAT1 | 0.476 | Upregulated | 6.14E-05 | 8.84E-03 |
| SORL1 | 0.477 | Upregulated | 2.96E-03 | 6.01E-02 |
| TSTA3 | 0.477 | Upregulated | 3.70E-02 | 2.31E-01 |
| RALB | 0.479 | Upregulated | 7.72E-05 | 9.70E-03 |

| | | | | |
|---|---|---|---|---|
| SEPX1 | 0.482 | Upregulated | 3.24E-04 | 2.00E-02 |
| TLR2 | 0.483 | Upregulated | 3.56E-03 | 6.66E-02 |
| SPARC | 0.483 | Upregulated | 6.26E-03 | 9.03E-02 |
| MAML3 | 0.483 | Upregulated | 3.84E-04 | 2.17E-02 |
| GBP4 | 0.486 | Upregulated | 1.98E-03 | 4.95E-02 |
| TNFSF13B | 0.487 | Upregulated | 5.31E-05 | 8.37E-03 |
| SLC6A12 | 0.492 | Upregulated | 2.51E-04 | 1.76E-02 |
| GPR160 | 0.492 | Upregulated | 2.85E-03 | 5.92E-02 |
| PF4V1 | 0.497 | Upregulated | 3.39E-02 | 2.20E-01 |
| CSF2RB | 0.498 | Upregulated | 1.23E-04 | 1.21E-02 |
| NT5M | 0.498 | Upregulated | 1.42E-03 | 4.21E-02 |
| MXD1 | 0.500 | Upregulated | 9.40E-05 | 1.07E-02 |
| RTP4 | 0.500 | Upregulated | 1.92E-03 | 4.88E-02 |
| MPL | 0.501 | Upregulated | 2.33E-02 | 1.79E-01 |
| IL1RAP | 0.501 | Upregulated | 2.25E-04 | 1.69E-02 |
| CRISPLD2 | 0.501 | Upregulated | 1.06E-02 | 1.20E-01 |
| CR1 | 0.503 | Upregulated | 5.91E-03 | 8.77E-02 |
| FPR1 | 0.503 | Upregulated | 2.46E-03 | 5.47E-02 |
| GADD45G | 0.503 | Upregulated | 2.11E-05 | 5.83E-03 |
| CSF3R | 0.504 | Upregulated | 2.53E-03 | 5.56E-02 |
| GLDN | 0.504 | Upregulated | 4.82E-03 | 7.79E-02 |
| NOD2 | 0.505 | Upregulated | 1.48E-04 | 1.34E-02 |
| GRAMD1B | 0.509 | Upregulated | 6.64E-05 | 9.21E-03 |
| AQP9 | 0.510 | Upregulated | 1.71E-03 | 4.59E-02 |
| MYBPC3 | 0.511 | Upregulated | 1.24E-04 | 1.22E-02 |
| NFXL1 | 0.513 | Upregulated | 2.63E-03 | 5.64E-02 |
| LIN7A | 0.515 | Upregulated | 2.61E-03 | 5.62E-02 |
| HIST1H2BC | 0.515 | Upregulated | 7.96E-05 | 9.83E-03 |
| SLC6A6 | 0.517 | Upregulated | 2.63E-04 | 1.82E-02 |
| IFIT2 | 0.517 | Upregulated | 1.09E-02 | 1.21E-01 |
| TLR6 | 0.518 | Upregulated | 5.52E-06 | 3.36E-03 |
| SIRPB1 | 0.518 | Upregulated | 2.68E-04 | 1.84E-02 |
| FLVCR2 | 0.518 | Upregulated | 1.84E-06 | 1.92E-03 |
| DYSF | 0.518 | Upregulated | 7.51E-03 | 9.90E-02 |
| VAMP5 | 0.519 | Upregulated | 3.76E-05 | 7.21E-03 |
| OBFC1 | 0.521 | Upregulated | 9.13E-09 | 5.21E-05 |
| RFX2 | 0.521 | Upregulated | 1.65E-03 | 4.51E-02 |
| TNFAIP2 | 0.524 | Upregulated | 7.58E-05 | 9.70E-03 |
| SLAMF8 | 0.524 | Upregulated | 7.65E-05 | 9.70E-03 |
| ZMYND15 | 0.527 | Upregulated | 1.38E-03 | 4.14E-02 |
| EVI2A | 0.528 | Upregulated | 1.51E-03 | 4.33E-02 |
| SOCS1 | 0.529 | Upregulated | 1.31E-03 | 4.01E-02 |
| GBP2 | 0.530 | Upregulated | 2.89E-08 | 1.11E-04 |
| NLRC4 | 0.532 | Upregulated | 1.04E-03 | 3.62E-02 |
| FCGR2A | 0.532 | Upregulated | 1.98E-04 | 1.57E-02 |
| PGS1 | 0.533 | Upregulated | 2.84E-04 | 1.88E-02 |
| LRRK2 | 0.533 | Upregulated | 1.38E-04 | 1.28E-02 |
| FAM26F | 0.535 | Upregulated | 5.04E-04 | 2.53E-02 |
| RBM47 | 0.539 | Upregulated | 2.13E-05 | 5.83E-03 |
| P2RY14 | 0.539 | Upregulated | 3.40E-05 | 7.03E-03 |
| SDPR | 0.539 | Upregulated | 8.84E-04 | 3.33E-02 |

| | | | | |
|---|---|---|---|---|
| RAB20 | 0.541 | Upregulated | 8.19E-05 | 1.00E-02 |
| IL13RA1 | 0.542 | Upregulated | 2.91E-05 | 6.74E-03 |
| MMRN1 | 0.543 | Upregulated | 6.94E-03 | 9.56E-02 |
| LRG1 | 0.543 | Upregulated | 8.76E-03 | 1.07E-01 |
| IL18RAP | 0.544 | Upregulated | 1.31E-03 | 4.03E-02 |
| OSM | 0.544 | Upregulated | 5.10E-04 | 2.54E-02 |
| LAP3 | 0.545 | Upregulated | 7.21E-04 | 3.03E-02 |
| RAB24 | 0.549 | Upregulated | 9.54E-08 | 2.76E-04 |
| IL27 | 0.550 | Upregulated | 2.09E-04 | 1.63E-02 |
| ALDH1A1 | 0.551 | Upregulated | 1.63E-04 | 1.42E-02 |
| STX11 | 0.554 | Upregulated | 1.64E-05 | 5.52E-03 |
| EMR2 | 0.556 | Upregulated | 1.92E-03 | 4.88E-02 |
| TIFA | 0.556 | Upregulated | 5.93E-05 | 8.64E-03 |
| GNG10 | 0.557 | Upregulated | 1.04E-04 | 1.12E-02 |
| APOL6 | 0.558 | Upregulated | 7.35E-06 | 3.81E-03 |
| CLEC1B | 0.559 | Upregulated | 1.30E-02 | 1.32E-01 |
| DSC2 | 0.559 | Upregulated | 2.12E-03 | 5.12E-02 |
| POLB | 0.560 | Upregulated | 2.35E-10 | 8.16E-06 |
| TLR8 | 0.561 | Upregulated | 6.21E-04 | 2.81E-02 |
| HCG27 | 0.561 | Upregulated | 1.72E-06 | 1.92E-03 |
| PPBP | 0.562 | Upregulated | 1.65E-03 | 4.51E-02 |
| CXCL9 | 0.565 | Upregulated | 1.19E-05 | 4.76E-03 |
| SH3BGRL2 | 0.568 | Upregulated | 3.34E-03 | 6.46E-02 |
| NAMPT | 0.571 | Upregulated | 4.29E-03 | 7.35E-02 |
| ADM | 0.571 | Upregulated | 1.54E-03 | 4.36E-02 |
| STX3 | 0.572 | Upregulated | 1.18E-04 | 1.20E-02 |
| KIFC3 | 0.572 | Upregulated | 8.48E-05 | 1.02E-02 |
| CXCL10 | 0.573 | Upregulated | 1.07E-02 | 1.20E-01 |
| ZNF438 | 0.577 | Upregulated | 1.02E-04 | 1.11E-02 |
| FPR2 | 0.578 | Upregulated | 2.26E-03 | 5.25E-02 |
| ALPK1 | 0.579 | Upregulated | 4.13E-05 | 7.52E-03 |
| CMTM2 | 0.581 | Upregulated | 1.47E-02 | 1.41E-01 |
| STK3 | 0.583 | Upregulated | 1.84E-06 | 1.92E-03 |
| MYL9 | 0.584 | Upregulated | 1.06E-02 | 1.20E-01 |
| PROS1 | 0.587 | Upregulated | 3.50E-03 | 6.59E-02 |
| LIMK2 | 0.588 | Upregulated | 9.76E-05 | 1.08E-02 |
| ROPN1L | 0.589 | Upregulated | 3.67E-04 | 2.13E-02 |
| RHD | 0.592 | Upregulated | 4.92E-02 | 2.66E-01 |
| TPST1 | 0.597 | Upregulated | 6.05E-03 | 8.85E-02 |
| TGFA | 0.601 | Upregulated | 1.80E-04 | 1.49E-02 |
| RAB3IL1 | 0.605 | Upregulated | 1.96E-02 | 1.65E-01 |
| SERPING1 | 0.607 | Upregulated | 5.31E-06 | 3.36E-03 |
| FFAR2 | 0.610 | Upregulated | 2.46E-04 | 1.75E-02 |
| KREMEN1 | 0.613 | Upregulated | 1.57E-03 | 4.40E-02 |
| IL8RB | 0.615 | Upregulated | 1.04E-03 | 3.61E-02 |
| C2 | 0.616 | Upregulated | 2.16E-05 | 5.85E-03 |
| ANXA3 | 0.617 | Upregulated | 1.33E-02 | 1.34E-01 |
| GPR97 | 0.617 | Upregulated | 7.41E-04 | 3.07E-02 |
| BEST1 | 0.619 | Upregulated | 5.95E-06 | 3.49E-03 |
| IL18R1 | 0.623 | Upregulated | 2.82E-03 | 5.88E-02 |
| SIPA1L2 | 0.623 | Upregulated | 8.88E-04 | 3.34E-02 |

| | | | | |
|---|---|---|---|---|
| CXCR1 | 0.634 | Upregulated | 8.84E-04 | 3.33E-02 |
| IGSF6 | 0.634 | Upregulated | 1.73E-05 | 5.56E-03 |
| PDCD1LG2 | 0.635 | Upregulated | 6.66E-05 | 9.21E-03 |
| LHFPL2 | 0.636 | Upregulated | 1.17E-06 | 1.63E-03 |
| CMBL | 0.638 | Upregulated | 7.10E-03 | 9.66E-02 |
| PLEK2 | 0.643 | Upregulated | 2.87E-02 | 2.00E-01 |
| GPR109B | 0.646 | Upregulated | 8.29E-05 | 1.01E-02 |
| P2RY13 | 0.651 | Upregulated | 1.81E-05 | 5.68E-03 |
| C1QC | 0.653 | Upregulated | 1.28E-02 | 1.31E-01 |
| PTGS2 | 0.656 | Upregulated | 1.68E-04 | 1.45E-02 |
| SORT1 | 0.663 | Upregulated | 5.47E-05 | 8.39E-03 |
| LPCAT2 | 0.683 | Upregulated | 2.26E-04 | 1.70E-02 |
| F2RL1 | 0.686 | Upregulated | 8.00E-06 | 4.03E-03 |
| SOD2 | 0.690 | Upregulated | 6.86E-05 | 9.26E-03 |
| WARS | 0.691 | Upregulated | 1.06E-05 | 4.58E-03 |
| PSTPIP2 | 0.702 | Upregulated | 1.28E-06 | 1.64E-03 |
| GPR109A | 0.702 | Upregulated | 2.13E-04 | 1.64E-02 |
| DHRS9 | 0.716 | Upregulated | 9.66E-07 | 1.46E-03 |
| IL1B | 0.721 | Upregulated | 5.63E-05 | 8.56E-03 |
| C1QB | 0.732 | Upregulated | 7.78E-03 | 1.01E-01 |
| KCNJ2 | 0.756 | Upregulated | 3.12E-06 | 2.52E-03 |
| KCNJ15 | 0.756 | Upregulated | 2.63E-05 | 6.66E-03 |
| SMARCD3 | 0.760 | Upregulated | 2.77E-05 | 6.66E-03 |
| LY96 | 0.772 | Upregulated | 3.63E-04 | 2.12E-02 |
| INDO | 0.774 | Upregulated | 5.91E-03 | 8.77E-02 |
| FER1L3 | 0.811 | Upregulated | 7.01E-07 | 1.11E-03 |
| CACNA1E | 0.823 | Upregulated | 6.35E-05 | 9.10E-03 |
| ZDHHC19 | 0.841 | Upregulated | 1.64E-02 | 1.50E-01 |
| GBP6 | 0.865 | Upregulated | 4.44E-09 | 4.40E-05 |
| GBP1 | 0.877 | Upregulated | 5.42E-06 | 3.36E-03 |
| CEACAM1 | 0.883 | Upregulated | 1.91E-05 | 5.72E-03 |
| CD274 | 0.883 | Upregulated | 5.46E-07 | 9.96E-04 |
| IDO1 | 0.915 | Upregulated | 9.70E-04 | 3.48E-02 |
| MYOF | 0.919 | Upregulated | 9.41E-08 | 2.76E-04 |
| GK | 0.944 | Upregulated | 2.96E-07 | 6.42E-04 |
| CASP5 | 0.953 | Upregulated | 3.56E-05 | 7.03E-03 |
| CARD17 | 0.960 | Upregulated | 6.01E-07 | 1.04E-03 |
| FCGR1B | 1.051 | Upregulated | 3.98E-08 | 1.38E-04 |
| HLA-DRB1 | 1.100 | Upregulated | 2.34E-02 | 1.80E-01 |
| FCGR1C | 1.110 | Upregulated | 5.07E-09 | 4.40E-05 |
| GBP5 | 1.128 | Upregulated | 3.21E-09 | 4.40E-05 |
| C4BPA | 1.147 | Upregulated | 1.78E-03 | 4.69E-02 |
| FCGR1A | 1.219 | Upregulated | 1.05E-08 | 5.21E-05 |
| BATF2 | 1.223 | Upregulated | 8.13E-09 | 5.21E-05 |
| ANKRD22 | 1.245 | Upregulated | 2.36E-07 | 5.46E-04 |

**Supplementary Table S3b. Differentially expressed genes_South Africa_Male**

| Gene | log FoldChange | Direction of expression | P.Value | adj.P.Val |
|---|---|---|---|---|
| CDKN1C | -0.870 | Downregulated | 9.34E-09 | 1.81E-06 |
| TNFRSF13B | -0.769 | Downregulated | 3.77E-10 | 2.15E-07 |
| GLDC | -0.727 | Downregulated | 4.79E-06 | 1.39E-04 |
| TNFRSF17 | -0.719 | Downregulated | 1.33E-04 | 1.76E-03 |
| LRRN3 | -0.691 | Downregulated | 4.57E-05 | 7.71E-04 |
| VPREB3 | -0.681 | Downregulated | 2.27E-05 | 4.50E-04 |
| CDC20 | -0.665 | Downregulated | 5.50E-06 | 1.54E-04 |
| SCGB3A1 | -0.654 | Downregulated | 7.32E-04 | 6.47E-03 |
| CCNB2 | -0.644 | Downregulated | 2.84E-06 | 9.78E-05 |
| HRK | -0.636 | Downregulated | 4.82E-08 | 5.15E-06 |
| CACNA2D3 | -0.614 | Downregulated | 3.91E-06 | 1.20E-04 |
| CRIP2 | -0.607 | Downregulated | 4.12E-09 | 1.04E-06 |
| KIAA0101 | -0.607 | Downregulated | 8.33E-05 | 1.23E-03 |
| EBI2 | -0.605 | Downregulated | 1.46E-07 | 1.20E-05 |
| CD79A | -0.597 | Downregulated | 2.31E-07 | 1.67E-05 |
| CD52 | -0.593 | Downregulated | 1.20E-05 | 2.77E-04 |
| ITM2C | -0.586 | Downregulated | 2.03E-06 | 7.54E-05 |
| PRSS33 | -0.583 | Downregulated | 3.92E-03 | 2.40E-02 |
| TOP2A | -0.575 | Downregulated | 2.37E-05 | 4.64E-04 |
| ZBED2 | -0.564 | Downregulated | 3.39E-08 | 4.11E-06 |
| TXNDC5 | -0.563 | Downregulated | 2.81E-04 | 3.08E-03 |
| OSBPL10 | -0.560 | Downregulated | 3.81E-06 | 1.18E-04 |
| EIF2B5 | -0.557 | Downregulated | 3.13E-07 | 2.07E-05 |
| AURKB | -0.557 | Downregulated | 4.13E-06 | 1.25E-04 |
| CHI3L2 | -0.556 | Downregulated | 2.43E-06 | 8.70E-05 |
| PNOC | -0.556 | Downregulated | 1.79E-07 | 1.38E-05 |
| HBZ | -0.554 | Downregulated | 4.82E-02 | 1.60E-01 |
| FCRLA | -0.552 | Downregulated | 1.56E-05 | 3.39E-04 |
| SLC16A10 | -0.551 | Downregulated | 4.02E-05 | 7.00E-04 |
| ID3 | -0.550 | Downregulated | 2.87E-06 | 9.86E-05 |
| ICOS | -0.528 | Downregulated | 3.71E-06 | 1.16E-04 |
| AGMAT | -0.527 | Downregulated | 2.20E-08 | 3.09E-06 |
| PDCD2L | -0.525 | Downregulated | 1.00E-09 | 4.34E-07 |
| IGJ | -0.521 | Downregulated | 5.52E-03 | 3.12E-02 |
| CLECL1 | -0.520 | Downregulated | 4.16E-04 | 4.20E-03 |
| CDC45L | -0.518 | Downregulated | 1.26E-04 | 1.69E-03 |
| CCR9 | -0.516 | Downregulated | 2.67E-08 | 3.46E-06 |
| STRBP | -0.514 | Downregulated | 1.36E-06 | 5.74E-05 |
| CD27 | -0.513 | Downregulated | 2.29E-06 | 8.31E-05 |
| GZMK | -0.513 | Downregulated | 7.60E-05 | 1.15E-03 |
| NCAPG | -0.511 | Downregulated | 1.08E-04 | 1.50E-03 |
| MGC29506 | -0.510 | Downregulated | 2.28E-04 | 2.65E-03 |
| GPR18 | -0.507 | Downregulated | 2.98E-07 | 1.99E-05 |
| LIMS2 | -0.503 | Downregulated | 7.80E-07 | 3.91E-05 |
| LEF1 | -0.501 | Downregulated | 4.95E-04 | 4.78E-03 |
| SIRPG | -0.494 | Downregulated | 8.53E-06 | 2.14E-04 |
| CD19 | -0.488 | Downregulated | 2.73E-05 | 5.16E-04 |

| | | | | |
|---|---|---|---|---|
| ANKRD55 | -0.488 | Downregulated | 5.71E-06 | 1.57E-04 |
| AURKA | -0.485 | Downregulated | 5.31E-07 | 2.94E-05 |
| PDK4 | -0.484 | Downregulated | 4.23E-04 | 4.26E-03 |
| CDKAL1 | -0.476 | Downregulated | 1.76E-11 | 2.04E-08 |
| FAM3C | -0.475 | Downregulated | 9.52E-07 | 4.48E-05 |
| GPR44 | -0.474 | Downregulated | 1.72E-03 | 1.26E-02 |
| CENPA | -0.472 | Downregulated | 2.11E-06 | 7.81E-05 |
| CRIP1 | -0.469 | Downregulated | 4.77E-11 | 4.36E-08 |
| TIMD4 | -0.467 | Downregulated | 1.17E-03 | 9.29E-03 |
| AXIN2 | -0.467 | Downregulated | 5.42E-04 | 5.12E-03 |
| TNFRSF13C | -0.464 | Downregulated | 2.23E-05 | 4.44E-04 |
| BCAS4 | -0.464 | Downregulated | 1.89E-07 | 1.43E-05 |
| MCM7 | -0.464 | Downregulated | 1.14E-07 | 9.97E-06 |
| CKS1B | -0.463 | Downregulated | 4.54E-06 | 1.34E-04 |
| GINS2 | -0.461 | Downregulated | 5.42E-05 | 8.80E-04 |
| CXCR3 | -0.455 | Downregulated | 1.21E-07 | 1.04E-05 |
| BUB1 | -0.453 | Downregulated | 7.45E-04 | 6.55E-03 |
| HES4 | -0.452 | Downregulated | 4.88E-03 | 2.84E-02 |
| APOBEC3B | -0.452 | Downregulated | 4.04E-04 | 4.10E-03 |
| FAIM3 | -0.451 | Downregulated | 2.68E-06 | 9.38E-05 |
| CHEK1 | -0.451 | Downregulated | 2.14E-06 | 7.90E-05 |
| THOC3 | -0.450 | Downregulated | 2.23E-06 | 8.13E-05 |
| WDR92 | -0.449 | Downregulated | 5.35E-10 | 2.69E-07 |
| CLDND1 | -0.448 | Downregulated | 3.30E-09 | 9.13E-07 |
| RRM2 | -0.447 | Downregulated | 1.41E-04 | 1.83E-03 |
| MCM2 | -0.445 | Downregulated | 3.38E-05 | 6.11E-04 |
| RFC4 | -0.444 | Downregulated | 1.79E-07 | 1.38E-05 |
| E2F5 | -0.443 | Downregulated | 1.76E-05 | 3.70E-04 |
| POU2AF1 | -0.442 | Downregulated | 2.04E-05 | 4.15E-04 |
| CDKN3 | -0.442 | Downregulated | 6.00E-04 | 5.54E-03 |
| EVL | -0.442 | Downregulated | 2.00E-06 | 7.46E-05 |
| PAICS | -0.441 | Downregulated | 4.17E-08 | 4.61E-06 |
| TYMS | -0.440 | Downregulated | 8.37E-04 | 7.16E-03 |
| KIFC1 | -0.440 | Downregulated | 2.53E-05 | 4.88E-04 |
| IMPDH2 | -0.439 | Downregulated | 1.08E-07 | 9.81E-06 |
| BCAR3 | -0.438 | Downregulated | 1.08E-04 | 1.50E-03 |
| RFC3 | -0.438 | Downregulated | 2.34E-07 | 1.68E-05 |
| MCM4 | -0.437 | Downregulated | 1.21E-05 | 2.80E-04 |
| NSA2 | -0.437 | Downregulated | 7.47E-08 | 7.41E-06 |
| FLJ11795 | -0.437 | Downregulated | 1.95E-05 | 4.01E-04 |
| HIBCH | -0.437 | Downregulated | 1.16E-09 | 4.58E-07 |
| ZNF260 | -0.436 | Downregulated | 3.58E-08 | 4.22E-06 |
| DLGAP5 | -0.435 | Downregulated | 1.61E-04 | 2.03E-03 |
| ALDH18A1 | -0.434 | Downregulated | 3.58E-08 | 4.22E-06 |
| MOBKL2B | -0.433 | Downregulated | 9.88E-05 | 1.40E-03 |
| CEP55 | -0.432 | Downregulated | 4.41E-05 | 7.51E-04 |
| ABCB9 | -0.431 | Downregulated | 5.28E-04 | 5.02E-03 |
| EBF1 | -0.430 | Downregulated | 2.65E-04 | 2.96E-03 |
| CDCA5 | -0.429 | Downregulated | 2.60E-04 | 2.92E-03 |
| FAM102A | -0.429 | Downregulated | 2.55E-05 | 4.90E-04 |
| PRKAR1B | -0.429 | Downregulated | 2.38E-08 | 3.23E-06 |

| | | | | |
|---|---|---|---|---|
| CTLA4 | -0.428 | Downregulated | 2.70E-06 | 9.44E-05 |
| COBLL1 | -0.428 | Downregulated | 2.11E-04 | 2.50E-03 |
| MRPS26 | -0.426 | Downregulated | 2.26E-09 | 7.19E-07 |
| EPHA4 | -0.424 | Downregulated | 9.06E-07 | 4.34E-05 |
| GPRC5D | -0.423 | Downregulated | 7.36E-05 | 1.12E-03 |
| KIAA0125 | -0.423 | Downregulated | 1.32E-05 | 2.99E-04 |
| TRIB2 | -0.423 | Downregulated | 1.18E-05 | 2.74E-04 |
| CCR7 | -0.422 | Downregulated | 2.84E-03 | 1.86E-02 |
| PACSIN1 | -0.422 | Downregulated | 1.01E-04 | 1.42E-03 |
| DNAJC9 | -0.421 | Downregulated | 1.63E-07 | 1.29E-05 |
| ZNF573 | -0.419 | Downregulated | 1.32E-04 | 1.74E-03 |
| RAN | -0.419 | Downregulated | 1.17E-08 | 2.10E-06 |
| CCNA2 | -0.418 | Downregulated | 2.81E-04 | 3.08E-03 |
| TMEM14A | -0.418 | Downregulated | 4.88E-06 | 1.41E-04 |
| PACAP | -0.418 | Downregulated | 5.39E-05 | 8.75E-04 |
| HSPC111 | -0.417 | Downregulated | 1.63E-06 | 6.45E-05 |
| ABLIM1 | -0.417 | Downregulated | 7.85E-05 | 1.18E-03 |
| CCDC99 | -0.416 | Downregulated | 2.00E-06 | 7.46E-05 |
| OLIG1 | -0.416 | Downregulated | 4.71E-04 | 4.61E-03 |
| SLC25A4 | -0.415 | Downregulated | 3.89E-06 | 1.20E-04 |
| CLIP3 | -0.414 | Downregulated | 8.71E-06 | 2.17E-04 |
| QPRT | -0.414 | Downregulated | 2.25E-06 | 8.19E-05 |
| MTSS1 | -0.414 | Downregulated | 4.60E-07 | 2.70E-05 |
| COX11 | -0.413 | Downregulated | 3.59E-07 | 2.25E-05 |
| CD72 | -0.413 | Downregulated | 3.33E-05 | 6.06E-04 |
| HNRNPH1 | -0.412 | Downregulated | 3.32E-06 | 1.08E-04 |
| DENND5B | -0.412 | Downregulated | 6.64E-05 | 1.03E-03 |
| ASPM | -0.411 | Downregulated | 1.87E-04 | 2.28E-03 |
| DPPA4 | -0.411 | Downregulated | 1.44E-04 | 1.87E-03 |
| SLAMF1 | -0.411 | Downregulated | 5.25E-06 | 1.48E-04 |
| MELK | -0.411 | Downregulated | 4.05E-05 | 7.03E-04 |
| CKAP2L | -0.410 | Downregulated | 1.69E-04 | 2.10E-03 |
| NTHL1 | -0.410 | Downregulated | 1.30E-08 | 2.28E-06 |
| FLNB | -0.409 | Downregulated | 1.62E-06 | 6.40E-05 |
| CDCA2 | -0.408 | Downregulated | 2.39E-06 | 8.58E-05 |
| RPLP0 | -0.408 | Downregulated | 5.81E-05 | 9.30E-04 |
| CDCA4 | -0.407 | Downregulated | 1.35E-07 | 1.13E-05 |
| PRC1 | -0.407 | Downregulated | 5.53E-06 | 1.54E-04 |
| PDE7B | -0.406 | Downregulated | 3.78E-05 | 6.69E-04 |
| UFSP2 | -0.406 | Downregulated | 2.73E-07 | 1.87E-05 |
| TMEM156 | -0.405 | Downregulated | 4.62E-08 | 5.00E-06 |
| CDT1 | -0.405 | Downregulated | 2.39E-03 | 1.63E-02 |
| CTPS | -0.404 | Downregulated | 2.88E-07 | 1.94E-05 |
| TRIP13 | -0.404 | Downregulated | 9.65E-05 | 1.37E-03 |
| HSZFP36 | -0.404 | Downregulated | 8.23E-07 | 4.06E-05 |
| ANKRD57 | -0.404 | Downregulated | 8.66E-06 | 2.16E-04 |
| STAP1 | -0.404 | Downregulated | 8.67E-04 | 7.37E-03 |
| RIOK2 | -0.403 | Downregulated | 2.44E-07 | 1.72E-05 |
| IL28RA | -0.403 | Downregulated | 1.41E-05 | 3.14E-04 |
| POLA1 | -0.402 | Downregulated | 4.85E-09 | 1.17E-06 |
| PBK | -0.402 | Downregulated | 1.35E-04 | 1.77E-03 |

| | | | | |
|---|---|---|---|---|
| PHGDH | -0.402 | Downregulated | 3.40E-04 | 3.58E-03 |
| SAMD3 | -0.402 | Downregulated | 1.68E-04 | 2.09E-03 |
| VPREB1 | -0.402 | Downregulated | 1.46E-02 | 6.64E-02 |
| SCML4 | -0.401 | Downregulated | 9.37E-07 | 4.45E-05 |
| KLHL3 | -0.400 | Downregulated | 1.05E-04 | 1.47E-03 |
| SIGIRR | -0.400 | Downregulated | 9.77E-09 | 1.85E-06 |
| FAM84B | -0.398 | Downregulated | 4.67E-05 | 7.82E-04 |
| KIF20B | -0.398 | Downregulated | 3.12E-06 | 1.04E-04 |
| PTPRK | -0.398 | Downregulated | 1.44E-04 | 1.87E-03 |
| SPIB | -0.397 | Downregulated | 2.13E-04 | 2.52E-03 |
| VIL2 | -0.396 | Downregulated | 8.02E-08 | 7.83E-06 |
| PPP3CC | -0.395 | Downregulated | 5.55E-06 | 1.54E-04 |
| BZW2 | -0.394 | Downregulated | 7.11E-07 | 3.66E-05 |
| ELP4 | -0.393 | Downregulated | 8.90E-09 | 1.75E-06 |
| STT3B | -0.392 | Downregulated | 1.81E-06 | 6.98E-05 |
| FAM44B | -0.391 | Downregulated | 5.60E-06 | 1.55E-04 |
| HSPH1 | -0.390 | Downregulated | 6.95E-03 | 3.73E-02 |
| ADA | -0.390 | Downregulated | 9.46E-07 | 4.47E-05 |
| STOML2 | -0.390 | Downregulated | 4.91E-06 | 1.42E-04 |
| GSG2 | -0.390 | Downregulated | 6.93E-06 | 1.81E-04 |
| FAM167A | -0.389 | Downregulated | 1.60E-04 | 2.03E-03 |
| MCEE | -0.388 | Downregulated | 7.08E-05 | 1.08E-03 |
| BOLA3 | -0.388 | Downregulated | 1.35E-06 | 5.73E-05 |
| HLA-DOB | -0.388 | Downregulated | 6.14E-04 | 5.65E-03 |
| SIRT4 | -0.387 | Downregulated | 1.94E-06 | 7.31E-05 |
| KBTBD8 | -0.386 | Downregulated | 5.24E-06 | 1.48E-04 |
| CLEC4F | -0.385 | Downregulated | 1.50E-03 | 1.13E-02 |
| PVRIG | -0.385 | Downregulated | 1.33E-04 | 1.75E-03 |
| ALS2CR4 | -0.384 | Downregulated | 8.42E-07 | 4.13E-05 |
| GTSF1L | -0.384 | Downregulated | 2.85E-08 | 3.60E-06 |
| PASK | -0.383 | Downregulated | 5.02E-04 | 4.83E-03 |
| HDAC1 | -0.383 | Downregulated | 2.32E-09 | 7.24E-07 |
| MAP4K1 | -0.382 | Downregulated | 5.65E-06 | 1.56E-04 |
| CDCA3 | -0.380 | Downregulated | 7.14E-05 | 1.09E-03 |
| FAM134B | -0.379 | Downregulated | 3.08E-05 | 5.70E-04 |
| STAMBPL1 | -0.378 | Downregulated | 1.91E-04 | 2.31E-03 |
| PARP1 | -0.378 | Downregulated | 9.20E-07 | 4.39E-05 |
| PARK7 | -0.378 | Downregulated | 1.42E-04 | 1.85E-03 |
| MCM6 | -0.377 | Downregulated | 3.49E-05 | 6.27E-04 |
| TINP1 | -0.377 | Downregulated | 1.15E-05 | 2.69E-04 |
| NOP58 | -0.377 | Downregulated | 1.05E-04 | 1.46E-03 |
| ZNF593 | -0.377 | Downregulated | 2.98E-07 | 1.99E-05 |
| REXO4 | -0.376 | Downregulated | 6.05E-08 | 6.31E-06 |
| BTLA | -0.375 | Downregulated | 6.33E-04 | 5.77E-03 |
| RAD51C | -0.375 | Downregulated | 3.42E-07 | 2.19E-05 |
| CCR6 | -0.375 | Downregulated | 1.60E-04 | 2.03E-03 |
| SLC38A1 | -0.374 | Downregulated | 2.93E-08 | 3.66E-06 |
| PDCD1 | -0.374 | Downregulated | 8.13E-04 | 6.99E-03 |
| TK1 | -0.374 | Downregulated | 5.57E-04 | 5.23E-03 |
| RRM1 | -0.374 | Downregulated | 7.03E-05 | 1.08E-03 |
| STMN3 | -0.374 | Downregulated | 1.27E-04 | 1.69E-03 |

| | | | | |
|---|---|---|---|---|
| TEX10 | -0.373 | Downregulated | 6.93E-10 | 3.39E-07 |
| HJURP | -0.373 | Downregulated | 2.68E-05 | 5.09E-04 |
| LIMA1 | -0.372 | Downregulated | 5.34E-05 | 8.68E-04 |
| CD79B | -0.372 | Downregulated | 5.08E-04 | 4.87E-03 |
| KIFAP3 | -0.371 | Downregulated | 4.80E-05 | 8.00E-04 |
| STK39 | -0.371 | Downregulated | 1.12E-04 | 1.55E-03 |
| SNRPF | -0.371 | Downregulated | 3.10E-06 | 1.04E-04 |
| CD248 | -0.371 | Downregulated | 1.59E-03 | 1.18E-02 |
| BLR1 | -0.370 | Downregulated | 4.37E-04 | 4.36E-03 |
| GPX7 | -0.370 | Downregulated | 1.64E-05 | 3.52E-04 |
| PPA1 | -0.370 | Downregulated | 2.71E-05 | 5.13E-04 |
| WDR54 | -0.369 | Downregulated | 1.58E-06 | 6.35E-05 |
| FAM159A | -0.369 | Downregulated | 9.77E-06 | 2.38E-04 |
| MEI1 | -0.369 | Downregulated | 3.30E-08 | 4.03E-06 |
| TRIM47 | -0.369 | Downregulated | 4.31E-07 | 2.59E-05 |
| AHI1 | -0.369 | Downregulated | 2.61E-04 | 2.93E-03 |
| ANKRD46 | -0.368 | Downregulated | 3.52E-05 | 6.30E-04 |
| IPO7 | -0.368 | Downregulated | 1.81E-06 | 7.00E-05 |
| FAM83D | -0.368 | Downregulated | 5.62E-06 | 1.55E-04 |
| UBA5 | -0.367 | Downregulated | 2.41E-06 | 8.63E-05 |
| MRPL12 | -0.367 | Downregulated | 2.44E-08 | 3.27E-06 |
| TMEM118 | -0.367 | Downregulated | 2.10E-06 | 7.80E-05 |
| TIMELESS | -0.366 | Downregulated | 1.27E-05 | 2.90E-04 |
| NCR3 | -0.366 | Downregulated | 4.13E-05 | 7.13E-04 |
| ZMYND19 | -0.366 | Downregulated | 3.60E-07 | 2.25E-05 |
| GNG3 | -0.366 | Downregulated | 2.78E-08 | 3.54E-06 |
| LIME1 | -0.365 | Downregulated | 1.26E-05 | 2.88E-04 |
| RAB30 | -0.365 | Downregulated | 2.99E-05 | 5.58E-04 |
| TMEM194A | -0.365 | Downregulated | 6.97E-06 | 1.82E-04 |
| RPL22 | -0.365 | Downregulated | 2.86E-05 | 5.37E-04 |
| NCBP2 | -0.364 | Downregulated | 7.70E-09 | 1.63E-06 |
| CHAF1B | -0.364 | Downregulated | 9.20E-06 | 2.27E-04 |
| FAM113B | -0.363 | Downregulated | 4.52E-05 | 7.64E-04 |
| CD8A | -0.362 | Downregulated | 3.98E-03 | 2.42E-02 |
| HDDC2 | -0.362 | Downregulated | 1.58E-06 | 6.34E-05 |
| DDX50 | -0.362 | Downregulated | 3.36E-06 | 1.09E-04 |
| TBC1D4 | -0.362 | Downregulated | 1.61E-04 | 2.03E-03 |
| EZH2 | -0.361 | Downregulated | 7.16E-06 | 1.86E-04 |
| CD40LG | -0.360 | Downregulated | 2.65E-03 | 1.76E-02 |
| KIAA0355 | -0.360 | Downregulated | 1.80E-06 | 6.95E-05 |
| ZNF215 | -0.360 | Downregulated | 3.93E-06 | 1.20E-04 |
| CUTA | -0.360 | Downregulated | 4.44E-09 | 1.10E-06 |
| SDF2L1 | -0.360 | Downregulated | 6.39E-05 | 9.99E-04 |
| CSE1L | -0.360 | Downregulated | 1.57E-07 | 1.26E-05 |
| CCDC76 | -0.358 | Downregulated | 3.52E-04 | 3.68E-03 |
| SEL1L3 | -0.357 | Downregulated | 9.89E-05 | 1.40E-03 |
| VRK1 | -0.357 | Downregulated | 1.21E-04 | 1.64E-03 |
| PPIL1 | -0.357 | Downregulated | 3.33E-06 | 1.08E-04 |
| LSM2 | -0.357 | Downregulated | 1.84E-10 | 1.21E-07 |
| ZFP82 | -0.357 | Downregulated | 1.01E-05 | 2.44E-04 |
| DENND2D | -0.356 | Downregulated | 4.90E-06 | 1.42E-04 |

| | | | | |
|---|---|---|---|---|
| WDR57 | -0.356 | Downregulated | 3.11E-10 | 1.83E-07 |
| RBM17 | -0.356 | Downregulated | 5.28E-06 | 1.49E-04 |
| GOT1 | -0.356 | Downregulated | 1.03E-07 | 9.43E-06 |
| CCDC134 | -0.355 | Downregulated | 8.99E-11 | 6.78E-08 |
| TULP4 | -0.355 | Downregulated | 6.88E-08 | 6.88E-06 |
| UCHL5 | -0.355 | Downregulated | 9.90E-06 | 2.41E-04 |
| GTPBP4 | -0.355 | Downregulated | 1.16E-05 | 2.71E-04 |
| CCNF | -0.355 | Downregulated | 1.45E-05 | 3.20E-04 |
| EXOSC7 | -0.354 | Downregulated | 4.70E-07 | 2.74E-05 |
| SRPRB | -0.353 | Downregulated | 3.27E-06 | 1.07E-04 |
| POLR2D | -0.353 | Downregulated | 6.09E-11 | 5.28E-08 |
| PARM1 | -0.353 | Downregulated | 6.26E-05 | 9.84E-04 |
| CDCA7L | -0.352 | Downregulated | 4.94E-09 | 1.17E-06 |
| OPN3 | -0.352 | Downregulated | 4.76E-11 | 4.36E-08 |
| DNAJC19 | -0.351 | Downregulated | 5.22E-07 | 2.91E-05 |
| FAM43A | -0.351 | Downregulated | 1.38E-06 | 5.80E-05 |
| ZNF256 | -0.351 | Downregulated | 2.91E-04 | 3.17E-03 |
| PWP1 | -0.351 | Downregulated | 4.04E-08 | 4.57E-06 |
| GLO1 | -0.351 | Downregulated | 1.37E-04 | 1.79E-03 |
| KIF20A | -0.350 | Downregulated | 2.20E-04 | 2.58E-03 |
| EBI3 | -0.350 | Downregulated | 5.47E-04 | 5.16E-03 |
| ATAD2 | -0.350 | Downregulated | 1.39E-05 | 3.12E-04 |
| CDC25C | -0.350 | Downregulated | 1.80E-05 | 3.76E-04 |
| SLAMF6 | -0.349 | Downregulated | 3.00E-06 | 1.01E-04 |
| RFTN1 | -0.349 | Downregulated | 9.06E-07 | 4.34E-05 |
| CPA5 | -0.349 | Downregulated | 1.67E-04 | 2.09E-03 |
| ITK | -0.349 | Downregulated | 5.18E-04 | 4.95E-03 |
| TTC27 | -0.349 | Downregulated | 3.86E-05 | 6.79E-04 |
| TCL1A | -0.348 | Downregulated | 1.20E-02 | 5.72E-02 |
| MPP6 | -0.347 | Downregulated | 7.63E-05 | 1.15E-03 |
| DDX47 | -0.347 | Downregulated | 1.11E-07 | 9.91E-06 |
| POLR1C | -0.347 | Downregulated | 4.07E-10 | 2.20E-07 |
| UCK2 | -0.347 | Downregulated | 1.91E-06 | 7.23E-05 |
| UBE2T | -0.346 | Downregulated | 4.80E-04 | 4.67E-03 |
| TFAM | -0.345 | Downregulated | 2.95E-05 | 5.51E-04 |
| GNL3 | -0.345 | Downregulated | 1.09E-04 | 1.52E-03 |
| HNRNPA1 | -0.344 | Downregulated | 8.85E-09 | 1.75E-06 |
| GLCCI1 | -0.344 | Downregulated | 1.04E-06 | 4.76E-05 |
| DNAJB11 | -0.344 | Downregulated | 2.16E-07 | 1.60E-05 |
| LSM4 | -0.344 | Downregulated | 2.23E-07 | 1.64E-05 |
| LAS1L | -0.344 | Downregulated | 1.16E-08 | 2.10E-06 |
| GSPT2 | -0.344 | Downregulated | 4.89E-06 | 1.42E-04 |
| TPX2 | -0.343 | Downregulated | 1.23E-04 | 1.66E-03 |
| CD83 | -0.343 | Downregulated | 2.12E-05 | 4.27E-04 |
| KMO | -0.343 | Downregulated | 3.80E-04 | 3.91E-03 |
| NT5DC2 | -0.343 | Downregulated | 6.03E-04 | 5.56E-03 |
| CELSR3 | -0.343 | Downregulated | 6.06E-05 | 9.60E-04 |
| HRASLS2 | -0.343 | Downregulated | 5.92E-04 | 5.49E-03 |
| ARMET | -0.343 | Downregulated | 5.09E-05 | 8.37E-04 |
| PSMD10 | -0.343 | Downregulated | 5.30E-07 | 2.94E-05 |
| KRR1 | -0.343 | Downregulated | 4.78E-08 | 5.12E-06 |

| | | | | |
|---|---|---|---|---|
| WDR12 | -0.342 | Downregulated | 5.82E-06 | 1.59E-04 |
| SAAL1 | -0.342 | Downregulated | 1.59E-06 | 6.36E-05 |
| ZFP90 | -0.342 | Downregulated | 2.38E-07 | 1.70E-05 |
| NHP2 | -0.342 | Downregulated | 1.36E-08 | 2.32E-06 |
| CHCHD6 | -0.342 | Downregulated | 3.77E-06 | 1.17E-04 |
| D4S234E | -0.341 | Downregulated | 1.34E-02 | 6.21E-02 |
| SCG5 | -0.341 | Downregulated | 4.12E-08 | 4.59E-06 |
| ADAM23 | -0.341 | Downregulated | 8.01E-04 | 6.92E-03 |
| TUBB3 | -0.341 | Downregulated | 2.60E-05 | 4.98E-04 |
| DDX10 | -0.341 | Downregulated | 2.71E-06 | 9.48E-05 |
| N6AMT2 | -0.340 | Downregulated | 1.08E-09 | 4.41E-07 |
| ILF2 | -0.340 | Downregulated | 6.50E-08 | 6.69E-06 |
| SFTPD | -0.340 | Downregulated | 2.52E-05 | 4.86E-04 |
| CCNE1 | -0.339 | Downregulated | 2.14E-06 | 7.90E-05 |
| FARS2 | -0.339 | Downregulated | 6.98E-09 | 1.52E-06 |
| NXT1 | -0.339 | Downregulated | 4.02E-08 | 4.57E-06 |
| OR2K2 | -0.339 | Downregulated | 5.23E-03 | 2.99E-02 |
| FAM58A | -0.339 | Downregulated | 2.41E-07 | 1.71E-05 |
| PPAN | -0.339 | Downregulated | 4.29E-06 | 1.29E-04 |
| API5 | -0.338 | Downregulated | 2.97E-06 | 1.01E-04 |
| CD320 | -0.338 | Downregulated | 6.29E-05 | 9.87E-04 |
| FCER2 | -0.338 | Downregulated | 2.30E-02 | 9.32E-02 |
| ALG8 | -0.338 | Downregulated | 3.65E-09 | 9.67E-07 |
| ZW10 | -0.338 | Downregulated | 3.93E-08 | 4.52E-06 |
| ATXN7L1 | -0.338 | Downregulated | 3.73E-05 | 6.62E-04 |
| DDX18 | -0.337 | Downregulated | 7.35E-06 | 1.91E-04 |
| PYHIN1 | -0.337 | Downregulated | 1.51E-03 | 1.13E-02 |
| PEBP1 | -0.336 | Downregulated | 3.52E-07 | 2.22E-05 |
| RBM14 | -0.336 | Downregulated | 2.50E-06 | 8.87E-05 |
| BHLHB3 | -0.336 | Downregulated | 5.66E-06 | 1.56E-04 |
| THOC1 | -0.336 | Downregulated | 2.46E-05 | 4.78E-04 |
| KIF4A | -0.336 | Downregulated | 2.59E-04 | 2.92E-03 |
| TNFRSF25 | -0.336 | Downregulated | 8.89E-05 | 1.29E-03 |
| CBR4 | -0.336 | Downregulated | 3.88E-06 | 1.20E-04 |
| FLJ23834 | -0.335 | Downregulated | 3.78E-04 | 3.90E-03 |
| FCRL3 | -0.335 | Downregulated | 3.34E-04 | 3.54E-03 |
| RPL13A | -0.335 | Downregulated | 9.61E-05 | 1.37E-03 |
| PPAT | -0.334 | Downregulated | 2.42E-05 | 4.73E-04 |
| AP3M2 | -0.334 | Downregulated | 2.64E-06 | 9.30E-05 |
| KLF12 | -0.334 | Downregulated | 3.07E-04 | 3.31E-03 |
| PPIH | -0.334 | Downregulated | 1.06E-07 | 9.69E-06 |
| PTDSS1 | -0.334 | Downregulated | 1.15E-07 | 9.99E-06 |
| RBM4B | -0.334 | Downregulated | 1.55E-10 | 1.10E-07 |
| PTTG1 | -0.333 | Downregulated | 2.65E-04 | 2.97E-03 |
| MDFIC | -0.333 | Downregulated | 1.16E-08 | 2.10E-06 |
| CD28 | -0.333 | Downregulated | 3.99E-04 | 4.06E-03 |
| PIGK | -0.333 | Downregulated | 2.23E-04 | 2.60E-03 |
| KLHDC5 | -0.333 | Downregulated | 2.78E-06 | 9.65E-05 |
| MRPL14 | -0.333 | Downregulated | 1.24E-04 | 1.67E-03 |
| NUP37 | -0.333 | Downregulated | 6.63E-07 | 3.47E-05 |
| HMMR | -0.333 | Downregulated | 3.29E-03 | 2.09E-02 |

| | | | | |
|---|---|---|---|---|
| NDUFS3 | -0.333 | Downregulated | 1.35E-07 | 1.13E-05 |
| RPA3 | -0.332 | Downregulated | 6.13E-04 | 5.64E-03 |
| SHMT2 | -0.332 | Downregulated | 1.23E-06 | 5.37E-05 |
| SH2D1A | -0.332 | Downregulated | 4.79E-04 | 4.66E-03 |
| UCKL1 | -0.331 | Downregulated | 1.76E-09 | 6.23E-07 |
| SMYD4 | -0.331 | Downregulated | 2.35E-07 | 1.68E-05 |
| RPAIN | -0.331 | Downregulated | 1.01E-06 | 4.67E-05 |
| MMACHC | -0.331 | Downregulated | 1.40E-06 | 5.86E-05 |
| SET | -0.331 | Downregulated | 5.94E-07 | 3.19E-05 |
| TMEM168 | -0.331 | Downregulated | 1.01E-05 | 2.45E-04 |
| SS18L2 | -0.331 | Downregulated | 3.71E-06 | 1.16E-04 |
| NOL11 | -0.331 | Downregulated | 5.03E-05 | 8.31E-04 |
| GRWD1 | -0.330 | Downregulated | 5.45E-07 | 2.99E-05 |
| TMEM204 | -0.330 | Downregulated | 2.46E-03 | 1.66E-02 |
| PDCD5 | -0.329 | Downregulated | 5.69E-08 | 6.00E-06 |
| NUBP2 | -0.329 | Downregulated | 4.82E-07 | 2.77E-05 |
| RUVBL1 | -0.329 | Downregulated | 7.55E-09 | 1.61E-06 |
| SIVA | -0.329 | Downregulated | 9.35E-10 | 4.21E-07 |
| SAMM50 | -0.328 | Downregulated | 1.06E-08 | 1.98E-06 |
| SDAD1 | -0.328 | Downregulated | 1.55E-08 | 2.56E-06 |
| CD3D | -0.328 | Downregulated | 1.15E-03 | 9.21E-03 |
| TUBB | -0.327 | Downregulated | 8.44E-07 | 4.14E-05 |
| AGPAT5 | -0.327 | Downregulated | 1.90E-04 | 2.31E-03 |
| EIF2A | -0.327 | Downregulated | 3.64E-04 | 3.78E-03 |
| CD24 | -0.326 | Downregulated | 4.96E-03 | 2.88E-02 |
| FAM117B | -0.326 | Downregulated | 5.67E-04 | 5.30E-03 |
| SELS | -0.326 | Downregulated | 3.03E-06 | 1.02E-04 |
| TYSND1 | -0.326 | Downregulated | 4.44E-06 | 1.32E-04 |
| BYSL | -0.326 | Downregulated | 7.13E-06 | 1.85E-04 |
| FLJ46020 | -0.326 | Downregulated | 5.28E-07 | 2.94E-05 |
| MTERFD1 | -0.326 | Downregulated | 1.37E-05 | 3.08E-04 |
| MRPS9 | -0.326 | Downregulated | 3.86E-07 | 2.38E-05 |
| TECR | -0.325 | Downregulated | 5.18E-07 | 2.90E-05 |
| C1QBP | -0.325 | Downregulated | 3.68E-06 | 1.15E-04 |
| HSPA8 | -0.325 | Downregulated | 7.77E-04 | 6.76E-03 |
| TSPAN3 | -0.325 | Downregulated | 4.54E-04 | 4.49E-03 |
| ATF7IP2 | -0.324 | Downregulated | 7.91E-06 | 2.01E-04 |
| ENOPH1 | -0.324 | Downregulated | 1.22E-04 | 1.65E-03 |
| DIS3L | -0.324 | Downregulated | 6.77E-06 | 1.78E-04 |
| COX5A | -0.324 | Downregulated | 6.38E-09 | 1.43E-06 |
| SLC25A15 | -0.324 | Downregulated | 1.59E-08 | 2.58E-06 |
| GPATCH4 | -0.324 | Downregulated | 9.01E-05 | 1.31E-03 |
| ADK | -0.324 | Downregulated | 5.14E-07 | 2.88E-05 |
| SIVA1 | -0.324 | Downregulated | 3.49E-08 | 4.18E-06 |
| NUPL2 | -0.324 | Downregulated | 2.95E-06 | 1.00E-04 |
| NUSAP1 | -0.323 | Downregulated | 3.35E-04 | 3.54E-03 |
| NPM3 | -0.322 | Downregulated | 3.77E-05 | 6.68E-04 |
| HIST1H4C | -0.322 | Downregulated | 1.64E-03 | 1.21E-02 |
| CEP78 | -0.322 | Downregulated | 9.66E-04 | 8.03E-03 |
| NIF3L1 | -0.322 | Downregulated | 1.22E-05 | 2.81E-04 |
| FIGNL1 | -0.322 | Downregulated | 1.64E-07 | 1.29E-05 |

| | | | | |
|---|---|---|---|---|
| ATIC | -0.322 | Downregulated | 4.54E-06 | 1.34E-04 |
| ZNF22 | -0.322 | Downregulated | 1.47E-04 | 1.90E-03 |
| ZNF296 | -0.321 | Downregulated | 8.24E-07 | 4.06E-05 |
| HERC2 | -0.321 | Downregulated | 2.15E-05 | 4.32E-04 |
| CRTC3 | -0.321 | Downregulated | 1.40E-06 | 5.86E-05 |
| RRP15 | -0.321 | Downregulated | 2.42E-05 | 4.73E-04 |
| RALA | -0.321 | Downregulated | 3.55E-10 | 2.06E-07 |
| LMNB2 | -0.321 | Downregulated | 4.69E-06 | 1.37E-04 |
| ZNF121 | -0.320 | Downregulated | 4.23E-05 | 7.27E-04 |
| MRPS24 | -0.320 | Downregulated | 1.21E-07 | 1.04E-05 |
| LCMT2 | -0.320 | Downregulated | 2.82E-06 | 9.75E-05 |
| MCM10 | -0.320 | Downregulated | 2.78E-04 | 3.06E-03 |
| PDE4D | -0.320 | Downregulated | 6.73E-05 | 1.04E-03 |
| PRMT7 | -0.320 | Downregulated | 1.12E-06 | 5.03E-05 |
| IL27RA | -0.320 | Downregulated | 2.31E-08 | 3.17E-06 |
| GPT2 | -0.320 | Downregulated | 1.13E-06 | 5.04E-05 |
| FAM50B | -0.320 | Downregulated | 3.00E-06 | 1.01E-04 |
| ADSL | -0.320 | Downregulated | 1.54E-07 | 1.24E-05 |
| MRPL46 | -0.319 | Downregulated | 2.58E-07 | 1.79E-05 |
| SEH1L | -0.319 | Downregulated | 3.73E-08 | 4.35E-06 |
| NLRP2 | -0.319 | Downregulated | 5.35E-04 | 5.07E-03 |
| MIPEP | -0.319 | Downregulated | 3.62E-06 | 1.14E-04 |
| GINS3 | -0.319 | Downregulated | 2.07E-04 | 2.46E-03 |
| CNOT6 | -0.319 | Downregulated | 6.34E-07 | 3.34E-05 |
| CCNC | -0.318 | Downregulated | 7.58E-05 | 1.14E-03 |
| PET112L | -0.318 | Downregulated | 5.75E-06 | 1.58E-04 |
| MPV17 | -0.318 | Downregulated | 5.56E-09 | 1.29E-06 |
| STAG3L1 | -0.318 | Downregulated | 4.26E-06 | 1.28E-04 |
| DPH5 | -0.318 | Downregulated | 1.41E-05 | 3.14E-04 |
| SERP2 | -0.318 | Downregulated | 5.81E-07 | 3.14E-05 |
| HMGN1 | -0.318 | Downregulated | 9.57E-07 | 4.48E-05 |
| SLC29A1 | -0.318 | Downregulated | 3.41E-06 | 1.10E-04 |
| PIGP | -0.318 | Downregulated | 1.91E-06 | 7.24E-05 |
| MRPS31 | -0.318 | Downregulated | 6.87E-06 | 1.80E-04 |
| BLK | -0.317 | Downregulated | 2.52E-03 | 1.70E-02 |
| DNAJC24 | -0.317 | Downregulated | 6.80E-07 | 3.54E-05 |
| SLC7A6 | -0.317 | Downregulated | 1.56E-06 | 6.29E-05 |
| NSMCE2 | -0.317 | Downregulated | 4.98E-04 | 4.80E-03 |
| CD247 | -0.317 | Downregulated | 4.97E-04 | 4.79E-03 |
| CNIH | -0.317 | Downregulated | 4.69E-05 | 7.85E-04 |
| FAHD2B | -0.317 | Downregulated | 4.84E-07 | 2.77E-05 |
| TMED10 | -0.316 | Downregulated | 6.89E-07 | 3.58E-05 |
| KIAA1324L | -0.316 | Downregulated | 1.04E-03 | 8.51E-03 |
| MRPL35 | -0.316 | Downregulated | 1.81E-05 | 3.78E-04 |
| PLK4 | -0.316 | Downregulated | 1.25E-04 | 1.68E-03 |
| AES | -0.316 | Downregulated | 1.65E-05 | 3.53E-04 |
| RAB33A | -0.315 | Downregulated | 1.13E-07 | 9.97E-06 |
| DDX21 | -0.315 | Downregulated | 7.86E-06 | 2.01E-04 |
| IGSF21 | -0.315 | Downregulated | 1.50E-04 | 1.92E-03 |
| MARCKSL1 | -0.315 | Downregulated | 2.99E-06 | 1.01E-04 |
| RNMTL1 | -0.315 | Downregulated | 2.62E-07 | 1.81E-05 |

| | | | | |
|---|---|---|---|---|
| EXOSC6 | -0.315 | Downregulated | 4.51E-07 | 2.67E-05 |
| CENPM | -0.315 | Downregulated | 5.76E-04 | 5.37E-03 |
| MTA3 | -0.314 | Downregulated | 1.34E-07 | 1.12E-05 |
| MRPL2 | -0.314 | Downregulated | 1.05E-06 | 4.81E-05 |
| NELL2 | -0.314 | Downregulated | 8.22E-03 | 4.25E-02 |
| NOLC1 | -0.314 | Downregulated | 1.60E-06 | 6.38E-05 |
| OGT | -0.314 | Downregulated | 1.90E-05 | 3.92E-04 |
| GTF3A | -0.314 | Downregulated | 2.28E-07 | 1.65E-05 |
| IGFBP4 | -0.314 | Downregulated | 3.88E-05 | 6.83E-04 |
| TMEM194 | -0.314 | Downregulated | 6.09E-06 | 1.64E-04 |
| DONSON | -0.314 | Downregulated | 2.40E-05 | 4.70E-04 |
| FMO4 | -0.314 | Downregulated | 4.06E-07 | 2.47E-05 |
| BCL2L12 | -0.314 | Downregulated | 9.04E-09 | 1.76E-06 |
| SIDT1 | -0.314 | Downregulated | 4.00E-05 | 6.97E-04 |
| KNTC1 | -0.313 | Downregulated | 2.09E-06 | 7.78E-05 |
| TBC1D19 | -0.313 | Downregulated | 8.89E-07 | 4.31E-05 |
| H2AFZ | -0.313 | Downregulated | 3.14E-07 | 2.07E-05 |
| CD96 | -0.313 | Downregulated | 2.02E-04 | 2.41E-03 |
| FASTKD1 | -0.313 | Downregulated | 2.90E-06 | 9.93E-05 |
| BCCIP | -0.313 | Downregulated | 2.28E-08 | 3.14E-06 |
| PAFAH1B3 | -0.312 | Downregulated | 4.16E-06 | 1.26E-04 |
| GAPT | -0.312 | Downregulated | 3.90E-05 | 6.85E-04 |
| POLR2H | -0.312 | Downregulated | 2.15E-06 | 7.92E-05 |
| NOP16 | -0.312 | Downregulated | 8.11E-07 | 4.02E-05 |
| APOD | -0.312 | Downregulated | 4.43E-03 | 2.63E-02 |
| ZNF548 | -0.312 | Downregulated | 8.41E-05 | 1.24E-03 |
| ICT1 | -0.311 | Downregulated | 4.17E-06 | 1.26E-04 |
| CHEK2 | -0.311 | Downregulated | 4.27E-06 | 1.28E-04 |
| GEMIN8 | -0.311 | Downregulated | 1.24E-07 | 1.06E-05 |
| POLR3K | -0.311 | Downregulated | 1.14E-09 | 4.57E-07 |
| CCT8 | -0.311 | Downregulated | 1.93E-08 | 2.85E-06 |
| CDCA7 | -0.311 | Downregulated | 1.98E-06 | 7.41E-05 |
| TOMM22 | -0.311 | Downregulated | 2.54E-07 | 1.77E-05 |
| BIRC5 | -0.310 | Downregulated | 1.71E-04 | 2.12E-03 |
| AUTS2 | -0.310 | Downregulated | 1.33E-03 | 1.03E-02 |
| SAE1 | -0.310 | Downregulated | 4.80E-07 | 2.77E-05 |
| QSOX2 | -0.310 | Downregulated | 6.55E-05 | 1.02E-03 |
| LAX1 | -0.310 | Downregulated | 6.21E-05 | 9.79E-04 |
| GATA3 | -0.310 | Downregulated | 2.45E-05 | 4.78E-04 |
| RPUSD2 | -0.309 | Downregulated | 6.29E-07 | 3.32E-05 |
| PDCD2 | -0.309 | Downregulated | 3.62E-08 | 4.26E-06 |
| KIAA0391 | -0.309 | Downregulated | 1.64E-09 | 5.98E-07 |
| CKB | -0.309 | Downregulated | 3.34E-04 | 3.53E-03 |
| TUBG1 | -0.308 | Downregulated | 2.45E-07 | 1.72E-05 |
| NUP160 | -0.308 | Downregulated | 1.03E-04 | 1.44E-03 |
| ANAPC10 | -0.308 | Downregulated | 4.29E-03 | 2.57E-02 |
| SGPP1 | -0.308 | Downregulated | 2.05E-05 | 4.18E-04 |
| MRPL52 | -0.307 | Downregulated | 4.66E-06 | 1.37E-04 |
| TXNDC12 | -0.307 | Downregulated | 6.89E-03 | 3.70E-02 |
| PRMT1 | -0.307 | Downregulated | 1.15E-06 | 5.15E-05 |
| CD38 | -0.307 | Downregulated | 1.40E-02 | 6.41E-02 |

| | | | | |
|---|---|---|---|---|
| WDR61 | -0.307 | Downregulated | 3.36E-05 | 6.09E-04 |
| PDXP | -0.307 | Downregulated | 5.75E-06 | 1.58E-04 |
| BEX2 | -0.306 | Downregulated | 4.69E-03 | 2.75E-02 |
| SERPINA10 | -0.306 | Downregulated | 7.93E-05 | 1.19E-03 |
| COX10 | -0.306 | Downregulated | 2.84E-08 | 3.60E-06 |
| MDH1 | -0.306 | Downregulated | 1.82E-05 | 3.80E-04 |
| SEC22A | -0.306 | Downregulated | 4.06E-06 | 1.23E-04 |
| EXOSC5 | -0.305 | Downregulated | 7.01E-07 | 3.63E-05 |
| STT3A | -0.305 | Downregulated | 1.07E-06 | 4.88E-05 |
| HSP90B1 | -0.305 | Downregulated | 1.36E-03 | 1.05E-02 |
| PSMB5 | -0.304 | Downregulated | 1.98E-06 | 7.41E-05 |
| HIBADH | -0.304 | Downregulated | 1.15E-04 | 1.58E-03 |
| TMED3 | -0.304 | Downregulated | 1.17E-06 | 5.21E-05 |
| TMEM209 | -0.304 | Downregulated | 7.62E-04 | 6.66E-03 |
| HNRNPA0 | -0.304 | Downregulated | 2.81E-06 | 9.73E-05 |
| ASNSD1 | -0.304 | Downregulated | 5.16E-04 | 4.93E-03 |
| PPIE | -0.304 | Downregulated | 4.72E-07 | 2.74E-05 |
| TOP2B | -0.303 | Downregulated | 9.62E-07 | 4.50E-05 |
| RRS1 | -0.303 | Downregulated | 3.32E-07 | 2.15E-05 |
| KIAA1407 | -0.303 | Downregulated | 3.92E-05 | 6.87E-04 |
| PCMTD2 | -0.303 | Downregulated | 1.94E-03 | 1.38E-02 |
| LPIN1 | -0.302 | Downregulated | 1.98E-03 | 1.40E-02 |
| PRPF4 | -0.302 | Downregulated | 1.85E-11 | 2.06E-08 |
| AGA | -0.302 | Downregulated | 1.11E-04 | 1.54E-03 |
| CDCA8 | -0.302 | Downregulated | 9.71E-06 | 2.37E-04 |
| MRPS30 | -0.301 | Downregulated | 1.43E-06 | 5.93E-05 |
| WDR36 | -0.301 | Downregulated | 5.47E-05 | 8.86E-04 |
| SYT17 | -0.301 | Downregulated | 1.19E-04 | 1.62E-03 |
| PMM1 | -0.301 | Downregulated | 4.43E-03 | 2.63E-02 |
| ZNF236 | -0.301 | Downregulated | 2.48E-05 | 4.80E-04 |
| MCPH1 | -0.301 | Downregulated | 4.40E-13 | 1.70E-09 |
| METAP2 | -0.301 | Downregulated | 5.32E-07 | 2.94E-05 |
| ANAPC7 | -0.301 | Downregulated | 1.20E-06 | 5.27E-05 |
| NUP107 | -0.301 | Downregulated | 1.77E-04 | 2.19E-03 |
| POLE2 | -0.301 | Downregulated | 8.03E-05 | 1.20E-03 |
| MAL | -0.301 | Downregulated | 3.00E-02 | 1.14E-01 |
| NCAPG2 | -0.301 | Downregulated | 4.44E-05 | 7.55E-04 |
| ZNF30 | -0.300 | Downregulated | 5.16E-05 | 8.45E-04 |
| CLYBL | -0.300 | Downregulated | 2.16E-04 | 2.55E-03 |
| SNRNP40 | -0.300 | Downregulated | 6.98E-09 | 1.52E-06 |
| EEF2K | -0.300 | Downregulated | 2.35E-07 | 1.69E-05 |
| UCHL3 | -0.300 | Downregulated | 1.94E-05 | 4.00E-04 |
| CHST12 | -0.300 | Downregulated | 4.12E-03 | 2.49E-02 |
| UNC84A | -0.300 | Downregulated | 9.10E-05 | 1.32E-03 |
| LYAR | -0.300 | Downregulated | 6.45E-07 | 3.38E-05 |
| CPSF6 | -0.300 | Downregulated | 1.06E-05 | 2.52E-04 |
| SFMBT1 | -0.300 | Downregulated | 5.89E-07 | 3.17E-05 |
| GNB1L | -0.300 | Downregulated | 1.89E-08 | 2.81E-06 |
| CYFIP2 | -0.300 | Downregulated | 5.26E-05 | 8.58E-04 |
| CYBASC3 | -0.299 | Downregulated | 1.25E-04 | 1.67E-03 |
| NDUFA12 | -0.299 | Downregulated | 7.86E-06 | 2.01E-04 |

| | | | | |
|---|---|---|---|---|
| HINT1 | -0.299 | Downregulated | 4.18E-02 | 1.44E-01 |
| METTL3 | -0.299 | Downregulated | 2.78E-08 | 3.54E-06 |
| POLE3 | -0.298 | Downregulated | 1.00E-07 | 9.23E-06 |
| SWAP70 | -0.298 | Downregulated | 2.19E-04 | 2.57E-03 |
| RP9 | -0.298 | Downregulated | 4.09E-06 | 1.24E-04 |
| PDHB | -0.298 | Downregulated | 5.75E-08 | 6.05E-06 |
| USE1 | -0.298 | Downregulated | 1.32E-06 | 5.67E-05 |
| NCL | -0.298 | Downregulated | 6.39E-09 | 1.43E-06 |
| MRPL54 | -0.297 | Downregulated | 4.60E-05 | 7.74E-04 |
| PCID2 | -0.297 | Downregulated | 7.79E-07 | 3.91E-05 |
| TMEM160 | -0.297 | Downregulated | 1.82E-08 | 2.76E-06 |
| RNGTT | -0.297 | Downregulated | 7.03E-05 | 1.08E-03 |
| NDUFS5 | -0.297 | Downregulated | 1.13E-03 | 9.06E-03 |
| SMARCAD1 | -0.296 | Downregulated | 6.13E-04 | 5.64E-03 |
| ZDHHC16 | -0.296 | Downregulated | 8.59E-10 | 3.97E-07 |
| MORC2 | -0.296 | Downregulated | 1.01E-05 | 2.44E-04 |
| ARL5A | -0.296 | Downregulated | 2.17E-04 | 2.56E-03 |
| MAGED1 | -0.296 | Downregulated | 1.93E-06 | 7.28E-05 |
| MCM5 | -0.296 | Downregulated | 3.76E-05 | 6.66E-04 |
| CXXC5 | -0.296 | Downregulated | 1.65E-04 | 2.07E-03 |
| IL23A | -0.296 | Downregulated | 2.18E-04 | 2.57E-03 |
| TMEM126A | -0.296 | Downregulated | 1.14E-03 | 9.15E-03 |
| TMEM48 | -0.296 | Downregulated | 1.47E-06 | 6.06E-05 |
| UPF3B | -0.295 | Downregulated | 1.73E-05 | 3.65E-04 |
| CDRT4 | -0.295 | Downregulated | 1.22E-04 | 1.65E-03 |
| CDK2 | -0.295 | Downregulated | 3.99E-09 | 1.02E-06 |
| CDK2AP2 | -0.295 | Downregulated | 1.70E-05 | 3.62E-04 |
| ATL2 | -0.295 | Downregulated | 2.45E-08 | 3.27E-06 |
| MPHOSPH9 | -0.295 | Downregulated | 3.88E-06 | 1.20E-04 |
| FUBP1 | -0.295 | Downregulated | 2.38E-05 | 4.67E-04 |
| SPRED1 | -0.295 | Downregulated | 6.55E-03 | 3.57E-02 |
| IDH3A | -0.295 | Downregulated | 1.11E-07 | 9.91E-06 |
| KRTCAP2 | -0.294 | Downregulated | 2.18E-08 | 3.07E-06 |
| WEE1 | -0.294 | Downregulated | 3.11E-04 | 3.34E-03 |
| CCR4 | -0.294 | Downregulated | 3.11E-06 | 1.04E-04 |
| FNBP4 | -0.294 | Downregulated | 7.92E-04 | 6.86E-03 |
| LY9 | -0.294 | Downregulated | 1.55E-04 | 1.98E-03 |
| LAGE3 | -0.294 | Downregulated | 6.23E-05 | 9.81E-04 |
| UHRF1 | -0.294 | Downregulated | 2.47E-05 | 4.78E-04 |
| SLC35F2 | -0.294 | Downregulated | 6.21E-05 | 9.79E-04 |
| CENPP | -0.294 | Downregulated | 3.44E-05 | 6.19E-04 |
| PGAM5 | -0.293 | Downregulated | 2.36E-10 | 1.46E-07 |
| TIMM9 | -0.293 | Downregulated | 1.09E-06 | 4.93E-05 |
| TIMM44 | -0.293 | Downregulated | 2.32E-06 | 8.39E-05 |
| POLR3B | -0.293 | Downregulated | 1.24E-05 | 2.86E-04 |
| SNRPG | -0.293 | Downregulated | 2.27E-02 | 9.23E-02 |
| TSPYL5 | -0.293 | Downregulated | 5.26E-04 | 5.01E-03 |
| LARS2 | -0.293 | Downregulated | 1.62E-05 | 3.47E-04 |
| ALDH7A1 | -0.293 | Downregulated | 2.10E-05 | 4.24E-04 |
| LBH | -0.293 | Downregulated | 1.27E-05 | 2.90E-04 |
| DDX55 | -0.293 | Downregulated | 2.83E-06 | 9.75E-05 |

| | | | | |
|---|---|---|---|---|
| NDUFAF2 | -0.293 | Downregulated | 1.54E-04 | 1.96E-03 |
| DTX3 | -0.292 | Downregulated | 4.98E-05 | 8.25E-04 |
| PDIA6 | -0.292 | Downregulated | 1.06E-09 | 4.40E-07 |
| POLR2I | -0.292 | Downregulated | 7.61E-08 | 7.52E-06 |
| CCDC34 | -0.291 | Downregulated | 1.75E-03 | 1.27E-02 |
| DCPS | -0.291 | Downregulated | 2.48E-06 | 8.84E-05 |
| TLE1 | -0.291 | Downregulated | 1.42E-03 | 1.09E-02 |
| THEM4 | -0.291 | Downregulated | 7.82E-04 | 6.79E-03 |
| NSUN6 | -0.291 | Downregulated | 4.72E-07 | 2.74E-05 |
| HSPA9 | -0.291 | Downregulated | 1.15E-05 | 2.70E-04 |
| KIF2C | -0.291 | Downregulated | 6.65E-05 | 1.03E-03 |
| TXNDC14 | -0.291 | Downregulated | 1.68E-08 | 2.62E-06 |
| FOXO1 | -0.291 | Downregulated | 1.13E-04 | 1.56E-03 |
| TARBP1 | -0.291 | Downregulated | 5.37E-04 | 5.08E-03 |
| NSMCE4A | -0.291 | Downregulated | 2.44E-04 | 2.79E-03 |
| DGUOK | -0.291 | Downregulated | 3.77E-11 | 3.63E-08 |
| PPIAL4A | -0.290 | Downregulated | 3.93E-05 | 6.88E-04 |
| BPNT1 | -0.290 | Downregulated | 1.58E-05 | 3.41E-04 |
| DNAJC8 | -0.290 | Downregulated | 2.04E-07 | 1.53E-05 |
| CSTF3 | -0.290 | Downregulated | 9.06E-07 | 4.34E-05 |
| SDCCAG3 | -0.290 | Downregulated | 1.59E-07 | 1.27E-05 |
| COPS3 | -0.290 | Downregulated | 9.81E-07 | 4.54E-05 |
| TOMM20 | -0.289 | Downregulated | 3.60E-04 | 3.74E-03 |
| RBM15 | -0.289 | Downregulated | 1.64E-05 | 3.52E-04 |
| TRAF3IP3 | -0.289 | Downregulated | 3.70E-06 | 1.16E-04 |
| PRKCH | -0.289 | Downregulated | 1.25E-03 | 9.82E-03 |
| EZR | -0.289 | Downregulated | 9.05E-07 | 4.34E-05 |
| ZNF33B | -0.289 | Downregulated | 2.66E-05 | 5.06E-04 |
| CD2 | -0.289 | Downregulated | 1.05E-03 | 8.57E-03 |
| CDC23 | -0.289 | Downregulated | 7.71E-06 | 1.97E-04 |
| DKC1 | -0.288 | Downregulated | 4.66E-06 | 1.37E-04 |
| FEN1 | -0.288 | Downregulated | 1.86E-05 | 3.86E-04 |
| FBXO22 | -0.288 | Downregulated | 1.77E-06 | 6.86E-05 |
| METAP1 | -0.288 | Downregulated | 5.79E-07 | 3.14E-05 |
| PAAF1 | -0.288 | Downregulated | 1.12E-06 | 5.03E-05 |
| DCTPP1 | -0.288 | Downregulated | 3.42E-06 | 1.10E-04 |
| EBNA1BP2 | -0.287 | Downregulated | 4.81E-05 | 8.01E-04 |
| GATM | -0.287 | Downregulated | 1.13E-03 | 9.07E-03 |
| ZNF589 | -0.287 | Downregulated | 3.34E-07 | 2.15E-05 |
| SMARCE1 | -0.286 | Downregulated | 1.31E-05 | 2.97E-04 |
| HPGD | -0.286 | Downregulated | 1.11E-02 | 5.38E-02 |
| OSBPL9 | -0.286 | Downregulated | 6.28E-05 | 9.86E-04 |
| ZNF285A | -0.286 | Downregulated | 5.49E-04 | 5.17E-03 |
| FBLN2 | -0.286 | Downregulated | 2.03E-03 | 1.43E-02 |
| SLC25A29 | -0.286 | Downregulated | 3.90E-04 | 3.99E-03 |
| SEC11A | -0.286 | Downregulated | 1.28E-06 | 5.53E-05 |
| EIF2S3 | -0.286 | Downregulated | 6.98E-11 | 5.76E-08 |
| GINS4 | -0.286 | Downregulated | 4.03E-06 | 1.23E-04 |
| GPR55 | -0.286 | Downregulated | 2.70E-05 | 5.12E-04 |
| CPNE5 | -0.286 | Downregulated | 1.57E-03 | 1.17E-02 |
| GLRX3 | -0.285 | Downregulated | 5.58E-04 | 5.24E-03 |

| | | | | |
|---|---|---|---|---|
| RRP1B | -0.285 | Downregulated | 4.20E-05 | 7.23E-04 |
| KIF22 | -0.285 | Downregulated | 5.98E-07 | 3.20E-05 |
| GOT2 | -0.285 | Downregulated | 1.08E-06 | 4.89E-05 |
| ACTL6A | -0.285 | Downregulated | 9.01E-06 | 2.23E-04 |
| RSRC1 | -0.285 | Downregulated | 6.37E-05 | 9.96E-04 |
| RAPGEF5 | -0.284 | Downregulated | 5.94E-04 | 5.50E-03 |
| CLASP2 | -0.284 | Downregulated | 5.37E-05 | 8.72E-04 |
| HIRIP3 | -0.284 | Downregulated | 4.28E-06 | 1.29E-04 |
| MANEA | -0.284 | Downregulated | 6.67E-04 | 6.00E-03 |
| ZNF286A | -0.284 | Downregulated | 3.48E-06 | 1.11E-04 |
| PLS3 | -0.284 | Downregulated | 2.86E-05 | 5.38E-04 |
| TBCA | -0.284 | Downregulated | 1.01E-05 | 2.43E-04 |
| PARP15 | -0.284 | Downregulated | 2.63E-03 | 1.76E-02 |
| CENPE | -0.283 | Downregulated | 2.24E-04 | 2.62E-03 |
| TDP1 | -0.283 | Downregulated | 1.60E-05 | 3.45E-04 |
| CHAF1A | -0.283 | Downregulated | 1.52E-05 | 3.31E-04 |
| KLHL20 | -0.283 | Downregulated | 2.32E-06 | 8.39E-05 |
| CSNK2A1 | -0.283 | Downregulated | 6.71E-14 | 3.33E-10 |
| SRM | -0.283 | Downregulated | 7.89E-05 | 1.18E-03 |
| TWISTNB | -0.282 | Downregulated | 2.91E-04 | 3.17E-03 |
| AK3 | -0.282 | Downregulated | 2.46E-06 | 8.77E-05 |
| ZNF462 | -0.282 | Downregulated | 3.10E-05 | 5.73E-04 |
| LSM8 | -0.282 | Downregulated | 2.39E-04 | 2.74E-03 |
| EIF4A2 | -0.282 | Downregulated | 1.71E-04 | 2.12E-03 |
| DRG1 | -0.282 | Downregulated | 3.64E-08 | 4.26E-06 |
| RPS5 | -0.282 | Downregulated | 4.98E-04 | 4.80E-03 |
| QRSL1 | -0.281 | Downregulated | 3.51E-07 | 2.22E-05 |
| CKAP5 | -0.281 | Downregulated | 4.46E-07 | 2.64E-05 |
| BFSP2 | -0.281 | Downregulated | 1.04E-04 | 1.46E-03 |
| NDUFB8 | -0.281 | Downregulated | 3.52E-08 | 4.20E-06 |
| PLSCR3 | -0.281 | Downregulated | 3.42E-06 | 1.10E-04 |
| ZNF621 | -0.281 | Downregulated | 1.85E-05 | 3.84E-04 |
| APEX1 | -0.281 | Downregulated | 2.15E-05 | 4.32E-04 |
| MED20 | -0.280 | Downregulated | 9.77E-06 | 2.38E-04 |
| RNASEH2A | -0.280 | Downregulated | 8.30E-05 | 1.23E-03 |
| NAT10 | -0.280 | Downregulated | 5.61E-06 | 1.55E-04 |
| PIK3C2B | -0.280 | Downregulated | 8.01E-04 | 6.92E-03 |
| RAE1 | -0.280 | Downregulated | 1.14E-07 | 9.97E-06 |
| LTV1 | -0.280 | Downregulated | 2.99E-05 | 5.58E-04 |
| SMC2 | -0.280 | Downregulated | 2.57E-06 | 9.07E-05 |
| VPS35 | -0.280 | Downregulated | 1.61E-06 | 6.38E-05 |
| WDR21A | -0.280 | Downregulated | 1.85E-05 | 3.85E-04 |
| MYB | -0.280 | Downregulated | 1.38E-04 | 1.80E-03 |
| MAT2A | -0.279 | Downregulated | 1.75E-06 | 6.81E-05 |
| MTG1 | -0.279 | Downregulated | 2.35E-05 | 4.62E-04 |
| CD5 | -0.279 | Downregulated | 2.86E-03 | 1.87E-02 |
| RPP21 | -0.279 | Downregulated | 5.06E-09 | 1.20E-06 |
| MFGE8 | -0.279 | Downregulated | 1.49E-03 | 1.12E-02 |
| NUP85 | -0.279 | Downregulated | 7.37E-09 | 1.59E-06 |
| ASF1B | -0.279 | Downregulated | 1.01E-04 | 1.42E-03 |
| BCS1L | -0.279 | Downregulated | 1.57E-05 | 3.39E-04 |

| | | | | |
|---|---|---|---|---|
| ARHGEF5L | -0.278 | Downregulated | 6.39E-05 | 9.99E-04 |
| ATP6V0E2 | -0.278 | Downregulated | 5.69E-04 | 5.31E-03 |
| NIT2 | -0.278 | Downregulated | 1.15E-05 | 2.70E-04 |
| IAH1 | -0.278 | Downregulated | 2.32E-05 | 4.58E-04 |
| SIGLEC8 | -0.278 | Downregulated | 2.29E-04 | 2.66E-03 |
| PRPS1 | -0.278 | Downregulated | 2.61E-05 | 4.99E-04 |
| GTF2E2 | -0.278 | Downregulated | 8.94E-07 | 4.32E-05 |
| SNX5 | -0.278 | Downregulated | 3.28E-06 | 1.07E-04 |
| KIF11 | -0.277 | Downregulated | 3.17E-03 | 2.03E-02 |
| ELL3 | -0.277 | Downregulated | 1.75E-04 | 2.16E-03 |
| GOLGA3 | -0.277 | Downregulated | 1.27E-08 | 2.26E-06 |
| LPAR5 | -0.277 | Downregulated | 2.23E-04 | 2.61E-03 |
| CCDC21 | -0.277 | Downregulated | 1.00E-05 | 2.43E-04 |
| AIMP2 | -0.277 | Downregulated | 9.68E-08 | 9.00E-06 |
| DOCK9 | -0.277 | Downregulated | 1.71E-03 | 1.25E-02 |
| SLC4A7 | -0.277 | Downregulated | 2.72E-04 | 3.02E-03 |
| AHCY | -0.277 | Downregulated | 1.54E-05 | 3.36E-04 |
| EPPB9 | -0.277 | Downregulated | 1.32E-04 | 1.75E-03 |
| ATRIP | -0.277 | Downregulated | 2.27E-08 | 3.14E-06 |
| CCDC102A | -0.277 | Downregulated | 9.61E-04 | 8.00E-03 |
| THOC4 | -0.277 | Downregulated | 3.32E-07 | 2.15E-05 |
| ZDHHC23 | -0.276 | Downregulated | 3.59E-04 | 3.74E-03 |
| MRPS15 | -0.276 | Downregulated | 5.23E-06 | 1.48E-04 |
| RAB7L1 | -0.276 | Downregulated | 2.75E-06 | 9.57E-05 |
| POLR2F | -0.276 | Downregulated | 5.37E-07 | 2.95E-05 |
| MGC15763 | -0.276 | Downregulated | 1.23E-02 | 5.81E-02 |
| PAQR7 | -0.276 | Downregulated | 4.03E-05 | 7.00E-04 |
| CRELD2 | -0.276 | Downregulated | 7.49E-06 | 1.93E-04 |
| CENPBD1 | -0.276 | Downregulated | 1.98E-05 | 4.06E-04 |
| CYP2J2 | -0.276 | Downregulated | 6.70E-04 | 6.01E-03 |
| NMT2 | -0.276 | Downregulated | 1.86E-03 | 1.34E-02 |
| RSL1D1 | -0.276 | Downregulated | 3.23E-06 | 1.06E-04 |
| RQCD1 | -0.276 | Downregulated | 7.87E-06 | 2.01E-04 |
| ATPAF2 | -0.276 | Downregulated | 7.04E-07 | 3.64E-05 |
| SFRS6 | -0.275 | Downregulated | 4.82E-06 | 1.40E-04 |
| FAM24B | -0.275 | Downregulated | 4.75E-07 | 2.75E-05 |
| SLC15A2 | -0.275 | Downregulated | 3.37E-05 | 6.09E-04 |
| TARP | -0.275 | Downregulated | 2.15E-02 | 8.88E-02 |
| SAC3D1 | -0.275 | Downregulated | 8.55E-09 | 1.74E-06 |
| TCERG1 | -0.275 | Downregulated | 1.88E-06 | 7.15E-05 |
| UQCRFS1 | -0.274 | Downregulated | 2.51E-07 | 1.76E-05 |
| GPN1 | -0.274 | Downregulated | 3.91E-06 | 1.20E-04 |
| CCT4 | -0.274 | Downregulated | 4.67E-06 | 1.37E-04 |
| MCM3 | -0.274 | Downregulated | 5.01E-06 | 1.44E-04 |
| CR2 | -0.274 | Downregulated | 1.45E-02 | 6.59E-02 |
| COMMD3 | -0.274 | Downregulated | 4.38E-04 | 4.36E-03 |
| KIAA1826 | -0.274 | Downregulated | 4.30E-04 | 4.30E-03 |
| PDIA5 | -0.274 | Downregulated | 1.73E-04 | 2.14E-03 |
| CCDC127 | -0.274 | Downregulated | 1.10E-07 | 9.87E-06 |
| WBSCR22 | -0.274 | Downregulated | 1.60E-06 | 6.38E-05 |
| ARPC5L | -0.274 | Downregulated | 2.18E-06 | 7.98E-05 |

| | | | | |
|---|---|---|---|---|
| EMG1 | -0.274 | Downregulated | 3.35E-04 | 3.54E-03 |
| OIP5 | -0.274 | Downregulated | 1.90E-04 | 2.30E-03 |
| RCAN3 | -0.274 | Downregulated | 1.62E-04 | 2.04E-03 |
| LIPN | -0.273 | Downregulated | 5.07E-03 | 2.92E-02 |
| METTL5 | -0.273 | Downregulated | 5.15E-04 | 4.92E-03 |
| FKBP11 | -0.273 | Downregulated | 3.77E-03 | 2.33E-02 |
| AOF2 | -0.273 | Downregulated | 9.40E-07 | 4.45E-05 |
| NDUFAF1 | -0.273 | Downregulated | 1.18E-03 | 9.36E-03 |
| MYH10 | -0.273 | Downregulated | 2.51E-04 | 2.85E-03 |
| CSTB | -0.273 | Downregulated | 1.45E-07 | 1.19E-05 |
| PDCL3 | -0.273 | Downregulated | 2.44E-07 | 1.72E-05 |
| GNL2 | -0.273 | Downregulated | 6.70E-05 | 1.03E-03 |
| UBAP2 | -0.273 | Downregulated | 1.34E-07 | 1.12E-05 |
| ENDOG | -0.272 | Downregulated | 1.76E-05 | 3.70E-04 |
| DARS | -0.272 | Downregulated | 1.22E-06 | 5.37E-05 |
| CDK5RAP1 | -0.272 | Downregulated | 2.44E-08 | 3.27E-06 |
| CCDC106 | -0.272 | Downregulated | 1.15E-03 | 9.19E-03 |
| TCTEX1D2 | -0.272 | Downregulated | 3.13E-04 | 3.35E-03 |
| MRPL36 | -0.272 | Downregulated | 1.72E-04 | 2.13E-03 |
| CCDC66 | -0.272 | Downregulated | 2.11E-04 | 2.50E-03 |
| ZBTB9 | -0.272 | Downregulated | 1.08E-06 | 4.89E-05 |
| TP53 | -0.272 | Downregulated | 8.18E-08 | 7.92E-06 |
| RAGE | -0.272 | Downregulated | 1.68E-03 | 1.23E-02 |
| DPAGT1 | -0.272 | Downregulated | 4.66E-07 | 2.72E-05 |
| GFPT1 | -0.272 | Downregulated | 7.91E-04 | 6.86E-03 |
| ITGB1BP1 | -0.272 | Downregulated | 5.00E-06 | 1.44E-04 |
| HNRNPD | -0.272 | Downregulated | 2.03E-07 | 1.53E-05 |
| DET1 | -0.271 | Downregulated | 4.63E-07 | 2.72E-05 |
| CAMK1 | -0.271 | Downregulated | 1.44E-03 | 1.10E-02 |
| ETFB | -0.271 | Downregulated | 1.35E-06 | 5.73E-05 |
| CXXC1 | -0.271 | Downregulated | 1.13E-07 | 9.97E-06 |
| ACAD11 | -0.271 | Downregulated | 2.26E-04 | 2.63E-03 |
| HADH | -0.271 | Downregulated | 1.18E-04 | 1.61E-03 |
| EPRS | -0.271 | Downregulated | 3.25E-05 | 5.94E-04 |
| ZNF35 | -0.271 | Downregulated | 3.11E-06 | 1.04E-04 |
| RPL36 | -0.270 | Downregulated | 1.18E-06 | 5.22E-05 |
| RDH13 | -0.270 | Downregulated | 2.62E-05 | 5.00E-04 |
| NIP7 | -0.270 | Downregulated | 2.95E-04 | 3.20E-03 |
| THNSL1 | -0.270 | Downregulated | 2.56E-05 | 4.92E-04 |
| PEX11B | -0.270 | Downregulated | 1.10E-07 | 9.89E-06 |
| ZNF259 | -0.270 | Downregulated | 8.90E-08 | 8.44E-06 |
| ATP5J | -0.270 | Downregulated | 1.09E-03 | 8.84E-03 |
| TACO1 | -0.269 | Downregulated | 5.10E-04 | 4.89E-03 |
| TUBB2B | -0.269 | Downregulated | 3.45E-05 | 6.20E-04 |
| SLC41A3 | -0.269 | Downregulated | 6.49E-05 | 1.01E-03 |
| PIK3IP1 | -0.269 | Downregulated | 9.86E-03 | 4.90E-02 |
| RPS24 | -0.269 | Downregulated | 1.68E-02 | 7.35E-02 |
| MAEA | -0.269 | Downregulated | 2.94E-06 | 1.00E-04 |
| KRT72 | -0.269 | Downregulated | 3.57E-02 | 1.29E-01 |
| TRIM32 | -0.269 | Downregulated | 4.68E-05 | 7.83E-04 |
| TCL6 | -0.269 | Downregulated | 5.12E-04 | 4.90E-03 |

| | | | | |
|---|---|---|---|---|
| CASP2 | -0.269 | Downregulated | 2.22E-11 | 2.40E-08 |
| GORASP2 | -0.269 | Downregulated | 3.45E-08 | 4.17E-06 |
| GPA33 | -0.268 | Downregulated | 7.54E-03 | 3.98E-02 |
| ATG9B | -0.268 | Downregulated | 8.50E-06 | 2.13E-04 |
| ESYT1 | -0.268 | Downregulated | 1.33E-04 | 1.76E-03 |
| CRYGS | -0.268 | Downregulated | 2.90E-07 | 1.95E-05 |
| PUS1 | -0.268 | Downregulated | 6.09E-06 | 1.64E-04 |
| PNKD | -0.268 | Downregulated | 2.75E-05 | 5.20E-04 |
| MED6 | -0.268 | Downregulated | 2.38E-09 | 7.37E-07 |
| ZNF416 | -0.268 | Downregulated | 3.99E-05 | 6.96E-04 |
| CCDC109B | -0.268 | Downregulated | 1.16E-04 | 1.59E-03 |
| TOMM5 | -0.267 | Downregulated | 2.89E-02 | 1.11E-01 |
| WDR34 | -0.267 | Downregulated | 1.23E-04 | 1.66E-03 |
| IL7R | -0.267 | Downregulated | 2.71E-02 | 1.06E-01 |
| HNRNPM | -0.267 | Downregulated | 6.89E-08 | 6.88E-06 |
| MKI67IP | -0.267 | Downregulated | 2.89E-03 | 1.89E-02 |
| ZNF79 | -0.267 | Downregulated | 9.06E-07 | 4.34E-05 |
| RPL12 | -0.267 | Downregulated | 2.37E-04 | 2.73E-03 |
| ATP5A1 | -0.267 | Downregulated | 6.52E-08 | 6.70E-06 |
| CXCR5 | -0.267 | Downregulated | 1.21E-02 | 5.74E-02 |
| NCAPD3 | -0.267 | Downregulated | 1.19E-06 | 5.27E-05 |
| OLIG2 | -0.267 | Downregulated | 4.60E-02 | 1.55E-01 |
| ZNF263 | -0.266 | Downregulated | 6.39E-04 | 5.81E-03 |
| WFS1 | -0.266 | Downregulated | 2.18E-04 | 2.57E-03 |
| ABHD10 | -0.266 | Downregulated | 4.85E-06 | 1.41E-04 |
| MTBP | -0.266 | Downregulated | 3.64E-06 | 1.14E-04 |
| TRAPPC6A | -0.266 | Downregulated | 6.16E-05 | 9.73E-04 |
| GNG7 | -0.266 | Downregulated | 5.41E-03 | 3.07E-02 |
| FAM38A | -0.266 | Downregulated | 2.10E-05 | 4.24E-04 |
| PPP1R8 | -0.266 | Downregulated | 6.38E-06 | 1.70E-04 |
| FXYD5 | -0.265 | Downregulated | 4.78E-06 | 1.39E-04 |
| ZNF594 | -0.265 | Downregulated | 8.05E-04 | 6.94E-03 |
| DHRSX | 0.265 | Upregulated | 2.79E-05 | 5.26E-04 |
| HDGF | 0.266 | Upregulated | 2.78E-03 | 1.83E-02 |
| PCDHB9 | 0.266 | Upregulated | 1.79E-04 | 2.20E-03 |
| NFKB2 | 0.266 | Upregulated | 4.48E-04 | 4.44E-03 |
| CEBPD | 0.266 | Upregulated | 6.10E-03 | 3.37E-02 |
| GFI1B | 0.267 | Upregulated | 1.31E-03 | 1.02E-02 |
| SAMD4A | 0.267 | Upregulated | 1.24E-02 | 5.85E-02 |
| THBS3 | 0.267 | Upregulated | 5.61E-06 | 1.55E-04 |
| RRBP1 | 0.267 | Upregulated | 6.66E-04 | 5.99E-03 |
| FAM176B | 0.267 | Upregulated | 1.14E-04 | 1.57E-03 |
| RNF123 | 0.267 | Upregulated | 5.67E-04 | 5.30E-03 |
| KIF1B | 0.267 | Upregulated | 8.02E-03 | 4.17E-02 |
| RTP4 | 0.267 | Upregulated | 2.70E-02 | 1.05E-01 |
| BAT3 | 0.268 | Upregulated | 2.69E-04 | 3.00E-03 |
| XRN1 | 0.268 | Upregulated | 9.44E-05 | 1.35E-03 |
| TRIM10 | 0.268 | Upregulated | 7.71E-03 | 4.04E-02 |
| LRP10 | 0.269 | Upregulated | 7.11E-05 | 1.09E-03 |
| ACTN1 | 0.269 | Upregulated | 2.22E-03 | 1.54E-02 |
| CMPK1 | 0.269 | Upregulated | 1.68E-03 | 1.23E-02 |

| | | | | |
|---|---|---|---|---|
| ZNF281 | 0.269 | Upregulated | 3.91E-04 | 4.00E-03 |
| CMIP | 0.269 | Upregulated | 6.49E-04 | 5.87E-03 |
| HCN4 | 0.269 | Upregulated | 2.31E-06 | 8.36E-05 |
| FOSL2 | 0.270 | Upregulated | 8.70E-05 | 1.27E-03 |
| C1RL | 0.270 | Upregulated | 4.35E-03 | 2.60E-02 |
| AP2A1 | 0.270 | Upregulated | 9.99E-03 | 4.95E-02 |
| NDUFB9 | 0.270 | Upregulated | 7.35E-04 | 6.48E-03 |
| SPTA1 | 0.270 | Upregulated | 4.40E-03 | 2.62E-02 |
| PRIM2 | 0.270 | Upregulated | 2.40E-03 | 1.63E-02 |
| SIRPA | 0.271 | Upregulated | 1.82E-02 | 7.81E-02 |
| CYBB | 0.271 | Upregulated | 2.89E-04 | 3.15E-03 |
| SMNDC1 | 0.271 | Upregulated | 2.09E-03 | 1.47E-02 |
| KCNMB1 | 0.271 | Upregulated | 9.53E-04 | 7.95E-03 |
| FAM76A | 0.271 | Upregulated | 1.16E-05 | 2.70E-04 |
| B4GALT5 | 0.271 | Upregulated | 6.28E-03 | 3.45E-02 |
| TTC7B | 0.271 | Upregulated | 1.04E-04 | 1.46E-03 |
| ELL | 0.272 | Upregulated | 3.91E-06 | 1.20E-04 |
| FAH | 0.272 | Upregulated | 3.94E-03 | 2.41E-02 |
| FLJ45337 | 0.272 | Upregulated | 1.33E-04 | 1.76E-03 |
| MT1G | 0.272 | Upregulated | 2.29E-02 | 9.29E-02 |
| ARG2 | 0.272 | Upregulated | 3.20E-06 | 1.05E-04 |
| 02-Mar | 0.272 | Upregulated | 1.20E-03 | 9.53E-03 |
| DRAM1 | 0.272 | Upregulated | 3.88E-03 | 2.38E-02 |
| VISA | 0.272 | Upregulated | 6.82E-06 | 1.79E-04 |
| ISCA1 | 0.273 | Upregulated | 2.69E-03 | 1.78E-02 |
| YY1 | 0.273 | Upregulated | 1.81E-05 | 3.79E-04 |
| RAPGEFL1 | 0.273 | Upregulated | 2.31E-05 | 4.56E-04 |
| BAGE2 | 0.274 | Upregulated | 1.31E-05 | 2.97E-04 |
| EPAS1 | 0.274 | Upregulated | 6.26E-03 | 3.44E-02 |
| ACOX1 | 0.274 | Upregulated | 9.26E-04 | 7.76E-03 |
| YPEL3 | 0.274 | Upregulated | 4.79E-03 | 2.80E-02 |
| TNIP1 | 0.274 | Upregulated | 7.77E-05 | 1.17E-03 |
| GPRIN3 | 0.274 | Upregulated | 1.46E-02 | 6.62E-02 |
| SLC22A16 | 0.274 | Upregulated | 2.30E-02 | 9.33E-02 |
| FAM21C | 0.275 | Upregulated | 4.81E-09 | 1.17E-06 |
| FLJ41603 | 0.275 | Upregulated | 4.64E-05 | 7.79E-04 |
| FAM63A | 0.275 | Upregulated | 6.74E-08 | 6.80E-06 |
| EIF1AX | 0.276 | Upregulated | 9.56E-07 | 4.48E-05 |
| HERPUD2 | 0.276 | Upregulated | 3.81E-07 | 2.36E-05 |
| IRAK3 | 0.276 | Upregulated | 2.00E-02 | 8.38E-02 |
| LGALS8 | 0.276 | Upregulated | 1.09E-06 | 4.93E-05 |
| SAR1A | 0.277 | Upregulated | 1.59E-04 | 2.02E-03 |
| GRAP2 | 0.277 | Upregulated | 1.70E-04 | 2.12E-03 |
| PNPLA8 | 0.277 | Upregulated | 7.14E-04 | 6.34E-03 |
| WDR26 | 0.277 | Upregulated | 1.20E-04 | 1.63E-03 |
| CCNDBP1 | 0.277 | Upregulated | 1.82E-03 | 1.31E-02 |
| HK3 | 0.277 | Upregulated | 2.59E-02 | 1.02E-01 |
| NBN | 0.277 | Upregulated | 5.35E-04 | 5.07E-03 |
| RPH3A | 0.277 | Upregulated | 1.38E-02 | 6.34E-02 |
| CAV2 | 0.278 | Upregulated | 1.00E-03 | 8.26E-03 |
| SP100 | 0.278 | Upregulated | 2.28E-03 | 1.57E-02 |

| | | | | |
|---|---|---|---|---|
| PPP4R2 | 0.278 | Upregulated | 1.15E-05 | 2.70E-04 |
| WRB | 0.278 | Upregulated | 1.01E-04 | 1.43E-03 |
| ZNF777 | 0.278 | Upregulated | 2.91E-06 | 9.93E-05 |
| ATG2A | 0.278 | Upregulated | 6.09E-07 | 3.24E-05 |
| QRICH1 | 0.279 | Upregulated | 1.47E-04 | 1.89E-03 |
| PNPT1 | 0.279 | Upregulated | 3.26E-03 | 2.08E-02 |
| CUTL1 | 0.279 | Upregulated | 5.22E-05 | 8.53E-04 |
| DUSP1 | 0.280 | Upregulated | 1.44E-02 | 6.55E-02 |
| TDRD1 | 0.280 | Upregulated | 9.72E-04 | 8.07E-03 |
| PIK3CG | 0.280 | Upregulated | 3.46E-06 | 1.11E-04 |
| WDR51B | 0.280 | Upregulated | 4.09E-04 | 4.14E-03 |
| CXCL14 | 0.280 | Upregulated | 8.91E-05 | 1.30E-03 |
| CHSY1 | 0.280 | Upregulated | 1.14E-04 | 1.57E-03 |
| TRIM24 | 0.280 | Upregulated | 2.76E-09 | 8.20E-07 |
| IP6K1 | 0.281 | Upregulated | 3.36E-06 | 1.09E-04 |
| ATF4 | 0.282 | Upregulated | 1.40E-04 | 1.83E-03 |
| GLUL | 0.282 | Upregulated | 4.43E-04 | 4.40E-03 |
| SF3B1 | 0.282 | Upregulated | 9.48E-04 | 7.92E-03 |
| DMXL1 | 0.282 | Upregulated | 1.27E-03 | 9.93E-03 |
| PILRA | 0.282 | Upregulated | 7.49E-03 | 3.96E-02 |
| TMEM154 | 0.283 | Upregulated | 1.53E-04 | 1.95E-03 |
| MKNK1 | 0.283 | Upregulated | 7.79E-04 | 6.78E-03 |
| GPBP1L1 | 0.283 | Upregulated | 2.27E-07 | 1.65E-05 |
| GUCY1B3 | 0.284 | Upregulated | 4.33E-05 | 7.41E-04 |
| FAM83F | 0.284 | Upregulated | 2.70E-02 | 1.05E-01 |
| FKBP8 | 0.284 | Upregulated | 3.08E-02 | 1.16E-01 |
| ATF1 | 0.284 | Upregulated | 2.35E-04 | 2.71E-03 |
| HEBP2 | 0.284 | Upregulated | 1.66E-03 | 1.23E-02 |
| MMP25 | 0.285 | Upregulated | 7.12E-03 | 3.80E-02 |
| A2M | 0.285 | Upregulated | 1.49E-04 | 1.92E-03 |
| UGCG | 0.285 | Upregulated | 1.07E-04 | 1.49E-03 |
| HSD17B11 | 0.285 | Upregulated | 9.54E-04 | 7.96E-03 |
| HSN2 | 0.285 | Upregulated | 4.21E-09 | 1.06E-06 |
| ELOVL7 | 0.285 | Upregulated | 1.80E-05 | 3.76E-04 |
| TRPM2 | 0.285 | Upregulated | 4.82E-07 | 2.77E-05 |
| USH1G | 0.286 | Upregulated | 3.10E-04 | 3.34E-03 |
| JUNB | 0.286 | Upregulated | 2.50E-03 | 1.68E-02 |
| KIAA0408 | 0.286 | Upregulated | 5.76E-04 | 5.37E-03 |
| SNAP23 | 0.286 | Upregulated | 3.34E-04 | 3.54E-03 |
| HIST1H4H | 0.286 | Upregulated | 3.13E-02 | 1.18E-01 |
| MAP1LC3B2 | 0.287 | Upregulated | 1.66E-03 | 1.22E-02 |
| PPP1R15B | 0.287 | Upregulated | 5.13E-06 | 1.47E-04 |
| TUBA3D | 0.287 | Upregulated | 7.31E-08 | 7.26E-06 |
| IQGAP1 | 0.287 | Upregulated | 7.56E-04 | 6.62E-03 |
| ROCK1 | 0.287 | Upregulated | 9.00E-04 | 7.59E-03 |
| CETP | 0.288 | Upregulated | 8.46E-04 | 7.23E-03 |
| ARFGEF1 | 0.288 | Upregulated | 2.18E-04 | 2.56E-03 |
| BOK | 0.288 | Upregulated | 5.74E-05 | 9.20E-04 |
| TRIM9 | 0.289 | Upregulated | 1.61E-03 | 1.20E-02 |
| FLJ42957 | 0.289 | Upregulated | 3.75E-03 | 2.32E-02 |
| HSPA4 | 0.289 | Upregulated | 1.58E-03 | 1.17E-02 |

| | | | | |
|---|---|---|---|---|
| EIF4G3 | 0.289 | Upregulated | 6.90E-08 | 6.88E-06 |
| STAB1 | 0.289 | Upregulated | 4.09E-03 | 2.48E-02 |
| GABARAPL2 | 0.290 | Upregulated | 4.80E-05 | 8.00E-04 |
| TMEM8 | 0.290 | Upregulated | 3.09E-04 | 3.33E-03 |
| CEP27 | 0.290 | Upregulated | 6.57E-03 | 3.58E-02 |
| UHMK1 | 0.290 | Upregulated | 2.71E-04 | 3.01E-03 |
| SFRS12 | 0.290 | Upregulated | 1.59E-04 | 2.01E-03 |
| FLJ22662 | 0.291 | Upregulated | 2.05E-02 | 8.55E-02 |
| LYZ | 0.291 | Upregulated | 3.73E-04 | 3.86E-03 |
| FGFR1OP2 | 0.292 | Upregulated | 6.35E-05 | 9.94E-04 |
| TDRD9 | 0.292 | Upregulated | 3.93E-02 | 1.38E-01 |
| TRPM6 | 0.292 | Upregulated | 1.24E-02 | 5.84E-02 |
| PDK1 | 0.293 | Upregulated | 4.40E-05 | 7.50E-04 |
| STOM | 0.293 | Upregulated | 4.26E-04 | 4.28E-03 |
| NR1D1 | 0.294 | Upregulated | 3.08E-03 | 1.99E-02 |
| TIFA | 0.294 | Upregulated | 1.63E-03 | 1.21E-02 |
| MAP1A | 0.294 | Upregulated | 8.38E-03 | 4.32E-02 |
| CAT | 0.295 | Upregulated | 6.78E-04 | 6.08E-03 |
| HCG27 | 0.295 | Upregulated | 1.86E-03 | 1.34E-02 |
| GAS2L1 | 0.295 | Upregulated | 4.82E-04 | 4.69E-03 |
| HIST1H4E | 0.295 | Upregulated | 3.17E-04 | 3.39E-03 |
| RNF10 | 0.295 | Upregulated | 2.13E-02 | 8.81E-02 |
| AGTRAP | 0.295 | Upregulated | 3.39E-03 | 2.14E-02 |
| OR51S1 | 0.296 | Upregulated | 9.56E-05 | 1.36E-03 |
| CDC34 | 0.296 | Upregulated | 2.04E-02 | 8.52E-02 |
| TPM4 | 0.297 | Upregulated | 2.00E-04 | 2.39E-03 |
| GALM | 0.297 | Upregulated | 3.11E-03 | 2.00E-02 |
| RNF13 | 0.297 | Upregulated | 1.02E-05 | 2.46E-04 |
| TRIM38 | 0.297 | Upregulated | 3.50E-06 | 1.12E-04 |
| ATXN1 | 0.297 | Upregulated | 9.54E-07 | 4.48E-05 |
| PLEKHO2 | 0.297 | Upregulated | 1.23E-03 | 9.69E-03 |
| RNPC3 | 0.297 | Upregulated | 2.15E-07 | 1.60E-05 |
| UBE2B | 0.298 | Upregulated | 3.15E-05 | 5.80E-04 |
| NDST1 | 0.298 | Upregulated | 3.35E-05 | 6.07E-04 |
| GPR42 | 0.298 | Upregulated | 5.72E-03 | 3.20E-02 |
| TMEM140 | 0.298 | Upregulated | 6.79E-03 | 3.67E-02 |
| ETV6 | 0.298 | Upregulated | 1.93E-06 | 7.29E-05 |
| RNF19B | 0.298 | Upregulated | 7.38E-04 | 6.50E-03 |
| SDSL | 0.298 | Upregulated | 2.45E-03 | 1.66E-02 |
| HBQ1 | 0.298 | Upregulated | 4.48E-02 | 1.52E-01 |
| ZRANB1 | 0.298 | Upregulated | 4.15E-04 | 4.19E-03 |
| NCF1 | 0.298 | Upregulated | 1.45E-03 | 1.10E-02 |
| PTCRA | 0.299 | Upregulated | 1.07E-03 | 8.67E-03 |
| WWC3 | 0.299 | Upregulated | 8.48E-07 | 4.15E-05 |
| NCF4 | 0.299 | Upregulated | 4.78E-03 | 2.79E-02 |
| MBD4 | 0.300 | Upregulated | 2.18E-03 | 1.51E-02 |
| MYO1F | 0.300 | Upregulated | 3.78E-03 | 2.33E-02 |
| RHCE | 0.301 | Upregulated | 1.03E-03 | 8.44E-03 |
| PYGL | 0.301 | Upregulated | 1.74E-02 | 7.56E-02 |
| CCNT2 | 0.301 | Upregulated | 2.80E-04 | 3.08E-03 |
| ZC3H11A | 0.301 | Upregulated | 4.39E-06 | 1.30E-04 |

| | | | | |
|---|---|---|---|---|
| HSPA6 | 0.301 | Upregulated | 1.70E-03 | 1.25E-02 |
| RBMS1 | 0.301 | Upregulated | 5.86E-04 | 5.43E-03 |
| KIAA1618 | 0.301 | Upregulated | 1.25E-04 | 1.68E-03 |
| RERE | 0.302 | Upregulated | 2.47E-05 | 4.78E-04 |
| GALNT3 | 0.302 | Upregulated | 6.63E-04 | 5.96E-03 |
| MARK2 | 0.302 | Upregulated | 9.80E-05 | 1.39E-03 |
| THBS1 | 0.302 | Upregulated | 1.61E-02 | 7.11E-02 |
| TAP1 | 0.302 | Upregulated | 9.03E-04 | 7.61E-03 |
| BAGE3 | 0.302 | Upregulated | 1.74E-03 | 1.27E-02 |
| LCOR | 0.302 | Upregulated | 7.26E-06 | 1.88E-04 |
| SP110 | 0.303 | Upregulated | 7.38E-06 | 1.91E-04 |
| PNPLA2 | 0.303 | Upregulated | 7.50E-03 | 3.96E-02 |
| MOBKL1A | 0.303 | Upregulated | 1.80E-06 | 6.95E-05 |
| CD300A | 0.303 | Upregulated | 5.42E-04 | 5.12E-03 |
| DENND1A | 0.303 | Upregulated | 3.61E-05 | 6.45E-04 |
| FNDC3B | 0.304 | Upregulated | 5.91E-05 | 9.43E-04 |
| OSBPL2 | 0.304 | Upregulated | 1.76E-05 | 3.70E-04 |
| DNAJC25-GNG10 | 0.304 | Upregulated | 5.85E-06 | 1.59E-04 |
| WNK1 | 0.304 | Upregulated | 4.43E-04 | 4.40E-03 |
| RAP1B | 0.305 | Upregulated | 1.55E-04 | 1.97E-03 |
| EMR2 | 0.305 | Upregulated | 1.40E-02 | 6.43E-02 |
| OSBPL11 | 0.305 | Upregulated | 1.43E-03 | 1.09E-02 |
| IFI30 | 0.306 | Upregulated | 1.29E-03 | 1.00E-02 |
| GP1BA | 0.306 | Upregulated | 2.45E-05 | 4.76E-04 |
| ADAM8 | 0.306 | Upregulated | 4.31E-04 | 4.31E-03 |
| SPIN1 | 0.306 | Upregulated | 1.50E-04 | 1.92E-03 |
| GLDN | 0.306 | Upregulated | 6.87E-03 | 3.70E-02 |
| GPR146 | 0.307 | Upregulated | 1.28E-02 | 6.01E-02 |
| ZNF185 | 0.307 | Upregulated | 1.85E-03 | 1.33E-02 |
| SLC25A37 | 0.307 | Upregulated | 2.18E-02 | 8.96E-02 |
| LCP2 | 0.307 | Upregulated | 1.36E-07 | 1.13E-05 |
| IRX3 | 0.307 | Upregulated | 1.79E-02 | 7.70E-02 |
| TRIML2 | 0.307 | Upregulated | 3.40E-06 | 1.10E-04 |
| CD36 | 0.307 | Upregulated | 3.81E-03 | 2.34E-02 |
| BCL2L1 | 0.307 | Upregulated | 4.67E-02 | 1.57E-01 |
| CLEC4E | 0.307 | Upregulated | 7.30E-03 | 3.88E-02 |
| ASPRV1 | 0.307 | Upregulated | 8.96E-03 | 4.55E-02 |
| TAF4B | 0.307 | Upregulated | 1.95E-04 | 2.34E-03 |
| GCA | 0.307 | Upregulated | 3.76E-03 | 2.32E-02 |
| TMSL3 | 0.308 | Upregulated | 1.03E-05 | 2.48E-04 |
| CREG1 | 0.308 | Upregulated | 2.32E-03 | 1.59E-02 |
| PTAFR | 0.308 | Upregulated | 7.17E-03 | 3.82E-02 |
| SYAP1 | 0.308 | Upregulated | 9.53E-06 | 2.34E-04 |
| SLC9A8 | 0.308 | Upregulated | 8.22E-05 | 1.22E-03 |
| NOTCH2NL | 0.309 | Upregulated | 3.06E-06 | 1.03E-04 |
| SLC25A44 | 0.309 | Upregulated | 4.98E-05 | 8.25E-04 |
| SESTD1 | 0.310 | Upregulated | 1.40E-06 | 5.86E-05 |
| FAM20A | 0.310 | Upregulated | 5.16E-03 | 2.96E-02 |
| SIRPB1 | 0.311 | Upregulated | 6.11E-03 | 3.37E-02 |
| LY6G6D | 0.311 | Upregulated | 2.15E-03 | 1.50E-02 |
| TOPORS | 0.311 | Upregulated | 7.30E-07 | 3.71E-05 |

| | | | | |
|---|---|---|---|---|
| TSN | 0.312 | Upregulated | 1.54E-05 | 3.35E-04 |
| HES6 | 0.313 | Upregulated | 2.78E-02 | 1.08E-01 |
| IGFBP1 | 0.313 | Upregulated | 4.34E-05 | 7.41E-04 |
| CTR9 | 0.313 | Upregulated | 1.36E-05 | 3.06E-04 |
| PLIN5 | 0.314 | Upregulated | 9.79E-04 | 8.11E-03 |
| SLC38A5 | 0.314 | Upregulated | 2.70E-03 | 1.79E-02 |
| RPN2 | 0.314 | Upregulated | 2.91E-03 | 1.90E-02 |
| SLAMF7 | 0.314 | Upregulated | 3.89E-07 | 2.39E-05 |
| HIST2H2AC | 0.315 | Upregulated | 4.17E-03 | 2.51E-02 |
| RNF130 | 0.315 | Upregulated | 1.34E-04 | 1.76E-03 |
| ZCCHC2 | 0.316 | Upregulated | 4.17E-04 | 4.20E-03 |
| RNF19A | 0.316 | Upregulated | 2.50E-03 | 1.68E-02 |
| LOXL3 | 0.316 | Upregulated | 1.46E-06 | 6.04E-05 |
| LPCAT2 | 0.316 | Upregulated | 2.01E-02 | 8.41E-02 |
| ARHGAP26 | 0.317 | Upregulated | 1.09E-03 | 8.82E-03 |
| TMEM49 | 0.317 | Upregulated | 5.85E-05 | 9.34E-04 |
| TIMP2 | 0.317 | Upregulated | 7.23E-03 | 3.85E-02 |
| HOXC10 | 0.317 | Upregulated | 2.22E-03 | 1.54E-02 |
| CORO1C | 0.317 | Upregulated | 5.86E-06 | 1.59E-04 |
| KLF5 | 0.318 | Upregulated | 8.48E-08 | 8.17E-06 |
| TGM2 | 0.318 | Upregulated | 8.30E-03 | 4.29E-02 |
| REM2 | 0.318 | Upregulated | 1.49E-03 | 1.13E-02 |
| PARP9 | 0.318 | Upregulated | 5.28E-04 | 5.02E-03 |
| BCL3 | 0.319 | Upregulated | 2.09E-03 | 1.47E-02 |
| ACTA2 | 0.320 | Upregulated | 1.11E-03 | 8.96E-03 |
| ST6GALNAC2 | 0.320 | Upregulated | 3.21E-03 | 2.06E-02 |
| TNFSF13B | 0.320 | Upregulated | 2.65E-03 | 1.76E-02 |
| BMX | 0.320 | Upregulated | 1.21E-04 | 1.64E-03 |
| LBA1 | 0.320 | Upregulated | 1.08E-04 | 1.50E-03 |
| HMGB1 | 0.320 | Upregulated | 1.15E-04 | 1.58E-03 |
| TNFSF10 | 0.320 | Upregulated | 2.35E-03 | 1.60E-02 |
| GUCY1A3 | 0.320 | Upregulated | 4.66E-05 | 7.81E-04 |
| GIMAP1 | 0.321 | Upregulated | 1.40E-03 | 1.07E-02 |
| CHD1 | 0.321 | Upregulated | 2.85E-04 | 3.12E-03 |
| CTRL | 0.321 | Upregulated | 3.64E-07 | 2.27E-05 |
| ZNF600 | 0.322 | Upregulated | 1.03E-03 | 8.45E-03 |
| CFB | 0.322 | Upregulated | 1.12E-08 | 2.07E-06 |
| NFIA | 0.322 | Upregulated | 2.16E-04 | 2.55E-03 |
| CENTD2 | 0.322 | Upregulated | 1.48E-08 | 2.50E-06 |
| NAMPT | 0.322 | Upregulated | 2.52E-02 | 9.98E-02 |
| CBX3 | 0.322 | Upregulated | 1.24E-04 | 1.67E-03 |
| GCLC | 0.322 | Upregulated | 4.39E-06 | 1.30E-04 |
| MICAL2 | 0.322 | Upregulated | 3.97E-03 | 2.42E-02 |
| MBOAT2 | 0.323 | Upregulated | 2.33E-06 | 8.40E-05 |
| RANGRF | 0.323 | Upregulated | 3.29E-04 | 3.49E-03 |
| CPD | 0.323 | Upregulated | 1.45E-03 | 1.10E-02 |
| ZBTB2 | 0.323 | Upregulated | 1.14E-05 | 2.69E-04 |
| MPP1 | 0.323 | Upregulated | 1.33E-02 | 6.16E-02 |
| RHBDF2 | 0.323 | Upregulated | 3.21E-09 | 8.99E-07 |
| YIPF3 | 0.324 | Upregulated | 7.11E-09 | 1.54E-06 |
| CD163 | 0.324 | Upregulated | 1.65E-02 | 7.25E-02 |

| | | | | |
|---|---|---|---|---|
| NADK | 0.325 | Upregulated | 4.08E-04 | 4.13E-03 |
| CCL4L1 | 0.325 | Upregulated | 1.62E-02 | 7.15E-02 |
| RHBDL2 | 0.326 | Upregulated | 2.54E-04 | 2.87E-03 |
| RBM7 | 0.326 | Upregulated | 2.71E-04 | 3.01E-03 |
| TMEM88 | 0.327 | Upregulated | 1.97E-03 | 1.40E-02 |
| OPLAH | 0.328 | Upregulated | 1.39E-02 | 6.37E-02 |
| SUCNR1 | 0.328 | Upregulated | 1.44E-05 | 3.17E-04 |
| ECGF1 | 0.328 | Upregulated | 5.26E-04 | 5.01E-03 |
| GPX8 | 0.328 | Upregulated | 4.71E-04 | 4.61E-03 |
| RHOB | 0.328 | Upregulated | 6.55E-04 | 5.92E-03 |
| DTX3L | 0.329 | Upregulated | 1.56E-03 | 1.17E-02 |
| FLJ45445 | 0.329 | Upregulated | 1.45E-03 | 1.10E-02 |
| RPL9 | 0.330 | Upregulated | 2.82E-02 | 1.09E-01 |
| S100A8 | 0.330 | Upregulated | 8.55E-03 | 4.38E-02 |
| TUBB1 | 0.330 | Upregulated | 9.79E-03 | 4.87E-02 |
| CLEC4D | 0.330 | Upregulated | 5.22E-03 | 2.99E-02 |
| NT5M | 0.330 | Upregulated | 1.54E-03 | 1.15E-02 |
| FGL2 | 0.330 | Upregulated | 2.98E-04 | 3.23E-03 |
| PAK2 | 0.330 | Upregulated | 4.02E-02 | 1.41E-01 |
| ROPN1L | 0.330 | Upregulated | 4.44E-03 | 2.63E-02 |
| GAA | 0.331 | Upregulated | 4.29E-04 | 4.30E-03 |
| CTSO | 0.331 | Upregulated | 8.08E-06 | 2.05E-04 |
| ANKDD1A | 0.331 | Upregulated | 5.75E-03 | 3.21E-02 |
| DYNC1LI1 | 0.332 | Upregulated | 4.21E-06 | 1.27E-04 |
| ALDH5A1 | 0.332 | Upregulated | 1.08E-03 | 8.77E-03 |
| UBA6 | 0.333 | Upregulated | 2.34E-04 | 2.70E-03 |
| EXOC8 | 0.333 | Upregulated | 3.16E-05 | 5.80E-04 |
| PPP2R5B | 0.333 | Upregulated | 4.82E-04 | 4.69E-03 |
| MSRB2 | 0.334 | Upregulated | 2.59E-05 | 4.97E-04 |
| SP3 | 0.334 | Upregulated | 2.51E-06 | 8.91E-05 |
| HS2ST1 | 0.334 | Upregulated | 3.17E-06 | 1.05E-04 |
| CREBBP | 0.334 | Upregulated | 6.68E-06 | 1.76E-04 |
| FCGR3A | 0.334 | Upregulated | 7.51E-04 | 6.58E-03 |
| CARS2 | 0.335 | Upregulated | 1.29E-08 | 2.28E-06 |
| TMEM180 | 0.335 | Upregulated | 5.05E-06 | 1.45E-04 |
| RBM47 | 0.336 | Upregulated | 1.15E-03 | 9.21E-03 |
| EVI5 | 0.336 | Upregulated | 1.22E-03 | 9.60E-03 |
| PPM2C | 0.336 | Upregulated | 5.97E-05 | 9.50E-04 |
| TREM1 | 0.337 | Upregulated | 4.05E-03 | 2.46E-02 |
| ATP11B | 0.337 | Upregulated | 2.95E-05 | 5.52E-04 |
| CARS | 0.337 | Upregulated | 1.32E-11 | 1.64E-08 |
| PHLDB1 | 0.338 | Upregulated | 2.85E-03 | 1.87E-02 |
| MAGT1 | 0.338 | Upregulated | 1.39E-05 | 3.12E-04 |
| NMI | 0.339 | Upregulated | 1.38E-06 | 5.80E-05 |
| DDEF2 | 0.339 | Upregulated | 1.88E-06 | 7.15E-05 |
| FBXO6 | 0.339 | Upregulated | 7.46E-04 | 6.55E-03 |
| FLJ20489 | 0.340 | Upregulated | 9.16E-03 | 4.63E-02 |
| WDR23 | 0.340 | Upregulated | 1.47E-05 | 3.23E-04 |
| GBP2 | 0.340 | Upregulated | 4.38E-07 | 2.62E-05 |
| CD63 | 0.340 | Upregulated | 7.96E-05 | 1.19E-03 |
| DCTN4 | 0.340 | Upregulated | 1.49E-06 | 6.12E-05 |

| | | | | |
|---|---|---|---|---|
| ASGR2 | 0.342 | Upregulated | 4.57E-03 | 2.69E-02 |
| SECTM1 | 0.342 | Upregulated | 8.00E-06 | 2.03E-04 |
| CA2 | 0.342 | Upregulated | 7.67E-03 | 4.03E-02 |
| CALCOCO2 | 0.343 | Upregulated | 4.62E-10 | 2.46E-07 |
| HIST2H2AA4 | 0.343 | Upregulated | 2.62E-03 | 1.75E-02 |
| PANX2 | 0.343 | Upregulated | 1.18E-02 | 5.65E-02 |
| TLR5 | 0.343 | Upregulated | 3.65E-03 | 2.27E-02 |
| PPM1B | 0.344 | Upregulated | 3.57E-06 | 1.13E-04 |
| ALOX12 | 0.344 | Upregulated | 9.38E-03 | 4.71E-02 |
| CLEC5A | 0.344 | Upregulated | 1.99E-02 | 8.35E-02 |
| PRRG4 | 0.345 | Upregulated | 5.34E-07 | 2.94E-05 |
| UBE4A | 0.345 | Upregulated | 3.43E-05 | 6.17E-04 |
| SACM1L | 0.345 | Upregulated | 2.19E-04 | 2.57E-03 |
| GABARAP | 0.345 | Upregulated | 1.22E-07 | 1.04E-05 |
| SERINC3 | 0.345 | Upregulated | 2.68E-06 | 9.39E-05 |
| PTPN2 | 0.346 | Upregulated | 2.35E-06 | 8.44E-05 |
| ABCC4 | 0.346 | Upregulated | 2.16E-04 | 2.55E-03 |
| KBTBD7 | 0.346 | Upregulated | 5.54E-04 | 5.20E-03 |
| TIPRL | 0.346 | Upregulated | 4.13E-05 | 7.13E-04 |
| TLR4 | 0.347 | Upregulated | 1.64E-03 | 1.21E-02 |
| RELB | 0.347 | Upregulated | 7.15E-05 | 1.09E-03 |
| CLEC7A | 0.347 | Upregulated | 3.53E-03 | 2.21E-02 |
| PPP1R12A | 0.347 | Upregulated | 4.77E-04 | 4.65E-03 |
| ITPRIP | 0.348 | Upregulated | 3.70E-06 | 1.16E-04 |
| TRIP12 | 0.348 | Upregulated | 1.02E-05 | 2.46E-04 |
| ARHGAP18 | 0.348 | Upregulated | 3.09E-07 | 2.05E-05 |
| JAK2 | 0.348 | Upregulated | 1.94E-04 | 2.34E-03 |
| FLJ27255 | 0.348 | Upregulated | 5.48E-06 | 1.53E-04 |
| CLEC1B | 0.348 | Upregulated | 1.92E-02 | 8.12E-02 |
| ESPN | 0.348 | Upregulated | 3.12E-02 | 1.17E-01 |
| RTF1 | 0.349 | Upregulated | 1.04E-03 | 8.52E-03 |
| PTMS | 0.349 | Upregulated | 7.02E-03 | 3.76E-02 |
| IL4R | 0.349 | Upregulated | 1.75E-04 | 2.16E-03 |
| ST3GAL4 | 0.350 | Upregulated | 1.67E-03 | 1.23E-02 |
| SFMBT2 | 0.350 | Upregulated | 1.92E-03 | 1.37E-02 |
| HCK | 0.350 | Upregulated | 5.07E-04 | 4.86E-03 |
| SCGB1C1 | 0.350 | Upregulated | 1.04E-03 | 8.49E-03 |
| UBE1C | 0.350 | Upregulated | 7.91E-07 | 3.95E-05 |
| STX11 | 0.351 | Upregulated | 3.80E-04 | 3.91E-03 |
| MXI1 | 0.351 | Upregulated | 1.95E-02 | 8.23E-02 |
| CTRC | 0.352 | Upregulated | 8.09E-06 | 2.05E-04 |
| GHRL | 0.352 | Upregulated | 4.11E-03 | 2.48E-02 |
| SPOCD1 | 0.352 | Upregulated | 1.05E-02 | 5.14E-02 |
| TMEM106A | 0.352 | Upregulated | 9.73E-03 | 4.85E-02 |
| USP8 | 0.352 | Upregulated | 3.86E-06 | 1.19E-04 |
| RFWD2 | 0.353 | Upregulated | 2.50E-06 | 8.87E-05 |
| FPR2 | 0.353 | Upregulated | 9.97E-03 | 4.94E-02 |
| SLC6A6 | 0.353 | Upregulated | 5.17E-04 | 4.94E-03 |
| AFTPH | 0.354 | Upregulated | 1.96E-06 | 7.36E-05 |
| PTGS1 | 0.354 | Upregulated | 4.44E-05 | 7.55E-04 |
| OAS3 | 0.354 | Upregulated | 9.86E-03 | 4.90E-02 |

| | | | | |
|---|---|---|---|---|
| KIAA0367 | 0.355 | Upregulated | 6.16E-03 | 3.39E-02 |
| GDPD5 | 0.355 | Upregulated | 9.16E-05 | 1.32E-03 |
| DHRS12 | 0.355 | Upregulated | 7.13E-05 | 1.09E-03 |
| PTGR2 | 0.355 | Upregulated | 1.80E-04 | 2.21E-03 |
| ADIPOR1 | 0.355 | Upregulated | 1.25E-02 | 5.87E-02 |
| PBX1 | 0.356 | Upregulated | 3.86E-03 | 2.37E-02 |
| TRIM56 | 0.356 | Upregulated | 9.97E-08 | 9.22E-06 |
| ZNF438 | 0.356 | Upregulated | 6.23E-04 | 5.71E-03 |
| STEAP4 | 0.357 | Upregulated | 1.67E-03 | 1.23E-02 |
| FNDC3A | 0.357 | Upregulated | 3.02E-04 | 3.27E-03 |
| RNF24 | 0.357 | Upregulated | 4.40E-03 | 2.62E-02 |
| NFIL3 | 0.357 | Upregulated | 2.08E-03 | 1.46E-02 |
| ALS2CR14 | 0.359 | Upregulated | 7.45E-04 | 6.55E-03 |
| CPEB4 | 0.359 | Upregulated | 1.96E-04 | 2.36E-03 |
| FLI1 | 0.359 | Upregulated | 5.89E-05 | 9.40E-04 |
| ODF3B | 0.359 | Upregulated | 4.33E-06 | 1.29E-04 |
| MCTP1 | 0.361 | Upregulated | 1.05E-06 | 4.78E-05 |
| HNRPC | 0.361 | Upregulated | 9.93E-05 | 1.40E-03 |
| PKN2 | 0.361 | Upregulated | 2.87E-07 | 1.94E-05 |
| GM2A | 0.361 | Upregulated | 4.45E-05 | 7.55E-04 |
| LTB4R | 0.361 | Upregulated | 1.28E-03 | 9.97E-03 |
| ZNF860 | 0.361 | Upregulated | 1.16E-04 | 1.59E-03 |
| SV2A | 0.361 | Upregulated | 2.79E-04 | 3.07E-03 |
| CTSA | 0.361 | Upregulated | 2.51E-04 | 2.84E-03 |
| SPATA2L | 0.362 | Upregulated | 2.82E-09 | 8.22E-07 |
| HIST1H2AD | 0.362 | Upregulated | 5.17E-06 | 1.47E-04 |
| RTN3 | 0.363 | Upregulated | 2.89E-05 | 5.41E-04 |
| OR52K2 | 0.364 | Upregulated | 1.95E-06 | 7.33E-05 |
| GADD45B | 0.364 | Upregulated | 3.29E-07 | 2.14E-05 |
| MPL | 0.364 | Upregulated | 1.12E-02 | 5.44E-02 |
| ACRBP | 0.365 | Upregulated | 3.47E-03 | 2.18E-02 |
| EPB41L3 | 0.365 | Upregulated | 3.64E-05 | 6.49E-04 |
| SLC22A4 | 0.365 | Upregulated | 3.53E-03 | 2.21E-02 |
| LY96 | 0.365 | Upregulated | 2.28E-02 | 9.25E-02 |
| HLX | 0.365 | Upregulated | 6.32E-05 | 9.90E-04 |
| ASAP2 | 0.365 | Upregulated | 1.56E-06 | 6.29E-05 |
| MTF2 | 0.365 | Upregulated | 2.83E-07 | 1.92E-05 |
| APOL6 | 0.366 | Upregulated | 1.52E-05 | 3.31E-04 |
| DDX60L | 0.366 | Upregulated | 2.06E-03 | 1.45E-02 |
| ARHGAP25 | 0.366 | Upregulated | 1.63E-08 | 2.58E-06 |
| NFXL1 | 0.367 | Upregulated | 1.01E-02 | 4.98E-02 |
| ECHDC1 | 0.367 | Upregulated | 3.57E-06 | 1.13E-04 |
| RPS28 | 0.367 | Upregulated | 1.21E-03 | 9.58E-03 |
| LY6G6F | 0.367 | Upregulated | 4.59E-04 | 4.53E-03 |
| CD226 | 0.367 | Upregulated | 3.41E-03 | 2.15E-02 |
| NUBPL | 0.368 | Upregulated | 7.48E-04 | 6.56E-03 |
| JMJD1A | 0.368 | Upregulated | 1.21E-05 | 2.80E-04 |
| FPR1 | 0.368 | Upregulated | 1.48E-03 | 1.12E-02 |
| CECR6 | 0.369 | Upregulated | 3.74E-04 | 3.87E-03 |
| SORL1 | 0.369 | Upregulated | 1.50E-03 | 1.13E-02 |
| NAT8B | 0.369 | Upregulated | 1.35E-03 | 1.04E-02 |

| | | | | |
|---|---|---|---|---|
| ZNF223 | 0.369 | Upregulated | 2.36E-03 | 1.61E-02 |
| HIST2H2AA3 | 0.369 | Upregulated | 1.09E-03 | 8.84E-03 |
| RAB6A | 0.370 | Upregulated | 3.33E-06 | 1.08E-04 |
| DARC | 0.370 | Upregulated | 2.03E-02 | 8.48E-02 |
| SLC1A5 | 0.370 | Upregulated | 6.21E-05 | 9.79E-04 |
| MEGF9 | 0.370 | Upregulated | 2.65E-04 | 2.96E-03 |
| BLZF1 | 0.370 | Upregulated | 3.13E-05 | 5.76E-04 |
| SSH1 | 0.371 | Upregulated | 7.34E-04 | 6.48E-03 |
| PRO1853 | 0.371 | Upregulated | 1.28E-03 | 9.99E-03 |
| SLK | 0.371 | Upregulated | 1.10E-05 | 2.60E-04 |
| TRAFD1 | 0.372 | Upregulated | 3.96E-07 | 2.43E-05 |
| TGFA | 0.372 | Upregulated | 1.68E-03 | 1.23E-02 |
| ATF5 | 0.372 | Upregulated | 2.32E-04 | 2.69E-03 |
| TNFAIP6 | 0.372 | Upregulated | 1.59E-06 | 6.37E-05 |
| LYPLA1 | 0.372 | Upregulated | 1.32E-06 | 5.67E-05 |
| VSIG4 | 0.373 | Upregulated | 6.27E-04 | 5.73E-03 |
| TSHZ3 | 0.373 | Upregulated | 7.49E-04 | 6.57E-03 |
| MYBL1 | 0.373 | Upregulated | 1.69E-03 | 1.24E-02 |
| MOBKL1B | 0.374 | Upregulated | 9.62E-06 | 2.35E-04 |
| HIAT1 | 0.374 | Upregulated | 2.74E-08 | 3.52E-06 |
| MPO | 0.374 | Upregulated | 1.73E-02 | 7.52E-02 |
| FLJ34047 | 0.374 | Upregulated | 1.57E-03 | 1.17E-02 |
| IL13RA1 | 0.375 | Upregulated | 1.41E-04 | 1.83E-03 |
| KLHDC8B | 0.375 | Upregulated | 5.37E-04 | 5.08E-03 |
| BEST1 | 0.375 | Upregulated | 5.64E-04 | 5.28E-03 |
| DDX6 | 0.375 | Upregulated | 5.62E-05 | 9.04E-04 |
| FCHO2 | 0.375 | Upregulated | 3.31E-03 | 2.10E-02 |
| TET2 | 0.376 | Upregulated | 1.65E-06 | 6.50E-05 |
| DICER1 | 0.376 | Upregulated | 4.69E-08 | 5.05E-06 |
| FTH1 | 0.376 | Upregulated | 4.66E-05 | 7.81E-04 |
| ITGAM | 0.377 | Upregulated | 7.73E-04 | 6.73E-03 |
| SPHAR | 0.377 | Upregulated | 5.04E-06 | 1.45E-04 |
| CR1 | 0.377 | Upregulated | 2.10E-03 | 1.47E-02 |
| CARD16 | 0.378 | Upregulated | 5.56E-06 | 1.54E-04 |
| PREX1 | 0.378 | Upregulated | 1.53E-05 | 3.33E-04 |
| EMR1 | 0.378 | Upregulated | 1.31E-02 | 6.11E-02 |
| EIF2AK4 | 0.378 | Upregulated | 5.13E-05 | 8.43E-04 |
| HLA-DRB3 | 0.379 | Upregulated | 4.22E-04 | 4.25E-03 |
| KIAA1539 | 0.380 | Upregulated | 5.18E-06 | 1.47E-04 |
| RAX2 | 0.380 | Upregulated | 9.35E-05 | 1.34E-03 |
| FCGR2A | 0.380 | Upregulated | 1.15E-03 | 9.19E-03 |
| FZD2 | 0.380 | Upregulated | 2.20E-04 | 2.58E-03 |
| TFE3 | 0.380 | Upregulated | 5.92E-07 | 3.18E-05 |
| ABLIM3 | 0.381 | Upregulated | 1.92E-04 | 2.32E-03 |
| TMEM86B | 0.381 | Upregulated | 1.06E-03 | 8.65E-03 |
| ARID4B | 0.381 | Upregulated | 1.48E-06 | 6.10E-05 |
| MBNL2 | 0.382 | Upregulated | 4.16E-04 | 4.20E-03 |
| IFP38 | 0.382 | Upregulated | 1.04E-05 | 2.49E-04 |
| IL1RAP | 0.382 | Upregulated | 1.12E-04 | 1.54E-03 |
| PXN | 0.383 | Upregulated | 7.69E-06 | 1.97E-04 |
| CSF3R | 0.383 | Upregulated | 1.73E-03 | 1.26E-02 |

| | | | | |
|---|---|---|---|---|
| IFI27L2 | 0.383 | Upregulated | 8.21E-07 | 4.06E-05 |
| AQP12A | 0.383 | Upregulated | 7.43E-05 | 1.13E-03 |
| MAPK14 | 0.384 | Upregulated | 1.07E-03 | 8.71E-03 |
| MED21 | 0.384 | Upregulated | 5.43E-08 | 5.76E-06 |
| GTF2H1 | 0.384 | Upregulated | 7.31E-06 | 1.90E-04 |
| TMEM56 | 0.384 | Upregulated | 8.51E-03 | 4.37E-02 |
| RAB24 | 0.384 | Upregulated | 2.69E-05 | 5.11E-04 |
| THEX1 | 0.385 | Upregulated | 5.60E-06 | 1.55E-04 |
| IFIT3 | 0.385 | Upregulated | 3.22E-02 | 1.20E-01 |
| CAPZA1 | 0.385 | Upregulated | 8.03E-07 | 3.99E-05 |
| FAM104A | 0.385 | Upregulated | 4.95E-04 | 4.78E-03 |
| SEC24A | 0.386 | Upregulated | 1.07E-04 | 1.49E-03 |
| C1QA | 0.386 | Upregulated | 1.07E-04 | 1.49E-03 |
| FLVCR2 | 0.386 | Upregulated | 4.52E-06 | 1.34E-04 |
| IL1B | 0.387 | Upregulated | 4.38E-03 | 2.61E-02 |
| NOL10 | 0.387 | Upregulated | 7.72E-04 | 6.72E-03 |
| RAXL1 | 0.387 | Upregulated | 1.40E-05 | 3.13E-04 |
| ERGIC1 | 0.387 | Upregulated | 3.40E-05 | 6.13E-04 |
| TRIM58 | 0.387 | Upregulated | 4.66E-02 | 1.57E-01 |
| EIF1AD | 0.387 | Upregulated | 2.16E-05 | 4.34E-04 |
| ALPK1 | 0.387 | Upregulated | 2.73E-04 | 3.02E-03 |
| SLC5A8 | 0.388 | Upregulated | 1.33E-03 | 1.03E-02 |
| OSBPL8 | 0.388 | Upregulated | 3.53E-07 | 2.22E-05 |
| CYB561 | 0.388 | Upregulated | 1.71E-13 | 7.42E-10 |
| GRN | 0.388 | Upregulated | 6.65E-05 | 1.03E-03 |
| GPER | 0.388 | Upregulated | 1.16E-02 | 5.57E-02 |
| ZMYND15 | 0.388 | Upregulated | 1.41E-03 | 1.07E-02 |
| ANPEP | 0.389 | Upregulated | 6.72E-03 | 3.64E-02 |
| SCO2 | 0.389 | Upregulated | 3.11E-04 | 3.34E-03 |
| MTMR6 | 0.390 | Upregulated | 5.59E-05 | 9.00E-04 |
| VWCE | 0.390 | Upregulated | 4.82E-02 | 1.60E-01 |
| PLA2G4A | 0.390 | Upregulated | 1.04E-07 | 9.53E-06 |
| CXCL5 | 0.390 | Upregulated | 8.83E-04 | 7.49E-03 |
| FAM175A | 0.390 | Upregulated | 1.94E-04 | 2.34E-03 |
| DSC2 | 0.391 | Upregulated | 3.80E-03 | 2.34E-02 |
| ZDHHC18 | 0.391 | Upregulated | 1.23E-04 | 1.66E-03 |
| RNASEH2B | 0.392 | Upregulated | 5.39E-04 | 5.10E-03 |
| HSD3B7 | 0.392 | Upregulated | 6.42E-04 | 5.83E-03 |
| TSPAN5 | 0.394 | Upregulated | 2.50E-02 | 9.92E-02 |
| GNA13 | 0.394 | Upregulated | 1.32E-06 | 5.66E-05 |
| RGMA | 0.395 | Upregulated | 2.23E-08 | 3.13E-06 |
| IGSF6 | 0.396 | Upregulated | 3.90E-04 | 3.99E-03 |
| CTBS | 0.396 | Upregulated | 5.87E-06 | 1.59E-04 |
| ANKRD9 | 0.397 | Upregulated | 8.27E-04 | 7.08E-03 |
| IL17RA | 0.398 | Upregulated | 6.59E-05 | 1.02E-03 |
| MICALCL | 0.400 | Upregulated | 2.01E-03 | 1.42E-02 |
| HSPC159 | 0.400 | Upregulated | 1.71E-04 | 2.13E-03 |
| TAOK1 | 0.400 | Upregulated | 3.46E-05 | 6.21E-04 |
| SHKBP1 | 0.400 | Upregulated | 2.25E-04 | 2.62E-03 |
| CMBL | 0.400 | Upregulated | 1.11E-02 | 5.37E-02 |
| SQRDL | 0.401 | Upregulated | 1.19E-06 | 5.25E-05 |

| | | | | |
|---|---|---|---|---|
| IL18RAP | 0.401 | Upregulated | 1.13E-03 | 9.08E-03 |
| MFSD11 | 0.402 | Upregulated | 1.87E-06 | 7.15E-05 |
| UBE2H | 0.402 | Upregulated | 1.77E-05 | 3.72E-04 |
| TCN2 | 0.402 | Upregulated | 6.58E-04 | 5.95E-03 |
| HSPB1 | 0.403 | Upregulated | 4.35E-04 | 4.34E-03 |
| CNIH4 | 0.404 | Upregulated | 6.58E-05 | 1.02E-03 |
| RNF149 | 0.405 | Upregulated | 8.57E-06 | 2.14E-04 |
| TCN1 | 0.405 | Upregulated | 9.51E-03 | 4.76E-02 |
| FURIN | 0.405 | Upregulated | 4.57E-05 | 7.71E-04 |
| CCDC125 | 0.405 | Upregulated | 3.72E-05 | 6.61E-04 |
| PINK1 | 0.405 | Upregulated | 2.54E-05 | 4.88E-04 |
| CRISPLD2 | 0.406 | Upregulated | 4.40E-03 | 2.62E-02 |
| DNTTIP2 | 0.408 | Upregulated | 2.08E-05 | 4.23E-04 |
| SAP30 | 0.409 | Upregulated | 7.88E-04 | 6.84E-03 |
| ZAK | 0.410 | Upregulated | 1.44E-05 | 3.18E-04 |
| CXCL9 | 0.410 | Upregulated | 5.23E-05 | 8.53E-04 |
| INDO | 0.410 | Upregulated | 2.09E-02 | 8.68E-02 |
| SESN3 | 0.410 | Upregulated | 2.36E-02 | 9.49E-02 |
| CXCR1 | 0.411 | Upregulated | 6.06E-03 | 3.35E-02 |
| LSM6 | 0.411 | Upregulated | 3.10E-07 | 2.05E-05 |
| SDPR | 0.412 | Upregulated | 8.07E-04 | 6.96E-03 |
| BMP8B | 0.412 | Upregulated | 9.14E-04 | 7.69E-03 |
| GNB4 | 0.413 | Upregulated | 2.24E-07 | 1.64E-05 |
| NAT13 | 0.413 | Upregulated | 1.21E-07 | 1.04E-05 |
| NPTN | 0.413 | Upregulated | 9.76E-06 | 2.38E-04 |
| USP25 | 0.414 | Upregulated | 1.74E-06 | 6.80E-05 |
| NFIX | 0.414 | Upregulated | 5.39E-03 | 3.06E-02 |
| FAM117A | 0.415 | Upregulated | 9.48E-06 | 2.33E-04 |
| CCDC52 | 0.415 | Upregulated | 5.82E-04 | 5.41E-03 |
| FAM73A | 0.416 | Upregulated | 3.01E-06 | 1.02E-04 |
| DPYD | 0.416 | Upregulated | 1.24E-06 | 5.40E-05 |
| DGAT2 | 0.416 | Upregulated | 1.88E-04 | 2.28E-03 |
| NAP1L1 | 0.417 | Upregulated | 2.10E-05 | 4.24E-04 |
| CBS | 0.417 | Upregulated | 3.50E-03 | 2.19E-02 |
| FAM21A | 0.418 | Upregulated | 6.00E-06 | 1.62E-04 |
| RAD21 | 0.419 | Upregulated | 1.54E-06 | 6.26E-05 |
| VWF | 0.419 | Upregulated | 1.60E-03 | 1.19E-02 |
| BPI | 0.420 | Upregulated | 1.40E-02 | 6.40E-02 |
| CSF2RB | 0.420 | Upregulated | 4.15E-05 | 7.17E-04 |
| CPPED1 | 0.421 | Upregulated | 2.09E-05 | 4.23E-04 |
| LTBR | 0.424 | Upregulated | 3.68E-05 | 6.56E-04 |
| GTF2H2 | 0.426 | Upregulated | 6.06E-05 | 9.60E-04 |
| LMO2 | 0.427 | Upregulated | 1.02E-06 | 4.69E-05 |
| SLC14A1 | 0.427 | Upregulated | 7.32E-05 | 1.11E-03 |
| ELOVL5 | 0.429 | Upregulated | 3.16E-06 | 1.05E-04 |
| PLXDC2 | 0.429 | Upregulated | 8.07E-06 | 2.05E-04 |
| ZBTB44 | 0.430 | Upregulated | 1.75E-06 | 6.81E-05 |
| GPR109B | 0.430 | Upregulated | 4.66E-05 | 7.81E-04 |
| GADD45G | 0.430 | Upregulated | 1.79E-05 | 3.74E-04 |
| BEND7 | 0.431 | Upregulated | 1.70E-07 | 1.33E-05 |
| ZNF641 | 0.431 | Upregulated | 1.61E-05 | 3.46E-04 |

| | | | | |
|---|---|---|---|---|
| GP9 | 0.431 | Upregulated | 2.30E-03 | 1.58E-02 |
| RAB11FIP1 | 0.432 | Upregulated | 1.56E-06 | 6.29E-05 |
| PTPLAD2 | 0.432 | Upregulated | 2.47E-04 | 2.81E-03 |
| ZBTB11 | 0.432 | Upregulated | 1.26E-05 | 2.89E-04 |
| ANKRD33 | 0.433 | Upregulated | 2.68E-05 | 5.09E-04 |
| IGF2BP2 | 0.433 | Upregulated | 8.67E-03 | 4.43E-02 |
| RSAD2 | 0.433 | Upregulated | 3.94E-02 | 1.39E-01 |
| RTN4 | 0.434 | Upregulated | 1.07E-09 | 4.40E-07 |
| PHF20L1 | 0.434 | Upregulated | 3.51E-09 | 9.47E-07 |
| IRF2 | 0.435 | Upregulated | 4.08E-08 | 4.57E-06 |
| CFP | 0.435 | Upregulated | 6.70E-06 | 1.77E-04 |
| CMTM5 | 0.435 | Upregulated | 3.65E-04 | 3.78E-03 |
| SLC6A8 | 0.436 | Upregulated | 6.27E-03 | 3.44E-02 |
| SLC40A1 | 0.436 | Upregulated | 4.05E-06 | 1.23E-04 |
| SBDS | 0.437 | Upregulated | 2.60E-07 | 1.80E-05 |
| CD46 | 0.437 | Upregulated | 2.74E-06 | 9.55E-05 |
| CLTC | 0.438 | Upregulated | 1.35E-06 | 5.73E-05 |
| PLXNC1 | 0.438 | Upregulated | 8.75E-05 | 1.28E-03 |
| ITGB5 | 0.439 | Upregulated | 4.60E-05 | 7.75E-04 |
| SOD2 | 0.440 | Upregulated | 5.76E-04 | 5.37E-03 |
| IDI1 | 0.441 | Upregulated | 1.58E-05 | 3.41E-04 |
| RHAG | 0.441 | Upregulated | 8.56E-04 | 7.29E-03 |
| FECH | 0.442 | Upregulated | 5.37E-03 | 3.06E-02 |
| DHRS9 | 0.442 | Upregulated | 1.04E-05 | 2.49E-04 |
| PTGS2 | 0.443 | Upregulated | 2.89E-04 | 3.15E-03 |
| MEFV | 0.443 | Upregulated | 1.26E-05 | 2.89E-04 |
| BMP2K | 0.443 | Upregulated | 4.43E-07 | 2.64E-05 |
| PLAGL1 | 0.444 | Upregulated | 1.61E-08 | 2.58E-06 |
| SLC26A8 | 0.446 | Upregulated | 2.23E-05 | 4.44E-04 |
| SPRYD3 | 0.447 | Upregulated | 1.38E-03 | 1.06E-02 |
| KCTD12 | 0.448 | Upregulated | 8.68E-06 | 2.16E-04 |
| B3GNT5 | 0.448 | Upregulated | 3.55E-06 | 1.13E-04 |
| NXT2 | 0.448 | Upregulated | 7.50E-06 | 1.93E-04 |
| RAB6B | 0.448 | Upregulated | 3.19E-08 | 3.96E-06 |
| SPATA13 | 0.449 | Upregulated | 4.40E-04 | 4.38E-03 |
| KEL | 0.449 | Upregulated | 2.26E-04 | 2.63E-03 |
| CCR1 | 0.450 | Upregulated | 1.02E-04 | 1.44E-03 |
| ADM | 0.451 | Upregulated | 7.35E-04 | 6.48E-03 |
| NDUFAF3 | 0.451 | Upregulated | 1.46E-04 | 1.89E-03 |
| VCAN | 0.451 | Upregulated | 1.49E-04 | 1.91E-03 |
| TMOD1 | 0.452 | Upregulated | 2.01E-02 | 8.44E-02 |
| PLEK2 | 0.452 | Upregulated | 3.44E-03 | 2.17E-02 |
| SOCS3 | 0.453 | Upregulated | 3.49E-06 | 1.11E-04 |
| SAMSN1 | 0.453 | Upregulated | 3.76E-05 | 6.66E-04 |
| SEC24D | 0.454 | Upregulated | 1.28E-06 | 5.53E-05 |
| GPR97 | 0.454 | Upregulated | 2.96E-03 | 1.93E-02 |
| SBNO2 | 0.454 | Upregulated | 1.38E-06 | 5.80E-05 |
| GBP4 | 0.455 | Upregulated | 8.00E-06 | 2.03E-04 |
| NLK | 0.455 | Upregulated | 2.30E-05 | 4.55E-04 |
| LTF | 0.456 | Upregulated | 6.81E-03 | 3.68E-02 |
| USP49 | 0.457 | Upregulated | 3.28E-05 | 5.97E-04 |

| | | | | |
|---|---|---|---|---|
| ECE1 | 0.457 | Upregulated | 1.45E-06 | 6.02E-05 |
| GPR175 | 0.459 | Upregulated | 1.76E-02 | 7.62E-02 |
| APOL1 | 0.459 | Upregulated | 4.87E-09 | 1.17E-06 |
| RBM38 | 0.459 | Upregulated | 1.51E-03 | 1.13E-02 |
| ESAM | 0.460 | Upregulated | 2.47E-04 | 2.81E-03 |
| TYMP | 0.461 | Upregulated | 2.14E-05 | 4.31E-04 |
| LAP3 | 0.463 | Upregulated | 1.01E-05 | 2.43E-04 |
| MCTS1 | 0.465 | Upregulated | 2.35E-04 | 2.71E-03 |
| EAF1 | 0.465 | Upregulated | 1.34E-07 | 1.12E-05 |
| PCAF | 0.465 | Upregulated | 2.16E-07 | 1.60E-05 |
| FFAR2 | 0.466 | Upregulated | 1.58E-03 | 1.18E-02 |
| HNRPLL | 0.466 | Upregulated | 2.01E-09 | 6.70E-07 |
| FRMD3 | 0.466 | Upregulated | 1.85E-07 | 1.41E-05 |
| TSC22D1 | 0.467 | Upregulated | 5.66E-06 | 1.56E-04 |
| MGC13057 | 0.468 | Upregulated | 2.01E-03 | 1.42E-02 |
| VTI1B | 0.468 | Upregulated | 8.60E-08 | 8.25E-06 |
| CIR1 | 0.471 | Upregulated | 7.15E-13 | 1.91E-09 |
| ITGAX | 0.472 | Upregulated | 7.03E-06 | 1.83E-04 |
| ZCCHC6 | 0.474 | Upregulated | 1.99E-10 | 1.25E-07 |
| NBEAL2 | 0.475 | Upregulated | 3.48E-08 | 4.18E-06 |
| GRAMD1B | 0.475 | Upregulated | 8.51E-06 | 2.13E-04 |
| TNS1 | 0.475 | Upregulated | 1.51E-02 | 6.79E-02 |
| HIST1H3D | 0.476 | Upregulated | 3.72E-06 | 1.16E-04 |
| TSPAN9 | 0.476 | Upregulated | 3.38E-04 | 3.56E-03 |
| USP6 | 0.477 | Upregulated | 1.30E-05 | 2.95E-04 |
| EPB49 | 0.478 | Upregulated | 1.07E-02 | 5.24E-02 |
| GSTA5 | 0.480 | Upregulated | 1.85E-03 | 1.33E-02 |
| LACTB | 0.481 | Upregulated | 1.04E-06 | 4.78E-05 |
| ABP1 | 0.483 | Upregulated | 1.03E-02 | 5.05E-02 |
| NOD2 | 0.483 | Upregulated | 4.70E-06 | 1.37E-04 |
| SSTR2 | 0.485 | Upregulated | 2.64E-05 | 5.04E-04 |
| GRINA | 0.485 | Upregulated | 2.97E-05 | 5.55E-04 |
| YPEL4 | 0.486 | Upregulated | 4.37E-06 | 1.30E-04 |
| GNG11 | 0.487 | Upregulated | 6.11E-05 | 9.66E-04 |
| LRG1 | 0.488 | Upregulated | 1.08E-03 | 8.78E-03 |
| TANK | 0.489 | Upregulated | 3.74E-09 | 9.75E-07 |
| XK | 0.490 | Upregulated | 1.87E-03 | 1.34E-02 |
| PRDM1 | 0.490 | Upregulated | 2.87E-07 | 1.94E-05 |
| POTEE | 0.492 | Upregulated | 4.30E-07 | 2.59E-05 |
| JAM3 | 0.493 | Upregulated | 4.07E-05 | 7.05E-04 |
| ETV7 | 0.493 | Upregulated | 3.88E-06 | 1.20E-04 |
| MMRN1 | 0.494 | Upregulated | 2.70E-05 | 5.11E-04 |
| SERPING1 | 0.494 | Upregulated | 1.60E-06 | 6.38E-05 |
| CEACAM6 | 0.494 | Upregulated | 1.57E-02 | 7.00E-02 |
| MBOAT7 | 0.495 | Upregulated | 1.49E-04 | 1.91E-03 |
| TLR2 | 0.495 | Upregulated | 6.52E-04 | 5.90E-03 |
| GMPR | 0.496 | Upregulated | 8.68E-03 | 4.43E-02 |
| DUSP3 | 0.496 | Upregulated | 3.02E-09 | 8.65E-07 |
| RPS4Y2 | 0.496 | Upregulated | 3.49E-06 | 1.11E-04 |
| TIMM10 | 0.497 | Upregulated | 1.22E-03 | 9.65E-03 |
| LRRK2 | 0.498 | Upregulated | 7.31E-07 | 3.71E-05 |

| | | | | |
|---|---|---|---|---|
| DAPP1 | 0.499 | Upregulated | 7.96E-11 | 6.28E-08 |
| OSM | 0.499 | Upregulated | 1.47E-05 | 3.24E-04 |
| DYSF | 0.499 | Upregulated | 9.66E-04 | 8.03E-03 |
| FAS | 0.500 | Upregulated | 8.39E-09 | 1.73E-06 |
| RAB20 | 0.500 | Upregulated | 8.86E-07 | 4.30E-05 |
| SPOPL | 0.502 | Upregulated | 2.58E-06 | 9.10E-05 |
| CXCL10 | 0.502 | Upregulated | 1.57E-03 | 1.17E-02 |
| HIST1H1C | 0.504 | Upregulated | 2.14E-05 | 4.31E-04 |
| CD97 | 0.504 | Upregulated | 1.79E-06 | 6.95E-05 |
| PF4V1 | 0.506 | Upregulated | 8.68E-04 | 7.38E-03 |
| FCGR3B | 0.507 | Upregulated | 3.94E-04 | 4.02E-03 |
| SIPA1L2 | 0.508 | Upregulated | 3.79E-04 | 3.91E-03 |
| NCF2 | 0.508 | Upregulated | 1.72E-07 | 1.34E-05 |
| KLF1 | 0.509 | Upregulated | 2.55E-04 | 2.88E-03 |
| F13A1 | 0.509 | Upregulated | 2.95E-04 | 3.20E-03 |
| FAM8A1 | 0.509 | Upregulated | 1.61E-08 | 2.58E-06 |
| AIM2 | 0.510 | Upregulated | 2.53E-08 | 3.34E-06 |
| ANXA4 | 0.511 | Upregulated | 2.08E-05 | 4.22E-04 |
| WARS | 0.511 | Upregulated | 3.54E-06 | 1.13E-04 |
| RHD | 0.513 | Upregulated | 1.78E-03 | 1.29E-02 |
| LCN2 | 0.514 | Upregulated | 4.85E-03 | 2.82E-02 |
| SLC6A12 | 0.514 | Upregulated | 3.09E-05 | 5.71E-04 |
| RRAGD | 0.514 | Upregulated | 6.00E-06 | 1.62E-04 |
| PDZK1IP1 | 0.516 | Upregulated | 1.18E-02 | 5.65E-02 |
| KCNJ2 | 0.516 | Upregulated | 1.09E-05 | 2.57E-04 |
| RPL23 | 0.517 | Upregulated | 3.54E-03 | 2.22E-02 |
| EMB | 0.517 | Upregulated | 2.53E-08 | 3.34E-06 |
| SPARC | 0.517 | Upregulated | 9.70E-06 | 2.37E-04 |
| FBXO38 | 0.518 | Upregulated | 8.40E-07 | 4.13E-05 |
| DPYSL5 | 0.519 | Upregulated | 2.08E-02 | 8.64E-02 |
| IL18R1 | 0.520 | Upregulated | 2.58E-03 | 1.73E-02 |
| GPR84 | 0.521 | Upregulated | 3.66E-03 | 2.27E-02 |
| TNFAIP2 | 0.521 | Upregulated | 3.17E-06 | 1.05E-04 |
| ACTR3 | 0.523 | Upregulated | 3.18E-06 | 1.05E-04 |
| BNIP3L | 0.525 | Upregulated | 1.26E-04 | 1.68E-03 |
| GIMAP2 | 0.525 | Upregulated | 9.40E-06 | 2.32E-04 |
| CARD17 | 0.525 | Upregulated | 2.67E-05 | 5.07E-04 |
| GP6 | 0.526 | Upregulated | 4.66E-07 | 2.72E-05 |
| METTL7A | 0.527 | Upregulated | 2.79E-06 | 9.68E-05 |
| CHPT1 | 0.528 | Upregulated | 1.34E-04 | 1.76E-03 |
| GPR65 | 0.531 | Upregulated | 3.93E-10 | 2.20E-07 |
| LIMK2 | 0.536 | Upregulated | 3.10E-05 | 5.73E-04 |
| AHSP | 0.538 | Upregulated | 1.12E-02 | 5.43E-02 |
| FAM44A | 0.538 | Upregulated | 6.73E-08 | 6.80E-06 |
| VAMP5 | 0.538 | Upregulated | 1.42E-07 | 1.17E-05 |
| MMP9 | 0.541 | Upregulated | 1.19E-02 | 5.66E-02 |
| GNG10 | 0.541 | Upregulated | 2.25E-06 | 8.19E-05 |
| TFPI | 0.542 | Upregulated | 6.75E-07 | 3.53E-05 |
| NRGN | 0.542 | Upregulated | 3.19E-04 | 3.41E-03 |
| RNF213 | 0.543 | Upregulated | 2.76E-04 | 3.04E-03 |
| KLHL2 | 0.543 | Upregulated | 1.74E-05 | 3.68E-04 |

| | | | | |
|---|---|---|---|---|
| ADCY3 | 0.545 | Upregulated | 5.10E-06 | 1.46E-04 |
| KIFC3 | 0.548 | Upregulated | 8.69E-09 | 1.74E-06 |
| PDCD1LG2 | 0.549 | Upregulated | 9.18E-07 | 4.39E-05 |
| RAP1BL | 0.550 | Upregulated | 2.07E-05 | 4.20E-04 |
| C2 | 0.553 | Upregulated | 8.61E-08 | 8.25E-06 |
| ALDH1A1 | 0.553 | Upregulated | 2.30E-06 | 8.35E-05 |
| ZDHHC19 | 0.561 | Upregulated | 3.40E-02 | 1.25E-01 |
| HIF1A | 0.563 | Upregulated | 1.01E-06 | 4.66E-05 |
| BAGE5 | 0.564 | Upregulated | 1.13E-07 | 9.97E-06 |
| SMARCD3 | 0.566 | Upregulated | 1.00E-05 | 2.43E-04 |
| FER1L3 | 0.568 | Upregulated | 5.27E-06 | 1.49E-04 |
| TXNL1 | 0.569 | Upregulated | 5.16E-06 | 1.47E-04 |
| TUBA4A | 0.570 | Upregulated | 5.69E-09 | 1.31E-06 |
| SLC2A14 | 0.576 | Upregulated | 1.23E-04 | 1.66E-03 |
| CTDSPL | 0.576 | Upregulated | 9.80E-06 | 2.39E-04 |
| SFRS11 | 0.576 | Upregulated | 9.46E-07 | 4.47E-05 |
| HMGB1L1 | 0.577 | Upregulated | 3.86E-08 | 4.46E-06 |
| CLK1 | 0.578 | Upregulated | 1.19E-05 | 2.75E-04 |
| PPP2CB | 0.579 | Upregulated | 3.26E-07 | 2.13E-05 |
| PRKAR1A | 0.580 | Upregulated | 3.82E-03 | 2.35E-02 |
| FLJ20309 | 0.585 | Upregulated | 6.16E-05 | 9.73E-04 |
| FAM129A | 0.588 | Upregulated | 3.26E-06 | 1.07E-04 |
| CYP4F3 | 0.588 | Upregulated | 2.45E-04 | 2.79E-03 |
| FYB | 0.588 | Upregulated | 4.46E-12 | 6.19E-09 |
| C4BPA | 0.590 | Upregulated | 1.53E-02 | 6.85E-02 |
| VPS26 | 0.590 | Upregulated | 3.29E-07 | 2.14E-05 |
| SOCS1 | 0.590 | Upregulated | 2.31E-08 | 3.17E-06 |
| ITPRIPL2 | 0.592 | Upregulated | 1.23E-06 | 5.37E-05 |
| RAB3IL1 | 0.593 | Upregulated | 5.75E-04 | 5.36E-03 |
| SAMD14 | 0.594 | Upregulated | 8.20E-06 | 2.07E-04 |
| MYOF | 0.594 | Upregulated | 3.73E-06 | 1.16E-04 |
| TPST1 | 0.596 | Upregulated | 4.50E-04 | 4.46E-03 |
| TREML1 | 0.596 | Upregulated | 3.07E-05 | 5.70E-04 |
| TMCC2 | 0.596 | Upregulated | 4.81E-04 | 4.68E-03 |
| CEACAM8 | 0.600 | Upregulated | 5.30E-03 | 3.02E-02 |
| SIAH2 | 0.600 | Upregulated | 8.60E-06 | 2.15E-04 |
| NCOA1 | 0.601 | Upregulated | 7.82E-09 | 1.64E-06 |
| CA1 | 0.605 | Upregulated | 2.26E-02 | 9.19E-02 |
| WSB1 | 0.607 | Upregulated | 3.08E-09 | 8.76E-07 |
| MYADM | 0.610 | Upregulated | 1.41E-05 | 3.14E-04 |
| HECA | 0.610 | Upregulated | 2.48E-08 | 3.29E-06 |
| HBD | 0.611 | Upregulated | 1.58E-03 | 1.18E-02 |
| PPBP | 0.612 | Upregulated | 4.22E-05 | 7.27E-04 |
| TMEM119 | 0.616 | Upregulated | 5.69E-03 | 3.19E-02 |
| HDAC4 | 0.617 | Upregulated | 1.33E-06 | 5.69E-05 |
| P2RY13 | 0.617 | Upregulated | 2.90E-07 | 1.95E-05 |
| TMEM158 | 0.617 | Upregulated | 2.94E-04 | 3.20E-03 |
| ANP32A | 0.617 | Upregulated | 1.86E-08 | 2.79E-06 |
| TTRAP | 0.618 | Upregulated | 8.00E-08 | 7.83E-06 |
| PTGES3 | 0.618 | Upregulated | 2.71E-06 | 9.48E-05 |
| KIAA1033 | 0.623 | Upregulated | 2.75E-06 | 9.57E-05 |

| | | | | |
|---|---|---|---|---|
| SNX10 | 0.623 | Upregulated | 2.95E-07 | 1.98E-05 |
| HIST2H2AB | 0.623 | Upregulated | 5.13E-07 | 2.88E-05 |
| HPSE | 0.625 | Upregulated | 3.67E-07 | 2.29E-05 |
| ERGIC2 | 0.625 | Upregulated | 1.01E-06 | 4.67E-05 |
| SLAMF8 | 0.625 | Upregulated | 6.65E-08 | 6.78E-06 |
| SH3BGRL2 | 0.628 | Upregulated | 5.57E-06 | 1.55E-04 |
| GK | 0.629 | Upregulated | 3.25E-06 | 1.06E-04 |
| SORT1 | 0.630 | Upregulated | 1.03E-07 | 9.44E-06 |
| SELPLG | 0.636 | Upregulated | 5.26E-09 | 1.23E-06 |
| PPIB | 0.639 | Upregulated | 4.34E-07 | 2.59E-05 |
| HSPA1A | 0.640 | Upregulated | 4.54E-05 | 7.67E-04 |
| BIN2 | 0.642 | Upregulated | 2.32E-08 | 3.17E-06 |
| CACNA1E | 0.643 | Upregulated | 2.27E-04 | 2.64E-03 |
| ADI1 | 0.646 | Upregulated | 6.20E-08 | 6.42E-06 |
| RBPMS2 | 0.654 | Upregulated | 8.90E-04 | 7.53E-03 |
| GBP1 | 0.654 | Upregulated | 1.76E-07 | 1.37E-05 |
| IL27 | 0.654 | Upregulated | 2.17E-06 | 7.95E-05 |
| FAM49B | 0.656 | Upregulated | 6.61E-08 | 6.76E-06 |
| EPB42 | 0.659 | Upregulated | 4.43E-03 | 2.63E-02 |
| ITGB3 | 0.667 | Upregulated | 1.32E-06 | 5.67E-05 |
| PROS1 | 0.673 | Upregulated | 1.11E-06 | 5.00E-05 |
| PSG9 | 0.682 | Upregulated | 1.10E-05 | 2.60E-04 |
| HIST1H2BC | 0.683 | Upregulated | 3.00E-09 | 8.65E-07 |
| ZFP36L1 | 0.686 | Upregulated | 1.14E-07 | 9.97E-06 |
| Septin 7 | 0.686 | Upregulated | 1.52E-06 | 6.21E-05 |
| SPAST | 0.687 | Upregulated | 1.33E-07 | 1.12E-05 |
| CAST | 0.694 | Upregulated | 2.01E-12 | 3.48E-09 |
| GPR109A | 0.696 | Upregulated | 8.71E-08 | 8.33E-06 |
| HIST1H2BE | 0.696 | Upregulated | 2.30E-09 | 7.24E-07 |
| ILK | 0.700 | Upregulated | 5.64E-10 | 2.80E-07 |
| KCNJ15 | 0.704 | Upregulated | 3.26E-07 | 2.13E-05 |
| SLC4A1 | 0.705 | Upregulated | 5.87E-04 | 5.44E-03 |
| BLVRB | 0.710 | Upregulated | 5.54E-06 | 1.54E-04 |
| C1GALT1 | 0.713 | Upregulated | 6.05E-13 | 1.91E-09 |
| F2RL1 | 0.717 | Upregulated | 3.32E-09 | 9.13E-07 |
| CEACAM1 | 0.729 | Upregulated | 9.10E-08 | 8.56E-06 |
| SELENBP1 | 0.733 | Upregulated | 3.79E-03 | 2.33E-02 |
| MBNL3 | 0.734 | Upregulated | 1.82E-05 | 3.80E-04 |
| CASP5 | 0.735 | Upregulated | 6.58E-06 | 1.74E-04 |
| WSB2 | 0.740 | Upregulated | 4.70E-10 | 2.47E-07 |
| FCGR2C | 0.741 | Upregulated | 4.05E-08 | 4.57E-06 |
| PSTPIP2 | 0.751 | Upregulated | 4.79E-09 | 1.17E-06 |
| ANXA3 | 0.757 | Upregulated | 1.13E-04 | 1.56E-03 |
| GBP3 | 0.759 | Upregulated | 2.74E-09 | 8.20E-07 |
| S100A12 | 0.762 | Upregulated | 7.34E-04 | 6.48E-03 |
| ACSL1 | 0.775 | Upregulated | 3.47E-07 | 2.21E-05 |
| LHFPL2 | 0.783 | Upregulated | 2.80E-14 | 2.23E-10 |
| SERPINA13 | 0.797 | Upregulated | 3.49E-03 | 2.19E-02 |
| KREMEN1 | 0.799 | Upregulated | 1.82E-07 | 1.40E-05 |
| MYL9 | 0.803 | Upregulated | 3.52E-07 | 2.22E-05 |
| SELP | 0.805 | Upregulated | 5.64E-11 | 5.02E-08 |

| | | | | |
|---|---|---|---|---|
| CD164 | 0.806 | Upregulated | 1.33E-08 | 2.31E-06 |
| AQP10 | 0.811 | Upregulated | 4.59E-07 | 2.70E-05 |
| HLA-G | 0.815 | Upregulated | 2.25E-12 | 3.71E-09 |
| LGALS3 | 0.816 | Upregulated | 1.60E-08 | 2.58E-06 |
| FLJ20273 | 0.821 | Upregulated | 1.33E-08 | 2.31E-06 |
| APOBEC3A | 0.830 | Upregulated | 2.08E-09 | 6.81E-07 |
| RAB33B | 0.835 | Upregulated | 5.77E-08 | 6.05E-06 |
| P2RY14 | 0.839 | Upregulated | 1.24E-09 | 4.66E-07 |
| RAP1GAP | 0.842 | Upregulated | 8.58E-03 | 4.39E-02 |
| HBE1 | 0.843 | Upregulated | 8.91E-06 | 2.21E-04 |
| C1QB | 0.844 | Upregulated | 6.67E-05 | 1.03E-03 |
| ALAS2 | 0.846 | Upregulated | 3.46E-04 | 3.63E-03 |
| C1QC | 0.847 | Upregulated | 1.34E-05 | 3.02E-04 |
| ITGA2B | 0.853 | Upregulated | 4.39E-07 | 2.62E-05 |
| GBP6 | 0.855 | Upregulated | 1.76E-15 | 3.04E-11 |
| PTMA | 0.867 | Upregulated | 1.09E-06 | 4.92E-05 |
| CD274 | 0.904 | Upregulated | 1.04E-08 | 1.96E-06 |
| PIK3AP1 | 0.915 | Upregulated | 7.92E-10 | 3.82E-07 |
| GBP5 | 0.945 | Upregulated | 1.28E-12 | 2.61E-09 |
| OSBP2 | 0.992 | Upregulated | 1.23E-05 | 2.84E-04 |
| TLR1 | 1.090 | Upregulated | 1.93E-10 | 1.24E-07 |
| FCGR1B | 1.100 | Upregulated | 1.72E-10 | 1.15E-07 |
| FAM26F | 1.117 | Upregulated | 3.59E-14 | 2.23E-10 |
| BATF2 | 1.130 | Upregulated | 9.89E-13 | 2.25E-09 |
| FCGR1A | 1.181 | Upregulated | 2.72E-11 | 2.78E-08 |
| ANKRD22 | 1.232 | Upregulated | 1.67E-11 | 2.00E-08 |
| FCGR1C | 1.324 | Upregulated | 3.86E-14 | 2.23E-10 |

**Supplementary Table S3c. Differentially expressed genes_Malawi_Male**

| Gene | log FoldChange | Direction of expression | P.Value | adj.P.Val |
|---|---|---|---|---|
| RPL14 | -0.690 | Downregulated | 2.68E-03 | 3.53E-01 |
| RETN | -0.643 | Downregulated | 2.90E-02 | 5.81E-01 |
| ORM1 | -0.631 | Downregulated | 3.21E-02 | 5.87E-01 |
| PTMA | -0.613 | Downregulated | 3.12E-02 | 5.86E-01 |
| BPI | -0.590 | Downregulated | 3.47E-02 | 5.95E-01 |
| HSPA1A | -0.524 | Downregulated | 3.26E-02 | 5.88E-01 |
| LRRN3 | -0.522 | Downregulated | 1.23E-02 | 4.94E-01 |
| PTPLAD2 | -0.519 | Downregulated | 3.07E-03 | 3.58E-01 |
| CLK1 | -0.501 | Downregulated | 7.98E-03 | 4.63E-01 |
| LGALS3 | -0.483 | Downregulated | 3.13E-02 | 5.86E-01 |
| ACTR3 | -0.478 | Downregulated | 1.18E-02 | 4.93E-01 |
| CD24 | -0.470 | Downregulated | 1.23E-03 | 2.85E-01 |
| ZNF281 | -0.464 | Downregulated | 3.02E-03 | 3.58E-01 |
| ORM2 | -0.459 | Downregulated | 4.15E-02 | 6.06E-01 |
| BLVRB | -0.458 | Downregulated | 2.78E-02 | 5.76E-01 |
| SSTR2 | -0.454 | Downregulated | 6.18E-03 | 4.48E-01 |
| METTL7A | -0.452 | Downregulated | 9.49E-03 | 4.63E-01 |
| ZNF223 | -0.450 | Downregulated | 3.01E-03 | 3.58E-01 |
| ABCA13 | -0.449 | Downregulated | 4.95E-02 | 6.27E-01 |
| NUBPL | -0.447 | Downregulated | 4.56E-03 | 4.13E-01 |
| CPOX | -0.431 | Downregulated | 4.33E-04 | 2.06E-01 |
| HECA | -0.423 | Downregulated | 1.41E-02 | 5.03E-01 |
| TMEM106A | -0.423 | Downregulated | 1.29E-02 | 4.97E-01 |
| SACM1L | -0.421 | Downregulated | 1.10E-02 | 4.85E-01 |
| PTGES3 | -0.419 | Downregulated | 2.93E-02 | 5.81E-01 |
| RPS4Y2 | -0.406 | Downregulated | 1.73E-02 | 5.25E-01 |
| HNRPC | -0.401 | Downregulated | 1.33E-02 | 4.98E-01 |
| MFSD11 | -0.399 | Downregulated | 4.47E-03 | 4.10E-01 |
| CCDC125 | -0.398 | Downregulated | 6.13E-03 | 4.48E-01 |
| TXNL1 | -0.390 | Downregulated | 4.33E-02 | 6.11E-01 |
| PNPT1 | -0.386 | Downregulated | 2.96E-03 | 3.58E-01 |
| KBTBD7 | -0.381 | Downregulated | 1.77E-02 | 5.26E-01 |
| ADI1 | -0.376 | Downregulated | 4.90E-02 | 6.27E-01 |
| RPS28 | -0.376 | Downregulated | 1.87E-02 | 5.37E-01 |
| SFRS11 | -0.373 | Downregulated | 2.49E-02 | 5.71E-01 |
| IFP38 | -0.373 | Downregulated | 1.95E-02 | 5.39E-01 |
| LRRC6 | -0.367 | Downregulated | 3.02E-02 | 5.83E-01 |
| USP49 | -0.364 | Downregulated | 2.26E-02 | 5.59E-01 |
| MBNL2 | -0.363 | Downregulated | 3.12E-02 | 5.86E-01 |
| HMGB1L1 | -0.362 | Downregulated | 4.23E-02 | 6.08E-01 |
| VPS26 | -0.362 | Downregulated | 2.97E-02 | 5.81E-01 |
| HRK | -0.361 | Downregulated | 3.41E-02 | 5.93E-01 |
| UPLP | -0.360 | Downregulated | 2.17E-03 | 3.36E-01 |
| SLC38A2 | -0.359 | Downregulated | 1.04E-02 | 4.71E-01 |
| PRIM2 | -0.357 | Downregulated | 4.64E-03 | 4.13E-01 |
| APOBEC3A | -0.353 | Downregulated | 4.59E-02 | 6.16E-01 |
| CEP27 | -0.352 | Downregulated | 6.98E-03 | 4.57E-01 |

| | | | | |
|---|---|---|---|---|
| EVI5 | -0.350 | Downregulated | 5.04E-03 | 4.30E-01 |
| EIF3A | -0.349 | Downregulated | 1.15E-02 | 4.89E-01 |
| BLZF1 | -0.347 | Downregulated | 4.19E-03 | 4.00E-01 |
| ALS2CR14 | -0.345 | Downregulated | 3.12E-03 | 3.58E-01 |
| FAM129C | -0.342 | Downregulated | 4.53E-02 | 6.14E-01 |
| RAXL1 | -0.340 | Downregulated | 5.76E-03 | 4.39E-01 |
| NIPSNAP3B | -0.340 | Downregulated | 1.43E-03 | 3.13E-01 |
| SH3YL1 | -0.337 | Downregulated | 9.12E-03 | 4.63E-01 |
| CAT | -0.337 | Downregulated | 1.03E-02 | 4.69E-01 |
| MINPP1 | -0.334 | Downregulated | 1.42E-02 | 5.04E-01 |
| ZNF860 | -0.330 | Downregulated | 1.11E-02 | 4.87E-01 |
| SLC5A8 | -0.329 | Downregulated | 3.50E-02 | 5.96E-01 |
| MYB | -0.322 | Downregulated | 1.01E-02 | 4.68E-01 |
| SBDS | -0.321 | Downregulated | 3.95E-02 | 6.03E-01 |
| MBD4 | -0.320 | Downregulated | 1.11E-02 | 4.87E-01 |
| ALDH5A1 | -0.317 | Downregulated | 3.75E-02 | 6.02E-01 |
| FAM98A | -0.316 | Downregulated | 3.70E-02 | 5.99E-01 |
| POTEE | -0.316 | Downregulated | 1.80E-02 | 5.26E-01 |
| FAM153B | -0.314 | Downregulated | 2.41E-02 | 5.71E-01 |
| DCTN4 | -0.314 | Downregulated | 1.28E-02 | 4.97E-01 |
| FAM175A | -0.312 | Downregulated | 2.80E-02 | 5.76E-01 |
| ZNF600 | -0.311 | Downregulated | 9.54E-03 | 4.63E-01 |
| FLJ44124 | -0.310 | Downregulated | 9.03E-03 | 4.63E-01 |
| RAX2 | -0.309 | Downregulated | 3.88E-02 | 6.02E-01 |
| EIF2AK4 | -0.308 | Downregulated | 2.22E-02 | 5.55E-01 |
| PRG3 | -0.307 | Downregulated | 3.58E-02 | 5.97E-01 |
| TAPT1 | -0.307 | Downregulated | 1.23E-03 | 2.85E-01 |
| NXT2 | -0.306 | Downregulated | 2.94E-02 | 5.81E-01 |
| ANP32A | -0.303 | Downregulated | 3.91E-02 | 6.03E-01 |
| PPM1B | -0.301 | Downregulated | 1.22E-02 | 4.93E-01 |
| ANKRD30B | -0.298 | Downregulated | 4.02E-03 | 3.94E-01 |
| JMJD1A | -0.298 | Downregulated | 1.29E-02 | 4.97E-01 |
| RHBDL2 | -0.297 | Downregulated | 9.38E-03 | 4.63E-01 |
| CUL4A | -0.297 | Downregulated | 1.69E-02 | 5.25E-01 |
| HSD17B7 | -0.295 | Downregulated | 7.08E-03 | 4.57E-01 |
| SERPINB2 | -0.295 | Downregulated | 1.64E-02 | 5.23E-01 |
| RAB39B | -0.294 | Downregulated | 8.34E-03 | 4.63E-01 |
| SFRS12 | -0.294 | Downregulated | 1.25E-02 | 4.95E-01 |
| PEX3 | -0.293 | Downregulated | 7.69E-04 | 2.54E-01 |
| TARS | -0.293 | Downregulated | 3.80E-03 | 3.90E-01 |
| GALNT3 | -0.291 | Downregulated | 2.30E-02 | 5.59E-01 |
| ATF1 | -0.290 | Downregulated | 1.11E-02 | 4.87E-01 |
| HMGB1 | -0.289 | Downregulated | 2.86E-02 | 5.81E-01 |
| MEX3C | -0.288 | Downregulated | 2.53E-02 | 5.71E-01 |
| ST13 | -0.287 | Downregulated | 1.64E-02 | 5.23E-01 |
| ALPP | -0.287 | Downregulated | 1.10E-02 | 4.86E-01 |
| B3GNT5 | -0.286 | Downregulated | 1.84E-02 | 5.31E-01 |
| G3BP1 | -0.286 | Downregulated | 4.34E-02 | 6.11E-01 |
| FLI1 | -0.286 | Downregulated | 3.33E-02 | 5.91E-01 |
| PKN2 | -0.284 | Downregulated | 4.81E-02 | 6.25E-01 |
| BUB3 | -0.284 | Downregulated | 2.69E-02 | 5.75E-01 |

| | | | | |
|---|---|---|---|---|
| UGP2 | -0.282 | Downregulated | 3.60E-02 | 5.97E-01 |
| Septin 11 | -0.280 | Downregulated | 2.00E-02 | 5.42E-01 |
| PRO1853 | -0.279 | Downregulated | 4.95E-02 | 6.27E-01 |
| FSTL3 | -0.279 | Downregulated | 3.88E-03 | 3.93E-01 |
| ZNF420 | -0.278 | Downregulated | 2.47E-03 | 3.48E-01 |
| NDUFB9 | -0.275 | Downregulated | 3.57E-02 | 5.97E-01 |
| GPX8 | -0.273 | Downregulated | 2.81E-02 | 5.78E-01 |
| HSPC268 | -0.272 | Downregulated | 1.80E-02 | 5.26E-01 |
| NLRP8 | -0.271 | Downregulated | 3.46E-03 | 3.72E-01 |
| KLHL24 | -0.271 | Downregulated | 1.38E-02 | 4.99E-01 |
| MGC42367 | -0.270 | Downregulated | 3.17E-02 | 5.86E-01 |
| ADIPOQ | -0.270 | Downregulated | 1.35E-04 | 1.57E-01 |
| PPP1R14A | -0.269 | Downregulated | 2.46E-02 | 5.71E-01 |
| HLA-DMA | 0.265 | Upregulated | 2.16E-02 | 5.51E-01 |
| ATP1B1 | 0.266 | Upregulated | 1.56E-04 | 1.57E-01 |
| BAIAP2L1 | 0.266 | Upregulated | 1.05E-02 | 4.74E-01 |
| GGT1 | 0.267 | Upregulated | 2.14E-02 | 5.51E-01 |
| NPNT | 0.268 | Upregulated | 4.43E-04 | 2.08E-01 |
| CYP2C9 | 0.268 | Upregulated | 1.05E-02 | 4.73E-01 |
| LRRC25 | 0.269 | Upregulated | 2.76E-02 | 5.76E-01 |
| GHRL | 0.270 | Upregulated | 4.53E-02 | 6.14E-01 |
| RILPL2 | 0.270 | Upregulated | 2.74E-03 | 3.58E-01 |
| OR2K2 | 0.271 | Upregulated | 3.17E-02 | 5.86E-01 |
| RNF19A | 0.272 | Upregulated | 1.33E-02 | 4.98E-01 |
| NOD2 | 0.273 | Upregulated | 4.30E-02 | 6.09E-01 |
| FFAR3 | 0.275 | Upregulated | 9.42E-03 | 4.63E-01 |
| BATF3 | 0.277 | Upregulated | 7.58E-03 | 4.61E-01 |
| ADAP2 | 0.277 | Upregulated | 9.61E-03 | 4.64E-01 |
| RNF13 | 0.277 | Upregulated | 6.19E-03 | 4.48E-01 |
| OR9Q1 | 0.278 | Upregulated | 1.17E-03 | 2.83E-01 |
| ASCL2 | 0.278 | Upregulated | 2.29E-02 | 5.59E-01 |
| P76 | 0.282 | Upregulated | 1.75E-03 | 3.17E-01 |
| POLB | 0.282 | Upregulated | 4.70E-04 | 2.08E-01 |
| WDR23 | 0.282 | Upregulated | 2.65E-02 | 5.71E-01 |
| CBR1 | 0.283 | Upregulated | 7.11E-03 | 4.57E-01 |
| MAFB | 0.283 | Upregulated | 2.92E-02 | 5.81E-01 |
| HSPC159 | 0.283 | Upregulated | 1.77E-02 | 5.26E-01 |
| NUDT16 | 0.284 | Upregulated | 6.70E-03 | 4.56E-01 |
| TCHHL1 | 0.287 | Upregulated | 8.40E-03 | 4.63E-01 |
| PCSK6 | 0.288 | Upregulated | 4.50E-05 | 9.19E-02 |
| SORT1 | 0.288 | Upregulated | 2.86E-02 | 5.81E-01 |
| ITGB5 | 0.289 | Upregulated | 3.19E-02 | 5.86E-01 |
| CD151 | 0.289 | Upregulated | 1.11E-02 | 4.87E-01 |
| TMEM149 | 0.290 | Upregulated | 1.92E-03 | 3.26E-01 |
| CDKN2C | 0.291 | Upregulated | 1.41E-04 | 1.57E-01 |
| WDFY1 | 0.291 | Upregulated | 5.28E-04 | 2.16E-01 |
| CD33 | 0.292 | Upregulated | 2.69E-02 | 5.75E-01 |
| TAP2 | 0.294 | Upregulated | 5.90E-03 | 4.44E-01 |
| BMP6 | 0.295 | Upregulated | 3.42E-02 | 5.93E-01 |
| TRAFD1 | 0.295 | Upregulated | 1.15E-03 | 2.83E-01 |
| GADD45B | 0.295 | Upregulated | 6.73E-03 | 4.56E-01 |

| | | | | |
|---|---|---|---|---|
| APOBEC3G | 0.296 | Upregulated | 6.55E-03 | 4.56E-01 |
| SLAMF8 | 0.297 | Upregulated | 2.86E-02 | 5.81E-01 |
| TNFAIP6 | 0.297 | Upregulated | 3.03E-03 | 3.58E-01 |
| FAH | 0.298 | Upregulated | 2.06E-02 | 5.45E-01 |
| DDX58 | 0.299 | Upregulated | 2.75E-02 | 5.76E-01 |
| KCNJ2 | 0.300 | Upregulated | 2.59E-02 | 5.71E-01 |
| BEST1 | 0.302 | Upregulated | 4.68E-02 | 6.20E-01 |
| MAX | 0.302 | Upregulated | 5.52E-03 | 4.38E-01 |
| ODF3B | 0.303 | Upregulated | 5.20E-05 | 9.50E-02 |
| SP100 | 0.303 | Upregulated | 4.01E-02 | 6.03E-01 |
| GGTLC2 | 0.305 | Upregulated | 2.46E-02 | 5.71E-01 |
| TAX1BP3 | 0.305 | Upregulated | 7.68E-04 | 2.54E-01 |
| CMIP | 0.305 | Upregulated | 3.68E-03 | 3.83E-01 |
| PSMB10 | 0.306 | Upregulated | 1.54E-04 | 1.57E-01 |
| HOPX | 0.311 | Upregulated | 6.05E-05 | 1.00E-01 |
| ACTA2 | 0.312 | Upregulated | 3.26E-02 | 5.88E-01 |
| HIST1H1E | 0.314 | Upregulated | 3.07E-02 | 5.84E-01 |
| TMSB15A | 0.316 | Upregulated | 2.38E-03 | 3.44E-01 |
| SUFU | 0.316 | Upregulated | 4.37E-03 | 4.05E-01 |
| LUC7L | 0.318 | Upregulated | 1.61E-04 | 1.57E-01 |
| DDEF2 | 0.318 | Upregulated | 9.38E-04 | 2.67E-01 |
| OBFC1 | 0.320 | Upregulated | 1.57E-04 | 1.57E-01 |
| SCGB1C1 | 0.321 | Upregulated | 3.20E-02 | 5.87E-01 |
| TMEM51 | 0.323 | Upregulated | 3.04E-02 | 5.83E-01 |
| CTSA | 0.323 | Upregulated | 5.53E-03 | 4.38E-01 |
| MR1 | 0.325 | Upregulated | 6.80E-05 | 1.03E-01 |
| RP1L1 | 0.325 | Upregulated | 9.92E-03 | 4.66E-01 |
| ZNF185 | 0.326 | Upregulated | 1.48E-02 | 5.07E-01 |
| PGRMC1 | 0.327 | Upregulated | 6.18E-03 | 4.48E-01 |
| PTPRC | 0.327 | Upregulated | 3.16E-02 | 5.86E-01 |
| SNX20 | 0.328 | Upregulated | 1.08E-03 | 2.83E-01 |
| PARP14 | 0.329 | Upregulated | 2.57E-02 | 5.71E-01 |
| SPATA20 | 0.330 | Upregulated | 2.18E-02 | 5.52E-01 |
| IFITM1 | 0.330 | Upregulated | 2.51E-03 | 3.48E-01 |
| PAK2 | 0.330 | Upregulated | 5.51E-03 | 4.38E-01 |
| IL15 | 0.331 | Upregulated | 4.62E-04 | 2.08E-01 |
| SAMD9L | 0.332 | Upregulated | 4.55E-02 | 6.14E-01 |
| PSMB9 | 0.332 | Upregulated | 1.40E-03 | 3.08E-01 |
| GALM | 0.334 | Upregulated | 4.50E-02 | 6.14E-01 |
| TNFSF10 | 0.334 | Upregulated | 2.43E-02 | 5.71E-01 |
| GRAMD1B | 0.337 | Upregulated | 1.27E-02 | 4.96E-01 |
| CABP5 | 0.337 | Upregulated | 2.17E-02 | 5.51E-01 |
| ACP2 | 0.338 | Upregulated | 8.37E-04 | 2.62E-01 |
| IFIH1 | 0.339 | Upregulated | 3.72E-02 | 6.02E-01 |
| GK | 0.340 | Upregulated | 2.70E-02 | 5.75E-01 |
| IRF1 | 0.340 | Upregulated | 4.93E-04 | 2.08E-01 |
| SPARC | 0.340 | Upregulated | 2.08E-02 | 5.45E-01 |
| RHOU | 0.341 | Upregulated | 1.90E-03 | 3.26E-01 |
| FRMD3 | 0.342 | Upregulated | 1.91E-02 | 5.39E-01 |
| CD3G | 0.343 | Upregulated | 4.79E-02 | 6.24E-01 |
| JAM3 | 0.343 | Upregulated | 1.30E-02 | 4.97E-01 |

| | | | | |
|---|---|---|---|---|
| ACOX1 | 0.343 | Upregulated | 1.34E-02 | 4.98E-01 |
| SCO2 | 0.343 | Upregulated | 1.43E-02 | 5.04E-01 |
| ALOX12 | 0.344 | Upregulated | 3.88E-02 | 6.02E-01 |
| ABLIM3 | 0.345 | Upregulated | 4.06E-03 | 3.95E-01 |
| TNNC2 | 0.347 | Upregulated | 6.25E-03 | 4.48E-01 |
| FGL2 | 0.347 | Upregulated | 5.77E-03 | 4.39E-01 |
| ETV7 | 0.349 | Upregulated | 6.86E-03 | 4.56E-01 |
| GPR42 | 0.350 | Upregulated | 5.64E-03 | 4.38E-01 |
| KIFC3 | 0.351 | Upregulated | 1.23E-02 | 4.94E-01 |
| CCR1 | 0.354 | Upregulated | 4.84E-02 | 6.26E-01 |
| EPB41L3 | 0.356 | Upregulated | 2.25E-02 | 5.58E-01 |
| ITGB3 | 0.358 | Upregulated | 4.84E-02 | 6.26E-01 |
| ARHGEF10L | 0.361 | Upregulated | 4.12E-05 | 9.19E-02 |
| RARRES3 | 0.365 | Upregulated | 3.84E-03 | 3.90E-01 |
| SOCS1 | 0.365 | Upregulated | 8.26E-03 | 4.63E-01 |
| EIF3CL | 0.369 | Upregulated | 9.98E-03 | 4.66E-01 |
| VAMP5 | 0.370 | Upregulated | 5.20E-04 | 2.15E-01 |
| IL1B | 0.372 | Upregulated | 4.39E-02 | 6.13E-01 |
| PLAUR | 0.372 | Upregulated | 7.83E-03 | 4.63E-01 |
| HIST1H2BJ | 0.373 | Upregulated | 3.20E-02 | 5.87E-01 |
| SLC6A12 | 0.374 | Upregulated | 3.24E-03 | 3.64E-01 |
| ANKDD1A | 0.375 | Upregulated | 2.64E-03 | 3.53E-01 |
| ITPRIPL2 | 0.376 | Upregulated | 2.91E-03 | 3.58E-01 |
| TAP1 | 0.378 | Upregulated | 6.75E-03 | 4.56E-01 |
| KLHDC8B | 0.379 | Upregulated | 4.23E-02 | 6.07E-01 |
| DDX60 | 0.380 | Upregulated | 4.32E-02 | 6.11E-01 |
| FSTL1 | 0.382 | Upregulated | 8.77E-03 | 4.63E-01 |
| PROS1 | 0.384 | Upregulated | 3.11E-02 | 5.86E-01 |
| IL12RB1 | 0.386 | Upregulated | 3.67E-03 | 3.83E-01 |
| APOL1 | 0.387 | Upregulated | 6.64E-04 | 2.42E-01 |
| MT1A | 0.388 | Upregulated | 2.74E-02 | 5.75E-01 |
| IFI35 | 0.388 | Upregulated | 6.62E-03 | 4.56E-01 |
| GADD45G | 0.390 | Upregulated | 2.57E-03 | 3.52E-01 |
| IFITM3 | 0.391 | Upregulated | 1.53E-02 | 5.14E-01 |
| NDUFAF3 | 0.391 | Upregulated | 7.16E-03 | 4.57E-01 |
| IFIT5 | 0.392 | Upregulated | 1.38E-02 | 4.99E-01 |
| GPBAR1 | 0.393 | Upregulated | 1.27E-02 | 4.96E-01 |
| TYMP | 0.393 | Upregulated | 1.75E-03 | 3.17E-01 |
| FLJ20309 | 0.398 | Upregulated | 2.53E-02 | 5.71E-01 |
| ECGF1 | 0.401 | Upregulated | 4.25E-04 | 2.05E-01 |
| IL27 | 0.401 | Upregulated | 1.82E-02 | 5.28E-01 |
| SDPR | 0.403 | Upregulated | 3.43E-02 | 5.94E-01 |
| ESAM | 0.403 | Upregulated | 3.39E-02 | 5.93E-01 |
| SAMD4A | 0.404 | Upregulated | 1.91E-02 | 5.39E-01 |
| PSME2 | 0.404 | Upregulated | 4.99E-06 | 2.34E-02 |
| SMARCD3 | 0.404 | Upregulated | 9.84E-03 | 4.66E-01 |
| CXCL9 | 0.406 | Upregulated | 8.21E-03 | 4.63E-01 |
| MMRN1 | 0.406 | Upregulated | 2.99E-02 | 5.81E-01 |
| FAM21A | 0.407 | Upregulated | 3.84E-02 | 6.02E-01 |
| CMTM5 | 0.408 | Upregulated | 1.43E-02 | 5.05E-01 |
| TMEM140 | 0.410 | Upregulated | 1.54E-02 | 5.15E-01 |

| | | | | |
|---|---|---|---|---|
| GBP2 | 0.411 | Upregulated | 2.43E-05 | 7.41E-02 |
| IRX3 | 0.414 | Upregulated | 5.67E-03 | 4.38E-01 |
| STAT1 | 0.414 | Upregulated | 1.47E-03 | 3.14E-01 |
| TSPAN9 | 0.415 | Upregulated | 2.21E-02 | 5.55E-01 |
| ACRBP | 0.416 | Upregulated | 2.89E-02 | 5.81E-01 |
| GP6 | 0.418 | Upregulated | 5.68E-04 | 2.19E-01 |
| MYL9 | 0.419 | Upregulated | 3.37E-02 | 5.93E-01 |
| CLDN5 | 0.443 | Upregulated | 2.91E-02 | 5.81E-01 |
| LY6E | 0.444 | Upregulated | 3.50E-02 | 5.96E-01 |
| TFPI | 0.445 | Upregulated | 5.99E-03 | 4.44E-01 |
| GCH1 | 0.450 | Upregulated | 3.13E-03 | 3.58E-01 |
| RBAK | 0.451 | Upregulated | 3.25E-02 | 5.88E-01 |
| TRIM22 | 0.458 | Upregulated | 2.04E-02 | 5.45E-01 |
| OASL | 0.462 | Upregulated | 4.76E-02 | 6.23E-01 |
| MYOF | 0.470 | Upregulated | 8.35E-03 | 4.63E-01 |
| FCGR1C | 0.470 | Upregulated | 9.76E-03 | 4.66E-01 |
| SERPING1 | 0.474 | Upregulated | 2.66E-03 | 3.53E-01 |
| TIMM10 | 0.474 | Upregulated | 1.30E-02 | 4.97E-01 |
| LGALS3BP | 0.480 | Upregulated | 2.72E-02 | 5.75E-01 |
| TREML1 | 0.481 | Upregulated | 2.74E-02 | 5.75E-01 |
| C1QA | 0.490 | Upregulated | 1.68E-04 | 1.57E-01 |
| C2 | 0.492 | Upregulated | 3.10E-04 | 1.91E-01 |
| FBXO6 | 0.493 | Upregulated | 6.71E-04 | 2.43E-01 |
| RTP4 | 0.497 | Upregulated | 1.13E-02 | 4.87E-01 |
| UBE2L6 | 0.498 | Upregulated | 1.55E-03 | 3.16E-01 |
| MT2A | 0.510 | Upregulated | 9.06E-03 | 4.63E-01 |
| GBP6 | 0.512 | Upregulated | 4.71E-06 | 2.34E-02 |
| TMEM158 | 0.520 | Upregulated | 2.02E-02 | 5.45E-01 |
| EPSTI1 | 0.534 | Upregulated | 6.90E-03 | 4.56E-01 |
| FER1L3 | 0.535 | Upregulated | 1.11E-03 | 2.83E-01 |
| PVALB | 0.539 | Upregulated | 6.87E-03 | 4.56E-01 |
| CASP5 | 0.539 | Upregulated | 3.05E-02 | 5.83E-01 |
| LAP3 | 0.548 | Upregulated | 5.45E-04 | 2.19E-01 |
| SOD2 | 0.555 | Upregulated | 4.10E-04 | 2.03E-01 |
| APOL6 | 0.557 | Upregulated | 1.41E-05 | 5.44E-02 |
| GP9 | 0.571 | Upregulated | 8.10E-03 | 4.63E-01 |
| CD226 | 0.574 | Upregulated | 1.01E-03 | 2.80E-01 |
| CXCL10 | 0.575 | Upregulated | 9.24E-03 | 4.63E-01 |
| GBP4 | 0.577 | Upregulated | 9.76E-04 | 2.75E-01 |
| WARS | 0.582 | Upregulated | 1.53E-06 | 2.34E-02 |
| FCGR1B | 0.593 | Upregulated | 1.82E-03 | 3.21E-01 |
| IFIT2 | 0.598 | Upregulated | 1.03E-02 | 4.69E-01 |
| ITGA2B | 0.604 | Upregulated | 1.45E-02 | 5.05E-01 |
| FCGR1A | 0.616 | Upregulated | 1.20E-03 | 2.84E-01 |
| CD274 | 0.632 | Upregulated | 4.21E-04 | 2.05E-01 |
| C1QC | 0.677 | Upregulated | 3.12E-03 | 3.58E-01 |
| ANKRD22 | 0.691 | Upregulated | 2.12E-04 | 1.67E-01 |
| PDCD1LG2 | 0.725 | Upregulated | 7.54E-07 | 2.34E-02 |
| GBP1 | 0.735 | Upregulated | 3.69E-04 | 2.00E-01 |
| IFIT3 | 0.751 | Upregulated | 1.66E-02 | 5.23E-01 |
| BATF2 | 0.850 | Upregulated | 5.93E-05 | 1.00E-01 |

| | | | | |
|---|---|---|---|---|
| GBP5 | 0.966 | Upregulated | 2.39E-06 | 2.34E-02 |
| C1QB | 0.995 | Upregulated | 6.85E-04 | 2.45E-01 |

**Supplementary Table S3d. Differentially expressed genes_Kenya_Female**

| Gene | log FoldChange | Direction of expression | P.Value | adj.P.Val |
|---|---|---|---|---|
| GYPA | -0.858 | Downregulated | 4.74E-02 | 3.18E-01 |
| GZMK | -0.795 | Downregulated | 1.17E-03 | 3.68E-02 |
| LRRN3 | -0.699 | Downregulated | 1.77E-02 | 1.90E-01 |
| RPL23 | -0.686 | Downregulated | 6.45E-03 | 1.04E-01 |
| NELL2 | -0.685 | Downregulated | 1.18E-03 | 3.69E-02 |
| RPL14 | -0.676 | Downregulated | 7.82E-03 | 1.17E-01 |
| CD3D | -0.673 | Downregulated | 2.17E-04 | 1.22E-02 |
| RPS23 | -0.669 | Downregulated | 4.64E-02 | 3.15E-01 |
| GAL | -0.662 | Downregulated | 1.42E-02 | 1.68E-01 |
| RASGRP1 | -0.648 | Downregulated | 5.83E-04 | 2.27E-02 |
| CLC | -0.633 | Downregulated | 3.11E-02 | 2.57E-01 |
| KIAA0101 | -0.619 | Downregulated | 1.91E-02 | 1.98E-01 |
| MCOLN2 | -0.606 | Downregulated | 2.07E-03 | 5.29E-02 |
| SH2D1A | -0.594 | Downregulated | 1.24E-03 | 3.82E-02 |
| TC2N | -0.590 | Downregulated | 1.61E-03 | 4.53E-02 |
| CD27 | -0.581 | Downregulated | 1.13E-02 | 1.47E-01 |
| IFNG | -0.569 | Downregulated | 2.19E-02 | 2.14E-01 |
| GYPE | -0.566 | Downregulated | 4.77E-02 | 3.19E-01 |
| CD8A | -0.565 | Downregulated | 1.41E-02 | 1.67E-01 |
| PVRIG | -0.552 | Downregulated | 5.27E-03 | 9.23E-02 |
| RPL7 | -0.545 | Downregulated | 6.40E-03 | 1.04E-01 |
| TSPAN7 | -0.541 | Downregulated | 4.32E-02 | 3.04E-01 |
| CENPK | -0.538 | Downregulated | 1.12E-02 | 1.46E-01 |
| OCIAD2 | -0.536 | Downregulated | 1.05E-03 | 3.45E-02 |
| TARP | -0.536 | Downregulated | 8.69E-03 | 1.26E-01 |
| GZMA | -0.528 | Downregulated | 9.09E-03 | 1.30E-01 |
| TOP2A | -0.527 | Downregulated | 3.15E-02 | 2.60E-01 |
| CHST12 | -0.527 | Downregulated | 3.61E-03 | 7.38E-02 |
| SKAP1 | -0.527 | Downregulated | 9.50E-04 | 3.21E-02 |
| MS4A1 | -0.526 | Downregulated | 1.25E-02 | 1.56E-01 |
| LAIR2 | -0.520 | Downregulated | 3.33E-02 | 2.67E-01 |
| ETS1 | -0.518 | Downregulated | 1.71E-03 | 4.68E-02 |
| TYMS | -0.517 | Downregulated | 1.33E-02 | 1.63E-01 |
| RPL26 | -0.512 | Downregulated | 1.21E-02 | 1.54E-01 |
| PARM1 | -0.511 | Downregulated | 4.24E-02 | 3.01E-01 |
| EBI2 | -0.508 | Downregulated | 1.77E-02 | 1.90E-01 |
| TXNDC12 | -0.503 | Downregulated | 5.98E-05 | 5.70E-03 |
| GZMM | -0.499 | Downregulated | 5.66E-03 | 9.63E-02 |
| TIMD4 | -0.499 | Downregulated | 2.65E-02 | 2.36E-01 |
| CD2 | -0.498 | Downregulated | 1.69E-03 | 4.65E-02 |
| HSD17B8 | -0.497 | Downregulated | 4.38E-04 | 1.89E-02 |
| LDHB | -0.495 | Downregulated | 1.26E-03 | 3.86E-02 |
| CXCR3 | -0.489 | Downregulated | 2.77E-03 | 6.37E-02 |
| GPR18 | -0.489 | Downregulated | 3.17E-03 | 6.88E-02 |
| SPOCK2 | -0.487 | Downregulated | 3.23E-03 | 6.94E-02 |
| CD52 | -0.485 | Downregulated | 2.27E-03 | 5.62E-02 |
| SLC25A23 | -0.484 | Downregulated | 2.96E-04 | 1.46E-02 |
| FAIM3 | -0.483 | Downregulated | 1.70E-02 | 1.86E-01 |

| | | | | |
|---|---|---|---|---|
| SP4 | -0.481 | Downregulated | 1.90E-04 | 1.12E-02 |
| EOMES | -0.481 | Downregulated | 1.48E-02 | 1.72E-01 |
| TMEM204 | -0.480 | Downregulated | 6.24E-03 | 1.02E-01 |
| PTPRCAP | -0.479 | Downregulated | 5.48E-03 | 9.44E-02 |
| CD6 | -0.477 | Downregulated | 5.51E-03 | 9.46E-02 |
| ITK | -0.475 | Downregulated | 2.53E-03 | 6.03E-02 |
| CD96 | -0.474 | Downregulated | 1.81E-03 | 4.86E-02 |
| NOC3L | -0.473 | Downregulated | 1.45E-02 | 1.70E-01 |
| KLHL3 | -0.470 | Downregulated | 2.87E-02 | 2.48E-01 |
| KLRD1 | -0.469 | Downregulated | 1.18E-02 | 1.51E-01 |
| TBC1D4 | -0.467 | Downregulated | 6.15E-03 | 1.02E-01 |
| HNRNPH1 | -0.465 | Downregulated | 3.18E-03 | 6.90E-02 |
| RPL5 | -0.462 | Downregulated | 2.81E-03 | 6.43E-02 |
| GPR114 | -0.461 | Downregulated | 1.57E-02 | 1.77E-01 |
| PASK | -0.459 | Downregulated | 2.61E-03 | 6.13E-02 |
| HDDC2 | -0.459 | Downregulated | 1.61E-03 | 4.53E-02 |
| PYHIN1 | -0.458 | Downregulated | 2.16E-02 | 2.13E-01 |
| ABCB10 | -0.457 | Downregulated | 2.43E-03 | 5.89E-02 |
| TRIB2 | -0.456 | Downregulated | 9.56E-03 | 1.34E-01 |
| E2F2 | -0.456 | Downregulated | 1.82E-02 | 1.92E-01 |
| STMN3 | -0.455 | Downregulated | 2.55E-03 | 6.06E-02 |
| PBK | -0.455 | Downregulated | 2.97E-02 | 2.52E-01 |
| ASPM | -0.454 | Downregulated | 3.77E-02 | 2.86E-01 |
| COX6C | -0.452 | Downregulated | 3.16E-03 | 6.87E-02 |
| SMC6 | -0.451 | Downregulated | 2.96E-02 | 2.52E-01 |
| SSPN | -0.450 | Downregulated | 3.11E-02 | 2.57E-01 |
| RFX7 | -0.449 | Downregulated | 2.54E-04 | 1.33E-02 |
| LIME1 | -0.449 | Downregulated | 9.80E-03 | 1.36E-01 |
| RPS18 | -0.449 | Downregulated | 1.82E-03 | 4.87E-02 |
| MRPL40 | -0.447 | Downregulated | 3.77E-02 | 2.86E-01 |
| TGFBR3 | -0.446 | Downregulated | 2.68E-02 | 2.38E-01 |
| SLAMF6 | -0.446 | Downregulated | 3.53E-03 | 7.30E-02 |
| ZBED2 | -0.444 | Downregulated | 4.61E-02 | 3.14E-01 |
| LYPD3 | -0.442 | Downregulated | 4.51E-02 | 3.11E-01 |
| PCNT | -0.441 | Downregulated | 9.61E-04 | 3.23E-02 |
| CCDC14 | -0.441 | Downregulated | 4.44E-04 | 1.91E-02 |
| ITGB3BP | -0.441 | Downregulated | 6.24E-03 | 1.02E-01 |
| MAP4K1 | -0.440 | Downregulated | 1.65E-02 | 1.82E-01 |
| RPS28 | -0.437 | Downregulated | 8.27E-03 | 1.22E-01 |
| PCMTD2 | -0.436 | Downregulated | 1.97E-03 | 5.13E-02 |
| PEBP1 | -0.436 | Downregulated | 8.62E-04 | 2.99E-02 |
| LAX1 | -0.436 | Downregulated | 4.83E-03 | 8.85E-02 |
| PTPN4 | -0.435 | Downregulated | 3.03E-03 | 6.75E-02 |
| KLRB1 | -0.433 | Downregulated | 1.35E-02 | 1.64E-01 |
| LZTFL1 | -0.432 | Downregulated | 1.68E-02 | 1.85E-01 |
| STAMBPL1 | -0.431 | Downregulated | 1.09E-02 | 1.45E-01 |
| ITPR3 | -0.431 | Downregulated | 2.06E-03 | 5.28E-02 |
| SNRNP70 | -0.431 | Downregulated | 3.48E-02 | 2.73E-01 |
| BCAS4 | -0.430 | Downregulated | 8.52E-04 | 2.98E-02 |
| KIF20B | -0.429 | Downregulated | 1.62E-02 | 1.81E-01 |
| TMEM107 | -0.427 | Downregulated | 2.07E-02 | 2.07E-01 |

| | | | | |
|---|---|---|---|---|
| ZNF23 | -0.427 | Downregulated | 2.06E-03 | 5.28E-02 |
| STK39 | -0.426 | Downregulated | 3.08E-03 | 6.78E-02 |
| RPL21 | -0.426 | Downregulated | 4.30E-03 | 8.26E-02 |
| CTSW | -0.425 | Downregulated | 4.22E-02 | 3.01E-01 |
| SEC11C | -0.425 | Downregulated | 1.04E-02 | 1.41E-01 |
| CEP78 | -0.423 | Downregulated | 1.59E-02 | 1.79E-01 |
| LY9 | -0.422 | Downregulated | 1.65E-03 | 4.60E-02 |
| CD3E | -0.422 | Downregulated | 3.94E-03 | 7.78E-02 |
| SEL1L3 | -0.421 | Downregulated | 2.57E-03 | 6.08E-02 |
| RPS3A | -0.421 | Downregulated | 1.02E-02 | 1.39E-01 |
| TFRC | -0.420 | Downregulated | 2.63E-03 | 6.17E-02 |
| CD244 | -0.420 | Downregulated | 1.16E-03 | 3.68E-02 |
| ITGB7 | -0.420 | Downregulated | 3.67E-03 | 7.42E-02 |
| PRKCH | -0.420 | Downregulated | 7.61E-03 | 1.16E-01 |
| MSC | -0.420 | Downregulated | 6.98E-03 | 1.09E-01 |
| SH3YL1 | -0.419 | Downregulated | 1.69E-03 | 4.66E-02 |
| DTL | -0.419 | Downregulated | 1.15E-02 | 1.49E-01 |
| PRSS23 | -0.417 | Downregulated | 3.64E-02 | 2.81E-01 |
| ICOS | -0.416 | Downregulated | 1.73E-02 | 1.87E-01 |
| RPA1 | -0.416 | Downregulated | 2.48E-04 | 1.32E-02 |
| PARP15 | -0.416 | Downregulated | 1.21E-02 | 1.54E-01 |
| CELSR3 | -0.415 | Downregulated | 4.22E-02 | 3.01E-01 |
| KIAA0831 | -0.415 | Downregulated | 1.21E-03 | 3.76E-02 |
| MYB | -0.414 | Downregulated | 2.46E-04 | 1.32E-02 |
| SLC4A7 | -0.414 | Downregulated | 3.09E-03 | 6.79E-02 |
| RAP2A | -0.413 | Downregulated | 7.41E-04 | 2.70E-02 |
| ESYT1 | -0.413 | Downregulated | 3.24E-03 | 6.94E-02 |
| RPL31 | -0.412 | Downregulated | 2.97E-02 | 2.52E-01 |
| UROS | -0.411 | Downregulated | 3.73E-04 | 1.70E-02 |
| TK1 | -0.410 | Downregulated | 2.30E-02 | 2.19E-01 |
| CD7 | -0.409 | Downregulated | 1.70E-02 | 1.86E-01 |
| RPL17 | -0.409 | Downregulated | 1.79E-02 | 1.91E-01 |
| MRPL1 | -0.408 | Downregulated | 2.66E-02 | 2.37E-01 |
| S1PR1 | -0.407 | Downregulated | 3.82E-03 | 7.63E-02 |
| RAD51AP1 | -0.405 | Downregulated | 8.13E-03 | 1.20E-01 |
| CACNA2D3 | -0.404 | Downregulated | 3.38E-02 | 2.70E-01 |
| CMPK1 | -0.403 | Downregulated | 5.51E-03 | 9.46E-02 |
| AKR1B1 | -0.403 | Downregulated | 3.22E-03 | 6.94E-02 |
| STAT4 | -0.402 | Downregulated | 2.57E-03 | 6.08E-02 |
| ASF1A | -0.401 | Downregulated | 3.52E-04 | 1.64E-02 |
| IRF4 | -0.400 | Downregulated | 4.91E-03 | 8.94E-02 |
| ECHDC2 | -0.400 | Downregulated | 1.97E-03 | 5.13E-02 |
| BHLHB3 | -0.400 | Downregulated | 1.32E-02 | 1.62E-01 |
| MDC1 | -0.399 | Downregulated | 2.67E-04 | 1.38E-02 |
| LANCL1 | -0.397 | Downregulated | 5.27E-04 | 2.13E-02 |
| NMT2 | -0.397 | Downregulated | 2.42E-02 | 2.25E-01 |
| CCND2 | -0.397 | Downregulated | 4.25E-03 | 8.18E-02 |
| VPS36 | -0.396 | Downregulated | 2.76E-03 | 6.36E-02 |
| ZNF529 | -0.396 | Downregulated | 2.17E-04 | 1.22E-02 |
| FAM113B | -0.395 | Downregulated | 3.07E-03 | 6.78E-02 |
| PTTG1 | -0.395 | Downregulated | 4.99E-03 | 9.03E-02 |

| | | | | |
|---|---|---|---|---|
| GPA33 | -0.395 | Downregulated | 2.48E-02 | 2.28E-01 |
| ENOSF1 | -0.395 | Downregulated | 2.19E-02 | 2.14E-01 |
| FAM167A | -0.394 | Downregulated | 3.67E-02 | 2.82E-01 |
| RPIA | -0.393 | Downregulated | 3.61E-02 | 2.79E-01 |
| CDC7 | -0.392 | Downregulated | 4.44E-03 | 8.42E-02 |
| COL4A4 | -0.392 | Downregulated | 1.39E-02 | 1.66E-01 |
| MEF2D | -0.391 | Downregulated | 7.35E-05 | 6.47E-03 |
| INPP4B | -0.391 | Downregulated | 8.92E-05 | 7.26E-03 |
| KLRG1 | -0.390 | Downregulated | 4.27E-04 | 1.86E-02 |
| RPS24 | -0.390 | Downregulated | 3.91E-02 | 2.91E-01 |
| TMEM181 | -0.389 | Downregulated | 3.04E-03 | 6.76E-02 |
| NAT6 | -0.389 | Downregulated | 8.39E-03 | 1.23E-01 |
| RAB33A | -0.389 | Downregulated | 2.89E-05 | 3.65E-03 |
| UQCRHL | -0.386 | Downregulated | 3.10E-03 | 6.79E-02 |
| RPS15A | -0.385 | Downregulated | 2.43E-03 | 5.90E-02 |
| IL23A | -0.384 | Downregulated | 1.15E-02 | 1.49E-01 |
| NCALD | -0.383 | Downregulated | 4.74E-02 | 3.18E-01 |
| COX7C | -0.383 | Downregulated | 1.10E-02 | 1.45E-01 |
| BANK1 | -0.382 | Downregulated | 1.53E-02 | 1.74E-01 |
| MTX3 | -0.381 | Downregulated | 4.51E-03 | 8.50E-02 |
| LPAR5 | -0.381 | Downregulated | 4.52E-03 | 8.51E-02 |
| ATP6V0E2 | -0.380 | Downregulated | 7.05E-03 | 1.10E-01 |
| TIGA1 | -0.378 | Downregulated | 4.53E-04 | 1.93E-02 |
| MCM6 | -0.376 | Downregulated | 8.55E-03 | 1.24E-01 |
| PPP3CC | -0.376 | Downregulated | 5.38E-03 | 9.35E-02 |
| HPCAL4 | -0.376 | Downregulated | 1.44E-02 | 1.69E-01 |
| KLF12 | -0.375 | Downregulated | 2.14E-02 | 2.11E-01 |
| RRM1 | -0.375 | Downregulated | 8.07E-03 | 1.20E-01 |
| NOP58 | -0.373 | Downregulated | 5.22E-03 | 9.19E-02 |
| RBX1 | -0.372 | Downregulated | 1.32E-02 | 1.62E-01 |
| TMEM14B | -0.372 | Downregulated | 4.14E-04 | 1.84E-02 |
| CPOX | -0.371 | Downregulated | 2.51E-03 | 6.00E-02 |
| PLEKHA1 | -0.371 | Downregulated | 1.23E-02 | 1.55E-01 |
| CD5 | -0.371 | Downregulated | 3.97E-02 | 2.92E-01 |
| MCM5 | -0.371 | Downregulated | 2.01E-03 | 5.19E-02 |
| NUCKS1 | -0.370 | Downregulated | 2.98E-03 | 6.70E-02 |
| WEE1 | -0.369 | Downregulated | 2.13E-02 | 2.11E-01 |
| Septin 11 | -0.369 | Downregulated | 2.11E-02 | 2.10E-01 |
| UPF3A | -0.368 | Downregulated | 3.16E-04 | 1.53E-02 |
| CUL4A | -0.368 | Downregulated | 1.97E-02 | 2.02E-01 |
| CD247 | -0.368 | Downregulated | 3.83E-02 | 2.88E-01 |
| Septin 6 | -0.368 | Downregulated | 4.87E-03 | 8.88E-02 |
| GTF3A | -0.368 | Downregulated | 4.19E-04 | 1.84E-02 |
| USP12 | -0.368 | Downregulated | 4.71E-02 | 3.17E-01 |
| CD3G | -0.368 | Downregulated | 4.82E-02 | 3.21E-01 |
| ARL4C | -0.367 | Downregulated | 1.56E-03 | 4.47E-02 |
| CYFIP2 | -0.367 | Downregulated | 1.09E-03 | 3.52E-02 |
| SPNS3 | -0.366 | Downregulated | 2.24E-02 | 2.16E-01 |
| TOX | -0.366 | Downregulated | 4.49E-02 | 3.10E-01 |
| MAL | -0.366 | Downregulated | 4.23E-02 | 3.01E-01 |
| WDR54 | -0.365 | Downregulated | 1.40E-02 | 1.67E-01 |

| | | | | |
|---|---|---|---|---|
| CXCR7 | -0.364 | Downregulated | 2.79E-02 | 2.44E-01 |
| IL2RB | -0.364 | Downregulated | 4.25E-02 | 3.01E-01 |
| BAI2 | -0.364 | Downregulated | 4.19E-02 | 3.00E-01 |
| BIN1 | -0.363 | Downregulated | 1.53E-02 | 1.74E-01 |
| ZNF540 | -0.363 | Downregulated | 1.40E-02 | 1.67E-01 |
| ABCE1 | -0.363 | Downregulated | 1.36E-02 | 1.64E-01 |
| SETBP1 | -0.362 | Downregulated | 1.92E-02 | 1.99E-01 |
| ZNF256 | -0.362 | Downregulated | 2.60E-02 | 2.35E-01 |
| GLOD4 | -0.362 | Downregulated | 1.74E-03 | 4.73E-02 |
| MRPL13 | -0.361 | Downregulated | 4.34E-03 | 8.31E-02 |
| CLIP3 | -0.361 | Downregulated | 3.92E-02 | 2.91E-01 |
| RFTN1 | -0.361 | Downregulated | 1.13E-02 | 1.47E-01 |
| TTK | -0.361 | Downregulated | 3.76E-02 | 2.85E-01 |
| TMEM14A | -0.360 | Downregulated | 4.64E-02 | 3.15E-01 |
| HRASLS2 | -0.359 | Downregulated | 2.76E-02 | 2.43E-01 |
| EPHA4 | -0.358 | Downregulated | 2.29E-02 | 2.19E-01 |
| BMI1 | -0.358 | Downregulated | 4.93E-04 | 2.05E-02 |
| MGC3020 | -0.358 | Downregulated | 1.33E-02 | 1.62E-01 |
| GRPEL2 | -0.358 | Downregulated | 8.90E-05 | 7.26E-03 |
| RPS17 | -0.358 | Downregulated | 5.47E-03 | 9.44E-02 |
| SNX25 | -0.357 | Downregulated | 2.14E-02 | 2.11E-01 |
| MXD4 | -0.357 | Downregulated | 9.42E-03 | 1.33E-01 |
| FNBP4 | -0.356 | Downregulated | 3.30E-03 | 7.01E-02 |
| LAMA5 | -0.356 | Downregulated | 4.33E-02 | 3.05E-01 |
| ARHGEF3 | -0.355 | Downregulated | 9.73E-03 | 1.35E-01 |
| KLRC1 | -0.355 | Downregulated | 3.10E-02 | 2.57E-01 |
| LRFN3 | -0.355 | Downregulated | 7.15E-03 | 1.11E-01 |
| TARBP1 | -0.355 | Downregulated | 3.62E-02 | 2.80E-01 |
| ZNF91 | -0.354 | Downregulated | 1.94E-02 | 2.00E-01 |
| FAM62B | -0.354 | Downregulated | 2.58E-03 | 6.08E-02 |
| ST6GALNAC6 | -0.354 | Downregulated | 5.47E-03 | 9.44E-02 |
| BEX2 | -0.354 | Downregulated | 3.29E-02 | 2.65E-01 |
| CIRBP | -0.354 | Downregulated | 2.41E-03 | 5.87E-02 |
| RPL22 | -0.353 | Downregulated | 1.05E-02 | 1.41E-01 |
| CCDC65 | -0.352 | Downregulated | 3.33E-03 | 7.05E-02 |
| RAB40B | -0.352 | Downregulated | 5.20E-03 | 9.17E-02 |
| GFI1 | -0.352 | Downregulated | 2.37E-02 | 2.23E-01 |
| CRYZ | -0.351 | Downregulated | 1.91E-02 | 1.98E-01 |
| SLC27A5 | -0.350 | Downregulated | 5.45E-05 | 5.36E-03 |
| RPL13A | -0.350 | Downregulated | 1.48E-02 | 1.72E-01 |
| ZNF831 | -0.350 | Downregulated | 2.94E-02 | 2.50E-01 |
| UAP1 | -0.349 | Downregulated | 2.12E-02 | 2.10E-01 |
| COCH | -0.348 | Downregulated | 5.12E-03 | 9.12E-02 |
| ZNF827 | -0.348 | Downregulated | 4.03E-02 | 2.95E-01 |
| CDR2 | -0.347 | Downregulated | 1.99E-02 | 2.03E-01 |
| PTPN22 | -0.347 | Downregulated | 3.57E-03 | 7.35E-02 |
| EVL | -0.347 | Downregulated | 1.49E-02 | 1.73E-01 |
| DNAJC9 | -0.347 | Downregulated | 1.08E-02 | 1.44E-01 |
| CD70 | -0.347 | Downregulated | 2.48E-02 | 2.28E-01 |
| TOMM5 | -0.344 | Downregulated | 1.57E-02 | 1.78E-01 |
| RABGGTB | -0.344 | Downregulated | 4.96E-04 | 2.05E-02 |

| | | | | |
|---|---|---|---|---|
| CCDC88C | -0.344 | Downregulated | 3.86E-03 | 7.67E-02 |
| NDUFA4 | -0.344 | Downregulated | 3.32E-03 | 7.05E-02 |
| FBL | -0.344 | Downregulated | 1.27E-02 | 1.58E-01 |
| RPL10A | -0.344 | Downregulated | 1.05E-02 | 1.42E-01 |
| CENPE | -0.343 | Downregulated | 1.05E-02 | 1.42E-01 |
| IFI27L1 | -0.342 | Downregulated | 2.22E-02 | 2.15E-01 |
| RPL15 | -0.341 | Downregulated | 5.45E-03 | 9.42E-02 |
| RFC4 | -0.341 | Downregulated | 2.24E-02 | 2.16E-01 |
| RSL24D1 | -0.341 | Downregulated | 4.24E-02 | 3.01E-01 |
| CD248 | -0.340 | Downregulated | 4.96E-02 | 3.26E-01 |
| DENND2D | -0.339 | Downregulated | 7.66E-03 | 1.16E-01 |
| HSPE1 | -0.339 | Downregulated | 2.45E-02 | 2.27E-01 |
| KLHDC2 | -0.339 | Downregulated | 4.23E-04 | 1.85E-02 |
| CD28 | -0.339 | Downregulated | 3.17E-02 | 2.60E-01 |
| DYRK2 | -0.336 | Downregulated | 7.91E-03 | 1.18E-01 |
| CCT2 | -0.336 | Downregulated | 2.66E-03 | 6.19E-02 |
| HMGN1 | -0.336 | Downregulated | 2.99E-03 | 6.70E-02 |
| ARL5A | -0.336 | Downregulated | 7.85E-03 | 1.18E-01 |
| KIAA1324L | -0.335 | Downregulated | 4.09E-02 | 2.96E-01 |
| CARD11 | -0.335 | Downregulated | 5.55E-03 | 9.51E-02 |
| CAPN12 | -0.334 | Downregulated | 3.26E-02 | 2.64E-01 |
| IL32 | -0.334 | Downregulated | 4.99E-02 | 3.26E-01 |
| AIDA | -0.334 | Downregulated | 1.85E-03 | 4.92E-02 |
| MBNL1 | -0.333 | Downregulated | 3.56E-03 | 7.34E-02 |
| RRAS2 | -0.332 | Downregulated | 3.82E-02 | 2.87E-01 |
| NUSAP1 | -0.332 | Downregulated | 2.87E-02 | 2.48E-01 |
| ZZZ3 | -0.332 | Downregulated | 2.14E-02 | 2.11E-01 |
| EBP | -0.331 | Downregulated | 6.44E-03 | 1.04E-01 |
| SRP72 | -0.331 | Downregulated | 4.71E-03 | 8.73E-02 |
| TRIM2 | -0.331 | Downregulated | 3.13E-02 | 2.59E-01 |
| RPL12 | -0.331 | Downregulated | 6.56E-04 | 2.48E-02 |
| HINT1 | -0.330 | Downregulated | 1.73E-02 | 1.87E-01 |
| REXO2 | -0.330 | Downregulated | 2.64E-02 | 2.36E-01 |
| TRAPPC6A | -0.330 | Downregulated | 2.17E-03 | 5.45E-02 |
| KIF22 | -0.330 | Downregulated | 1.16E-03 | 3.68E-02 |
| BOLA3 | -0.330 | Downregulated | 3.78E-03 | 7.59E-02 |
| RNASEH2B | -0.330 | Downregulated | 3.88E-02 | 2.90E-01 |
| CLEC2D | -0.329 | Downregulated | 3.32E-03 | 7.04E-02 |
| PRC1 | -0.328 | Downregulated | 4.21E-02 | 3.01E-01 |
| MGC72104 | -0.328 | Downregulated | 3.81E-02 | 2.87E-01 |
| GIMAP5 | -0.327 | Downregulated | 1.47E-02 | 1.71E-01 |
| SENP7 | -0.327 | Downregulated | 4.69E-03 | 8.71E-02 |
| ATM | -0.327 | Downregulated | 1.91E-04 | 1.12E-02 |
| MRPL50 | -0.327 | Downregulated | 1.95E-02 | 2.01E-01 |
| RRN3 | -0.327 | Downregulated | 6.77E-03 | 1.07E-01 |
| NUP88 | -0.326 | Downregulated | 2.03E-03 | 5.23E-02 |
| CCNF | -0.326 | Downregulated | 1.79E-02 | 1.91E-01 |
| FAM122B | -0.326 | Downregulated | 8.55E-04 | 2.98E-02 |
| PPP1R16B | -0.325 | Downregulated | 2.38E-02 | 2.24E-01 |
| PIGQ | -0.325 | Downregulated | 4.55E-02 | 3.12E-01 |
| GPR55 | -0.325 | Downregulated | 2.43E-03 | 5.89E-02 |

| | | | | |
|---|---|---|---|---|
| P2RY10 | -0.325 | Downregulated | 2.48E-02 | 2.29E-01 |
| FAM117B | -0.325 | Downregulated | 2.31E-02 | 2.20E-01 |
| METAP2 | -0.325 | Downregulated | 1.22E-03 | 3.79E-02 |
| RNF144 | -0.324 | Downregulated | 1.13E-02 | 1.47E-01 |
| ZNF22 | -0.324 | Downregulated | 4.52E-03 | 8.51E-02 |
| PFAAP5 | -0.323 | Downregulated | 1.26E-03 | 3.85E-02 |
| KIAA1430 | -0.323 | Downregulated | 3.71E-02 | 2.84E-01 |
| UPLP | -0.323 | Downregulated | 2.48E-02 | 2.28E-01 |
| STAG3L3 | -0.323 | Downregulated | 4.18E-03 | 8.09E-02 |
| SIT1 | -0.323 | Downregulated | 1.69E-02 | 1.85E-01 |
| RPLP0 | -0.323 | Downregulated | 4.73E-02 | 3.18E-01 |
| MND1 | -0.323 | Downregulated | 2.24E-02 | 2.16E-01 |
| OPTN | -0.323 | Downregulated | 3.50E-02 | 2.74E-01 |
| KNTC1 | -0.323 | Downregulated | 1.47E-02 | 1.71E-01 |
| MARCKSL1 | -0.323 | Downregulated | 2.04E-02 | 2.06E-01 |
| MIS12 | -0.322 | Downregulated | 3.23E-03 | 6.94E-02 |
| MKL2 | -0.322 | Downregulated | 8.75E-03 | 1.26E-01 |
| MOAP1 | -0.322 | Downregulated | 6.27E-04 | 2.40E-02 |
| TMEM200A | -0.322 | Downregulated | 6.79E-04 | 2.54E-02 |
| ALG5 | -0.322 | Downregulated | 4.30E-03 | 8.26E-02 |
| ATIC | -0.322 | Downregulated | 9.78E-03 | 1.36E-01 |
| CA5B | -0.321 | Downregulated | 9.91E-03 | 1.37E-01 |
| DDX18 | -0.321 | Downregulated | 1.96E-03 | 5.12E-02 |
| ORMDL3 | -0.321 | Downregulated | 3.06E-02 | 2.56E-01 |
| PPP1R2 | -0.321 | Downregulated | 1.22E-03 | 3.77E-02 |
| MRPL32 | -0.321 | Downregulated | 1.04E-03 | 3.42E-02 |
| SUB1 | -0.320 | Downregulated | 1.33E-02 | 1.62E-01 |
| KLHDC5 | -0.319 | Downregulated | 9.13E-04 | 3.12E-02 |
| TOMM7 | -0.319 | Downregulated | 1.36E-02 | 1.64E-01 |
| KIAA1370 | -0.319 | Downregulated | 2.35E-02 | 2.22E-01 |
| CBLB | -0.319 | Downregulated | 2.63E-02 | 2.36E-01 |
| SH3GLB2 | -0.319 | Downregulated | 2.52E-02 | 2.30E-01 |
| CDCA7 | -0.319 | Downregulated | 1.06E-02 | 1.42E-01 |
| NIPA1 | -0.318 | Downregulated | 1.02E-03 | 3.37E-02 |
| TOMM20 | -0.318 | Downregulated | 2.88E-03 | 6.52E-02 |
| CPA5 | -0.318 | Downregulated | 1.18E-02 | 1.51E-01 |
| DEXI | -0.318 | Downregulated | 1.23E-03 | 3.79E-02 |
| CARHSP1 | -0.318 | Downregulated | 2.11E-02 | 2.09E-01 |
| CATSPERB | -0.318 | Downregulated | 1.43E-03 | 4.22E-02 |
| DLAT | -0.318 | Downregulated | 4.14E-02 | 2.98E-01 |
| MRPS28 | -0.317 | Downregulated | 1.50E-02 | 1.73E-01 |
| PRKCQ | -0.317 | Downregulated | 9.15E-03 | 1.30E-01 |
| MEX3C | -0.317 | Downregulated | 3.60E-02 | 2.79E-01 |
| NIF3L1 | -0.316 | Downregulated | 1.29E-03 | 3.93E-02 |
| SLC38A1 | -0.316 | Downregulated | 5.45E-03 | 9.42E-02 |
| CRTC3 | -0.316 | Downregulated | 3.06E-03 | 6.78E-02 |
| RNF157 | -0.316 | Downregulated | 2.16E-03 | 5.43E-02 |
| MAT2A | -0.316 | Downregulated | 1.59E-04 | 1.02E-02 |
| LYRM7 | -0.315 | Downregulated | 2.51E-03 | 6.00E-02 |
| FBLN5 | -0.315 | Downregulated | 1.08E-02 | 1.44E-01 |
| FLJ33590 | -0.315 | Downregulated | 4.99E-02 | 3.26E-01 |

| | | | | |
|---|---|---|---|---|
| LPIN1 | -0.315 | Downregulated | 1.45E-02 | 1.70E-01 |
| ERAL1 | -0.314 | Downregulated | 1.43E-02 | 1.68E-01 |
| PRICKLE4 | -0.314 | Downregulated | 1.68E-04 | 1.05E-02 |
| MAGEF1 | -0.314 | Downregulated | 7.90E-03 | 1.18E-01 |
| NPAT | -0.314 | Downregulated | 1.62E-04 | 1.03E-02 |
| CHCHD6 | -0.314 | Downregulated | 3.41E-02 | 2.71E-01 |
| PAQR9 | -0.314 | Downregulated | 1.47E-02 | 1.71E-01 |
| ARRDC5 | -0.313 | Downregulated | 6.05E-03 | 1.00E-01 |
| TMEM57 | -0.313 | Downregulated | 1.41E-02 | 1.67E-01 |
| RCOR3 | -0.312 | Downregulated | 1.57E-03 | 4.47E-02 |
| GRAMD3 | -0.312 | Downregulated | 2.55E-02 | 2.32E-01 |
| BTAF1 | -0.312 | Downregulated | 8.20E-03 | 1.21E-01 |
| GLO1 | -0.311 | Downregulated | 1.62E-03 | 4.55E-02 |
| HNRPDL | -0.311 | Downregulated | 9.47E-03 | 1.33E-01 |
| MRPS6 | -0.310 | Downregulated | 5.41E-03 | 9.40E-02 |
| NAP1L4 | -0.310 | Downregulated | 5.14E-04 | 2.09E-02 |
| NCAPG2 | -0.310 | Downregulated | 3.33E-02 | 2.67E-01 |
| MINPP1 | -0.310 | Downregulated | 3.77E-02 | 2.86E-01 |
| Septin 1 | -0.309 | Downregulated | 1.71E-02 | 1.86E-01 |
| DPH5 | -0.309 | Downregulated | 2.16E-03 | 5.43E-02 |
| BIVM | -0.309 | Downregulated | 2.50E-02 | 2.29E-01 |
| RPL4 | -0.309 | Downregulated | 1.35E-02 | 1.64E-01 |
| AK1 | -0.309 | Downregulated | 1.28E-02 | 1.59E-01 |
| LSM7 | -0.309 | Downregulated | 6.09E-03 | 1.01E-01 |
| CTLA4 | -0.308 | Downregulated | 2.79E-02 | 2.44E-01 |
| IMPDH2 | -0.308 | Downregulated | 3.10E-02 | 2.57E-01 |
| GOLGA8B | -0.308 | Downregulated | 3.88E-04 | 1.75E-02 |
| RBM15 | -0.308 | Downregulated | 1.91E-02 | 1.98E-01 |
| PCID2 | -0.308 | Downregulated | 5.88E-03 | 9.85E-02 |
| SPTAN1 | -0.307 | Downregulated | 6.20E-03 | 1.02E-01 |
| EXOSC8 | -0.307 | Downregulated | 1.69E-03 | 4.65E-02 |
| DDX55 | -0.306 | Downregulated | 1.95E-03 | 5.10E-02 |
| TSPYL5 | -0.306 | Downregulated | 3.61E-02 | 2.80E-01 |
| RPS6 | -0.306 | Downregulated | 3.50E-03 | 7.26E-02 |
| FCGBP | -0.305 | Downregulated | 1.66E-03 | 4.60E-02 |
| ZFP90 | -0.305 | Downregulated | 5.77E-03 | 9.76E-02 |
| PDXP | -0.304 | Downregulated | 1.84E-02 | 1.94E-01 |
| PI4K2B | -0.304 | Downregulated | 6.43E-03 | 1.04E-01 |
| USP7 | -0.304 | Downregulated | 2.53E-02 | 2.31E-01 |
| SCGB1D4 | -0.304 | Downregulated | 2.04E-02 | 2.06E-01 |
| IFP38 | -0.304 | Downregulated | 3.19E-02 | 2.61E-01 |
| ZNF428 | -0.304 | Downregulated | 1.74E-03 | 4.73E-02 |
| NOL11 | -0.303 | Downregulated | 1.59E-02 | 1.79E-01 |
| MFSD6 | -0.303 | Downregulated | 7.21E-03 | 1.11E-01 |
| CEP290 | -0.302 | Downregulated | 2.90E-02 | 2.48E-01 |
| LYAR | -0.302 | Downregulated | 9.38E-03 | 1.32E-01 |
| SFRS2B | -0.302 | Downregulated | 1.46E-02 | 1.71E-01 |
| ZNF30 | -0.301 | Downregulated | 2.67E-02 | 2.38E-01 |
| EEF1E1 | -0.301 | Downregulated | 1.74E-02 | 1.88E-01 |
| LBH | -0.300 | Downregulated | 7.08E-03 | 1.10E-01 |
| RPL35 | -0.300 | Downregulated | 8.83E-03 | 1.27E-01 |

| | | | | |
|---|---|---|---|---|
| USP24 | -0.300 | Downregulated | 4.17E-04 | 1.84E-02 |
| EIF3J | -0.299 | Downregulated | 1.04E-02 | 1.41E-01 |
| APEX1 | -0.299 | Downregulated | 8.96E-03 | 1.28E-01 |
| ANGEL2 | -0.299 | Downregulated | 4.50E-03 | 8.50E-02 |
| NUCB2 | -0.299 | Downregulated | 3.40E-02 | 2.71E-01 |
| SLC25A42 | -0.299 | Downregulated | 4.86E-03 | 8.87E-02 |
| GLS | -0.299 | Downregulated | 1.46E-03 | 4.31E-02 |
| BCL11B | -0.299 | Downregulated | 2.98E-03 | 6.70E-02 |
| MRPS33 | -0.299 | Downregulated | 1.27E-02 | 1.58E-01 |
| NEIL2 | -0.298 | Downregulated | 4.10E-03 | 7.98E-02 |
| BRIX1 | -0.298 | Downregulated | 1.55E-02 | 1.76E-01 |
| DBP | -0.297 | Downregulated | 7.92E-03 | 1.18E-01 |
| ACAT1 | -0.297 | Downregulated | 5.64E-03 | 9.60E-02 |
| NSMCE4A | -0.297 | Downregulated | 7.87E-03 | 1.18E-01 |
| CDCA1 | -0.297 | Downregulated | 1.09E-02 | 1.44E-01 |
| OGT | -0.296 | Downregulated | 1.05E-03 | 3.44E-02 |
| SS18L2 | -0.296 | Downregulated | 3.18E-03 | 6.90E-02 |
| SIGLEC8 | -0.296 | Downregulated | 4.96E-02 | 3.26E-01 |
| EIF3A | -0.295 | Downregulated | 2.80E-02 | 2.45E-01 |
| POLR1E | -0.295 | Downregulated | 4.25E-02 | 3.01E-01 |
| KCNK12 | -0.295 | Downregulated | 3.29E-02 | 2.65E-01 |
| BCKDHB | -0.295 | Downregulated | 1.26E-03 | 3.87E-02 |
| SAE1 | -0.294 | Downregulated | 1.08E-02 | 1.44E-01 |
| RPAIN | -0.294 | Downregulated | 5.79E-03 | 9.78E-02 |
| FCRL5 | -0.294 | Downregulated | 3.53E-02 | 2.76E-01 |
| SUSD3 | -0.294 | Downregulated | 5.83E-03 | 9.82E-02 |
| KIF14 | -0.293 | Downregulated | 3.34E-02 | 2.68E-01 |
| HIST1H4C | -0.293 | Downregulated | 1.79E-02 | 1.91E-01 |
| MRPL14 | -0.293 | Downregulated | 7.98E-03 | 1.18E-01 |
| NELF | -0.293 | Downregulated | 1.42E-02 | 1.68E-01 |
| XPO4 | -0.293 | Downregulated | 1.59E-02 | 1.79E-01 |
| RALGDS | -0.292 | Downregulated | 4.73E-04 | 1.99E-02 |
| PRKRIR | -0.292 | Downregulated | 1.24E-03 | 3.82E-02 |
| KRT28 | -0.292 | Downregulated | 2.89E-02 | 2.48E-01 |
| MLLT11 | -0.292 | Downregulated | 9.75E-03 | 1.35E-01 |
| PABPC4 | -0.292 | Downregulated | 9.13E-03 | 1.30E-01 |
| SFRS6 | -0.291 | Downregulated | 3.31E-03 | 7.04E-02 |
| STOML2 | -0.291 | Downregulated | 9.36E-03 | 1.32E-01 |
| LIG1 | -0.291 | Downregulated | 2.58E-02 | 2.33E-01 |
| MBNL2 | -0.291 | Downregulated | 3.24E-02 | 2.63E-01 |
| CES3 | -0.291 | Downregulated | 3.01E-02 | 2.54E-01 |
| CHAC2 | -0.291 | Downregulated | 3.16E-02 | 2.60E-01 |
| POLR2H | -0.291 | Downregulated | 7.81E-03 | 1.17E-01 |
| RBBP7 | -0.291 | Downregulated | 1.71E-02 | 1.86E-01 |
| SBDS | -0.290 | Downregulated | 4.88E-02 | 3.22E-01 |
| TBC1D19 | -0.290 | Downregulated | 2.33E-02 | 2.21E-01 |
| TET1 | -0.290 | Downregulated | 1.33E-02 | 1.62E-01 |
| DOCK10 | -0.290 | Downregulated | 3.40E-02 | 2.71E-01 |
| UBE2Q2 | -0.290 | Downregulated | 2.08E-03 | 5.30E-02 |
| SLFN13 | -0.289 | Downregulated | 3.44E-02 | 2.72E-01 |
| ZFP161 | -0.289 | Downregulated | 8.55E-04 | 2.98E-02 |

| | | | | |
|---|---|---|---|---|
| COPS3 | -0.289 | Downregulated | 5.32E-03 | 9.30E-02 |
| TBC1D10C | -0.289 | Downregulated | 9.86E-03 | 1.36E-01 |
| TECR | -0.289 | Downregulated | 1.59E-02 | 1.79E-01 |
| PTPLB | -0.289 | Downregulated | 1.45E-02 | 1.70E-01 |
| STAG3L2 | -0.289 | Downregulated | 1.53E-02 | 1.74E-01 |
| TSEN15 | -0.288 | Downregulated | 1.24E-02 | 1.56E-01 |
| PLSCR3 | -0.288 | Downregulated | 5.85E-03 | 9.83E-02 |
| GTSF1L | -0.288 | Downregulated | 2.42E-02 | 2.25E-01 |
| BZW2 | -0.288 | Downregulated | 1.30E-02 | 1.60E-01 |
| PPAT | -0.288 | Downregulated | 2.75E-02 | 2.43E-01 |
| MST4 | -0.287 | Downregulated | 1.80E-03 | 4.84E-02 |
| CHD9 | -0.287 | Downregulated | 3.48E-03 | 7.24E-02 |
| RPS3 | -0.287 | Downregulated | 5.01E-03 | 9.03E-02 |
| MKI67IP | -0.286 | Downregulated | 3.91E-03 | 7.74E-02 |
| CERK | -0.286 | Downregulated | 4.92E-03 | 8.94E-02 |
| DUS4L | -0.286 | Downregulated | 1.43E-02 | 1.68E-01 |
| NDUFS5 | -0.285 | Downregulated | 2.21E-02 | 2.15E-01 |
| UBE2N | -0.285 | Downregulated | 2.47E-03 | 5.95E-02 |
| TOP2B | -0.285 | Downregulated | 7.91E-03 | 1.18E-01 |
| PPP1CC | -0.285 | Downregulated | 6.43E-04 | 2.44E-02 |
| TAF9 | -0.284 | Downregulated | 2.35E-03 | 5.77E-02 |
| CKS1B | -0.284 | Downregulated | 4.41E-02 | 3.07E-01 |
| RPL7A | -0.283 | Downregulated | 1.36E-02 | 1.64E-01 |
| ERH | -0.283 | Downregulated | 6.18E-03 | 1.02E-01 |
| CSAG2 | -0.283 | Downregulated | 2.63E-02 | 2.36E-01 |
| CTGLF3 | -0.283 | Downregulated | 3.92E-03 | 7.76E-02 |
| OXCT1 | -0.282 | Downregulated | 3.15E-03 | 6.86E-02 |
| ELP3 | -0.282 | Downregulated | 3.00E-03 | 6.71E-02 |
| SOD1 | -0.282 | Downregulated | 1.31E-03 | 3.97E-02 |
| FASN | -0.281 | Downregulated | 2.36E-02 | 2.23E-01 |
| ASMTL | -0.281 | Downregulated | 3.51E-03 | 7.26E-02 |
| ILF3 | -0.281 | Downregulated | 6.75E-03 | 1.07E-01 |
| PRPS1 | -0.281 | Downregulated | 3.39E-03 | 7.11E-02 |
| NPIP | -0.280 | Downregulated | 4.85E-03 | 8.86E-02 |
| MIF | -0.280 | Downregulated | 3.00E-02 | 2.53E-01 |
| ZNF26 | -0.279 | Downregulated | 4.24E-04 | 1.85E-02 |
| RPS6KB1 | -0.279 | Downregulated | 1.10E-02 | 1.45E-01 |
| ARL6IP1 | -0.279 | Downregulated | 2.80E-04 | 1.42E-02 |
| CSE1L | -0.279 | Downregulated | 6.03E-03 | 1.00E-01 |
| BLM | -0.279 | Downregulated | 1.34E-02 | 1.63E-01 |
| NARG1L | -0.278 | Downregulated | 4.39E-02 | 3.07E-01 |
| DNMT1 | -0.278 | Downregulated | 2.81E-02 | 2.46E-01 |
| FKBP2 | -0.277 | Downregulated | 1.42E-02 | 1.68E-01 |
| TMEM177 | -0.277 | Downregulated | 2.03E-03 | 5.23E-02 |
| CCDC109B | -0.277 | Downregulated | 2.11E-02 | 2.10E-01 |
| PNPLA7 | -0.277 | Downregulated | 4.36E-02 | 3.06E-01 |
| PUS7 | -0.276 | Downregulated | 2.58E-04 | 1.35E-02 |
| AGK | -0.276 | Downregulated | 1.90E-02 | 1.98E-01 |
| DARS | -0.276 | Downregulated | 2.22E-03 | 5.55E-02 |
| KAT2B | -0.275 | Downregulated | 4.80E-02 | 3.20E-01 |
| OSTC | -0.275 | Downregulated | 1.22E-02 | 1.55E-01 |

| | | | | |
|---|---|---|---|---|
| SET | -0.275 | Downregulated | 1.02E-02 | 1.39E-01 |
| TOMM70A | -0.275 | Downregulated | 2.44E-04 | 1.31E-02 |
| RASSF7 | -0.274 | Downregulated | 1.01E-02 | 1.38E-01 |
| MPHOSPH9 | -0.274 | Downregulated | 3.02E-02 | 2.54E-01 |
| PRKRA | -0.274 | Downregulated | 1.77E-02 | 1.90E-01 |
| FOXO1 | -0.274 | Downregulated | 8.95E-03 | 1.28E-01 |
| IQGAP2 | -0.274 | Downregulated | 3.10E-03 | 6.80E-02 |
| EDG1 | -0.273 | Downregulated | 2.06E-02 | 2.07E-01 |
| DUSP8 | -0.273 | Downregulated | 2.17E-02 | 2.13E-01 |
| LRPPRC | -0.273 | Downregulated | 9.11E-05 | 7.26E-03 |
| DERL1 | -0.273 | Downregulated | 3.32E-03 | 7.04E-02 |
| LRRC31 | -0.272 | Downregulated | 5.61E-03 | 9.59E-02 |
| NET1 | -0.272 | Downregulated | 3.11E-02 | 2.58E-01 |
| VIL2 | -0.272 | Downregulated | 3.29E-02 | 2.65E-01 |
| LNX2 | -0.272 | Downregulated | 2.89E-02 | 2.48E-01 |
| KIAA1128 | -0.272 | Downregulated | 3.55E-04 | 1.64E-02 |
| ZNF84 | -0.272 | Downregulated | 2.25E-02 | 2.17E-01 |
| MTMR10 | -0.272 | Downregulated | 2.03E-03 | 5.23E-02 |
| RAPGEF6 | -0.272 | Downregulated | 4.66E-03 | 8.67E-02 |
| CD8B | -0.271 | Downregulated | 2.98E-02 | 2.52E-01 |
| NDUFAF2 | -0.271 | Downregulated | 9.80E-03 | 1.36E-01 |
| DIMT1L | -0.271 | Downregulated | 3.07E-03 | 6.78E-02 |
| SFRS10 | -0.271 | Downregulated | 6.07E-03 | 1.01E-01 |
| FAM38A | -0.271 | Downregulated | 4.34E-03 | 8.31E-02 |
| EEF1B2 | -0.270 | Downregulated | 4.19E-02 | 3.00E-01 |
| CD47 | -0.270 | Downregulated | 2.36E-02 | 2.22E-01 |
| SUCLG2 | -0.270 | Downregulated | 1.02E-02 | 1.39E-01 |
| ARMC1 | -0.270 | Downregulated | 1.47E-03 | 4.33E-02 |
| FAM190B | -0.270 | Downregulated | 3.74E-03 | 7.53E-02 |
| C1QBP | -0.270 | Downregulated | 1.27E-02 | 1.58E-01 |
| FAM128A | -0.270 | Downregulated | 1.90E-03 | 5.00E-02 |
| RIC3 | -0.269 | Downregulated | 1.39E-03 | 4.14E-02 |
| AMOT | -0.269 | Downregulated | 3.97E-03 | 7.82E-02 |
| ELOVL4 | -0.269 | Downregulated | 1.38E-02 | 1.66E-01 |
| ZNF512 | -0.269 | Downregulated | 6.30E-04 | 2.40E-02 |
| NLRC3 | -0.269 | Downregulated | 4.02E-02 | 2.94E-01 |
| CCBP2 | -0.268 | Downregulated | 3.83E-02 | 2.88E-01 |
| NUP54 | -0.268 | Downregulated | 2.32E-02 | 2.21E-01 |
| NDUFB2 | -0.267 | Downregulated | 6.24E-03 | 1.02E-01 |
| PRPF8 | -0.267 | Downregulated | 9.94E-03 | 1.37E-01 |
| ZNF140 | -0.266 | Downregulated | 5.82E-03 | 9.80E-02 |
| GSPT2 | -0.266 | Downregulated | 2.72E-02 | 2.41E-01 |
| GPR88 | -0.266 | Downregulated | 1.46E-02 | 1.71E-01 |
| WDR61 | -0.266 | Downregulated | 6.98E-03 | 1.09E-01 |
| PGA5 | -0.266 | Downregulated | 2.48E-02 | 2.28E-01 |
| AES | -0.266 | Downregulated | 3.92E-02 | 2.91E-01 |
| FARS2 | -0.266 | Downregulated | 1.02E-02 | 1.39E-01 |
| LILRA1 | 0.265 | Upregulated | 2.65E-02 | 2.37E-01 |
| DAPP1 | 0.265 | Upregulated | 1.00E-02 | 1.37E-01 |
| ABO | 0.265 | Upregulated | 1.94E-02 | 2.00E-01 |
| LIN37 | 0.265 | Upregulated | 1.49E-02 | 1.72E-01 |

| | | | | |
|---|---|---|---|---|
| BTN2A1 | 0.265 | Upregulated | 6.20E-04 | 2.38E-02 |
| HIAT1 | 0.265 | Upregulated | 2.60E-04 | 1.36E-02 |
| PAPOLG | 0.265 | Upregulated | 2.02E-03 | 5.21E-02 |
| ABR | 0.266 | Upregulated | 8.79E-03 | 1.27E-01 |
| COTL1 | 0.266 | Upregulated | 1.93E-02 | 2.00E-01 |
| C5 | 0.266 | Upregulated | 1.89E-02 | 1.97E-01 |
| WDFY1 | 0.266 | Upregulated | 3.98E-03 | 7.84E-02 |
| TMEM2 | 0.266 | Upregulated | 1.55E-02 | 1.76E-01 |
| CDC42EP4 | 0.266 | Upregulated | 1.25E-02 | 1.56E-01 |
| USP32 | 0.266 | Upregulated | 1.29E-03 | 3.93E-02 |
| NKIRAS1 | 0.266 | Upregulated | 1.19E-02 | 1.52E-01 |
| RBM18 | 0.267 | Upregulated | 2.29E-03 | 5.67E-02 |
| FAM21C | 0.267 | Upregulated | 7.45E-03 | 1.14E-01 |
| SNAP23 | 0.267 | Upregulated | 2.75E-02 | 2.42E-01 |
| RMI1 | 0.267 | Upregulated | 2.47E-02 | 2.28E-01 |
| ATP6V1C1 | 0.267 | Upregulated | 9.76E-03 | 1.35E-01 |
| ARPC1A | 0.268 | Upregulated | 4.55E-03 | 8.54E-02 |
| ARRDC4 | 0.268 | Upregulated | 4.42E-02 | 3.08E-01 |
| FBXO38 | 0.268 | Upregulated | 8.23E-04 | 2.92E-02 |
| TNNT1 | 0.268 | Upregulated | 3.18E-02 | 2.61E-01 |
| BCORL1 | 0.269 | Upregulated | 2.59E-03 | 6.10E-02 |
| SDCCAG8 | 0.269 | Upregulated | 1.23E-02 | 1.55E-01 |
| BCAS2 | 0.269 | Upregulated | 2.17E-04 | 1.22E-02 |
| RASSF5 | 0.269 | Upregulated | 4.31E-02 | 3.04E-01 |
| TXNIP | 0.269 | Upregulated | 1.13E-03 | 3.61E-02 |
| FAM160A2 | 0.269 | Upregulated | 2.66E-02 | 2.38E-01 |
| FAM91A1 | 0.269 | Upregulated | 2.81E-03 | 6.43E-02 |
| NFKBIE | 0.269 | Upregulated | 1.07E-03 | 3.48E-02 |
| DNAJC13 | 0.269 | Upregulated | 1.69E-02 | 1.85E-01 |
| PRKAR2A | 0.269 | Upregulated | 1.57E-03 | 4.47E-02 |
| GPR155 | 0.270 | Upregulated | 3.87E-04 | 1.74E-02 |
| LAMP1 | 0.270 | Upregulated | 4.43E-03 | 8.42E-02 |
| NDN | 0.270 | Upregulated | 1.39E-02 | 1.66E-01 |
| RB1 | 0.271 | Upregulated | 1.11E-02 | 1.46E-01 |
| S100Z | 0.271 | Upregulated | 9.50E-04 | 3.21E-02 |
| BPTF | 0.271 | Upregulated | 2.48E-03 | 5.96E-02 |
| RASA2 | 0.271 | Upregulated | 9.18E-03 | 1.30E-01 |
| MSR1 | 0.272 | Upregulated | 7.73E-04 | 2.79E-02 |
| SLIC1 | 0.272 | Upregulated | 2.64E-03 | 6.18E-02 |
| N4BP1 | 0.272 | Upregulated | 1.13E-03 | 3.61E-02 |
| GLIPR1 | 0.272 | Upregulated | 3.08E-03 | 6.78E-02 |
| OR9G4 | 0.272 | Upregulated | 6.76E-03 | 1.07E-01 |
| ACP2 | 0.272 | Upregulated | 2.82E-02 | 2.46E-01 |
| ORMDL2 | 0.272 | Upregulated | 1.12E-03 | 3.59E-02 |
| GTPBP2 | 0.272 | Upregulated | 4.94E-02 | 3.25E-01 |
| TMCO3 | 0.272 | Upregulated | 4.17E-02 | 2.99E-01 |
| ITLN2 | 0.272 | Upregulated | 2.54E-02 | 2.31E-01 |
| NT5C2 | 0.272 | Upregulated | 5.71E-03 | 9.69E-02 |
| MAN2A2 | 0.273 | Upregulated | 2.32E-02 | 2.21E-01 |
| RRAGC | 0.273 | Upregulated | 9.90E-04 | 3.30E-02 |
| CHIC2 | 0.273 | Upregulated | 3.16E-03 | 6.87E-02 |

| | | | | |
|---|---|---|---|---|
| RCVRN | 0.274 | Upregulated | 7.88E-03 | 1.18E-01 |
| TIPARP | 0.274 | Upregulated | 3.67E-03 | 7.42E-02 |
| ARHGAP9 | 0.274 | Upregulated | 1.63E-03 | 4.55E-02 |
| CCR2 | 0.274 | Upregulated | 2.47E-04 | 1.32E-02 |
| ANKHD1-EIF4EBP3 | 0.274 | Upregulated | 9.81E-03 | 1.36E-01 |
| SCARB2 | 0.274 | Upregulated | 7.79E-04 | 2.80E-02 |
| AP3S2 | 0.274 | Upregulated | 5.50E-03 | 9.46E-02 |
| PKM2 | 0.275 | Upregulated | 1.56E-02 | 1.76E-01 |
| RGS19 | 0.275 | Upregulated | 2.31E-02 | 2.20E-01 |
| STK35 | 0.276 | Upregulated | 2.07E-03 | 5.29E-02 |
| PSMB8 | 0.276 | Upregulated | 2.75E-04 | 1.40E-02 |
| DEGS1 | 0.277 | Upregulated | 1.08E-03 | 3.50E-02 |
| STARD8 | 0.277 | Upregulated | 3.03E-03 | 6.75E-02 |
| GALC | 0.277 | Upregulated | 1.20E-04 | 8.62E-03 |
| MUTYH | 0.278 | Upregulated | 4.95E-03 | 8.98E-02 |
| ZBTB47 | 0.278 | Upregulated | 1.79E-03 | 4.83E-02 |
| DDEF2 | 0.278 | Upregulated | 4.16E-02 | 2.99E-01 |
| KPNB1 | 0.278 | Upregulated | 3.00E-04 | 1.47E-02 |
| SCARF1 | 0.278 | Upregulated | 4.79E-03 | 8.82E-02 |
| NR2C2 | 0.278 | Upregulated | 6.73E-03 | 1.07E-01 |
| CLDN9 | 0.279 | Upregulated | 6.80E-04 | 2.54E-02 |
| GPR77 | 0.279 | Upregulated | 9.53E-03 | 1.34E-01 |
| CCDC137 | 0.280 | Upregulated | 2.26E-02 | 2.17E-01 |
| GSN | 0.280 | Upregulated | 3.18E-03 | 6.90E-02 |
| PPP2R3A | 0.281 | Upregulated | 2.24E-03 | 5.57E-02 |
| SNX30 | 0.281 | Upregulated | 7.67E-03 | 1.16E-01 |
| PCIF1 | 0.281 | Upregulated | 3.29E-03 | 7.01E-02 |
| ERGIC1 | 0.281 | Upregulated | 3.35E-02 | 2.68E-01 |
| PDLIM5 | 0.281 | Upregulated | 5.03E-04 | 2.07E-02 |
| MGAT1 | 0.282 | Upregulated | 6.98E-04 | 2.59E-02 |
| PHC3 | 0.282 | Upregulated | 2.39E-02 | 2.24E-01 |
| FAM129B | 0.282 | Upregulated | 1.36E-02 | 1.64E-01 |
| LCN8 | 0.282 | Upregulated | 3.68E-02 | 2.82E-01 |
| CASZ1 | 0.282 | Upregulated | 3.64E-03 | 7.41E-02 |
| CENTD2 | 0.282 | Upregulated | 3.07E-03 | 6.78E-02 |
| ERF | 0.283 | Upregulated | 1.07E-02 | 1.43E-01 |
| GLB1 | 0.283 | Upregulated | 1.72E-04 | 1.06E-02 |
| MYO10 | 0.283 | Upregulated | 2.61E-02 | 2.35E-01 |
| OR52K2 | 0.284 | Upregulated | 3.48E-03 | 7.24E-02 |
| WDR45L | 0.284 | Upregulated | 8.16E-03 | 1.21E-01 |
| MOBKL2A | 0.284 | Upregulated | 3.26E-03 | 6.96E-02 |
| PXN | 0.284 | Upregulated | 1.41E-02 | 1.67E-01 |
| XKR8 | 0.285 | Upregulated | 3.50E-04 | 1.63E-02 |
| ESRRA | 0.285 | Upregulated | 4.69E-03 | 8.71E-02 |
| ZFP106 | 0.285 | Upregulated | 3.44E-03 | 7.18E-02 |
| HIST2H4A | 0.286 | Upregulated | 3.92E-03 | 7.75E-02 |
| OTOS | 0.286 | Upregulated | 1.21E-02 | 1.54E-01 |
| SMPDL3A | 0.286 | Upregulated | 3.04E-02 | 2.55E-01 |
| COQ10A | 0.286 | Upregulated | 3.53E-03 | 7.30E-02 |
| BCKDK | 0.286 | Upregulated | 1.23E-03 | 3.81E-02 |
| SLC2A6 | 0.287 | Upregulated | 1.40E-03 | 4.16E-02 |

| | | | | |
|---|---|---|---|---|
| CYTH2 | 0.287 | Upregulated | 1.63E-02 | 1.81E-01 |
| PNPLA1 | 0.287 | Upregulated | 1.09E-02 | 1.44E-01 |
| VPS39 | 0.287 | Upregulated | 6.66E-03 | 1.06E-01 |
| SLC27A1 | 0.288 | Upregulated | 4.86E-02 | 3.22E-01 |
| ELL | 0.288 | Upregulated | 2.69E-04 | 1.38E-02 |
| GPR162 | 0.288 | Upregulated | 4.20E-02 | 3.00E-01 |
| CSF2RA | 0.289 | Upregulated | 8.47E-03 | 1.24E-01 |
| TKT | 0.289 | Upregulated | 3.94E-03 | 7.78E-02 |
| ATPAF2 | 0.289 | Upregulated | 2.98E-02 | 2.52E-01 |
| NR1H2 | 0.289 | Upregulated | 1.38E-04 | 9.46E-03 |
| IFRD1 | 0.289 | Upregulated | 9.75E-03 | 1.35E-01 |
| SLC16A6 | 0.289 | Upregulated | 1.60E-03 | 4.51E-02 |
| PLP2 | 0.289 | Upregulated | 3.30E-02 | 2.65E-01 |
| SLC27A3 | 0.289 | Upregulated | 6.74E-03 | 1.07E-01 |
| GLRX | 0.289 | Upregulated | 8.56E-03 | 1.25E-01 |
| SLC45A3 | 0.289 | Upregulated | 3.70E-02 | 2.83E-01 |
| CBLN3 | 0.290 | Upregulated | 2.04E-02 | 2.06E-01 |
| KAL1 | 0.290 | Upregulated | 1.80E-02 | 1.92E-01 |
| PRCP | 0.290 | Upregulated | 5.68E-03 | 9.65E-02 |
| SCN9A | 0.290 | Upregulated | 1.24E-02 | 1.56E-01 |
| SECTM1 | 0.290 | Upregulated | 1.21E-02 | 1.54E-01 |
| PSMB3 | 0.291 | Upregulated | 3.63E-04 | 1.67E-02 |
| CSNK1A1 | 0.291 | Upregulated | 6.17E-03 | 1.02E-01 |
| MS4A4A | 0.291 | Upregulated | 3.53E-02 | 2.76E-01 |
| KYNU | 0.291 | Upregulated | 6.29E-03 | 1.03E-01 |
| DPYD | 0.292 | Upregulated | 1.95E-02 | 2.00E-01 |
| GDAP2 | 0.292 | Upregulated | 2.13E-03 | 5.39E-02 |
| BLOC1S1 | 0.292 | Upregulated | 1.37E-02 | 1.65E-01 |
| GAB3 | 0.292 | Upregulated | 2.37E-02 | 2.23E-01 |
| STRADA | 0.292 | Upregulated | 5.84E-04 | 2.27E-02 |
| RIPK2 | 0.292 | Upregulated | 4.94E-05 | 4.96E-03 |
| PPP1R11 | 0.292 | Upregulated | 7.02E-03 | 1.09E-01 |
| ATOX1 | 0.292 | Upregulated | 2.80E-03 | 6.42E-02 |
| ZNF131 | 0.293 | Upregulated | 7.27E-03 | 1.12E-01 |
| KIF5B | 0.293 | Upregulated | 1.42E-02 | 1.68E-01 |
| ECM1 | 0.293 | Upregulated | 1.48E-03 | 4.34E-02 |
| MCTP1 | 0.293 | Upregulated | 2.57E-03 | 6.07E-02 |
| DIP2B | 0.293 | Upregulated | 8.76E-04 | 3.02E-02 |
| RSPH9 | 0.293 | Upregulated | 1.05E-02 | 1.42E-01 |
| INHBB | 0.294 | Upregulated | 3.36E-03 | 7.07E-02 |
| ZFHX3 | 0.295 | Upregulated | 1.98E-02 | 2.03E-01 |
| CDH23 | 0.295 | Upregulated | 1.70E-03 | 4.67E-02 |
| DPH3 | 0.295 | Upregulated | 6.76E-03 | 1.07E-01 |
| CFB | 0.296 | Upregulated | 3.79E-03 | 7.60E-02 |
| MAP3K3 | 0.296 | Upregulated | 7.97E-04 | 2.84E-02 |
| CCDC88B | 0.296 | Upregulated | 5.86E-03 | 9.84E-02 |
| CINP | 0.296 | Upregulated | 3.98E-02 | 2.93E-01 |
| RIPK1 | 0.296 | Upregulated | 2.08E-04 | 1.18E-02 |
| TOLLIP | 0.297 | Upregulated | 5.92E-03 | 9.90E-02 |
| RBMS2 | 0.297 | Upregulated | 1.20E-02 | 1.53E-01 |
| SLC25A20 | 0.298 | Upregulated | 1.01E-02 | 1.38E-01 |

| | | | | |
|---|---|---|---|---|
| UAP1L1 | 0.298 | Upregulated | 1.52E-03 | 4.39E-02 |
| NKX2-5 | 0.298 | Upregulated | 1.28E-02 | 1.59E-01 |
| KIAA0226 | 0.299 | Upregulated | 1.48E-03 | 4.34E-02 |
| RAB5C | 0.299 | Upregulated | 1.53E-04 | 1.01E-02 |
| RIN3 | 0.299 | Upregulated | 6.51E-03 | 1.05E-01 |
| SLC4A1AP | 0.299 | Upregulated | 3.86E-02 | 2.89E-01 |
| CNN2 | 0.299 | Upregulated | 4.24E-03 | 8.18E-02 |
| STAM2 | 0.300 | Upregulated | 7.94E-03 | 1.18E-01 |
| LYSMD2 | 0.300 | Upregulated | 4.30E-04 | 1.86E-02 |
| FIG4 | 0.301 | Upregulated | 9.61E-04 | 3.23E-02 |
| KLHL8 | 0.301 | Upregulated | 6.98E-04 | 2.59E-02 |
| SRPK1 | 0.301 | Upregulated | 1.27E-02 | 1.58E-01 |
| NIT1 | 0.301 | Upregulated | 1.89E-03 | 4.98E-02 |
| ITGB2 | 0.301 | Upregulated | 4.93E-04 | 2.05E-02 |
| GBE1 | 0.301 | Upregulated | 5.78E-03 | 9.77E-02 |
| CBR1 | 0.301 | Upregulated | 6.01E-03 | 1.00E-01 |
| SETD7 | 0.301 | Upregulated | 4.51E-03 | 8.50E-02 |
| RCN3 | 0.301 | Upregulated | 1.50E-03 | 4.36E-02 |
| CLEC4G | 0.301 | Upregulated | 2.11E-03 | 5.35E-02 |
| PVRL2 | 0.301 | Upregulated | 6.37E-03 | 1.04E-01 |
| TPP1 | 0.302 | Upregulated | 3.05E-03 | 6.78E-02 |
| TUBA1A | 0.302 | Upregulated | 1.42E-04 | 9.67E-03 |
| CEBPA | 0.302 | Upregulated | 3.30E-02 | 2.65E-01 |
| OR51A2 | 0.302 | Upregulated | 8.36E-03 | 1.23E-01 |
| HLA-G | 0.303 | Upregulated | 4.65E-02 | 3.15E-01 |
| CCNYL1 | 0.303 | Upregulated | 2.86E-03 | 6.50E-02 |
| RASSF4 | 0.303 | Upregulated | 1.48E-03 | 4.33E-02 |
| HERPUD2 | 0.303 | Upregulated | 4.64E-04 | 1.96E-02 |
| EGR1 | 0.303 | Upregulated | 6.11E-03 | 1.01E-01 |
| IL13 | 0.303 | Upregulated | 4.33E-02 | 3.04E-01 |
| CHI3L1 | 0.304 | Upregulated | 5.02E-03 | 9.03E-02 |
| FBXO39 | 0.304 | Upregulated | 3.00E-02 | 2.53E-01 |
| USF1 | 0.304 | Upregulated | 2.81E-04 | 1.42E-02 |
| MAEA | 0.304 | Upregulated | 2.05E-02 | 2.06E-01 |
| RAB11FIP1 | 0.304 | Upregulated | 3.62E-03 | 7.39E-02 |
| RRAS | 0.304 | Upregulated | 1.80E-02 | 1.92E-01 |
| NUPL1 | 0.304 | Upregulated | 2.75E-03 | 6.34E-02 |
| NOTCH1 | 0.305 | Upregulated | 2.48E-03 | 5.96E-02 |
| ZNF654 | 0.306 | Upregulated | 6.64E-03 | 1.06E-01 |
| SLC22A18 | 0.306 | Upregulated | 4.48E-04 | 1.92E-02 |
| ARPC1B | 0.306 | Upregulated | 4.94E-03 | 8.97E-02 |
| TAGAP | 0.307 | Upregulated | 8.93E-05 | 7.26E-03 |
| PSAP | 0.307 | Upregulated | 3.09E-03 | 6.79E-02 |
| GAS6 | 0.308 | Upregulated | 2.15E-03 | 5.43E-02 |
| CSAD | 0.308 | Upregulated | 5.20E-03 | 9.17E-02 |
| NECAB1 | 0.308 | Upregulated | 2.67E-02 | 2.38E-01 |
| CYBA | 0.308 | Upregulated | 1.70E-03 | 4.67E-02 |
| OAF | 0.308 | Upregulated | 1.75E-02 | 1.89E-01 |
| FAM65B | 0.308 | Upregulated | 4.56E-04 | 1.93E-02 |
| ABHD3 | 0.309 | Upregulated | 6.29E-03 | 1.03E-01 |
| ARFIP1 | 0.309 | Upregulated | 2.17E-02 | 2.13E-01 |

| | | | | |
|---|---|---|---|---|
| ABHD2 | 0.309 | Upregulated | 1.51E-02 | 1.74E-01 |
| TMUB2 | 0.309 | Upregulated | 4.66E-03 | 8.67E-02 |
| STOML1 | 0.309 | Upregulated | 6.18E-03 | 1.02E-01 |
| TPM4 | 0.309 | Upregulated | 1.12E-02 | 1.46E-01 |
| SLC37A3 | 0.309 | Upregulated | 4.69E-02 | 3.17E-01 |
| LILRB1 | 0.309 | Upregulated | 4.12E-03 | 8.02E-02 |
| SMCHD1 | 0.310 | Upregulated | 9.18E-03 | 1.30E-01 |
| BAT5 | 0.310 | Upregulated | 2.33E-04 | 1.27E-02 |
| PADI2 | 0.310 | Upregulated | 1.07E-02 | 1.43E-01 |
| ZNF341 | 0.311 | Upregulated | 5.50E-03 | 9.46E-02 |
| MTHFS | 0.311 | Upregulated | 1.35E-02 | 1.64E-01 |
| TMEM184B | 0.311 | Upregulated | 3.77E-04 | 1.71E-02 |
| ANKRD50 | 0.311 | Upregulated | 8.04E-03 | 1.19E-01 |
| STXBP2 | 0.311 | Upregulated | 3.74E-02 | 2.85E-01 |
| VPS18 | 0.312 | Upregulated | 1.33E-02 | 1.63E-01 |
| GDPD3 | 0.312 | Upregulated | 1.64E-04 | 1.03E-02 |
| SIL1 | 0.312 | Upregulated | 4.23E-02 | 3.01E-01 |
| ARRB2 | 0.312 | Upregulated | 4.37E-03 | 8.35E-02 |
| DHX34 | 0.312 | Upregulated | 7.07E-04 | 2.61E-02 |
| FOSL2 | 0.313 | Upregulated | 1.11E-02 | 1.46E-01 |
| SLC35F5 | 0.314 | Upregulated | 6.24E-03 | 1.02E-01 |
| TTYH3 | 0.314 | Upregulated | 2.74E-03 | 6.33E-02 |
| SERTAD1 | 0.314 | Upregulated | 1.22E-03 | 3.79E-02 |
| NAGA | 0.314 | Upregulated | 1.27E-02 | 1.58E-01 |
| RRM2B | 0.314 | Upregulated | 8.02E-03 | 1.19E-01 |
| PIK3CD | 0.315 | Upregulated | 2.72E-04 | 1.40E-02 |
| CCDC149 | 0.315 | Upregulated | 6.70E-03 | 1.07E-01 |
| TMEM188 | 0.315 | Upregulated | 1.48E-03 | 4.34E-02 |
| CALCOCO2 | 0.315 | Upregulated | 9.27E-03 | 1.31E-01 |
| VWA5A | 0.315 | Upregulated | 5.61E-03 | 9.59E-02 |
| ACOT8 | 0.315 | Upregulated | 4.76E-03 | 8.79E-02 |
| ATP6V0B | 0.315 | Upregulated | 7.33E-04 | 2.68E-02 |
| SLC8A1 | 0.315 | Upregulated | 2.90E-04 | 1.44E-02 |
| HSDL2 | 0.315 | Upregulated | 4.45E-02 | 3.09E-01 |
| CD40 | 0.315 | Upregulated | 7.31E-03 | 1.13E-01 |
| FCGRT | 0.316 | Upregulated | 9.11E-03 | 1.30E-01 |
| SLC9A1 | 0.316 | Upregulated | 6.78E-03 | 1.07E-01 |
| LAMP2 | 0.316 | Upregulated | 1.09E-02 | 1.44E-01 |
| LAMP3 | 0.316 | Upregulated | 3.70E-02 | 2.84E-01 |
| CYB5R4 | 0.317 | Upregulated | 4.21E-03 | 8.13E-02 |
| BID | 0.317 | Upregulated | 5.67E-04 | 2.23E-02 |
| CPNE8 | 0.317 | Upregulated | 2.28E-02 | 2.19E-01 |
| QSOX1 | 0.317 | Upregulated | 1.74E-02 | 1.88E-01 |
| RRP12 | 0.317 | Upregulated | 3.73E-02 | 2.84E-01 |
| NCSTN | 0.317 | Upregulated | 2.65E-03 | 6.19E-02 |
| CIR1 | 0.317 | Upregulated | 2.75E-03 | 6.34E-02 |
| GSTO1 | 0.318 | Upregulated | 2.56E-03 | 6.07E-02 |
| SOLH | 0.318 | Upregulated | 1.71E-03 | 4.67E-02 |
| MAPK3 | 0.318 | Upregulated | 7.71E-04 | 2.79E-02 |
| VCX-C | 0.319 | Upregulated | 2.53E-03 | 6.02E-02 |
| NEXN | 0.319 | Upregulated | 1.06E-02 | 1.43E-01 |

| | | | | |
|---|---|---|---|---|
| HSD17B11 | 0.319 | Upregulated | 1.26E-02 | 1.57E-01 |
| PPCDC | 0.319 | Upregulated | 6.43E-03 | 1.04E-01 |
| RAP1A | 0.319 | Upregulated | 1.06E-03 | 3.46E-02 |
| VAV1 | 0.320 | Upregulated | 7.30E-04 | 2.67E-02 |
| BAZ2B | 0.320 | Upregulated | 5.75E-03 | 9.73E-02 |
| SUMF1 | 0.320 | Upregulated | 1.06E-03 | 3.46E-02 |
| ASAP2 | 0.320 | Upregulated | 1.62E-02 | 1.80E-01 |
| ACER3 | 0.320 | Upregulated | 2.38E-02 | 2.24E-01 |
| ZCCHC2 | 0.320 | Upregulated | 2.23E-02 | 2.16E-01 |
| FLJ45244 | 0.320 | Upregulated | 5.87E-03 | 9.85E-02 |
| MICAL1 | 0.320 | Upregulated | 6.01E-05 | 5.70E-03 |
| LRFN1 | 0.320 | Upregulated | 4.57E-03 | 8.56E-02 |
| MYCBP | 0.320 | Upregulated | 1.37E-02 | 1.65E-01 |
| ZDHHC12 | 0.320 | Upregulated | 5.69E-04 | 2.23E-02 |
| PRDM5 | 0.320 | Upregulated | 6.22E-03 | 1.02E-01 |
| EIF4G3 | 0.321 | Upregulated | 1.99E-03 | 5.17E-02 |
| MSL3 | 0.321 | Upregulated | 2.08E-03 | 5.30E-02 |
| PANK2 | 0.321 | Upregulated | 4.33E-05 | 4.55E-03 |
| ADAP1 | 0.321 | Upregulated | 4.47E-03 | 8.47E-02 |
| KIAA1632 | 0.321 | Upregulated | 5.86E-04 | 2.28E-02 |
| ELF4 | 0.322 | Upregulated | 6.62E-04 | 2.50E-02 |
| XRN1 | 0.322 | Upregulated | 4.95E-04 | 2.05E-02 |
| MSL3L1 | 0.323 | Upregulated | 1.03E-03 | 3.40E-02 |
| IL8 | 0.323 | Upregulated | 3.01E-02 | 2.54E-01 |
| PRKCD | 0.323 | Upregulated | 1.59E-04 | 1.02E-02 |
| NUAK2 | 0.323 | Upregulated | 2.11E-03 | 5.35E-02 |
| SRBD1 | 0.323 | Upregulated | 1.21E-04 | 8.66E-03 |
| MKNK1 | 0.323 | Upregulated | 3.65E-03 | 7.41E-02 |
| OSTF1 | 0.324 | Upregulated | 1.11E-04 | 8.25E-03 |
| MTMR3 | 0.324 | Upregulated | 2.91E-02 | 2.49E-01 |
| TFE3 | 0.324 | Upregulated | 1.86E-02 | 1.95E-01 |
| FLJ27255 | 0.324 | Upregulated | 5.24E-03 | 9.20E-02 |
| ZNF385A | 0.324 | Upregulated | 1.41E-03 | 4.17E-02 |
| HIST1H4E | 0.325 | Upregulated | 1.48E-02 | 1.72E-01 |
| MGC2752 | 0.325 | Upregulated | 3.12E-04 | 1.52E-02 |
| CUTL1 | 0.326 | Upregulated | 5.55E-03 | 9.51E-02 |
| PFTK1 | 0.326 | Upregulated | 2.54E-02 | 2.31E-01 |
| NFAM1 | 0.326 | Upregulated | 2.29E-03 | 5.66E-02 |
| MME | 0.326 | Upregulated | 2.35E-03 | 5.77E-02 |
| SEL1L | 0.326 | Upregulated | 9.21E-03 | 1.31E-01 |
| SAMD4B | 0.327 | Upregulated | 1.59E-03 | 4.51E-02 |
| GPSM3 | 0.327 | Upregulated | 6.02E-05 | 5.70E-03 |
| HIST2H4B | 0.328 | Upregulated | 2.18E-03 | 5.46E-02 |
| ENTPD1 | 0.328 | Upregulated | 9.89E-03 | 1.36E-01 |
| DHRS4L2 | 0.328 | Upregulated | 6.81E-03 | 1.07E-01 |
| FAM126B | 0.328 | Upregulated | 1.59E-03 | 4.51E-02 |
| SNAG1 | 0.328 | Upregulated | 4.57E-03 | 8.56E-02 |
| SSH2 | 0.328 | Upregulated | 3.22E-03 | 6.94E-02 |
| GPR177 | 0.329 | Upregulated | 4.94E-02 | 3.25E-01 |
| RNF217 | 0.329 | Upregulated | 8.31E-03 | 1.22E-01 |
| BTNL8 | 0.330 | Upregulated | 4.45E-03 | 8.44E-02 |

| | | | | |
|---|---|---|---|---|
| MFAP3 | 0.330 | Upregulated | 6.16E-04 | 2.37E-02 |
| SNX13 | 0.330 | Upregulated | 1.10E-02 | 1.45E-01 |
| RAB7A | 0.330 | Upregulated | 2.24E-04 | 1.24E-02 |
| EHBP1L1 | 0.331 | Upregulated | 6.40E-05 | 5.91E-03 |
| IRF9 | 0.331 | Upregulated | 2.67E-04 | 1.38E-02 |
| LSP1 | 0.331 | Upregulated | 3.85E-04 | 1.74E-02 |
| DNAJB12 | 0.332 | Upregulated | 9.12E-05 | 7.26E-03 |
| MLL3 | 0.332 | Upregulated | 2.31E-03 | 5.70E-02 |
| GORASP1 | 0.332 | Upregulated | 1.18E-03 | 3.69E-02 |
| TRIM6 | 0.332 | Upregulated | 6.67E-03 | 1.06E-01 |
| GPR42 | 0.332 | Upregulated | 3.26E-02 | 2.64E-01 |
| CD58 | 0.333 | Upregulated | 9.32E-03 | 1.32E-01 |
| CARD9 | 0.333 | Upregulated | 1.72E-02 | 1.86E-01 |
| CTSZ | 0.333 | Upregulated | 7.76E-04 | 2.80E-02 |
| ITGB5 | 0.333 | Upregulated | 2.64E-02 | 2.36E-01 |
| PTTG1IP | 0.334 | Upregulated | 1.06E-04 | 8.02E-03 |
| FAM53C | 0.334 | Upregulated | 6.86E-03 | 1.08E-01 |
| BRWD3 | 0.334 | Upregulated | 7.36E-03 | 1.13E-01 |
| MTHFD2 | 0.334 | Upregulated | 7.46E-03 | 1.14E-01 |
| GAS2L1 | 0.334 | Upregulated | 1.69E-02 | 1.85E-01 |
| NIN | 0.334 | Upregulated | 4.56E-03 | 8.55E-02 |
| ICAM1 | 0.334 | Upregulated | 1.30E-04 | 9.12E-03 |
| MS4A7 | 0.335 | Upregulated | 1.17E-02 | 1.50E-01 |
| FAM193B | 0.335 | Upregulated | 2.29E-04 | 1.26E-02 |
| AVIL | 0.337 | Upregulated | 7.14E-03 | 1.11E-01 |
| ACPP | 0.337 | Upregulated | 1.31E-02 | 1.61E-01 |
| ELF2 | 0.337 | Upregulated | 1.12E-02 | 1.46E-01 |
| ADORA2B | 0.337 | Upregulated | 4.53E-02 | 3.11E-01 |
| NXNL1 | 0.337 | Upregulated | 1.10E-03 | 3.56E-02 |
| KIAA1009 | 0.337 | Upregulated | 3.23E-04 | 1.56E-02 |
| AFF1 | 0.338 | Upregulated | 2.73E-04 | 1.40E-02 |
| RIPK3 | 0.338 | Upregulated | 6.80E-03 | 1.07E-01 |
| PATL1 | 0.339 | Upregulated | 6.86E-05 | 6.17E-03 |
| YIPF1 | 0.339 | Upregulated | 1.76E-04 | 1.07E-02 |
| PCDHB8 | 0.340 | Upregulated | 3.40E-03 | 7.12E-02 |
| EXTL3 | 0.340 | Upregulated | 4.09E-04 | 1.82E-02 |
| Septin 4 | 0.341 | Upregulated | 2.33E-05 | 3.29E-03 |
| SBF2 | 0.342 | Upregulated | 9.08E-05 | 7.26E-03 |
| PHF20L1 | 0.342 | Upregulated | 1.42E-02 | 1.68E-01 |
| KLF6 | 0.342 | Upregulated | 1.52E-03 | 4.39E-02 |
| IRAK4 | 0.342 | Upregulated | 4.96E-03 | 8.99E-02 |
| KIAA0513 | 0.342 | Upregulated | 6.80E-03 | 1.07E-01 |
| TBC1D23 | 0.343 | Upregulated | 2.27E-02 | 2.18E-01 |
| TRIM27 | 0.344 | Upregulated | 4.75E-04 | 1.99E-02 |
| LYRM1 | 0.344 | Upregulated | 4.44E-04 | 1.91E-02 |
| CENPBD1 | 0.344 | Upregulated | 1.34E-03 | 4.03E-02 |
| UGCGL1 | 0.344 | Upregulated | 2.78E-02 | 2.44E-01 |
| ARID4B | 0.344 | Upregulated | 5.24E-03 | 9.20E-02 |
| KCNE1L | 0.345 | Upregulated | 7.05E-04 | 2.60E-02 |
| TESK2 | 0.345 | Upregulated | 1.98E-04 | 1.15E-02 |
| MARCO | 0.346 | Upregulated | 2.14E-02 | 2.11E-01 |

| | | | | |
|---|---|---|---|---|
| RDH5 | 0.346 | Upregulated | 6.25E-03 | 1.02E-01 |
| KCTD17 | 0.346 | Upregulated | 2.52E-03 | 6.02E-02 |
| SLC24A4 | 0.346 | Upregulated | 8.86E-03 | 1.28E-01 |
| S100A6 | 0.346 | Upregulated | 9.96E-03 | 1.37E-01 |
| PPP4R1 | 0.347 | Upregulated | 2.18E-03 | 5.46E-02 |
| NHSL2 | 0.347 | Upregulated | 1.46E-04 | 9.84E-03 |
| HLA-F | 0.348 | Upregulated | 1.00E-04 | 7.67E-03 |
| CANT1 | 0.348 | Upregulated | 2.18E-04 | 1.22E-02 |
| NBEAL2 | 0.349 | Upregulated | 4.47E-03 | 8.47E-02 |
| ANKRD57 | 0.349 | Upregulated | 7.01E-03 | 1.09E-01 |
| RRBP1 | 0.350 | Upregulated | 3.87E-03 | 7.69E-02 |
| PAQR6 | 0.350 | Upregulated | 7.69E-03 | 1.16E-01 |
| ACOX1 | 0.350 | Upregulated | 1.45E-03 | 4.28E-02 |
| BATF | 0.350 | Upregulated | 2.86E-02 | 2.47E-01 |
| ZNF230 | 0.350 | Upregulated | 8.82E-03 | 1.27E-01 |
| SERPINB8 | 0.350 | Upregulated | 2.10E-03 | 5.34E-02 |
| 01-Mar | 0.350 | Upregulated | 7.11E-03 | 1.10E-01 |
| MAP3K5 | 0.351 | Upregulated | 1.04E-04 | 7.94E-03 |
| EDEM2 | 0.351 | Upregulated | 1.75E-04 | 1.07E-02 |
| TOR1AIP2 | 0.351 | Upregulated | 7.04E-03 | 1.10E-01 |
| RGS14 | 0.351 | Upregulated | 5.44E-04 | 2.18E-02 |
| PHCA | 0.352 | Upregulated | 9.82E-03 | 1.36E-01 |
| ADAR | 0.352 | Upregulated | 7.90E-04 | 2.82E-02 |
| FLJ10986 | 0.352 | Upregulated | 9.53E-03 | 1.34E-01 |
| IDH1 | 0.352 | Upregulated | 5.25E-04 | 2.12E-02 |
| TXNDC3 | 0.352 | Upregulated | 3.12E-02 | 2.58E-01 |
| PROK1 | 0.352 | Upregulated | 2.25E-03 | 5.59E-02 |
| ZNF292 | 0.353 | Upregulated | 3.54E-03 | 7.32E-02 |
| LCP1 | 0.354 | Upregulated | 1.59E-04 | 1.02E-02 |
| HELB | 0.354 | Upregulated | 1.98E-03 | 5.16E-02 |
| FBXL2 | 0.355 | Upregulated | 2.83E-04 | 1.42E-02 |
| CD53 | 0.355 | Upregulated | 2.86E-04 | 1.43E-02 |
| OS9 | 0.355 | Upregulated | 6.50E-05 | 5.98E-03 |
| UBQLNL | 0.355 | Upregulated | 1.38E-02 | 1.66E-01 |
| WFDC1 | 0.355 | Upregulated | 2.23E-03 | 5.56E-02 |
| AVL9 | 0.356 | Upregulated | 1.38E-04 | 9.46E-03 |
| SLCO2A1 | 0.357 | Upregulated | 3.56E-02 | 2.77E-01 |
| NSF | 0.357 | Upregulated | 2.07E-04 | 1.18E-02 |
| RGL2 | 0.357 | Upregulated | 4.68E-04 | 1.97E-02 |
| ARHGEF10L | 0.357 | Upregulated | 1.24E-04 | 8.82E-03 |
| TLR7 | 0.358 | Upregulated | 2.97E-02 | 2.52E-01 |
| OSBPL11 | 0.358 | Upregulated | 1.11E-02 | 1.46E-01 |
| ANKS1A | 0.358 | Upregulated | 9.48E-04 | 3.21E-02 |
| ZNF366 | 0.358 | Upregulated | 3.39E-03 | 7.12E-02 |
| FAM8A1 | 0.358 | Upregulated | 5.64E-03 | 9.60E-02 |
| PRICKLE1 | 0.359 | Upregulated | 4.65E-02 | 3.15E-01 |
| HN1 | 0.359 | Upregulated | 1.18E-03 | 3.69E-02 |
| ZNF319 | 0.359 | Upregulated | 2.58E-03 | 6.08E-02 |
| GMIP | 0.360 | Upregulated | 4.76E-04 | 1.99E-02 |
| TRIM38 | 0.360 | Upregulated | 1.19E-04 | 8.62E-03 |
| LRP3 | 0.360 | Upregulated | 8.34E-03 | 1.23E-01 |

| | | | | |
|---|---|---|---|---|
| IFIT5 | 0.360 | Upregulated | 7.28E-03 | 1.12E-01 |
| TMEM154 | 0.360 | Upregulated | 1.89E-03 | 4.98E-02 |
| CASP9 | 0.361 | Upregulated | 3.64E-05 | 4.06E-03 |
| TMLHE | 0.361 | Upregulated | 1.19E-04 | 8.62E-03 |
| GNG8 | 0.361 | Upregulated | 1.69E-02 | 1.85E-01 |
| SUOX | 0.361 | Upregulated | 2.34E-04 | 1.27E-02 |
| GSDMD | 0.362 | Upregulated | 3.52E-04 | 1.64E-02 |
| NDUFAF3 | 0.362 | Upregulated | 4.49E-02 | 3.10E-01 |
| PRR13 | 0.362 | Upregulated | 5.78E-04 | 2.26E-02 |
| NAGK | 0.362 | Upregulated | 1.44E-03 | 4.24E-02 |
| CIDEB | 0.363 | Upregulated | 4.41E-03 | 8.39E-02 |
| NFKBIZ | 0.364 | Upregulated | 1.01E-05 | 2.15E-03 |
| LST1 | 0.364 | Upregulated | 5.11E-03 | 9.12E-02 |
| SKAP2 | 0.364 | Upregulated | 1.02E-02 | 1.39E-01 |
| LONRF3 | 0.364 | Upregulated | 3.84E-03 | 7.65E-02 |
| ANTXR2 | 0.364 | Upregulated | 1.70E-03 | 4.67E-02 |
| DISC1 | 0.364 | Upregulated | 3.03E-04 | 1.48E-02 |
| PLAC8 | 0.364 | Upregulated | 4.20E-03 | 8.12E-02 |
| LILRA6 | 0.365 | Upregulated | 8.16E-04 | 2.90E-02 |
| KIAA1618 | 0.365 | Upregulated | 7.64E-04 | 2.76E-02 |
| TNS3 | 0.365 | Upregulated | 1.27E-02 | 1.58E-01 |
| ERO1L | 0.366 | Upregulated | 1.34E-03 | 4.03E-02 |
| SIRT7 | 0.366 | Upregulated | 1.49E-03 | 4.36E-02 |
| CEACAM4 | 0.366 | Upregulated | 1.57E-02 | 1.77E-01 |
| EVI2A | 0.367 | Upregulated | 3.98E-02 | 2.92E-01 |
| DNTTIP1 | 0.367 | Upregulated | 6.88E-05 | 6.17E-03 |
| FAM26F | 0.367 | Upregulated | 3.63E-02 | 2.80E-01 |
| MTX1 | 0.367 | Upregulated | 1.03E-03 | 3.40E-02 |
| GBP3 | 0.368 | Upregulated | 8.88E-03 | 1.28E-01 |
| NUP214 | 0.368 | Upregulated | 1.68E-03 | 4.65E-02 |
| HIVEP1 | 0.368 | Upregulated | 9.56E-03 | 1.34E-01 |
| CMIP | 0.368 | Upregulated | 5.02E-04 | 2.07E-02 |
| CLEC4A | 0.368 | Upregulated | 1.68E-03 | 4.65E-02 |
| CSGALNACT2 | 0.369 | Upregulated | 4.07E-03 | 7.94E-02 |
| WWC3 | 0.369 | Upregulated | 1.73E-02 | 1.87E-01 |
| SLC35D3 | 0.369 | Upregulated | 3.68E-02 | 2.83E-01 |
| DHX8 | 0.370 | Upregulated | 3.05E-03 | 6.77E-02 |
| ODF3B | 0.370 | Upregulated | 1.35E-05 | 2.50E-03 |
| ZFAND3 | 0.371 | Upregulated | 4.74E-04 | 1.99E-02 |
| RHBDF2 | 0.371 | Upregulated | 7.73E-05 | 6.76E-03 |
| SLA | 0.371 | Upregulated | 3.36E-03 | 7.07E-02 |
| SCLT1 | 0.371 | Upregulated | 4.31E-03 | 8.26E-02 |
| TMEM91 | 0.372 | Upregulated | 2.74E-02 | 2.42E-01 |
| CD44 | 0.373 | Upregulated | 1.49E-03 | 4.34E-02 |
| NTSR1 | 0.373 | Upregulated | 3.44E-02 | 2.72E-01 |
| RASSF3 | 0.373 | Upregulated | 4.47E-03 | 8.47E-02 |
| NAPRT1 | 0.373 | Upregulated | 8.63E-04 | 2.99E-02 |
| IFI16 | 0.373 | Upregulated | 3.41E-04 | 1.60E-02 |
| MBOAT2 | 0.374 | Upregulated | 5.24E-03 | 9.20E-02 |
| ROS1 | 0.374 | Upregulated | 2.05E-02 | 2.06E-01 |
| HLA-B | 0.374 | Upregulated | 3.26E-05 | 3.81E-03 |

| | | | | |
|---|---|---|---|---|
| CARS2 | 0.374 | Upregulated | 2.10E-04 | 1.19E-02 |
| CREBBP | 0.375 | Upregulated | 1.25E-02 | 1.57E-01 |
| SCPEP1 | 0.375 | Upregulated | 1.17E-03 | 3.68E-02 |
| MOBKL1B | 0.375 | Upregulated | 7.24E-03 | 1.12E-01 |
| APBB1IP | 0.375 | Upregulated | 3.53E-04 | 1.64E-02 |
| FTH1 | 0.376 | Upregulated | 1.92E-02 | 1.99E-01 |
| ATG7 | 0.377 | Upregulated | 1.97E-03 | 5.13E-02 |
| DIRC2 | 0.377 | Upregulated | 9.85E-03 | 1.36E-01 |
| TFEB | 0.377 | Upregulated | 7.00E-04 | 2.59E-02 |
| SERPINB1 | 0.378 | Upregulated | 8.73E-03 | 1.26E-01 |
| RAF1 | 0.378 | Upregulated | 5.90E-06 | 1.69E-03 |
| LRP10 | 0.378 | Upregulated | 1.82E-04 | 1.10E-02 |
| TM9SF1 | 0.378 | Upregulated | 1.12E-04 | 8.31E-03 |
| FLII | 0.378 | Upregulated | 1.79E-03 | 4.83E-02 |
| IMPA2 | 0.378 | Upregulated | 2.09E-02 | 2.08E-01 |
| FYB | 0.378 | Upregulated | 1.16E-03 | 3.68E-02 |
| GPR108 | 0.378 | Upregulated | 1.21E-04 | 8.66E-03 |
| TNFAIP8L2 | 0.378 | Upregulated | 8.28E-05 | 6.99E-03 |
| MYOM1 | 0.378 | Upregulated | 2.71E-03 | 6.28E-02 |
| WSB1 | 0.379 | Upregulated | 8.50E-03 | 1.24E-01 |
| TRIM9 | 0.379 | Upregulated | 1.22E-02 | 1.55E-01 |
| SLC7A7 | 0.379 | Upregulated | 1.93E-03 | 5.07E-02 |
| PCNX | 0.379 | Upregulated | 1.25E-03 | 3.84E-02 |
| GAB2 | 0.379 | Upregulated | 3.58E-05 | 4.04E-03 |
| LYZ | 0.380 | Upregulated | 3.07E-02 | 2.56E-01 |
| RHOG | 0.380 | Upregulated | 8.22E-04 | 2.92E-02 |
| TRIM24 | 0.380 | Upregulated | 2.05E-03 | 5.26E-02 |
| OGFRL1 | 0.381 | Upregulated | 8.33E-04 | 2.94E-02 |
| TICAM2 | 0.381 | Upregulated | 2.28E-04 | 1.25E-02 |
| ABHD5 | 0.381 | Upregulated | 1.50E-02 | 1.73E-01 |
| ELMOD2 | 0.382 | Upregulated | 1.65E-03 | 4.60E-02 |
| FKBP5 | 0.382 | Upregulated | 1.53E-02 | 1.74E-01 |
| GLCE | 0.383 | Upregulated | 3.85E-03 | 7.67E-02 |
| KCND1 | 0.383 | Upregulated | 9.82E-04 | 3.29E-02 |
| TBKBP1 | 0.383 | Upregulated | 2.48E-02 | 2.28E-01 |
| TMEM49 | 0.383 | Upregulated | 1.59E-03 | 4.50E-02 |
| GNG5 | 0.383 | Upregulated | 7.08E-05 | 6.31E-03 |
| WAS | 0.383 | Upregulated | 4.57E-05 | 4.73E-03 |
| AGPAT2 | 0.384 | Upregulated | 2.31E-04 | 1.26E-02 |
| ADAM17 | 0.385 | Upregulated | 1.89E-05 | 2.95E-03 |
| MPP7 | 0.385 | Upregulated | 3.69E-03 | 7.44E-02 |
| SLC25A34 | 0.386 | Upregulated | 1.23E-02 | 1.55E-01 |
| ASAP1 | 0.387 | Upregulated | 2.56E-03 | 6.07E-02 |
| POR | 0.387 | Upregulated | 8.34E-03 | 1.23E-01 |
| CHMP2A | 0.387 | Upregulated | 5.17E-04 | 2.10E-02 |
| TOR1B | 0.387 | Upregulated | 6.90E-03 | 1.08E-01 |
| DCUN1D3 | 0.387 | Upregulated | 8.61E-05 | 7.13E-03 |
| MR1 | 0.387 | Upregulated | 3.65E-03 | 7.41E-02 |
| CD86 | 0.388 | Upregulated | 5.33E-03 | 9.31E-02 |
| ZBTB16 | 0.388 | Upregulated | 9.40E-03 | 1.32E-01 |
| PITPNA | 0.388 | Upregulated | 7.31E-03 | 1.13E-01 |

| | | | | |
|---|---|---|---|---|
| HS1BP3 | 0.388 | Upregulated | 4.25E-04 | 1.85E-02 |
| EFHD2 | 0.389 | Upregulated | 2.62E-04 | 1.36E-02 |
| FGR | 0.390 | Upregulated | 1.91E-03 | 5.01E-02 |
| TNFAIP3 | 0.390 | Upregulated | 1.63E-04 | 1.03E-02 |
| UGCG | 0.390 | Upregulated | 1.37E-02 | 1.65E-01 |
| HEBP1 | 0.390 | Upregulated | 8.43E-03 | 1.23E-01 |
| RCBTB2 | 0.390 | Upregulated | 3.89E-04 | 1.75E-02 |
| LONRF1 | 0.390 | Upregulated | 4.62E-03 | 8.62E-02 |
| DDIT3 | 0.391 | Upregulated | 7.03E-03 | 1.10E-01 |
| KCTD21 | 0.391 | Upregulated | 1.08E-04 | 8.13E-03 |
| PDK3 | 0.391 | Upregulated | 3.22E-04 | 1.56E-02 |
| EXT1 | 0.392 | Upregulated | 3.90E-03 | 7.74E-02 |
| ARAP3 | 0.392 | Upregulated | 6.56E-03 | 1.05E-01 |
| TDRD7 | 0.392 | Upregulated | 1.01E-03 | 3.35E-02 |
| ADCK4 | 0.392 | Upregulated | 8.83E-04 | 3.04E-02 |
| SRGN | 0.392 | Upregulated | 4.32E-05 | 4.55E-03 |
| NQO2 | 0.393 | Upregulated | 1.78E-02 | 1.90E-01 |
| MCL1 | 0.393 | Upregulated | 8.58E-06 | 1.98E-03 |
| APH1B | 0.393 | Upregulated | 9.86E-04 | 3.29E-02 |
| AKAP10 | 0.393 | Upregulated | 2.64E-03 | 6.17E-02 |
| PRDM1 | 0.393 | Upregulated | 8.69E-04 | 3.01E-02 |
| SELP | 0.393 | Upregulated | 3.91E-02 | 2.91E-01 |
| ANXA2 | 0.394 | Upregulated | 4.01E-03 | 7.87E-02 |
| ADCY4 | 0.394 | Upregulated | 2.00E-03 | 5.18E-02 |
| CASP8 | 0.394 | Upregulated | 1.31E-03 | 3.97E-02 |
| CST3 | 0.395 | Upregulated | 1.50E-02 | 1.73E-01 |
| ST8SIA4 | 0.395 | Upregulated | 7.76E-05 | 6.77E-03 |
| TK2 | 0.396 | Upregulated | 1.40E-04 | 9.55E-03 |
| MOSPD2 | 0.397 | Upregulated | 2.69E-03 | 6.25E-02 |
| CKLF | 0.397 | Upregulated | 2.15E-03 | 5.42E-02 |
| TBXAS1 | 0.397 | Upregulated | 1.17E-03 | 3.68E-02 |
| TMEM8 | 0.398 | Upregulated | 1.52E-03 | 4.40E-02 |
| SLC12A9 | 0.399 | Upregulated | 4.24E-04 | 1.85E-02 |
| CCNJL | 0.399 | Upregulated | 1.72E-02 | 1.87E-01 |
| CETP | 0.399 | Upregulated | 1.36E-03 | 4.07E-02 |
| MS4A14 | 0.399 | Upregulated | 1.50E-03 | 4.36E-02 |
| CBX8 | 0.400 | Upregulated | 1.09E-03 | 3.52E-02 |
| TCF7L2 | 0.400 | Upregulated | 3.07E-03 | 6.78E-02 |
| HLA-E | 0.401 | Upregulated | 1.60E-05 | 2.71E-03 |
| NLRP6 | 0.402 | Upregulated | 1.57E-03 | 4.47E-02 |
| SLC19A1 | 0.402 | Upregulated | 2.53E-03 | 6.03E-02 |
| PAK1 | 0.402 | Upregulated | 2.48E-04 | 1.32E-02 |
| BAGE5 | 0.402 | Upregulated | 3.12E-03 | 6.82E-02 |
| VAMP3 | 0.402 | Upregulated | 2.09E-04 | 1.19E-02 |
| HIST1H2AC | 0.402 | Upregulated | 1.65E-02 | 1.82E-01 |
| C3AR1 | 0.402 | Upregulated | 2.58E-02 | 2.34E-01 |
| NDEL1 | 0.403 | Upregulated | 2.47E-04 | 1.32E-02 |
| ZMAT5 | 0.403 | Upregulated | 1.36E-04 | 9.43E-03 |
| RAB34 | 0.404 | Upregulated | 4.55E-04 | 1.93E-02 |
| TMEM164 | 0.404 | Upregulated | 2.59E-05 | 3.50E-03 |
| HERC3 | 0.404 | Upregulated | 2.36E-03 | 5.80E-02 |

| | | | | |
|---|---|---|---|---|
| B3GNT8 | 0.404 | Upregulated | 1.44E-02 | 1.69E-01 |
| JMJD1C | 0.404 | Upregulated | 1.63E-03 | 4.56E-02 |
| UNC93B1 | 0.404 | Upregulated | 5.51E-03 | 9.46E-02 |
| LCP2 | 0.404 | Upregulated | 7.88E-05 | 6.78E-03 |
| C1GALT1C1 | 0.405 | Upregulated | 1.37E-03 | 4.10E-02 |
| HINT3 | 0.405 | Upregulated | 1.46E-04 | 9.84E-03 |
| PICALM | 0.405 | Upregulated | 6.67E-04 | 2.50E-02 |
| SF3B1 | 0.405 | Upregulated | 1.91E-02 | 1.98E-01 |
| RXRA | 0.405 | Upregulated | 1.86E-03 | 4.94E-02 |
| OGFR | 0.405 | Upregulated | 3.40E-05 | 3.91E-03 |
| IGFBP7 | 0.405 | Upregulated | 3.94E-04 | 1.77E-02 |
| ARHGEF11 | 0.405 | Upregulated | 5.52E-04 | 2.19E-02 |
| KLF4 | 0.405 | Upregulated | 1.30E-03 | 3.94E-02 |
| MAP1LC3B2 | 0.406 | Upregulated | 6.29E-04 | 2.40E-02 |
| MBD6 | 0.406 | Upregulated | 1.91E-05 | 2.96E-03 |
| SHISA5 | 0.406 | Upregulated | 1.29E-03 | 3.93E-02 |
| LPGAT1 | 0.407 | Upregulated | 8.97E-05 | 7.26E-03 |
| DHRS7B | 0.407 | Upregulated | 4.17E-04 | 1.84E-02 |
| B3GNTL1 | 0.407 | Upregulated | 4.68E-04 | 1.97E-02 |
| THOC5 | 0.407 | Upregulated | 6.68E-05 | 6.08E-03 |
| ANKRD33 | 0.407 | Upregulated | 7.78E-03 | 1.17E-01 |
| DLG4 | 0.407 | Upregulated | 2.19E-03 | 5.49E-02 |
| BAGE3 | 0.407 | Upregulated | 4.05E-03 | 7.92E-02 |
| HIPK3 | 0.407 | Upregulated | 2.17E-03 | 5.45E-02 |
| TUFT1 | 0.408 | Upregulated | 1.50E-02 | 1.73E-01 |
| LTA4H | 0.408 | Upregulated | 3.82E-03 | 7.63E-02 |
| CKAP4 | 0.408 | Upregulated | 3.90E-02 | 2.90E-01 |
| SOCS4 | 0.409 | Upregulated | 5.51E-03 | 9.46E-02 |
| NFE2L2 | 0.409 | Upregulated | 1.79E-04 | 1.08E-02 |
| PAPSS2 | 0.410 | Upregulated | 2.92E-03 | 6.61E-02 |
| CD63 | 0.411 | Upregulated | 1.32E-03 | 3.99E-02 |
| NLRP3 | 0.411 | Upregulated | 3.18E-03 | 6.89E-02 |
| ATG16L2 | 0.412 | Upregulated | 7.08E-04 | 2.61E-02 |
| GUCY1B3 | 0.412 | Upregulated | 5.58E-03 | 9.55E-02 |
| SLC15A4 | 0.413 | Upregulated | 1.77E-04 | 1.08E-02 |
| EPAS1 | 0.413 | Upregulated | 2.44E-02 | 2.27E-01 |
| OAS3 | 0.413 | Upregulated | 1.93E-02 | 1.99E-01 |
| TBC1D14 | 0.413 | Upregulated | 5.25E-04 | 2.12E-02 |
| FLJ42957 | 0.413 | Upregulated | 1.16E-02 | 1.50E-01 |
| RASGRP4 | 0.414 | Upregulated | 1.50E-04 | 1.00E-02 |
| STYXL1 | 0.414 | Upregulated | 2.86E-04 | 1.43E-02 |
| EPS15L1 | 0.415 | Upregulated | 1.98E-04 | 1.15E-02 |
| FLJ20309 | 0.415 | Upregulated | 1.48E-02 | 1.72E-01 |
| MARK2 | 0.416 | Upregulated | 9.21E-04 | 3.12E-02 |
| ALDH2 | 0.416 | Upregulated | 7.72E-03 | 1.17E-01 |
| HIP1 | 0.416 | Upregulated | 6.27E-03 | 1.03E-01 |
| MOBK1B | 0.417 | Upregulated | 6.76E-04 | 2.53E-02 |
| MS4A6A | 0.417 | Upregulated | 5.59E-03 | 9.57E-02 |
| GIT2 | 0.417 | Upregulated | 1.22E-04 | 8.71E-03 |
| LY6E | 0.417 | Upregulated | 2.60E-02 | 2.34E-01 |
| KIAA0232 | 0.418 | Upregulated | 9.14E-03 | 1.30E-01 |

| | | | | |
|---|---|---|---|---|
| NTN3 | 0.418 | Upregulated | 2.39E-04 | 1.29E-02 |
| OSGIN2 | 0.418 | Upregulated | 3.19E-05 | 3.77E-03 |
| TMEM55A | 0.419 | Upregulated | 4.39E-03 | 8.36E-02 |
| LGALS9 | 0.419 | Upregulated | 3.00E-04 | 1.47E-02 |
| ETV6 | 0.419 | Upregulated | 2.50E-04 | 1.33E-02 |
| IL10RB | 0.420 | Upregulated | 2.40E-04 | 1.29E-02 |
| IRF2 | 0.420 | Upregulated | 1.68E-04 | 1.05E-02 |
| B9D2 | 0.420 | Upregulated | 1.76E-05 | 2.83E-03 |
| CRK | 0.421 | Upregulated | 1.33E-04 | 9.27E-03 |
| HIST1H2AE | 0.421 | Upregulated | 7.71E-03 | 1.16E-01 |
| SAMSN1 | 0.421 | Upregulated | 1.17E-03 | 3.68E-02 |
| OBFC2A | 0.421 | Upregulated | 6.90E-04 | 2.57E-02 |
| M6PRBP1 | 0.421 | Upregulated | 2.76E-04 | 1.41E-02 |
| KLHL15 | 0.421 | Upregulated | 1.37E-02 | 1.65E-01 |
| CMTM6 | 0.422 | Upregulated | 2.05E-05 | 3.07E-03 |
| PLEK | 0.422 | Upregulated | 2.65E-04 | 1.37E-02 |
| SH2B2 | 0.422 | Upregulated | 4.06E-04 | 1.81E-02 |
| CSNK1D | 0.422 | Upregulated | 1.14E-05 | 2.30E-03 |
| SLC15A3 | 0.423 | Upregulated | 1.73E-04 | 1.06E-02 |
| TNFRSF1B | 0.424 | Upregulated | 9.88E-05 | 7.60E-03 |
| HRB | 0.424 | Upregulated | 1.03E-02 | 1.40E-01 |
| XKR3 | 0.424 | Upregulated | 1.50E-02 | 1.73E-01 |
| DHRS13 | 0.424 | Upregulated | 6.13E-03 | 1.01E-01 |
| RAB43 | 0.424 | Upregulated | 1.17E-04 | 8.54E-03 |
| TMCC3 | 0.424 | Upregulated | 5.48E-03 | 9.44E-02 |
| DNAJC25-GNG10 | 0.425 | Upregulated | 1.08E-05 | 2.23E-03 |
| OSBPL2 | 0.425 | Upregulated | 3.82E-04 | 1.73E-02 |
| SP110 | 0.426 | Upregulated | 3.68E-05 | 4.07E-03 |
| TCIRG1 | 0.426 | Upregulated | 3.87E-04 | 1.74E-02 |
| TMEM33 | 0.426 | Upregulated | 4.39E-03 | 8.37E-02 |
| EXOSC4 | 0.426 | Upregulated | 4.79E-03 | 8.82E-02 |
| BIN3 | 0.427 | Upregulated | 4.81E-04 | 2.01E-02 |
| PLAGL1 | 0.427 | Upregulated | 4.47E-05 | 4.65E-03 |
| ZNF350 | 0.427 | Upregulated | 6.38E-03 | 1.04E-01 |
| STAT1 | 0.428 | Upregulated | 5.03E-04 | 2.07E-02 |
| STAT2 | 0.428 | Upregulated | 1.68E-03 | 4.65E-02 |
| MIDN | 0.428 | Upregulated | 4.75E-05 | 4.85E-03 |
| ARID4A | 0.428 | Upregulated | 1.68E-03 | 4.65E-02 |
| QPCT | 0.429 | Upregulated | 7.56E-04 | 2.74E-02 |
| PLB1 | 0.429 | Upregulated | 1.78E-02 | 1.91E-01 |
| EFCBP1 | 0.429 | Upregulated | 8.58E-03 | 1.25E-01 |
| DUSP6 | 0.430 | Upregulated | 8.06E-04 | 2.87E-02 |
| OPLAH | 0.430 | Upregulated | 2.49E-02 | 2.29E-01 |
| TNFSF14 | 0.430 | Upregulated | 6.29E-03 | 1.03E-01 |
| NFE2 | 0.430 | Upregulated | 3.23E-02 | 2.63E-01 |
| CFP | 0.430 | Upregulated | 1.54E-03 | 4.44E-02 |
| VCAN | 0.431 | Upregulated | 1.55E-02 | 1.76E-01 |
| METRNL | 0.431 | Upregulated | 5.79E-04 | 2.26E-02 |
| GPER | 0.431 | Upregulated | 2.85E-02 | 2.47E-01 |
| P2RX7 | 0.431 | Upregulated | 1.80E-04 | 1.09E-02 |
| ITPK1 | 0.431 | Upregulated | 6.65E-05 | 6.08E-03 |

| | | | | |
|---|---|---|---|---|
| DDX6 | 0.431 | Upregulated | 2.03E-02 | 2.05E-01 |
| PDLIM7 | 0.431 | Upregulated | 1.79E-04 | 1.08E-02 |
| IGF1R | 0.431 | Upregulated | 1.86E-03 | 4.94E-02 |
| BAMBI | 0.432 | Upregulated | 3.52E-02 | 2.75E-01 |
| APOL2 | 0.432 | Upregulated | 4.49E-06 | 1.46E-03 |
| QKI | 0.432 | Upregulated | 1.32E-03 | 3.99E-02 |
| HDAC4 | 0.433 | Upregulated | 2.40E-02 | 2.25E-01 |
| PIK3AP1 | 0.433 | Upregulated | 1.65E-02 | 1.82E-01 |
| SLITRK4 | 0.433 | Upregulated | 5.07E-04 | 2.08E-02 |
| XAF1 | 0.433 | Upregulated | 2.85E-02 | 2.47E-01 |
| C1QA | 0.434 | Upregulated | 7.86E-04 | 2.81E-02 |
| CCDC17 | 0.434 | Upregulated | 6.42E-04 | 2.44E-02 |
| ASGR1 | 0.435 | Upregulated | 8.52E-04 | 2.98E-02 |
| MYD88 | 0.435 | Upregulated | 1.91E-04 | 1.12E-02 |
| CBS | 0.435 | Upregulated | 4.84E-03 | 8.86E-02 |
| LMO2 | 0.436 | Upregulated | 7.97E-03 | 1.18E-01 |
| DHRSX | 0.436 | Upregulated | 1.85E-04 | 1.11E-02 |
| GPR27 | 0.437 | Upregulated | 5.97E-05 | 5.70E-03 |
| CLDN23 | 0.438 | Upregulated | 5.62E-03 | 9.60E-02 |
| TMC4 | 0.438 | Upregulated | 1.31E-03 | 3.98E-02 |
| SESTD1 | 0.439 | Upregulated | 5.67E-06 | 1.65E-03 |
| MXD3 | 0.440 | Upregulated | 3.76E-03 | 7.56E-02 |
| GPBAR1 | 0.440 | Upregulated | 1.94E-02 | 2.00E-01 |
| FAM176B | 0.440 | Upregulated | 1.61E-04 | 1.03E-02 |
| SNX27 | 0.441 | Upregulated | 3.63E-05 | 4.06E-03 |
| CXCL16 | 0.441 | Upregulated | 1.12E-04 | 8.31E-03 |
| IFIH1 | 0.441 | Upregulated | 2.31E-03 | 5.70E-02 |
| PXK | 0.441 | Upregulated | 1.51E-03 | 4.39E-02 |
| PTPRC | 0.442 | Upregulated | 1.08E-02 | 1.44E-01 |
| FAS | 0.444 | Upregulated | 3.41E-04 | 1.60E-02 |
| IFNGR2 | 0.444 | Upregulated | 6.22E-04 | 2.38E-02 |
| HTATIP2 | 0.445 | Upregulated | 1.44E-05 | 2.58E-03 |
| FKBP15 | 0.445 | Upregulated | 7.45E-05 | 6.55E-03 |
| SMAP2 | 0.446 | Upregulated | 2.30E-04 | 1.26E-02 |
| KLHL21 | 0.447 | Upregulated | 1.21E-03 | 3.75E-02 |
| SLC16A5 | 0.449 | Upregulated | 5.05E-04 | 2.07E-02 |
| GLDN | 0.450 | Upregulated | 8.51E-03 | 1.24E-01 |
| PSMB9 | 0.450 | Upregulated | 3.63E-05 | 4.06E-03 |
| LRPAP1 | 0.450 | Upregulated | 1.14E-03 | 3.64E-02 |
| CHSY1 | 0.450 | Upregulated | 2.10E-03 | 5.33E-02 |
| CTBS | 0.450 | Upregulated | 4.91E-04 | 2.04E-02 |
| GAA | 0.450 | Upregulated | 7.92E-04 | 2.83E-02 |
| SLC11A1 | 0.450 | Upregulated | 3.23E-03 | 6.94E-02 |
| CCNA1 | 0.450 | Upregulated | 3.16E-02 | 2.60E-01 |
| CCDC151 | 0.450 | Upregulated | 8.67E-04 | 3.00E-02 |
| ITGA5 | 0.451 | Upregulated | 3.26E-04 | 1.56E-02 |
| CD55 | 0.451 | Upregulated | 1.16E-03 | 3.67E-02 |
| LRP1 | 0.451 | Upregulated | 6.50E-03 | 1.05E-01 |
| CASP4 | 0.452 | Upregulated | 9.66E-06 | 2.09E-03 |
| AKIRIN2 | 0.452 | Upregulated | 3.76E-05 | 4.14E-03 |
| CTBP2 | 0.453 | Upregulated | 1.31E-05 | 2.48E-03 |

| | | | | |
|---|---|---|---|---|
| DDX58 | 0.453 | Upregulated | 1.13E-03 | 3.61E-02 |
| HEMK1 | 0.453 | Upregulated | 4.33E-05 | 4.55E-03 |
| ACOT9 | 0.453 | Upregulated | 1.35E-04 | 9.37E-03 |
| LOXL3 | 0.453 | Upregulated | 3.22E-03 | 6.94E-02 |
| COL4A3BP | 0.454 | Upregulated | 3.06E-04 | 1.50E-02 |
| SETX | 0.454 | Upregulated | 6.72E-05 | 6.10E-03 |
| DTX2 | 0.454 | Upregulated | 3.05E-06 | 1.28E-03 |
| MAP4K4 | 0.454 | Upregulated | 7.45E-04 | 2.71E-02 |
| MAP1A | 0.455 | Upregulated | 1.26E-02 | 1.57E-01 |
| SLC45A4 | 0.455 | Upregulated | 5.69E-04 | 2.23E-02 |
| GNAI3 | 0.456 | Upregulated | 3.17E-04 | 1.53E-02 |
| FLOT2 | 0.456 | Upregulated | 3.00E-04 | 1.47E-02 |
| TFPI | 0.457 | Upregulated | 1.63E-02 | 1.81E-01 |
| RTN3 | 0.457 | Upregulated | 6.44E-03 | 1.04E-01 |
| ALOX5AP | 0.458 | Upregulated | 7.11E-04 | 2.62E-02 |
| KIAA0556 | 0.458 | Upregulated | 6.48E-06 | 1.77E-03 |
| SOS2 | 0.459 | Upregulated | 1.90E-03 | 5.00E-02 |
| CTNNA1 | 0.459 | Upregulated | 8.42E-04 | 2.97E-02 |
| RBCK1 | 0.459 | Upregulated | 3.62E-06 | 1.32E-03 |
| ATP6V1B2 | 0.459 | Upregulated | 1.26E-05 | 2.48E-03 |
| CBARA1 | 0.460 | Upregulated | 2.13E-04 | 1.20E-02 |
| CPVL | 0.461 | Upregulated | 2.07E-03 | 5.29E-02 |
| AQP12A | 0.461 | Upregulated | 3.24E-05 | 3.81E-03 |
| RENBP | 0.461 | Upregulated | 2.54E-05 | 3.48E-03 |
| MICB | 0.461 | Upregulated | 3.81E-06 | 1.36E-03 |
| KCTD14 | 0.461 | Upregulated | 2.85E-02 | 2.47E-01 |
| NDST1 | 0.461 | Upregulated | 1.62E-04 | 1.03E-02 |
| CNOT3 | 0.462 | Upregulated | 1.35E-03 | 4.06E-02 |
| RIN2 | 0.462 | Upregulated | 2.60E-02 | 2.35E-01 |
| SPTLC2 | 0.462 | Upregulated | 1.51E-04 | 1.00E-02 |
| NLRP12 | 0.462 | Upregulated | 8.23E-03 | 1.21E-01 |
| ELMO2 | 0.463 | Upregulated | 1.71E-03 | 4.67E-02 |
| KRT23 | 0.464 | Upregulated | 8.97E-05 | 7.26E-03 |
| EDG4 | 0.464 | Upregulated | 2.52E-04 | 1.33E-02 |
| DHRS12 | 0.464 | Upregulated | 8.73E-05 | 7.18E-03 |
| SHKBP1 | 0.464 | Upregulated | 4.00E-04 | 1.79E-02 |
| RBMS1 | 0.465 | Upregulated | 2.22E-03 | 5.54E-02 |
| ATP6V0D1 | 0.466 | Upregulated | 3.30E-04 | 1.57E-02 |
| HAL | 0.466 | Upregulated | 5.26E-03 | 9.23E-02 |
| GGA3 | 0.466 | Upregulated | 2.82E-04 | 1.42E-02 |
| EMR1 | 0.467 | Upregulated | 3.65E-02 | 2.81E-01 |
| PPP1R15A | 0.467 | Upregulated | 2.53E-04 | 1.33E-02 |
| CD33 | 0.467 | Upregulated | 1.56E-03 | 4.47E-02 |
| MLKL | 0.468 | Upregulated | 5.98E-05 | 5.70E-03 |
| ASPHD2 | 0.468 | Upregulated | 3.72E-06 | 1.34E-03 |
| TMEM51 | 0.468 | Upregulated | 6.55E-03 | 1.05E-01 |
| PRRG4 | 0.469 | Upregulated | 8.32E-05 | 6.99E-03 |
| RILPL2 | 0.469 | Upregulated | 6.07E-05 | 5.73E-03 |
| ADAM8 | 0.469 | Upregulated | 2.77E-04 | 1.41E-02 |
| RIT1 | 0.470 | Upregulated | 6.34E-05 | 5.89E-03 |
| PREX1 | 0.470 | Upregulated | 2.67E-03 | 6.21E-02 |

| | | | | |
|---|---|---|---|---|
| CYBB | 0.470 | Upregulated | 2.82E-05 | 3.61E-03 |
| NETO2 | 0.471 | Upregulated | 2.86E-03 | 6.48E-02 |
| TOM1 | 0.471 | Upregulated | 2.78E-05 | 3.61E-03 |
| FAM20A | 0.471 | Upregulated | 1.23E-02 | 1.55E-01 |
| ACTN1 | 0.471 | Upregulated | 3.28E-04 | 1.56E-02 |
| DTNBP1 | 0.473 | Upregulated | 1.49E-04 | 9.95E-03 |
| ABCD1 | 0.473 | Upregulated | 3.16E-04 | 1.53E-02 |
| CDS2 | 0.473 | Upregulated | 5.03E-05 | 5.03E-03 |
| MAP3K11 | 0.473 | Upregulated | 1.54E-04 | 1.01E-02 |
| TMEM144 | 0.474 | Upregulated | 6.28E-03 | 1.03E-01 |
| FRMD3 | 0.474 | Upregulated | 3.88E-03 | 7.70E-02 |
| MYADM | 0.475 | Upregulated | 6.55E-03 | 1.05E-01 |
| SULT1B1 | 0.475 | Upregulated | 7.19E-03 | 1.11E-01 |
| STAT3 | 0.476 | Upregulated | 2.19E-05 | 3.21E-03 |
| RNASEL | 0.476 | Upregulated | 1.06E-04 | 8.04E-03 |
| MX2 | 0.477 | Upregulated | 1.15E-02 | 1.49E-01 |
| FCGR2C | 0.477 | Upregulated | 6.99E-04 | 2.59E-02 |
| SIGLEC7 | 0.477 | Upregulated | 2.59E-03 | 6.10E-02 |
| AIG1 | 0.478 | Upregulated | 9.53E-04 | 3.22E-02 |
| GCA | 0.478 | Upregulated | 7.22E-03 | 1.12E-01 |
| SIRPD | 0.479 | Upregulated | 3.34E-03 | 7.06E-02 |
| SELPLG | 0.479 | Upregulated | 3.96E-03 | 7.80E-02 |
| TRIB1 | 0.479 | Upregulated | 2.47E-03 | 5.95E-02 |
| IFNGR1 | 0.480 | Upregulated | 3.71E-04 | 1.69E-02 |
| NUMB | 0.480 | Upregulated | 2.88E-04 | 1.43E-02 |
| DDAH2 | 0.480 | Upregulated | 7.03E-04 | 2.60E-02 |
| LBR | 0.480 | Upregulated | 8.73E-03 | 1.26E-01 |
| HIF1A | 0.480 | Upregulated | 9.07E-03 | 1.30E-01 |
| ZNF397 | 0.480 | Upregulated | 3.29E-04 | 1.56E-02 |
| JAK2 | 0.480 | Upregulated | 2.28E-04 | 1.25E-02 |
| MAK | 0.481 | Upregulated | 1.51E-02 | 1.73E-01 |
| ORAI2 | 0.481 | Upregulated | 1.76E-03 | 4.76E-02 |
| CYTH4 | 0.481 | Upregulated | 2.94E-05 | 3.69E-03 |
| POLB | 0.481 | Upregulated | 3.27E-08 | 6.68E-05 |
| KIAA0319L | 0.481 | Upregulated | 1.07E-04 | 8.05E-03 |
| STARD10 | 0.482 | Upregulated | 9.40E-05 | 7.41E-03 |
| RHOT1 | 0.483 | Upregulated | 6.12E-06 | 1.73E-03 |
| CDC42EP3 | 0.484 | Upregulated | 6.82E-03 | 1.08E-01 |
| MOSC1 | 0.484 | Upregulated | 3.21E-02 | 2.62E-01 |
| ATF6 | 0.485 | Upregulated | 1.70E-06 | 9.07E-04 |
| ZBP1 | 0.486 | Upregulated | 4.82E-03 | 8.85E-02 |
| GRB2 | 0.486 | Upregulated | 1.59E-04 | 1.02E-02 |
| GM2A | 0.486 | Upregulated | 9.54E-04 | 3.22E-02 |
| LPPR2 | 0.486 | Upregulated | 8.25E-04 | 2.92E-02 |
| ATF5 | 0.486 | Upregulated | 3.65E-03 | 7.42E-02 |
| ACSL4 | 0.487 | Upregulated | 1.68E-04 | 1.05E-02 |
| SSH1 | 0.488 | Upregulated | 1.69E-03 | 4.65E-02 |
| VASP | 0.488 | Upregulated | 1.55E-05 | 2.66E-03 |
| TRAFD1 | 0.489 | Upregulated | 1.65E-05 | 2.75E-03 |
| PHF21A | 0.489 | Upregulated | 2.35E-05 | 3.30E-03 |
| NACC2 | 0.490 | Upregulated | 4.30E-04 | 1.86E-02 |

| | | | | |
|---|---|---|---|---|
| GLIPR2 | 0.490 | Upregulated | 1.41E-05 | 2.57E-03 |
| KIAA0367 | 0.490 | Upregulated | 4.66E-02 | 3.16E-01 |
| TAP2 | 0.490 | Upregulated | 1.82E-05 | 2.89E-03 |
| ZNFX1 | 0.491 | Upregulated | 4.27E-05 | 4.55E-03 |
| MVP | 0.491 | Upregulated | 1.49E-05 | 2.63E-03 |
| PYCARD | 0.491 | Upregulated | 4.78E-05 | 4.86E-03 |
| ZYX | 0.491 | Upregulated | 3.39E-04 | 1.60E-02 |
| PARP10 | 0.492 | Upregulated | 2.03E-04 | 1.17E-02 |
| NFAT5 | 0.493 | Upregulated | 2.31E-03 | 5.70E-02 |
| TSPO | 0.493 | Upregulated | 1.80E-03 | 4.84E-02 |
| GNA15 | 0.494 | Upregulated | 5.94E-04 | 2.30E-02 |
| CFLAR | 0.494 | Upregulated | 9.49E-04 | 3.21E-02 |
| ACTA2 | 0.494 | Upregulated | 6.62E-03 | 1.06E-01 |
| HSD3B7 | 0.495 | Upregulated | 7.36E-03 | 1.13E-01 |
| CTSL1 | 0.495 | Upregulated | 7.02E-03 | 1.09E-01 |
| GRAMD1A | 0.495 | Upregulated | 1.14E-04 | 8.40E-03 |
| PRIC285 | 0.495 | Upregulated | 5.75E-03 | 9.73E-02 |
| PARP14 | 0.495 | Upregulated | 1.76E-04 | 1.07E-02 |
| PGCP | 0.496 | Upregulated | 1.56E-03 | 4.47E-02 |
| LILRB3 | 0.496 | Upregulated | 2.48E-05 | 3.40E-03 |
| SLC46A2 | 0.496 | Upregulated | 1.40E-03 | 4.16E-02 |
| GUCY1A3 | 0.496 | Upregulated | 4.08E-04 | 1.82E-02 |
| SLC25A44 | 0.497 | Upregulated | 1.12E-04 | 8.32E-03 |
| RAB31 | 0.497 | Upregulated | 3.24E-03 | 6.94E-02 |
| HIST1H4D | 0.497 | Upregulated | 6.41E-03 | 1.04E-01 |
| TMEM149 | 0.498 | Upregulated | 2.68E-05 | 3.57E-03 |
| ECHDC3 | 0.498 | Upregulated | 2.13E-02 | 2.11E-01 |
| TMEM127 | 0.500 | Upregulated | 5.62E-04 | 2.22E-02 |
| RAB32 | 0.500 | Upregulated | 3.66E-03 | 7.42E-02 |
| FCN1 | 0.500 | Upregulated | 1.60E-04 | 1.02E-02 |
| AOAH | 0.501 | Upregulated | 1.39E-04 | 9.46E-03 |
| LPAR2 | 0.502 | Upregulated | 2.35E-04 | 1.27E-02 |
| PSCD4 | 0.502 | Upregulated | 8.24E-06 | 1.97E-03 |
| SLC22A4 | 0.505 | Upregulated | 3.75E-02 | 2.85E-01 |
| ADAP2 | 0.505 | Upregulated | 5.91E-04 | 2.30E-02 |
| IFNAR1 | 0.505 | Upregulated | 1.51E-04 | 1.00E-02 |
| PRAM1 | 0.506 | Upregulated | 1.98E-04 | 1.15E-02 |
| TM6SF1 | 0.506 | Upregulated | 2.54E-03 | 6.03E-02 |
| OAS1 | 0.507 | Upregulated | 3.16E-02 | 2.60E-01 |
| TBC1D2 | 0.507 | Upregulated | 5.03E-04 | 2.07E-02 |
| TBC1D24 | 0.509 | Upregulated | 1.52E-04 | 1.01E-02 |
| MPZL1 | 0.510 | Upregulated | 8.79E-03 | 1.27E-01 |
| NBN | 0.510 | Upregulated | 4.09E-04 | 1.82E-02 |
| DENND5A | 0.511 | Upregulated | 8.99E-06 | 1.99E-03 |
| KIAA0040 | 0.511 | Upregulated | 8.49E-05 | 7.08E-03 |
| PTGS2 | 0.511 | Upregulated | 6.85E-03 | 1.08E-01 |
| AKAP13 | 0.513 | Upregulated | 3.34E-05 | 3.85E-03 |
| CD300LB | 0.513 | Upregulated | 1.80E-03 | 4.84E-02 |
| HAUS4 | 0.513 | Upregulated | 6.14E-04 | 2.37E-02 |
| CD300LF | 0.514 | Upregulated | 1.21E-03 | 3.75E-02 |
| TMEM180 | 0.515 | Upregulated | 2.52E-04 | 1.33E-02 |

| | | | | |
|---|---|---|---|---|
| SLCO3A1 | 0.515 | Upregulated | 1.99E-05 | 3.02E-03 |
| BAZ1A | 0.515 | Upregulated | 2.95E-06 | 1.28E-03 |
| MOV10 | 0.515 | Upregulated | 3.50E-04 | 1.63E-02 |
| CATSPER1 | 0.516 | Upregulated | 1.48E-03 | 4.34E-02 |
| SPATA13 | 0.517 | Upregulated | 1.75E-02 | 1.89E-01 |
| LILRB2 | 0.517 | Upregulated | 1.38E-04 | 9.46E-03 |
| DYNLT1 | 0.517 | Upregulated | 1.30E-04 | 9.14E-03 |
| PPP1R3B | 0.518 | Upregulated | 2.04E-05 | 3.07E-03 |
| TLR1 | 0.520 | Upregulated | 4.21E-02 | 3.01E-01 |
| FAR2 | 0.520 | Upregulated | 2.29E-04 | 1.26E-02 |
| GNB4 | 0.520 | Upregulated | 2.72E-04 | 1.40E-02 |
| RNF130 | 0.520 | Upregulated | 1.71E-03 | 4.67E-02 |
| CMPK2 | 0.521 | Upregulated | 1.94E-02 | 2.00E-01 |
| TMEM120A | 0.521 | Upregulated | 7.85E-05 | 6.78E-03 |
| CTSA | 0.521 | Upregulated | 8.47E-04 | 2.98E-02 |
| CASP1 | 0.521 | Upregulated | 9.49E-05 | 7.45E-03 |
| MAFB | 0.521 | Upregulated | 1.86E-03 | 4.94E-02 |
| C5AR1 | 0.521 | Upregulated | 5.46E-03 | 9.43E-02 |
| PACSIN2 | 0.522 | Upregulated | 4.32E-05 | 4.55E-03 |
| EMILIN2 | 0.522 | Upregulated | 5.17E-04 | 2.10E-02 |
| ARHGAP27 | 0.523 | Upregulated | 1.25E-03 | 3.84E-02 |
| DENND1A | 0.524 | Upregulated | 8.64E-05 | 7.14E-03 |
| PHTF1 | 0.524 | Upregulated | 1.08E-03 | 3.49E-02 |
| ZFP36 | 0.524 | Upregulated | 1.99E-05 | 3.02E-03 |
| GADD45G | 0.524 | Upregulated | 3.59E-05 | 4.04E-03 |
| IRAK2 | 0.524 | Upregulated | 1.17E-04 | 8.52E-03 |
| ZNF429 | 0.525 | Upregulated | 2.73E-03 | 6.31E-02 |
| SAT1 | 0.525 | Upregulated | 9.05E-06 | 1.99E-03 |
| TSEN34 | 0.525 | Upregulated | 9.08E-05 | 7.26E-03 |
| CPEB4 | 0.526 | Upregulated | 7.65E-03 | 1.16E-01 |
| SELL | 0.526 | Upregulated | 3.10E-05 | 3.74E-03 |
| MON1B | 0.526 | Upregulated | 7.24E-04 | 2.65E-02 |
| KLHDC8B | 0.526 | Upregulated | 5.15E-03 | 9.13E-02 |
| RAB3D | 0.526 | Upregulated | 4.61E-05 | 4.76E-03 |
| GBA | 0.526 | Upregulated | 1.09E-04 | 8.15E-03 |
| LBA1 | 0.527 | Upregulated | 4.96E-06 | 1.55E-03 |
| PELI1 | 0.527 | Upregulated | 1.31E-05 | 2.48E-03 |
| LITAF | 0.527 | Upregulated | 1.09E-05 | 2.24E-03 |
| PTPRJ | 0.527 | Upregulated | 1.42E-04 | 9.67E-03 |
| SIGLEC9 | 0.528 | Upregulated | 3.15E-03 | 6.87E-02 |
| IGF2R | 0.529 | Upregulated | 5.52E-04 | 2.19E-02 |
| TDRD9 | 0.530 | Upregulated | 3.46E-02 | 2.73E-01 |
| CHST15 | 0.530 | Upregulated | 5.48E-04 | 2.19E-02 |
| HIST1H2BK | 0.530 | Upregulated | 3.36E-04 | 1.58E-02 |
| KIAA0247 | 0.530 | Upregulated | 2.70E-05 | 3.58E-03 |
| SLC2A3 | 0.532 | Upregulated | 4.78E-04 | 1.99E-02 |
| DTX3L | 0.532 | Upregulated | 4.19E-04 | 1.84E-02 |
| HERC5 | 0.533 | Upregulated | 3.82E-02 | 2.87E-01 |
| PTPRE | 0.534 | Upregulated | 5.24E-06 | 1.58E-03 |
| GNS | 0.534 | Upregulated | 3.55E-05 | 4.03E-03 |
| MRVI1 | 0.534 | Upregulated | 6.66E-05 | 6.08E-03 |

| | | | | |
|---|---|---|---|---|
| CTSS | 0.534 | Upregulated | 2.96E-05 | 3.69E-03 |
| MTF1 | 0.535 | Upregulated | 2.53E-04 | 1.33E-02 |
| BMX | 0.535 | Upregulated | 6.74E-03 | 1.07E-01 |
| SLC9A8 | 0.535 | Upregulated | 1.57E-04 | 1.02E-02 |
| PYGL | 0.536 | Upregulated | 8.98E-03 | 1.29E-01 |
| IFITM2 | 0.537 | Upregulated | 4.80E-06 | 1.54E-03 |
| NAIP | 0.537 | Upregulated | 5.53E-04 | 2.19E-02 |
| RRAGD | 0.539 | Upregulated | 4.50E-03 | 8.50E-02 |
| PPP1R3D | 0.541 | Upregulated | 5.00E-04 | 2.06E-02 |
| DGAT2 | 0.541 | Upregulated | 2.48E-03 | 5.95E-02 |
| RSPH3 | 0.541 | Upregulated | 1.12E-05 | 2.28E-03 |
| IL17RA | 0.541 | Upregulated | 7.82E-04 | 2.81E-02 |
| MYO1F | 0.541 | Upregulated | 1.32E-04 | 9.19E-03 |
| CTRL | 0.543 | Upregulated | 1.47E-04 | 9.84E-03 |
| CLEC7A | 0.544 | Upregulated | 9.08E-04 | 3.11E-02 |
| ITPRIPL2 | 0.544 | Upregulated | 2.17E-03 | 5.45E-02 |
| PLEKHO2 | 0.545 | Upregulated | 2.09E-05 | 3.08E-03 |
| ITGAM | 0.545 | Upregulated | 3.20E-03 | 6.93E-02 |
| GYG1 | 0.546 | Upregulated | 8.93E-03 | 1.28E-01 |
| PSG3 | 0.547 | Upregulated | 7.82E-06 | 1.91E-03 |
| SNX10 | 0.547 | Upregulated | 4.90E-03 | 8.92E-02 |
| ITGAX | 0.547 | Upregulated | 2.07E-04 | 1.18E-02 |
| IGF2BP3 | 0.548 | Upregulated | 5.15E-03 | 9.13E-02 |
| AIM2 | 0.549 | Upregulated | 1.84E-04 | 1.10E-02 |
| LTB4R | 0.550 | Upregulated | 2.06E-03 | 5.28E-02 |
| AMICA1 | 0.551 | Upregulated | 7.71E-05 | 6.75E-03 |
| NINJ1 | 0.552 | Upregulated | 9.74E-04 | 3.26E-02 |
| EPHB1 | 0.552 | Upregulated | 1.05E-03 | 3.44E-02 |
| AIF1 | 0.553 | Upregulated | 1.51E-04 | 1.00E-02 |
| IL1F9 | 0.554 | Upregulated | 2.57E-04 | 1.35E-02 |
| PTAFR | 0.554 | Upregulated | 1.17E-03 | 3.68E-02 |
| GHRL | 0.554 | Upregulated | 2.40E-04 | 1.29E-02 |
| ERLIN1 | 0.557 | Upregulated | 1.21E-04 | 8.66E-03 |
| CCRL2 | 0.557 | Upregulated | 4.94E-04 | 2.05E-02 |
| NRBF2 | 0.557 | Upregulated | 3.19E-05 | 3.77E-03 |
| RNF13 | 0.559 | Upregulated | 1.56E-06 | 8.46E-04 |
| FADS1 | 0.559 | Upregulated | 9.44E-03 | 1.33E-01 |
| HECW2 | 0.559 | Upregulated | 9.17E-04 | 3.12E-02 |
| CD300C | 0.559 | Upregulated | 1.83E-03 | 4.88E-02 |
| PISD | 0.560 | Upregulated | 2.44E-05 | 3.36E-03 |
| NFKB2 | 0.561 | Upregulated | 8.56E-06 | 1.98E-03 |
| SEMA4A | 0.561 | Upregulated | 1.83E-03 | 4.89E-02 |
| SH3GLB1 | 0.562 | Upregulated | 3.54E-04 | 1.64E-02 |
| IFITM1 | 0.562 | Upregulated | 6.21E-05 | 5.85E-03 |
| PELI2 | 0.562 | Upregulated | 5.51E-05 | 5.40E-03 |
| TAPBP | 0.563 | Upregulated | 3.04E-06 | 1.28E-03 |
| PGD | 0.564 | Upregulated | 2.86E-03 | 6.48E-02 |
| GBP2 | 0.564 | Upregulated | 6.31E-06 | 1.74E-03 |
| GLT1D1 | 0.564 | Upregulated | 7.79E-04 | 2.80E-02 |
| IL15 | 0.564 | Upregulated | 1.74E-06 | 9.17E-04 |
| TSHZ3 | 0.564 | Upregulated | 4.29E-03 | 8.25E-02 |

| | | | | |
|---|---|---|---|---|
| CAPNS2 | 0.566 | Upregulated | 1.32E-05 | 2.48E-03 |
| PARP9 | 0.567 | Upregulated | 9.35E-05 | 7.39E-03 |
| SDCBP | 0.567 | Upregulated | 6.03E-04 | 2.33E-02 |
| HCG9 | 0.568 | Upregulated | 1.83E-03 | 4.89E-02 |
| WDFY3 | 0.569 | Upregulated | 3.89E-05 | 4.26E-03 |
| NCF2 | 0.569 | Upregulated | 8.41E-04 | 2.97E-02 |
| RELB | 0.569 | Upregulated | 3.66E-07 | 4.24E-04 |
| OASL | 0.571 | Upregulated | 1.99E-02 | 2.03E-01 |
| SAMD4A | 0.571 | Upregulated | 4.52E-04 | 1.93E-02 |
| UBTD1 | 0.571 | Upregulated | 1.08E-03 | 3.49E-02 |
| UBE2L6 | 0.572 | Upregulated | 6.31E-05 | 5.89E-03 |
| KCNE3 | 0.573 | Upregulated | 1.75E-05 | 2.83E-03 |
| ITPRIP | 0.573 | Upregulated | 4.87E-06 | 1.55E-03 |
| CLEC1A | 0.573 | Upregulated | 1.66E-05 | 2.76E-03 |
| TYROBP | 0.574 | Upregulated | 2.69E-05 | 3.57E-03 |
| IL4R | 0.574 | Upregulated | 8.99E-04 | 3.08E-02 |
| LAT2 | 0.575 | Upregulated | 4.14E-05 | 4.47E-03 |
| HIST2H2AB | 0.575 | Upregulated | 6.57E-04 | 2.48E-02 |
| ERI1 | 0.576 | Upregulated | 9.84E-04 | 3.29E-02 |
| UBXN2B | 0.576 | Upregulated | 1.28E-04 | 9.07E-03 |
| LGALS3BP | 0.578 | Upregulated | 5.08E-03 | 9.10E-02 |
| IFI35 | 0.578 | Upregulated | 3.24E-04 | 1.56E-02 |
| GALM | 0.579 | Upregulated | 8.95E-04 | 3.07E-02 |
| GRN | 0.579 | Upregulated | 5.14E-04 | 2.09E-02 |
| CXCL1 | 0.579 | Upregulated | 1.26E-04 | 8.91E-03 |
| KIAA1598 | 0.580 | Upregulated | 3.78E-03 | 7.59E-02 |
| MARCKS | 0.580 | Upregulated | 3.14E-05 | 3.77E-03 |
| CPD | 0.580 | Upregulated | 3.32E-04 | 1.58E-02 |
| ST3GAL6 | 0.581 | Upregulated | 7.84E-04 | 2.81E-02 |
| PFKFB3 | 0.582 | Upregulated | 3.09E-03 | 6.79E-02 |
| CYP4F3 | 0.583 | Upregulated | 3.33E-02 | 2.67E-01 |
| OSCAR | 0.583 | Upregulated | 3.03E-03 | 6.75E-02 |
| SORL1 | 0.583 | Upregulated | 1.24E-03 | 3.82E-02 |
| TRIM5 | 0.585 | Upregulated | 5.50E-04 | 2.19E-02 |
| ZNF200 | 0.586 | Upregulated | 2.86E-06 | 1.26E-03 |
| FLOT1 | 0.587 | Upregulated | 1.87E-04 | 1.11E-02 |
| ZC3H12A | 0.588 | Upregulated | 1.64E-04 | 1.03E-02 |
| FLVCR2 | 0.588 | Upregulated | 3.23E-04 | 1.56E-02 |
| SQRDL | 0.590 | Upregulated | 4.17E-05 | 4.47E-03 |
| HLX | 0.590 | Upregulated | 8.39E-04 | 2.96E-02 |
| SIRPB1 | 0.591 | Upregulated | 7.79E-03 | 1.17E-01 |
| TREML2 | 0.592 | Upregulated | 3.02E-05 | 3.70E-03 |
| CTRC | 0.593 | Upregulated | 2.42E-05 | 3.36E-03 |
| SAMHD1 | 0.595 | Upregulated | 1.69E-04 | 1.05E-02 |
| ZAK | 0.595 | Upregulated | 2.75E-03 | 6.34E-02 |
| GBP4 | 0.595 | Upregulated | 7.95E-05 | 6.81E-03 |
| INDO | 0.596 | Upregulated | 1.44E-02 | 1.69E-01 |
| TRIM21 | 0.597 | Upregulated | 8.24E-07 | 6.63E-04 |
| RERE | 0.599 | Upregulated | 5.16E-05 | 5.12E-03 |
| KDM6B | 0.600 | Upregulated | 8.62E-06 | 1.98E-03 |
| FLJ14166 | 0.600 | Upregulated | 2.34E-04 | 1.27E-02 |

| | | | | |
|---|---|---|---|---|
| TBC1D8 | 0.600 | Upregulated | 4.36E-04 | 1.88E-02 |
| S100A11 | 0.601 | Upregulated | 4.28E-04 | 1.86E-02 |
| IER3 | 0.601 | Upregulated | 1.16E-03 | 3.68E-02 |
| EIF4E3 | 0.601 | Upregulated | 2.85E-05 | 3.61E-03 |
| CD14 | 0.602 | Upregulated | 6.01E-04 | 2.33E-02 |
| MEFV | 0.602 | Upregulated | 2.80E-04 | 1.42E-02 |
| LILRA2 | 0.603 | Upregulated | 6.76E-04 | 2.53E-02 |
| NADK | 0.603 | Upregulated | 4.82E-05 | 4.89E-03 |
| NPC2 | 0.605 | Upregulated | 1.97E-07 | 2.74E-04 |
| INSL3 | 0.605 | Upregulated | 4.35E-03 | 8.33E-02 |
| NUDT16 | 0.605 | Upregulated | 1.87E-05 | 2.94E-03 |
| XPO6 | 0.606 | Upregulated | 1.51E-05 | 2.64E-03 |
| APOL1 | 0.607 | Upregulated | 1.30E-08 | 4.37E-05 |
| ARHGAP26 | 0.608 | Upregulated | 1.50E-05 | 2.64E-03 |
| ST6GALNAC2 | 0.609 | Upregulated | 6.80E-04 | 2.54E-02 |
| SSFA2 | 0.609 | Upregulated | 4.52E-05 | 4.70E-03 |
| PIK3CB | 0.610 | Upregulated | 9.58E-04 | 3.22E-02 |
| FAM160B1 | 0.610 | Upregulated | 9.53E-05 | 7.45E-03 |
| SLAMF8 | 0.611 | Upregulated | 1.65E-04 | 1.04E-02 |
| DENND3 | 0.613 | Upregulated | 8.32E-05 | 6.99E-03 |
| CDA | 0.613 | Upregulated | 5.63E-03 | 9.60E-02 |
| CYP1B1 | 0.614 | Upregulated | 4.66E-02 | 3.16E-01 |
| GNG10 | 0.614 | Upregulated | 6.72E-04 | 2.52E-02 |
| CADM4 | 0.616 | Upregulated | 2.07E-05 | 3.07E-03 |
| FLJ22662 | 0.616 | Upregulated | 1.80E-03 | 4.84E-02 |
| FBXL5 | 0.616 | Upregulated | 1.91E-04 | 1.12E-02 |
| LHFPL2 | 0.617 | Upregulated | 2.71E-03 | 6.27E-02 |
| EIF2C4 | 0.618 | Upregulated | 2.12E-04 | 1.20E-02 |
| REPS2 | 0.619 | Upregulated | 7.29E-04 | 2.67E-02 |
| SLC43A2 | 0.621 | Upregulated | 4.94E-05 | 4.96E-03 |
| FNDC3B | 0.622 | Upregulated | 4.10E-05 | 4.43E-03 |
| CEBPB | 0.623 | Upregulated | 2.99E-05 | 3.70E-03 |
| AGPAT9 | 0.624 | Upregulated | 1.05E-03 | 3.44E-02 |
| FLJ10357 | 0.624 | Upregulated | 8.32E-05 | 6.99E-03 |
| RNF144B | 0.624 | Upregulated | 2.07E-05 | 3.07E-03 |
| SLC22A16 | 0.625 | Upregulated | 6.55E-04 | 2.48E-02 |
| DOK3 | 0.625 | Upregulated | 3.34E-04 | 1.58E-02 |
| TNFRSF10B | 0.625 | Upregulated | 1.49E-03 | 4.34E-02 |
| FBXL13 | 0.626 | Upregulated | 1.75E-04 | 1.07E-02 |
| CDC42EP2 | 0.626 | Upregulated | 8.62E-06 | 1.98E-03 |
| FES | 0.627 | Upregulated | 9.60E-05 | 7.46E-03 |
| LACTB | 0.628 | Upregulated | 7.72E-06 | 1.90E-03 |
| ZCCHC6 | 0.628 | Upregulated | 2.58E-05 | 3.50E-03 |
| CXCL10 | 0.629 | Upregulated | 2.57E-02 | 2.33E-01 |
| P2RY14 | 0.629 | Upregulated | 4.00E-06 | 1.41E-03 |
| NFKBIA | 0.630 | Upregulated | 3.63E-06 | 1.32E-03 |
| VNN3 | 0.630 | Upregulated | 1.38E-04 | 9.46E-03 |
| SLC16A3 | 0.631 | Upregulated | 4.69E-04 | 1.97E-02 |
| S1PR3 | 0.631 | Upregulated | 1.49E-04 | 9.95E-03 |
| HIST1H2BD | 0.632 | Upregulated | 5.88E-03 | 9.85E-02 |
| HK3 | 0.632 | Upregulated | 3.05E-03 | 6.77E-02 |

| | | | | |
|---|---|---|---|---|
| SPI1 | 0.633 | Upregulated | 2.25E-04 | 1.25E-02 |
| LRRC25 | 0.634 | Upregulated | 2.06E-04 | 1.18E-02 |
| HIST1H3D | 0.636 | Upregulated | 1.09E-03 | 3.52E-02 |
| SNX20 | 0.637 | Upregulated | 2.66E-07 | 3.56E-04 |
| LATS2 | 0.638 | Upregulated | 3.00E-05 | 3.70E-03 |
| VAMP5 | 0.639 | Upregulated | 2.86E-06 | 1.26E-03 |
| TRIM22 | 0.639 | Upregulated | 3.28E-05 | 3.82E-03 |
| BRI3 | 0.640 | Upregulated | 9.58E-05 | 7.46E-03 |
| RNF149 | 0.640 | Upregulated | 4.04E-05 | 4.39E-03 |
| GADD45B | 0.640 | Upregulated | 4.22E-06 | 1.46E-03 |
| CDK5RAP2 | 0.640 | Upregulated | 6.19E-04 | 2.38E-02 |
| SV2A | 0.641 | Upregulated | 3.57E-05 | 4.04E-03 |
| IFI30 | 0.642 | Upregulated | 6.81E-05 | 6.16E-03 |
| SP100 | 0.642 | Upregulated | 9.43E-05 | 7.42E-03 |
| IRF1 | 0.643 | Upregulated | 6.52E-08 | 1.13E-04 |
| LYN | 0.644 | Upregulated | 1.35E-06 | 8.11E-04 |
| MAML3 | 0.644 | Upregulated | 2.44E-05 | 3.36E-03 |
| ROPN1L | 0.644 | Upregulated | 2.82E-03 | 6.43E-02 |
| USP10 | 0.646 | Upregulated | 9.68E-04 | 3.25E-02 |
| SAMD9L | 0.646 | Upregulated | 1.13E-04 | 8.32E-03 |
| MSRB2 | 0.647 | Upregulated | 8.26E-05 | 6.99E-03 |
| KCNMB1 | 0.648 | Upregulated | 2.85E-05 | 3.61E-03 |
| AXUD1 | 0.648 | Upregulated | 1.52E-05 | 2.64E-03 |
| HCK | 0.650 | Upregulated | 8.69E-05 | 7.16E-03 |
| DRAM1 | 0.650 | Upregulated | 9.61E-05 | 7.46E-03 |
| TRIM25 | 0.652 | Upregulated | 2.85E-06 | 1.26E-03 |
| MANSC1 | 0.653 | Upregulated | 3.65E-03 | 7.41E-02 |
| EPSTI1 | 0.657 | Upregulated | 3.20E-04 | 1.55E-02 |
| PFKFB4 | 0.659 | Upregulated | 1.58E-04 | 1.02E-02 |
| FRAT2 | 0.661 | Upregulated | 1.43E-04 | 9.71E-03 |
| TLR6 | 0.661 | Upregulated | 5.57E-06 | 1.64E-03 |
| MMRN1 | 0.661 | Upregulated | 2.89E-03 | 6.54E-02 |
| PGS1 | 0.662 | Upregulated | 3.68E-04 | 1.69E-02 |
| SIGLEC5 | 0.662 | Upregulated | 1.56E-03 | 4.47E-02 |
| ETV7 | 0.665 | Upregulated | 5.38E-05 | 5.31E-03 |
| EPB41L3 | 0.667 | Upregulated | 4.32E-05 | 4.55E-03 |
| CRISPLD2 | 0.669 | Upregulated | 1.67E-03 | 4.62E-02 |
| TLE3 | 0.670 | Upregulated | 4.76E-05 | 4.85E-03 |
| HIST1H2BG | 0.671 | Upregulated | 5.11E-04 | 2.09E-02 |
| KCNE1 | 0.673 | Upregulated | 1.35E-03 | 4.04E-02 |
| BST1 | 0.673 | Upregulated | 1.97E-03 | 5.13E-02 |
| TREML4 | 0.675 | Upregulated | 3.20E-03 | 6.92E-02 |
| RNF19B | 0.677 | Upregulated | 6.07E-07 | 5.64E-04 |
| MMP25 | 0.678 | Upregulated | 1.21E-04 | 8.69E-03 |
| NCF4 | 0.678 | Upregulated | 2.56E-04 | 1.34E-02 |
| GPR160 | 0.679 | Upregulated | 5.19E-04 | 2.10E-02 |
| TAP1 | 0.679 | Upregulated | 1.55E-05 | 2.66E-03 |
| TNFAIP6 | 0.679 | Upregulated | 9.41E-07 | 6.79E-04 |
| HIST1H2BF | 0.679 | Upregulated | 1.17E-03 | 3.68E-02 |
| TREM1 | 0.680 | Upregulated | 4.30E-03 | 8.26E-02 |
| TYMP | 0.681 | Upregulated | 1.30E-05 | 2.48E-03 |

| | | | | |
|---|---|---|---|---|
| HRH2 | 0.682 | Upregulated | 2.61E-04 | 1.36E-02 |
| IL1R2 | 0.682 | Upregulated | 3.48E-05 | 3.99E-03 |
| FAM21A | 0.684 | Upregulated | 1.31E-02 | 1.61E-01 |
| ZNF467 | 0.685 | Upregulated | 4.05E-04 | 1.81E-02 |
| AGTRAP | 0.687 | Upregulated | 9.07E-05 | 7.26E-03 |
| SERPING1 | 0.688 | Upregulated | 5.24E-06 | 1.58E-03 |
| RAB24 | 0.689 | Upregulated | 4.48E-06 | 1.46E-03 |
| SVIL | 0.690 | Upregulated | 9.95E-05 | 7.62E-03 |
| SLC6A6 | 0.690 | Upregulated | 6.67E-07 | 5.64E-04 |
| IL13RA1 | 0.692 | Upregulated | 9.81E-05 | 7.58E-03 |
| HIST2H2BE | 0.694 | Upregulated | 1.03E-03 | 3.40E-02 |
| PLXDC2 | 0.695 | Upregulated | 2.44E-04 | 1.31E-02 |
| TSC22D3 | 0.695 | Upregulated | 3.70E-04 | 1.69E-02 |
| PLAUR | 0.698 | Upregulated | 2.30E-05 | 3.27E-03 |
| EMR2 | 0.699 | Upregulated | 7.46E-04 | 2.71E-02 |
| DSC2 | 0.699 | Upregulated | 4.87E-04 | 2.02E-02 |
| RALB | 0.699 | Upregulated | 4.43E-06 | 1.46E-03 |
| CD97 | 0.700 | Upregulated | 8.65E-08 | 1.39E-04 |
| FGL2 | 0.700 | Upregulated | 1.44E-06 | 8.42E-04 |
| NFIL3 | 0.701 | Upregulated | 2.82E-04 | 1.42E-02 |
| ALDH1A1 | 0.702 | Upregulated | 2.20E-04 | 1.23E-02 |
| VNN1 | 0.704 | Upregulated | 2.39E-02 | 2.24E-01 |
| TMEM88 | 0.706 | Upregulated | 4.29E-04 | 1.86E-02 |
| CLEC4D | 0.706 | Upregulated | 2.26E-03 | 5.61E-02 |
| CD163 | 0.707 | Upregulated | 2.05E-03 | 5.26E-02 |
| TNFRSF1A | 0.709 | Upregulated | 6.53E-07 | 5.64E-04 |
| DMXL2 | 0.709 | Upregulated | 1.00E-06 | 6.79E-04 |
| KIF1B | 0.710 | Upregulated | 3.56E-04 | 1.64E-02 |
| TNFSF10 | 0.710 | Upregulated | 6.70E-06 | 1.77E-03 |
| NCF1 | 0.714 | Upregulated | 8.69E-06 | 1.98E-03 |
| HCG27 | 0.715 | Upregulated | 2.26E-05 | 3.25E-03 |
| F2RL1 | 0.715 | Upregulated | 4.18E-04 | 1.84E-02 |
| RP2 | 0.715 | Upregulated | 6.23E-05 | 5.85E-03 |
| TCN2 | 0.716 | Upregulated | 6.88E-05 | 6.17E-03 |
| REM2 | 0.716 | Upregulated | 1.09E-04 | 8.15E-03 |
| MSL1 | 0.718 | Upregulated | 7.35E-06 | 1.88E-03 |
| SLC31A2 | 0.721 | Upregulated | 2.12E-06 | 1.07E-03 |
| CLEC4E | 0.723 | Upregulated | 2.23E-04 | 1.24E-02 |
| RTP4 | 0.724 | Upregulated | 9.27E-05 | 7.35E-03 |
| IL6R | 0.725 | Upregulated | 6.27E-05 | 5.86E-03 |
| HIST1H1T | 0.728 | Upregulated | 1.31E-04 | 9.19E-03 |
| B4GALT5 | 0.729 | Upregulated | 5.76E-05 | 5.57E-03 |
| NTNG2 | 0.730 | Upregulated | 1.64E-04 | 1.04E-02 |
| FZD2 | 0.730 | Upregulated | 7.82E-05 | 6.78E-03 |
| SORT1 | 0.731 | Upregulated | 1.59E-04 | 1.02E-02 |
| SOCS3 | 0.733 | Upregulated | 3.68E-05 | 4.07E-03 |
| SLC2A14 | 0.734 | Upregulated | 1.50E-03 | 4.37E-02 |
| IRAK3 | 0.737 | Upregulated | 5.09E-04 | 2.08E-02 |
| CD300A | 0.737 | Upregulated | 3.58E-07 | 4.24E-04 |
| HPSE | 0.740 | Upregulated | 3.27E-04 | 1.56E-02 |
| BCL3 | 0.740 | Upregulated | 6.66E-06 | 1.77E-03 |

| | | | | |
|---|---|---|---|---|
| IDO1 | 0.741 | Upregulated | 2.27E-03 | 5.62E-02 |
| TRPM6 | 0.743 | Upregulated | 5.40E-04 | 2.17E-02 |
| DHRS9 | 0.743 | Upregulated | 7.13E-05 | 6.31E-03 |
| SLC22A15 | 0.746 | Upregulated | 1.72E-04 | 1.06E-02 |
| PANX2 | 0.746 | Upregulated | 3.47E-03 | 7.23E-02 |
| SEPX1 | 0.746 | Upregulated | 2.98E-05 | 3.70E-03 |
| GNAQ | 0.746 | Upregulated | 1.53E-04 | 1.01E-02 |
| MNDA | 0.746 | Upregulated | 1.82E-05 | 2.89E-03 |
| SCO2 | 0.747 | Upregulated | 1.44E-05 | 2.58E-03 |
| TIFA | 0.749 | Upregulated | 7.70E-06 | 1.90E-03 |
| FRAT1 | 0.750 | Upregulated | 2.43E-05 | 3.36E-03 |
| WARS | 0.750 | Upregulated | 1.84E-06 | 9.54E-04 |
| DDX60L | 0.750 | Upregulated | 1.36E-04 | 9.43E-03 |
| ECGF1 | 0.753 | Upregulated | 8.90E-06 | 1.98E-03 |
| HIST1H2BE | 0.753 | Upregulated | 2.62E-04 | 1.36E-02 |
| GCH1 | 0.754 | Upregulated | 1.09E-06 | 7.16E-04 |
| TPST1 | 0.754 | Upregulated | 3.55E-03 | 7.33E-02 |
| ZDHHC18 | 0.754 | Upregulated | 1.46E-06 | 8.42E-04 |
| TIMM10 | 0.758 | Upregulated | 1.08E-04 | 8.10E-03 |
| JUNB | 0.758 | Upregulated | 3.40E-06 | 1.32E-03 |
| SIRPA | 0.759 | Upregulated | 1.57E-04 | 1.02E-02 |
| STK3 | 0.759 | Upregulated | 6.69E-06 | 1.77E-03 |
| MYBPC3 | 0.760 | Upregulated | 9.54E-05 | 7.45E-03 |
| HIST2H2AA3 | 0.760 | Upregulated | 1.17E-03 | 3.68E-02 |
| SLC6A12 | 0.760 | Upregulated | 1.39E-05 | 2.56E-03 |
| RSAD2 | 0.763 | Upregulated | 2.57E-02 | 2.33E-01 |
| IL1RAP | 0.763 | Upregulated | 1.17E-04 | 8.54E-03 |
| RBM47 | 0.764 | Upregulated | 2.80E-05 | 3.61E-03 |
| IRF7 | 0.766 | Upregulated | 3.30E-03 | 7.01E-02 |
| C1RL | 0.768 | Upregulated | 2.56E-05 | 3.48E-03 |
| C2 | 0.768 | Upregulated | 3.55E-06 | 1.32E-03 |
| TGFA | 0.768 | Upregulated | 7.82E-04 | 2.81E-02 |
| PLXNC1 | 0.769 | Upregulated | 1.04E-04 | 7.91E-03 |
| GPR141 | 0.769 | Upregulated | 2.79E-04 | 1.42E-02 |
| ECE1 | 0.769 | Upregulated | 5.13E-06 | 1.58E-03 |
| IGSF6 | 0.770 | Upregulated | 3.33E-05 | 3.85E-03 |
| PILRA | 0.772 | Upregulated | 1.35E-05 | 2.50E-03 |
| RAB20 | 0.772 | Upregulated | 3.05E-06 | 1.28E-03 |
| APOB48R | 0.776 | Upregulated | 5.62E-05 | 5.46E-03 |
| HIST1H2BC | 0.777 | Upregulated | 1.13E-03 | 3.61E-02 |
| MXD1 | 0.779 | Upregulated | 1.33E-05 | 2.48E-03 |
| HIST2H2AC | 0.779 | Upregulated | 6.21E-04 | 2.38E-02 |
| ASGR2 | 0.780 | Upregulated | 7.78E-05 | 6.77E-03 |
| CECR6 | 0.781 | Upregulated | 1.16E-04 | 8.48E-03 |
| FCAR | 0.786 | Upregulated | 1.59E-04 | 1.02E-02 |
| NLRC4 | 0.786 | Upregulated | 1.14E-04 | 8.39E-03 |
| MEGF9 | 0.787 | Upregulated | 3.06E-05 | 3.72E-03 |
| SBNO2 | 0.788 | Upregulated | 3.56E-07 | 4.24E-04 |
| SLC26A8 | 0.791 | Upregulated | 3.13E-05 | 3.76E-03 |
| LMNB1 | 0.792 | Upregulated | 1.04E-05 | 2.18E-03 |
| SOCS1 | 0.794 | Upregulated | 2.63E-05 | 3.53E-03 |

| | | | | |
|---|---|---|---|---|
| ETS2 | 0.796 | Upregulated | 1.02E-05 | 2.15E-03 |
| PDCD1LG2 | 0.798 | Upregulated | 3.37E-06 | 1.32E-03 |
| BASP1 | 0.799 | Upregulated | 5.87E-05 | 5.64E-03 |
| LIN7A | 0.801 | Upregulated | 4.12E-04 | 1.83E-02 |
| HIST2H2AA4 | 0.805 | Upregulated | 2.90E-04 | 1.44E-02 |
| ALPK1 | 0.806 | Upregulated | 4.92E-06 | 1.55E-03 |
| ABCA1 | 0.808 | Upregulated | 9.86E-05 | 7.60E-03 |
| CSTA | 0.809 | Upregulated | 8.78E-06 | 1.98E-03 |
| TIMP2 | 0.814 | Upregulated | 1.44E-05 | 2.58E-03 |
| MAPK14 | 0.814 | Upregulated | 5.59E-05 | 5.45E-03 |
| FCER1G | 0.814 | Upregulated | 6.57E-06 | 1.77E-03 |
| ERV3 | 0.814 | Upregulated | 3.61E-06 | 1.32E-03 |
| BEST1 | 0.819 | Upregulated | 1.65E-05 | 2.75E-03 |
| HIST1H4H | 0.823 | Upregulated | 3.40E-04 | 1.60E-02 |
| DUSP3 | 0.824 | Upregulated | 7.74E-07 | 6.39E-04 |
| LRRK2 | 0.825 | Upregulated | 1.84E-05 | 2.91E-03 |
| BRSK1 | 0.831 | Upregulated | 1.05E-05 | 2.19E-03 |
| ADCY3 | 0.835 | Upregulated | 5.58E-05 | 5.45E-03 |
| LTBR | 0.836 | Upregulated | 1.87E-06 | 9.54E-04 |
| CEBPD | 0.837 | Upregulated | 6.18E-06 | 1.73E-03 |
| GPR84 | 0.838 | Upregulated | 1.99E-03 | 5.17E-02 |
| TNFAIP2 | 0.839 | Upregulated | 1.36E-06 | 8.11E-04 |
| MBOAT7 | 0.841 | Upregulated | 3.07E-05 | 3.73E-03 |
| VNN2 | 0.842 | Upregulated | 8.58E-05 | 7.13E-03 |
| STX3 | 0.842 | Upregulated | 2.31E-05 | 3.27E-03 |
| CLEC5A | 0.848 | Upregulated | 2.36E-03 | 5.80E-02 |
| ACSL1 | 0.848 | Upregulated | 1.34E-03 | 4.03E-02 |
| APOL6 | 0.854 | Upregulated | 1.02E-08 | 4.05E-05 |
| SERPINA1 | 0.855 | Upregulated | 5.51E-06 | 1.64E-03 |
| PSTPIP2 | 0.860 | Upregulated | 4.74E-05 | 4.85E-03 |
| FBXO6 | 0.862 | Upregulated | 4.14E-06 | 1.45E-03 |
| STX11 | 0.865 | Upregulated | 1.01E-06 | 6.79E-04 |
| ST3GAL4 | 0.866 | Upregulated | 2.73E-06 | 1.26E-03 |
| DUSP1 | 0.869 | Upregulated | 7.72E-06 | 1.90E-03 |
| FCGR3B | 0.873 | Upregulated | 1.23E-04 | 8.76E-03 |
| LAP3 | 0.874 | Upregulated | 1.54E-06 | 8.46E-04 |
| GRAMD1B | 0.876 | Upregulated | 8.83E-08 | 1.39E-04 |
| ARL11 | 0.876 | Upregulated | 1.33E-06 | 8.11E-04 |
| FLJ20273 | 0.877 | Upregulated | 1.73E-04 | 1.06E-02 |
| CSF2RB | 0.878 | Upregulated | 4.86E-07 | 5.11E-04 |
| CR1 | 0.880 | Upregulated | 3.62E-04 | 1.66E-02 |
| SOD2 | 0.881 | Upregulated | 1.23E-05 | 2.46E-03 |
| PSG9 | 0.883 | Upregulated | 1.53E-05 | 2.65E-03 |
| BCL6 | 0.884 | Upregulated | 1.90E-05 | 2.95E-03 |
| EGLN1 | 0.886 | Upregulated | 1.74E-05 | 2.82E-03 |
| LILRA5 | 0.887 | Upregulated | 5.90E-04 | 2.29E-02 |
| FAM129A | 0.889 | Upregulated | 8.15E-06 | 1.97E-03 |
| IL8RB | 0.893 | Upregulated | 5.43E-04 | 2.18E-02 |
| IL27 | 0.893 | Upregulated | 1.01E-06 | 6.79E-04 |
| RFX2 | 0.893 | Upregulated | 2.24E-05 | 3.24E-03 |
| MCTP2 | 0.893 | Upregulated | 1.97E-05 | 3.01E-03 |

| | | | | |
|---|---|---|---|---|
| IL18RAP | 0.894 | Upregulated | 3.49E-05 | 3.99E-03 |
| METTL7B | 0.895 | Upregulated | 1.60E-03 | 4.51E-02 |
| NOD2 | 0.896 | Upregulated | 3.28E-06 | 1.32E-03 |
| RNF24 | 0.907 | Upregulated | 4.49E-06 | 1.46E-03 |
| DYSF | 0.911 | Upregulated | 1.98E-04 | 1.15E-02 |
| TNFSF13B | 0.914 | Upregulated | 4.27E-06 | 1.46E-03 |
| FCGR2A | 0.917 | Upregulated | 6.91E-06 | 1.81E-03 |
| TLR8 | 0.918 | Upregulated | 3.31E-05 | 3.85E-03 |
| TLR5 | 0.927 | Upregulated | 9.11E-05 | 7.26E-03 |
| TMEM140 | 0.927 | Upregulated | 3.15E-06 | 1.29E-03 |
| GBP6 | 0.929 | Upregulated | 1.42E-08 | 4.37E-05 |
| IFIT3 | 0.930 | Upregulated | 7.38E-04 | 2.69E-02 |
| CCR1 | 0.931 | Upregulated | 1.13E-05 | 2.29E-03 |
| GBP5 | 0.934 | Upregulated | 5.52E-06 | 1.64E-03 |
| TECPR2 | 0.934 | Upregulated | 1.30E-05 | 2.48E-03 |
| TMEM119 | 0.935 | Upregulated | 3.11E-03 | 6.81E-02 |
| OSM | 0.937 | Upregulated | 7.17E-05 | 6.33E-03 |
| IFIT2 | 0.939 | Upregulated | 4.45E-05 | 4.65E-03 |
| DOCK5 | 0.942 | Upregulated | 1.40E-07 | 2.02E-04 |
| HSPA6 | 0.943 | Upregulated | 6.62E-07 | 5.64E-04 |
| CSF3R | 0.945 | Upregulated | 4.46E-06 | 1.46E-03 |
| AQP9 | 0.947 | Upregulated | 8.52E-06 | 1.98E-03 |
| FOS | 0.948 | Upregulated | 8.17E-06 | 1.97E-03 |
| ANXA3 | 0.956 | Upregulated | 5.71E-03 | 9.69E-02 |
| ZNF438 | 0.962 | Upregulated | 8.91E-06 | 1.98E-03 |
| TLR4 | 0.969 | Upregulated | 1.25E-05 | 2.48E-03 |
| PROK2 | 0.971 | Upregulated | 3.74E-04 | 1.70E-02 |
| ZMYND15 | 0.973 | Upregulated | 8.74E-07 | 6.74E-04 |
| KCNJ2 | 0.973 | Upregulated | 3.33E-06 | 1.32E-03 |
| IL18R1 | 0.983 | Upregulated | 6.37E-04 | 2.42E-02 |
| GBP1 | 0.989 | Upregulated | 1.21E-06 | 7.62E-04 |
| P2RY13 | 0.990 | Upregulated | 1.06E-05 | 2.21E-03 |
| FPR1 | 0.993 | Upregulated | 1.95E-05 | 2.99E-03 |
| IFITM3 | 0.997 | Upregulated | 7.26E-06 | 1.87E-03 |
| CXCR1 | 1.004 | Upregulated | 5.31E-05 | 5.25E-03 |
| FER1L3 | 1.011 | Upregulated | 3.44E-06 | 1.32E-03 |
| STEAP4 | 1.021 | Upregulated | 4.93E-05 | 4.96E-03 |
| GPR97 | 1.027 | Upregulated | 1.59E-05 | 2.71E-03 |
| KREMEN1 | 1.037 | Upregulated | 6.22E-05 | 5.85E-03 |
| LRG1 | 1.045 | Upregulated | 1.55E-04 | 1.01E-02 |
| MYOF | 1.048 | Upregulated | 4.51E-06 | 1.46E-03 |
| ASPRV1 | 1.053 | Upregulated | 5.28E-08 | 9.65E-05 |
| LIMK2 | 1.057 | Upregulated | 5.10E-08 | 9.65E-05 |
| C1QC | 1.067 | Upregulated | 3.29E-04 | 1.56E-02 |
| SMARCD3 | 1.068 | Upregulated | 1.29E-05 | 2.48E-03 |
| PLSCR1 | 1.080 | Upregulated | 6.94E-06 | 1.81E-03 |
| KCNJ15 | 1.083 | Upregulated | 8.78E-06 | 1.98E-03 |
| CEACAM3 | 1.104 | Upregulated | 8.40E-07 | 6.63E-04 |
| CEACAM1 | 1.108 | Upregulated | 2.75E-05 | 3.61E-03 |
| LPCAT2 | 1.113 | Upregulated | 5.96E-06 | 1.69E-03 |
| ADM | 1.127 | Upregulated | 1.19E-06 | 7.62E-04 |

| | | | | |
|---|---|---|---|---|
| C1QB | 1.134 | Upregulated | 4.84E-04 | 2.02E-02 |
| GPR109B | 1.134 | Upregulated | 2.10E-08 | 4.86E-05 |
| ALPL | 1.137 | Upregulated | 9.28E-05 | 7.35E-03 |
| FFAR2 | 1.139 | Upregulated | 2.66E-06 | 1.26E-03 |
| SIPA1L2 | 1.142 | Upregulated | 1.73E-05 | 2.82E-03 |
| NAMPT | 1.153 | Upregulated | 6.29E-06 | 1.74E-03 |
| IL1RN | 1.156 | Upregulated | 2.77E-05 | 3.61E-03 |
| CARD17 | 1.165 | Upregulated | 9.53E-07 | 6.79E-04 |
| FPR2 | 1.170 | Upregulated | 6.22E-07 | 5.64E-04 |
| TLR2 | 1.194 | Upregulated | 6.17E-07 | 5.64E-04 |
| CACNA1E | 1.247 | Upregulated | 1.50E-06 | 8.42E-04 |
| IL1B | 1.283 | Upregulated | 7.46E-09 | 3.70E-05 |
| GPR109A | 1.337 | Upregulated | 1.05E-08 | 4.05E-05 |
| FOLR3 | 1.349 | Upregulated | 2.11E-04 | 1.19E-02 |
| GK | 1.352 | Upregulated | 8.69E-10 | 1.01E-05 |
| CD274 | 1.449 | Upregulated | 5.06E-09 | 2.93E-05 |
| BATF2 | 1.541 | Upregulated | 3.31E-09 | 2.29E-05 |
| FCGR1C | 1.557 | Upregulated | 1.79E-08 | 4.44E-05 |
| FCGR1B | 1.580 | Upregulated | 1.64E-08 | 4.37E-05 |
| ANKRD22 | 1.718 | Upregulated | 1.57E-08 | 4.37E-05 |
| CASP5 | 1.820 | Upregulated | 2.65E-08 | 5.74E-05 |
| FCGR1A | 1.830 | Upregulated | 4.78E-10 | 8.29E-06 |

**Supplementary Table S3e. Differentially expressed genes_South Africa_Female**

| Gene | log FoldChange | Direction of expression | P.Value | adj.P.Val |
|---|---|---|---|---|
| CD177 | -0.682 | Downregulated | 3.06E-02 | 1.93E-01 |
| CDC20 | -0.663 | Downregulated | 7.36E-04 | 2.33E-02 |
| TNFRSF13B | -0.654 | Downregulated | 9.35E-05 | 7.49E-03 |
| CCNA1 | -0.609 | Downregulated | 3.25E-02 | 1.99E-01 |
| ZNF573 | -0.603 | Downregulated | 2.27E-05 | 3.21E-03 |
| CDKN1C | -0.596 | Downregulated | 6.89E-04 | 2.25E-02 |
| AURKB | -0.588 | Downregulated | 1.51E-04 | 9.67E-03 |
| CCNB2 | -0.587 | Downregulated | 1.25E-03 | 3.06E-02 |
| MGC29506 | -0.551 | Downregulated | 3.25E-03 | 5.26E-02 |
| GLDC | -0.545 | Downregulated | 1.00E-02 | 1.03E-01 |
| TXNDC5 | -0.530 | Downregulated | 9.08E-03 | 9.70E-02 |
| LAG3 | -0.527 | Downregulated | 5.64E-03 | 7.40E-02 |
| GALR2 | -0.521 | Downregulated | 1.35E-06 | 6.96E-04 |
| EIF2B5 | -0.517 | Downregulated | 2.34E-06 | 9.22E-04 |
| ORM1 | -0.514 | Downregulated | 3.89E-02 | 2.21E-01 |
| CLDND2 | -0.502 | Downregulated | 2.28E-04 | 1.23E-02 |
| SDF2L1 | -0.496 | Downregulated | 1.25E-05 | 2.31E-03 |
| PACAP | -0.494 | Downregulated | 8.92E-05 | 7.34E-03 |
| MSC | -0.494 | Downregulated | 1.94E-03 | 3.91E-02 |
| BUB1 | -0.490 | Downregulated | 9.76E-03 | 1.01E-01 |
| KIAA0101 | -0.489 | Downregulated | 9.42E-03 | 9.91E-02 |
| CDC45L | -0.486 | Downregulated | 6.01E-03 | 7.69E-02 |
| CHST12 | -0.485 | Downregulated | 4.46E-06 | 1.27E-03 |
| PARK7 | -0.474 | Downregulated | 2.76E-06 | 9.87E-04 |
| KIFC1 | -0.471 | Downregulated | 5.98E-04 | 2.08E-02 |
| MCM4 | -0.470 | Downregulated | 3.18E-04 | 1.49E-02 |
| STOML2 | -0.470 | Downregulated | 1.61E-07 | 3.10E-04 |
| VIL2 | -0.465 | Downregulated | 3.12E-07 | 3.87E-04 |
| IL28RA | -0.463 | Downregulated | 1.96E-05 | 2.96E-03 |
| CDT1 | -0.452 | Downregulated | 5.07E-03 | 6.94E-02 |
| NLRP2 | -0.447 | Downregulated | 3.57E-04 | 1.58E-02 |
| HESX1 | -0.443 | Downregulated | 4.29E-02 | 2.33E-01 |
| GPRC5D | -0.442 | Downregulated | 2.59E-03 | 4.63E-02 |
| BHLHB3 | -0.441 | Downregulated | 2.80E-05 | 3.64E-03 |
| MCM2 | -0.441 | Downregulated | 1.39E-03 | 3.23E-02 |
| CD38 | -0.441 | Downregulated | 4.02E-03 | 6.03E-02 |
| TK1 | -0.438 | Downregulated | 1.59E-03 | 3.49E-02 |
| TOP2A | -0.436 | Downregulated | 1.19E-02 | 1.13E-01 |
| CXCR3 | -0.435 | Downregulated | 8.05E-04 | 2.45E-02 |
| HERC2 | -0.426 | Downregulated | 4.97E-05 | 5.13E-03 |
| PMM1 | -0.426 | Downregulated | 6.15E-04 | 2.12E-02 |
| NDUFAF1 | -0.421 | Downregulated | 5.21E-06 | 1.38E-03 |
| ITM2C | -0.421 | Downregulated | 5.79E-03 | 7.52E-02 |

| | | | | |
|---|---|---|---|---|
| CHEK1 | -0.417 | Downregulated | 3.41E-04 | 1.54E-02 |
| GSG2 | -0.417 | Downregulated | 2.41E-04 | 1.28E-02 |
| UIMC1 | -0.417 | Downregulated | 1.70E-04 | 1.04E-02 |
| EBI3 | -0.415 | Downregulated | 3.15E-03 | 5.18E-02 |
| TMEM110 | -0.414 | Downregulated | 8.23E-07 | 5.84E-04 |
| CDCA5 | -0.414 | Downregulated | 7.60E-03 | 8.81E-02 |
| TACO1 | -0.411 | Downregulated | 5.35E-04 | 1.95E-02 |
| MIIP | -0.409 | Downregulated | 5.32E-04 | 1.94E-02 |
| CENPM | -0.409 | Downregulated | 4.76E-04 | 1.85E-02 |
| CENPA | -0.408 | Downregulated | 1.54E-03 | 3.43E-02 |
| BIRC5 | -0.408 | Downregulated | 1.29E-04 | 8.92E-03 |
| EPPB9 | -0.405 | Downregulated | 3.79E-04 | 1.63E-02 |
| JSRP1 | -0.404 | Downregulated | 2.20E-03 | 4.18E-02 |
| NCAPG | -0.403 | Downregulated | 1.96E-02 | 1.49E-01 |
| HJURP | -0.401 | Downregulated | 3.67E-04 | 1.60E-02 |
| KIF4A | -0.400 | Downregulated | 7.88E-04 | 2.44E-02 |
| TYMS | -0.399 | Downregulated | 6.42E-03 | 7.95E-02 |
| RFC3 | -0.397 | Downregulated | 1.45E-04 | 9.44E-03 |
| CCDC134 | -0.396 | Downregulated | 6.72E-07 | 5.80E-04 |
| AURKA | -0.396 | Downregulated | 2.61E-03 | 4.64E-02 |
| MKI67 | -0.395 | Downregulated | 3.19E-04 | 1.49E-02 |
| CCNA2 | -0.393 | Downregulated | 6.28E-03 | 7.88E-02 |
| NT5DC2 | -0.392 | Downregulated | 6.47E-03 | 7.98E-02 |
| CDCA2 | -0.391 | Downregulated | 6.94E-04 | 2.26E-02 |
| TRIP13 | -0.390 | Downregulated | 3.39E-03 | 5.41E-02 |
| CKAP2L | -0.389 | Downregulated | 5.96E-03 | 7.67E-02 |
| UFSP2 | -0.388 | Downregulated | 2.09E-06 | 8.64E-04 |
| MCM10 | -0.387 | Downregulated | 1.93E-03 | 3.90E-02 |
| PBK | -0.386 | Downregulated | 7.61E-03 | 8.81E-02 |
| ERI1 | -0.384 | Downregulated | 4.14E-04 | 1.72E-02 |
| THOC6 | -0.384 | Downregulated | 9.74E-07 | 5.88E-04 |
| CDCA3 | -0.383 | Downregulated | 2.16E-03 | 4.14E-02 |
| BCAR3 | -0.383 | Downregulated | 3.42E-03 | 5.44E-02 |
| TPX2 | -0.380 | Downregulated | 1.88E-03 | 3.86E-02 |
| NUBP2 | -0.379 | Downregulated | 8.34E-06 | 1.75E-03 |
| FAM83D | -0.377 | Downregulated | 4.27E-04 | 1.76E-02 |
| TBC1D7 | -0.377 | Downregulated | 1.28E-07 | 2.97E-04 |
| TUBB | -0.375 | Downregulated | 4.54E-06 | 1.27E-03 |
| EBF1 | -0.373 | Downregulated | 1.76E-02 | 1.40E-01 |
| PTDSS1 | -0.373 | Downregulated | 3.52E-06 | 1.09E-03 |
| QPRT | -0.372 | Downregulated | 1.25E-04 | 8.72E-03 |
| CD79A | -0.371 | Downregulated | 4.57E-03 | 6.54E-02 |
| CEP55 | -0.371 | Downregulated | 6.03E-03 | 7.70E-02 |
| PRC1 | -0.370 | Downregulated | 2.32E-03 | 4.33E-02 |
| EVL | -0.366 | Downregulated | 2.14E-04 | 1.18E-02 |
| ZNF263 | -0.366 | Downregulated | 5.13E-06 | 1.38E-03 |
| POU2AF1 | -0.366 | Downregulated | 6.65E-03 | 8.11E-02 |

| | | | | |
|---|---|---|---|---|
| PHGDH | -0.365 | Downregulated | 5.36E-03 | 7.19E-02 |
| CDC25C | -0.364 | Downregulated | 3.49E-04 | 1.56E-02 |
| EBP | -0.364 | Downregulated | 7.82E-06 | 1.69E-03 |
| MAP4K1 | -0.364 | Downregulated | 1.95E-04 | 1.11E-02 |
| RPS6KA2 | -0.362 | Downregulated | 2.97E-03 | 5.02E-02 |
| ALDH18A1 | -0.362 | Downregulated | 1.48E-04 | 9.61E-03 |
| ABCB9 | -0.361 | Downregulated | 4.05E-02 | 2.26E-01 |
| CCNF | -0.359 | Downregulated | 1.96E-03 | 3.92E-02 |
| HDAC1 | -0.358 | Downregulated | 1.18E-06 | 6.47E-04 |
| TUBB3 | -0.358 | Downregulated | 1.54E-04 | 9.77E-03 |
| DLGAP5 | -0.357 | Downregulated | 2.02E-02 | 1.51E-01 |
| HSPC111 | -0.357 | Downregulated | 2.76E-04 | 1.38E-02 |
| MRPL2 | -0.356 | Downregulated | 8.21E-07 | 5.84E-04 |
| CPNE5 | -0.355 | Downregulated | 1.27E-03 | 3.10E-02 |
| CELSR3 | -0.353 | Downregulated | 4.22E-03 | 6.24E-02 |
| CHAD | -0.353 | Downregulated | 2.09E-05 | 3.07E-03 |
| CHCHD6 | -0.353 | Downregulated | 1.95E-04 | 1.11E-02 |
| PTTG1 | -0.352 | Downregulated | 1.23E-03 | 3.04E-02 |
| MESP1 | -0.350 | Downregulated | 2.62E-04 | 1.35E-02 |
| EWSR1 | -0.350 | Downregulated | 9.00E-07 | 5.88E-04 |
| CD19 | -0.349 | Downregulated | 8.22E-03 | 9.23E-02 |
| HERC6 | -0.349 | Downregulated | 4.79E-02 | 2.47E-01 |
| APOD | -0.348 | Downregulated | 5.99E-04 | 2.08E-02 |
| ADAM23 | -0.348 | Downregulated | 2.94E-03 | 5.00E-02 |
| ZBED2 | -0.348 | Downregulated | 1.27E-03 | 3.09E-02 |
| MELK | -0.347 | Downregulated | 5.25E-03 | 7.08E-02 |
| DENND5B | -0.346 | Downregulated | 6.93E-03 | 8.34E-02 |
| MIB2 | -0.344 | Downregulated | 4.75E-06 | 1.31E-03 |
| WDR34 | -0.344 | Downregulated | 1.91E-04 | 1.11E-02 |
| BIK | -0.344 | Downregulated | 4.34E-03 | 6.31E-02 |
| HMMR | -0.344 | Downregulated | 1.90E-02 | 1.46E-01 |
| CD320 | -0.343 | Downregulated | 1.10E-03 | 2.87E-02 |
| HARS | -0.343 | Downregulated | 1.34E-05 | 2.34E-03 |
| LIMS2 | -0.343 | Downregulated | 4.42E-03 | 6.38E-02 |
| TECR | -0.343 | Downregulated | 4.49E-05 | 4.90E-03 |
| POLR2I | -0.341 | Downregulated | 6.70E-07 | 5.80E-04 |
| RRM2 | -0.341 | Downregulated | 2.09E-02 | 1.55E-01 |
| ZBTB46 | -0.340 | Downregulated | 3.83E-03 | 5.85E-02 |
| KIF20A | -0.340 | Downregulated | 4.23E-03 | 6.24E-02 |
| DPAGT1 | -0.338 | Downregulated | 3.68E-07 | 3.87E-04 |
| NTHL1 | -0.338 | Downregulated | 1.54E-04 | 9.78E-03 |
| CHAF1B | -0.337 | Downregulated | 1.91E-03 | 3.88E-02 |
| FAM195B | -0.337 | Downregulated | 1.51E-07 | 3.10E-04 |
| OSBPL10 | -0.336 | Downregulated | 1.53E-02 | 1.30E-01 |
| ADA | -0.335 | Downregulated | 1.02E-03 | 2.74E-02 |
| UBE2J1 | -0.335 | Downregulated | 2.89E-05 | 3.69E-03 |
| ZNF593 | -0.335 | Downregulated | 9.01E-05 | 7.38E-03 |

| | | | | |
|---|---|---|---|---|
| PSMB5 | -0.334 | Downregulated | 1.03E-07 | 2.55E-04 |
| ZNF296 | -0.333 | Downregulated | 5.25E-05 | 5.22E-03 |
| BLVRA | -0.333 | Downregulated | 4.77E-04 | 1.85E-02 |
| SIL1 | -0.332 | Downregulated | 1.95E-05 | 2.96E-03 |
| WFS1 | -0.331 | Downregulated | 1.85E-03 | 3.82E-02 |
| KIF3C | -0.331 | Downregulated | 2.41E-04 | 1.28E-02 |
| STXBP2 | -0.331 | Downregulated | 1.74E-02 | 1.39E-01 |
| TNFRSF13C | -0.330 | Downregulated | 6.15E-03 | 7.77E-02 |
| GRWD1 | -0.330 | Downregulated | 1.90E-06 | 8.14E-04 |
| MCM7 | -0.329 | Downregulated | 3.65E-03 | 5.69E-02 |
| CCDC24 | -0.329 | Downregulated | 1.45E-04 | 9.43E-03 |
| PACSIN1 | -0.329 | Downregulated | 1.87E-02 | 1.45E-01 |
| HIST1H3C | -0.328 | Downregulated | 3.25E-04 | 1.51E-02 |
| TEX264 | -0.328 | Downregulated | 4.13E-07 | 4.13E-04 |
| SCG5 | -0.327 | Downregulated | 4.23E-06 | 1.23E-03 |
| KIAA0125 | -0.327 | Downregulated | 5.68E-03 | 7.43E-02 |
| GNB1L | -0.327 | Downregulated | 1.61E-08 | 7.96E-05 |
| MOXD1 | -0.325 | Downregulated | 6.23E-04 | 2.13E-02 |
| SIGLEC1 | -0.325 | Downregulated | 4.97E-02 | 2.52E-01 |
| ZBTB32 | -0.325 | Downregulated | 5.28E-04 | 1.94E-02 |
| BCAT1 | -0.325 | Downregulated | 8.00E-04 | 2.45E-02 |
| HOXB7 | -0.324 | Downregulated | 1.05E-03 | 2.79E-02 |
| STT3A | -0.324 | Downregulated | 2.00E-04 | 1.13E-02 |
| GPR55 | -0.324 | Downregulated | 1.75E-04 | 1.05E-02 |
| GNG3 | -0.323 | Downregulated | 2.77E-04 | 1.38E-02 |
| MYL6B | -0.323 | Downregulated | 4.37E-04 | 1.78E-02 |
| NDUFB7 | -0.323 | Downregulated | 3.34E-04 | 1.53E-02 |
| ATRIP | -0.322 | Downregulated | 1.80E-06 | 7.94E-04 |
| SLC25A4 | -0.322 | Downregulated | 3.93E-03 | 5.95E-02 |
| HIST1H4K | -0.321 | Downregulated | 2.96E-03 | 5.02E-02 |
| TRAM2 | -0.320 | Downregulated | 1.07E-02 | 1.07E-01 |
| DNAJC3 | -0.320 | Downregulated | 1.89E-03 | 3.87E-02 |
| CKB | -0.319 | Downregulated | 1.76E-03 | 3.70E-02 |
| GINS2 | -0.319 | Downregulated | 1.89E-02 | 1.46E-01 |
| MED8 | -0.319 | Downregulated | 5.06E-08 | 1.86E-04 |
| FXYD6 | -0.318 | Downregulated | 5.37E-03 | 7.19E-02 |
| UCK2 | -0.318 | Downregulated | 4.94E-04 | 1.88E-02 |
| FKBP11 | -0.318 | Downregulated | 9.85E-03 | 1.02E-01 |
| CRIP2 | -0.317 | Downregulated | 2.71E-02 | 1.79E-01 |
| SEL1L3 | -0.317 | Downregulated | 9.01E-04 | 2.59E-02 |
| RAPGEF5 | -0.317 | Downregulated | 1.93E-03 | 3.90E-02 |
| TIMELESS | -0.316 | Downregulated | 4.66E-04 | 1.83E-02 |
| PARM1 | -0.316 | Downregulated | 9.00E-03 | 9.66E-02 |
| PDCD1 | -0.316 | Downregulated | 2.39E-02 | 1.67E-01 |
| GINS4 | -0.316 | Downregulated | 9.16E-05 | 7.43E-03 |
| ANKRD54 | -0.315 | Downregulated | 1.72E-05 | 2.78E-03 |
| WDR70 | -0.315 | Downregulated | 2.98E-05 | 3.74E-03 |

| | | | | |
|---|---|---|---|---|
| WDR51A | -0.314 | Downregulated | 1.14E-03 | 2.92E-02 |
| BYSL | -0.314 | Downregulated | 4.53E-04 | 1.80E-02 |
| MCOLN2 | -0.314 | Downregulated | 2.16E-02 | 1.57E-01 |
| TEX10 | -0.314 | Downregulated | 1.53E-05 | 2.60E-03 |
| CCNE1 | -0.313 | Downregulated | 5.79E-04 | 2.05E-02 |
| ANKRD55 | -0.313 | Downregulated | 3.47E-02 | 2.06E-01 |
| IGFBP2 | -0.312 | Downregulated | 9.53E-03 | 9.98E-02 |
| SSNA1 | -0.312 | Downregulated | 4.18E-06 | 1.23E-03 |
| EEF1D | -0.312 | Downregulated | 9.53E-04 | 2.65E-02 |
| SPIB | -0.311 | Downregulated | 2.88E-02 | 1.86E-01 |
| FOXRED1 | -0.310 | Downregulated | 1.58E-04 | 9.90E-03 |
| TIMP1 | -0.310 | Downregulated | 1.40E-02 | 1.24E-01 |
| PDCD2L | -0.310 | Downregulated | 1.01E-03 | 2.73E-02 |
| ARMET | -0.309 | Downregulated | 4.46E-03 | 6.42E-02 |
| FLJ35801 | -0.309 | Downregulated | 5.22E-04 | 1.93E-02 |
| REXO4 | -0.309 | Downregulated | 4.15E-05 | 4.82E-03 |
| SPSB1 | -0.309 | Downregulated | 1.99E-05 | 2.97E-03 |
| TTC21A | -0.308 | Downregulated | 8.80E-03 | 9.54E-02 |
| RRP7A | -0.308 | Downregulated | 7.38E-04 | 2.34E-02 |
| SCAMP5 | -0.308 | Downregulated | 9.72E-04 | 2.68E-02 |
| LAS1L | -0.307 | Downregulated | 4.37E-05 | 4.88E-03 |
| CDKAL1 | -0.307 | Downregulated | 2.91E-05 | 3.69E-03 |
| TMEM62 | -0.307 | Downregulated | 1.87E-05 | 2.89E-03 |
| LGALS9 | -0.306 | Downregulated | 2.83E-03 | 4.88E-02 |
| EMP1 | -0.306 | Downregulated | 2.91E-03 | 4.96E-02 |
| LAMA5 | -0.306 | Downregulated | 7.57E-03 | 8.78E-02 |
| ST14 | -0.306 | Downregulated | 3.39E-04 | 1.54E-02 |
| EZR | -0.305 | Downregulated | 4.89E-05 | 5.09E-03 |
| ETFB | -0.305 | Downregulated | 3.01E-06 | 1.00E-03 |
| PPAN | -0.304 | Downregulated | 3.62E-04 | 1.59E-02 |
| MRPL12 | -0.304 | Downregulated | 7.73E-04 | 2.41E-02 |
| SKP2 | -0.304 | Downregulated | 2.58E-05 | 3.43E-03 |
| LGALS1 | -0.304 | Downregulated | 5.31E-04 | 1.94E-02 |
| ASPM | -0.304 | Downregulated | 2.04E-02 | 1.53E-01 |
| ZNHIT2 | -0.303 | Downregulated | 1.67E-04 | 1.03E-02 |
| FLJ33590 | -0.303 | Downregulated | 2.88E-02 | 1.86E-01 |
| ACER3 | -0.303 | Downregulated | 2.86E-03 | 4.92E-02 |
| CRYBB2 | -0.302 | Downregulated | 6.13E-03 | 7.76E-02 |
| NPM3 | -0.302 | Downregulated | 7.90E-04 | 2.44E-02 |
| FIBP | -0.302 | Downregulated | 6.49E-09 | 6.92E-05 |
| TAF15 | -0.302 | Downregulated | 3.73E-06 | 1.12E-03 |
| DEFB1 | -0.300 | Downregulated | 2.92E-02 | 1.87E-01 |
| BPNT1 | -0.300 | Downregulated | 2.38E-03 | 4.39E-02 |
| POLR2D | -0.299 | Downregulated | 2.59E-07 | 3.45E-04 |
| FUBP1 | -0.298 | Downregulated | 1.03E-06 | 6.02E-04 |
| VCX | -0.297 | Downregulated | 2.83E-02 | 1.84E-01 |
| PKM2 | -0.296 | Downregulated | 2.09E-03 | 4.07E-02 |

| | | | | |
|---|---|---|---|---|
| ZNF121 | -0.296 | Downregulated | 8.52E-03 | 9.42E-02 |
| SFXN5 | -0.296 | Downregulated | 1.18E-03 | 2.95E-02 |
| MT1F | -0.295 | Downregulated | 4.68E-03 | 6.61E-02 |
| WDR79 | -0.295 | Downregulated | 2.44E-05 | 3.33E-03 |
| MSLN | -0.294 | Downregulated | 8.63E-03 | 9.48E-02 |
| REEP1 | -0.294 | Downregulated | 3.75E-02 | 2.16E-01 |
| BLK | -0.294 | Downregulated | 2.35E-02 | 1.66E-01 |
| PPP3CC | -0.293 | Downregulated | 7.09E-04 | 2.28E-02 |
| SRR | -0.293 | Downregulated | 3.24E-05 | 3.96E-03 |
| IMP4 | -0.293 | Downregulated | 1.47E-04 | 9.52E-03 |
| MYH10 | -0.293 | Downregulated | 2.68E-03 | 4.72E-02 |
| FLJ11795 | -0.293 | Downregulated | 4.18E-02 | 2.30E-01 |
| PAICS | -0.292 | Downregulated | 2.77E-03 | 4.81E-02 |
| ALG8 | -0.292 | Downregulated | 1.45E-04 | 9.43E-03 |
| SUV39H1 | -0.292 | Downregulated | 1.11E-03 | 2.88E-02 |
| BCS1L | -0.292 | Downregulated | 3.08E-04 | 1.47E-02 |
| ATPAF2 | -0.291 | Downregulated | 6.61E-06 | 1.54E-03 |
| SFXN1 | -0.291 | Downregulated | 1.36E-03 | 3.19E-02 |
| NIT2 | -0.291 | Downregulated | 1.00E-04 | 7.71E-03 |
| UCKL1 | -0.290 | Downregulated | 5.73E-06 | 1.44E-03 |
| RFC4 | -0.290 | Downregulated | 3.78E-03 | 5.81E-02 |
| MEI1 | -0.290 | Downregulated | 2.86E-04 | 1.41E-02 |
| C2CD4B | -0.289 | Downregulated | 9.44E-05 | 7.49E-03 |
| POLD1 | -0.289 | Downregulated | 3.10E-04 | 1.47E-02 |
| MYO1D | -0.288 | Downregulated | 6.48E-03 | 7.99E-02 |
| CSTB | -0.288 | Downregulated | 1.04E-06 | 6.02E-04 |
| ILDR1 | -0.288 | Downregulated | 1.61E-03 | 3.51E-02 |
| TBKBP1 | -0.287 | Downregulated | 2.62E-03 | 4.66E-02 |
| CCDC99 | -0.287 | Downregulated | 1.37E-02 | 1.22E-01 |
| MMACHC | -0.287 | Downregulated | 2.80E-04 | 1.39E-02 |
| PREB | -0.287 | Downregulated | 1.54E-06 | 7.44E-04 |
| NDUFS8 | -0.287 | Downregulated | 6.43E-05 | 5.98E-03 |
| ZNF260 | -0.287 | Downregulated | 1.51E-03 | 3.39E-02 |
| WDR54 | -0.286 | Downregulated | 3.17E-03 | 5.20E-02 |
| CHRNB1 | -0.286 | Downregulated | 1.82E-07 | 3.10E-04 |
| BARD1 | -0.286 | Downregulated | 1.55E-03 | 3.44E-02 |
| AEBP1 | -0.286 | Downregulated | 9.74E-03 | 1.01E-01 |
| FAM64A | -0.285 | Downregulated | 5.29E-04 | 1.94E-02 |
| UHRF1 | -0.285 | Downregulated | 2.55E-03 | 4.59E-02 |
| GAB3 | -0.285 | Downregulated | 1.40E-04 | 9.29E-03 |
| FDX1L | -0.285 | Downregulated | 4.34E-06 | 1.25E-03 |
| MRPS9 | -0.284 | Downregulated | 1.36E-04 | 9.18E-03 |
| PRMT7 | -0.284 | Downregulated | 8.48E-04 | 2.52E-02 |
| NSDHL | -0.284 | Downregulated | 5.91E-08 | 1.86E-04 |
| TUBB2C | -0.283 | Downregulated | 7.50E-05 | 6.66E-03 |
| PWP1 | -0.283 | Downregulated | 3.96E-04 | 1.68E-02 |
| SPRED1 | -0.283 | Downregulated | 1.70E-02 | 1.38E-01 |

| | | | | |
|---|---|---|---|---|
| LRRC56 | -0.283 | Downregulated | 2.50E-04 | 1.30E-02 |
| GSTM4 | -0.283 | Downregulated | 2.21E-03 | 4.19E-02 |
| ZFP82 | -0.282 | Downregulated | 1.48E-03 | 3.35E-02 |
| MAPK13 | -0.282 | Downregulated | 2.26E-03 | 4.25E-02 |
| TROAP | -0.282 | Downregulated | 1.01E-03 | 2.74E-02 |
| RICH2 | -0.282 | Downregulated | 1.95E-03 | 3.92E-02 |
| CHI3L2 | -0.282 | Downregulated | 4.24E-02 | 2.32E-01 |
| COX5A | -0.282 | Downregulated | 4.99E-05 | 5.14E-03 |
| ERCC5 | -0.282 | Downregulated | 1.83E-04 | 1.08E-02 |
| AHI1 | -0.282 | Downregulated | 1.17E-02 | 1.12E-01 |
| TUBG1 | -0.282 | Downregulated | 3.01E-04 | 1.45E-02 |
| RRP9 | -0.282 | Downregulated | 1.93E-04 | 1.11E-02 |
| CSRP1 | -0.282 | Downregulated | 1.64E-06 | 7.58E-04 |
| POLA1 | -0.282 | Downregulated | 1.55E-03 | 3.43E-02 |
| LSM2 | -0.282 | Downregulated | 2.34E-05 | 3.24E-03 |
| SLC25A29 | -0.281 | Downregulated | 1.16E-02 | 1.12E-01 |
| B9D1 | -0.281 | Downregulated | 9.80E-04 | 2.69E-02 |
| TMEM155 | -0.280 | Downregulated | 7.74E-03 | 8.91E-02 |
| PNOC | -0.280 | Downregulated | 2.25E-02 | 1.62E-01 |
| SAMM50 | -0.279 | Downregulated | 1.90E-04 | 1.11E-02 |
| TCF4 | -0.279 | Downregulated | 7.31E-04 | 2.33E-02 |
| HIRIP3 | -0.279 | Downregulated | 8.84E-05 | 7.34E-03 |
| WDR92 | -0.279 | Downregulated | 7.03E-04 | 2.28E-02 |
| MLF1IP | -0.279 | Downregulated | 3.90E-03 | 5.91E-02 |
| PSMA5 | -0.279 | Downregulated | 1.79E-06 | 7.94E-04 |
| BAG1 | -0.278 | Downregulated | 7.87E-04 | 2.44E-02 |
| ENTPD7 | -0.278 | Downregulated | 3.19E-03 | 5.20E-02 |
| CHAF1A | -0.277 | Downregulated | 1.30E-03 | 3.11E-02 |
| WDR25 | -0.277 | Downregulated | 4.78E-05 | 5.02E-03 |
| ENDOG | -0.277 | Downregulated | 9.86E-04 | 2.70E-02 |
| POLR2F | -0.276 | Downregulated | 2.00E-07 | 3.15E-04 |
| WIBG | -0.276 | Downregulated | 4.57E-05 | 4.93E-03 |
| KRTCAP2 | -0.276 | Downregulated | 2.36E-05 | 3.25E-03 |
| MIXL1 | -0.275 | Downregulated | 3.46E-03 | 5.47E-02 |
| ICA1 | -0.275 | Downregulated | 8.08E-03 | 9.16E-02 |
| PSMB6 | -0.274 | Downregulated | 1.28E-05 | 2.33E-03 |
| ITIH4 | -0.274 | Downregulated | 2.78E-03 | 4.82E-02 |
| DHCR24 | -0.274 | Downregulated | 2.69E-03 | 4.73E-02 |
| SYF2 | -0.274 | Downregulated | 7.91E-04 | 2.44E-02 |
| PLK4 | -0.274 | Downregulated | 1.45E-02 | 1.26E-01 |
| PDIA4 | -0.274 | Downregulated | 3.66E-03 | 5.70E-02 |
| ST7L | -0.274 | Downregulated | 3.09E-06 | 1.00E-03 |
| LMNB2 | -0.274 | Downregulated | 1.47E-03 | 3.33E-02 |
| GINS3 | -0.273 | Downregulated | 3.58E-03 | 5.62E-02 |
| TMEM14A | -0.273 | Downregulated | 2.28E-02 | 1.63E-01 |
| ATXN7L1 | -0.273 | Downregulated | 1.74E-03 | 3.68E-02 |
| NAV1 | -0.273 | Downregulated | 2.23E-03 | 4.22E-02 |

| | | | | |
|---|---|---|---|---|
| LDHA | -0.273 | Downregulated | 7.10E-05 | 6.44E-03 |
| ANKRD52 | -0.272 | Downregulated | 1.81E-06 | 7.94E-04 |
| HIBCH | -0.272 | Downregulated | 3.17E-04 | 1.49E-02 |
| ACTG2 | -0.272 | Downregulated | 4.10E-02 | 2.27E-01 |
| MASTL | -0.271 | Downregulated | 9.69E-04 | 2.67E-02 |
| CYC1 | -0.271 | Downregulated | 1.21E-06 | 6.57E-04 |
| TIMM44 | -0.271 | Downregulated | 5.88E-04 | 2.06E-02 |
| OIP5 | -0.271 | Downregulated | 6.69E-03 | 8.14E-02 |
| KIF11 | -0.271 | Downregulated | 3.56E-02 | 2.10E-01 |
| NUSAP1 | -0.270 | Downregulated | 1.44E-02 | 1.26E-01 |
| ARPC5L | -0.270 | Downregulated | 1.01E-04 | 7.71E-03 |
| SFTPD | -0.270 | Downregulated | 3.16E-03 | 5.18E-02 |
| CDCA8 | -0.270 | Downregulated | 2.10E-03 | 4.07E-02 |
| MAP2K5 | -0.270 | Downregulated | 1.57E-04 | 9.90E-03 |
| PPP1R14A | -0.270 | Downregulated | 3.46E-03 | 5.47E-02 |
| POLA2 | -0.269 | Downregulated | 7.79E-05 | 6.76E-03 |
| UBE2G1 | -0.269 | Downregulated | 7.19E-08 | 2.08E-04 |
| CCDC92 | -0.269 | Downregulated | 4.21E-04 | 1.75E-02 |
| EIF2A | -0.268 | Downregulated | 1.14E-03 | 2.92E-02 |
| CST7 | -0.268 | Downregulated | 3.20E-02 | 1.98E-01 |
| CASC1 | -0.268 | Downregulated | 1.22E-02 | 1.15E-01 |
| ACTN4 | -0.268 | Downregulated | 4.40E-05 | 4.88E-03 |
| DCTPP1 | -0.268 | Downregulated | 6.81E-05 | 6.28E-03 |
| PPP1R11 | -0.268 | Downregulated | 5.51E-05 | 5.37E-03 |
| MPP6 | -0.268 | Downregulated | 1.37E-02 | 1.22E-01 |
| ALG9 | -0.268 | Downregulated | 9.75E-05 | 7.62E-03 |
| NKG7 | -0.268 | Downregulated | 9.38E-03 | 9.89E-02 |
| CD70 | -0.268 | Downregulated | 3.34E-03 | 5.36E-02 |
| GPR137B | -0.267 | Downregulated | 3.64E-03 | 5.69E-02 |
| PLAC8 | -0.267 | Downregulated | 2.89E-03 | 4.94E-02 |
| EXOSC4 | -0.267 | Downregulated | 1.41E-02 | 1.24E-01 |
| EPC1 | -0.266 | Downregulated | 3.02E-03 | 5.05E-02 |
| MRPL38 | -0.266 | Downregulated | 5.82E-04 | 2.05E-02 |
| PUSL1 | -0.266 | Downregulated | 3.08E-04 | 1.47E-02 |
| SETBP1 | -0.265 | Downregulated | 1.15E-03 | 2.92E-02 |
| PMM2 | -0.265 | Downregulated | 3.40E-04 | 1.54E-02 |
| MRPL40 | -0.265 | Downregulated | 4.07E-03 | 6.08E-02 |
| MED21 | 0.265 | Upregulated | 1.42E-03 | 3.26E-02 |
| CNIH4 | 0.265 | Upregulated | 3.17E-02 | 1.97E-01 |
| ALS2CR14 | 0.266 | Upregulated | 3.38E-02 | 2.03E-01 |
| DPYS | 0.266 | Upregulated | 2.44E-03 | 4.45E-02 |
| FRMD3 | 0.267 | Upregulated | 4.08E-02 | 2.27E-01 |
| NCOA1 | 0.267 | Upregulated | 1.79E-02 | 1.42E-01 |
| FLVCR2 | 0.267 | Upregulated | 1.04E-02 | 1.05E-01 |
| PPP2R5B | 0.268 | Upregulated | 6.61E-03 | 8.08E-02 |
| MCTP1 | 0.268 | Upregulated | 2.09E-03 | 4.07E-02 |
| PTPN2 | 0.268 | Upregulated | 1.60E-03 | 3.50E-02 |

| | | | | |
|---|---|---|---|---|
| KRT3 | 0.268 | Upregulated | 3.04E-04 | 1.46E-02 |
| MEGF9 | 0.268 | Upregulated | 3.48E-02 | 2.07E-01 |
| SMNDC1 | 0.269 | Upregulated | 5.65E-03 | 7.41E-02 |
| PKN2 | 0.269 | Upregulated | 4.65E-03 | 6.58E-02 |
| CPD | 0.270 | Upregulated | 1.76E-02 | 1.40E-01 |
| SNAP23 | 0.270 | Upregulated | 5.11E-03 | 6.97E-02 |
| HCG9 | 0.270 | Upregulated | 1.18E-02 | 1.13E-01 |
| MXD1 | 0.270 | Upregulated | 3.78E-02 | 2.17E-01 |
| FURIN | 0.270 | Upregulated | 1.91E-02 | 1.47E-01 |
| JMJD1A | 0.271 | Upregulated | 1.11E-02 | 1.09E-01 |
| NAP1L1 | 0.271 | Upregulated | 4.32E-02 | 2.34E-01 |
| NLK | 0.271 | Upregulated | 4.01E-02 | 2.25E-01 |
| SLC25A32 | 0.272 | Upregulated | 4.20E-03 | 6.21E-02 |
| MOBKL1B | 0.272 | Upregulated | 1.80E-02 | 1.42E-01 |
| OSBPL11 | 0.272 | Upregulated | 6.03E-03 | 7.70E-02 |
| NARG1L | 0.272 | Upregulated | 8.48E-06 | 1.75E-03 |
| WDR26 | 0.273 | Upregulated | 1.82E-04 | 1.08E-02 |
| ZNF23 | 0.273 | Upregulated | 1.64E-04 | 1.02E-02 |
| ATF1 | 0.273 | Upregulated | 9.08E-03 | 9.70E-02 |
| CECR6 | 0.273 | Upregulated | 3.26E-02 | 2.00E-01 |
| MCOLN1 | 0.274 | Upregulated | 1.34E-02 | 1.20E-01 |
| HAL | 0.275 | Upregulated | 2.56E-02 | 1.74E-01 |
| TMSL3 | 0.275 | Upregulated | 1.96E-03 | 3.92E-02 |
| SAMSN1 | 0.275 | Upregulated | 1.93E-02 | 1.48E-01 |
| ZFYVE16 | 0.275 | Upregulated | 6.91E-05 | 6.31E-03 |
| IL6R | 0.276 | Upregulated | 1.68E-02 | 1.37E-01 |
| EIF2AK4 | 0.276 | Upregulated | 2.15E-02 | 1.57E-01 |
| PNPLA8 | 0.277 | Upregulated | 6.79E-03 | 8.23E-02 |
| PRPH | 0.278 | Upregulated | 2.34E-03 | 4.35E-02 |
| NAB1 | 0.278 | Upregulated | 1.11E-03 | 2.89E-02 |
| ARAP2 | 0.279 | Upregulated | 4.28E-04 | 1.76E-02 |
| SPTA1 | 0.281 | Upregulated | 3.54E-02 | 2.09E-01 |
| CEP27 | 0.282 | Upregulated | 3.54E-02 | 2.09E-01 |
| CDKL1 | 0.283 | Upregulated | 9.57E-04 | 2.66E-02 |
| ZNF641 | 0.283 | Upregulated | 3.14E-02 | 1.96E-01 |
| CYB561 | 0.283 | Upregulated | 1.64E-05 | 2.70E-03 |
| F11R | 0.283 | Upregulated | 4.95E-04 | 1.88E-02 |
| USP12 | 0.284 | Upregulated | 3.43E-03 | 5.45E-02 |
| CCNT2 | 0.284 | Upregulated | 8.33E-03 | 9.29E-02 |
| TRIML2 | 0.284 | Upregulated | 5.05E-05 | 5.16E-03 |
| PPP1R12A | 0.284 | Upregulated | 1.96E-02 | 1.49E-01 |
| ZFAND6 | 0.286 | Upregulated | 1.89E-05 | 2.91E-03 |
| PFDN5 | 0.286 | Upregulated | 4.89E-02 | 2.50E-01 |
| CCDC125 | 0.286 | Upregulated | 2.47E-02 | 1.71E-01 |
| NXT2 | 0.286 | Upregulated | 1.32E-02 | 1.19E-01 |
| DPYD | 0.287 | Upregulated | 1.01E-02 | 1.03E-01 |
| KBTBD7 | 0.287 | Upregulated | 1.22E-02 | 1.15E-01 |

| | | | | |
|---|---|---|---|---|
| BIN2 | 0.287 | Upregulated | 2.54E-02 | 1.73E-01 |
| BIRC2 | 0.287 | Upregulated | 5.57E-04 | 1.99E-02 |
| EIF1AD | 0.287 | Upregulated | 6.24E-03 | 7.85E-02 |
| HIAT1 | 0.288 | Upregulated | 6.57E-04 | 2.20E-02 |
| TIPRL | 0.288 | Upregulated | 5.45E-03 | 7.25E-02 |
| DNAJA4 | 0.289 | Upregulated | 3.59E-02 | 2.11E-01 |
| CTBS | 0.289 | Upregulated | 7.86E-03 | 8.99E-02 |
| TAOK1 | 0.289 | Upregulated | 8.19E-03 | 9.22E-02 |
| BAGE2 | 0.289 | Upregulated | 5.30E-05 | 5.24E-03 |
| PTAFR | 0.290 | Upregulated | 3.81E-02 | 2.18E-01 |
| UHMK1 | 0.291 | Upregulated | 1.02E-03 | 2.74E-02 |
| ZNF860 | 0.291 | Upregulated | 1.11E-02 | 1.09E-01 |
| LACTB | 0.292 | Upregulated | 1.52E-02 | 1.30E-01 |
| GADD45G | 0.292 | Upregulated | 2.67E-02 | 1.78E-01 |
| DDX17 | 0.292 | Upregulated | 3.25E-03 | 5.27E-02 |
| ABHD13 | 0.293 | Upregulated | 3.40E-06 | 1.08E-03 |
| TRIM38 | 0.293 | Upregulated | 5.58E-04 | 1.99E-02 |
| CLTC | 0.293 | Upregulated | 5.14E-03 | 6.99E-02 |
| APOL1 | 0.293 | Upregulated | 8.68E-04 | 2.55E-02 |
| RPS28 | 0.294 | Upregulated | 4.62E-02 | 2.42E-01 |
| UBE1C | 0.294 | Upregulated | 2.80E-04 | 1.39E-02 |
| HSPA6 | 0.294 | Upregulated | 2.49E-02 | 1.71E-01 |
| UBE2B | 0.294 | Upregulated | 1.86E-03 | 3.83E-02 |
| CAPZA1 | 0.294 | Upregulated | 1.81E-03 | 3.77E-02 |
| CTSS | 0.294 | Upregulated | 9.20E-03 | 9.77E-02 |
| JMJD1C | 0.295 | Upregulated | 6.93E-04 | 2.26E-02 |
| CD55 | 0.295 | Upregulated | 1.75E-02 | 1.40E-01 |
| SFRS12 | 0.296 | Upregulated | 2.64E-04 | 1.36E-02 |
| RILP | 0.296 | Upregulated | 7.41E-03 | 8.65E-02 |
| GPX8 | 0.296 | Upregulated | 1.01E-02 | 1.03E-01 |
| PRDM8 | 0.297 | Upregulated | 2.70E-06 | 9.87E-04 |
| KIAA0247 | 0.297 | Upregulated | 5.79E-04 | 2.05E-02 |
| PLXDC2 | 0.297 | Upregulated | 1.38E-02 | 1.23E-01 |
| DMXL1 | 0.298 | Upregulated | 1.35E-03 | 3.17E-02 |
| IL1F9 | 0.298 | Upregulated | 6.93E-03 | 8.34E-02 |
| IGSF6 | 0.298 | Upregulated | 2.43E-02 | 1.69E-01 |
| FOS | 0.298 | Upregulated | 3.62E-02 | 2.12E-01 |
| CYP4F12 | 0.300 | Upregulated | 5.18E-03 | 7.02E-02 |
| DCTN4 | 0.300 | Upregulated | 6.48E-04 | 2.18E-02 |
| BEST1 | 0.301 | Upregulated | 4.54E-02 | 2.40E-01 |
| PABPC1 | 0.301 | Upregulated | 1.59E-03 | 3.49E-02 |
| DSC2 | 0.301 | Upregulated | 3.03E-02 | 1.92E-01 |
| USP15 | 0.302 | Upregulated | 6.25E-04 | 2.13E-02 |
| KLHDC8B | 0.302 | Upregulated | 4.31E-02 | 2.33E-01 |
| KCNJ10 | 0.302 | Upregulated | 6.51E-03 | 8.00E-02 |
| SH3YL1 | 0.302 | Upregulated | 2.21E-03 | 4.19E-02 |
| CXCL1 | 0.303 | Upregulated | 8.28E-03 | 9.26E-02 |

| | | | | |
|---|---|---|---|---|
| PPM2C | 0.303 | Upregulated | 2.88E-03 | 4.93E-02 |
| ARL17P1 | 0.303 | Upregulated | 3.80E-03 | 5.82E-02 |
| ABCA13 | 0.305 | Upregulated | 3.81E-02 | 2.18E-01 |
| EVI5 | 0.305 | Upregulated | 2.03E-02 | 1.52E-01 |
| RP1L1 | 0.305 | Upregulated | 1.31E-02 | 1.18E-01 |
| ITLN1 | 0.306 | Upregulated | 3.27E-02 | 2.00E-01 |
| OPTN | 0.306 | Upregulated | 8.65E-03 | 9.49E-02 |
| ARID4A | 0.306 | Upregulated | 3.73E-04 | 1.62E-02 |
| RBM7 | 0.307 | Upregulated | 1.66E-02 | 1.36E-01 |
| DUSP3 | 0.307 | Upregulated | 3.01E-03 | 5.05E-02 |
| TAGAP | 0.308 | Upregulated | 9.23E-05 | 7.47E-03 |
| CAMK2A | 0.308 | Upregulated | 1.22E-02 | 1.15E-01 |
| CCR1 | 0.309 | Upregulated | 4.92E-02 | 2.51E-01 |
| POTEE | 0.309 | Upregulated | 6.82E-03 | 8.24E-02 |
| HNRPC | 0.309 | Upregulated | 4.93E-03 | 6.82E-02 |
| ECHDC1 | 0.309 | Upregulated | 8.83E-04 | 2.56E-02 |
| GBP4 | 0.309 | Upregulated | 2.04E-02 | 1.53E-01 |
| HLX | 0.309 | Upregulated | 1.62E-02 | 1.34E-01 |
| WDR51B | 0.310 | Upregulated | 3.38E-04 | 1.54E-02 |
| PLAGL1 | 0.310 | Upregulated | 4.96E-04 | 1.88E-02 |
| CISD2 | 0.311 | Upregulated | 4.40E-02 | 2.36E-01 |
| RAB6A | 0.312 | Upregulated | 9.88E-04 | 2.70E-02 |
| CPPED1 | 0.313 | Upregulated | 3.66E-03 | 5.70E-02 |
| RAB2B | 0.313 | Upregulated | 1.20E-02 | 1.14E-01 |
| MBNL1 | 0.314 | Upregulated | 8.51E-04 | 2.52E-02 |
| RNF13 | 0.315 | Upregulated | 4.40E-04 | 1.78E-02 |
| TSC22D1 | 0.316 | Upregulated | 4.95E-03 | 6.84E-02 |
| NR1D1 | 0.316 | Upregulated | 1.08E-02 | 1.07E-01 |
| GSPT1 | 0.316 | Upregulated | 2.22E-02 | 1.60E-01 |
| PTPLAD2 | 0.316 | Upregulated | 2.98E-02 | 1.90E-01 |
| CROP | 0.316 | Upregulated | 1.17E-02 | 1.12E-01 |
| FLJ20489 | 0.317 | Upregulated | 3.13E-02 | 1.95E-01 |
| HSPA4 | 0.318 | Upregulated | 1.12E-04 | 8.12E-03 |
| MICAL2 | 0.319 | Upregulated | 1.29E-02 | 1.18E-01 |
| KRCC1 | 0.319 | Upregulated | 1.31E-04 | 8.97E-03 |
| PTPRC | 0.319 | Upregulated | 7.03E-03 | 8.39E-02 |
| RRM2B | 0.319 | Upregulated | 2.00E-03 | 3.97E-02 |
| RNF149 | 0.319 | Upregulated | 6.35E-03 | 7.91E-02 |
| RBM12B | 0.320 | Upregulated | 3.40E-04 | 1.54E-02 |
| NMI | 0.321 | Upregulated | 5.10E-04 | 1.91E-02 |
| B3GNT5 | 0.322 | Upregulated | 5.47E-03 | 7.26E-02 |
| EPB41 | 0.322 | Upregulated | 3.41E-02 | 2.04E-01 |
| TBC1D10B | 0.323 | Upregulated | 6.36E-03 | 7.91E-02 |
| PPIB | 0.323 | Upregulated | 1.32E-02 | 1.19E-01 |
| SLC45A3 | 0.323 | Upregulated | 5.91E-03 | 7.63E-02 |
| ALDH5A1 | 0.323 | Upregulated | 5.97E-04 | 2.08E-02 |
| DNTTIP2 | 0.324 | Upregulated | 2.91E-03 | 4.97E-02 |

| | | | | |
|---|---|---|---|---|
| RBM47 | 0.324 | Upregulated | 1.43E-02 | 1.25E-01 |
| NPTN | 0.324 | Upregulated | 5.41E-03 | 7.22E-02 |
| PLA2G4A | 0.325 | Upregulated | 1.85E-05 | 2.89E-03 |
| SPATA13 | 0.325 | Upregulated | 3.89E-02 | 2.21E-01 |
| MAP2K3 | 0.325 | Upregulated | 1.25E-02 | 1.16E-01 |
| GYPA | 0.325 | Upregulated | 8.24E-03 | 9.24E-02 |
| CCNDBP1 | 0.325 | Upregulated | 3.62E-03 | 5.67E-02 |
| BPGM | 0.326 | Upregulated | 4.10E-02 | 2.28E-01 |
| TOPORS | 0.326 | Upregulated | 1.86E-07 | 3.10E-04 |
| CREBBP | 0.328 | Upregulated | 1.95E-04 | 1.11E-02 |
| EIF1B | 0.328 | Upregulated | 7.67E-04 | 2.40E-02 |
| STEAP4 | 0.328 | Upregulated | 3.98E-02 | 2.24E-01 |
| KEL | 0.328 | Upregulated | 1.80E-02 | 1.42E-01 |
| KIR3DS1 | 0.329 | Upregulated | 2.35E-02 | 1.66E-01 |
| TMEM86B | 0.329 | Upregulated | 3.40E-02 | 2.04E-01 |
| TMEM49 | 0.329 | Upregulated | 3.65E-04 | 1.60E-02 |
| MBNL2 | 0.329 | Upregulated | 4.47E-03 | 6.43E-02 |
| YPEL4 | 0.329 | Upregulated | 7.22E-03 | 8.51E-02 |
| YOD1 | 0.329 | Upregulated | 1.04E-03 | 2.76E-02 |
| ILK | 0.332 | Upregulated | 1.49E-02 | 1.28E-01 |
| REM2 | 0.332 | Upregulated | 1.75E-02 | 1.40E-01 |
| ISCA1L | 0.333 | Upregulated | 7.72E-05 | 6.75E-03 |
| RAB11FIP1 | 0.333 | Upregulated | 1.40E-03 | 3.23E-02 |
| RNF130 | 0.333 | Upregulated | 3.70E-03 | 5.74E-02 |
| EAF1 | 0.334 | Upregulated | 1.18E-03 | 2.96E-02 |
| FLI1 | 0.335 | Upregulated | 2.95E-03 | 5.01E-02 |
| ZBTB44 | 0.337 | Upregulated | 6.31E-04 | 2.14E-02 |
| GABARAPL2 | 0.337 | Upregulated | 1.55E-04 | 9.78E-03 |
| CXCR1 | 0.337 | Upregulated | 4.23E-02 | 2.31E-01 |
| GCA | 0.338 | Upregulated | 1.10E-02 | 1.08E-01 |
| BAGE3 | 0.338 | Upregulated | 3.52E-03 | 5.54E-02 |
| ARHGAP25 | 0.338 | Upregulated | 3.15E-04 | 1.49E-02 |
| CBX3 | 0.339 | Upregulated | 4.10E-04 | 1.72E-02 |
| DUSP1 | 0.339 | Upregulated | 3.14E-02 | 1.96E-01 |
| CD97 | 0.339 | Upregulated | 2.58E-03 | 4.63E-02 |
| NAT13 | 0.339 | Upregulated | 4.11E-04 | 1.72E-02 |
| ISCA1 | 0.340 | Upregulated | 9.64E-04 | 2.67E-02 |
| FCHO2 | 0.341 | Upregulated | 1.57E-02 | 1.32E-01 |
| ANP32A | 0.341 | Upregulated | 1.35E-03 | 3.17E-02 |
| SLC31A2 | 0.342 | Upregulated | 1.09E-02 | 1.08E-01 |
| ZNF223 | 0.342 | Upregulated | 2.43E-02 | 1.69E-01 |
| IL13RA1 | 0.342 | Upregulated | 8.24E-03 | 9.24E-02 |
| GHRL | 0.342 | Upregulated | 1.00E-02 | 1.03E-01 |
| DMXL2 | 0.345 | Upregulated | 8.21E-04 | 2.48E-02 |
| SAP30 | 0.346 | Upregulated | 2.17E-02 | 1.58E-01 |
| FAM129A | 0.346 | Upregulated | 1.27E-02 | 1.17E-01 |
| SUMO4 | 0.347 | Upregulated | 1.36E-06 | 6.96E-04 |

| | | | | |
|---|---|---|---|---|
| FAM116B | 0.348 | Upregulated | 4.41E-03 | 6.38E-02 |
| SLK | 0.348 | Upregulated | 9.94E-04 | 2.71E-02 |
| GIMAP1 | 0.349 | Upregulated | 1.67E-02 | 1.37E-01 |
| TRIM10 | 0.349 | Upregulated | 1.17E-02 | 1.12E-01 |
| IFP38 | 0.349 | Upregulated | 1.60E-03 | 3.50E-02 |
| CSNK1A1 | 0.349 | Upregulated | 2.48E-07 | 3.45E-04 |
| HSD17B11 | 0.350 | Upregulated | 9.72E-04 | 2.68E-02 |
| APOL6 | 0.351 | Upregulated | 2.96E-03 | 5.02E-02 |
| PROS1 | 0.351 | Upregulated | 3.00E-02 | 1.91E-01 |
| CA2 | 0.351 | Upregulated | 3.26E-02 | 2.00E-01 |
| OR51S1 | 0.351 | Upregulated | 1.95E-04 | 1.11E-02 |
| IL1B | 0.352 | Upregulated | 4.88E-02 | 2.50E-01 |
| MS4A2 | 0.352 | Upregulated | 6.17E-04 | 2.12E-02 |
| TNFRSF10B | 0.354 | Upregulated | 1.04E-02 | 1.05E-01 |
| AIM2 | 0.355 | Upregulated | 4.38E-04 | 1.78E-02 |
| WDR23 | 0.356 | Upregulated | 3.10E-04 | 1.47E-02 |
| TTC32 | 0.356 | Upregulated | 7.98E-09 | 6.92E-05 |
| PRRG4 | 0.357 | Upregulated | 5.46E-05 | 5.34E-03 |
| SERPING1 | 0.358 | Upregulated | 7.56E-03 | 8.78E-02 |
| SEC14L1 | 0.359 | Upregulated | 9.47E-04 | 2.65E-02 |
| ZBTB11 | 0.359 | Upregulated | 1.77E-03 | 3.72E-02 |
| ARL4A | 0.359 | Upregulated | 5.48E-03 | 7.27E-02 |
| UBE2O | 0.360 | Upregulated | 2.73E-02 | 1.80E-01 |
| SF3B1 | 0.361 | Upregulated | 8.64E-05 | 7.26E-03 |
| PMAIP1 | 0.361 | Upregulated | 9.04E-05 | 7.38E-03 |
| MCTS1 | 0.361 | Upregulated | 1.33E-02 | 1.20E-01 |
| MAP1LC3B2 | 0.361 | Upregulated | 1.90E-03 | 3.87E-02 |
| EVI2A | 0.362 | Upregulated | 1.22E-02 | 1.15E-01 |
| FAS | 0.363 | Upregulated | 2.73E-03 | 4.76E-02 |
| VTI1B | 0.363 | Upregulated | 2.50E-04 | 1.30E-02 |
| HIST1H2BC | 0.363 | Upregulated | 1.69E-02 | 1.38E-01 |
| ST6GALNAC4 | 0.364 | Upregulated | 1.21E-02 | 1.14E-01 |
| SDPR | 0.364 | Upregulated | 6.71E-03 | 8.15E-02 |
| RNF10 | 0.367 | Upregulated | 5.28E-03 | 7.12E-02 |
| GATA2 | 0.367 | Upregulated | 1.49E-03 | 3.35E-02 |
| PDCD1LG2 | 0.367 | Upregulated | 1.53E-02 | 1.30E-01 |
| CAT | 0.368 | Upregulated | 4.65E-04 | 1.83E-02 |
| TANK | 0.368 | Upregulated | 1.39E-05 | 2.39E-03 |
| GRINA | 0.368 | Upregulated | 6.93E-03 | 8.34E-02 |
| KCTD12 | 0.371 | Upregulated | 1.06E-03 | 2.79E-02 |
| HIST1H2BE | 0.371 | Upregulated | 1.23E-02 | 1.15E-01 |
| PHLDB1 | 0.372 | Upregulated | 5.66E-03 | 7.42E-02 |
| RNASEH2B | 0.373 | Upregulated | 1.26E-02 | 1.17E-01 |
| HEPACAM2 | 0.373 | Upregulated | 1.98E-02 | 1.50E-01 |
| GATA1 | 0.374 | Upregulated | 6.60E-03 | 8.08E-02 |
| KIFC3 | 0.374 | Upregulated | 2.13E-03 | 4.09E-02 |
| GRAMD1B | 0.374 | Upregulated | 1.65E-02 | 1.36E-01 |

| | | | | |
|---|---|---|---|---|
| ALPK1 | 0.374 | Upregulated | 7.86E-03 | 8.99E-02 |
| SLAMF8 | 0.376 | Upregulated | 2.45E-03 | 4.47E-02 |
| SBDS | 0.377 | Upregulated | 7.00E-04 | 2.27E-02 |
| TXNL1 | 0.377 | Upregulated | 1.19E-02 | 1.14E-01 |
| CPEB4 | 0.377 | Upregulated | 2.19E-03 | 4.18E-02 |
| KLHL15 | 0.378 | Upregulated | 1.18E-02 | 1.13E-01 |
| POLB | 0.378 | Upregulated | 3.41E-06 | 1.08E-03 |
| FHDC1 | 0.379 | Upregulated | 8.15E-04 | 2.47E-02 |
| MTF2 | 0.379 | Upregulated | 4.68E-05 | 4.96E-03 |
| TLR2 | 0.379 | Upregulated | 3.04E-02 | 1.92E-01 |
| YPEL3 | 0.380 | Upregulated | 1.21E-04 | 8.62E-03 |
| RAB24 | 0.381 | Upregulated | 1.17E-03 | 2.95E-02 |
| RPL7 | 0.382 | Upregulated | 7.85E-03 | 8.98E-02 |
| ARID4B | 0.382 | Upregulated | 1.72E-04 | 1.04E-02 |
| ZCCHC6 | 0.383 | Upregulated | 4.23E-05 | 4.82E-03 |
| MYADM | 0.384 | Upregulated | 1.23E-02 | 1.15E-01 |
| HDAC4 | 0.384 | Upregulated | 5.74E-03 | 7.48E-02 |
| PNPLA2 | 0.385 | Upregulated | 3.20E-03 | 5.21E-02 |
| MAOA | 0.387 | Upregulated | 1.38E-02 | 1.23E-01 |
| RNF19A | 0.387 | Upregulated | 1.17E-03 | 2.94E-02 |
| FCGR2C | 0.388 | Upregulated | 7.16E-03 | 8.47E-02 |
| ACSL1 | 0.388 | Upregulated | 2.98E-02 | 1.90E-01 |
| RAP1BL | 0.388 | Upregulated | 1.17E-02 | 1.12E-01 |
| MKRN1 | 0.388 | Upregulated | 4.58E-04 | 1.81E-02 |
| GYPC | 0.389 | Upregulated | 4.80E-03 | 6.71E-02 |
| ANXA4 | 0.389 | Upregulated | 9.65E-03 | 1.01E-01 |
| YBX1 | 0.389 | Upregulated | 5.79E-06 | 1.44E-03 |
| EMB | 0.390 | Upregulated | 2.14E-04 | 1.18E-02 |
| USP6 | 0.390 | Upregulated | 5.39E-04 | 1.96E-02 |
| SSTR2 | 0.390 | Upregulated | 6.97E-03 | 8.37E-02 |
| ACTR3 | 0.391 | Upregulated | 7.72E-03 | 8.89E-02 |
| DHRS9 | 0.391 | Upregulated | 3.24E-03 | 5.26E-02 |
| FBXO38 | 0.391 | Upregulated | 2.92E-04 | 1.43E-02 |
| ANKRD9 | 0.392 | Upregulated | 8.57E-03 | 9.44E-02 |
| PPBP | 0.392 | Upregulated | 9.42E-03 | 9.92E-02 |
| RTF1 | 0.393 | Upregulated | 1.25E-03 | 3.06E-02 |
| SFMBT2 | 0.393 | Upregulated | 3.68E-03 | 5.71E-02 |
| ADI1 | 0.394 | Upregulated | 3.52E-03 | 5.54E-02 |
| OSBPL8 | 0.394 | Upregulated | 1.75E-05 | 2.79E-03 |
| FKBP8 | 0.394 | Upregulated | 4.81E-03 | 6.73E-02 |
| TSC22D3 | 0.396 | Upregulated | 1.43E-02 | 1.25E-01 |
| DAPP1 | 0.397 | Upregulated | 9.69E-05 | 7.59E-03 |
| RPS15A | 0.397 | Upregulated | 3.23E-02 | 1.99E-01 |
| KLF1 | 0.397 | Upregulated | 1.55E-02 | 1.31E-01 |
| TTRAP | 0.398 | Upregulated | 1.91E-03 | 3.88E-02 |
| ELOVL5 | 0.399 | Upregulated | 4.70E-04 | 1.83E-02 |
| PINK1 | 0.399 | Upregulated | 8.33E-04 | 2.49E-02 |

| | | | | |
|---|---|---|---|---|
| HOXC10 | 0.399 | Upregulated | 5.42E-04 | 1.96E-02 |
| OR2W3 | 0.399 | Upregulated | 1.77E-02 | 1.41E-01 |
| PCAF | 0.400 | Upregulated | 2.34E-05 | 3.24E-03 |
| SOD2 | 0.400 | Upregulated | 1.84E-02 | 1.44E-01 |
| CIR1 | 0.403 | Upregulated | 2.73E-05 | 3.59E-03 |
| COL10A1 | 0.404 | Upregulated | 1.01E-03 | 2.73E-02 |
| MYBL1 | 0.404 | Upregulated | 4.15E-03 | 6.16E-02 |
| SPOPL | 0.405 | Upregulated | 1.12E-03 | 2.90E-02 |
| FAM117A | 0.406 | Upregulated | 2.35E-04 | 1.26E-02 |
| FYB | 0.407 | Upregulated | 8.76E-05 | 7.30E-03 |
| TCN1 | 0.409 | Upregulated | 3.61E-02 | 2.12E-01 |
| FCGR3A | 0.409 | Upregulated | 2.47E-05 | 3.34E-03 |
| SPRYD5 | 0.409 | Upregulated | 1.53E-04 | 9.74E-03 |
| KLHL2 | 0.411 | Upregulated | 5.34E-03 | 7.17E-02 |
| BMP2K | 0.412 | Upregulated | 3.64E-07 | 3.87E-04 |
| SACM1L | 0.413 | Upregulated | 6.75E-04 | 2.23E-02 |
| ITPRIPL2 | 0.413 | Upregulated | 1.14E-03 | 2.92E-02 |
| LGALS2 | 0.414 | Upregulated | 1.23E-02 | 1.15E-01 |
| NOD2 | 0.414 | Upregulated | 2.49E-03 | 4.53E-02 |
| SLC14A1 | 0.414 | Upregulated | 1.46E-03 | 3.31E-02 |
| ASCC2 | 0.416 | Upregulated | 6.03E-03 | 7.70E-02 |
| TIMM10 | 0.417 | Upregulated | 3.13E-02 | 1.95E-01 |
| MYL4 | 0.419 | Upregulated | 3.58E-02 | 2.10E-01 |
| CSF2RB | 0.420 | Upregulated | 1.39E-03 | 3.23E-02 |
| KREMEN1 | 0.420 | Upregulated | 7.89E-03 | 9.01E-02 |
| MAP1S | 0.421 | Upregulated | 1.68E-04 | 1.03E-02 |
| CARD17 | 0.421 | Upregulated | 4.85E-03 | 6.75E-02 |
| WNK1 | 0.423 | Upregulated | 9.10E-06 | 1.83E-03 |
| FAM21A | 0.423 | Upregulated | 1.81E-04 | 1.08E-02 |
| CREG1 | 0.424 | Upregulated | 3.78E-03 | 5.81E-02 |
| FAM8A1 | 0.424 | Upregulated | 3.58E-04 | 1.58E-02 |
| GNG10 | 0.425 | Upregulated | 2.11E-03 | 4.08E-02 |
| WSB1 | 0.426 | Upregulated | 7.20E-04 | 2.30E-02 |
| HIF1A | 0.427 | Upregulated | 4.77E-03 | 6.69E-02 |
| DPM2 | 0.428 | Upregulated | 4.01E-03 | 6.02E-02 |
| PTGES3 | 0.431 | Upregulated | 8.73E-03 | 9.52E-02 |
| TNFAIP2 | 0.431 | Upregulated | 6.75E-04 | 2.23E-02 |
| RRAGD | 0.434 | Upregulated | 2.65E-03 | 4.69E-02 |
| METTL7A | 0.438 | Upregulated | 1.03E-03 | 2.76E-02 |
| GPRIN3 | 0.438 | Upregulated | 2.51E-03 | 4.55E-02 |
| SRRD | 0.439 | Upregulated | 3.38E-03 | 5.41E-02 |
| MPP1 | 0.439 | Upregulated | 1.33E-02 | 1.20E-01 |
| FAM104A | 0.439 | Upregulated | 6.82E-04 | 2.25E-02 |
| PSTPIP2 | 0.441 | Upregulated | 4.13E-03 | 6.14E-02 |
| FCGR3B | 0.442 | Upregulated | 1.63E-02 | 1.35E-01 |
| PPP2CB | 0.444 | Upregulated | 4.87E-04 | 1.86E-02 |
| SESTD1 | 0.445 | Upregulated | 5.01E-06 | 1.36E-03 |

| | | | | |
|---|---|---|---|---|
| VPS26 | 0.446 | Upregulated | 6.14E-04 | 2.12E-02 |
| LY96 | 0.448 | Upregulated | 1.26E-02 | 1.16E-01 |
| GK | 0.449 | Upregulated | 8.52E-03 | 9.41E-02 |
| GUK1 | 0.449 | Upregulated | 5.74E-04 | 2.04E-02 |
| ZRANB1 | 0.449 | Upregulated | 1.61E-06 | 7.58E-04 |
| NCF2 | 0.449 | Upregulated | 7.32E-04 | 2.33E-02 |
| LRRK2 | 0.450 | Upregulated | 4.40E-04 | 1.78E-02 |
| NOL10 | 0.452 | Upregulated | 1.29E-03 | 3.11E-02 |
| RIOK3 | 0.454 | Upregulated | 1.40E-03 | 3.23E-02 |
| IGFBP1 | 0.454 | Upregulated | 1.42E-06 | 7.16E-04 |
| HLA-C | 0.456 | Upregulated | 1.18E-02 | 1.13E-01 |
| NFIX | 0.456 | Upregulated | 7.11E-03 | 8.45E-02 |
| KCNJ15 | 0.456 | Upregulated | 5.70E-03 | 7.44E-02 |
| PRDM1 | 0.456 | Upregulated | 4.66E-05 | 4.96E-03 |
| IL1RAP | 0.457 | Upregulated | 8.46E-05 | 7.16E-03 |
| FAM49B | 0.458 | Upregulated | 2.43E-03 | 4.45E-02 |
| CEACAM1 | 0.459 | Upregulated | 3.85E-02 | 2.19E-01 |
| TMEM158 | 0.462 | Upregulated | 1.57E-02 | 1.32E-01 |
| FPR2 | 0.462 | Upregulated | 6.99E-03 | 8.38E-02 |
| PBX1 | 0.463 | Upregulated | 3.35E-04 | 1.54E-02 |
| UBE2H | 0.463 | Upregulated | 6.85E-07 | 5.80E-04 |
| ASPRV1 | 0.465 | Upregulated | 1.10E-02 | 1.09E-01 |
| CMBL | 0.465 | Upregulated | 1.14E-02 | 1.11E-01 |
| RHD | 0.465 | Upregulated | 3.46E-02 | 2.06E-01 |
| BAGE5 | 0.468 | Upregulated | 1.40E-05 | 2.40E-03 |
| KIAA1033 | 0.468 | Upregulated | 2.20E-03 | 4.19E-02 |
| ZFP36L1 | 0.470 | Upregulated | 1.89E-03 | 3.86E-02 |
| GPR65 | 0.470 | Upregulated | 2.73E-05 | 3.59E-03 |
| PSG9 | 0.473 | Upregulated | 5.48E-03 | 7.27E-02 |
| WSB2 | 0.477 | Upregulated | 3.31E-03 | 5.33E-02 |
| RPL9 | 0.478 | Upregulated | 3.78E-03 | 5.81E-02 |
| CASP5 | 0.479 | Upregulated | 1.69E-02 | 1.38E-01 |
| BCL2L1 | 0.483 | Upregulated | 1.17E-02 | 1.12E-01 |
| PAK2 | 0.483 | Upregulated | 1.63E-02 | 1.35E-01 |
| GLRX5 | 0.487 | Upregulated | 3.68E-04 | 1.60E-02 |
| HMGB1L1 | 0.488 | Upregulated | 5.53E-04 | 1.98E-02 |
| PIK3AP1 | 0.491 | Upregulated | 6.38E-03 | 7.92E-02 |
| SLC25A39 | 0.491 | Upregulated | 1.70E-03 | 3.64E-02 |
| FGL2 | 0.492 | Upregulated | 5.80E-06 | 1.44E-03 |
| CSDA | 0.493 | Upregulated | 4.70E-05 | 4.97E-03 |
| GBP3 | 0.493 | Upregulated | 3.65E-04 | 1.60E-02 |
| HECA | 0.495 | Upregulated | 3.06E-04 | 1.46E-02 |
| SFRS11 | 0.495 | Upregulated | 2.42E-04 | 1.28E-02 |
| CAMP | 0.496 | Upregulated | 2.55E-02 | 1.74E-01 |
| KCNJ2 | 0.498 | Upregulated | 2.87E-03 | 4.92E-02 |
| CLEC7A | 0.498 | Upregulated | 1.05E-03 | 2.79E-02 |
| MGC13057 | 0.501 | Upregulated | 4.00E-03 | 6.01E-02 |

| | | | | |
|---|---|---|---|---|
| HCG27 | 0.505 | Upregulated | 1.29E-04 | 8.92E-03 |
| CYP4F3 | 0.507 | Upregulated | 7.28E-03 | 8.55E-02 |
| RBAK | 0.511 | Upregulated | 2.13E-03 | 4.09E-02 |
| GPR109B | 0.511 | Upregulated | 2.64E-04 | 1.36E-02 |
| NOV | 0.513 | Upregulated | 9.15E-04 | 2.60E-02 |
| SLC25A37 | 0.514 | Upregulated | 1.29E-03 | 3.11E-02 |
| RAD21 | 0.519 | Upregulated | 2.84E-07 | 3.65E-04 |
| UBXN6 | 0.519 | Upregulated | 1.34E-03 | 3.17E-02 |
| XK | 0.521 | Upregulated | 8.62E-03 | 9.48E-02 |
| GSTA5 | 0.521 | Upregulated | 6.36E-03 | 7.91E-02 |
| CD226 | 0.523 | Upregulated | 4.23E-05 | 4.82E-03 |
| CDC34 | 0.525 | Upregulated | 2.80E-04 | 1.39E-02 |
| HLA-G | 0.527 | Upregulated | 1.10E-04 | 8.09E-03 |
| SELPLG | 0.532 | Upregulated | 2.39E-04 | 1.28E-02 |
| CD46 | 0.533 | Upregulated | 3.02E-06 | 1.00E-03 |
| CAST | 0.535 | Upregulated | 5.60E-06 | 1.44E-03 |
| SNX10 | 0.536 | Upregulated | 4.93E-04 | 1.88E-02 |
| TTC25 | 0.544 | Upregulated | 4.48E-04 | 1.79E-02 |
| IL8 | 0.547 | Upregulated | 7.79E-05 | 6.76E-03 |
| MS4A3 | 0.548 | Upregulated | 3.19E-04 | 1.49E-02 |
| FAM83F | 0.549 | Upregulated | 1.38E-04 | 9.22E-03 |
| SPAST | 0.549 | Upregulated | 3.65E-04 | 1.60E-02 |
| ERGIC2 | 0.550 | Upregulated | 1.27E-03 | 3.09E-02 |
| TMEM56 | 0.550 | Upregulated | 3.27E-03 | 5.29E-02 |
| GIMAP2 | 0.552 | Upregulated | 2.95E-04 | 1.43E-02 |
| FAM44A | 0.554 | Upregulated | 2.38E-06 | 9.28E-04 |
| SOCS1 | 0.554 | Upregulated | 1.72E-03 | 3.66E-02 |
| GPR146 | 0.557 | Upregulated | 4.11E-04 | 1.72E-02 |
| TMCC2 | 0.560 | Upregulated | 6.75E-03 | 8.19E-02 |
| CD164 | 0.561 | Upregulated | 5.41E-04 | 1.96E-02 |
| GBP1 | 0.564 | Upregulated | 1.40E-03 | 3.24E-02 |
| CPA3 | 0.564 | Upregulated | 1.32E-04 | 8.98E-03 |
| HEMGN | 0.566 | Upregulated | 2.33E-02 | 1.65E-01 |
| SLC6A8 | 0.570 | Upregulated | 2.25E-03 | 4.24E-02 |
| RHAG | 0.570 | Upregulated | 2.26E-03 | 4.25E-02 |
| LHFPL2 | 0.573 | Upregulated | 1.24E-04 | 8.67E-03 |
| PIP5K2A | 0.576 | Upregulated | 8.19E-04 | 2.47E-02 |
| HBQ1 | 0.579 | Upregulated | 2.02E-04 | 1.14E-02 |
| RAB33B | 0.579 | Upregulated | 4.47E-03 | 6.43E-02 |
| FLJ20309 | 0.583 | Upregulated | 3.05E-04 | 1.46E-02 |
| Septin 7 | 0.585 | Upregulated | 3.61E-04 | 1.59E-02 |
| FECH | 0.585 | Upregulated | 1.28E-03 | 3.10E-02 |
| SPRYD3 | 0.589 | Upregulated | 3.13E-05 | 3.86E-03 |
| CEACAM6 | 0.594 | Upregulated | 7.63E-03 | 8.83E-02 |
| VWCE | 0.595 | Upregulated | 1.39E-02 | 1.23E-01 |
| FLJ20273 | 0.611 | Upregulated | 3.88E-04 | 1.66E-02 |
| PHOSPHO1 | 0.619 | Upregulated | 3.23E-04 | 1.50E-02 |

| | | | | |
|---|---|---|---|---|
| ALDH1A1 | 0.621 | Upregulated | 8.52E-04 | 2.52E-02 |
| C1GALT1 | 0.624 | Upregulated | 7.10E-06 | 1.60E-03 |
| HAGH | 0.630 | Upregulated | 3.16E-04 | 1.49E-02 |
| MICALCL | 0.630 | Upregulated | 3.85E-05 | 4.58E-03 |
| P2RY13 | 0.634 | Upregulated | 1.10E-04 | 8.09E-03 |
| RBM38 | 0.637 | Upregulated | 3.54E-05 | 4.24E-03 |
| DEFA1 | 0.639 | Upregulated | 4.38E-03 | 6.36E-02 |
| CCDC52 | 0.639 | Upregulated | 6.12E-06 | 1.45E-03 |
| RPL23 | 0.642 | Upregulated | 2.01E-03 | 3.98E-02 |
| CD274 | 0.643 | Upregulated | 1.94E-03 | 3.91E-02 |
| ADIPOR1 | 0.644 | Upregulated | 7.92E-05 | 6.82E-03 |
| MXI1 | 0.644 | Upregulated | 7.11E-05 | 6.44E-03 |
| FBXO7 | 0.647 | Upregulated | 1.16E-03 | 2.93E-02 |
| APOBEC3A | 0.653 | Upregulated | 1.06E-04 | 7.89E-03 |
| CLK1 | 0.653 | Upregulated | 1.95E-05 | 2.96E-03 |
| DEFA4 | 0.655 | Upregulated | 1.54E-02 | 1.31E-01 |
| GPR109A | 0.658 | Upregulated | 1.06E-04 | 7.90E-03 |
| SIAH2 | 0.662 | Upregulated | 9.77E-05 | 7.62E-03 |
| GMPR | 0.667 | Upregulated | 4.01E-03 | 6.02E-02 |
| PDZK1IP1 | 0.674 | Upregulated | 2.77E-03 | 4.81E-02 |
| ANKRD22 | 0.677 | Upregulated | 5.00E-03 | 6.88E-02 |
| CEACAM8 | 0.679 | Upregulated | 6.10E-03 | 7.74E-02 |
| BNIP3L | 0.680 | Upregulated | 1.55E-05 | 2.60E-03 |
| EPB49 | 0.683 | Upregulated | 8.79E-04 | 2.55E-02 |
| F2RL1 | 0.684 | Upregulated | 5.98E-06 | 1.45E-03 |
| LGALS3 | 0.686 | Upregulated | 4.33E-05 | 4.84E-03 |
| PTMA | 0.688 | Upregulated | 1.92E-03 | 3.89E-02 |
| CHPT1 | 0.696 | Upregulated | 2.30E-05 | 3.23E-03 |
| TSPAN5 | 0.700 | Upregulated | 2.79E-04 | 1.39E-02 |
| INDO | 0.701 | Upregulated | 7.64E-03 | 8.83E-02 |
| WDR40A | 0.722 | Upregulated | 1.01E-04 | 7.71E-03 |
| BATF2 | 0.726 | Upregulated | 5.91E-04 | 2.07E-02 |
| IDO1 | 0.732 | Upregulated | 4.91E-03 | 6.81E-02 |
| TUBB2A | 0.735 | Upregulated | 2.06E-03 | 4.04E-02 |
| FCGR1B | 0.735 | Upregulated | 4.39E-04 | 1.78E-02 |
| OSBP2 | 0.736 | Upregulated | 4.94E-03 | 6.83E-02 |
| GBP6 | 0.738 | Upregulated | 9.46E-06 | 1.87E-03 |
| PTGS2 | 0.739 | Upregulated | 6.20E-06 | 1.46E-03 |
| TLR1 | 0.747 | Upregulated | 2.47E-04 | 1.29E-02 |
| PLEK2 | 0.752 | Upregulated | 1.08E-04 | 8.01E-03 |
| RNF213 | 0.754 | Upregulated | 4.49E-07 | 4.21E-04 |
| DEFA3 | 0.755 | Upregulated | 4.63E-03 | 6.57E-02 |
| P2RY14 | 0.769 | Upregulated | 2.93E-06 | 1.00E-03 |
| FCER1A | 0.774 | Upregulated | 2.07E-03 | 4.05E-02 |
| HBD | 0.785 | Upregulated | 1.72E-03 | 3.67E-02 |
| AHSP | 0.787 | Upregulated | 5.02E-03 | 6.90E-02 |
| HDC | 0.797 | Upregulated | 5.21E-05 | 5.21E-03 |

| | | | | |
|---|---|---|---|---|
| STRADB | 0.803 | Upregulated | 1.47E-04 | 9.52E-03 |
| CLC | 0.809 | Upregulated | 1.30E-03 | 3.11E-02 |
| FCGR1A | 0.815 | Upregulated | 2.92E-04 | 1.43E-02 |
| TNS1 | 0.816 | Upregulated | 5.66E-04 | 2.02E-02 |
| GPR175 | 0.820 | Upregulated | 4.84E-05 | 5.07E-03 |
| SIGLEC14 | 0.823 | Upregulated | 4.35E-02 | 2.34E-01 |
| DEFA1B | 0.824 | Upregulated | 3.74E-03 | 5.77E-02 |
| IGF2BP2 | 0.828 | Upregulated | 8.15E-06 | 1.72E-03 |
| SNCA | 0.842 | Upregulated | 2.39E-03 | 4.41E-02 |
| RNF182 | 0.849 | Upregulated | 7.12E-04 | 2.29E-02 |
| CA1 | 0.850 | Upregulated | 1.87E-02 | 1.45E-01 |
| FAM26F | 0.854 | Upregulated | 3.56E-07 | 3.87E-04 |
| TMOD1 | 0.854 | Upregulated | 4.44E-04 | 1.79E-02 |
| GBP5 | 0.859 | Upregulated | 1.64E-06 | 7.58E-04 |
| KRT1 | 0.863 | Upregulated | 1.53E-02 | 1.30E-01 |
| TRIM58 | 0.873 | Upregulated | 1.63E-04 | 1.02E-02 |
| EPB42 | 0.899 | Upregulated | 1.37E-03 | 3.21E-02 |
| FCGR1C | 0.899 | Upregulated | 2.06E-05 | 3.04E-03 |
| GYPB | 0.901 | Upregulated | 5.00E-03 | 6.88E-02 |
| BLVRB | 0.922 | Upregulated | 6.23E-07 | 5.69E-04 |
| DPYSL5 | 0.923 | Upregulated | 4.20E-05 | 4.82E-03 |
| SESN3 | 0.930 | Upregulated | 1.96E-05 | 2.96E-03 |
| SLC4A1 | 0.965 | Upregulated | 2.70E-04 | 1.37E-02 |
| SELENBP1 | 0.975 | Upregulated | 2.37E-03 | 4.39E-02 |
| IFIT1L | 0.982 | Upregulated | 4.21E-03 | 6.22E-02 |
| MBNL3 | 1.005 | Upregulated | 1.37E-08 | 7.92E-05 |
| HBE1 | 1.132 | Upregulated | 9.20E-07 | 5.88E-04 |
| SERPINA13 | 1.225 | Upregulated | 6.01E-06 | 1.45E-03 |
| ALAS2 | 1.283 | Upregulated | 1.32E-05 | 2.34E-03 |

**Supplementary Table S3f. Differentially expressed genes_Malawi_Female**

| Gene | logFC | Direction of expression | P.Value | adj.P.Val |
|---|---|---|---|---|
| OLIG1 | -0.534 | Downregulated | 5.47E-03 | 3.74E-01 |
| CCL23 | -0.525 | Downregulated | 3.39E-02 | 4.66E-01 |
| CACNA2D3 | -0.516 | Downregulated | 2.17E-02 | 4.40E-01 |
| HRK | -0.508 | Downregulated | 5.69E-03 | 3.74E-01 |
| LRRN3 | -0.502 | Downregulated | 3.10E-02 | 4.63E-01 |
| ZNF683 | -0.479 | Downregulated | 9.45E-03 | 3.91E-01 |
| GPATCH4 | -0.475 | Downregulated | 6.44E-05 | 2.01E-01 |
| FCRL6 | -0.437 | Downregulated | 7.53E-03 | 3.74E-01 |
| TMEM16C | -0.435 | Downregulated | 6.64E-03 | 3.74E-01 |
| CDKN1C | -0.434 | Downregulated | 1.90E-02 | 4.30E-01 |
| CCDC83 | -0.432 | Downregulated | 7.75E-03 | 3.75E-01 |
| CCL2 | -0.414 | Downregulated | 4.79E-02 | 5.02E-01 |
| BUB1 | -0.411 | Downregulated | 2.66E-02 | 4.55E-01 |
| PARM1 | -0.408 | Downregulated | 3.54E-03 | 3.74E-01 |
| PDIA4 | -0.405 | Downregulated | 5.65E-04 | 3.20E-01 |
| SH2D1B | -0.402 | Downregulated | 1.46E-03 | 3.33E-01 |
| HPGD | -0.395 | Downregulated | 2.71E-02 | 4.55E-01 |
| SPIN4 | -0.391 | Downregulated | 1.16E-03 | 3.30E-01 |
| RRAS2 | -0.389 | Downregulated | 1.51E-04 | 2.70E-01 |
| TCL1A | -0.388 | Downregulated | 3.34E-02 | 4.64E-01 |
| FAM179B | -0.387 | Downregulated | 2.08E-02 | 4.38E-01 |
| IL28RA | -0.385 | Downregulated | 2.12E-03 | 3.60E-01 |
| SLC39A10 | -0.384 | Downregulated | 2.64E-03 | 3.71E-01 |
| FCRL3 | -0.378 | Downregulated | 1.02E-02 | 3.97E-01 |
| RASGRP1 | -0.375 | Downregulated | 2.64E-03 | 3.71E-01 |
| HERC2 | -0.365 | Downregulated | 2.22E-03 | 3.60E-01 |
| GPR114 | -0.364 | Downregulated | 6.37E-03 | 3.74E-01 |
| MBNL2 | -0.360 | Downregulated | 1.35E-02 | 4.03E-01 |
| KIR2DL1 | -0.359 | Downregulated | 2.35E-02 | 4.46E-01 |
| TSPAN13 | -0.358 | Downregulated | 3.52E-02 | 4.70E-01 |
| HSPH1 | -0.358 | Downregulated | 1.07E-02 | 3.97E-01 |
| SPON2 | -0.357 | Downregulated | 3.29E-02 | 4.64E-01 |
| BIK | -0.355 | Downregulated | 1.60E-02 | 4.19E-01 |
| QPRT | -0.354 | Downregulated | 4.48E-03 | 3.74E-01 |
| SPIB | -0.354 | Downregulated | 4.39E-02 | 4.93E-01 |
| GMNN | -0.353 | Downregulated | 2.45E-02 | 4.51E-01 |
| SPAST | -0.352 | Downregulated | 3.64E-02 | 4.75E-01 |
| CCDC34 | -0.350 | Downregulated | 5.74E-03 | 3.74E-01 |
| COX11 | -0.349 | Downregulated | 5.01E-03 | 3.74E-01 |
| PYHIN1 | -0.345 | Downregulated | 2.22E-02 | 4.41E-01 |
| SF3A3 | -0.345 | Downregulated | 2.20E-02 | 4.41E-01 |
| FAM179A | -0.344 | Downregulated | 1.89E-02 | 4.30E-01 |
| S1PR1 | -0.344 | Downregulated | 1.14E-02 | 3.97E-01 |

| | | | | |
|---|---|---|---|---|
| OSBPL10 | -0.343 | Downregulated | 2.58E-02 | 4.52E-01 |
| RPS6KA2 | -0.342 | Downregulated | 5.55E-03 | 3.74E-01 |
| HRASLS2 | -0.340 | Downregulated | 1.98E-02 | 4.32E-01 |
| FAM167A | -0.337 | Downregulated | 2.11E-02 | 4.40E-01 |
| FASLG | -0.335 | Downregulated | 4.92E-02 | 5.05E-01 |
| PASK | -0.333 | Downregulated | 1.30E-02 | 4.03E-01 |
| CCR9 | -0.331 | Downregulated | 1.96E-02 | 4.32E-01 |
| XYLT1 | -0.330 | Downregulated | 2.33E-03 | 3.60E-01 |
| SIGLEC8 | -0.330 | Downregulated | 1.61E-02 | 4.20E-01 |
| ZNF708 | -0.328 | Downregulated | 5.33E-03 | 3.74E-01 |
| STRBP | -0.321 | Downregulated | 5.58E-03 | 3.74E-01 |
| UAP1 | -0.321 | Downregulated | 1.27E-02 | 4.02E-01 |
| CLDND2 | -0.321 | Downregulated | 4.15E-02 | 4.88E-01 |
| ECT2 | -0.316 | Downregulated | 3.70E-03 | 3.74E-01 |
| ADCY9 | -0.316 | Downregulated | 8.41E-03 | 3.82E-01 |
| ITGB1BP1 | -0.315 | Downregulated | 1.73E-04 | 2.72E-01 |
| HSPA4 | -0.312 | Downregulated | 1.70E-02 | 4.24E-01 |
| CLIC3 | -0.311 | Downregulated | 3.09E-02 | 4.63E-01 |
| HSZFP36 | -0.311 | Downregulated | 1.50E-02 | 4.14E-01 |
| ABHD15 | -0.310 | Downregulated | 1.69E-03 | 3.41E-01 |
| SDF2L1 | -0.310 | Downregulated | 3.52E-04 | 3.13E-01 |
| CHURC1 | -0.309 | Downregulated | 4.13E-02 | 4.88E-01 |
| PTPRCAP | -0.309 | Downregulated | 3.51E-03 | 3.74E-01 |
| SIDT1 | -0.309 | Downregulated | 1.00E-03 | 3.30E-01 |
| MEX3C | -0.308 | Downregulated | 1.42E-02 | 4.06E-01 |
| TMEM14A | -0.308 | Downregulated | 8.55E-03 | 3.82E-01 |
| E2F5 | -0.303 | Downregulated | 1.13E-02 | 3.97E-01 |
| RAB30 | -0.300 | Downregulated | 5.81E-03 | 3.74E-01 |
| PMS1 | -0.300 | Downregulated | 4.36E-02 | 4.93E-01 |
| RANGRF | -0.299 | Downregulated | 3.87E-02 | 4.83E-01 |
| SP4 | -0.297 | Downregulated | 5.16E-03 | 3.74E-01 |
| PLB1 | -0.296 | Downregulated | 3.94E-02 | 4.85E-01 |
| SLC25A29 | -0.295 | Downregulated | 1.62E-02 | 4.20E-01 |
| ST6GAL1 | -0.295 | Downregulated | 7.20E-03 | 3.74E-01 |
| DNAJC25 | -0.294 | Downregulated | 1.71E-02 | 4.24E-01 |
| UBE1DC1 | -0.294 | Downregulated | 3.53E-03 | 3.74E-01 |
| EIF2AK3 | -0.293 | Downregulated | 5.17E-03 | 3.74E-01 |
| TMEM130 | -0.293 | Downregulated | 1.66E-03 | 3.41E-01 |
| MRPS6 | -0.293 | Downregulated | 5.85E-05 | 2.01E-01 |
| PTGDS | -0.293 | Downregulated | 4.58E-02 | 4.98E-01 |
| FKSG44 | -0.291 | Downregulated | 4.34E-02 | 4.93E-01 |
| TRAM2 | -0.290 | Downregulated | 1.38E-02 | 4.05E-01 |
| TRIM32 | -0.290 | Downregulated | 6.24E-04 | 3.20E-01 |
| ISOC1 | -0.290 | Downregulated | 4.33E-04 | 3.20E-01 |
| IRF4 | -0.290 | Downregulated | 1.08E-02 | 3.97E-01 |
| FANCF | -0.289 | Downregulated | 2.60E-03 | 3.71E-01 |
| Septin 11 | -0.289 | Downregulated | 1.19E-02 | 3.97E-01 |

| | | | | |
|---|---|---|---|---|
| SFRS13A | -0.287 | Downregulated | 9.67E-03 | 3.92E-01 |
| RDH10 | -0.286 | Downregulated | 4.30E-03 | 3.74E-01 |
| FAM83D | -0.285 | Downregulated | 2.32E-03 | 3.60E-01 |
| EPHA4 | -0.285 | Downregulated | 1.07E-02 | 3.97E-01 |
| CDCA4 | -0.284 | Downregulated | 2.59E-03 | 3.71E-01 |
| FAM98A | -0.283 | Downregulated | 3.30E-02 | 4.64E-01 |
| MAK16 | -0.283 | Downregulated | 3.81E-02 | 4.81E-01 |
| MESDC1 | -0.282 | Downregulated | 7.90E-03 | 3.76E-01 |
| AVEN | -0.282 | Downregulated | 1.15E-02 | 3.97E-01 |
| BUB3 | -0.281 | Downregulated | 1.34E-02 | 4.03E-01 |
| CELSR3 | -0.280 | Downregulated | 2.53E-02 | 4.52E-01 |
| MRPL1 | -0.278 | Downregulated | 3.79E-02 | 4.80E-01 |
| HMMR | -0.278 | Downregulated | 4.30E-02 | 4.92E-01 |
| CX3CR1 | -0.278 | Downregulated | 3.73E-02 | 4.78E-01 |
| DR1 | -0.276 | Downregulated | 2.95E-03 | 3.74E-01 |
| CD79B | -0.276 | Downregulated | 4.07E-02 | 4.87E-01 |
| OR7D2 | -0.276 | Downregulated | 2.19E-02 | 4.40E-01 |
| PM20D2 | -0.275 | Downregulated | 1.08E-02 | 3.97E-01 |
| GINS3 | -0.275 | Downregulated | 8.30E-03 | 3.80E-01 |
| XBP1 | -0.274 | Downregulated | 1.14E-03 | 3.30E-01 |
| SLC29A1 | -0.274 | Downregulated | 2.50E-02 | 4.52E-01 |
| CLCF1 | -0.273 | Downregulated | 3.02E-03 | 3.74E-01 |
| CRIP1 | -0.273 | Downregulated | 1.30E-03 | 3.30E-01 |
| GRAMD4 | -0.273 | Downregulated | 2.82E-03 | 3.74E-01 |
| ZNF649 | -0.272 | Downregulated | 1.02E-03 | 3.30E-01 |
| LIMA1 | -0.272 | Downregulated | 3.18E-02 | 4.63E-01 |
| TOX | -0.272 | Downregulated | 4.30E-02 | 4.92E-01 |
| CR2 | -0.271 | Downregulated | 2.78E-02 | 4.55E-01 |
| MYBL1 | -0.271 | Downregulated | 4.58E-02 | 4.98E-01 |
| IL2RB | -0.270 | Downregulated | 4.03E-02 | 4.87E-01 |
| RAB15 | -0.268 | Downregulated | 1.92E-02 | 4.32E-01 |
| UST | -0.268 | Downregulated | 1.26E-02 | 4.02E-01 |
| PGA5 | -0.267 | Downregulated | 2.70E-02 | 4.55E-01 |
| AHI1 | -0.266 | Downregulated | 3.49E-02 | 4.69E-01 |
| NCALD | -0.266 | Downregulated | 2.99E-02 | 4.60E-01 |
| LAX1 | -0.266 | Downregulated | 1.70E-02 | 4.24E-01 |
| LCLAT1 | -0.265 | Downregulated | 9.73E-03 | 3.92E-01 |
| CCR6 | -0.265 | Downregulated | 4.04E-02 | 4.87E-01 |
| RAB17 | 0.266 | Upregulated | 1.72E-03 | 3.43E-01 |
| NGFRAP1 | 0.266 | Upregulated | 1.86E-02 | 4.28E-01 |
| YOD1 | 0.267 | Upregulated | 1.12E-02 | 3.97E-01 |
| EIF2AK1 | 0.267 | Upregulated | 1.08E-02 | 3.97E-01 |
| FLCN | 0.267 | Upregulated | 4.08E-03 | 3.74E-01 |
| CCRL2 | 0.268 | Upregulated | 2.11E-02 | 4.40E-01 |
| DCST2 | 0.268 | Upregulated | 4.55E-03 | 3.74E-01 |
| CCDC23 | 0.269 | Upregulated | 2.00E-03 | 3.60E-01 |
| MAP1A | 0.269 | Upregulated | 4.52E-02 | 4.98E-01 |

| | | | | |
|---|---|---|---|---|
| MAP1S | 0.271 | Upregulated | 3.54E-02 | 4.71E-01 |
| VAMP5 | 0.271 | Upregulated | 1.92E-02 | 4.32E-01 |
| LYL1 | 0.272 | Upregulated | 3.38E-02 | 4.66E-01 |
| CITED4 | 0.273 | Upregulated | 2.98E-02 | 4.59E-01 |
| TPM1 | 0.273 | Upregulated | 1.78E-03 | 3.50E-01 |
| TSC22D3 | 0.274 | Upregulated | 3.62E-02 | 4.75E-01 |
| GADD45G | 0.276 | Upregulated | 2.58E-02 | 4.52E-01 |
| NAPA | 0.277 | Upregulated | 1.08E-02 | 3.97E-01 |
| FLJ41603 | 0.278 | Upregulated | 7.31E-03 | 3.74E-01 |
| ATG9A | 0.279 | Upregulated | 4.69E-03 | 3.74E-01 |
| CAMK2A | 0.279 | Upregulated | 1.70E-02 | 4.24E-01 |
| FAM117A | 0.280 | Upregulated | 2.24E-02 | 4.41E-01 |
| PA2G4 | 0.280 | Upregulated | 9.56E-03 | 3.92E-01 |
| TYMP | 0.280 | Upregulated | 1.92E-02 | 4.32E-01 |
| TFDP1 | 0.281 | Upregulated | 2.50E-02 | 4.52E-01 |
| STK11 | 0.281 | Upregulated | 4.72E-03 | 3.74E-01 |
| SCO2 | 0.282 | Upregulated | 2.12E-02 | 4.40E-01 |
| NDST1 | 0.282 | Upregulated | 2.47E-03 | 3.65E-01 |
| RANBP10 | 0.282 | Upregulated | 4.10E-02 | 4.87E-01 |
| CD151 | 0.284 | Upregulated | 1.14E-02 | 3.97E-01 |
| OR2T8 | 0.284 | Upregulated | 1.09E-02 | 3.97E-01 |
| HSN2 | 0.287 | Upregulated | 3.46E-03 | 3.74E-01 |
| HIST3H2A | 0.288 | Upregulated | 1.83E-02 | 4.28E-01 |
| GDE1 | 0.289 | Upregulated | 1.14E-02 | 3.97E-01 |
| SLC43A1 | 0.289 | Upregulated | 3.39E-03 | 3.74E-01 |
| GDF2 | 0.291 | Upregulated | 4.83E-03 | 3.74E-01 |
| CTSA | 0.291 | Upregulated | 1.58E-02 | 4.19E-01 |
| ITGB5 | 0.292 | Upregulated | 1.98E-02 | 4.32E-01 |
| EMID1 | 0.292 | Upregulated | 6.00E-03 | 3.74E-01 |
| MFSD2B | 0.294 | Upregulated | 3.56E-02 | 4.71E-01 |
| MFHAS1 | 0.294 | Upregulated | 2.09E-02 | 4.38E-01 |
| OLAH | 0.295 | Upregulated | 1.16E-02 | 3.97E-01 |
| GSPT1 | 0.297 | Upregulated | 4.91E-02 | 5.05E-01 |
| SDSL | 0.299 | Upregulated | 1.53E-02 | 4.16E-01 |
| CATSPER1 | 0.299 | Upregulated | 2.46E-02 | 4.51E-01 |
| PDCD1LG2 | 0.300 | Upregulated | 4.04E-02 | 4.87E-01 |
| HIST1H3H | 0.301 | Upregulated | 4.85E-02 | 5.03E-01 |
| ENDOD1 | 0.302 | Upregulated | 4.03E-03 | 3.74E-01 |
| PRKCDBP | 0.302 | Upregulated | 5.25E-03 | 3.74E-01 |
| AP2A1 | 0.302 | Upregulated | 2.45E-02 | 4.51E-01 |
| LY6G6F | 0.305 | Upregulated | 4.61E-02 | 4.99E-01 |
| GCGR | 0.306 | Upregulated | 3.29E-02 | 4.64E-01 |
| NME4 | 0.307 | Upregulated | 1.98E-02 | 4.32E-01 |
| DAP | 0.307 | Upregulated | 3.61E-03 | 3.74E-01 |
| SYTL4 | 0.308 | Upregulated | 1.90E-02 | 4.30E-01 |
| PPP2R5B | 0.311 | Upregulated | 3.53E-02 | 4.71E-01 |
| PTPRN | 0.312 | Upregulated | 1.28E-03 | 3.30E-01 |

| | | | | |
|---|---|---|---|---|
| MSI2 | 0.313 | Upregulated | 1.15E-02 | 3.97E-01 |
| IGFBP1 | 0.316 | Upregulated | 3.92E-03 | 3.74E-01 |
| P2RY13 | 0.316 | Upregulated | 4.22E-02 | 4.91E-01 |
| GBP4 | 0.317 | Upregulated | 2.93E-02 | 4.58E-01 |
| RMND5A | 0.318 | Upregulated | 2.36E-02 | 4.46E-01 |
| RAD23A | 0.319 | Upregulated | 2.98E-02 | 4.60E-01 |
| TMEM111 | 0.320 | Upregulated | 2.52E-02 | 4.52E-01 |
| GM2A | 0.321 | Upregulated | 1.80E-02 | 4.28E-01 |
| OR51S1 | 0.321 | Upregulated | 2.93E-04 | 2.99E-01 |
| CARD17 | 0.321 | Upregulated | 4.98E-02 | 5.06E-01 |
| RHCE | 0.322 | Upregulated | 2.75E-02 | 4.55E-01 |
| FRMD3 | 0.323 | Upregulated | 6.97E-03 | 3.74E-01 |
| SLC25A39 | 0.324 | Upregulated | 3.05E-02 | 4.63E-01 |
| OBFC1 | 0.324 | Upregulated | 6.72E-05 | 2.01E-01 |
| PNPLA2 | 0.325 | Upregulated | 4.09E-02 | 4.87E-01 |
| TFDP2 | 0.326 | Upregulated | 3.40E-02 | 4.66E-01 |
| RGS10 | 0.326 | Upregulated | 7.34E-03 | 3.74E-01 |
| AIF1 | 0.327 | Upregulated | 1.25E-02 | 4.02E-01 |
| RILP | 0.328 | Upregulated | 2.38E-02 | 4.48E-01 |
| TSPY3 | 0.330 | Upregulated | 1.30E-04 | 2.52E-01 |
| RRAGD | 0.331 | Upregulated | 1.10E-02 | 3.97E-01 |
| PHLDB1 | 0.332 | Upregulated | 4.72E-02 | 5.01E-01 |
| TRAK2 | 0.334 | Upregulated | 1.55E-02 | 4.16E-01 |
| RNF123 | 0.336 | Upregulated | 1.22E-02 | 3.99E-01 |
| CFB | 0.336 | Upregulated | 1.28E-06 | 4.41E-02 |
| CFH | 0.336 | Upregulated | 6.92E-05 | 2.01E-01 |
| SORT1 | 0.337 | Upregulated | 2.25E-02 | 4.41E-01 |
| HIST1H2BJ | 0.337 | Upregulated | 1.35E-02 | 4.03E-01 |
| AMFR | 0.339 | Upregulated | 2.79E-04 | 2.99E-01 |
| ZRANB1 | 0.339 | Upregulated | 2.35E-02 | 4.46E-01 |
| 08-Mar | 0.340 | Upregulated | 1.27E-02 | 4.02E-01 |
| DHRS9 | 0.342 | Upregulated | 2.71E-03 | 3.74E-01 |
| FAH | 0.344 | Upregulated | 3.43E-03 | 3.74E-01 |
| KCNJ10 | 0.346 | Upregulated | 1.36E-02 | 4.03E-01 |
| RTF1 | 0.347 | Upregulated | 2.51E-02 | 4.52E-01 |
| TMEM63B | 0.347 | Upregulated | 5.62E-03 | 3.74E-01 |
| VSIG4 | 0.348 | Upregulated | 4.37E-03 | 3.74E-01 |
| CTNNAL1 | 0.348 | Upregulated | 3.88E-02 | 4.83E-01 |
| CTDSPL | 0.348 | Upregulated | 4.14E-02 | 4.88E-01 |
| BZRPL1 | 0.348 | Upregulated | 2.98E-03 | 3.74E-01 |
| GCAT | 0.351 | Upregulated | 1.66E-02 | 4.23E-01 |
| PINK1 | 0.352 | Upregulated | 2.88E-02 | 4.56E-01 |
| GNA12 | 0.354 | Upregulated | 2.70E-02 | 4.55E-01 |
| RFESD | 0.357 | Upregulated | 2.00E-02 | 4.33E-01 |
| TNFAIP2 | 0.360 | Upregulated | 9.03E-03 | 3.90E-01 |
| SELP | 0.363 | Upregulated | 1.27E-02 | 4.02E-01 |
| ASCC2 | 0.363 | Upregulated | 2.87E-02 | 4.56E-01 |

| | | | | |
|---|---|---|---|---|
| ANK1 | 0.363 | Upregulated | 9.02E-03 | 3.90E-01 |
| MICAL2 | 0.363 | Upregulated | 3.18E-02 | 4.63E-01 |
| HOXC10 | 0.364 | Upregulated | 1.04E-02 | 3.97E-01 |
| H1F0 | 0.364 | Upregulated | 2.22E-02 | 4.41E-01 |
| FKBP8 | 0.367 | Upregulated | 4.04E-02 | 4.87E-01 |
| SH3BGRL2 | 0.368 | Upregulated | 3.95E-02 | 4.85E-01 |
| FOXO3 | 0.370 | Upregulated | 1.63E-03 | 3.41E-01 |
| SLC2A1 | 0.370 | Upregulated | 1.39E-02 | 4.05E-01 |
| PTPLA | 0.370 | Upregulated | 1.35E-02 | 4.03E-01 |
| HSD3B7 | 0.372 | Upregulated | 8.21E-03 | 3.80E-01 |
| TBC1D22B | 0.372 | Upregulated | 1.24E-02 | 4.02E-01 |
| P2RX1 | 0.374 | Upregulated | 3.24E-03 | 3.74E-01 |
| UBE2H | 0.374 | Upregulated | 1.33E-02 | 4.03E-01 |
| NT5M | 0.375 | Upregulated | 2.59E-03 | 3.71E-01 |
| SLC1A5 | 0.378 | Upregulated | 1.14E-02 | 3.97E-01 |
| GYPC | 0.378 | Upregulated | 3.46E-02 | 4.69E-01 |
| ADIPOR1 | 0.382 | Upregulated | 4.17E-02 | 4.89E-01 |
| ABCA13 | 0.384 | Upregulated | 6.02E-03 | 3.74E-01 |
| GPR146 | 0.384 | Upregulated | 3.73E-02 | 4.78E-01 |
| DPM2 | 0.385 | Upregulated | 3.24E-02 | 4.63E-01 |
| SLC14A1 | 0.387 | Upregulated | 5.40E-03 | 3.74E-01 |
| LHFPL2 | 0.390 | Upregulated | 2.73E-03 | 3.74E-01 |
| PBX1 | 0.390 | Upregulated | 1.88E-02 | 4.29E-01 |
| UBE2O | 0.390 | Upregulated | 4.79E-02 | 5.02E-01 |
| SOD2 | 0.391 | Upregulated | 2.31E-02 | 4.45E-01 |
| ATP5E | 0.392 | Upregulated | 1.50E-02 | 4.14E-01 |
| GFOD2 | 0.393 | Upregulated | 3.24E-03 | 3.74E-01 |
| CDC34 | 0.394 | Upregulated | 2.81E-02 | 4.55E-01 |
| TGM2 | 0.394 | Upregulated | 4.71E-02 | 5.01E-01 |
| TBCEL | 0.395 | Upregulated | 3.21E-02 | 4.63E-01 |
| HAGH | 0.396 | Upregulated | 3.96E-02 | 4.85E-01 |
| HDGF | 0.396 | Upregulated | 6.52E-03 | 3.74E-01 |
| IGSF6 | 0.396 | Upregulated | 6.45E-03 | 3.74E-01 |
| ERAP2 | 0.396 | Upregulated | 4.57E-02 | 4.98E-01 |
| FHDC1 | 0.397 | Upregulated | 7.66E-03 | 3.74E-01 |
| HIST1H2BG | 0.397 | Upregulated | 8.72E-03 | 3.85E-01 |
| NDUFAF3 | 0.399 | Upregulated | 2.69E-03 | 3.74E-01 |
| MS4A3 | 0.401 | Upregulated | 2.09E-03 | 3.60E-01 |
| KANK2 | 0.402 | Upregulated | 7.25E-03 | 3.74E-01 |
| GLRX5 | 0.403 | Upregulated | 6.61E-03 | 3.74E-01 |
| TDRD9 | 0.403 | Upregulated | 4.18E-02 | 4.89E-01 |
| MICALCL | 0.405 | Upregulated | 2.41E-02 | 4.50E-01 |
| ARHGEF12 | 0.406 | Upregulated | 1.14E-02 | 3.97E-01 |
| GATA1 | 0.408 | Upregulated | 3.86E-02 | 4.82E-01 |
| HIST3H2BB | 0.410 | Upregulated | 6.51E-03 | 3.74E-01 |
| TRIM10 | 0.411 | Upregulated | 1.71E-02 | 4.24E-01 |
| KIFC3 | 0.411 | Upregulated | 8.45E-04 | 3.30E-01 |

| | | | | |
|---|---|---|---|---|
| CISD2 | 0.413 | Upregulated | 3.66E-02 | 4.76E-01 |
| CLEC12A | 0.413 | Upregulated | 2.69E-02 | 4.55E-01 |
| SLC38A5 | 0.415 | Upregulated | 2.17E-03 | 3.60E-01 |
| GBP1 | 0.417 | Upregulated | 2.06E-02 | 4.36E-01 |
| FAM104A | 0.419 | Upregulated | 4.98E-03 | 3.74E-01 |
| CHPT1 | 0.419 | Upregulated | 2.92E-02 | 4.58E-01 |
| WDR40A | 0.420 | Upregulated | 2.06E-02 | 4.36E-01 |
| BNIP3L | 0.420 | Upregulated | 1.85E-02 | 4.28E-01 |
| BEND7 | 0.421 | Upregulated | 1.31E-04 | 2.52E-01 |
| WARS | 0.421 | Upregulated | 2.31E-03 | 3.60E-01 |
| SLC6A12 | 0.422 | Upregulated | 2.27E-03 | 3.60E-01 |
| FLJ20489 | 0.423 | Upregulated | 1.11E-02 | 3.97E-01 |
| GBP6 | 0.423 | Upregulated | 1.45E-03 | 3.32E-01 |
| CD36 | 0.425 | Upregulated | 2.66E-02 | 4.55E-01 |
| WNK1 | 0.432 | Upregulated | 5.89E-03 | 3.74E-01 |
| SRRD | 0.432 | Upregulated | 1.47E-02 | 4.13E-01 |
| HEPACAM2 | 0.433 | Upregulated | 2.78E-02 | 4.55E-01 |
| RIOK3 | 0.435 | Upregulated | 1.52E-02 | 4.16E-01 |
| CCDC52 | 0.438 | Upregulated | 7.44E-03 | 3.74E-01 |
| MAP2K3 | 0.439 | Upregulated | 7.17E-03 | 3.74E-01 |
| CMTM5 | 0.446 | Upregulated | 4.81E-03 | 3.74E-01 |
| UBXN6 | 0.447 | Upregulated | 3.52E-02 | 4.70E-01 |
| TMEM86B | 0.450 | Upregulated | 2.13E-02 | 4.40E-01 |
| HIST1H4H | 0.451 | Upregulated | 1.28E-02 | 4.02E-01 |
| SAMD14 | 0.451 | Upregulated | 4.30E-03 | 3.74E-01 |
| PTMS | 0.452 | Upregulated | 4.18E-03 | 3.74E-01 |
| SLC7A5 | 0.453 | Upregulated | 2.26E-02 | 4.42E-01 |
| MMRN1 | 0.456 | Upregulated | 1.25E-02 | 4.02E-01 |
| DYRK3 | 0.457 | Upregulated | 2.91E-04 | 2.99E-01 |
| PPBP | 0.458 | Upregulated | 1.48E-02 | 4.14E-01 |
| PIP5K2A | 0.458 | Upregulated | 4.95E-02 | 5.06E-01 |
| TCP11L2 | 0.462 | Upregulated | 6.99E-03 | 3.74E-01 |
| MYOF | 0.462 | Upregulated | 6.88E-03 | 3.74E-01 |
| CA2 | 0.472 | Upregulated | 3.54E-02 | 4.71E-01 |
| STRADB | 0.473 | Upregulated | 3.37E-02 | 4.65E-01 |
| CYP4F3 | 0.473 | Upregulated | 1.74E-02 | 4.26E-01 |
| FECH | 0.475 | Upregulated | 1.97E-02 | 4.32E-01 |
| FIS1 | 0.475 | Upregulated | 3.49E-03 | 3.74E-01 |
| TREML1 | 0.475 | Upregulated | 1.21E-02 | 3.99E-01 |
| C1QA | 0.479 | Upregulated | 8.78E-04 | 3.30E-01 |
| TPST1 | 0.480 | Upregulated | 4.04E-03 | 3.74E-01 |
| NP | 0.485 | Upregulated | 3.31E-03 | 3.74E-01 |
| ALDH1A1 | 0.487 | Upregulated | 5.06E-04 | 3.20E-01 |
| IGF2BP2 | 0.488 | Upregulated | 3.24E-02 | 4.63E-01 |
| RNF10 | 0.490 | Upregulated | 1.18E-02 | 3.97E-01 |
| PROS1 | 0.491 | Upregulated | 9.10E-03 | 3.90E-01 |
| TSPAN5 | 0.493 | Upregulated | 2.65E-02 | 4.55E-01 |

| | | | | |
|---|---|---|---|---|
| VWF | 0.497 | Upregulated | 2.14E-03 | 3.60E-01 |
| PHOSPHO1 | 0.498 | Upregulated | 9.18E-03 | 3.90E-01 |
| RHAG | 0.498 | Upregulated | 3.80E-02 | 4.81E-01 |
| COL17A1 | 0.503 | Upregulated | 3.26E-04 | 3.13E-01 |
| LGALS3BP | 0.509 | Upregulated | 2.24E-03 | 3.60E-01 |
| HPS1 | 0.512 | Upregulated | 1.59E-02 | 4.19E-01 |
| SESN3 | 0.513 | Upregulated | 1.96E-02 | 4.32E-01 |
| GBP5 | 0.516 | Upregulated | 7.65E-03 | 3.74E-01 |
| AQP10 | 0.516 | Upregulated | 1.71E-02 | 4.24E-01 |
| HBD | 0.519 | Upregulated | 3.48E-02 | 4.69E-01 |
| BCL2L1 | 0.519 | Upregulated | 3.14E-02 | 4.63E-01 |
| TSTA3 | 0.520 | Upregulated | 1.75E-02 | 4.26E-01 |
| FER1L3 | 0.521 | Upregulated | 2.29E-03 | 3.60E-01 |
| SIAH2 | 0.521 | Upregulated | 1.30E-02 | 4.03E-01 |
| ECHDC3 | 0.521 | Upregulated | 1.13E-03 | 3.30E-01 |
| MPP1 | 0.522 | Upregulated | 1.74E-02 | 4.26E-01 |
| VWCE | 0.528 | Upregulated | 3.42E-02 | 4.67E-01 |
| RBM38 | 0.537 | Upregulated | 1.32E-02 | 4.03E-01 |
| MPO | 0.538 | Upregulated | 2.03E-03 | 3.60E-01 |
| MXI1 | 0.544 | Upregulated | 1.55E-02 | 4.16E-01 |
| RP11-529I10.4 | 0.544 | Upregulated | 8.01E-03 | 3.77E-01 |
| EPB49 | 0.546 | Upregulated | 3.09E-02 | 4.63E-01 |
| MYL9 | 0.547 | Upregulated | 6.20E-03 | 3.74E-01 |
| SMARCD3 | 0.553 | Upregulated | 6.10E-04 | 3.20E-01 |
| GSTA5 | 0.554 | Upregulated | 2.59E-02 | 4.52E-01 |
| FAM46C | 0.556 | Upregulated | 7.13E-03 | 3.74E-01 |
| C2 | 0.558 | Upregulated | 3.82E-06 | 4.41E-02 |
| EPB41 | 0.558 | Upregulated | 7.72E-03 | 3.75E-01 |
| OLFM4 | 0.559 | Upregulated | 1.45E-02 | 4.09E-01 |
| CLEC1B | 0.562 | Upregulated | 1.15E-02 | 3.97E-01 |
| ANKRD9 | 0.564 | Upregulated | 4.92E-03 | 3.74E-01 |
| TMEM56 | 0.568 | Upregulated | 1.66E-02 | 4.23E-01 |
| FBXO7 | 0.570 | Upregulated | 1.95E-02 | 4.32E-01 |
| OLR1 | 0.576 | Upregulated | 4.20E-03 | 3.74E-01 |
| HMBS | 0.578 | Upregulated | 2.26E-02 | 4.42E-01 |
| MGC13057 | 0.579 | Upregulated | 1.11E-02 | 3.97E-01 |
| CREG1 | 0.592 | Upregulated | 7.68E-04 | 3.30E-01 |
| NFIX | 0.603 | Upregulated | 5.15E-03 | 3.74E-01 |
| TMEM158 | 0.607 | Upregulated | 1.26E-02 | 4.02E-01 |
| CTSG | 0.607 | Upregulated | 1.12E-02 | 3.97E-01 |
| TCN1 | 0.612 | Upregulated | 1.38E-03 | 3.30E-01 |
| A4GALT | 0.623 | Upregulated | 6.52E-05 | 2.01E-01 |
| AZU1 | 0.626 | Upregulated | 6.86E-04 | 3.20E-01 |
| KEL | 0.638 | Upregulated | 1.29E-03 | 3.30E-01 |
| KRT1 | 0.641 | Upregulated | 4.49E-02 | 4.97E-01 |
| ANKRD22 | 0.647 | Upregulated | 5.31E-03 | 3.74E-01 |
| CAMP | 0.647 | Upregulated | 6.85E-03 | 3.74E-01 |

| | | | | |
|---|---|---|---|---|
| CMBL | 0.658 | Upregulated | 2.56E-03 | 3.71E-01 |
| RHD | 0.662 | Upregulated | 2.09E-02 | 4.38E-01 |
| HEMGN | 0.669 | Upregulated | 2.03E-02 | 4.35E-01 |
| YPEL4 | 0.671 | Upregulated | 3.74E-04 | 3.16E-01 |
| HBE1 | 0.679 | Upregulated | 1.09E-03 | 3.30E-01 |
| KLF1 | 0.680 | Upregulated | 2.84E-03 | 3.74E-01 |
| ALAS2 | 0.684 | Upregulated | 2.54E-02 | 4.52E-01 |
| SLC6A8 | 0.696 | Upregulated | 3.87E-03 | 3.74E-01 |
| EPB42 | 0.704 | Upregulated | 2.18E-02 | 4.40E-01 |
| ITLN1 | 0.706 | Upregulated | 3.29E-03 | 3.74E-01 |
| BATF2 | 0.709 | Upregulated | 1.32E-03 | 3.30E-01 |
| AHSP | 0.715 | Upregulated | 1.28E-02 | 4.02E-01 |
| TMOD1 | 0.717 | Upregulated | 8.24E-03 | 3.80E-01 |
| TRIM58 | 0.720 | Upregulated | 1.20E-02 | 3.97E-01 |
| SELENBP1 | 0.720 | Upregulated | 2.24E-02 | 4.41E-01 |
| MAOA | 0.731 | Upregulated | 6.83E-04 | 3.20E-01 |
| ITGA2B | 0.735 | Upregulated | 2.40E-03 | 3.62E-01 |
| C1QC | 0.745 | Upregulated | 1.35E-03 | 3.30E-01 |
| RAB3IL1 | 0.753 | Upregulated | 2.44E-03 | 3.65E-01 |
| GMPR | 0.755 | Upregulated | 7.37E-03 | 3.74E-01 |
| TNS1 | 0.758 | Upregulated | 7.55E-03 | 3.74E-01 |
| PLEK2 | 0.761 | Upregulated | 9.97E-04 | 3.30E-01 |
| SLC4A1 | 0.766 | Upregulated | 1.36E-02 | 4.03E-01 |
| BPI | 0.774 | Upregulated | 5.63E-04 | 3.20E-01 |
| XK | 0.784 | Upregulated | 3.91E-03 | 3.74E-01 |
| IFIT1L | 0.804 | Upregulated | 2.85E-02 | 4.55E-01 |
| LTF | 0.805 | Upregulated | 2.53E-04 | 2.88E-01 |
| ELANE | 0.811 | Upregulated | 5.94E-04 | 3.20E-01 |
| OSBP2 | 0.820 | Upregulated | 8.51E-03 | 3.82E-01 |
| CEACAM6 | 0.851 | Upregulated | 2.23E-04 | 2.88E-01 |
| DEFA1 | 0.886 | Upregulated | 1.19E-03 | 3.30E-01 |
| C1QB | 0.899 | Upregulated | 1.34E-03 | 3.30E-01 |
| GYPB | 0.902 | Upregulated | 1.04E-02 | 3.97E-01 |
| TMCC2 | 0.955 | Upregulated | 9.51E-04 | 3.30E-01 |
| CEACAM8 | 0.983 | Upregulated | 1.64E-04 | 2.70E-01 |
| DEFA4 | 0.991 | Upregulated | 4.22E-04 | 3.20E-01 |
| DEFA3 | 0.995 | Upregulated | 1.14E-03 | 3.30E-01 |
| LCN2 | 1.014 | Upregulated | 2.74E-06 | 4.41E-02 |
| DEFA1B | 1.014 | Upregulated | 1.10E-03 | 3.30E-01 |
| MMP8 | 1.035 | Upregulated | 9.35E-04 | 3.30E-01 |
| CA1 | 1.304 | Upregulated | 6.80E-04 | 3.20E-01 |
| HLA-DRB5 | 1.564 | Upregulated | 1.44E-02 | 4.09E-01 |

**Table S4a. Boruta results for common genes - male samples**

| Gene | meanImp | medianImp | minImp | maxImp | normHits | decision |
|---|---|---|---|---|---|---|
| GBP6 | 36.430041 | 36.16817 | 33.884335 | 38.65185 | 1 | Confirmed |
| GBP5 | 32.898445 | 33.11192 | 29.787723 | 34.04048 | 1 | Confirmed |
| BATF2 | 22.436913 | 22.49872 | 20.846609 | 23.70502 | 1 | Confirmed |
| ANKRD22 | 18.663626 | 18.54131 | 17.439554 | 20.04337 | 1 | Confirmed |
| SOD2 | 17.373171 | 17.57984 | 15.796878 | 19.30882 | 1 | Confirmed |
| PDCD1LG2 | 14.741752 | 14.87248 | 12.942658 | 16.4788 | 1 | Confirmed |
| FRMD3 | 11.724309 | 11.96173 | 9.826894 | 13.77181 | 1 | Confirmed |
| GADD45G | 12.265104 | 12.33657 | 11.246658 | 13.49147 | 1 | Confirmed |
| GBP1 | 10.8894 | 10.749 | 10.024171 | 12.15226 | 1 | Confirmed |
| GBP4 | 7.998482 | 7.78443 | 6.431567 | 10.73415 | 0.9285714 | Confirmed |

**Table S4b. Boruta results for sex-specific genes - male samples**

| Gene | meanImp | medianImp | minImp | maxImp | normHits | decision |
|---|---|---|---|---|---|---|
| KIFC3 | 13.7423114 | 13.7661855 | 11.84239803 | 15.518722 | 1 | Confirmed |
| FCGR1C | 13.2751834 | 13.3032947 | 11.84503991 | 15.015823 | 1 | Confirmed |
| SOCS1 | 11.9105208 | 11.9562172 | 10.03978019 | 14.158947 | 1 | Confirmed |
| PROS1 | 12.1514147 | 12.2083367 | 10.09664077 | 13.775919 | 0.997493734 | Confirmed |
| WARS | 10.9826507 | 11.0512474 | 9.0270406 | 12.870868 | 0.997493734 | Confirmed |
| CD274 | 10.8463748 | 10.8553626 | 9.25092208 | 12.693105 | 0.997493734 | Confirmed |
| GBP2 | 10.6806379 | 10.6751822 | 8.70111204 | 12.557195 | 1 | Confirmed |
| SLAMF8 | 10.8908612 | 10.910814 | 8.54187648 | 12.53241 | 0.997493734 | Confirmed |
| FCGR1A | 10.5550541 | 10.6064497 | 8.46363066 | 12.517114 | 0.997493734 | Confirmed |
| MYOF | 10.1467587 | 10.1767069 | 7.89631584 | 12.199222 | 0.997493734 | Confirmed |
| APOL1 | 10.0728922 | 10.0604149 | 7.80906458 | 11.914271 | 0.997493734 | Confirmed |
| FCGR1B | 9.9745861 | 9.9965838 | 7.88826809 | 11.830238 | 0.997493734 | Confirmed |
| MYL9 | 9.3640917 | 9.3972836 | 7.62771473 | 11.714313 | 0.994987469 | Confirmed |
| SPARC | 9.0511233 | 9.0382677 | 7.03217056 | 11.197058 | 0.987468672 | Confirmed |
| SERPING1 | 8.8721939 | 8.8764801 | 6.97103726 | 11.099392 | 0.987468672 | Confirmed |
| LAP3 | 9.4916158 | 9.5223851 | 7.54904156 | 11.024971 | 0.992481203 | Confirmed |
| CXCL9 | 8.6077624 | 8.6373849 | 5.89323125 | 11.003203 | 0.984962406 | Confirmed |
| DDEF2 | 8.8715836 | 8.8703441 | 6.79890421 | 10.877378 | 0.989974937 | Confirmed |
| VAMP5 | 8.8227114 | 8.8008671 | 7.02541283 | 10.71623 | 0.987468672 | Confirmed |
| C2 | 8.5928859 | 8.5785931 | 6.40912149 | 10.708313 | 0.979949875 | Confirmed |
| ODF3B | 8.8085349 | 8.805408 | 6.79686491 | 10.482129 | 0.98245614 | Confirmed |
| FER1L3 | 8.8572169 | 8.8276471 | 6.90082205 | 10.419462 | 0.992481203 | Confirmed |
| GK | 7.6404299 | 7.6828565 | 5.6414001 | 10.054676 | 0.972431078 | Confirmed |
| JAM3 | 7.9677482 | 7.9604766 | 5.80608878 | 9.91267 | 0.974937343 | Confirmed |
| HRK | 7.6632132 | 7.7340345 | 4.44725257 | 9.665635 | 0.972431078 | Confirmed |
| FGL2 | 6.8102704 | 6.8508973 | 4.4240099 | 9.605082 | 0.939849624 | Confirmed |
| SORT1 | 7.7497914 | 7.7423928 | 5.65891843 | 9.504579 | 0.967418546 | Confirmed |
| TNFAIP6 | 7.3223831 | 7.377135 | 5.3946887 | 9.388958 | 0.967418546 | Confirmed |
| SDPR | 6.200698 | 6.2772902 | 3.47737317 | 8.694499 | 0.877192982 | Confirmed |
| RNF13 | 6.3842655 | 6.4089859 | 4.37471317 | 8.503777 | 0.909774436 | Confirmed |
| ABLIM3 | 5.972838 | 6.0339292 | 3.35164787 | 8.212618 | 0.869674185 | Confirmed |
| ETV7 | 5.5653319 | 5.5857173 | 2.74725379 | 7.961999 | 0.844611529 | Confirmed |
| APOL6 | 5.7762142 | 5.8008199 | 3.25924177 | 7.95782 | 0.854636591 | Confirmed |
| EPB41L3 | 5.357002 | 5.3563997 | 3.27871629 | 7.941658 | 0.79197995 | Confirmed |
| NDUFAF3 | 5.7141108 | 5.7418422 | 2.94163112 | 7.827567 | 0.834586466 | Confirmed |
| GRAMD1B | 5.6610826 | 5.6803186 | 3.40818965 | 7.771057 | 0.854636591 | Confirmed |
| KCNJ2 | 5.7823512 | 5.8462969 | 3.21831543 | 7.613731 | 0.86716792 | Confirmed |
| SMARCD3 | 5.8029219 | 5.8245533 | 3.5560115 | 7.584796 | 0.862155388 | Confirmed |
| TRAFD1 | 5.6340612 | 5.6313854 | 3.62304133 | 7.402625 | 0.852130326 | Confirmed |
| C1QA | 4.5626929 | 4.5558747 | 2.10740792 | 7.107393 | 0.65914787 | Confirmed |
| GADD45B | 5.2249677 | 5.2658178 | 3.04043005 | 6.923079 | 0.814536341 | Confirmed |
| TFPI | 4.4593908 | 4.5174967 | 1.34807626 | 7.103957 | 0.644110276 | Confirmed |
| HSPC159 | 4.6866746 | 4.7349762 | 2.34506694 | 7.050752 | 0.664160401 | Confirmed |
| TREML1 | 4.2208338 | 4.2770361 | 0.76505496 | 6.694488 | 0.591478697 | Confirmed |

| | | | | | | |
|---|---|---|---|---|---|---|
| BEST1 | 4.1123988 | 4.109332 | 1.35936912 | 6.353087 | 0.558897243 | Tentative |
| SLC6A12 | 3.8812562 | 3.927102 | 0.76884061 | 6.275805 | 0.493734336 | Tentative |
| CASP5 | 3.620383 | 3.7178368 | 1.08380858 | 6.043437 | 0.446115288 | Tentative |
| CXCL10 | 3.8654004 | 3.9400405 | 0.91517379 | 5.754931 | 0.50877193 | Tentative |
| IL27 | 3.6425078 | 3.6501416 | 1.16609121 | 5.706025 | 0.423558897 | Tentative |
| TYMP | 3.5033282 | 3.5741385 | 1.2822275 | 5.691273 | 0.157894737 | Rejected |
| ALOX12 | 3.5513499 | 3.5733718 | 1.45930796 | 5.48415 | 0.100250627 | Rejected |
| ACRBP | 3.1631896 | 3.2813097 | 0.67740079 | 5.458713 | 0.032581454 | Rejected |
| CTSA | 3.1853256 | 3.2823464 | 0.43635555 | 5.193593 | 0.072681704 | Rejected |
| FBXO6 | 2.486935 | 2.4199874 | -0.23853035 | 5.151331 | 0.01754386 | Rejected |
| NOD2 | 3.2942756 | 3.3017589 | 1.36001389 | 5.133221 | 0.165413534 | Rejected |
| TSPAN9 | 3.1479706 | 3.2056724 | 1.43096014 | 5.060162 | 0.040100251 | Rejected |
| C1QB | 3.0818315 | 3.281218 | 1.06923001 | 4.809772 | 0.037593985 | Rejected |
| C1QC | 2.7101942 | 2.6636039 | 0.86529827 | 4.725247 | 0.007518797 | Rejected |
| MMRN1 | 2.7778813 | 2.8565693 | 1.4589087 | 4.694719 | 0.005012531 | Rejected |
| RTP4 | 2.9109802 | 2.8663263 | 1.59729604 | 4.233369 | 0.015037594 | Rejected |
| ZNF185 | 2.4102534 | 2.266248 | 0.30465821 | 4.170073 | 0.005012531 | Rejected |
| ECGF1 | 2.1268234 | 2.1023419 | 0.03132223 | 4.142763 | 0.005012531 | Rejected |
| SAMD4A | 2.3112892 | 2.4544043 | 0.1313111 | 3.698547 | 0.015037594 | Rejected |
| SCO2 | 1.8509177 | 1.8357731 | 0.44475502 | 3.573217 | 0.005012531 | Rejected |
| TMEM140 | 1.1811508 | 1.1640807 | -0.29128402 | 3.461494 | 0.002506266 | Rejected |
| LRRN3 | 1.8852391 | 1.5765659 | 0.32727006 | 3.344625 | 0.002506266 | Rejected |
| TIMM10 | 1.5728656 | 1.490187 | 0.24295067 | 3.010167 | 0 | Rejected |
| IL1B | 1.7145706 | 1.7823847 | -0.0298767 | 2.711883 | 0 | Rejected |
| GPR42 | 1.6698694 | 1.5879778 | 0.65972905 | 2.661551 | 0 | Rejected |
| TNFSF10 | 0.6374357 | 0.7167922 | -1.55805258 | 2.390167 | 0 | Rejected |
| KLHDC8B | 0.7555988 | 1.0023083 | -0.93463499 | 2.297066 | 0 | Rejected |
| CCR1 | 0.5339866 | 0.4826921 | -0.80006594 | 2.184753 | 0 | Rejected |
| RPS28 | 0.9596018 | 0.9828889 | 0.19427849 | 2.154419 | 0 | Rejected |
| NDUFB9 | 0.2339495 | 0.4643918 | -1.42729811 | 2.13358 | 0 | Rejected |

**Table S4c. Boruta results for combined genes - male samples**

| Gene | meanImp | medianImp | minImp | maxImp | normHits | decision |
|---|---|---|---|---|---|---|
| GBP6 | 21.0591042 | 21.1077323 | 18.07034716 | 22.913909 | 1 | Confirmed |
| GBP5 | 20.05594856 | 20.1598139 | 17.48852589 | 22.048399 | 1 | Confirmed |
| KIFC3 | 12.87088548 | 12.8980546 | 10.71301336 | 14.690633 | 1 | Confirmed |
| BATF2 | 12.60285503 | 12.6159522 | 10.48235625 | 14.271701 | 1 | Confirmed |
| PROS1 | 11.91940358 | 11.8904294 | 8.67069942 | 13.618161 | 1 | Confirmed |
| FCGR1C | 10.26527854 | 10.2651226 | 8.23095922 | 12.284893 | 0.994987469 | Confirmed |
| ANKRD22 | 9.95482226 | 9.9454992 | 7.81321975 | 11.411938 | 1 | Confirmed |
| SOCS1 | 9.31429145 | 9.3205644 | 6.69756722 | 11.168841 | 0.994987469 | Confirmed |
| SLAMF8 | 8.45125622 | 8.4480411 | 6.53395582 | 10.424925 | 0.989974937 | Confirmed |
| MYL9 | 8.73937896 | 8.7649616 | 6.36594818 | 10.27692 | 0.987468672 | Confirmed |
| MYOF | 8.33335071 | 8.3546536 | 6.67546719 | 10.175241 | 0.987468672 | Confirmed |
| CD274 | 8.55019507 | 8.5652764 | 6.13951455 | 10.134296 | 0.987468672 | Confirmed |
| SPARC | 8.2601987 | 8.3075804 | 5.56559004 | 10.00831 | 0.987468672 | Confirmed |
| GBP2 | 7.76708145 | 7.7891517 | 5.71457081 | 9.911327 | 0.979949875 | Confirmed |
| JAM3 | 7.91260858 | 7.9455348 | 5.62856455 | 9.847205 | 0.977443609 | Confirmed |
| WARS | 8.02583078 | 8.0272707 | 6.16719312 | 9.750151 | 0.984962406 | Confirmed |
| FCGR1B | 7.66352864 | 7.6443377 | 6.21743245 | 9.677357 | 0.977443609 | Confirmed |
| PDCD1LG2 | 7.80476155 | 7.8063428 | 5.92614911 | 9.638028 | 0.977443609 | Confirmed |
| GBP1 | 7.92281198 | 7.9714036 | 5.89437781 | 9.564809 | 0.977443609 | Confirmed |
| DDEF2 | 7.60525769 | 7.639157 | 4.86633181 | 9.472605 | 0.964912281 | Confirmed |
| FER1L3 | 7.83592362 | 7.8576333 | 5.62783668 | 9.335835 | 0.974937343 | Confirmed |
| FCGR1A | 7.86392185 | 7.8984521 | 5.76301814 | 9.26466 | 0.974937343 | Confirmed |
| APOL1 | 7.62499851 | 7.6145162 | 6.20716511 | 9.235126 | 0.979949875 | Confirmed |
| C2 | 7.14011975 | 7.1367796 | 5.06083864 | 9.112747 | 0.94235589 | Confirmed |
| ODF3B | 7.60227302 | 7.671909 | 5.52377174 | 9.079185 | 0.977443609 | Confirmed |
| SORT1 | 6.69446895 | 6.7198866 | 5.17219851 | 8.941303 | 0.949874687 | Confirmed |
| CXCL9 | 6.84690205 | 6.8964168 | 4.29716145 | 8.886626 | 0.949874687 | Confirmed |
| HRK | 6.89929369 | 6.9519262 | 4.51329907 | 8.62817 | 0.957393484 | Confirmed |
| SOD2 | 6.56660129 | 6.5864235 | 4.40306038 | 8.619921 | 0.932330827 | Confirmed |
| NDUFAF3 | 6.29430971 | 6.3106083 | 3.76789224 | 8.440785 | 0.914786967 | Confirmed |
| VAMP5 | 6.7642492 | 6.7600441 | 4.86980336 | 8.360734 | 0.957393484 | Confirmed |
| SDPR | 6.21914441 | 6.2073611 | 4.09062557 | 8.283275 | 0.902255639 | Confirmed |
| LAP3 | 6.5949658 | 6.6213908 | 4.58629863 | 8.191734 | 0.932330827 | Confirmed |
| TNFAIP6 | 6.0793558 | 6.1115174 | 4.21710226 | 8.099344 | 0.889724311 | Confirmed |
| GBP4 | 6.2499854 | 6.320346 | 4.16574565 | 8.056874 | 0.90726817 | Confirmed |
| ABLIM3 | 5.46043811 | 5.4589444 | 3.07624266 | 7.807316 | 0.80952381 | Confirmed |
| SMARCD3 | 5.9473975 | 5.9755974 | 3.46077167 | 7.760641 | 0.879699248 | Confirmed |
| EPB41L3 | 5.56883457 | 5.5583376 | 2.35616918 | 7.72732 | 0.822055138 | Confirmed |
| GK | 5.69016122 | 5.6912881 | 4.10992643 | 7.557128 | 0.84962406 | Confirmed |
| RNF13 | 5.20345657 | 5.1757763 | 3.18902885 | 7.534539 | 0.761904762 | Confirmed |
| FGL2 | 5.57858871 | 5.5766524 | 2.96876024 | 7.514407 | 0.819548872 | Confirmed |
| SERPING1 | 6.13438344 | 6.1704168 | 4.31958983 | 7.506117 | 0.894736842 | Confirmed |
| C1QA | 5.01732704 | 5.0131158 | 2.0966777 | 7.477972 | 0.739348371 | Confirmed |
| HSPC159 | 4.98898649 | 4.9763962 | 2.60290003 | 7.373932 | 0.724310777 | Confirmed |
| GADD45G | 5.02574626 | 5.0085982 | 2.35672275 | 7.017517 | 0.724310777 | Confirmed |
| TFPI | 4.22491361 | 4.2080614 | 2.0439666 | 6.906794 | 0.551378446 | Tentative |

| | | | | | | |
|---|---|---|---|---|---|---|
| ALOX12 | 4.35490817 | 4.3779227 | 2.38318778 | 6.787894 | 0.586466165 | Tentative |
| FRMD3 | 4.17302261 | 4.1698504 | 1.65427681 | 6.76567 | 0.551378446 | Tentative |
| ACRBP | 3.40812279 | 3.3616971 | 0.80538216 | 6.660786 | 0.102756892 | Rejected |
| TREML1 | 4.33072677 | 4.4118575 | 1.74922203 | 6.591386 | 0.593984962 | Confirmed |
| APOL6 | 3.96415126 | 4.00281 | 0.72487128 | 6.399519 | 0.478696742 | Tentative |
| KCNJ2 | 4.22116697 | 4.2629804 | 1.46767077 | 6.398678 | 0.553884712 | Tentative |
| GADD45B | 4.17936322 | 4.1595036 | 2.12293602 | 6.201213 | 0.553884712 | Tentative |
| TSPAN9 | 3.70702746 | 3.7883156 | 0.51807443 | 5.816724 | 0.423558897 | Tentative |
| TRAFD1 | 3.97026542 | 4.0060305 | 1.96974143 | 5.71071 | 0.483709273 | Tentative |
| ETV7 | 3.69194388 | 3.7116722 | 0.4716077 | 5.697529 | 0.418546366 | Tentative |
| IL27 | 3.61745219 | 3.6250834 | 0.90373154 | 5.539756 | 0.383458647 | Rejected |
| TYMP | 3.21737112 | 3.2560359 | 0.51269317 | 5.489068 | 0.032581454 | Rejected |
| GRAMD1B | 3.44016057 | 3.5439215 | 1.25176142 | 5.047388 | 0.122807018 | Rejected |
| MMRN1 | 3.22357067 | 3.1923523 | 1.18571398 | 4.964163 | 0.030075188 | Rejected |
| BEST1 | 3.21691455 | 3.3756454 | 1.2142657 | 4.881486 | 0.035087719 | Rejected |
| CASP5 | 3.1989049 | 3.3939014 | 0.5423374 | 4.718511 | 0.035087719 | Rejected |
| CTSA | 2.8668374 | 3.0084272 | -0.10111647 | 4.673721 | 0.010025063 | Rejected |
| NOD2 | 2.59825911 | 2.4646566 | 0.57062586 | 4.577978 | 0.01754386 | Rejected |
| CXCL10 | 2.69657706 | 2.5571427 | 1.61814153 | 4.561261 | 0 | Rejected |
| C1QC | 2.56715271 | 2.6359291 | -0.17837158 | 4.40899 | 0.027568922 | Rejected |
| ECGF1 | 2.05673686 | 2.0968797 | 0.5134093 | 4.161976 | 0 | Rejected |
| SLC6A12 | 2.69816814 | 2.5530537 | 0.87686506 | 3.963211 | 0.027568922 | Rejected |
| IL1B | 1.66995287 | 1.6088321 | -0.43234241 | 3.91904 | 0.002506266 | Rejected |
| C1QB | 2.81213971 | 2.7418013 | 2.05878841 | 3.662479 | 0 | Rejected |
| RTP4 | 1.9651977 | 1.87503 | 0.69128596 | 3.342868 | 0 | Rejected |
| FBXO6 | 2.08008033 | 2.246047 | 0.23908672 | 3.329089 | 0 | Rejected |
| ZNF185 | 2.07112266 | 1.822747 | 1.0174546 | 3.110103 | 0 | Rejected |
| LRRN3 | 1.51037515 | 1.8196787 | -0.93620267 | 2.988466 | 0 | Rejected |
| TIMM10 | 1.31721161 | 1.3254627 | 0.02760877 | 2.759514 | 0 | Rejected |
| SCO2 | 0.98622686 | 0.908268 | -0.66140447 | 2.658617 | 0 | Rejected |
| RPS28 | 0.65056938 | 0.46317 | -0.69402522 | 2.605447 | 0 | Rejected |
| TNFSF10 | 0.20533185 | 0.2683612 | -2.2707891 | 2.576408 | 0 | Rejected |
| GPR42 | 1.37590919 | 1.3488923 | 0.19333521 | 2.534049 | 0 | Rejected |
| SAMD4A | 1.17618153 | 1.2751205 | -0.30877069 | 2.323127 | 0 | Rejected |
| CCR1 | 0.94674676 | 0.9404812 | -1.08427103 | 2.272363 | 0 | Rejected |
| TMEM140 | 0.73327459 | 0.7749705 | -0.70222885 | 2.036472 | 0 | Rejected |
| KLHDC8B | 0.48315048 | 0.4254588 | -1.61896523 | 1.805693 | 0 | Rejected |
| NDUFB9 | 0.03284057 | 0.1438987 | -1.17148559 | 1.172752 | 0 | Rejected |

**Table S4d. Boruta results for common genes - female samples**

| Gene | meanImp | medianImp | minImp | maxImp | normHits | decision |
|---|---|---|---|---|---|---|
| GBP6 | 31.16927593 | 31.0155545 | 28.560777 | 34.311668 | 1 | Confirmed |
| BATF2 | 16.41467897 | 16.3321821 | 13.031598 | 18.602326 | 1 | Confirmed |
| ANKRD22 | 13.49527748 | 13.3876528 | 11.535317 | 15.966719 | 1 | Confirmed |
| FRMD3 | 11.96108918 | 11.8979342 | 8.776709 | 14.805292 | 1 | Confirmed |
| GBP5 | 12.35362323 | 12.2599997 | 9.963999 | 14.545744 | 1 | Confirmed |
| GBP4 | 8.63355725 | 8.6239769 | 4.535197 | 11.774551 | 0.942029 | Confirmed |
| GBP1 | 7.02679106 | 6.8685651 | 4.956052 | 10.08593 | 0.8115942 | Confirmed |
| SOD2 | 6.48985821 | 6.3177521 | 3.281759 | 9.541075 | 0.7536232 | Confirmed |
| PDCD1LG2 | 5.67730769 | 5.809004 | 2.603099 | 8.486239 | 0.6956522 | Confirmed |
| GADD45G | -0.03462151 | 0.1530887 | -1.758906 | 1.614933 | 0 | Rejected |

**Table S4e. Boruta results for sex-specific genes - female samples**

| | meanImp | medianImp | minImp | maxImp | normHits | decision |
|---|---|---|---|---|---|---|
| ALDH1A1 | 16.8815595 | 16.8617371 | 13.9988436 | 20.548959 | 0.99749373 | Confirmed |
| LHFPL2 | 14.7691821 | 14.7951412 | 11.5250642 | 18.087439 | 0.99749373 | Confirmed |
| P2RY13 | 13.7212094 | 13.7990017 | 10.7821527 | 16.293865 | 0.99749373 | Confirmed |
| CELSR3 | 12.3022958 | 12.3740323 | 8.9925967 | 16.164066 | 0.9924812 | Confirmed |
| DHRS9 | 10.8485588 | 10.9036785 | 7.503283 | 13.674193 | 0.98496241 | Confirmed |
| RRAGD | 7.921829 | 7.9713016 | 4.9109907 | 11.246234 | 0.9273183 | Confirmed |
| CARD17 | 7.0666079 | 7.1379962 | 2.742443 | 10.203348 | 0.89473684 | Confirmed |
| TMEM14A | 6.1477214 | 6.1049093 | 2.8107273 | 10.088211 | 0.81704261 | Confirmed |
| IGSF6 | 5.6196674 | 5.7236351 | 1.8039245 | 9.044792 | 0.77192982 | Confirmed |
| TNFAIP2 | 5.445252 | 5.4442419 | 2.5059985 | 8.261963 | 0.73433584 | Confirmed |
| PARM1 | 5.1320935 | 5.1631582 | 2.2102517 | 8.091508 | 0.69172932 | Confirmed |
| CYP4F3 | 4.2745708 | 4.2795314 | 1.542449 | 7.116058 | 0.57393484 | Tentative |
| TSC22D3 | 2.4757996 | 2.4034257 | -0.2247386 | 5.311788 | 0.05012531 | Rejected |
| MBNL2 | 0.6221067 | 0.6377535 | -0.1696499 | 1.600592 | 0 | Rejected |

**Table S4f. Boruta results for combined genes - female samples**

| Gene | meanImp | medianImp | minImp | maxImp | normHits | decision |
|---|---|---|---|---|---|---|
| GBP6 | 24.1296923 | 24.1713267 | 20.3348766 | 27.193772 | 1 | Confirmed |
| ALDH1A1 | 12.2485612 | 12.2840455 | 8.8939713 | 15.918132 | 1 | Confirmed |
| BATF2 | 12.7725872 | 12.8064837 | 10.0108962 | 15.088029 | 1 | Confirmed |
| CELSR3 | 12.3346857 | 12.432846 | 9.6320334 | 14.948774 | 0.99749373 | Confirmed |
| P2RY13 | 11.8249832 | 11.8952972 | 8.9671002 | 13.891542 | 1 | Confirmed |
| GBP5 | 10.1449706 | 10.1553031 | 7.6068356 | 12.511997 | 0.9924812 | Confirmed |
| ANKRD22 | 9.4828046 | 9.5109135 | 7.4865483 | 11.288741 | 0.97994987 | Confirmed |
| FRMD3 | 7.5499419 | 7.5539151 | 3.336931 | 10.883715 | 0.9122807 | Confirmed |
| GBP4 | 7.2809769 | 7.3018039 | 3.9268041 | 10.45151 | 0.9047619 | Confirmed |
| DHRS9 | 7.1624551 | 7.2032987 | 4.223621 | 10.359254 | 0.89473684 | Confirmed |
| TMEM14A | 7.4135144 | 7.4549413 | 4.7307884 | 10.205328 | 0.90225564 | Confirmed |
| LHFPL2 | 7.4477482 | 7.4642773 | 4.0121484 | 9.723971 | 0.91729323 | Confirmed |
| RRAGD | 6.7980836 | 6.889804 | 3.8491001 | 9.499117 | 0.86967419 | Confirmed |
| GBP1 | 6.6943922 | 6.7198926 | 4.2149197 | 9.331664 | 0.86967419 | Confirmed |
| PDCD1LG2 | 4.9883255 | 4.9819221 | 2.1000886 | 8.416868 | 0.67669173 | Confirmed |
| CYP4F3 | 4.7039366 | 4.6924125 | 1.7206855 | 7.747512 | 0.60651629 | Confirmed |
| SOD2 | 3.9038365 | 3.9083087 | 0.6219084 | 6.376139 | 0.45864662 | Tentative |
| IGSF6 | 3.7877642 | 3.8229759 | 1.0185938 | 5.934225 | 0.21553885 | Rejected |
| CARD17 | 3.1379173 | 3.0608477 | 1.074574 | 5.247323 | 0.07518797 | Rejected |
| TNFAIP2 | 2.8921584 | 2.8664704 | 1.0651349 | 5.07394 | 0.04010025 | Rejected |
| PARM1 | 2.263561 | 2.4982048 | 0.4272889 | 3.946367 | 0.02005013 | Rejected |
| TSC22D3 | 2.0442321 | 2.1336036 | -0.7910183 | 3.934948 | 0.02005013 | Rejected |
| GADD45G | 0.5364012 | 0.5709168 | -0.9707828 | 1.970102 | 0 | Rejected |
| MBNL2 | 0.5279839 | 0.629561 | -0.8748642 | 1.63263 | 0 | Rejected |

**Table S5a. Random forest ranking of common genes - male samples**

| Gene | 0 | 1 | MeanDecreaseAccuracy | MeanDecreaseGini |
|---|---|---|---|---|
| GBP6 | 26.85305 | 16.906215 | 31.683838 | 32.034817 |
| GBP5 | 25.513959 | 13.405214 | 29.239414 | 23.569892 |
| BATF2 | 6.789585 | 9.982349 | 13.010232 | 11.081824 |
| ANKRD22 | 1.038613 | 15.142173 | 14.315255 | 7.243359 |
| SOD2 | 7.149043 | 13.11048 | 15.590107 | 6.498087 |
| FRMD3 | 6.850245 | 6.770698 | 9.930025 | 6.168159 |
| GADD45G | 13.153809 | 3.538055 | 13.183132 | 4.942345 |
| PDCD1LG2 | 9.029198 | 4.049255 | 10.471559 | 4.689351 |
| GBP1 | 6.806255 | -1.659798 | 4.35382 | 4.376651 |
| GBP4 | 4.921002 | 1.179467 | 5.008372 | 3.332624 |

**Table S5b. Random forest ranking of sex-specific genes - male samples**

| Gene | 0 | 1 | MeanDecreaseAccuracy | MeanDecreaseGini |
|---|---|---|---|---|
| SLAMF8 | 6.3605154 | 3.3448851 | 7.006609 | 3.885565 |
| GBP2 | 3.5005228 | 5.8255399 | 7.049066 | 3.417864 |
| WARS | 6.7413362 | 4.278484 | 7.573848 | 3.323366 |
| FCGR1C | 5.0188289 | 7.6662371 | 8.813723 | 3.308087 |
| VAMP5 | 6.6913379 | 3.6547438 | 7.405863 | 3.253605 |
| FCGR1B | 4.4480331 | 5.8118602 | 7.222423 | 3.212659 |
| FCGR1A | 4.9202283 | 5.7007277 | 8.015429 | 3.120683 |
| CD274 | 5.2726756 | 5.6349889 | 8.077081 | 3.067025 |
| DDEF2 | 6.527568 | 4.941998 | 8.13851 | 3.01603 |
| GK | 3.1133199 | 7.2957384 | 8.094023 | 2.972188 |
| C2 | 5.940884 | 3.2818771 | 6.74808 | 2.945306 |
| MYOF | 4.8640377 | 4.6300121 | 6.875027 | 2.924669 |
| KIFC3 | 7.549383 | 5.6856915 | 8.150253 | 2.916223 |
| APOL1 | 5.5264492 | 4.661584 | 7.15473 | 2.893536 |
| SOCS1 | 4.7656702 | 5.359928 | 6.627043 | 2.853457 |
| TNFAIP6 | 5.7423804 | 3.2623348 | 6.380741 | 2.750371 |
| PROS1 | 4.8854937 | 5.8256844 | 7.129607 | 2.669923 |
| MYL9 | 3.1639184 | 6.7839227 | 6.872637 | 2.631147 |
| ODF3B | 3.4694077 | 6.1991011 | 7.046895 | 2.604968 |
| FER1L3 | 4.9698424 | 1.4385461 | 4.889136 | 2.42545 |
| GADD45B | 2.0809235 | 3.7943517 | 4.496623 | 2.350756 |
| SPARC | 3.5412059 | 4.3614747 | 5.676885 | 2.344119 |
| SORT1 | 2.8998585 | 4.9782721 | 6.162479 | 2.33675 |
| TRAFD1 | 2.4831494 | 3.6943649 | 4.398027 | 2.322573 |
| JAM3 | 4.4255379 | 3.8030967 | 5.692019 | 2.285653 |
| SERPING1 | 3.5256796 | 5.2961445 | 6.790722 | 2.244593 |
| CXCL9 | 4.0987596 | 3.9640395 | 5.360985 | 2.231984 |
| LAP3 | 5.36533 | 4.2236326 | 6.893851 | 2.095669 |
| SDPR | 2.3610275 | 4.0076426 | 4.332588 | 2.090606 |
| NDUFAF3 | 3.7739105 | 4.5125105 | 5.461892 | 2.079767 |
| GRAMD1B | 3.9616273 | 1.2579091 | 4.207558 | 2.078688 |
| HRK | -0.2366263 | 5.525608 | 4.764051 | 2.028856 |
| KCNJ2 | 3.1211903 | 3.4651567 | 4.497001 | 1.991537 |
| ETV7 | 1.638047 | 3.2799514 | 3.745043 | 1.959777 |
| RNF13 | 4.3697166 | 2.2652666 | 5.172309 | 1.953743 |
| EPB41L3 | 0.9360896 | 3.7964447 | 3.287377 | 1.952763 |
| APOL6 | 5.7823327 | 2.7741646 | 6.321706 | 1.916964 |
| FGL2 | 4.042489 | 3.0079602 | 5.429296 | 1.872934 |
| SMARCD3 | 1.4498418 | 4.6161052 | 4.557262 | 1.861966 |
| C1QA | 1.9521476 | 3.1044028 | 3.618165 | 1.85394 |
| ABLIM3 | 3.3728482 | 0.4161203 | 3.017913 | 1.780005 |

**Table S5c. Random forest ranking of combined genes - male samples**

| Gene | 0 | 1 | MeanDecreaseAccuracy | MeanDecreaseGini |
|---|---|---|---|---|
| GBP5 | 9.493343 | 8.4324664 | 11.83248 | 4.815597 |
| GBP6 | 9.333889 | 8.2579779 | 11.352469 | 4.276445 |
| BATF2 | 7.141352 | 5.6546227 | 9.313102 | 3.851514 |
| FCGR1A | 4.004693 | 4.682695 | 6.434989 | 3.39816 |
| GBP2 | 3.714927 | 3.5694085 | 5.676463 | 3.103526 |
| FCGR1C | 4.526812 | 6.8802443 | 7.915653 | 2.982341 |
| SLAMF8 | 6.364656 | 2.9092666 | 6.932202 | 2.967006 |
| ANKRD22 | 4.874882 | 5.9488969 | 7.643207 | 2.942018 |
| SOCS1 | 4.622636 | 5.4608621 | 6.932436 | 2.876979 |
| WARS | 5.533539 | 2.5173156 | 5.910691 | 2.663352 |
| PDCD1LG2 | 4.335193 | 2.2339723 | 5.33045 | 2.648713 |
| FCGR1B | 5.071644 | 5.7913098 | 7.840718 | 2.572084 |
| MYOF | 4.382513 | 3.3590361 | 5.141536 | 2.558489 |
| GK | 3.323179 | 4.1337222 | 5.6628 | 2.418306 |
| C2 | 5.528623 | 2.5639683 | 6.005299 | 2.413225 |
| KIFC3 | 6.33701 | 6.3562921 | 8.076955 | 2.390207 |
| FER1L3 | 3.923201 | 3.2437218 | 4.908177 | 2.363151 |
| SPARC | 4.911654 | 5.1676307 | 7.223334 | 2.297806 |
| GBP1 | 5.604553 | 2.6501827 | 6.061411 | 2.277794 |
| MYL9 | 2.748259 | 5.8117335 | 5.970987 | 2.274581 |
| PROS1 | 5.533337 | 6.334237 | 7.435457 | 2.252399 |
| APOL1 | 4.119135 | 4.1364483 | 5.864572 | 2.243428 |
| CXCL9 | 4.842872 | 1.3130057 | 4.604544 | 2.209691 |
| CD274 | 3.591547 | 4.794869 | 5.989814 | 2.181649 |
| SERPING1 | 2.783203 | 3.5360439 | 4.800298 | 2.155844 |
| SORT1 | 1.383839 | 3.9272805 | 3.884202 | 1.981258 |
| SDPR | 3.747754 | 3.8264981 | 5.279957 | 1.940164 |
| JAM3 | 5.385311 | 2.759238 | 6.15954 | 1.933827 |
| DDEF2 | 1.582051 | 5.7320056 | 5.351621 | 1.922254 |
| SMARCD3 | 1.122772 | 5.0552614 | 5.116973 | 1.879588 |
| LAP3 | 3.307207 | 4.1761171 | 5.694755 | 1.859777 |
| ODF3B | 2.756347 | 6.5725331 | 6.666332 | 1.821817 |
| GBP4 | 6.50473 | -0.3259609 | 5.502155 | 1.801695 |
| RNF13 | 3.530851 | 3.2194316 | 4.796829 | 1.781405 |
| SOD2 | 1.559944 | 4.3964604 | 4.418699 | 1.778277 |
| NDUFAF3 | 4.677582 | 2.5496125 | 5.422925 | 1.761619 |
| HRK | 1.953235 | 4.2906412 | 4.450087 | 1.725103 |
| TNFAIP6 | 2.88424 | 3.1711778 | 4.179561 | 1.679683 |
| EPB41L3 | 2.030035 | 4.5991194 | 4.850402 | 1.666171 |
| VAMP5 | 4.593342 | -0.5859247 | 4.173354 | 1.644344 |
| ABLIM3 | 3.526601 | 2.5328308 | 3.985526 | 1.576906 |
| FGL2 | 3.412066 | 3.5106891 | 5.115479 | 1.563082 |
| HSPC159 | 1.694498 | 2.1393536 | 2.75876 | 1.475525 |
| C1QA | 3.285017 | 3.0223376 | 4.462128 | 1.423082 |
| GADD45G | 3.348927 | 1.3537912 | 3.298919 | 1.257718 |

**Table S5d. Random forest ranking of common genes - female samples**

| Gene | 0 | 1 | MeanDecreaseAccuracy | MeanDecreaseGini |
|---|---|---|---|---|
| GBP6 | 16.193905 | 16.1555397 | 23.049954 | 14.148175 |
| BATF2 | 4.722679 | 7.5721439 | 9.611073 | 9.938644 |
| ANKRD22 | 6.912866 | 2.8858865 | 7.909797 | 7.677201 |
| GBP1 | 6.558794 | -0.8910952 | 5.100944 | 7.337241 |
| GBP4 | 10.212151 | -6.4484214 | 5.547266 | 6.851643 |
| GBP5 | 6.944235 | 3.0331474 | 8.179992 | 6.747179 |
| FRMD3 | 1.343125 | 9.3992519 | 8.204497 | 6.474825 |
| PDCD1LG2 | 9.003854 | -4.7695464 | 5.029923 | 6.298123 |
| SOD2 | -1.812995 | 6.0518315 | 3.369097 | 6.286328 |

**Table S5e. Random forest ranking of sex-specific genes - female samples**

| Gene | 0 | 1 | MeanDecreaseAccuracy | MeanDecreaseGini |
|---|---|---|---|---|
| ALDH1A1 | 8.307464 | 6.59599 | 10.596599 | 9.559485 |
| LHFPL2 | 6.291049 | 10.881822 | 11.942848 | 8.423035 |
| P2RY13 | 6.676107 | 4.7219377 | 8.805726 | 7.541981 |
| CELSR3 | 11.305437 | 1.8875281 | 9.831399 | 7.213729 |
| DHRS9 | 5.328487 | 3.7678682 | 6.923969 | 6.71582 |
| RRAGD | 5.492999 | 1.3980324 | 5.275592 | 6.527886 |
| TMEM14A | 2.081103 | 5.8816721 | 5.993017 | 5.536784 |
| CARD17 | 5.239523 | 0.8924714 | 4.651436 | 5.341185 |
| PARM1 | 2.010759 | 3.5951981 | 4.315581 | 5.046211 |
| TNFAIP2 | 2.695593 | 2.8608812 | 3.876492 | 5.034461 |
| IGSF6 | 5.039999 | 1.4530001 | 4.923552 | 4.902437 |

**Table S5f. Random forest ranking of combined genes - female samples**

| Gene | 0 | 1 | MeanDecreaseAccuracy | MeanDecreaseGini |
|---|---|---|---|---|
| GBP6 | 24.1417709 | 17.309941 | 30.6415993 | 26.7969 |
| CELSR3 | 11.8208574 | 8.0499654 | 13.5658224 | 7.628859 |
| ALDH1A1 | 11.6986517 | -0.4180516 | 9.4157787 | 4.472258 |
| GBP4 | 8.6802153 | -3.2244696 | 5.3883479 | 4.364962 |
| P2RY13 | 10.6156714 | 6.5432827 | 13.4120234 | 3.747703 |
| BATF2 | 4.4517843 | 1.5779692 | 5.3139543 | 3.611905 |
| FRMD3 | -0.1276316 | 15.5639533 | 13.6788922 | 3.361867 |
| TMEM14A | 1.0814019 | 5.1891336 | 4.8782233 | 3.094295 |
| PDCD1LG2 | 10.5726304 | -7.224671 | 5.8647702 | 2.975651 |
| RRAGD | 4.5821715 | 3.6141681 | 6.4677237 | 2.783188 |
| LHFPL2 | -1.332341 | 0.7714406 | -0.6223811 | 2.33661 |
| ANKRD22 | 5.6896429 | -2.0632446 | 3.977562 | 2.014635 |
| GBP1 | -0.7787344 | 4.8985054 | 3.6829307 | 1.750374 |
| DHRS9 | 2.3843581 | 3.6108281 | 4.3952053 | 1.709423 |
| GBP5 | 2.7897017 | 4.0366888 | 5.3972247 | 1.214465 |

**Table S6. Evaluation of other published pediatric gene signature in male and female samples.**

| Datasets | Age | Genes | All male samples | | | All female samples | | |
|---|---|---|---|---|---|---|---|---|
| | | | AUC | Sensitivity | Specificity | AUC | Sensitivity | Specificity |
| Hill lab (reanalyzed GSE39939 and GSE39940) | Pediatric | 4 (males) 4 (females) | 0.86 | 0.85 | 0.73 | 0.84 | 0.85 | 0.69 |
| Tornheim et al | Pediatric | 71 | 0.73 | 0.67 | 0.72 | 0.75 | 0.64 | 0.79 |
| Anderson et al | Pediatric | 49 | 0.87 | 0.89 | 0.73 | 0.86 | 0.77 | 0.85 |
| Gjoen et al - 2 | Pediatric | 10 | 0.78 | 0.71 | 0.76 | 0.71 | 0.56 | 0.81 |
| Gjoen et al - 1 | Pediatric | 7 | 0.73 | 0.69 | 0.69 | 0.66 | 0.61 | 0.69 |
| Sweeney et al (meta-analysis) | Adult/ Pediatric | 3 | 0.84 | 0.73 | 0.77 | 0.81 | 0.67 | 0.83 |
| Verhagen et al | Pediatric | 5 | 0.41 | 0.01 | 1 | 0.41 | 0.18 | 0.84 |
| Li et al | Pediatric | 1 | 0.49 | 0.41 | 0.64 | 0.46 | 0.26 | 0.82 |

**Table S7. Validation of pediatric, sex-based gene biomarker signature in adult population**

| Dataset | Males | | | Females | | |
|---|---|---|---|---|---|---|
| | AUC | Sensitivity | Specificity | AUC | Sensitivity | Specificity |
| Blankley (GSE83456) | 0.76 | 0.77 | 0.69 | 0.63 | 0.47 | 0.9 |
| Maertzdorf (GSE28623) | 0.69 | 0.52 | 0.94 | 0.76 | 0.57 | 0.86 |
| Walter (GSE73408) | 0.74 | 0.76 | 0.78 | 0.86 | 1 | 0.63 |
| Hoang (GSE144127) | 0.84 | 0.845 | 0.69 | 0.78 | 0.86 | 0.64 |
